# Synthesis, human topoisomerase IIα inhibitory properties and molecular modeling studies of anti-proliferative curcumin mimics†

**DOI:** 10.1039/c9ra05661k

**Published:** 2019-10-21

**Authors:** Nehmedo G. Fawzy, Siva S. Panda, Walid Fayad, ElSayed M. Shalaby, Aladdin M. Srour, Adel S. Girgis

**Affiliations:** Department of Pesticide Chemistry, National Research Centre Dokki Giza 12622 Egypt girgisas10@yahoo.com; Department of Chemistry & Physics, Augusta University Augusta GA 30912 USA; Drug Bioassay-Cell Culture Laboratory, Pharmacognosy Department, National Research Centre Dokki Giza 12622 Egypt; X-Ray Crystallography Lab., Physics Division, National Research Centre Dokki Giza 12622 Egypt; Department of Therapeutic Chemistry, National Research Centre Dokki Giza 12622 Egypt

## Abstract

3,5-Bis(arylidene)-*N*-substituted-4-oxo-piperidine-1-carboxamides 24–51 were synthesized as curcumin mimics in a facile pathway through reaction of 3,5-bis(arylidene)-4-piperidones with the appropriate isocyanate in the presence of triethylamine. The 3*E*,5*E*′-stereochemical configuration was conclusively supported by single crystal X-ray studies of compounds 25 and 34. Most of the synthesized piperidinecarboxamides showed high anti-proliferative properties with potency higher than that of 5-fluorouracil (clinically approved drug against colon, breast and skin cancers) through *in vitro* MTT bio-assay. Some of them revealed anti-proliferative properties at sub-micromolar values (IC_50_ = 0.56–0.70 μM for compounds 29, 30 and 34–38 against HCT116; and IC_50_ = 0.64 μM for compound 30 against A431 cell lines) with promising inhibitory properties against human DNA topoisomerase IIα. The safe profile of the anti-proliferative active agents against the RPE1 normal cell line may prove their selectivity towards carcinoma cells. Robust molecular models (2D-QSAR, 3D-pharmacophore) supported the SAR and validated the observed bio-properties.

## Abstract 2

Piperidinecarboxamides (curcumin mimics) show promising anti-proliferative properties against HCT116 (colon), MCF7 (breast) and A431 (squamous skin) carcinoma cell lines with potency higher than that of 5-fluorouracil.

## Graphical abstract

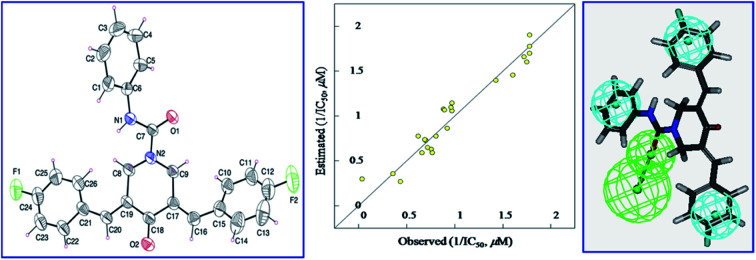

## Introduction

**Fig. 1:**
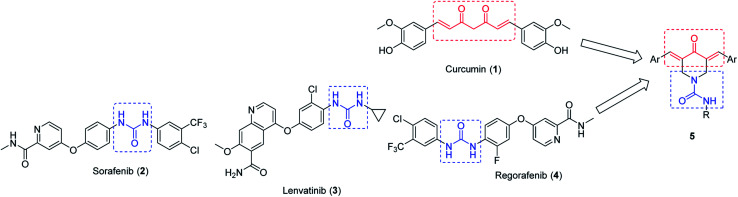
Rational design of 4-piperidone-1-carboxamides 5 as curccumin mimics.

Dienone is an attractive chemical motif utilized by many researchers for designing promising biologically/pharmacologically active agents.^1–4^ Curcumin 1 is one of the most famous dietary natural product dienones isolated from *Curcuma longa* and used in many Asian countries for its anti-inflammatory and wound healing properties (Ayurvedic medicine).^5–7^ Curcumin analogues exhibit an extensive broad spectrum of biological properties such as antibacterial,^8^ anti-tubercular,^9^ anti-HIV,^10^ antioxidant,^11^ antitumor,^7^ and anti-inflammatory^12^ activities and exhibit a potential therapeutic effect on Alzheimer's disease.^4^ Despite the safety profile and broad spectrum biological properties of curcumin, it could not be approved as a therapeutic agent due to its high metabolic instability, low water/plasma solubility and poor systemic bioavailability.^13,14^ The presence of an active methylene group conjugated with the two β-diketones reduce the stability of curcumin.^15–17^ Due to these facts, the present study is directed towards the investigation of novel 4-piperidone-1-carboxamides as curcumin mimics (Fig. 1). In other words, a slight modification of the curcumin pharmacophoric skeleton is considered utilizing only one ketonic function conjugated with the two unsaturated olefinic linkages.

Interest in the piperidone ring system originate from the diverse biological properties showed by 1,3-diarylidene-4-piperidones as antitumor,^18–24^ anti-mycobacterial,^25^ antimalarial^26^ and acetylcholinesterase inhibitor suggesting the usefulness for Alzheimer's disease treatment.^27^ The promising properties of 2,4-bis(arylidene)-8-methyl-8-azabicyclo[3.2.1]octan-3-ones against MCF7 (breast) and HepG2 (liver) carcinoma cell lines also prompted the present study.^28^

Rational for insertion of carboxamide residue at *N*-1 of the targeted 3,5-bis(arylidene)-4-piperidones is stemmed from the fact that many clinically approved cancer drugs possess 1,3-disubstituted urea function of which, Sorafenib 2, (Nexavar, Bayer Healthcare Pharmaceuticals Inc.) that was approved by the U. S. Food and Drug Administration (US-FDA on November 22, 2013) for the treatment of locally recurrent or metastatic, progressive, differentiated thyroid carcinoma along with its previous approval for the treatment of advanced renal cell carcinoma (2005) and advanced hepatocellular carcinoma (2007).^29–31^ Lenvatinib 3, was approved by US-FDA (2015) for the treatment of locally recurrent or metastatic, progressive thyroid cancer and recently in 2016 in combination with everolimus for treatment of advanced renal cancer following one prior anti-angiogenic therapy.^32^ Regorafenib 4, (Stivarga, Bayer HealthCare Pharmaceuticals Inc) approved by US-FDA in 2017 for treatment of hepatocellular carcinoma.^33^

Generally, rational design of the targeted agents 5 can be recognized as molecular conjugation of pharmacophoric units derived from modified curcuminoid scaffold 1 and uranyl fragment which is the bio-active residue of antitumor drugs sorafenib 2, lenvatinib 3 and regorafenib 4 (Fig. 1).

The targeted 4-piperidone-1-carboxamides 5 are screened against colon, breast and skin human carcinoma cell lines. 5-Fluorouracil (injection) is approved by FDA for clinical treatment of colorectal and breast cancer and topically for skin (basal cell) cancer.^34,35^ Additionally, pyrimidine scaffold of 5-fluorouracil can be recognized as bio-isosteric form to the targeted skeleton/ring system (piperidine).^36,37^ For these reasons, 5-fluorouracil is considered as a positive control in the present study.

## Results and discussion

### Chemistry

**Scheme 1:**
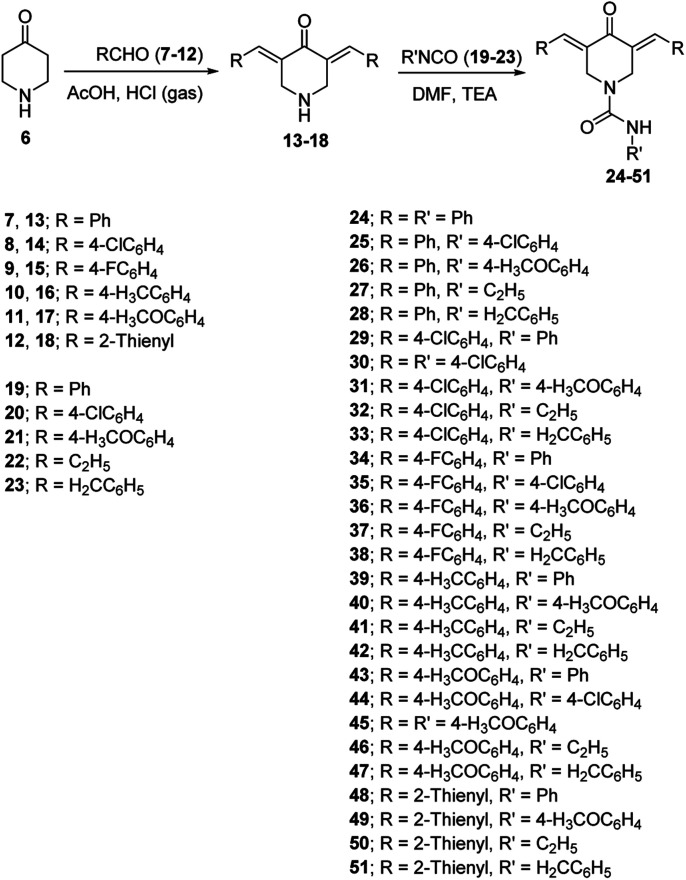
Synthetic route towards 3,5-bis(arylidene)-4-piperidone-1-carboxamides 24–51.

The synthetic pathway for the targeted 3,5-bis(arylidene)-*N*-substituted-4-oxo-piperidine-1-carboxamides 24–51 is depicted in Scheme 1, through reaction of the appropriate 3,5-bis(arylidene)-4-piperidone 13–18 with the corresponding isocyanate 19–23 in *N*,*N*-dimethylformamide (DMF) in the presence of quantitative amount of triethylamine. Spectroscopic (IR, ^1^H-NMR and ^13^C-NMR; ESI Fig. S1–S84†) and elemental analysis data support the structure. IR spectrum of compound 24 (representative of the synthesized family), exhibits the ketonic and amidic carbonyls at *ν* = 1666, 1651 cm^−1^, respectively. The piperidinyl methylene protons are observed as singlet signal at *δ*_H_ = 4.88 in ^1^H-NMR spectrum. The exocyclic olefinic protons are revealed as singlet signal at *δ*_H_ = 7.69 supporting the presence of *E*, *E′*-configuration.^38^^13^C-NMR spectrum of compound 24 shows the amidic and ketonic carbonyl carbons at *δ*_C_ = 155.1 and 186.8, respectively in addition to the piperidinyl methylene carbon at *δ*_C_ = 45.5. Single crystal X-ray studies of compounds 25 and 34 add conclusive support for the structure.

### X-ray crystallography

Compounds 25 and 34 are presented in ESI Fig. S85 and S86,† respectively (ORTEP preview). The two compounds were found to be crystallized in the monoclinic system with space group *C*2/*c* for compound 25 and *P*2_1_/*n* for compound 34 and with one molecule per asymmetric unit cell. The structures are central 4-oxopiperidine-1-carboxamide heterocycle attached at C17 and C19 to two benzylidenes in compound 25 and two 4-fluorobenzylidines in compound 34 forming *E*,*E*′-configuration. The carboxamide group is connected to 4-chlorophenyl and phenyl in compound 25 and 34, respectively. The geometrical parameters of the two compounds were found to be comparable to each other and with similar reported structures.^38,39^ All aryl rings in both compounds are planar as expected. The piperidine ring adopts a half-chair configuration with maximum deviation of 0.379 (3), 0.373 (2) Å at atom N_2_ for compounds 25 and 34, respectively (puckering parameters, *Q* = 0.556 (3), 0.539 (2) Å, Θ = 115.6 (3), 116.1 (3)° and *Φ* = 166.0 (4), 171.1 (2)° for compounds 25 and 34, respectively). In compound 25, the dihedral angles between the least-square plane of the central piperidinyl heterocycle and the phenyl rings (C1–C6, C10–C15 and C21–C26) are 51.89 (13), 47.92 (12) and 26.65 (14)°, respectively, and for compound 34, these dihedral angles are 54.23 (12), 38.27 (12) and 21.31 (14)°, respectively. Molecules in compound 25 are forming a supramolecular chain *via* intermolecular C26–H261⋯O1 interaction (ESI Fig. S87 and Table S1†). Similar supramolecular assembly is formed in compound 34*via* intermolecular C23–H23⋯O1 interaction (ESI Fig. S88 and Table S2†).

### Structure optimization studies

The computational studies in the current study are directed to determine the difference(s) between optimized geometric parameters (utilizing DFT, dentistry function theory technique by B3LYP method with 3-21G* basis set, ESI Fig. S89 and S90†) for compounds 25 and 34 and those experimentally observed (single crystal X-ray data). This will identify the effect(s) of lattice form (solid state) on these parameters.^40,41^ The optimized structures of compounds 25 and 34 reveal bond distances and angles comparable to the experimental values (ESI Tables S3 and S4†). The maximum difference in bond lengths and angles are 0.036 Å, 3.9° and 0.052 Å, 4.2° for compounds 25 and 34, respectively. The root-mean square errors (RMSE) in bond lengths and angles for compounds 25 and 34 are 0.018, 1.602; 0.022 and 1.493, respectively. The central piperidinyl heterocycle of the structures obtained theoretically and experimentally have been superimposed in order to globally compare their conformations (ESI Fig. S91 and S92†). The difference(s) in alignment observed can be explained in terms of crystal packing effects in the solid state which are not present in the gaseous state considered by DFT optimization.

### Anti-proliferative activity

Anti-proliferative properties of the synthesized piperidinecarboxamides 24–51 were investigated against HCT116 (colon), MCF7 (breast) and A431 (squamous skin) carcinoma cell lines by the standard MTT bio-assay utilizing 5-fluorouracil as a reference standard.^28^ From the results observed (Table 1, ESI Fig. S93–S95†), it has been noticed that most of the synthesized piperidinecarboxamides show potent anti-proliferative properties higher than that of 5-fluorouracil (clinically approved drug against colon, breast and skin cancers^34,35^) and curcumin (mimic scaffold). Some of the synthesized compounds reveal anti-proliferative properties at sub-micromolar values (IC_50_ = 0.56–0.70 μM for compounds 29, 30 and 34–38 against HCT116; and IC_50_ = 0.64 μM for compound 30 against A431 cell lines). Based on the anti-proliferative properties observed, structure–activity relationships (SAR) could be attained. Attachment of electron-withdrawing function (*e.g.* chlorine or fluorine) to the phenyl ring of exocyclic olefinic linkages oriented at *C*-3 and *C*-5 of piperidinecarboxamides, enhances the anti-proliferative properties relative to the electron-donating functions (methyl or methoxy). Insertion of benzylidene fragment at *C*-3 and *C*-5 of the targeted piperidones is important for developing antitumor active agents compared to the 2-thienylidene residue. Fluorine containing-compounds reveal higher anti-proliferative efficacy than the chlorine analogues against HCT116 cell line. Meanwhile, better antitumor properties were shown by the chlorine substituted-compounds compared to their fluorine analogues against MCF7 and A431 cell lines [compound 32 (IC_50_ = 2.35 μM) is an exception, exhibiting potency close to the corresponding analogue 37 (IC_50_ = 2.32 μM) against MCF7 cell line. The same observations for compounds 29 and 34 (IC_50_ = 1.29, 1.27 μM, respectively) against A431 cell line].

**Table 1:** Anti-proliferative properties of the synthesized compounds

ID	Compd	IC_50_^a^, *μ*M ± SD (therapeutic index)^b^			
		HCT116	MCF7	A431	RPE1
1	24	1.08 ± 0.18 (15.40)	1.83 ± 0.14 (9.09)	2.49 ± 0.31 (6.68)	16.63 ± 1.57
2	25	1.03 ± 0.29 (21.43)	2.09 ± 0.26 (10.56)	2.51 ± 0.24 (8.79)	22.07 ± 2.06
3	26	1.43 ± 0.20 (12.39)	2.48 ± 0.31 (7.15)	2.83 ± 0.36 (6.26)	17.72 ± 1.98
4	27	1.61 ± 0.14 (<31.06)	2.95 ± 0.24 (<16.95)	2.56 ± 0.29 (<19.53)	>50.00 ± 3.02
5	28	1.31 ± 0.22 (9.13)	2.58 ± 0.30 (4.64)	2.58 ± 0.27 (4.64)	11.96 ± 1.76
6	29	0.70 ± 0.06 (13.81)	1.33 ± 0.24 (7.27)	1.29 ± 0.15 (7.50)	9.67 ± 2.03
7	30	0.58 ± 0.07 (8.43)	1.13 ± 0.29 (4.33)	0.64 ± 0.08 (7.64)	4.89 ± 1.97
8	31	1.03 ± 0.13 (7.28)	1.44 ± 0.19 (5.21)	1.24 ± 0.14 (6.05)	7.50 ± 1.75
9	32	1.04 ± 0.11 (11.61)	2.35 ± 0.23 (5.14)	1.31 ± 0.09 (9.21)	12.07 ± 1.76
10	33	1.13 ± 0.19 (10.10)	2.14 ± 0.26 (5.33)	1.45 ± 0.12 (7.87)	11.41 ± 1.68
11	34	0.56 ± 0.04 (31.25)	2.34 ± 0.29 (7.48)	1.27 ± 0.09 (13.78)	17.50 ± 2.16
12	35	0.56 ± 0.06 (14.75)	1.36 ± 0.17 (6.07)	1.20 ± 0.14 (6.88)	8.26 ± 2.33
13	36	0.62 ± 0.05 (15.26)	1.71 ± 0.12 (5.53)	1.31 ± 0.08 (7.22)	9.46 ± 1.86
14	37	0.57 ± 0.07 (17.16)	2.32 ± 0.30 (4.22)	1.18 ± 0.11 (8.29)	9.78 ± 1.53
15	38	0.56 ± 0.03 (19.41)	2.16 ± 0.15 (5.03)	1.33 ± 0.17 (8.17)	10.87 ± 1.42
16	39	1.44 ± 0.17 (<34.72)	3.96 ± 0.29 (<12.63)	2.57 ± 0.15 (<19.46)	>50.00 ± 3.16
17	40	2.73 ± 0.20 (<18.32)	5.10 ± 0.30 (<9.80)	4.90 ± 0.34 (<10.20)	>50.00 ± 2.96
18	41	2.26 ± 0.23 (8.37)	4.58 ± 0.28 (4.13)	2.64 ± 0.27 (7.16)	18.91 ± 1.33
19	42	22.98 ± 1.82 (2.04)	4.90 ± 0.26 (9.58)	8.75 ± 0.40 (5.37)	46.96 ± 2.17
20	43	1.23 ± 0.15 (<40.65)	3.96 ± 0.23 (<12.63)	2.72 ± 0.20 (<18.38)	>50.00 ± 3.43
21	44	1.11 ± 0.09 (<45.05)	4.17 ± 0.27 (<11.99)	2.85 ± 0.16 (<17.54)	>50.00 ± 2.76
22	45	1.38 ± 0.15 (<36.23)	5.85 ± 0.31 (<8.55)	11.35 ± 1.10 (<4.41)	>50.00 ± 3.76
23	46	1.50 ± 0.14 (31.81)	5.73 ± 0.33 (8.33)	8.83 ± 1.26 (5.40)	47.72 ± 3.05
24	47	1.29 ± 0.19 (<38.76)	4.89 ± 0.25 (<10.22)	7.98 ± 1.34 (<6.27)	>50.00 ± 3.99
25	48	>50.00 ± 1.01 (—)	33.72 ± 1.38 (<1.48)	>50.00 ± 3.65 (—)	>50.00 ± 4.16
26	49	40.83 ± 2.61 (<1.22)	24.68 ± 1.99 (<2.03)	>50.00 ± 2.08 (—)	>50.00 ± 4.22
27	50	20.09 ± 2.44 (2.36)	21.67 ± 2.78 (2.19)	40.63 ± 2.88 (1.17)	47.39 ± 2.66
28	51	>50.00 ± 2.90 (—)	>50.00 ± 3.01 (—)	>50.00 ± 2.38 (—)	>50.00 ± 2.34
29	5-FU^c^	20.43 ± 1.99	3.15 ± 0.44	23.44 ± 2.09	NT^d^
30	Curcumin	38.25 ± 2.36	16.00 ± 2.04	NT^d^	NT^d^

a IC_50_ is the concentration producing 50% inhibition of cell growth relative to the control ± standard deviation (SD).

b Therapeutic index is the IC_50_ in normal cell (RPE1)/IC_50_ in cancer cell.

c 5-FU is 5-fluorouracil (standard reference).

d NT is not tested.

Screening the synthesized piperidinecarboxamides 24–51 against normal (non-cancer) RPE1 (human immortalized retinal pigment epithelial cell line) can prove the selectivity towards carcinoma cells. From the results obtained (Table 1, ESI Fig. S96†), it has been noticed that the promising anti-proliferative agents synthesized have IC_50_ towards RPE1 7–32, 4–11 and 5–14 folds relative to that of HCT116, MCF7 and A431 cell lines, respectively. Additionally, it has been noticed that most of the synthesized agents with methyl/methoxy benzylidenes have high selectivity towards the cancer cell lines relative to the normal/non-cancer cell (therapeutic index).

### Molecular modeling

#### 2D-QSAR study

Correlation between the biological properties and chemical structures can be identified by QSAR (quantitative structure–activity relationship) modeling in terms of physico-chemical parameters (descriptors). QSAR modeling is widely utilized by medicinal chemistry researchers for understanding/determining the rules governing the biological properties, validating the biological observations and predicating promising hits/leads.^42^

#### 2D-QSAR modelling

The synthesized 3,5-bis(arylidene)-*N*-substituted-4-oxo-piperidine-1-carboxamides 24–47 revealing variable anti-proliferative properties were employed for QSAR modeling (CODESSA-Pro software). Promising three descriptor BMLR-QSAR models were obtained with *R*^2^ (squared correlation coefficient) = 0.934, 0.951, 0.901 for HCT116, MCF7 and A431 cell lines, respectively (ESI Tables S5–S7,† Fig. S97–S99†).

#### HCT116 cell line 2D-QSAR modelling

Total molecular 1-center E–E repulsion/# of atoms is the first semi-empirical descriptor of the HCT116 2D-QSAR model (*t*-criterion = 15.070). The descriptor positively participates in the QSAR model determining 1/IC_50_ value in other words, the high descriptor value observes high potent agent as exhibited in compounds 35 and 41 [with descriptor values = 68.54209, 47.42904, respectively (ESI Table S8†)] that reveal estimated IC_50_ values 0.53 and 3.71 μM, respectively (Table 2). Total molecular one-center electron–electron repulsion energy is determined by eqn (1).^43^1
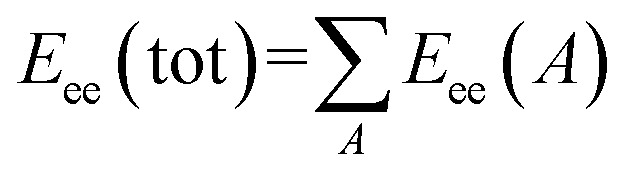
where, A is a given atomic species. *E*_ee_(A) is the electron–electron repulsion energy for atom A.

**Table 2:** Observed and estimated anti-proliferative activity values for the tested piperidinecarboxamides 24–47 according to the BMLR-QSAR models

Entry	Compd	HCT116			MCF7			A431		
		Observed IC_50_, μM	Estimated IC_50_, μM	Error^a^	Observed IC_50_, μM	Estimated IC_50_, μM	Error^a^	Observed IC_50_, μM	Estimated IC_50_, μM	Error^a^
1	24	1.08	1.17	−0.09	1.83	1.39	0.44	2.49	1.94	0.55
2	25	1.03	0.95	0.08	2.09	2.50	−0.41	2.51	2.21	0.30
3	26	1.43	1.39	0.04	2.48	2.50	−0.02	2.83	2.80	0.03
4	27	1.61	1.29	0.32	2.95	2.62	0.33	2.56	4.25	−1.69
5	28	1.31	1.60	−0.29	2.58	2.51	0.07	2.58	2.65	−0.07
6	29	0.70	0.72	−0.02	1.33	1.58	−0.25	1.29	1.08	0.21
7	30	0.58	0.60	−0.02	1.13	0.89	0.24	0.64	0.92	−0.28
8	31	1.03	0.88	0.15	1.44	1.54	−0.10	1.24	1.81	−0.57
9	32	1.04	0.91	0.13	2.35	2.12	0.23	1.31	1.39	−0.08
10	33	1.13	0.93	0.20	2.14	2.00	0.14	1.45	1.76	−0.31
11	34	0.56	0.59	−0.03	2.34	2.07	0.27	1.27	1.00	0.27
12	35	0.56	0.53	0.03	1.36	1.46	−0.10	1.20	1.07	0.13
13	36	0.62	0.69	−0.07	1.71	2.18	−0.47	1.31	0.98	0.33
14	37	0.57	0.63	−0.06	2.32	2.51	−0.19	1.18	1.03	0.15
15	38	0.56	0.56	0.00	2.16	2.53	−0.37	1.33	1.38	−0.05
16	39	1.44	1.36	0.08	3.96	4.46	−0.50	2.57	2.79	−0.22
17	40	2.73	2.87	−0.14	5.10	4.52	0.58	4.90	6.04	−1.14
18	41	2.26	3.71	−1.45	4.58	4.87	−0.29	2.64	3.32	−0.68
19	42	22.98	3.43	19.55	4.90	5.10	−0.20	8.75	6.78	1.97
20	43	1.23	1.29	−0.06	3.96	4.38	−0.42	2.72	3.09	−0.37
21	44	1.11	0.94	0.17	4.17	3.94	0.23	2.85	3.18	−0.33
22	45	1.38	1.55	−0.17	5.85	5.82	0.03	11.35	10.62	0.73
23	46	1.50	1.71	−0.21	5.73	5.13	0.60	8.83	5.63	3.20
24	47	1.29	1.72	−0.43	4.89	4.74	0.15	7.98	6.53	1.45

a Error is the difference between the observed and estimated bio-activity values.

Minimum 1-electron reaction index for atom O is an atomic type descriptor (*t*-criterion = −4.497) participating negatively in the QSAR model (coefficient = −88.6353). Because most of the descriptor values for the tested compounds are with negative sign, the low descriptor value reveals potent anti-proliferative agent as shown for compounds 35 and 41 (descriptor values = −0.00024, −0.00261, respectively). Fukui atomic one-electron reactivity index can be calculated by eqn (2).^43^2
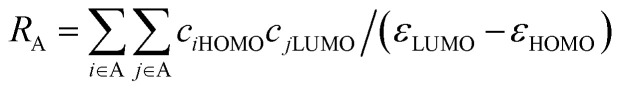
where, *c*_*i*HOMO_ stands for the highest occupied molecular orbital (HOMO) coefficients. *c*_*j*LUMO_ is the lowest unoccupied molecular orbital (LUMO) coefficients. *ε*_LUMO_ is the LUMO energy and *ε*_HOMO_ is for the HOMO energy.

Average information content (order 0) is a topological descriptor. Mean information content index can be calculated by eqn (3).^43^3
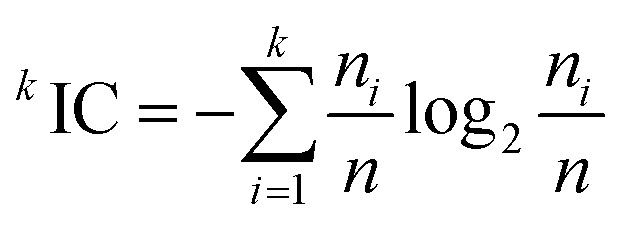
where, *n*_*i*_ is the number of atoms in the *i*^th^ class. *n* is the total number of atoms in the molecule. *k* is the number of atomic layers in the coordination sphere around a given atom that are accounted for.

#### MCF7 cell line 2D-QSAR modelling

FPSA-2 Fractional PPSA (PPSA-2/TMSA) (MOPAC PC) is a geometrical descriptor (*t*-criterion = 16.432) positively participated in the 2D-QSAR model expressing the IC_50_ values *i.e.*, the high descriptor value describes the low potent anti-proliferative agent as shown in compounds 30 and 45 [descriptor values = 1.3958, 2.85685 (ESI Table S9†) for estimated IC_50_ values 0.89, 5.82 μM, respectively (Table 2)]. Fractional total charge weighted partial positive surface area determines by eqn (4).^43^4
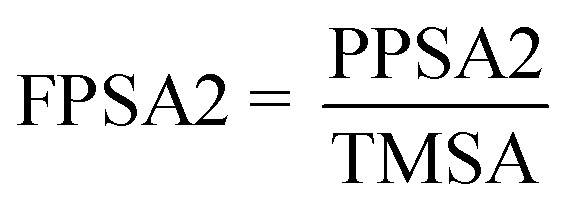
where, PPSA2 is the total weighed partial positively charged molecular surface area. TMSA is the total molecular surface area.

HA dependent HDSA-1 (Zefirov PC) is a charge-related descriptor has a mild power in the QSAR model due to its lowest coefficient value (−0.0318232) among all the descriptors of the attained model. This observation is consistent with the assigned SAR where, attachment of the benzylidene fragment oriented at the *C*-3 and *C*-5 of the constructed piperidones with electron-donating function (methyl or methoxy) decreases the anti-proliferative properties (IC_50_ of compounds 39–47 = 3.96–5.85 μM) relative to the electron-withdrawing function (IC_50_ of compounds 29–38 with chlorine or fluorine atoms = 1.13–2.35 μM). Hydrogen bonding donor ability of the molecule (HDSA1) can be calculated by eqn (5).^43^5
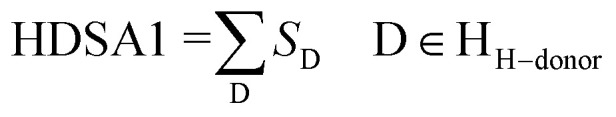
where, *S*_D_ is the solvent accessible surface area of H-bonding donor H atoms.

Partial charged surface area (MOPAC PC) for atom C is also a charge-related descriptor. Although this descriptor has the lowest value among all the descriptors of the 2D-QSAR model attained, it observes high effect to the estimated anti-proliferative properties due to its high coefficient value (−866.471) as shown in compounds 30 and 45 (descriptor values = 0.00317, 0.00256, respectively). Atomic charge weighted partial positively charged surface area and atomic charge weighted partial negatively charged surface area can be calculated by eqn (6) and (7), respectively.^43^6
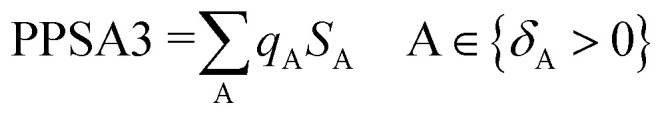
Where, *S*_A_ is the positively charged solvent-accessible atomic surface area and *q*_A_is the atomic partial charge.7
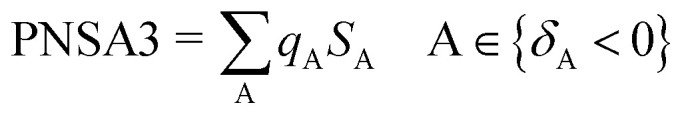
where, *S*_A_ is the negatively charged solvent-accessible atomic surface area and *q*_A_is the atomic partial charge.

#### A431 cell line 2D-QSAR modelling

Count of H-donors sites (MOPAC PC) is a charge-related descriptor with the highest level of significance (*t*-criterion = 10.621) among all the QSAR model descriptors. Although, this descriptor has the lowest coefficient value among all the descriptors of the model (coefficient = 0.068221), it seems one of the most important effective parameters controlling the estimated biological properties as shown by compounds 36 and 45 [descriptor values = 10, 16 (ESI Table S10†) for estimated IC_50_ values 0.98, 10.62 μM, respectively (Table 2)]. This descriptor supports the SAR assumptions regarding the effect of electron-donating groups (methyl or methoxy) *versus* electron-withdrawing elements (fluorine or chlorine) attaching to the exocyclic benzylidene residue of the synthesized piperidones.

Minimum resonance energy for bond H–C is a semi-empirical descriptor can be calculated by eqn (8).^43^8
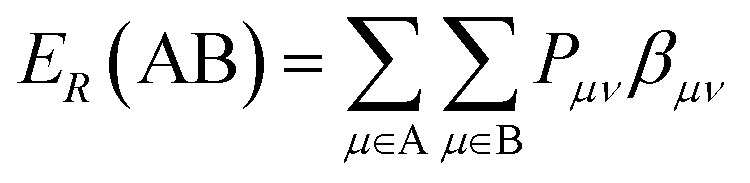
Where, A is a given atomic species. B is another atomic species. *P*_*μν*_ is the density matrix elements over atomic basis {*μν*}. *β*_*μν*_ is the resonance integrals on atomic basis {*μν*}.

Minimum e–n attraction for atom N is also a semi-empirical descriptor. Nuclear-electron attraction energy for a given atomic species can be determined by eqn (9).^43^9
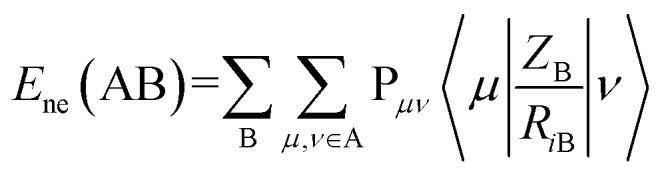
Where, A is a given atomic species, B other atoms. *P*_*μν*_is the density matrix elements over atomic basis {*μν*}. *Z*_B_ is the charge of atomic nucleus B. *R*_*i*B_ is the distance between the electron and atomic nucleus B. 
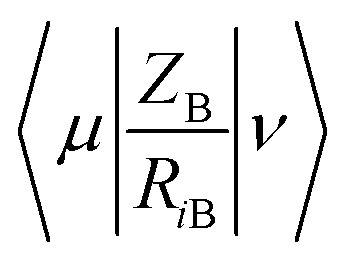
 is the electron-nuclear attraction integrals on atomic basis {*μν*}.

#### Validation of QSAR models

Internal validation technique is the most appropriate for the QSAR studies due to the short data set utilized.^37^ Reliability of the 2D-QSAR models is achieved based on the statistical parameters observed (*R*^2^ = 0.934, 0.951, 0.901; *R*^2^cvOO = 0.905, 0.931, 0.859; *R*^2^cvMO = 0.909, 0.933, 0.861 for HCT116, MCF7 and A431 carcinoma cell lines, respectively). High value differences between Fisher criteria (*F*) and standard deviation (*s*^2^) are also good indications for the goodness of QSAR models (*F* = 93.751, 130.382, 60.784; *s*^2^ = 0.019, 0.125, 0.012 for HCT116, MCF7 and A431 carcinoma cell lines, respectively). Additionally, most of the estimated/predicted biological properties are close to the experimentally observed values (Table 2) including the potent, mild and weak anti-proliferative agents tested.

#### 3D-Pharmacophore modeling

Discovery Studio 2.5 software was utilized for 3D-pharmacophore modeling. 3D-Pharmacophore modeling is an important computational technique used intensively in medicinal chemistry research to explore the parameters controlling biological properties in terms of interaction taking place between variable pharmacophoric features (*e.g.* hydrogen bonding acceptor/donor, hydrophobic, positive/negative ionizable… *etc.*) with functional groups or elemental of bio-active molecules.^44^ Piperidinecarboxamides 24–47 exhibiting variable anti-proliferative properties against the tested cell lines were employed for 3D-pharmacophore modeling. 3D-pharmacophore modeling of the tested compounds 24–47 against HCT116 (colon) carcinoma cell line shows HYPOGEN with 3D-array of four chemical features [three hydrophobics (H-1, H-2, H-3) and one hydrogen bonding acceptor (HBA)] (ESI Fig. S100 and S101†). Typical alignments were observed by all the tested compounds 24–47 in the hypothesized 3D-pharmacophore. The aryl groups of exocyclic olefinic linkages attached to the piperidinyl *C*-3 and *C*-5 are aligned with hydrophobics H-2 and H-3. However, the amidic carbonyl oxygen and the substituent of amidic group are aligned with hydrogen bonding acceptor (HBA) and hydrophobic H-1, respectively (ESI Fig. 102†).

Pharmacophoric model of the anti-proliferative active agents 24–47 against breast (MCF7) carcinoma cell line shows three chemical features [two hydrophobics (H-1, H-2) and one hydrogen bonding acceptor (HBA)] (ESI Fig. S103 and S104†). The substituent of the amidic fragment and aryl group of exocyclic olefinic linkage are fitted with hydrophobics H-1 and H-2, respectively. While the ketonic oxygen gives interaction with HBA for all the tested compounds (ESI Fig. S105†).

Two hydrogen bonding acceptors (HBA-1, HBA-2) and two hydrophobics (H-1, H-2) were viewed by the 3D-pharmacophore of the tested compounds 24–47 against squamous skin (A431) carcinoma cell line (ESI Fig. S106 and S107†). Ketonic and amidic oxygens are fitted with HBA-1 and HBA-2, respectively. However, the exocyclic olefinic linkage and substituent of the amidic fragment are fitted with hydrophobics H-1 and H-2, respectively for all the tested agents (ESI Fig. S108†).

Most of the estimated biological data are correlated with the experimental observations (Table 3). It has been also noticed that all the functions/elements interacted with the pharmacophoric features for all the tested HCT116, MCF7 or A431 cell lines are the controlling parameters governing bio-observations mentioned in SAR. This does not only support the mentioned SAR but also strengthen the assumptions for optimizing more potent anti-proliferative active hits.

**Table 3:** Best fit values and estimated anti-proliferative activity values for the tested piperidinecarboxamides 24–47 according to the 3D-pharmacophore modeling

Entry	Compd	HCT116			MCF7			A431		
		Observed IC_50_, μM	Estimated IC_50_, μM	Fit value	Observed IC_50_, μM	Estimated IC_50_, μM	Fit value	Observed IC_50_, μM	Estimated IC_50_, μM	Fit value
1	24	1.08	0.86	6.951	1.83	2.25	6.069	2.49	3.77	7.029
2	25	1.03	1.03	6.874	2.09	2.80	5.975	2.51	1.25	7.508
3	26	1.43	1.00	6.885	2.48	2.62	6.002	2.83	1.21	7.524
4	27	1.61	0.69	7.043	2.95	2.64	5.999	2.56	2.58	7.194
5	28	1.31	1.43	6.731	2.58	2.19	6.082	2.58	3.74	7.032
6	29	0.70	0.93	6.917	1.33	2.93	5.954	1.29	2.31	7.242
7	30	0.58	0.72	7.029	1.13	2.59	6.008	0.64	1.56	7.412
8	31	1.03	1.75	6.643	1.44	2.89	5.960	1.24	2.02	7.300
9	32	1.04	2.31	6.521	2.35	3.14	5.924	1.31	1.65	7.387
10	33	1.13	1.27	6.780	2.14	3.08	5.933	1.45	3.86	7.019
11	34	0.56	0.88	6.942	2.34	2.03	6.114	1.27	2.42	7.221
12	35	0.56	0.70	7.040	1.36	1.82	6.162	1.2	1.46	7.442
13	36	0.62	0.62	7.094	1.71	1.84	6.156	1.31	1.84	7.341
14	37	0.57	0.49	7.198	2.32	2.21	6.077	1.18	1.62	7.396
15	38	0.56	0.48	7.203	2.16	2.46	6.030	1.33	1.46	7.440
16	39	1.44	2.05	6.574	3.96	3.74	5.848	2.57	3.62	7.046
17	40	2.73	1.43	6.730	5.1	3.04	5.939	4.9	1.87	7.333
18	41	2.26	2.02	6.581	4.58	3.85	5.836	2.64	3.58	7.052
19	42	22.98	11.50	5.824	4.9	3.21	5.914	8.75	5.18	6.891
20	43	1.23	1.18	6.814	3.96	2.76	5.981	2.72	2.61	7.189
21	44	1.11	2.43	6.500	4.17	3.45	5.883	2.85	1.60	7.401
22	45	1.38	1.19	6.808	5.85	3.10	5.929	11.35	5.11	6.897
23	46	1.50	1.24	6.791	5.73	3.65	5.859	8.83	5.26	6.885
24	47	1.29	2.98	6.411	4.89	3.72	5.850	7.98	3.07	7.118

#### Human DNA topoisomerase IIα inhibitory properties

**Fig. 2:**
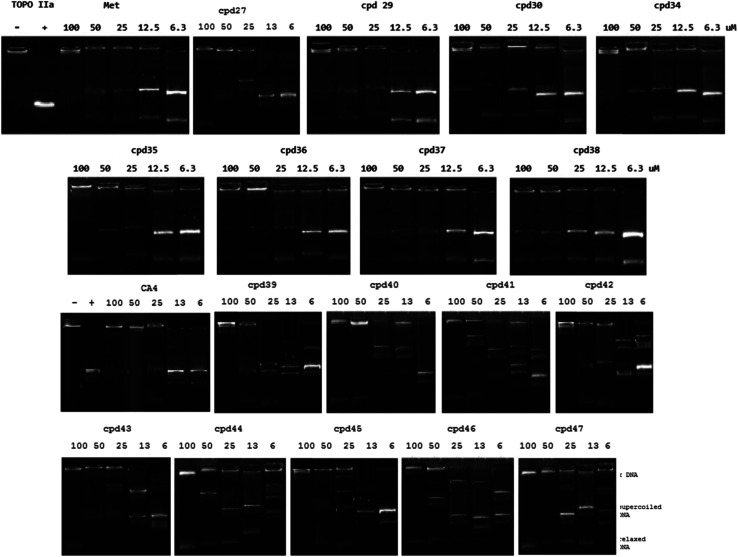
Gel assay for human DNA Topo IIα inhibition by the tested piperidinecarboxamides, Methotrexate (Met) and Combretastatin A4 (CA4).

DNA topoisomerases are the enzymes regulate DNA replication, transcription and repair. Inhibitors of topoisomerases I and II (Topo I, II) are effectively used as anticancer agents.^45^ The inhibitory properties of the promising anti-proliferative agents synthesized (29, 30 and 34–38) and those exhibited high safety profile against RPE1 (non-cancer cell line) relative to the tested cancer cell (27, 39–47), against human DNA topoisomerase IIα were investigated. Reports described the topoisomerase IIα inhibitory properties of 1,3-ylidene-4-piperidones encouraged these studies.^21,22^ From the results obtained (Table 4 and Fig. 2), it has been noticed that compound 36 reveals inhibitory properties against the tested enzyme with potency comparable to that of the standard references used (IC_50_ = 23.04, 22.02, 23.25 μM for compound 36, Combretastatin A-4 and Methotrexate, respectively). Promising inhibitory properties were also revealed by compounds 35, 37, 43 and 29 relative to the standard used (IC_50_ = 26.39, 27.23, 28.30, 28.97 μM, respectively). The slight differences shown due to the anti-proliferative properties of the tested compounds relative to their topoisomerase IIα inhibitory properties (Table 1 and 4) can be attributed to the fact that some tested analogues may have functional activities than the adopted topoisomerase IIα inhibitory activity considered in the current study. Generally, the topoisomerase IIα inhibitory properties of the tested compounds support the anti-proliferative properties observed with good sign indication for their mode of action that may assist in developing better hits/leads.

**Table 4:** Inhibitory properties of human DNA topoisomerase IIα for the tested piperidinecarboxamides, Methotrexate and Combretastatin

Entry	Compound	IC_50_ (μM ± SD)^a^
1	27	33.64 ± 1.30
2	29	28.97 ± 1.39
3	30	41.79 ± 2.42
4	34	30.69 ± 1.32
5	35	26.39 ± 1.61
6	36	23.04 ± 1.37
7	37	27.23 ± 1.72
8	38	35.01 ± 2.08
9	39	41.30 ± 1.60
10	40	40.35 ± 1.56
11	41	50.17 ± 1.94
12	42	31.49 ± 1.22
13	43	28.30 ± 1.09
14	44	46.18 ± 1.79
15	45	39.31 ± 1.52
16	46	56.23 ± 2.18
17	47	33.01 ± 1.28
18	Methotrexate (Met)	23.25 ± 1.31
19	Combretastatin A-4 (CA-4)	22.02 ± 0.85

a IC_50_ is the concentration producing 50% inhibition of the tested enzyme, SD is the standard division.

## Conclusion

(3*E*,5*E*)-3,5-Bis(arylidene)-*N*-substituted-4-oxo-piperidine-1-carboxamides 24–51 were synthesized in excellent yield (80–98%) through reaction of the appropriate 3,5-bis(arylidene)-4-piperidone 13–18 with the corresponding isocyanate 19–23 in DMF in the presence of triethylamine. Single crystal X-ray studies of compounds 25, 34 add good support for the geometrical stereoisomerism. Most of the synthesized piperidinecarboxamides show potent anti-proliferative properties higher than that of 5-fluorouracil (standard reference) through *in vitro* MTT testing against HCT116 (colon), MCF7 (breast) and A431 (squamous skin) carcinoma cell lines. Promising inhibitory properties were observed against DNA topoisomerase IIα by the tested anti-proliferative agents synthesized. Robust 2D-QSAR and 3D-pharmacophore modeling support the observed anti-proliferative properties. Eventually, it can be concluded that, the series of synthesized compounds may be considered a promising starting point for design of novel highly effective anti-proliferative small molecules based on the high biological activity exhibited towards cancer cells and safety profile against normal cell studied.

## Experimental section

Melting points were recorded on a Stuart SMP3 melting point apparatus. IR spectra (KBr) were recorded on a Shimadzu FT-IR 8400S spectrophotometer. NMR spectra (DMSO-*d*_6_) were taken in a Bruker 500 (^1^H: 500, ^13^C: 125 MHz) spectrometer. The starting compounds 13–18 ^46,47^ were prepared according to the reported procedures.

### Synthesis of 3,5-bis(arylidene)-*N*-substituted-4-oxo-piperidine-1-carboxamides 24–51 (general procedure)

A mixture of equimolar amounts of the appropriate 3,5-bis(arylidene)-4-piperidone 13–18 (2.5 mmol) and the corresponding isocyanate 19–23 in *N*,*N*-dimethylformamide (10 ml) containing triethylamine (2.5 mmol) was stirred at room temperature (25–30 °C) for the appropriate time (TLC control). The separated solid upon pouring the reaction mixture into water (200 ml) containing sodium chloride (1 g) was collected, washed with water and crystallized from a suitable solvent affording the corresponding 24–51.

### 3,5-Di[(*E*)-benzylidene]-4-oxo-*N*-phenylpiperidine-1-carboxamide (24)

Obtained from reaction of 13 and 19. Reaction time 10 h. Yellow microcrystals from *n*-butanol, mp 179–181 °C, yield 94% (0.93 g). IR: *ν*_max_/cm^−1^ 1666, 1651, 1605, 1535. ^1^H-NMR *δ* (ppm): 4.88 (s, 4H, 2 NCH_2_), 6.92 (t, *J* = 7.0 Hz, 1H, arom. H), 7.18 (t, *J* = 7.2 Hz, 2H, arom. H), 7.29 (d, *J* = 7.7 Hz, 2H, arom. H), 7.46–7.53 (m, 6H, arom. H), 7.60 (d, *J* = 7.1 Hz, 4H, arom. H), 7.69 (s, 2H, 2 olefinic CH), 8.89 (s, 1H, NH). ^13^C-NMR *δ* (ppm): 45.5 (N*C*H_2_), 119.9, 122.1, 128.3, 128.8, 129.5, 130.7, 133.1, 134.4, 135.8, 140.0 (arom. *C* + olefinic *C*), 155.1 (amidic *C*O), 186.8 (ketonic *C*O). Elemental analysis: C_26_H_22_N_2_O_2_ (394.47) required C, 79.17; H, 5.62; N, 7.10, found C, 79.34; H, 5.71; N, 7.29.

### 3,5-Di[(*E*)-benzylidene]-*N*-(4-chlorophenyl)-4-oxopiperidine-1-carboxamide (25)

Obtained from reaction of 13 and 20. Reaction time 10 h. Yellow microcrystals from *n*-butanol, mp 186–188 °C, yield 80% (0.86 g). IR: *ν*_max_/cm^−1^ 1674, 1655, 1609, 1578. ^1^H-NMR *δ* (ppm): 4.87 (s, 4H, 2 NCH_2_), 7.22 (d, *J* = 7.9 Hz, 2H, arom. H), 7.32 (d, *J* = 7.8 Hz, 2H, arom. H), 7.46–7.53 (m, 6H, arom. H), 7.59 (d, *J* = 7.0 Hz, 4H, arom. H), 7.67 (s, 2H, 2 olefinic CH), 9.01 (s, 1H, NH). ^13^C-NMR *δ* (ppm): 45.5 (N*C*H_2_), 121.3, 125.7, 128.1, 128.8, 129.5, 130.7, 133.0, 134.4, 135.8, 139.0 (arom. *C* + olefinic *C*), 154.9 (amidic *C*O), 186.7 (ketonic *C*O). Elemental analysis: C_26_H_21_ClN_2_O_2_ (428.92) required C, 72.81; H, 4.94; N, 6.53, found C, 72.89; H, 5.00; N, 6.64.

### 3,5-Di[(*E*)-benzylidene]-*N*-(4-methoxyphenyl)-4-oxopiperidine-1-carboxamide (26)

Obtained from reaction of 13 and 21. Reaction time 12 h. Yellow microcrystals from *n*-butanol, mp 184–186 °C, yield 94% (1.00 g). IR: *ν*_max_/cm^−1^ 1670, 1624, 1612, 1531. ^1^H-NMR *δ* (ppm): 3.67 (s, 3H, OCH_3_), 4.85 (s, 4H, 2 NCH_2_), 6.77 (d, *J* = 7.9 Hz, 2H, arom. H), 7.16 (d, *J* = 8.0 Hz, 2H, arom. H), 7.46–7.53 (m, 6H, arom. H), 7.59 (d, *J* = 7.3 Hz, 4H, arom. H), 7.67 (s, 2H, 2 olefinic CH), 8.71 (s, 1H, NH). ^13^C-NMR *δ* (ppm): 45.4 (N*C*H_2_), 55.1 (O*C*H_3_), 113.5, 122.0, 128.8, 129.5, 130.7, 132.8, 133.2, 134.4, 135.7, 154.7 (arom. *C* + olefinic *C*), 155.3 (amidic *C*O), 186.9 (ketonic *C*O). Elemental analysis: C_27_H_24_N_2_O_3_ (424.50) required C, 76.40; H, 5.70; N, 6.60, found C, 76.63; H, 5.79; N, 6.66.

### 3,5-Di[(*E*)-benzylidene]-*N*-ethyl-4-oxopiperidine-1-carboxamide (27)

Obtained from reaction of 13 and 22. Reaction time 24 h. Yellow microcrystals from benzene, mp 172–174 °C, yield 92% (0.80 g). IR: *ν*_max_/cm^−1^ 1674, 1620, 1535, 1447. ^1^H-NMR *δ* (ppm): 0.92 (t, *J* = 6.8 Hz, 3H, CH_3_), 2.97 (quintet, *J* = 6.4 Hz, 2H, N*CH*_*2*_CH_3_), 4.71 (s, 4H, 2 NCH_2_), 6.82 (s, 1H, NH), 7.44–7.52 (m, 6H, arom. H), 7.57 (d, *J* = 7.3 Hz, 4H, arom. H), 7.63 (s, 2H, 2 olefinic CH). ^13^C-NMR *δ* (ppm): 15.4 (*C*H_3_), 35.0 (N*C*H_2_CH_3_), 45.0 (N*C*H_2_), 128.7, 129.4, 130.7, 133.3, 134.5, 135.4 (arom. *C* + olefinic *C*), 157.0 (amidic *C*O), 187.0 (ketonic *C*O). Elemental analysis: C_22_H_22_N_2_O_2_ (346.43) required C, 76.28; H, 6.40; N, 8.09, found C, 76.39; H, 6.54; N, 7.95.

### *N*-Benzyl-3,5-di[(*E*)-benzylidene]-4-oxopiperidine-1-carboxamide (28)

Obtained from reaction of 13 and 23. Reaction time 24 h. Pale yellow microcrystals from benzene, mp 163–165 °C, yield 98% (1.00 g). IR: *ν*_max_/cm^−1^ 1651, 1605, 1574, 1531. ^1^H-NMR *δ* (ppm): 4.16 (s, 2H, PhCH_2_), 4.78 (s, 4H, 2 NCH_2_), 7.12–7.52 (m, 12H, 11 arom. H + NH), 7.58 (d, *J* = 7.2 Hz, 4H, arom. H), 7.65 (s, 2H, 2 olefinic CH). ^13^C-NMR *δ* (ppm): 43.4 (Ph*C*H_2_), 45.2 (N*C*H_2_), 126.4, 126.8, 128.0, 128.7, 129.4, 130.7, 133.3, 134.5, 135.6, 140.6 (arom. *C* + olefinic *C*), 157.3 (amidic *C*O), 186.9 (ketonic *C*O). Elemental analysis: C_27_H_24_N_2_O_2_ (408.50) required C, 79.39; H, 5.92; N, 6.86, found C, 79.59; H, 6.11; N, 7.03.

### 3,5-Bis[(*E*)-4-chlorobenzylidene]-4-oxo-*N*-phenylpiperidine-1-carboxamide (29)

Obtained from reaction of 14 and 19. Reaction time 12 h. Pale yellow microcrystals from *n*-butanol, mp 205–207 °C, yield 85% (0.99 g). IR: *ν*_max_/cm^−1^ 1651, 1605, 1535, 1489. ^1^H-NMR *δ* (ppm): 4.84 (s, 4H, 2 NCH_2_), 6.92 (t, *J* = 7.1 Hz, 1H, arom. H), 7.18 (t, *J* = 7.4 Hz, 2H, arom. H), 7.28 (d, *J* = 7.7 Hz, 2H, arom. H), 7.50–7.65 (m, 10H, 8 arom. H + 2 olefinic CH), 8.87 (s, 1H, NH). ^13^C-NMR *δ* (ppm): 45.5 (N*C*H_2_), 120.0, 122.2, 128.3, 128.7, 128.8, 132.2, 132.4, 132.5, 133.3, 133.6, 133.7, 134.2, 134.6, 136.4, 139.9 (arom. *C* + olefinic *C*), 155.1 (amidic *C*O), 186.5 (ketonic *C*O). Elemental analysis: C_26_H_20_Cl_2_N_2_O_2_ (463.36) required C, 67.40; H, 4.35; N, 6.05, found C, 67.27; H, 4.28; N, 6.17.

### 3,5-Bis[(*E*)-4-chlorobenzylidene]-*N*-(4-chlorophenyl)-4-oxopiperidine-1-carboxamide (30)

Obtained from reaction of 14 and 20. Reaction time 12 h. Pale yellow microcrystals from *n*-butanol, mp 185–187 °C, yield 87% (1.08 g). IR: *ν*_max_/cm^−1^ 1655, 1597, 1558, 1516. ^1^H-NMR *δ* (ppm): 4.83 (s, 4H, 2 NCH_2_), 7.23 (d, *J* = 7.8 Hz, 2H, arom. H), 7.31 (d, *J* = 7.8 Hz, 2H, arom. H), 7.51–7.65 (m, 10H, 8 arom. H + 2 olefinic CH), 9.00 (s, 1H, NH). ^13^C-NMR *δ* (ppm): 45.4 (N*C*H_2_), 121.3, 125.8, 128.2, 128.7, 128.8, 132.2, 132.36, 132.45, 133.2, 133.5, 133.69, 133.72, 134.2, 134.6, 136.5, 138.9 (arom. *C* + olefinic *C*), 154.9 (amidic *C*O), 186.4 (ketonic *C*O). Elemental analysis: C_26_H_19_Cl_3_N_2_O_2_ (497.80) required C, 62.73; H, 3.85; N, 5.63, found C, 62.61; H, 3.71; N, 5.47.

### 3,5-Bis[(*E*)-4-chlorobenzylidene]-*N*-(4-methoxyphenyl)-4-oxopiperidine-1-carboxamide (31)

Obtained from reaction of 14 and 21. Reaction time 12 h. Pale yellow microcrystals from *n*-butanol, mp 210–212 °C, yield 91% (1.12 g). IR: *ν*_max_/cm^−1^ 1670, 1636, 1605, 1589. ^1^H-NMR *δ* (ppm): 3.67 (s, 3H, OCH_3_), 4.81 (s, 4H, 2 NCH_2_), 6.77 (d, *J* = 7.9 Hz, 2H, arom. H), 7.16 (d, *J* = 7.9 Hz, 2H, arom. H), 7.51–7.64 (m, 10H, 8 arom. H + 2 olefinic CH), 8.69 (s, 1H, NH). ^13^C-NMR *δ* (ppm): 45.4 (N*C*H_2_), 55.1 (O*C*H_3_), 113.5, 122.1, 128.7, 128.8, 132.2, 132.4, 132.5, 132.7, 133.3, 133.67, 133.72, 134.2, 134.5, 136.5, 154.8 (arom. *C* + olefinic *C*), 155.3 (amidic *C*O), 186.6 (ketonic *C*O). Elemental analysis: C_27_H_22_Cl_2_N_2_O_3_ (493.38) required C, 65.73; H, 4.49; N, 5.68, found C, 65.64; H, 4.38; N, 5.88.

### 3,5-Bis[(*E*)-4-chlorobenzylidene]-*N*-ethyl-4-oxopiperidine-1-carboxamide (32)

Obtained from reaction of 14 and 22. Reaction time 24 h. Yellow microcrystals from *n*-butanol, mp 211–213 °C, yield 88% (0.92 g). IR: *ν*_max_/cm^−1^ 1670, 1628, 1612, 1535. ^1^H-NMR *δ* (ppm): 0.91 (t, *J* = 6.7 Hz, 3H, CH_3_), 2.95 (quintet, *J* = 6.0 Hz, 2H, N*CH*_2_CH_3_), 4.67 (s, 4H, 2 NCH_2_), 6.80 (s, 1H, NH), 7.55–7.60 (m, 10H, 8 arom. H + 2 olefinic CH). ^13^C-NMR *δ* (ppm): 15.4 (*C*H_3_), 34.9 (N*C*H_2_CH_3_), 44.9 (N*C*H_2_), 128.8, 132.4, 133.3, 133.8, 134.1, 134.3 (arom. *C* + olefinic *C*), 156.9 (amidic *C*O), 186.8 (ketonic *C*O). Elemental analysis: C_22_H_20_Cl_2_N_2_O_2_ (415.31) required C, 63.62; H, 4.85; N, 6.75, found C, 63.81; H, 4.93; N, 6.64.

### *N*-Benzyl-3,5-bis[(*E*)-4-chlorobenzylidene]-4-oxopiperidine-1-carboxamide (33)

Obtained from reaction of 14 and 23. Reaction time 24 h. Yellow microcrystals from *n*-butanol, mp 199–201 °C, yield 92% (1.10 g). IR: *ν*_max_/cm^−1^ 1674, 1628, 1612, 1528. ^1^H-NMR *δ* (ppm): 4.15 (s, 2H, PhCH_2_), 4.74 (s, 4H, 2 NCH_2_), 7.12 (d, *J* = 7.2 Hz, 2H, arom. H), 7.17 (t, *J* = 7.0 Hz, 1H, arom. H), 7.24 (t, *J* = 7.1 Hz, 2H, arom. H), 7.40 (br s, 1H, NH), 7.54 (d, *J* = 7.9 Hz, 4H, arom. H), 7.59 (d, *J* = 7.9 Hz, 4H, arom. H), 7.63 (s, 2H, 2 olefinic CH). ^13^C-NMR *δ* (ppm): 43.4 (Ph*C*H_2_), 45.2 (N*C*H_2_), 126.4, 126.7, 128.0, 128.7, 132.4, 133.3, 133.8, 134.1, 134.4, 140.5 (arom. *C* + olefinic *C*), 157.2 (amidic *C*O), 185.6 (ketonic *C*O). Elemental analysis: C_27_H_22_Cl_2_N_2_O_2_ (477.39) required C, 67.93; H, 4.65; N, 5.87, found C, 68.00; H, 4.56; N, 5.73.

### 3,5-Bis[(*E*)-4-fluorobenzylidene]-4-oxo-*N*-phenylpiperidine-1-carboxamide (34)

Obtained from reaction of 15 and 19. Reaction time 12 h. Pale yellow microcrystals from *n*-butanol, mp 191–193 °C, yield 87% (0.94 g). IR: *ν*_max_/cm^−1^ 1651, 1597, 1566, 1535. ^1^H-NMR *δ* (ppm): 4.85 (s, 4H, 2 NCH_2_), 6.92 (t, *J* = 7.0 Hz, 1H, arom. H), 7.18 (t, *J* = 7.4 Hz, 2H, arom. H), 7.29–7.36 (m, 6H, arom. H), 7.67 (br s, 6H, 4 arom. H + 2 olefinic CH), 8.88 (s, 1H, NH). ^13^C-NMR *δ* (ppm): 45.4 (N*C*H_2_), 115.7, 115.9, 120.0, 122.2, 128.3, 131.0, 132.8, 133.0, 133.1, 134.7, 139.9, 161.5, 163.5 (arom. *C* + olefinic *C*), 155.1 (amidic *C*O), 186.6 (ketonic *C*O). Elemental analysis: C_26_H_20_F_2_N_2_O_2_ (430.45) required C, 72.55; H, 4.68; N, 6.51, found: C, 72.41; H, 4.75; N, 6.59.

### *N*-(4-Chlorophenyl)-3,5-bis[(*E*)-4-fluorobenzylidene]-4-oxopiperidine-1-carboxamide (35)

Obtained from reaction of 15 and 20. Reaction time 12 h. Pale yellow microcrystals from *n*-butanol, mp 192–194 °C, yield 95% (1.10 g). IR: *ν*_max_/cm^−1^ 1655, 1600, 1574, 1504. ^1^H-NMR *δ* (ppm): 4.85 (s, 4H, 2 NCH_2_), 7.22–7.37 (m, 7H, arom. H), 7.55–7.58 (br d, 2H, arom. H), 7.66 (br s, 5H, 3 arom. H + 2 olefinic CH), 9.00 (s, 1H, NH). ^13^C-NMR *δ* (ppm): 45.4 (N*C*H_2_), 115.6, 115.7, 115.9, 119.8, 121.3, 125.7, 128.2, 131.0, 132.6, 132.67, 132.72, 133.1, 134.8, 139.0, 161.5, 163.5 (arom. *C* + olefinic *C*), 154.9 (amidic *C*O), 186.5 (ketonic *C*O). Elemental analysis: C_26_H_19_ClF_2_N_2_O_2_ (464.90) required C, 67.17; H, 4.12; N, 6.03, found C, 67.33; H, 4.17; N, 6.07.

### 3,5-Bis[(*E*)-4-fluorobenzylidene]-*N*-(4-methoxyphenyl)-4-oxopiperidine-1-carboxamide (36)

Obtained from reaction of 15 and 21. Reaction time 12 h. Pale yellow microcrystals from *n*-butanol, mp 189–191 °C, yield 97% (1.12 g). IR: *ν*_max_/cm^−1^ 1670, 1628, 1597, 1504. ^1^H-NMR *δ* (ppm): 3.67 (s, 3H, OCH_3_), 4.82 (s, 4H, 2 NCH_2_), 6.78 (d, *J* = 7.8 Hz, 2H, arom. H), 7.17 (d, *J* = 7.8 Hz, 2H, arom. H), 7.34 (t, *J* = 8.1 Hz, 4H, arom. H) 7.66 (br s, 6H, 4 arom. H + 2 olefinic CH), 8.71 (s, 1H, NH). ^13^C-NMR *δ* (ppm): 45.3 (N*C*H_2_), 55.1 (O*C*H_3_), 113.5, 115.7, 115.9, 122.1, 131.02, 131.04, 132.8, 132.9, 133.0, 133.1, 134.6, 154.8, 161.5, 163.5 (arom. *C* + olefinic *C*), 155.3 (amidic *C*O), 186.7 (ketonic *C*O). Elemental analysis: for C_27_H_22_F_2_N_2_O_3_ (460.48) required C, 70.43; H, 4.82; N, 6.08, found C, 70.49; H, 4.94; N, 6.01.

### *N*-Ethyl-3,5-bis[(*E*)-4-fluorobenzylidene]-4-oxopiperidine-1-carboxamide (37)

Obtained from reaction of 15 and 22. Reaction time 24 h. Pale yellow microcrystals from benzene, mp 180–182 °C, yield 94% (0.90 g). IR: *ν*_max_/cm^−1^ 1674, 1620, 1582, 1543. ^1^H-NMR *δ* (ppm): 0.92 (t, *J* = 6.9 Hz, 3H, CH_3_), 2.96 (quintet, *J* = 6.2 Hz, 2H, N*CH*_*2*_CH_3_), 4.68 (s, 4H, 2 NCH_2_), 6.81 (s, 1H, NH), 7.33 (t, *J* = 8.3 Hz, 4H, arom. H) 7.62 (br d, 6H, 4 arom. H + 2 olefinic CH). ^13^C-NMR *δ* (ppm): 15.4 (*C*H_3_), 34.9 (N*C*H_2_CH_3_), 44.9 (N*C*H_2_), 115.9, 131.1, 133.0, 134.4, 161.5, 163.5 (arom. *C* + olefinic *C*), 156.9 (amidic *C*O), 186.8 (ketonic *C*O). Elemental analysis: C_22_H_20_F_2_N_2_O_2_ (382.41) required C, 69.10; H, 5.27; N, 7.33, found C, 69.15; H, 5.36; N, 7.19.

### *N*-Benzyl-3,5-bis[(*E*)-4-fluorobenzylidene]-4-oxopiperidine-1-carboxamide (38)

Obtained from reaction of 15 and 23. Reaction time 24 h. Pale yellow microcrystals from *n*-butanol, mp 178–180 °C, yield 97% (1.02 g). IR: *ν*_max_/cm^−1^ 1674, 1606, 1582, 1543. ^1^H-NMR *δ* (ppm): 4.17 (s, 2H, PhCH_2_), 4.75 (s, 4H, 2 NCH_2_), 7.13 (d, *J* = 7.2 Hz, 2H, arom. H), 7.17 (t, *J* = 7.1 Hz, 1H, arom. H), 7.24 (t, *J* = 7.2 Hz, 2H, arom. H), 7.33 (t, *J* = 8.3 Hz, 4H, arom. H), 7.41 (br s, 1H, NH), 7.64 (br s, 6H, 4 arom. H + 2 olefinic CH). ^13^C-NMR *δ* (ppm): 43.4 (Ph*C*H_2_), 45.1 (N*C*H_2_), 115.7, 115.8, 126.4, 126.8, 128.0, 131.0, 131.1, 132.99, 133.0, 133.04, 133.1, 134.5, 140.6, 161.5, 163.5 (arom. *C* + olefinic *C*), 157.2 (amidic *C*O), 186.7 (ketonic *C*O). Elemental analysis: C_27_H_22_F_2_N_2_O_2_ (444.48) required C, 72.96; H, 4.99; N, 6.30, found C, 73.12; H, 4.92; N, 6.10.

### 3,5-Bis[(*E*)-4-methylbenzylidene]-4-oxo-*N*-phenylpiperidine-1-carboxamide (39)

Obtained from reaction of 16 and 19. Reaction time 12 h. Pale yellow microcrystals from *n*-butanol, mp 221–223 °C, yield 87% (0.92 g). IR: *ν*_max_/cm^−1^ 1670, 1643, 1601, 1535. ^1^H-NMR *δ* (ppm): 2.37 (s, 6H, 2 ArCH_3_), 4.85 (s, 4H, 2 NCH_2_), 6.91 (t, *J* = 7.1 Hz, 1H, arom. H), 7.18 (t, *J* = 7.3 Hz, 2H, arom. H), 7.28–7.33 (m, 6H, arom. H), 7.49 (d, *J* = 7.5 Hz, 4H, arom. H), 7.64 (s, 2H, olefinic CH), 8.88 (s, 1H, NH). ^13^C-NMR *δ* (ppm): 21.0 (Ar*C*H_3_), 45.5 (N*C*H_2_), 119.9, 122.1, 128.3, 129.4, 130.8, 131.7, 132.3, 135.7, 139.4, 140.0 (arom. *C* + olefinic *C*), 155.1 (amidic *C*O), 186.6 (ketonic *C*O). Elemental analysis: C_28_H_26_N_2_O_2_ (422.53) required C, 79.59; H, 6.20; N, 6.63, found C, 79.66; H, 6.31; N, 6.60.

### *N*-(4-Methoxyphenyl)-3,5-bis[(*E*)-4-methylbenzylidene]-4-oxopiperidine-1-carboxamide (40)

Obtained from reaction of 16 and 21. Reaction time 12 h. Pale yellow microcrystals from *n*-butanol, mp 230–232 °C, yield 98% (1.11 g). IR: *ν*_max_/cm^−1^ 1674, 1628, 1605, 1582. ^1^H-NMR *δ* (ppm): 2.36 (s, 6H, 2 ArCH_3_), 3.67 (s, 3H, OCH_3_), 4.83 (s, 4H, 2 NCH_2_), 6.77 (d, *J* = 7.7 Hz, 2H, arom. H), 7.18 (d, *J* = 7.7 Hz, 2H, arom. H), 7.31 (d, *J* = 7.0 Hz, 4H, arom. H) 7.48 (d, *J* = 7.1 Hz, 4H, rom. H), 7.63 (s, 2H, 2 olefinic CH), 8.71 (s, 1H, NH). ^13^C-NMR *δ* (ppm): 21.0 (Ar*C*H_3_), 45.5 (N*C*H_2_), 55.1 (O*C*H_3_), 113.5, 122.0, 129.4, 130.8, 131.7, 132.4, 132.9, 135.6, 139.4, 154.7 (arom. *C* + olefinic *C*), 155.3 (amidic *C*O), 186.7 (ketonic *C*O). Elemental analysis: C_29_H_28_N_2_O_3_ (452.55) required C, 76.97; H, 6.24; N, 6.19, found C, 76.81; H, 6.05; N, 6.10.

### *N*-Ethyl-3,5-bis[(*E*)-4-methylbenzylidene]-4-oxopiperidine-1-carboxamide (41)

Obtained from reaction of 16 and 22. Reaction time 24 h. Yellow microcrystals from benzene, mp 174–176 °C, yield 88% (0.82 g). IR: *ν*_max_/cm^−1^ 1670, 1628, 1605, 1535. ^1^H-NMR *δ* (ppm): 0.92 (t, *J* = 6.6 Hz, 3H, CH_3_), 2.37 (s, 6H, 2 ArCH_3_), 2.96 (quintet, *J* = 6.3 Hz, 2H, N*CH*_*2*_CH_3_), 4.68 (s, 4H, 2 NCH_2_), 6.79 (s, 1H, NH), 7.31 (d, *J* = 7.2 Hz, 4H, arom. H) 7.45 (d, *J* = 7.3 Hz, 4H, arom. H), 7.58 (s, 2H, 2 olefinic CH). ^13^C-NMR *δ* (ppm): 15.4 (NCH_2_*C*H_3_), 21.0 (Ar*C*H_3_), 34.9 (N*C*H_2_CH_3_), 45.0 (N*C*H_2_), 129.4, 130.8, 131.8, 132.5, 135.4, 139.3 (arom. *C* + olefinic *C*), 157.0 (amidic *C*O), 186.9 (ketonic *C*O). Elemental analysis: C_24_H_26_N_2_O_2_ (374.48) required C, 76.98; H, 7.00; N, 7.48, found C, 77.06; H, 6.94; N, 7.60.

### *N*-Benzyl-3,5-bis[(*E*)-4-methylbenzylidene]-4-oxopiperidine-1-carboxamide (42)

Obtained from reaction of 16 and 23. Reaction time 24 h. Yellow microcrystals from methanol, mp 166–168 °C, yield 92% (1.00 g). IR: *ν*_max_/cm^−1^ 1651, 1601, 1566, 1512. ^1^H-NMR *δ* (ppm): 2.36 (s, 6H, 2 ArCH_3_), 4.17 (s, 2H, PhCH_2_), 4.75 (s, 4H, 2 NCH_2_), 7.14 (d, *J* = 7.2 Hz, 2H, arom. H), 7.17 (d, *J* = 7.1 Hz, 1H, arom. H), 7.24 (t, *J* = 7.2 Hz, 2H, arom. H), 7.31 (d, *J* = 7.5 Hz, 4H, arom. H), 7.40 (br s, 1H, NH), 7.46 (d, *J* = 7.5 Hz, 4H, arom. H), 7.62 (s, 2H, 2 olefinic CH). ^13^C-NMR *δ* (ppm): 21.0 (Ar*C*H_3_), 43.5 (Ph*C*H_2_), 45.2 (N*C*H_2_), 126.4, 126.8, 128.0, 129.4, 130.8, 131.7, 132.5, 135.5, 139.3, 140.6 (arom. *C* + olefinic *C*), 157.2 (amidic *C*O), 186.7 (ketonic *C*O). Elemental analysis: C_29_H_28_N_2_O_2_ (436.56) required C, 79.79; H, 6.47; N, 6.42, found C, 79.92; H, 6.61; N, 6.48.

### 3,5-Bis[(*E*)-4-methoxybenzylidene]-4-oxo-*N*-phenylpiperidine-1-carboxamide (43)

Obtained from reaction of 17 and 19. Reaction time 12 h. Pale yellow microcrystals from *n*-butanol, mp 190–192 °C, yield 93% (1.05 g). IR: *ν*_max_/cm^−1^ 1670, 1643, 1597, 1558. ^1^H-NMR *δ* (ppm): 3.83 (s, 6H, 2 OCH_3_), 4.84 (s, 4H, 2 NCH_2_), 6.91 (t, *J* = 6.5 Hz, 1H, arom. H), 7.07 (d, *J* = 7.6 Hz, 4H, arom. H), 7.18 (t, *J* = 7.0 Hz, 2H, arom. H), 7.30 (d, *J* = 7.7 Hz, 2H, arom. H), 7.56 (d, *J* = 7.7 Hz, 4H, arom. H), 7.62 (s, 2H, olefinic CH), 8.88 (s, 1H, NH). ^13^C-NMR *δ* (ppm): 45.5 (N*C*H_2_), 55.4 (O*C*H_3_), 114.4, 119.9, 122.1, 127.1, 128.3, 131.0, 132.7, 135.4, 140.1, 160.3 (arom. *C* + olefinic *C*), 155.2 (amidic *C*O), 186.4 (ketonic *C*O). Elemental analysis: C_28_H_26_N_2_O_4_ (454.53) required C, 73.99; H, 5.77; N, 6.16, found C, 73.80; H, 5.82; N, 6.19.

### *N*-(4-Chlorophenyl)-3,5-bis[(*E*)-4-methoxybenzylidene]-4-oxopiperidine-1-carboxamide (44)

Obtained from reaction of 17 and 20. Reaction time 12 h. Pale yellow microcrystals from *n*-butanol, mp 185–187 °C, yield 84% (1.02 g). IR: *ν*_max_/cm^−1^ 1663, 1636, 1597, 1566. ^1^H-NMR *δ* (ppm): 3.83 (s, 6H, 2 OCH_3_), 4.84 (s, 4H, 2 NCH_2_), 7.07 (br d, 4H, arom. H), 7.24 (br s, 2H, arom. H), 7.34 (br s, 2H, arom. H) 7.55 (br s, 4H, rom. H), 7.62 (s, 2H, 2 olefinic CH), 9.01 (s, 1H, NH). ^13^C-NMR *δ* (ppm): 45.5 (N*C*H_2_), 55.3 (O*C*H_3_), 114.4, 121.3, 125.6, 127.1, 128.1, 130.8, 132.7, 135.5, 139.1, 160.3 (arom. *C* + olefinic *C*), 154.9 (amidic *C*O), 186.3 (ketonic *C*O). Elemental analysis: C_28_H_25_ClN_2_O_4_ (488.97) required C, 68.78; H, 5.15; N, 5.73, found C, 68.89; H, 5.07; N, 5.92.

### 3,5-Bis[(*E*)-4-methoxybenzylidene]-*N*-(4-methoxyphenyl)-4-oxopiperidine-1-carboxamide (45)

Obtained from reaction of 17 and 21. Reaction time 12 h. Yellow microcrystals from *n*-butanol, mp 212–214 °C, yield 80% (0.97 g). IR: *ν*_max_/cm^−1^ 1670, 1628, 1597, 1558. ^1^H-NMR *δ* (ppm): 3.67 (s, 3H, OCH_3_), 3.83 (s, 6H, 2 OCH_3_), 4.82 (s, 4H, 2 NCH_2_), 6.77 (d, *J* = 8.0 Hz, 2H, arom. H), 7.06 (d, *J* = 7.7 Hz, 4H, arom. H), 7.19 (d, *J* = 8.1 Hz, 2H, arom. H) 7.56 (d, *J* = 8.0 Hz, 4H, rom. H), 7.61 (s, 2H, 2 olefinic CH), 8.71 (s, 1H, NH). ^13^C-NMR *δ* (ppm): 45.4 (N*C*H_2_), 55.1 (O*C*H_3_), 55.3 (O*C*H_3_), 113.5, 114.3, 122.0, 127.1, 131.0, 132.7, 135.3, 155.3, 160.3 (arom. *C* + olefinic *C*), 154.7 (amidic *C*O), 186.5 (ketonic *C*O). Elemental analysis: C_29_H_28_N_2_O_5_ (484.55) required C, 71.88; H, 5.82; N, 5.78, found C, 71.94; H, 5.95; N, 5.86.

### *N*-Ethyl-3,5-bis[(*E*)-4-methoxybenzylidene]-4-oxopiperidine-1-carboxamide (46)

Obtained from reaction of 17 and 22. Reaction time 24 h. Yellow microcrystals from benzene, mp 173–175 °C, yield 98% (0.99 g). IR: *ν*_max_/cm^−1^ 1665, 1620, 1597, 1566. ^1^H-NMR *δ* (ppm): 0.93 (t, *J* = 6.4 Hz, 3H, CH_3_), 2.98 (br s, 2H, N*CH*_*2*_CH_3_), 3.82 (s, 6H, 2 OCH_3_), 4.67 (s, 4H, 2 NCH_2_), 6.81 (s, 1H, NH), 7.05 (d, *J* = 7.9 Hz, 4H, arom. H) 7.52 (d, *J* = 7.9 Hz, 4H, arom. H), 7.57 (s, 2H, 2 olefinic CH). ^13^C-NMR *δ* (ppm): 15.5 (*C*H_3_), 35.0 (N*C*H_2_CH_3_), 44.9 (N*C*H_2_), 55.3 (O*C*H_3_), 114.3, 127.2, 131.2, 132.7, 135.1, 160.2 (arom. *C* + olefinic *C*), 157.0 (amidic *C*O), 186.6 (ketonic *C*O). Elemental analysis: C_24_H_26_N_2_O_4_ (406.48) required C, 70.92; H, 6.45; N, 6.89, found C, 71.06; H, 6.51; N, 6.92.

### *N*-Benzyl-3,5-bis[(*E*)-4-methoxybenzylidene]-4-oxopiperidine-1-carboxamide (47)

Obtained from reaction of 17 and 23. Reaction time 24 h. Yellow microcrystals from *n*-butanol, mp 177–179 °C, yield 85% (1.00 g). IR: *ν*_max_/cm^−1^ 1670, 1628, 1597, 1535. ^1^H-NMR *δ* (ppm): 3.82 (s, 6H, 2 OCH_3_), 4.18 (s, 2H, PhCH_2_), 4.74 (s, 4H, 2 NCH_2_), 7.05 (d, *J* = 7.9 Hz, 4H, arom. H), 7.14–7.18 (m, 3H, arom. H), 7.24 (t, *J* = 7.2 Hz, 2H, arom. H), 7.41 (br s, 1H, NH), 7.54 (d, *J* = 7.9 Hz, 4H, arom. H), 7.60 (s, 2H, 2 olefinic CH). ^13^C-NMR *δ* (ppm): 43.5 (Ph*C*H_2_), 45.2 (N*C*H_2_), 55.3 (O*C*H_3_), 114.3, 126.4, 126.8, 127.1, 128.0, 131.2, 132.7, 135.2, 140.7, 160.2 (arom. *C* + olefinic *C*), 157.2 (amidic *C*O), 186.5 (ketonic *C*O). Elemental analysis: C_29_H_28_N_2_O_4_ (468.55) required C, 74.34; H, 6.02; N, 5.98, found C, 74.55; H, 6.06; N, 5.96.

### (3*E*,5*E*)-4-Oxo-*N*-phenyl-3,5-bis(thiophen-2-ylmethylene)piperidine-1-carboxamide (48)

Obtained from reaction of 18 and 19. Reaction time 12 h. Yellow microcrystals from *n*-butanol, mp 188–190 °C, yield 87% (0.88 g). IR: *ν*_max_/cm^−1^ 1670, 1647, 1589, 1535. ^1^H-NMR *δ* (ppm): 4.86 (s, 4H, 2 NCH_2_), 6.94 (t, *J* = 7.0 Hz, 1H, arom. H), 7.21 (t, *J* = 7.4 Hz, 2H, arom. H), 7.29 (br s, 2H, arom. H), 7.36 (d, *J* = 7.6 Hz, 2H, arom. H), 7.64 (s, 2H, olefinic CH), 7.86 (br s, 2H, arom. H), 7.96 (br s, 2H, arom. H), 9.08 (s, 1H, NH). ^13^C-NMR *δ* (ppm): 46.0 (N*C*H_2_), 120.2, 122.6, 128.4, 128.8, 129.0, 130.3, 132.7, 134.7, 138.2, 140.6 (arom. *C* + olefinic *C*), 156.0 (amidic *C*O), 185.9 (ketonic *C*O). Elemental analysis: C_22_H_18_N_2_O_2_S_2_ (406.52) required C, 65.00; H, 4.46; N, 6.89, found C, 64.93; H, 4.56; N, 6.95.

### (3*E*,5*E*)-*N*-(4-Methoxyphenyl)-4-oxo-3,5-bis(thiophen-2-ylmethylene)piperidine-1-carboxamide (49)

Obtained from reaction of 18 and 21. Reaction time 12 h. Yellow microcrystals from *n*-butanol, mp 194–196 °C, yield 84% (0.92 g). IR: *ν*_max_/cm^−1^ 1667, 1651, 1593, 1535. ^1^H-NMR *δ* (ppm): 3.68 (s, 3H, OCH_3_), 4.83 (s, 4H, 2 NCH_2_), 6.80 (d, *J* = 7.9 Hz, 2H, arom. H), 7.24 (d, *J* = 7.7 Hz, 2H, arom. H), 7.28 (br s, 2H, arom. H), 7.64 (s, 2H, 2 olefinic CH), 7.84 (br s, 2H, arom. H), 7.96 (br s, 2H, arom. H), 8.90 (s, 1H, NH). ^13^C-NMR *δ* (ppm): 45.4 (N*C*H_2_), 55.1 (O*C*H_3_), 113.5, 121.7, 127.9, 128.6, 129.9, 132.2, 133.0, 134.2, 137.7, 154.7 (arom. *C* + olefinic *C*), 155.7 (amidic *C*O), 185.5 (ketonic *C*O). Elemental analysis: C_23_H_20_N_2_O_3_S_2_ (436.54) required C, 63.28; H, 4.62; N, 6.42, found C, 63.12; H, 4.53; N, 6.56.

### (3*E*,5*E*)-*N*-Ethyl-4-oxo-3,5-bis(thiophen-2-ylmethylene)piperidine-1-carboxamide (50)

Obtained from reaction of 18 and 22. Reaction time 24 h. Yellow microcrystals from *n*-butanol, mp 197–199 °C, yield 96% (0.86 g). IR: *ν*_max_/cm^−1^ 1670, 1636, 1589, 1551. ^1^H-NMR *δ* (ppm): 0.97 (t, *J* = 6.5 Hz, 3H, CH_3_), 3.02 (br s, 2H, N*CH*_*2*_CH_3_), 4.69 (s, 4H, 2 NCH_2_), 6.91 (s, 1H, NH), 7.28 (d, *J* = 3.1 Hz, 2H, arom. H), 7.60 (s, 2H, 2 olefinic CH), 7.79 (br s, 2H, arom. H), 7.95 (br s, 2H, arom. H). ^13^C-NMR *δ* (ppm): 15.4 (*C*H_3_), 35.1 (N*C*H_2_CH_3_), 44.9 (N*C*H_2_), 127.6, 128.5, 130.2, 132.1, 134.0, 137.8 (arom. *C* + olefinic *C*), 157.3 (amidic *C*O), 185.7 (ketonic *C*O). Elemental analysis: C_18_H_18_N_2_O_2_S_2_ (358.47) required C, 60.31; H, 5.06; N, 7.81, found C, 60.23; H, 5.21; N, 7.98.

### (3*E*,5*E*)-*N*-Benzyl-4-oxo-3,5-bis(thiophen-2-ylmethylene)piperidine-1-carboxamide (51)

Obtained from reaction of 18 and 23. Reaction time 24 h. Yellow microcrystals from *n*-butanol, mp 175–177 °C, yield 95% (1.00 g). IR: *ν*_max_/cm^−1^ 1663, 1620, 1597, 1566. ^1^H-NMR *δ* (ppm): 4.24 (s, 2H, PhCH_2_), 4.76 (s, 4H, 2 NCH_2_), 7.20–7.28 (m, 7H, arom. H), 7.55 (br s, 1H, NH), 7.61 (s, 2H, 2 olefinic CH), 7.83 (br s, 2H, arom. H), 7.94 (br s, 2H, arom. H). ^13^C-NMR *δ* (ppm): 43.7 (Ph*C*H_2_), 45.1 (N*C*H_2_), 126.4, 126.9, 127.8, 128.1, 128.5, 130.1, 132.1, 134.0, 137.8, 140.6 (arom. *C* + olefinic *C*), 157.5 (amidic *C*O), 185.6 (ketonic *C*O). Elemental analysis: C_23_H_20_N_2_O_2_S_2_ (420.55) required C, 65.69; H, 4.79; N, 6.66, found C, 65.81; H, 4.85; N, 6.52.

### X-ray crystallography

Experimental part of X-ray crystallography is reported in the ESI.†

### *In vitro* antitumor screening

Experimental part of the *in vitro* antitumor screening is reported in the ESI.†

### 2D-QSAR studies

Experimental part of the 2D-QSAR studies is reported in the ESI.†

### Human DNA topoisomerase IIα inhibitory properties

Experimental part of the Human DNA topoisomerase IIα inhibitory properties determination is reported in the ESI.†

## Conflicts of interest

There is no conflict to declare.

## Supplementary Material

RA-009-C9RA05661K-s001

RA-009-C9RA05661K-s002
