# Supplementary material for: Synthesis, human topoisomerase IIα inhibitory properties and molecular modeling studies of anti-proliferative curcumin mimics
Source: RSC Adv. 2019 Oct 21;9(58):33761–74. doi: 10.1039/c9ra05661k (PMC9073595; doi:10.1039/c9ra05661k)
Supplement: RA-009-C9RA05661K-s001 [file RA-009-C9RA05661K-s001.pdf]

Electronic Supplementary Material (ESI) for RSC Advances.
This journal is © The Royal Society of Chemistry 2019

Synthesis, human topoisomerase IIα inhibitory properties and molecular modeling studies of anti-proliferative curcumin mimics

Nehmedo G. Fawzy,[a] Siva S. Panda,[b] Walid Fayad,[c] ElSayed M. Shalaby,[d] Aladdin M. Srour[e] and Adel S. Girgis,*[a]

[a]*Department of Pesticide Chemistry, National Research Centre, Dokki, Giza 12622, Egypt. E-mail:* girgisas10@yahoo.com

[b]*Department of Chemistry & Physics, Augusta University, Augusta, GA 30912, USA.*

[c]*Drug Bioassay-Cell Culture Laboratory, Pharmacognosy Department, National Research Centre, Dokki, Giza, 12622. Egypt.*

[d]*X-Ray Crystallography Lab., Physics Division, National Research Centre, Dokki, Giza 12622, Egypt.*

[e]*Department of Therapeutic Chemistry, National Research Centre, Dokki, Giza 12622, Egypt.*

Supplementary material

Table titles

Table S1. Hydrogen-bond geometry (Å, °) for compound **25**.

Table S2. Hydrogen-bond geometry (Å, °) for compound **34**.

Table S3. Selected intramolecular experimental (X-ray) and computational optimized geometrical parameters (bond lengths, Å) of compounds **25** and **34**.

Table S4. Selected intramolecular experimental (X-ray) and computational optimized geometrical parameters (bond angles, °) of compounds **25** and **34**.

Table S5. Descriptors of the BMLR-QSAR model for the synthesized piperidinecarboxamides **24–47** against HCT116 (colon) carcinoma cell line.

Table S6. Descriptors of the BMLR-QSAR model for the synthesized piperidinecarboxamides **24–47** against MCF7 (breast) carcinoma cell line.

Table S7. Descriptors of the BMLR-QSAR model for the synthesized piperidinecarboxamides **24–47** against A431 (squamous) carcinoma cell line.

Table S8. Molecular descriptor values of the BMLR-QSAR model for the synthesized piperidinecarboxamides **24–47** against HCT116 (colon) carcinoma cell line.

Table S9. Molecular descriptor values of the BMLR-QSAR model for the synthesized piperidinecarboxamides **24–47** against MCF7 (breast) carcinoma cell line.

Table S10. Molecular descriptor values of the BMLR-QSAR model for the synthesized piperidinecarboxamides **24–47** against A431 (squamous) carcinoma cell line.

Table S11. Crystal data and structure refinement parameters for compounds **25** and **34**.

Figure captions

Fig. S1. IR spectrum of compound **24** (KBr pellet).

Fig. S2. ^{1}H-NMR spectrum of compound **24** in DMSO-d_6.

Fig. S3. ^{13}C-NMR spectrum of compound **24** in DMSO-d_6.

Fig. S4. IR spectrum of compound **25** (KBr pellet).

Fig. S5. ^{1}H-NMR spectrum of compound **25** in DMSO-d_6.

Fig. S6. ^{13}C-NMR spectrum of compound **25** in DMSO-d_6.

Fig. S7. IR spectrum of compound **26** (KBr pellet).

Fig. S8. ^{1}H-NMR spectrum of compound **26** in DMSO-d_6.

Fig. S9. ^{13}C-NMR spectrum of compound **26** in DMSO-d_6.

Fig. S10. IR spectrum of compound **27** (KBr pellet).

Fig. S11. ^{1}H-NMR spectrum of compound **27** in DMSO-d_6.

Fig. S12. ^{13}C-NMR spectrum of compound **27** in DMSO-d_6.

Fig. S13. IR spectrum of compound **28** (KBr pellet).

Fig. S14. ^{1}H-NMR spectrum of compound **28** in DMSO-d_6.

Fig. S15. ^{13}C-NMR spectrum of compound **28** in DMSO-d_6.

Fig. S16. IR spectrum of compound **29** (KBr pellet).

Fig. S17. ^{1}H-NMR spectrum of compound **29** in DMSO-d_6.

Fig. S18. ^{13}C-NMR spectrum of compound **29** in DMSO-d_6.

Fig. S19. IR spectrum of compound **30** (KBr pellet).

Fig. S20. ^{1}H-NMR spectrum of compound **30** in DMSO-d_6.

Fig. S21. ^{13}C-NMR spectrum of compound **30** in DMSO-d_6.

Fig. S22. IR spectrum of compound **31** (KBr pellet).

Fig. S23. ^{1}H-NMR spectrum of compound **31** in DMSO-d_6.

Fig. S24. ^{13}C-NMR spectrum of compound **31** in DMSO-d_6.

Fig. S25. IR spectrum of compound **32** (KBr pellet).

Fig. S26. ^{1}H-NMR spectrum of compound **32** in DMSO-d_6.

Fig. S27. ^{13}C-NMR spectrum of compound **32** in DMSO-d_6.

Fig. S28. IR spectrum of compound **33** (KBr pellet).

Fig. S29. ^{1}H-NMR spectrum of compound **33** in DMSO-d_6.

Fig. S30. ^{13}C-NMR spectrum of compound **33** in DMSO-d_6.

Fig. S31. IR spectrum of compound **34** (KBr pellet).

Fig. S32. ^{1}H-NMR spectrum of compound **34** in DMSO-d_6.

Fig. S33. ^{13}C-NMR spectrum of compound **34** in DMSO-d_6.

Fig. S34. IR spectrum of compound **35** (KBr pellet).

Fig. S35. ^{1}H-NMR spectrum of compound **35** in DMSO-d_6.

Fig. S36. ^{13}C-NMR spectrum of compound **35** in DMSO-d_6.

Fig. S37. IR spectrum of compound **36** (KBr pellet).

Fig. S38. ^{1}H-NMR spectrum of compound **36** in DMSO-d_6.

Fig. S39. ^{13}C-NMR spectrum of compound **36** in DMSO-d_6.

Fig. S40. IR spectrum of compound **37** (KBr pellet).

Fig. S41. ^{1}H-NMR spectrum of compound **37** in DMSO-d_6.

Fig. S42. ^{13}C-NMR spectrum of compound **37** in DMSO-d_6.

Fig. S43. IR spectrum of compound **38** (KBr pellet).

Fig. S44. ^{1}H-NMR spectrum of compound **38** in DMSO-d_6.

Fig. S45. ^{13}C-NMR spectrum of compound **38** in DMSO-d_6.

Fig. S46. IR spectrum of compound **39** (KBr pellet).

Fig. S47. ^{1}H-NMR spectrum of compound **39** in DMSO-d_6.

Fig. S48. ^{13}C-NMR spectrum of compound **39** in DMSO-d_6.

Fig. S49. IR spectrum of compound **40** (KBr pellet).

Fig. S50. ^{1}H-NMR spectrum of compound **40** in DMSO-d_6.

Fig. S51. ^{13}C-NMR spectrum of compound **40** in DMSO-d_6.

Fig. S52. IR spectrum of compound **41** (KBr pellet).

Fig. S53. ^{1}H-NMR spectrum of compound **41** in DMSO-d_6.

Fig. S54. ^{13}C-NMR spectrum of compound **41** in DMSO-d_6.

Fig. S55. IR spectrum of compound **42** (KBr pellet).

Fig. S56. ^{1}H-NMR spectrum of compound **42** in DMSO-d_6.

Fig. S57. ^{13}C-NMR spectrum of compound **42** in DMSO-d_6.

Fig. S58. IR spectrum of compound **43** (KBr pellet).

Fig. S59. ^{1}H-NMR spectrum of compound **43** in DMSO-d_6.

Fig. S60. ^{13}C-NMR spectrum of compound **43** in DMSO-d_6.

Fig. S61. IR spectrum of compound **44** (KBr pellet).

Fig. S62. ^{1}H-NMR spectrum of compound **44** in DMSO-d_6.

Fig. S63. ^{13}C-NMR spectrum of compound **44** in DMSO-d_6.

Fig. S64. IR spectrum of compound **45** (KBr pellet).

Fig. S65. ^{1}H-NMR spectrum of compound **45** in DMSO-d_6.

Fig. S66. ^{13}C-NMR spectrum of compound **45** in DMSO-d_6.

Fig. S67. IR spectrum of compound **46** (KBr pellet).

Fig. S68. ^{1}H-NMR spectrum of compound **46** in DMSO-d_6.

Fig. S69. ^{13}C-NMR spectrum of compound **46** in DMSO-d_6.

Fig. S70. IR spectrum of compound **47** (KBr pellet).

Fig. S71. ^{1}H-NMR spectrum of compound **47** in DMSO-d_6.

Fig. S72. ^{13}C-NMR spectrum of compound **47** in DMSO-d_6.

Fig. S73. IR spectrum of compound **48** (KBr pellet).

Fig. S74. ^{1}H-NMR spectrum of compound **48** in DMSO-d_6.

Fig. S75. ^{13}C-NMR spectrum of compound **48** in DMSO-d_6.

Fig. S76. IR spectrum of compound **49** (KBr pellet).

Fig. S77. ^{1}H-NMR spectrum of compound **49** in DMSO-d_6.

Fig. S78. ^{13}C-NMR spectrum of compound **49** in DMSO-d_6.

Fig. S79. IR spectrum of compound **50** (KBr pellet).

Fig. S80. ^{1}H-NMR spectrum of compound **50** in DMSO-d_6.

Fig. S81. ^{13}C-NMR spectrum of compound **50** in DMSO-d_6.

Fig. S82. IR spectrum of compound **51** (KBr pellet).

Fig. S83. ^{1}H-NMR spectrum of compound **51** in DMSO-d_6.

Fig. S84. ^{13}C-NMR spectrum of compound **51** in DMSO-d_6.

Fig. S85. ORTEP view of compound **25** showing the atom-numbering scheme. Displacement ellipsoids are drawn at the 50 % probability level and H atoms are shown as small spheres of arbitrary radii.

Fig. S86. ORTEP view of compound **34** showing the atom-numbering scheme. Displacement ellipsoids are drawn at the 50 % probability level and H atoms are shown as small spheres of arbitrary radii.

Fig. S87. The crystal packing of compound **25**. The H atoms not engaged in the intermolecular interactions (dashed lines) have been skipped for clarity.

Fig. S88. The crystal packing of compound **34**. The H atoms not engaged in the intermolecular interactions (dashed lines) have been skipped for clarity.

Fig. S89. A projection of the optimized structure of compound **25** by DFT/B3LYP method with 3-21G* basis set.

Fig. S90. A projection of the optimized structure of compound **34** by DFT/B3LYP method with 3-21G* basis set.

Fig. S91. Overlay diagram of compound **25**; red (X-ray structure), green (DFT).

Fig. S92. Overlay diagram of compound **34**; red (X-ray structure), green (DFT).

Fig. S93. Dose-response curve for the tested compounds against HCT116 (colon cancer) cell line.

Fig. S94. Dose-response curve for the tested compounds against MCF7 (breast cancer) cell line.

Fig. S95. Dose-response curve for the tested compounds against A431 (squemous cancer) cell line.

Fig. S96. Dose-response curve for the tested compounds against RPE1 (retinal pigment epithelium) cell line.

Fig. S97. BMLR-QSAR model plot of correlations representing the observed *vs.* predicted $1/IC_{50}$, μM values for the tested compounds against HCT116 (colon) carcinoma cell line.

Fig. S98. BMLR-QSAR model plot of correlations representing the observed *vs.* predicted IC_{50}, μM values for the tested compounds against MCF7 (breast) carcinoma cell line.

Fig. S99. BMLR-QSAR model plot of correlations representing the observed *vs.* predicted log(IC_{50}), μM values for the tested compounds against A431 (squamous) carcinoma cell line.

Fig. S100. Constraint distances "H-1 – H-2 = 10.672, H-1 – H-3 = 7.810, H-2 – H-3 = 11.639, H-1 – HBA = 4.686, H-2 – HBA = 6.093, H-3 – HBA = 8.206 Å" of the generated 3D-pharmacophore for the tested piperidinecarboxamides **24–47** against HCT116 (colon) carcinoma cell line which contains three hydrophobics (H-1, H-2, H-3; light blue) and one hydrogen bonding acceptor (HBA; green).

Fig. S101. Constraint angles "H-1 – H-2 – H-3 = 40.69, H-3 – H-1 – HBA = 77.68, H-3 – H-2 – HBA = 42.09 °" of the generated 3D-pharmacophore for the tested piperidinecarboxamides **24–47** against HCT116 (colon) carcinoma cell line which contains three hydrophobics (H-1, H-2, H-3; light blue) and one hydrogen bonding acceptor (HBA; green).

Fig. S102. 3D-pharmacophore model mapped on the tested piperidinecarboxamides **24–47** against HCT116 (colon) carcinoma cell line.

Fig. S103. Constraint distances "H-1 – H-2 = 9.356, H-1 – HBA = 8.527, H-2 – HBA = 6.917 Å" of the generated 3D-pharmacophore for the tested piperidinecarboxamides **24–47** against MCF7 (breast) carcinoma cell line which contains two hydrophobics (H-1, H-2; light blue) and one hydrogen bonding acceptor (HBA; green).

Fig. S104. Constraint angle "H-2 – H-1 – HBA = 45.21 °" of the generated 3D-pharmacophore for the tested piperidinecarboxamides **24–47** against MCF7 (breast) carcinoma cell line which contains two hydrophobics (H-1, H-2; light blue) and one hydrogen bonding acceptor (HBA; green).

Fig. S105. 3D-pharmacophore model mapped on the tested piperidinecarboxamides **24–47** against MCF7 (breast) carcinoma cell line.

Fig. S106. Constraint distances "H-1 – H-2 = 8.270, H-1 – HBA-1 = 6.866, H-1 – HBA-2 = 4.726, H-2 – HBA-1 = 8.788, H-2 – HBA-2 = 4.683, HBA-1 – HBA-2 = 6.077 Å" of the generated 3D-pharmacophore for the tested piperidinecarboxamides **24–47** against A431 (squamous) carcinoma cell line which contains two hydrophobics (H-1, H-2; light blue) and two hydrogen bonding acceptor (HBA-1, HBA-2; green).

Fig. S107. Constraint angles "H-2 – H-1 – HBA-1 = 70.28, H-2 – HBA-2 – HBA-1 = 108.82 °" of the generated 3D-pharmacophore for the tested piperidinecarboxamides **24–47** against A431 (squamous) carcinoma cell line which contains two hydrophobics (H-1, H-2; light blue) and two hydrogen bonding acceptor (HBA-1, HBA-2; green).

Fig. S108. 3D-pharmacophore model mapped on the tested piperidinecarboxamides **24–47** against A431 (squamous) carcinoma cell line.

X-ray crystallography

Single crystals of compounds **25** and **34** were grown using slow solvent evaporation method. For compound **25**, good crystal has been selected, checked and mounted onto a thin glass fiber. The X-ray single crystal diffraction data were collected at room temperature (293 K) on an Enraf-Nonius 590 diffractometer with a Kappa CCD detector using graphite monochromated Mo-$K\alpha$ (λ = 0.71073 Å) radiation, at National Research Center of Egypt.[1,2] Reflection data has been recorded in the rotation mode using the ϕ and ω scan technique with $2\theta_{max}$ = 26.678 °. Unit cell parameters were determined from least-squares refinement with θ in the range $2 \leq \theta \leq 26$. Regarding compound **34**, Data collections were performed at the X-ray diffraction beamline (XRD1) of the Elettra Synchrotron, Trieste, Italy.[3] Suitable crystal was dipped in NHV oil (Jena Bioscience GmbH) and mounted on the goniometer head with a nylon loop. Complete datasets were collected at room temperature through the rotating crystal method with $2\theta_{max}$ = 30.961 °. Data were acquired using a monochromatic wavelength of 0.700 Å on a Pilatus 2M hybrid-pixel area detector. The diffraction data were indexed with θ in the range $2 \leq \theta \leq 30$ and integrated using XDS.[4] The structure was solved by direct methods using *SIR-92*[5] and *SUPERFLIP*[6] implemented in *CRYSTALS* program suit.[7] The refinement was carried out by full-matrix least-squares method on the positional and anisotropic temperature parameters of all non-hydrogen atoms based on F^2 using *CRYSTALS* package. All hydrogen atoms were positioned geometrically and were initially refined with soft restraints on the bond lengths and angles to regularize their geometry (C—H in the range 0.93–0.98 and N—H in the range 0.86–0.89) and U_{iso}(H) (in the range 1.2-1.5 times U_{eq} of the parent atom). Then, the positions were refined with riding constraints.[8] The general-purpose crystallographic tool *PLATON*[9] was used for the structure analysis and presentation of the results. The molecular graphics were carried out using *ORTEP-3* for Windows[10] program. Details of the data collection conditions and the parameters of the refinement process are given in Table S11. Crystallographic data for the structures in this paper have been deposited with the Cambridge Crystallographic Data Center as supplementary publication numbers CCDC 1855508 and CCDC 1855509. Copies of the data can be obtained, free of charge, on application to CCDC, 12 Union Road, Cambridge CB2 1EZ, UK [Fax: 144(0)1223 336033 or e-mail: deposit@ccdc.cam.ac.uk].

In-vitro antitumor screening

The targeted piperidinecarboxamides **24–51** were screened for their antitumor properties against HCT116 (colon), MCF7 (breast) and A431 (squamous skin) carcinoma cell lines by the standard mitochondrial dependent reduction of yellow MTT [3-(4,5-dimethylthiazol-2-yl)-2,5-diphenyl-tetrazolium bromide] to purple formazan technique.[11] Cells were suspended in McCoy's 5A medium for HCT116, DMEM for MCF-7 and A431 in addition to 1% antibiotic–antimycotic mixture (10000 μg ml^{-1} potassium penicillin, 10000 μg ml^{-1} streptomycin sulfate and 25 μg ml^{-1} amphotericin B), 10% fetal bovine serum and 1% L-glutamine at 37 °C, under 5% CO_2 and 95% humidity. Cells were seeded at concentration of 30000 cells per well in fresh complete growth medium in 96-well tissue culture microtiter plates for 24 h. Media was aspirated, fresh complete medium was added and cells were incubated with different concentrations of the tested compound to give a final concentration of [50, 25, 12.5 and 6.25 μM (3, 1.5, 7.5 and 0.375 μM for the high potent analogues)]. 0.5% DMSO was used as negative control and 5-fluorouracil was used as positive control (standard reference). Triplicate wells were prepared for each individual dose. After 72 h of incubation, medium was aspirated, 40 μl MTT salt (2.5 mg ml^{-1}) were added to each well and incubated for further 4 h at 37 °C. To stop the reaction and dissolve the formed crystals, 150 μl of 10% sodium dodecyl sulfate (SDS) in deionized water were added to each well and incubated overnight at 37 °C. The absorbance was then measured at 570 nm and a reference wavelength of 595 nm.

Data were collected as mean values for experiments performed in triplicates for each individual dose which had been measured by MTT assay. Control experiments did not exhibit significant change compared to the DMSO vehicle. The percentage of cell survival was calculated according to equ. (1).

$$Surviving\ fraction = \frac{Optical\ density\ (O.D.) of\ treated\ cells}{O.D. of\ control\ cells} \quad \ldots\ldots\ldots\ldots\ldots (1)$$

The synthesized compounds **24–51** were also tested against RPE1 (normal human immortalized retinal pigment epithelial cell line) cell to determine the toxicity/selectivity

towards normal cells relative to the carcinoma cell lines utilized. The IC_{50} (concentration required to produce 50% inhibition of cell growth compared to the control experiment) was determined using Graph-Pad PRISM version-5 software. Statistical calculations for determination of the mean and standard error values were determined by SPSS 16 software. The observed anti-proliferative properties are presented in Table 1 (Supplementary Figs. S93–S96).

2D-QSAR studies

The synthesized 3,5-bis(arylidene)-*N*-substituted-4-oxo-piperidine-1-carboxamides **24-47** revealing variable anti-proliferative properties were utilized for developing the 2D-QSAR modeling by CODESSA-Pro (comprehensive descriptors for structural and statistical analysis) software. Geometry of the compounds was initially optimized by AM1 technique using hyperChem 8.0 then, uploaded to CODESSA-Pro for final geometrical structure optimization by MOPAC.[12] CODESSA-Pro calculated 673 (for the tested compounds against HCT116 and MCF7 cell lines) and 711 (for the tested compounds against A431 cell line) molecular descriptors (constitutional, topological, geometrical, charge-related, semi-empirical, molecular-type, atomic-type and bond-type descriptors in addition to, thermodynamic descriptors in case of A431 cell line) for the exported anti-proliferative active agents. Mathematical transformation of the experimental values (including IC_{50}, $1/IC_{50}$, $\log(IC_{50})$ and $1/\log(IC_{50})$ μM) were used searching for the best QSAR model. The best multi-linear regression (BMLR) technique was utilized which is a stepwise search for the best n-parameter regression equations (where n stands for the number of descriptors used), based on the highest R^2 (squared correlation coefficient), R^2cvOO (squared cross-validation "leave one-out, LOO" coefficient), R^2cvMO (squared cross-validation "leave many-out up to 20% of the training set, LMO" coefficient), F (Fisher statistical significance criteria) values, and s^2 (standard deviation). The QSAR up to 3-descriptor model describing the biological activity of the anti-proliferative active agents were generated (obeying the thumb rule of 8:1 which is the ratio between the data points and the number of QSAR descriptor).

Human DNA topoisomerase IIα inhibitory properties

Human DNA topoisomerase IIα activity assay was undertaken for compounds **29**, **30** and **34–38** by the Confirmatory Diagnostic Unit, Egyptian Company for Production of Vaccines, Sera and Drugs (VACSERA), Cairo, Egypt according to the Topoisomerase II Assay Kit, (plasmid based) manufacturer's instructions (TopoGEN, Inc., 108 Aces Alley, Port Orange, Florida 32128, USA) using ROBONIK EIA reader (450 nm), DMSO as solvent and Methotrexate as a standard reference.

References

1. X-ray Crystallography Laboratory, National Research Centre of Egypt. Available from: http://www.xrdlab-nrc-eg.org
2. R. W. W. Hooft, Collect: Data collection software, 1998.
3. A. Lausi1, M. Polentarutti, S. Onesti, J. R. Plaisier, E. Busetto, G. Bais, L. Barba, A. Cassetta, G. Campi, D. Lamba, A. Pifferi, S. C. Mande, D. D. Sarma, S. M. Sharma and G. Paolucci, *Eur. Phys. J. Plus*, 2015, **130**, 43.
4. W. Kabsch, *Acta Crystallogr.*, 2010, **D66**, 125–132.
5. A. Altomare, G. Cascarano, C. Giacovazzo and A. Guagliardi, *J. Appl. Crystallogr.*, 1993, **26**, 343–350.
6. L. Palatinus, G. Chapuis, *J. Appl. Crystallogr.*, 2007, **40**, 786–790.
7. P. W. Betteridge, J. R. Carruthers, R. I. Cooper, K. Prout and D. J. Watkin, *J. Appl. Crystallogr.*, 2003, **36**, 1487.
8. R. I. Cooper, A. L. Thompson and D. J. Watkin, *J. Appl. Crystallogr.*, 2010, **43**, 1100–1107.
9. A. L. Spek, *J. Appl. Crystallogr.*, 2003, **36**, 7–13.
10. L. J. Farrugia, *J. Appl. Crystallogr.*, 1997, **30**, 565.
11. N. S. M. Ismail, R. F. George, R. A. T. Serya, F. N. Baselious, M. El-Manawaty, E. M. Shalaby and A. S. Girgis, *RSC Adv.*, 2016, **6**, 101911–101923.
12. E. A. Soliman, S. S. Panda, M. N. Aziz, E. M. Shalaby, N. Mishriky, F. M. Asaad and A. S. Girgis, *Eur. J. Med. Chem.*, 2017, **138**, 920–931.

Table S1. Hydrogen-bond geometry (Å, °) for compound **25**.

D—H···*A*	*D*—H	H···*A*	*D*···*A*	*D*—H···*A*
N1—H262···O2^{i}	0.86(2)	2.13(3)	2.950(3)	161(2)
C26—H261···O1^{ii}	0.95	2.57	3.172(5)	122

Symmetry codes: (i) 1/2-x,1/2-y,1-z; (ii) 1/2-x,1/2+y,1/2-z.

Table S2. Hydrogen-bond geometry (Å, °) for compound **34**.

D—H···*A*	*D*—H	H···*A*	*D*···*A*	*D*—H···*A*
N1—H2···O2^{i}	0.85(3)	2.07(2)	2.908(3)	165(2)
C23—H231···O1^{ii}	0.95	2.49	3.398(3)	161

Symmetry codes: (i) 1-x,1-y,-z; (ii) -1/2+x,3/2-y,-1/2+z.

Table S3. Selected intramolecular experimental (X-ray) and computational optimized geometrical parameters (bond lengths, Å) of compounds **25** and **34**.

Geometric parameters	Compound **25**		Compound **34**	
	Exp. X-ray data	DFT	Exp. X-ray data	DFT
O1—C7	1.229	1.245	1.215	1.245
O2—C18	1.234	1.248	1.237	1.248
N1—C6	1.41	1.41	1.410	1.414
N1—C7	1.371	1.391	1.368	1.387
N2—C7	1.361	1.397	1.373	1.401
N2—C8	1.45	1.469	1.457	1.468
N2—C9	1.451	1.470	1.450	1.470
C1—C2	1.381	1.391	1.391	1.392
C1—C6	1.388	1.406	1.382	1.406
C2—C3	1.377	1.393	1.354	1.397
C3—C4	1.373	1.392	1.360	1.396
C4—C5	1.385	1.393	1.383	1.394
C5—C6	1.389	1.405	1.384	1.404
C8—C19	1.512	1.514	1.507	1.514
C9—C17	1.5	1.526	1.505	1.526
C10—C11	1.383	1.393	1.373	1.390
C10—C15	1.386	1.411	1.398	1.412
C11—C12	1.368	1.397	1.338	1.390
C12—C13	1.374	1.398	1.347	1.390
C13—C14	1.381	1.391	1.408	1.388
C14—C15	1.393	1.413	1.385	1.413
C15—C16	1.472	1.461	1.457	1.459
C16—C17	1.341	1.353	1.339	1.353
C17—C18	1.496	1.503	1.485	1.502
C18—C19	1.49	1.503	1.484	1.502
C19—C20	1.342	1.347	1.353	1.347
C20—C21	1.459	1.471	1.454	1.469

C21—C22	1.403	1.410	1.409	1.411
C21—C26	1.38	1.409	1.391	1.409
C22—C23	1.376	1.394	1.376	1.390
C23—C24	1.378	1.399	1.366	1.391
C24—C25	1.366	1.397	1.377	1.389
C25—C26	1.387	1.395	1.388	1.392
C3—Cl1	1.746	1.766	---	---
C24—F1	---	---	1.360	1.367
C12—F2	---	---	1.356	1.367
RMSE	---	0.018	---	0.022
Maximum difference	---	0.036	---	0.052

Table S4. Selected intramolecular experimental (X-ray) and computational optimized geometrical parameters (bond angles, °) of compounds **25** and **34**.

Geometric parameters	Compound **25**		Compound **34**	
	Exp. X-ray data	DFT	Exp. X-ray data	DFT
C6—N1—C7	125.8	126.3	124.7	126.5
C7—N2—C8	128.6	124.7	124.8	124.8
C7—N2—C9	119.4	117.7	117.7	117.4
C8—N2—C9	111.9	113.1	113.1	113.2
C2—C1—C6	121.1	120.9	119.4	120.6
C1—C2—C3	119.6	119.4	120.8	120.3
C2—C3—C4	119.9	120.5	119.3	119.1
C3—C4—C5	120.9	120.2	122.1	121.2
C4—C5—C6	119.7	120.2	118.3	119.7
N1—C6—C5	123.2	123.4	122.4	123.4
N1—C6—C1	118	117.8	117.5	117.5
C5—C6—C1	118.8	118.8	120.1	119.0
N1—C7—N2	116.8	114.4	114.5	114.4
N2—C7—O1	120.7	121.3	121.4	121.0
N1—C7—O1	122.5	124.3	123.9	124.6
N2—C8—C19	111.3	107.6	110.2	107.8
N2—C9—C17	108.8	110.5	109.0	110.5
C11—C10—C15	121.2	120.7	121.2	120.9
C10—C11—C12	120	120.5	119.3	119.8
C11—C12—C13	120.6	119.7	123.1	120.8
C12—C13—C14	119	120.0	118.2	119.3
C13—C14—C15	122	121.2	120.7	121.5
C14—C15—C10	117.2	117.9	117.3	117.6
C14—C15—C16	118.3	117.2	118.7	117.3
C10—C15—C16	124.4	124.9	124.0	125.1
C15—C16—C17	128.2	131.2	129.0	131.4
C9—C17—C16	124.9	125.2	124.5	125.3

C9—C17—C18	116.5	119.5	117.3	119.4
C16—C17—C18	118.6	115.2	118.1	115.2
C17—C18—O2	120.3	121.6	120.4	121.5
C17—C18—C19	118.1	118.3	118.4	118.4
O2—C18—C19	121.5	120.1	121.2	120.1
C8—C19—C18	117.7	116.0	118.4	116.2
C8—C19—C20	123.5	126.1	123.8	126.1
C18—C19—C20	118.7	117.6	117.8	117.4
C19—C20—C21	130.8	127.1	131.4	127.2
C20—C21—C22	118.8	118.8	117.2	119.0
C20—C21—C26	124.3	122.7	125.3	122.8
C22—C21—C26	116.9	118.5	117.5	118.1
C21—C22—C23	121.2	120.7	122.0	121.0
C22—C23—C24	120.3	120.1	117.9	119.5
C23—C24—C25	119.8	119.8	123.0	120.9
C24—C25—C26	119.8	120.3	118.3	119.6
C25—C26—C21	122.1	120.6	121.3	120.9
C2—C3—Cl1	120.2	119.6	---	---
C4—C3—Cl1	119.9	119.9	---	---
C23—C24—F1	---	---	119.4	119.5
C25—C24—F1	---	---	117.6	119.6
C11—C12—F2	---	---	119.3	119.6
C13—C12—F2	---	---	117.6	119.6
RMSE	---	1.602	---	1.493
Maximum difference	---	3.9	---	4.2

Table S5. Descriptors of the BMLR-QSAR model for the synthesized piperidinecarboxamides **24–47** against HCT116 (colon) carcinoma cell line.

Entry	ID	Coefficient	*s*	*t*	Descriptor
1	0	–0.833447	0.542	–1.536	Intercept
2	D_1	0.102299	0.007	15.070	Tot. molecular 1-center E-E repulsion / # of atoms
3	D_2	–88.6353	19.707	–4.497	Min. 1-electron react. index for atom O
4	D_3	–2.287	0.419	–5.456	Average information content (order 0)

$N = 24$, $n = 3$, $R^2 = 0.934$, R^2cvOO = 0.905, R^2cvMO = 0.909, $F = 93.751$, $s^2 = 0.019$

$1/IC_{50}$ (μM) = –0.833447 + (0.102299 x D_1) – (88.6353 x D_2) – (2.287 x D_3)

Table S6. Descriptors of the BMLR-QSAR model for the synthesized piperidinecarboxamides **24–47** against MCF7 (breast) carcinoma cell line.

Entry	ID	Coefficient	*s*	*t*	Descriptor
1	0	–1.26351	0.520	–2.431	Intercept
2	D_1	4.27333	0.260	16.432	FPSA-2 Fractional PPSA (PPSA-2/TMSA) (MOPAC PC)
3	D_2	–0.0318232	0.006	–5.289	HA dependent HDSA-1 (Zefirov PC)
4	D_3	–866.471	104.142	–8.320	Partial charged surface area (MOPAC PC) for atom C

$N = 24$, $n = 3$, $R^2 = 0.951$, R^2cvOO = 0.931, R^2cvMO = 0.933, $F = 130.382$, $s^2 = 0.125$

IC_{50} (μM) = –1.26351 + (4.27333 x D_1) – (0.0318232 x D_2) – (866.471 x D_3)

Table S7. Descriptors of the BMLR-QSAR model for the synthesized piperidinecarboxamides **24–47** against A431 (squamous) carcinoma cell line.

Entry	ID	Coefficient	*s*	*t*	Descriptor
1	0	166.573	19.779	8.422	Intercept
2	D_1	0.068221	0.006	10.621	Count of H-donors sites (MOPAC PC)
3	D_2	–5.34709	1.134	–4.713	Min. resonance energy for bond H-C
4	D_3	–0.343666	0.056	–6.187	Min. e-n attraction for atom N

N = 24, n = 3, R^2 = 0.901, R^2cvOO = 0.859, R^2cvMO = 0.861, F = 60.784, s^2 = 0.012

log(IC$_{50}$, μM) = 166.573 + (0.068221 x D_1) – (5.34709 x D_2) – (0.343666 x D_3)

Table S8. Molecular descriptor values of the BMLR-QSAR model for the synthesized piperidinecarboxamides **24–47** against HCT116 (colon) carcinoma cell line.

Entry	Compd.	Descriptors[a]		
		D_1	D_2	D_3
1	**24**	51.78765	-0.00011	1.58224
2	**25**	56.32968	0.00004	1.6951
3	**26**	53.2964	-0.00043	1.72105
4	**27**	50.09242	-0.00489	1.72733
5	**28**	50.50078	-0.00001	1.62076
6	**29**	60.69776	-0.00073	1.76818
7	**30**	65.15003	0	1.82535
8	**31**	61.53756	-0.00023	1.8984
9	**32**	59.69894	-0.00263	1.92877
10	**33**	58.90671	0.00002	1.80133
11	**34**	64.11513	-0.00015	1.76818
12	**35**	68.54209	-0.00024	1.87833
13	**36**	64.7134	-0.00005	1.8984
14	**37**	63.41592	-0.00398	1.92877
15	**38**	62.16622	-0.00418	1.80133
16	**39**	49.26383	-0.00335	1.64676
17	**40**	50.76083	-0.00007	1.75654
18	**41**	47.42904	-0.00261	1.74045
19	**42**	48.2028	0	1.66427
20	**43**	54.56279	-0.00167	1.80272
21	**44**	58.44992	-0.00308	1.90463
22	**45**	55.67702	-0.00037	1.85923
23	**46**	53.28805	-0.00354	1.90057
24	**47**	53.25566	-0.00133	1.81451

[a] D_1 = Tot. molecular 1-center E-E repulsion / # of atoms, D_2 = Min. 1-electron react. index for atom O, D_3 = Average information content (order 0).

Table S9. Molecular descriptor values of the BMLR-QSAR model for the synthesized piperidinecarboxamides **24–47** against MCF7 (breast) carcinoma cell line.

Entry	Compd.	Descriptors[a]		
		D_1	D_2	D_3
1	**24**	2.24689	40.07887	0.00655
2	**25**	2.12439	42.94164	0.00456
3	**26**	2.50963	81.11199	0.00505
4	**27**	2.28674	41.51026	0.00528
5	**28**	2.30862	22.90221	0.00619
6	**29**	1.81189	58.68691	0.0035
7	**30**	1.3958	33.39906	0.00317
8	**31**	1.8106	74.43218	0.00296
9	**32**	1.72692	62.02682	0.00234
10	**33**	1.84054	45.80442	0.00363
11	**34**	2.05609	35.30757	0.00499
12	**35**	1.71978	34.35332	0.00408
13	**36**	2.2252	83.02051	0.00395
14	**37**	2.0041	41.51026	0.004
15	**38**	2.23988	52.0071	0.00476
16	**39**	2.73898	61.07256	0.00466
17	**40**	2.86792	79.68061	0.00455
18	**41**	2.65675	60.1183	0.00382
19	**42**	2.80097	38.17035	0.00506
20	**43**	2.71423	93.51736	0.00344
21	**44**	2.4645	93.51736	0.00272
22	**45**	2.85685	91.13171	0.00256
23	**46**	2.70558	85.88329	0.00282
24	**47**	2.73771	80.15774	0.00363

[a] D_1 = FPSA-2 Fractional PPSA (PPSA-2/TMSA) (MOPAC PC), D_2 = HA dependent HDSA-1 (Zefirov PC), D_3 = Partial charged surface area (MOPAC PC) for atom C.

Table S10. Molecular descriptor values of the BMLR-QSAR model for the synthesized piperidinecarboxamides **24–47** against A431 (squamous) carcinoma cell line.

Entry	Compd.	Descriptors[a]		
		D_1	D_2	D_3
1	**24**	7	10.3613	324.0338
2	**25**	7	10.3565	323.9441
3	**26**	10	10.3648	324.111
4	**27**	12	10.3565	324.1108
5	**28**	9	10.3599	324.0577
6	**29**	7	10.4147	323.9467
7	**30**	7	10.3771	324.7383
8	**31**	10	10.3843	324.3601
9	**32**	12	10.4167	324.5874
10	**33**	9	10.3675	324.459
11	**34**	7	10.3789	324.594
12	**35**	7	10.3786	324.5205
13	**36**	10	10.4128	324.693
14	**37**	12	10.3689	325.709
15	**38**	9	10.3669	324.7718
16	**39**	13	10.3766	324.5288
17	**40**	16	10.3811	324.0791
18	**41**	18	10.4128	324.7377
19	**42**	15	10.3611	324.0454
20	**43**	13	10.4129	323.8358
21	**44**	13	10.3893	324.1668
22	**45**	16	10.3517	323.823
23	**46**	18	10.3888	324.4444
24	**47**	15	10.3635	324.0558

[a] D_1 = Count of H-donors sites (MOPAC PC), D_2 = Min. resonance energy for bond H-C, D_3 = Min. e-n attraction for atom N.

Table S11. Crystal data and structure refinement parameters for compounds **25** and **34**.

Crystal data	Compound **25**	Compound **34**
Chemical formula	$C_{26}H_{21}ClN_2O_2$	$C_{26}H_{20}F_2N_2O_2$
M_r	428.90	430.45
Crystal system, space group	Monoclinic, C2/c	Monoclinic, P21/n
Temperature (K)	293	293
a, *b*, *c* (Å)	26.930 (3), 12.9179 (18), 12.9500 (14)	14.374 (3), 8.0590 (16), 18.554 (4)
β (°)	107.491 (3)	96.61 (3)
V (Å^3)	4296.7 (5)	2135.0 (8)
Z	8	4
Radiation type	Mo Kα	Mo Kα
μ (mm^{-1})	0.20	0.10
Crystal size (mm)	0.28 × 0.18 × 0.14	0.05 × 0.07 × 0.07
**Data collection**		
No. of measured, independent and observed [I > 2.0σ(I)] reflections	11327, 2861, 1790	29314, 6693, 5048
R_{int}	0.036	0.056
(sin θ/λ)$_{max}$ (Å^{-1})	0.632	0.735
**Refinement**		
$R[F^2 > 2\sigma(F^2)]$, $wR(F^2)$, S	0.039, 0.077, 0.95	0.064, 0.177, 1.00
No. of reflections	1790	5048
No. of parameters	173	294
$\Delta\rho_{max}$, $\Delta\rho_{min}$ (e Å^{-3})	0.13, −0.16	0.49, −0.41

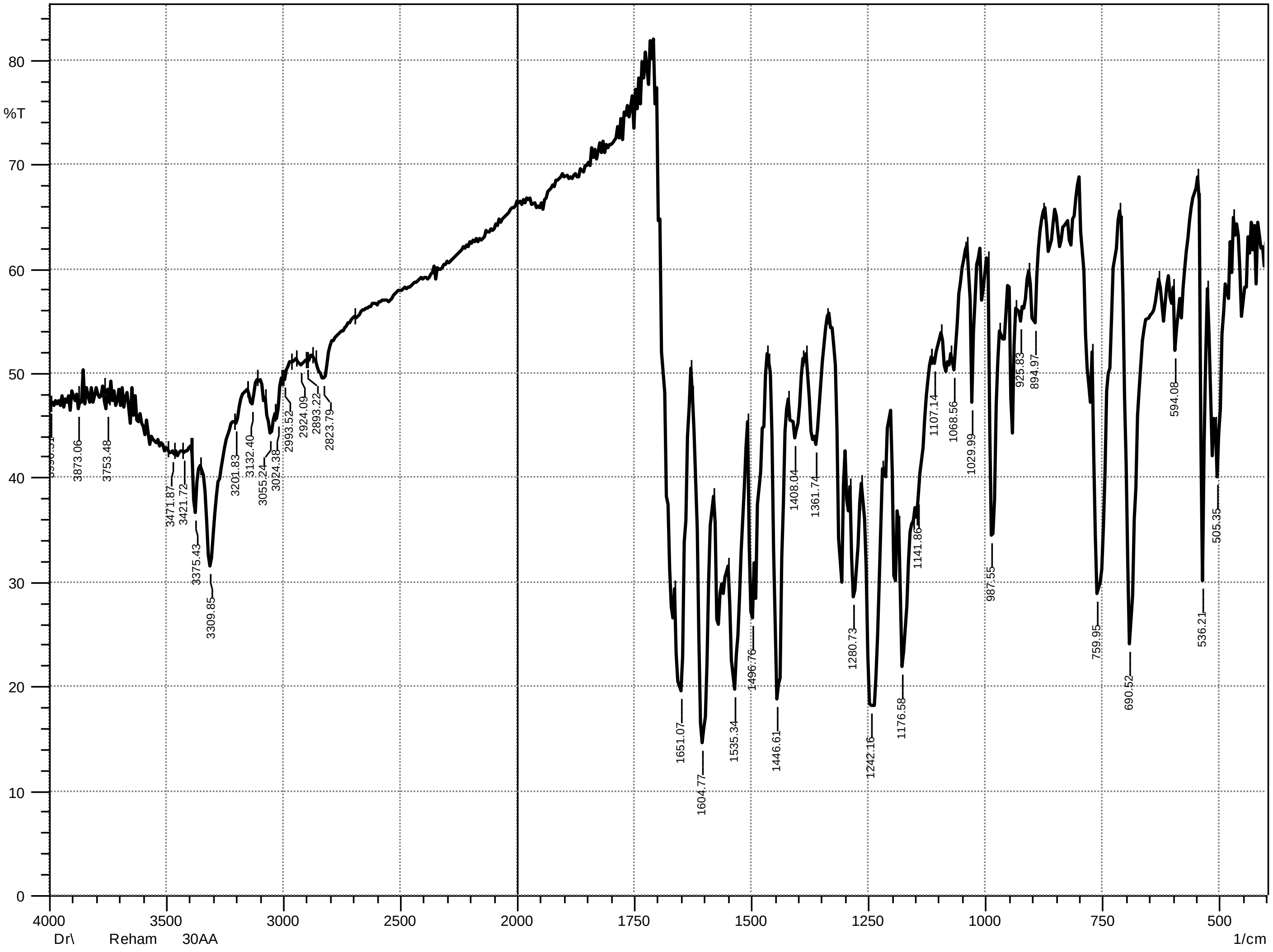

Fig. S1. IR spectrum of compound **24** (KBr pellet).

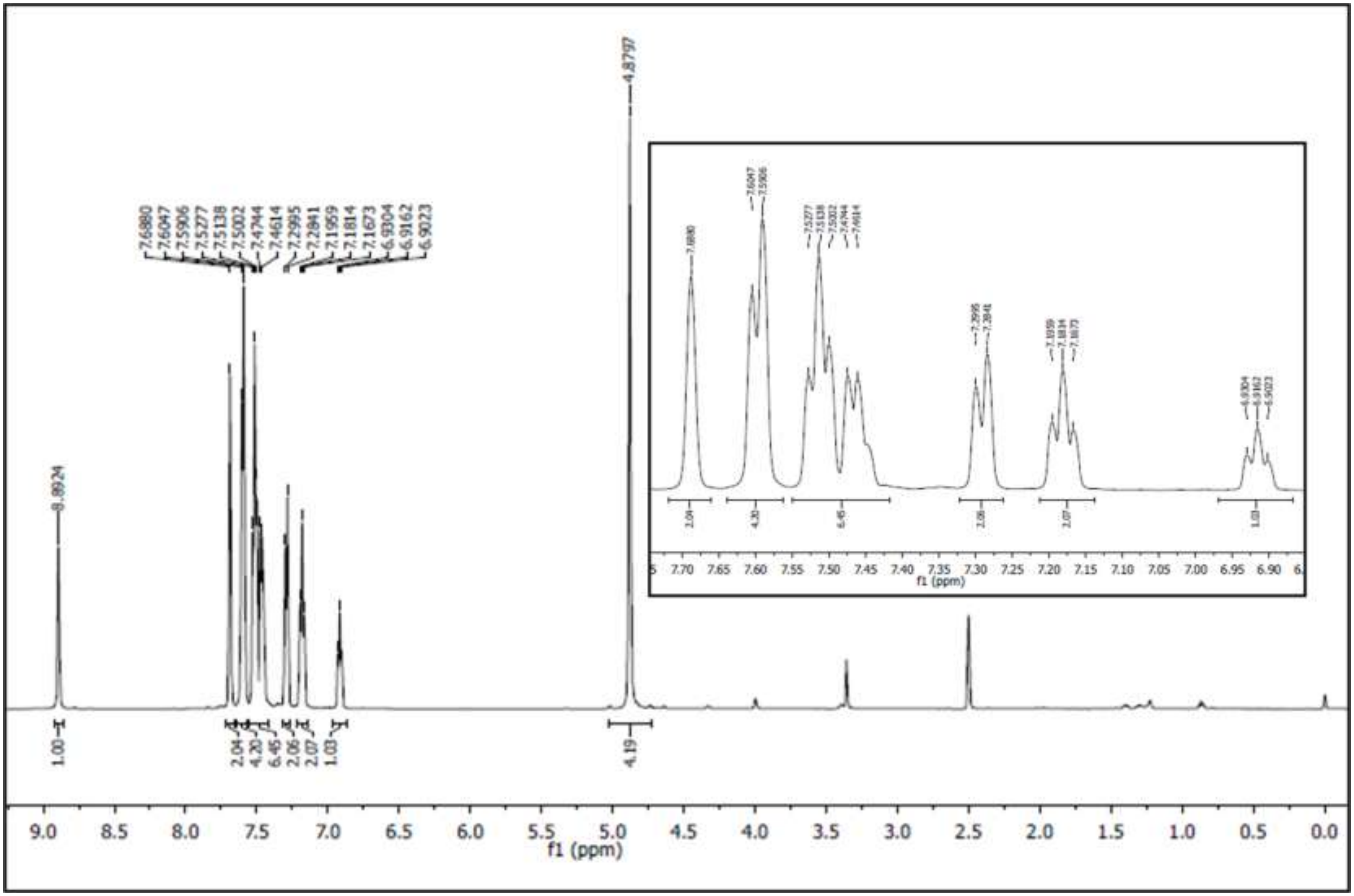

Fig. S2. ^{1}H-NMR spectrum of compound **24** in DMSO-d_6.

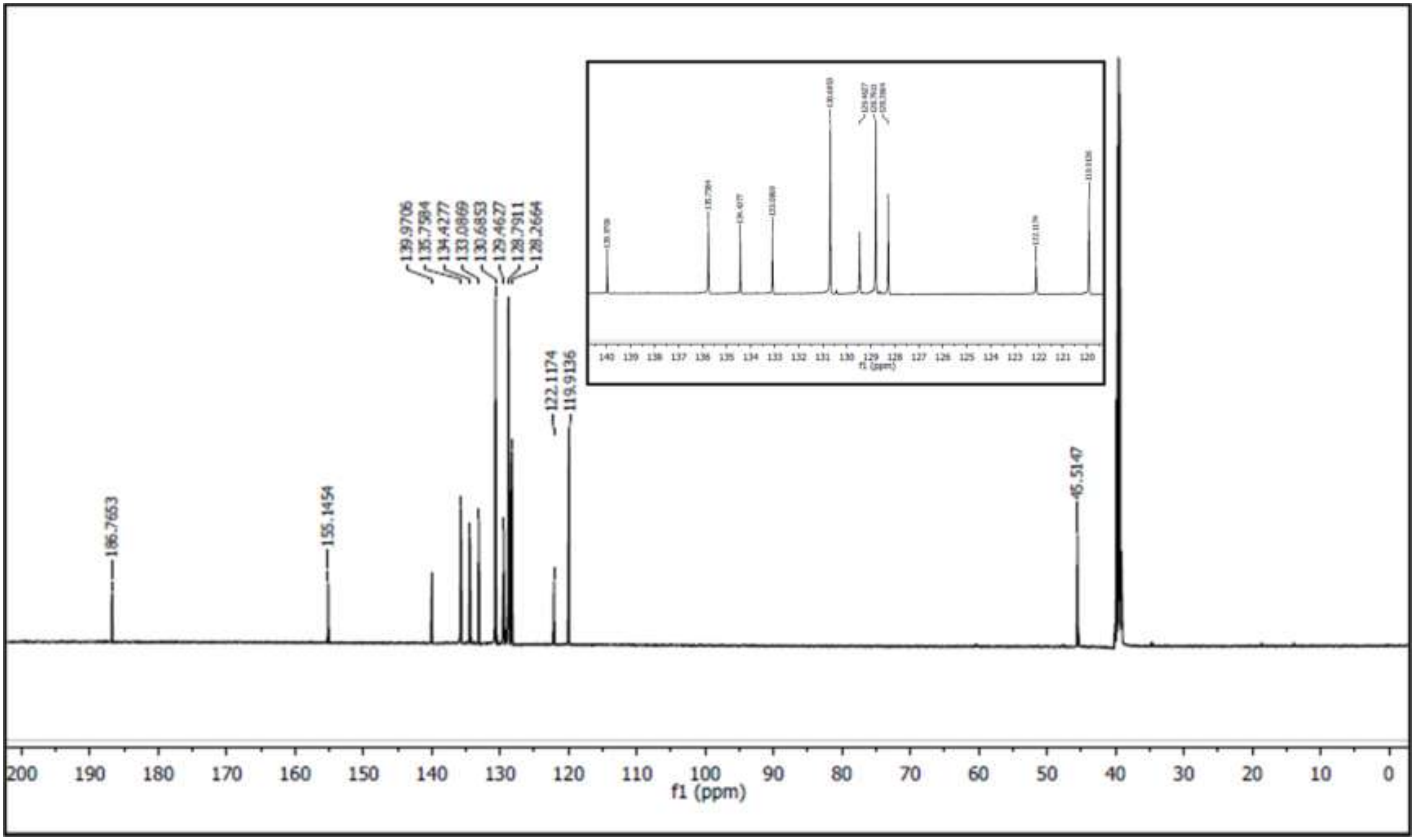

Fig. S3. ^{13}C-NMR spectrum of compound **24** in DMSO-d_6.

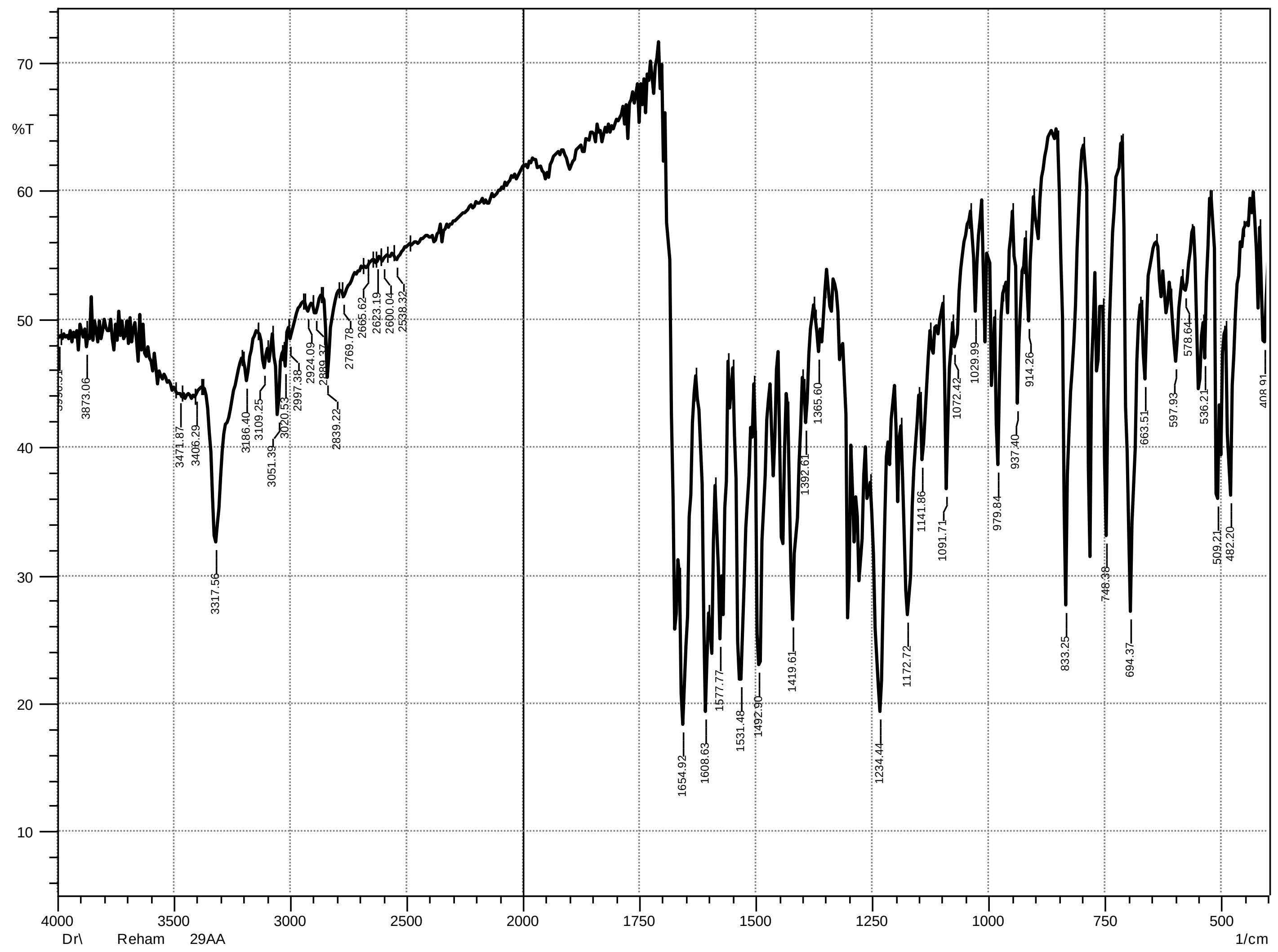

Fig. S4. IR spectrum of compound **25** (KBr pellet).

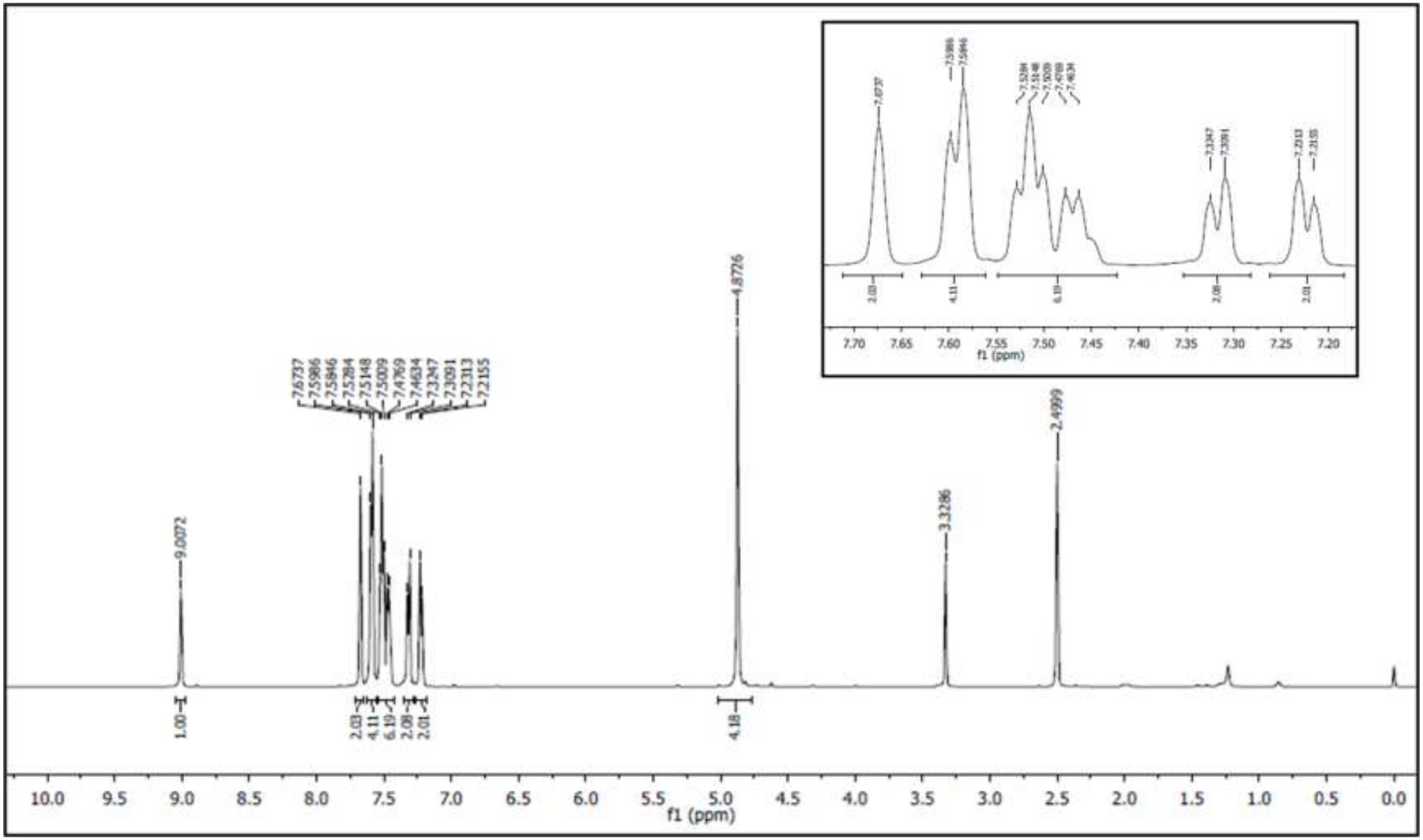

Fig. S5. ^{1}H-NMR spectrum of compound **25** in DMSO-d_6.

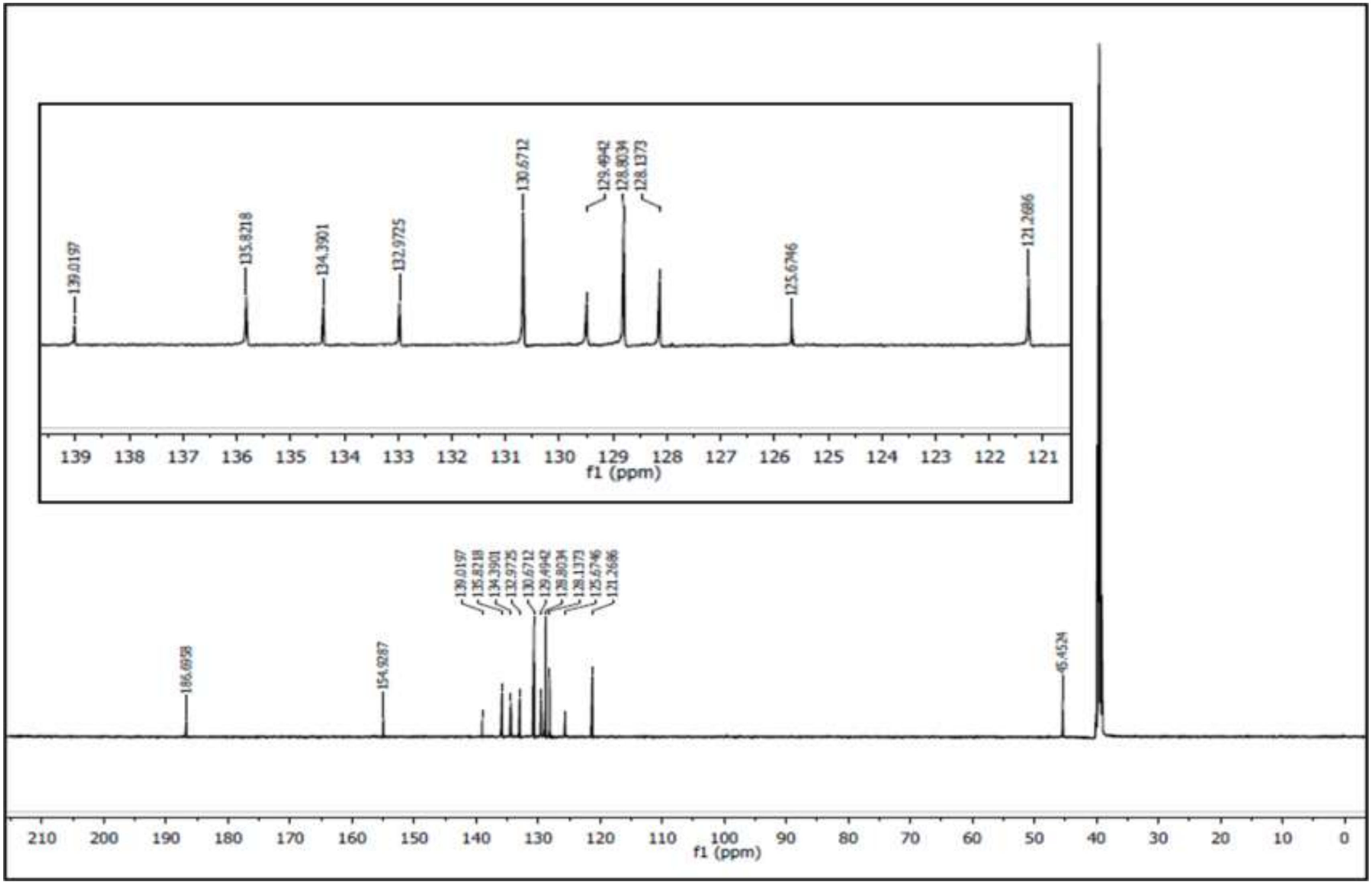

Fig. S6. ^{13}C-NMR spectrum of compound **25** in DMSO-d_6.

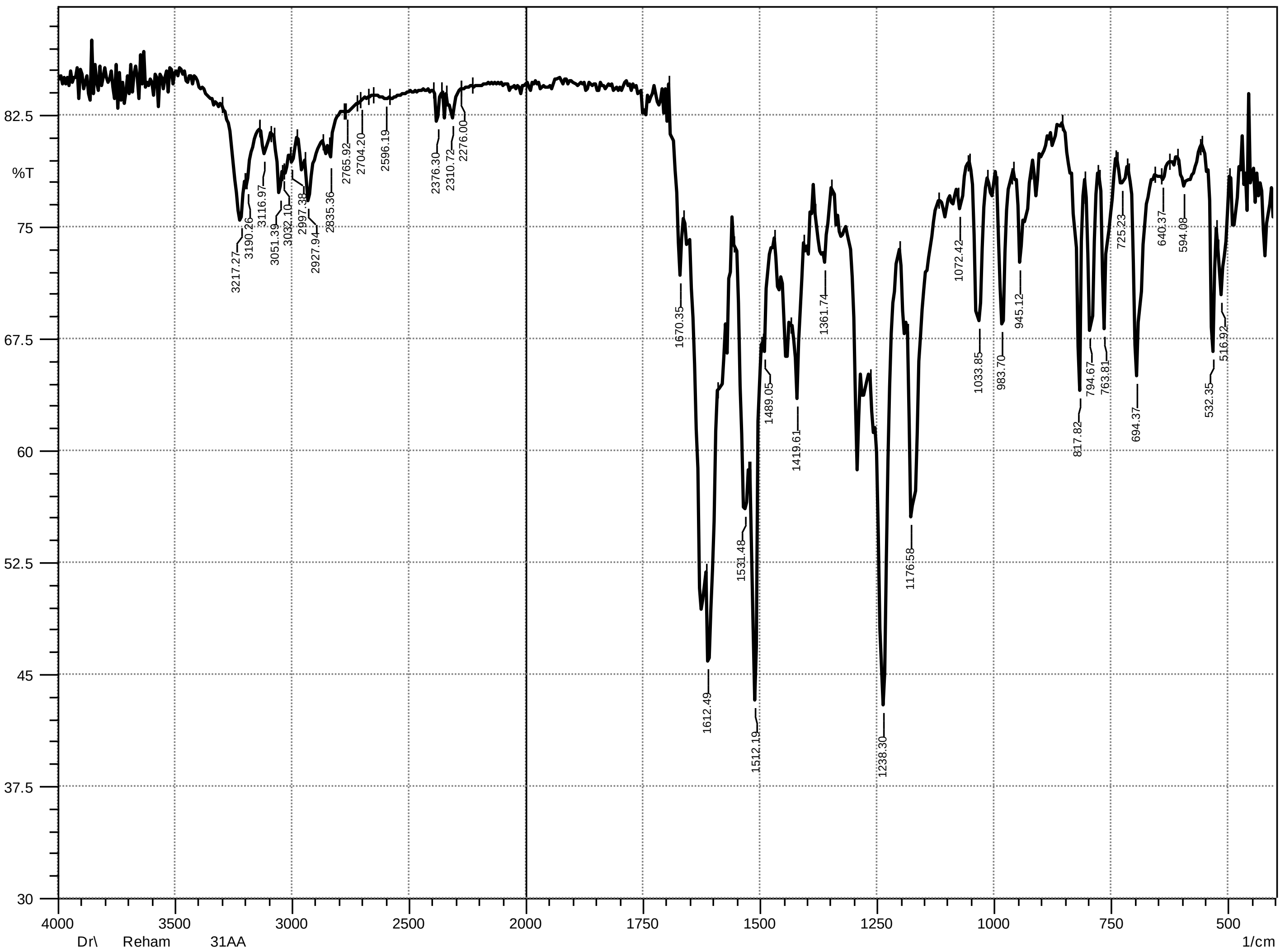

Fig. S7. IR spectrum of compound **26** (KBr pellet).

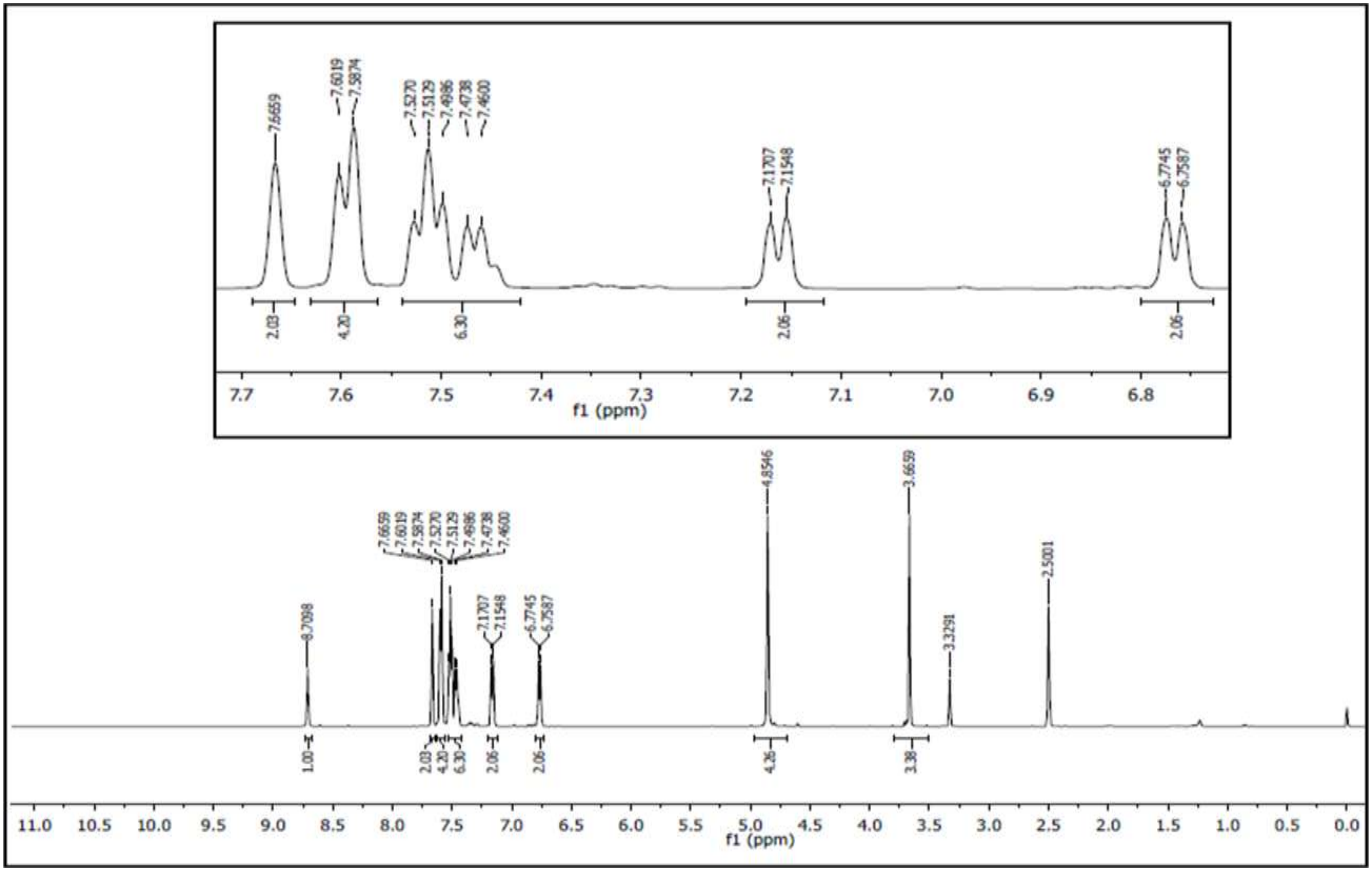

Fig. S8. ^{1}H-NMR spectrum of compound **26** in DMSO-d_6.

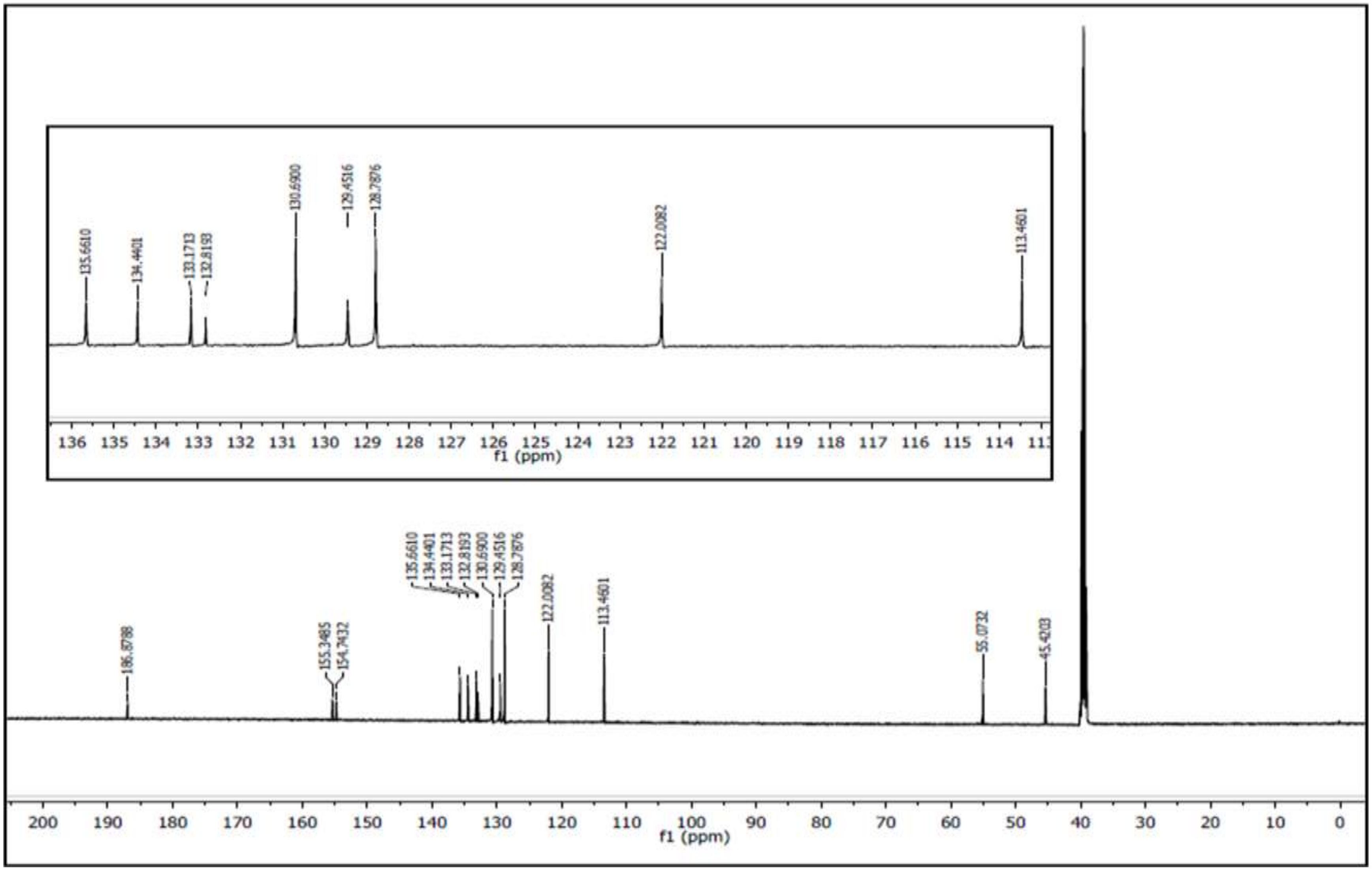

Fig. S9. ^{13}C-NMR spectrum of compound **26** in DMSO-d_6.

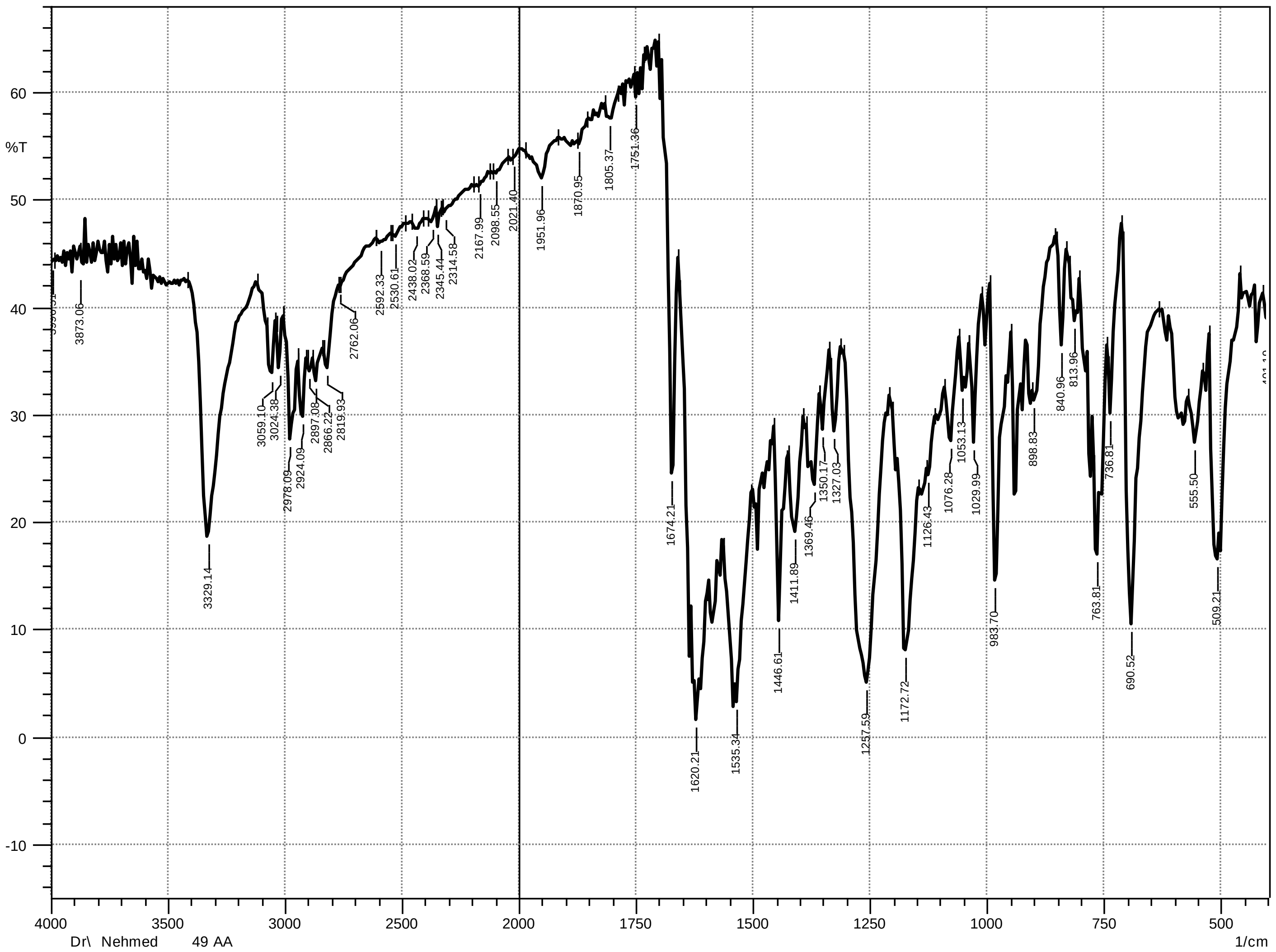

Fig. S10. IR spectrum of compound **27** (KBr pellet).

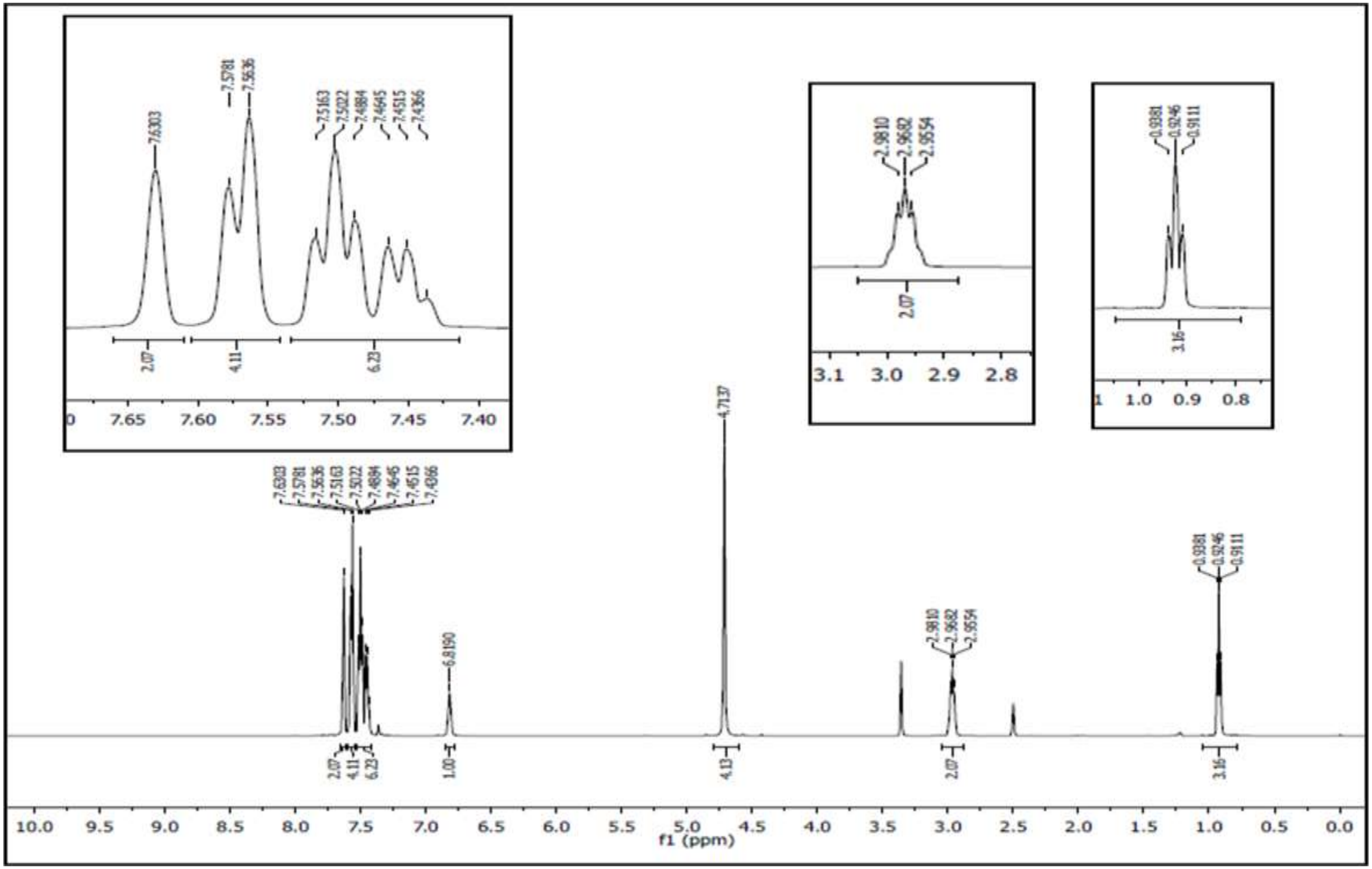

Fig. S11. ^{1}H-NMR spectrum of compound **27** in DMSO-d_6.

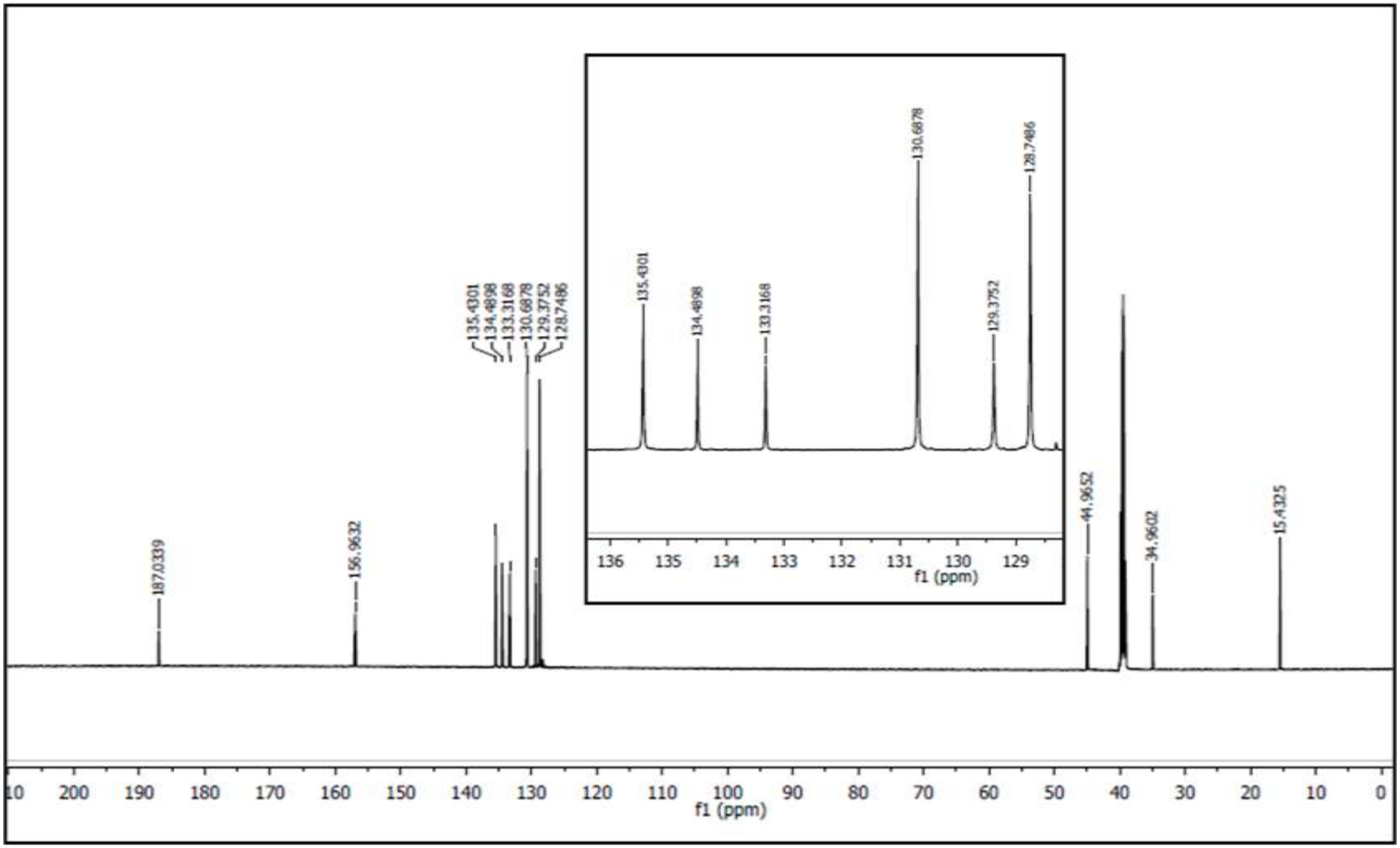

Fig. S12. ^{13}C-NMR spectrum of compound **27** in DMSO-d_6.

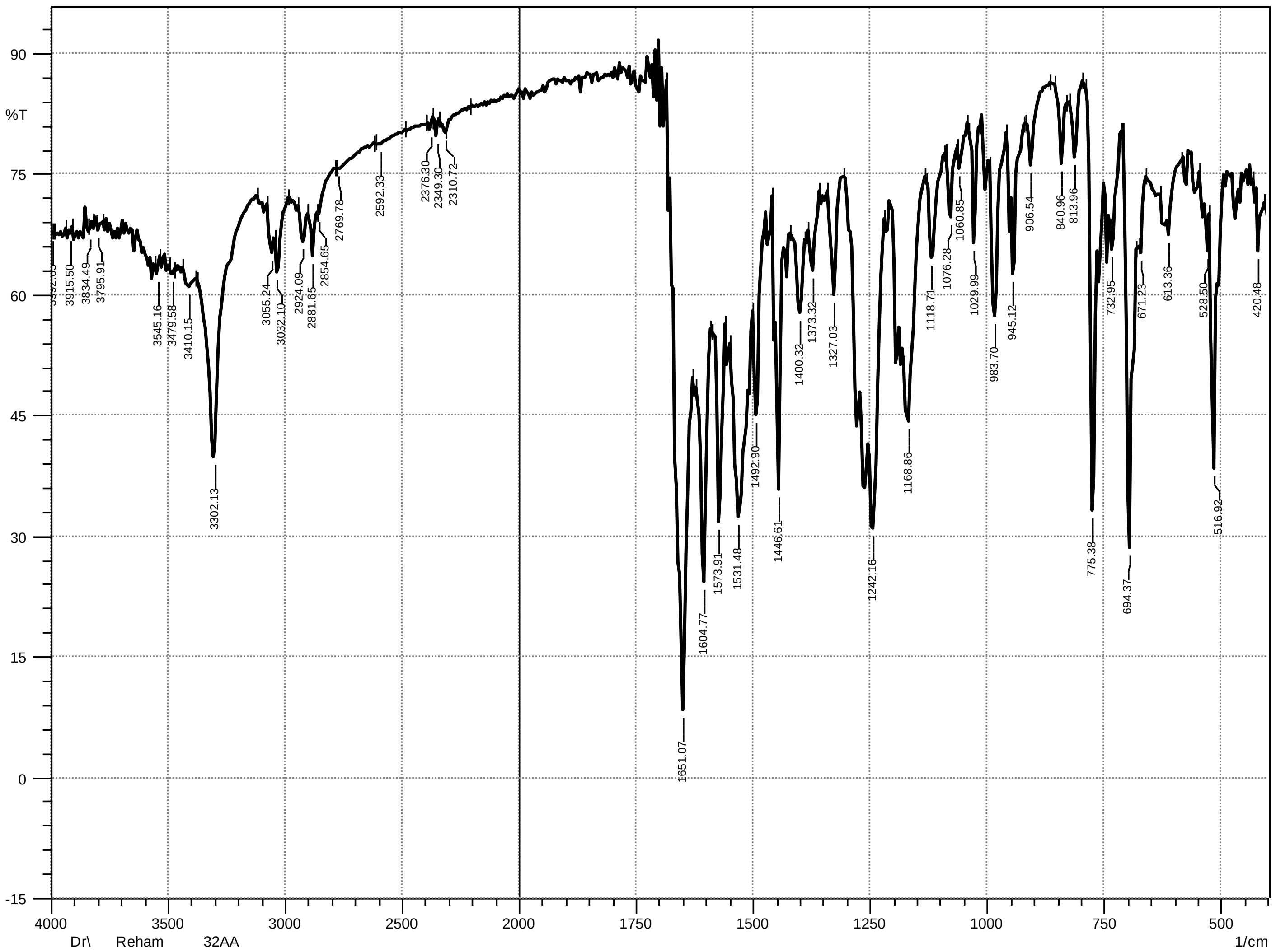

Fig. S13. IR spectrum of compound **28** (KBr pellet).

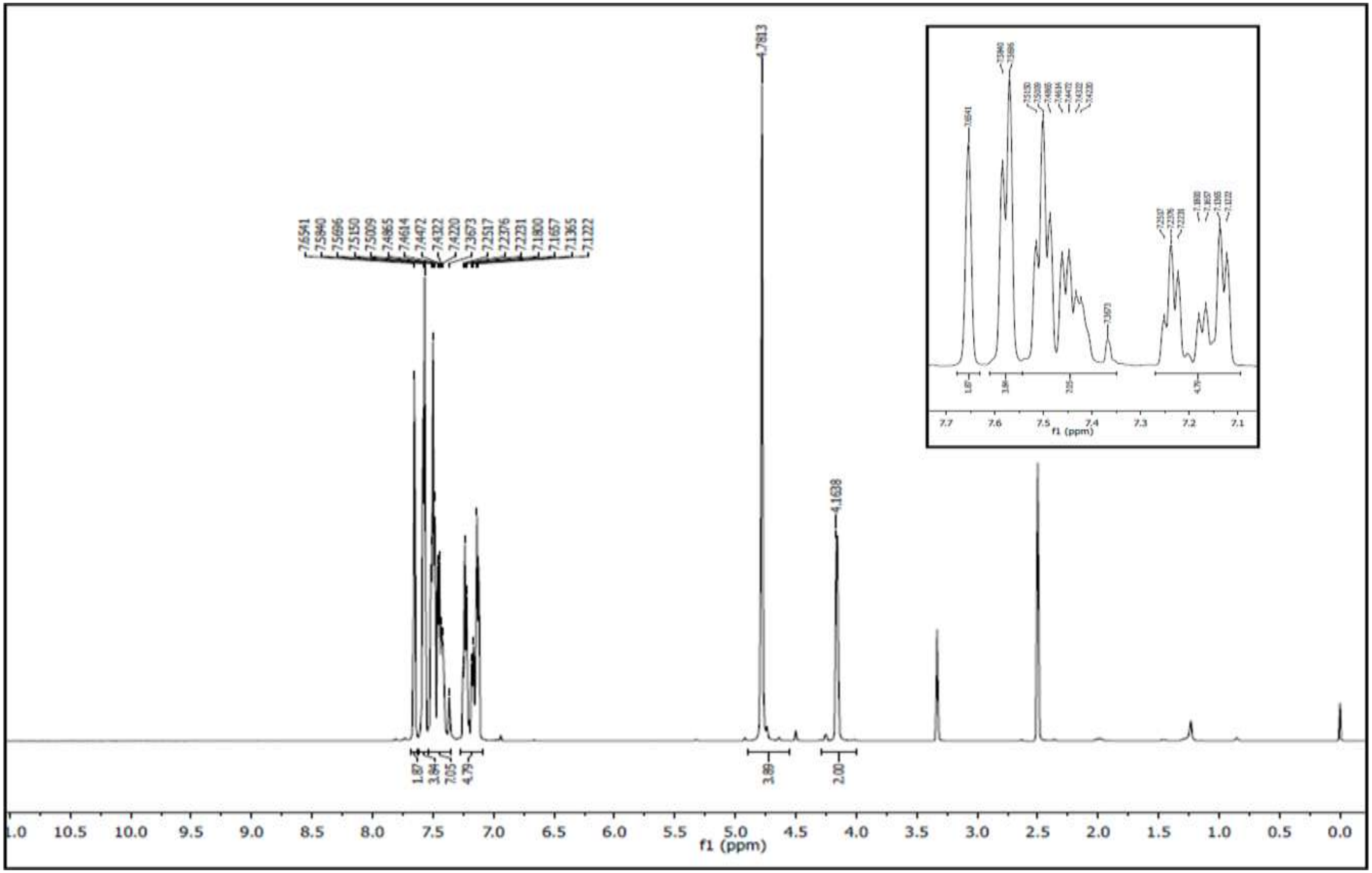

Fig. S14. ^{1}H-NMR spectrum of compound **28** in DMSO-d_6.

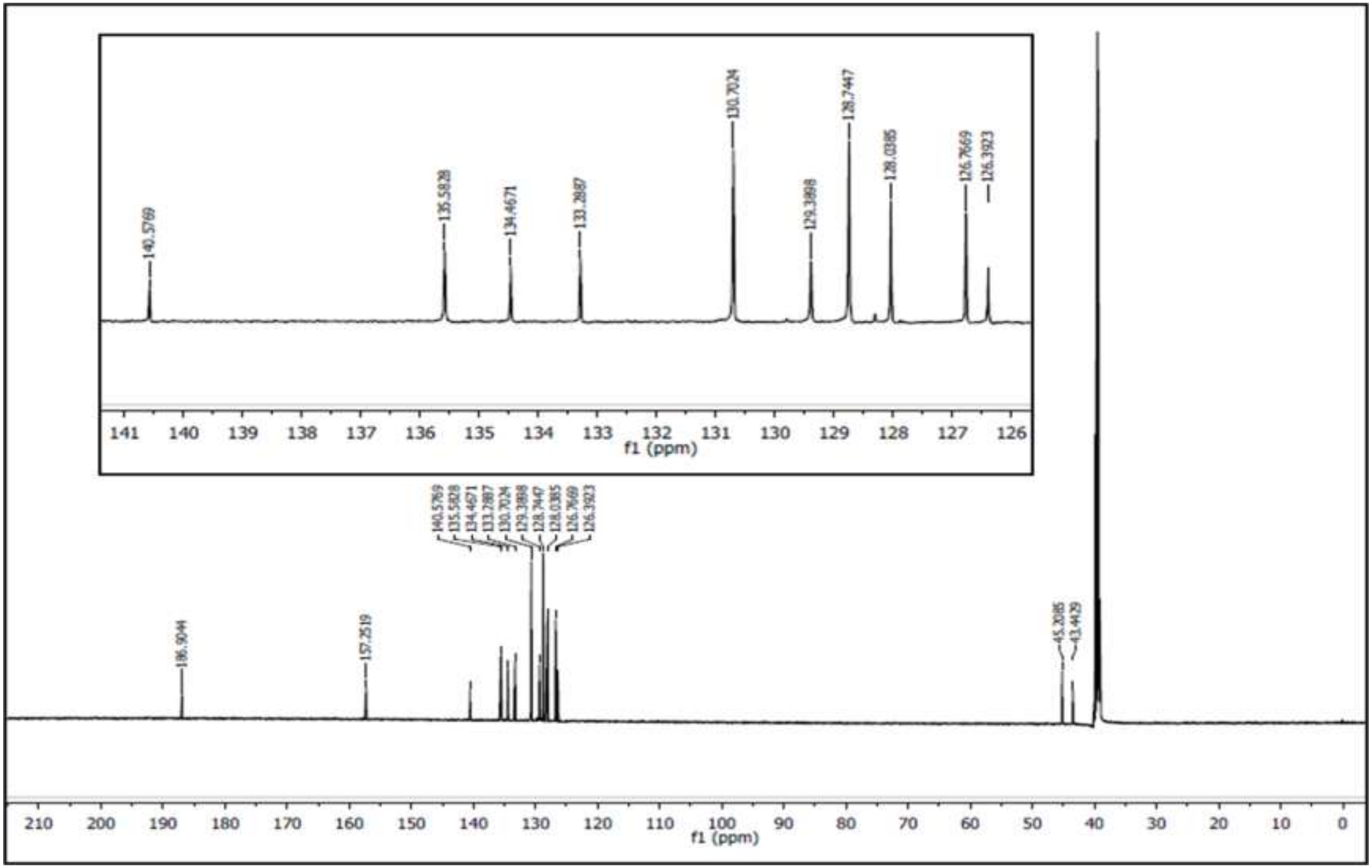

Fig. S15. ^{13}C-NMR spectrum of compound **28** in DMSO-d_6.

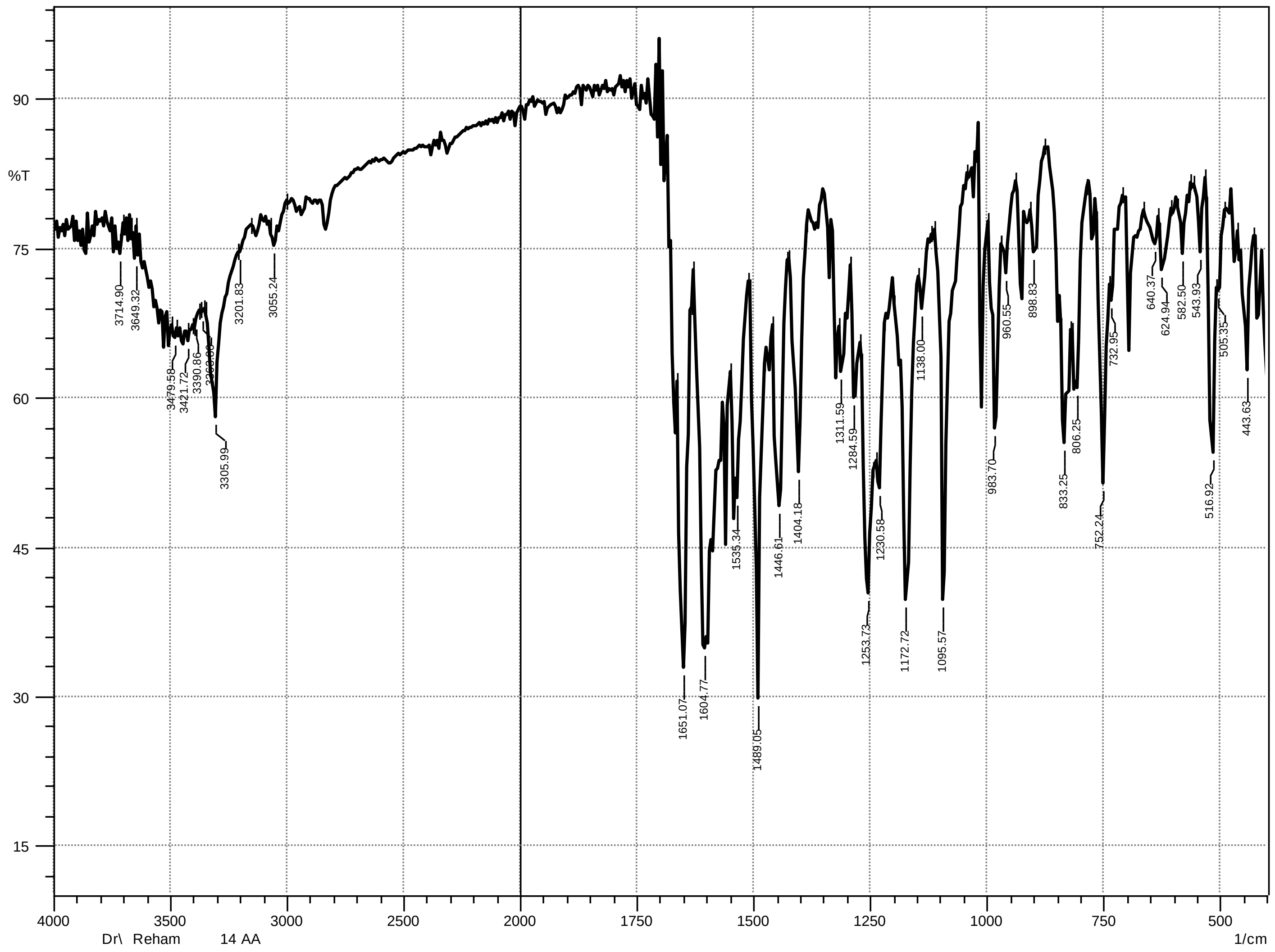

Fig. S16. IR spectrum of compound **29** (KBr pellet).

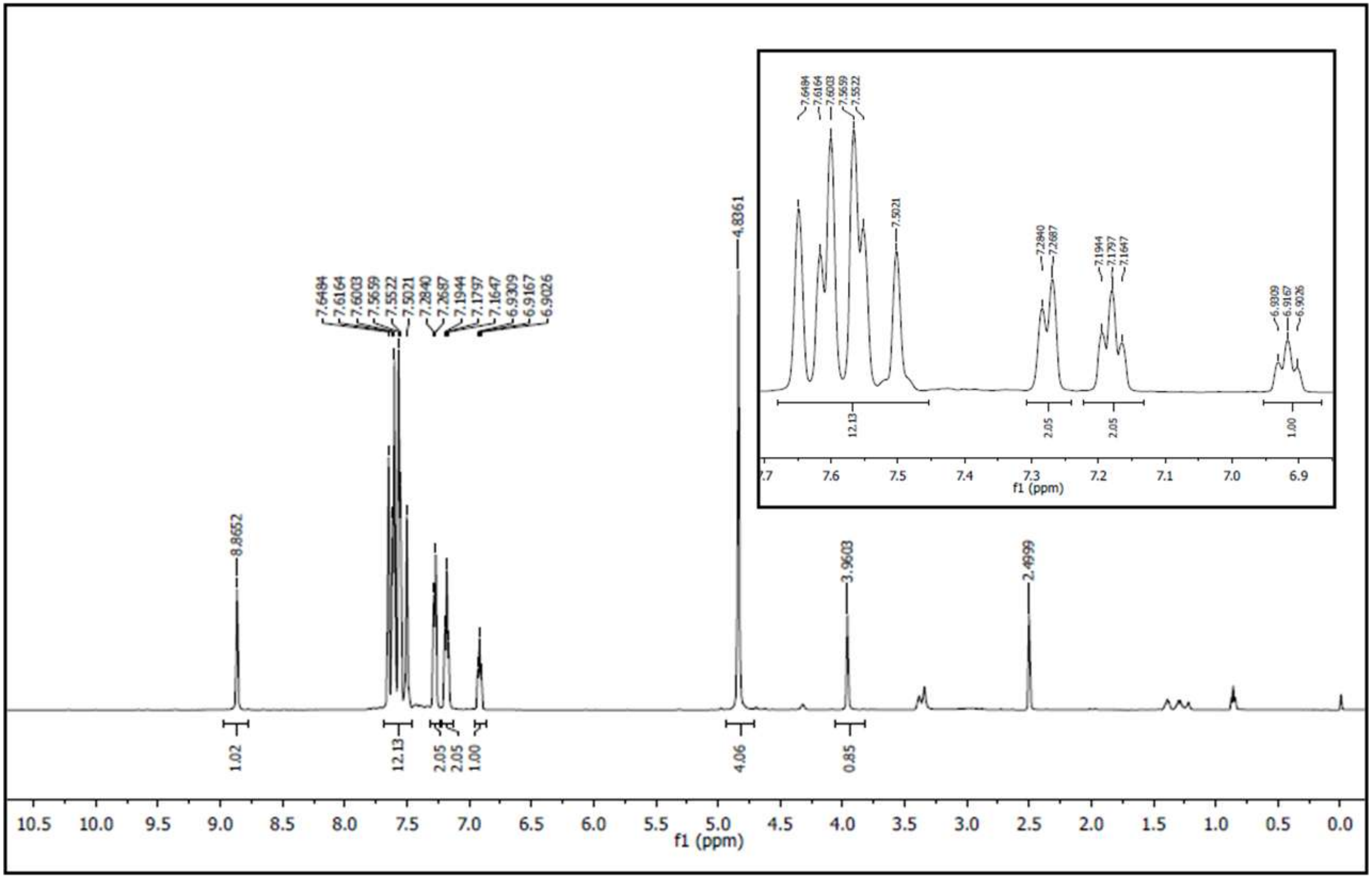

Fig. S17. ^{1}H-NMR spectrum of compound **29** in DMSO-d_6.

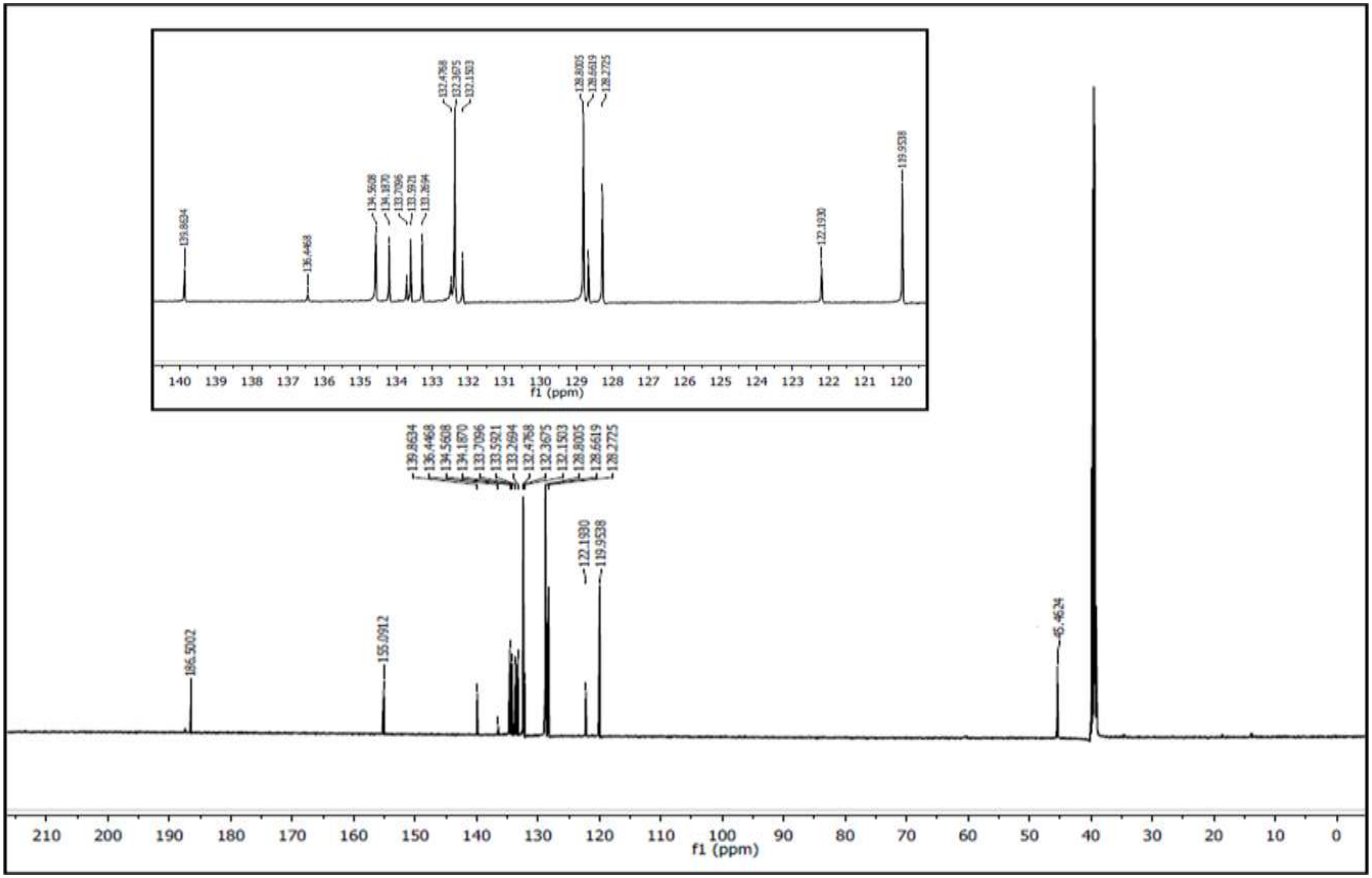

Fig. S18. ^{13}C-NMR spectrum of compound **29** in DMSO-d_6.

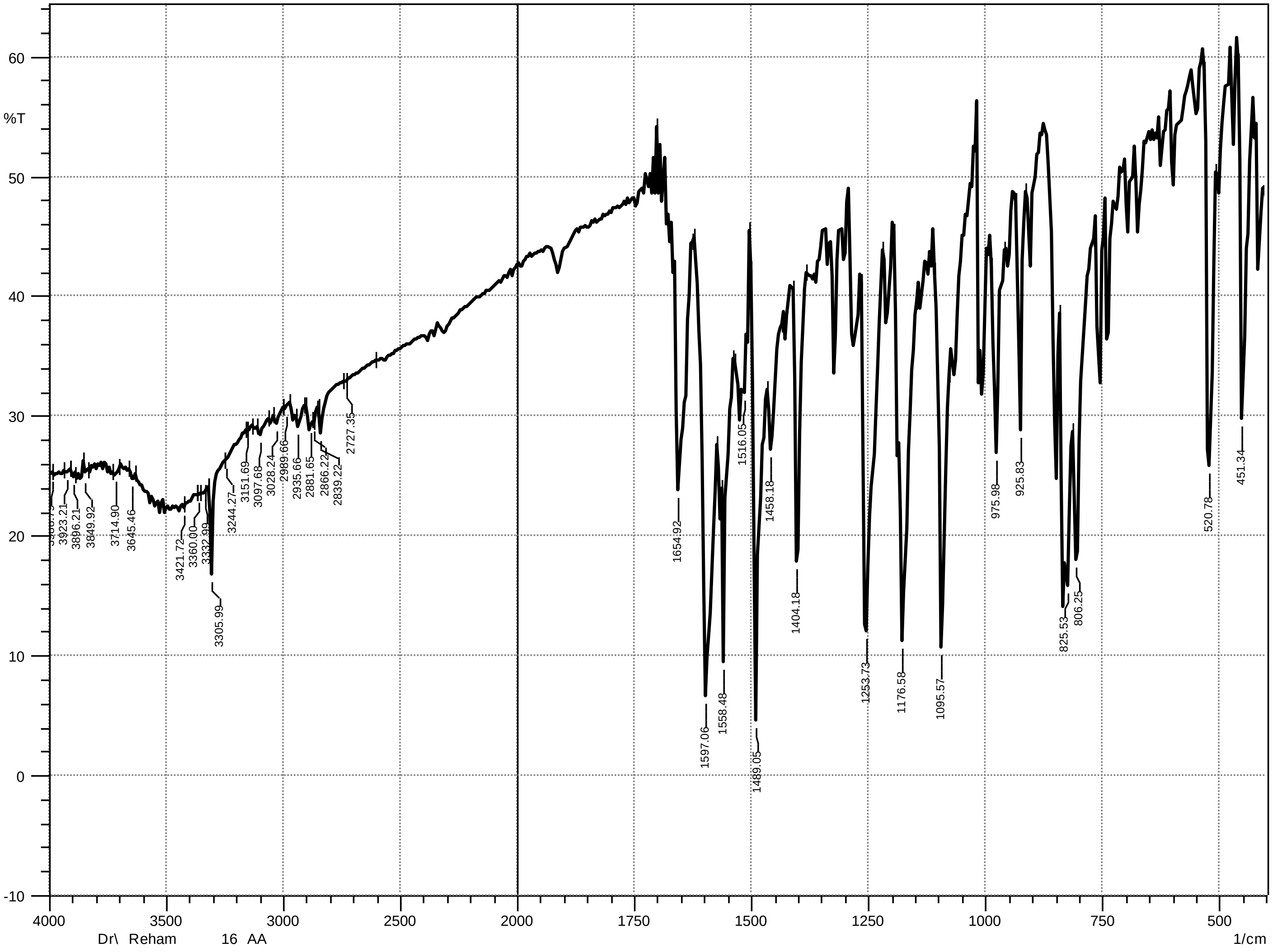

Fig. S19. IR spectrum of compound **30** (KBr pellet).

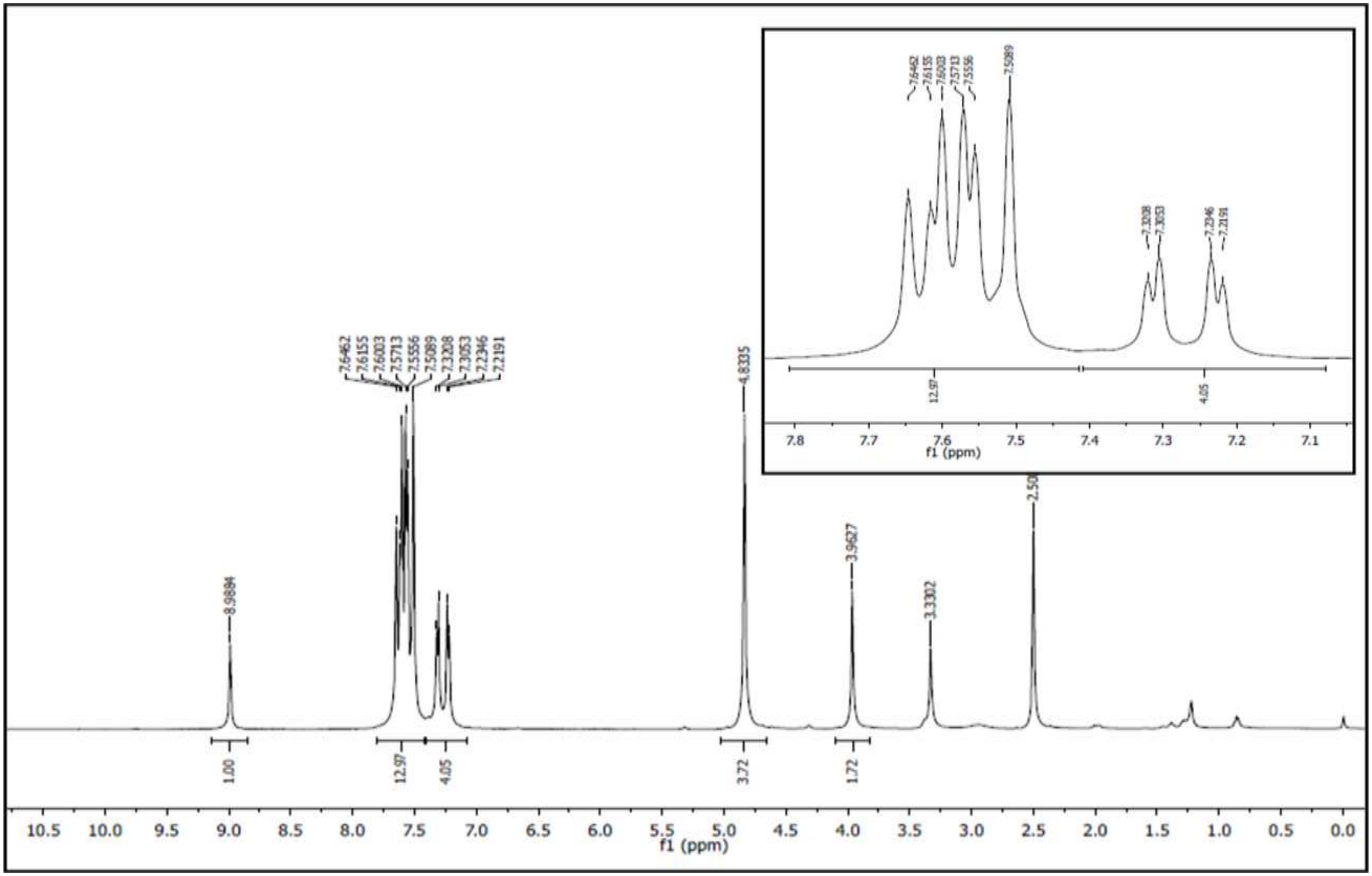

Fig. S20. ^{1}H-NMR spectrum of compound **30** in DMSO-d_6.

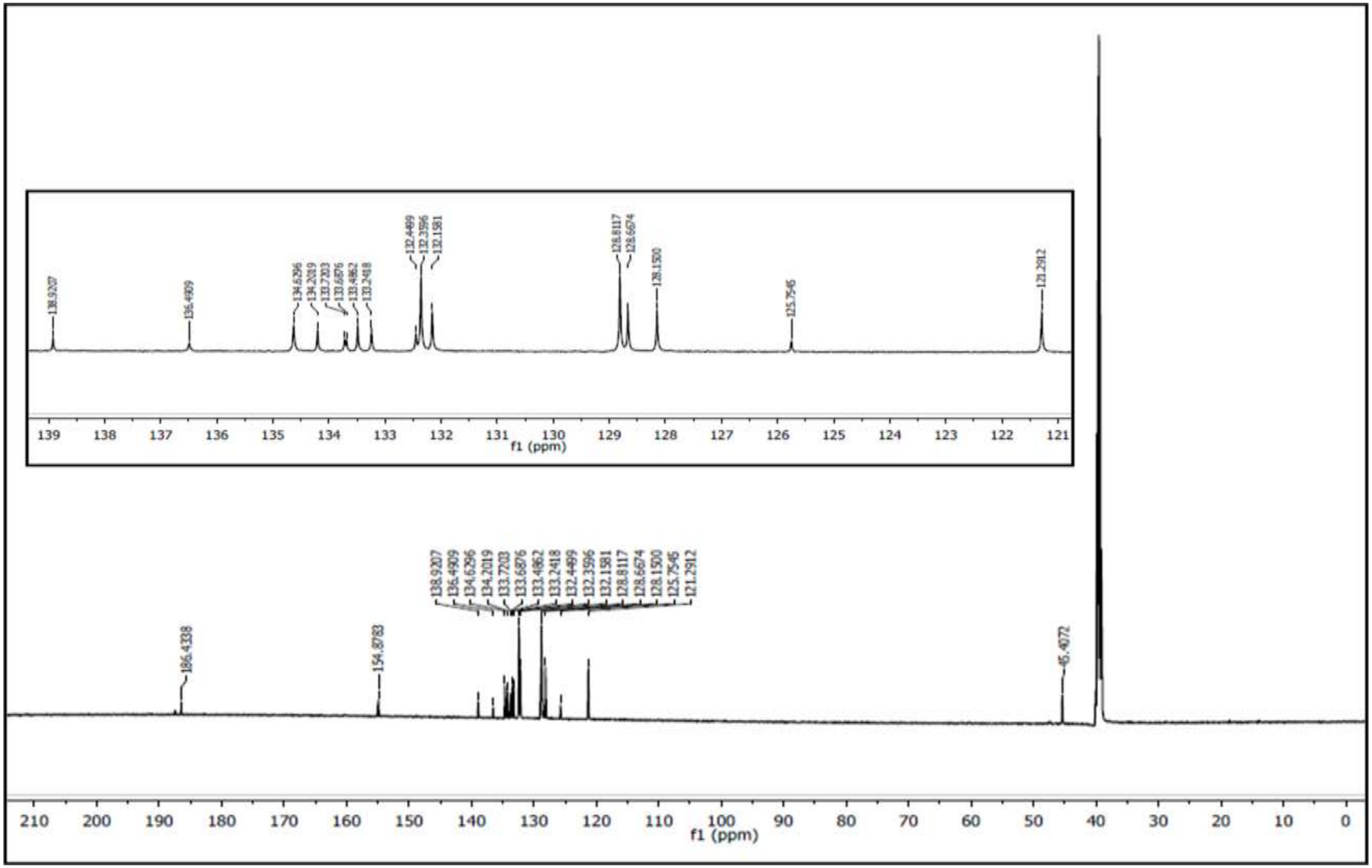

Fig. S21. ^{13}C-NMR spectrum of compound **30** in DMSO-d_6.

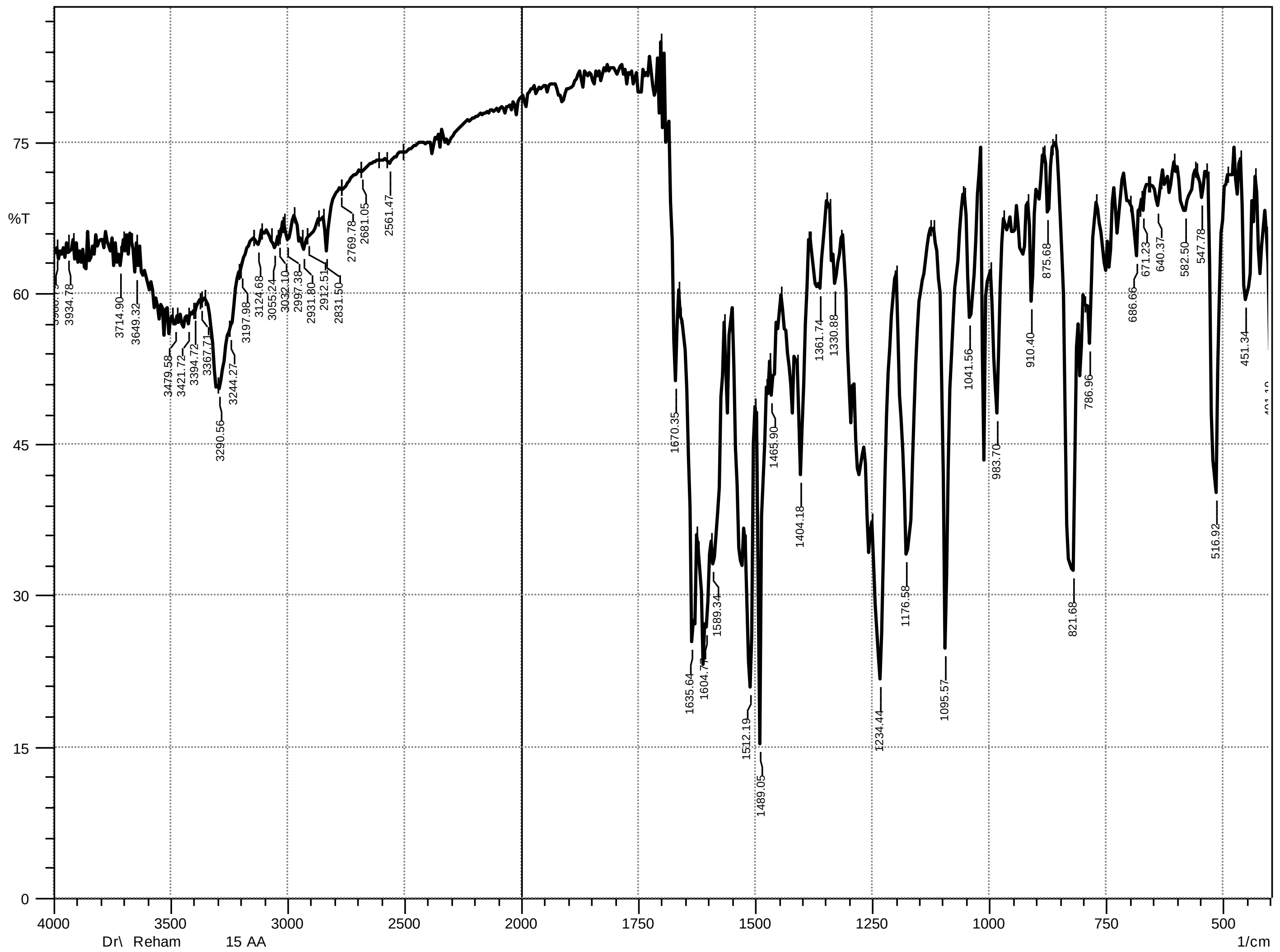

Fig. S22. IR spectrum of compound **31** (KBr pellet).

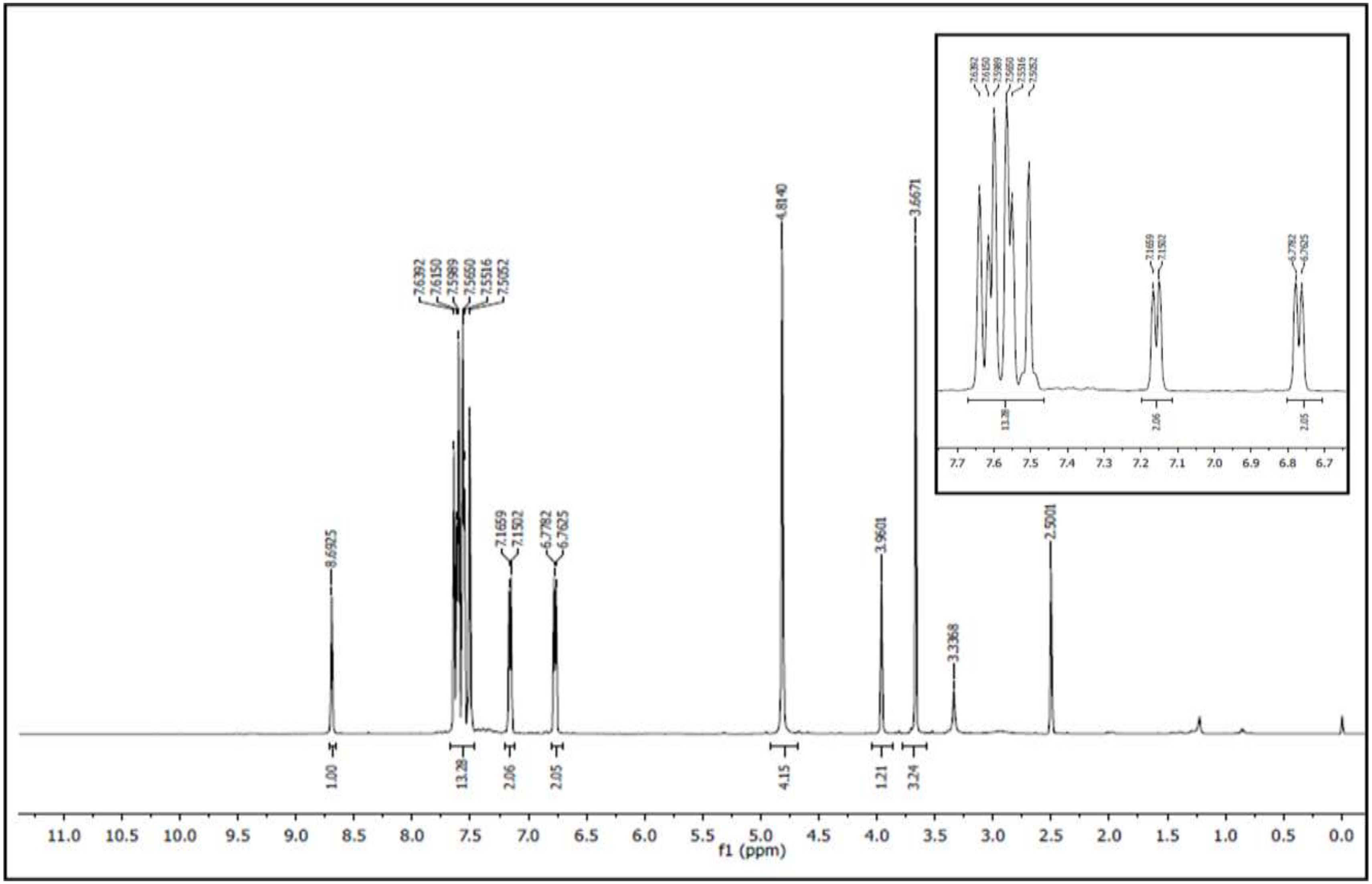

Fig. S23. ^{1}H-NMR spectrum of compound **31** in DMSO-d_6.

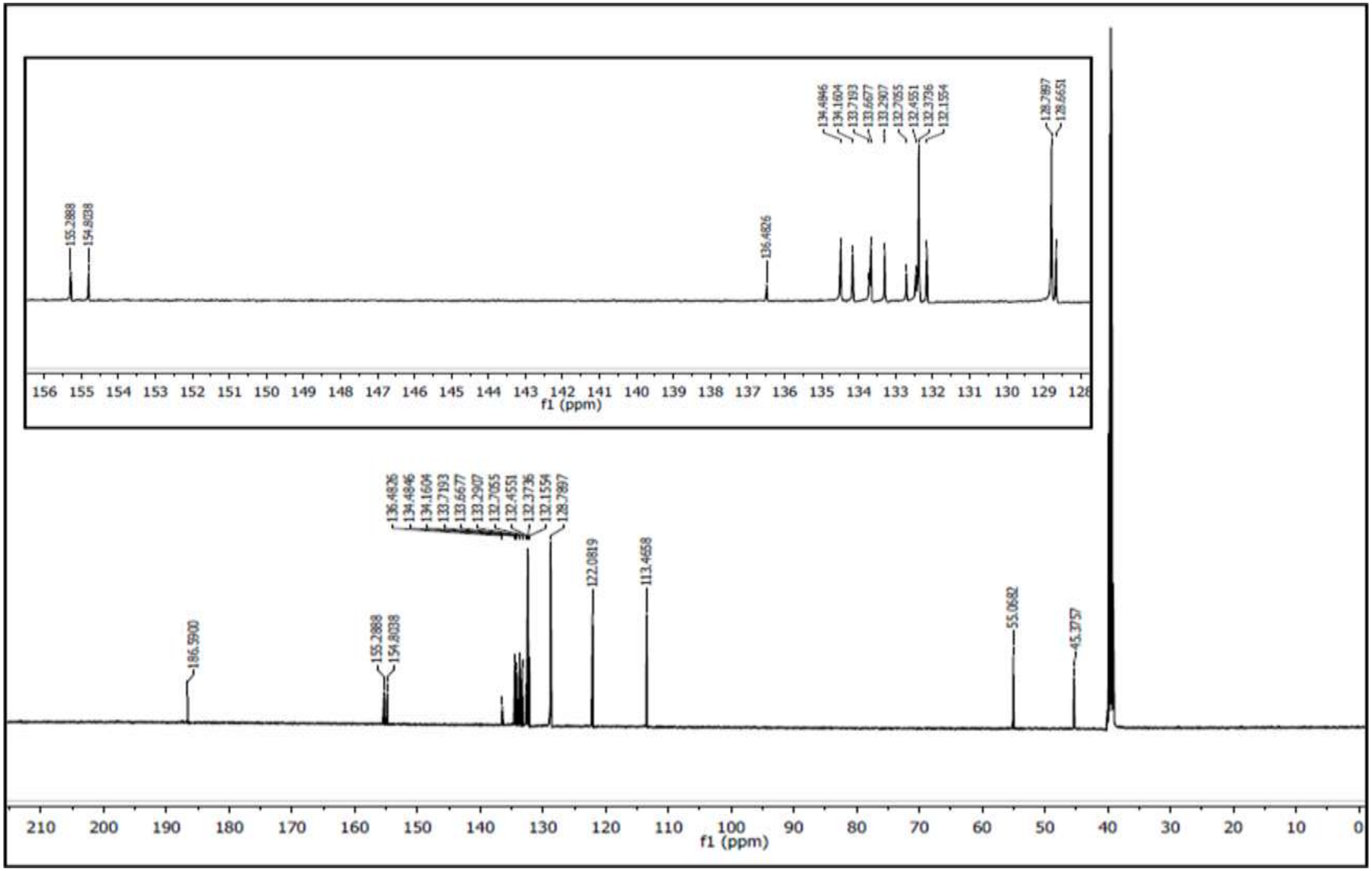

Fig. S24. ^{13}C-NMR spectrum of compound **31** in DMSO-d_6.

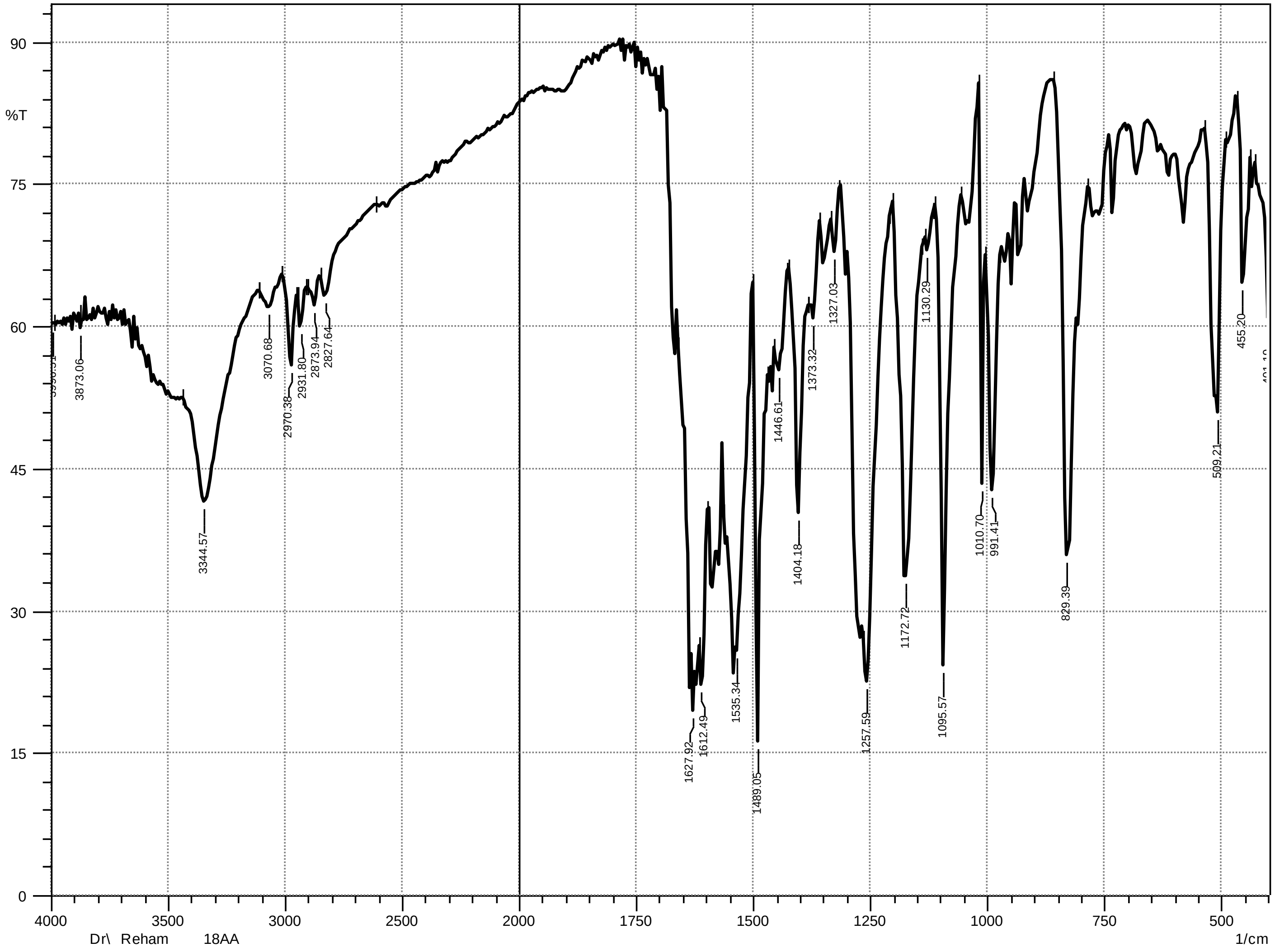

Fig. S25. IR spectrum of compound **32** (KBr pellet).

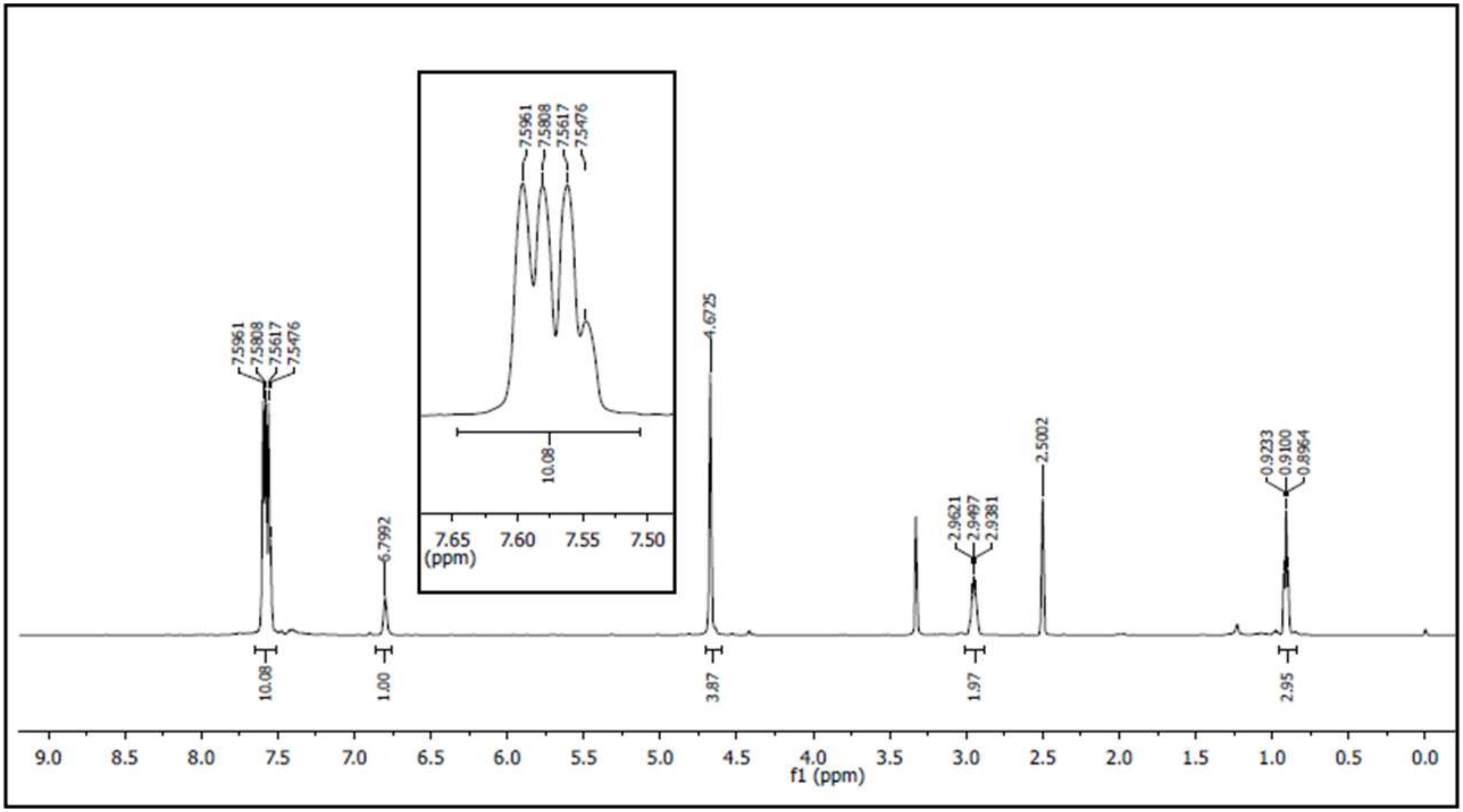

Fig. S26. ^{1}H-NMR spectrum of compound **32** in DMSO-d_6.

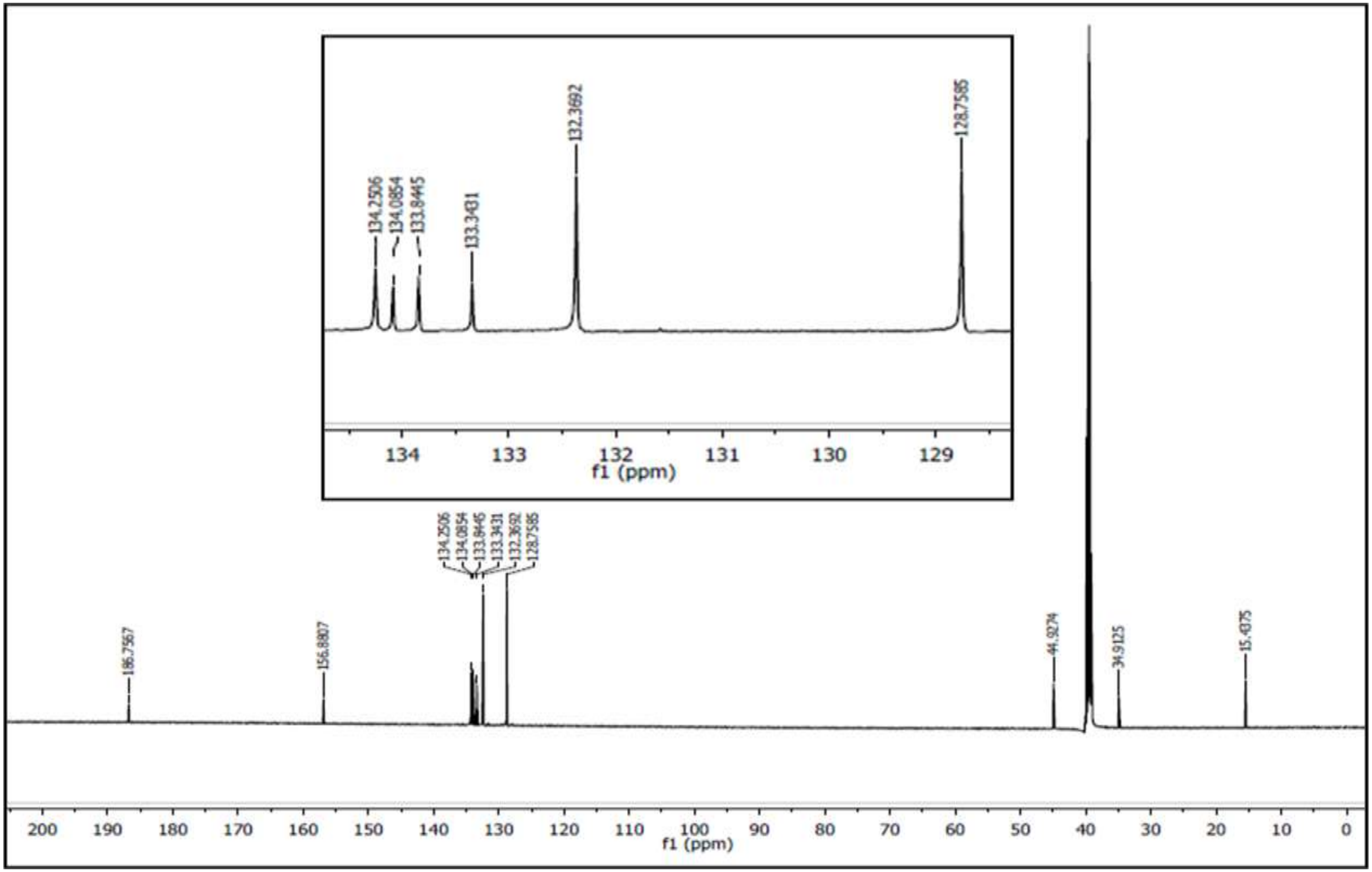

Fig. S27. ^{13}C-NMR spectrum of compound **32** in DMSO-d_6.

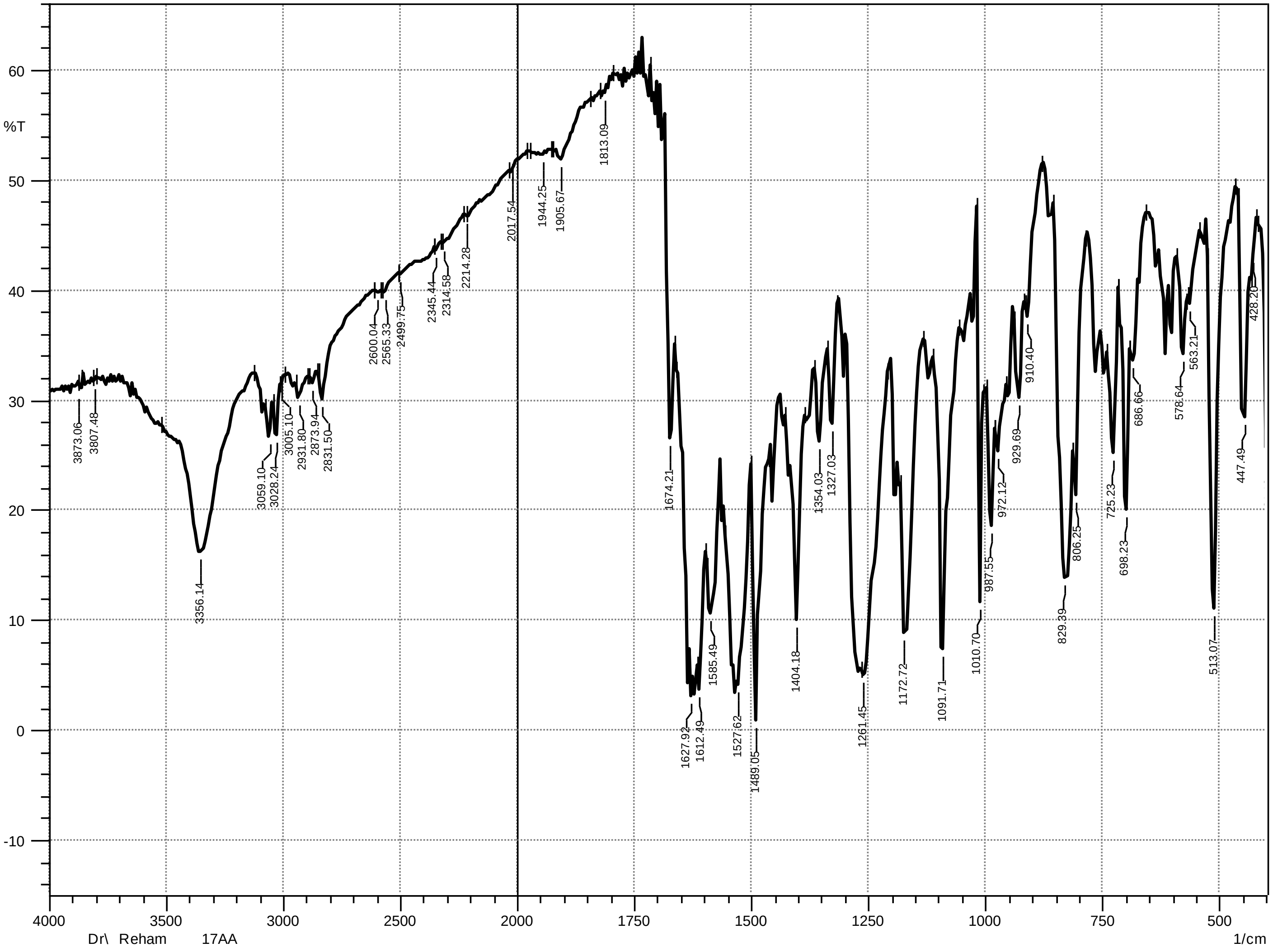

Fig. S28. IR spectrum of compound **33** (KBr pellet).

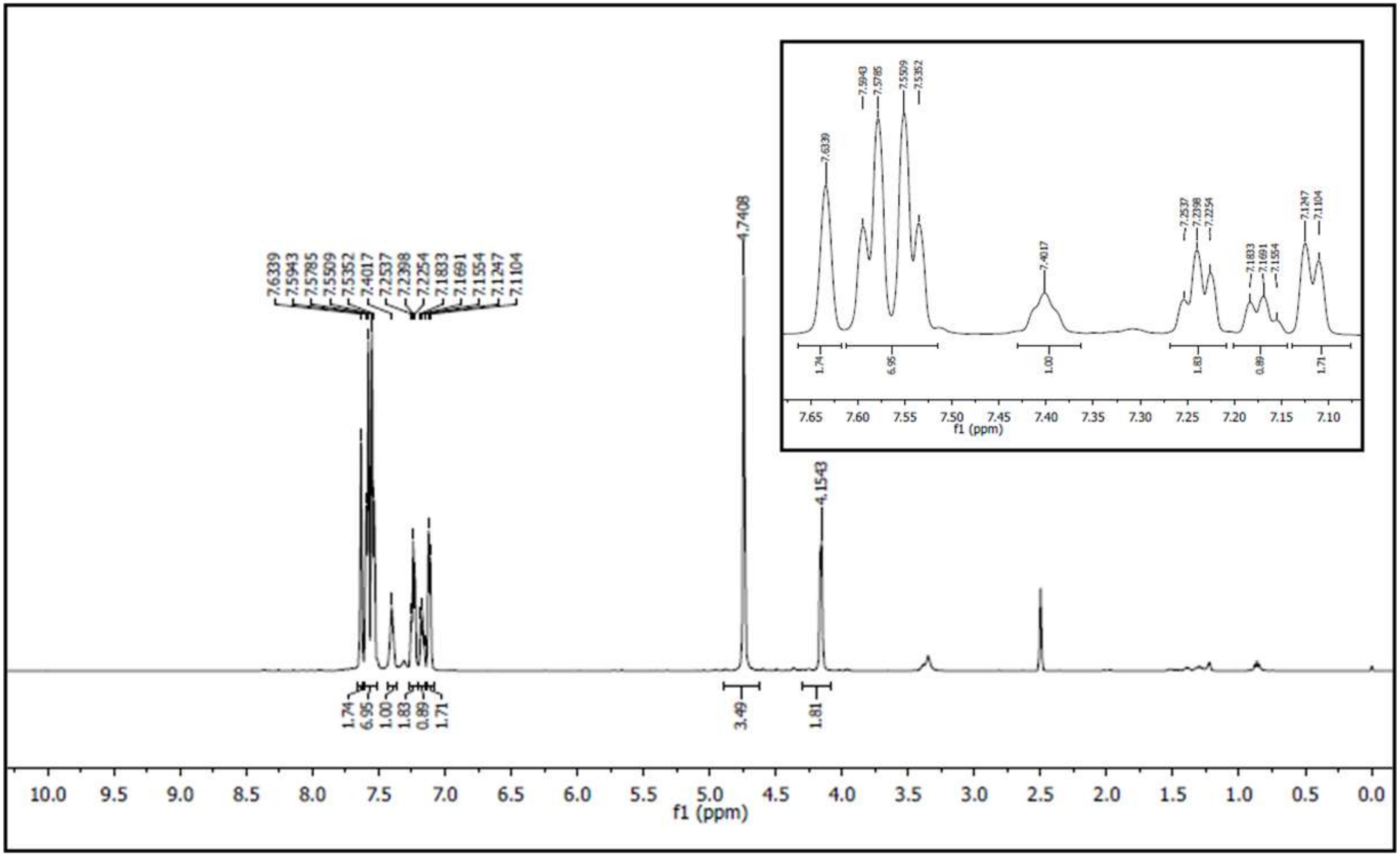

Fig. S29. ^{1}H-NMR spectrum of compound **33** in DMSO-d_6.

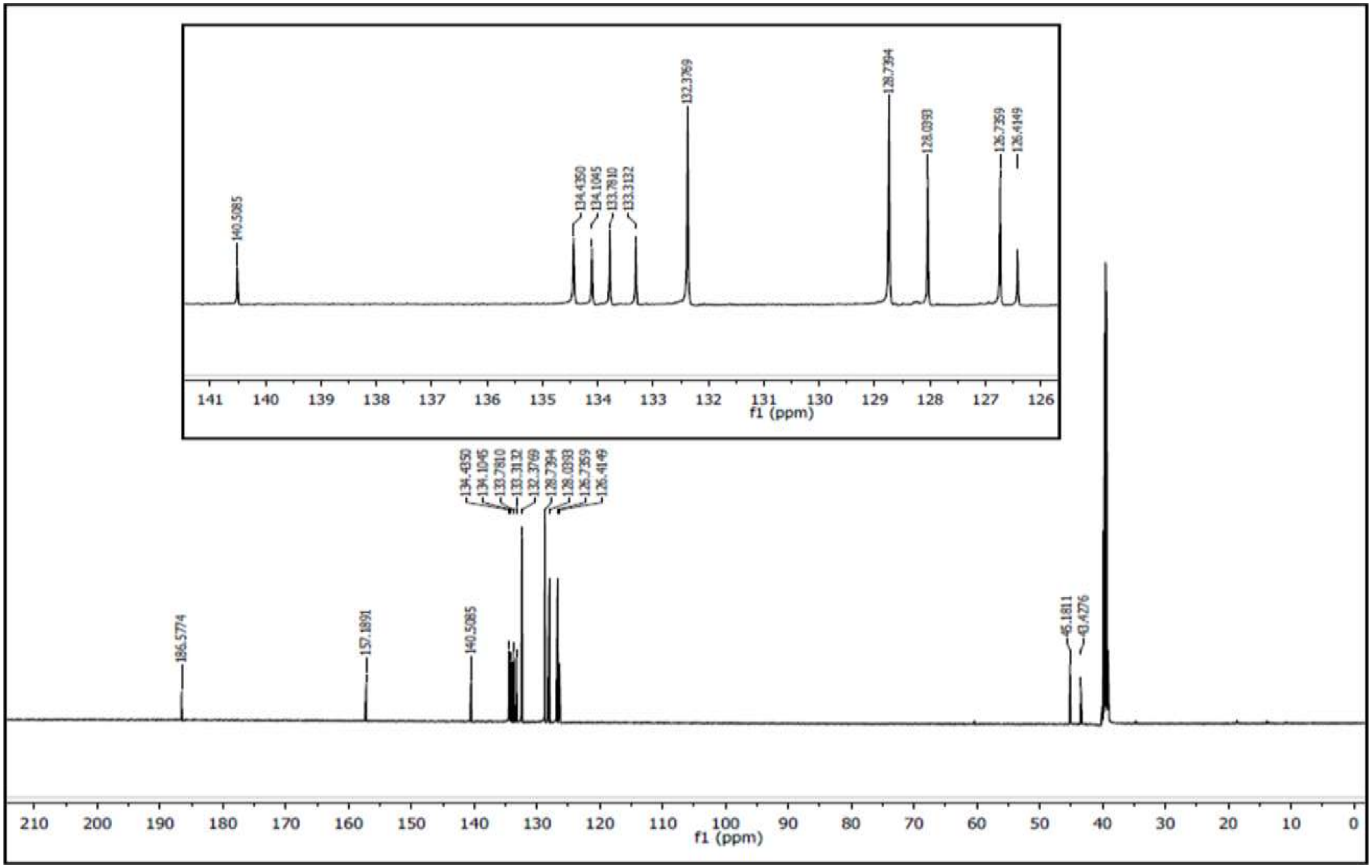

Fig. S30. ^{13}C-NMR spectrum of compound **33** in DMSO-d_6.

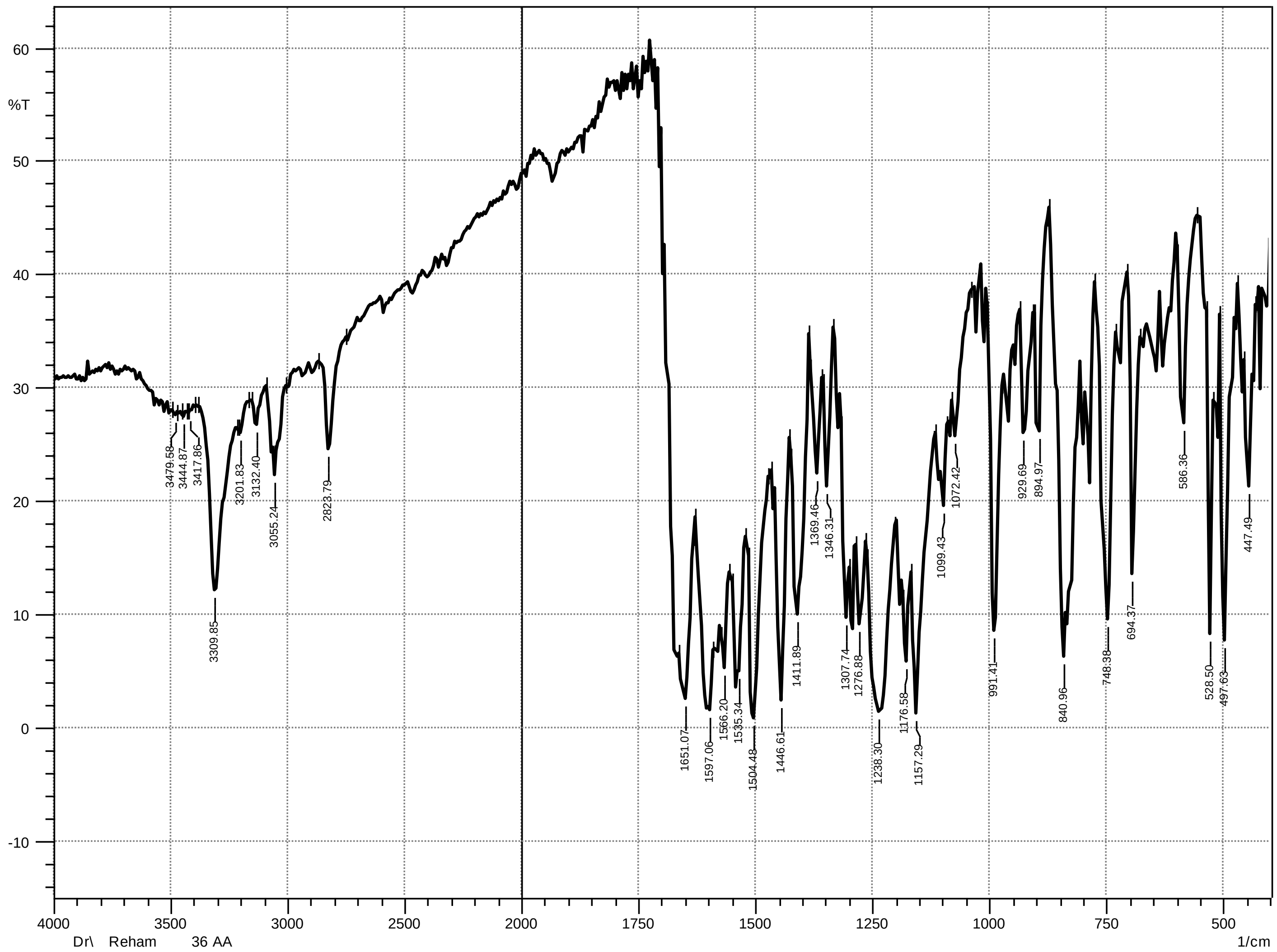

Fig. S31. IR spectrum of compound **34** (KBr pellet).

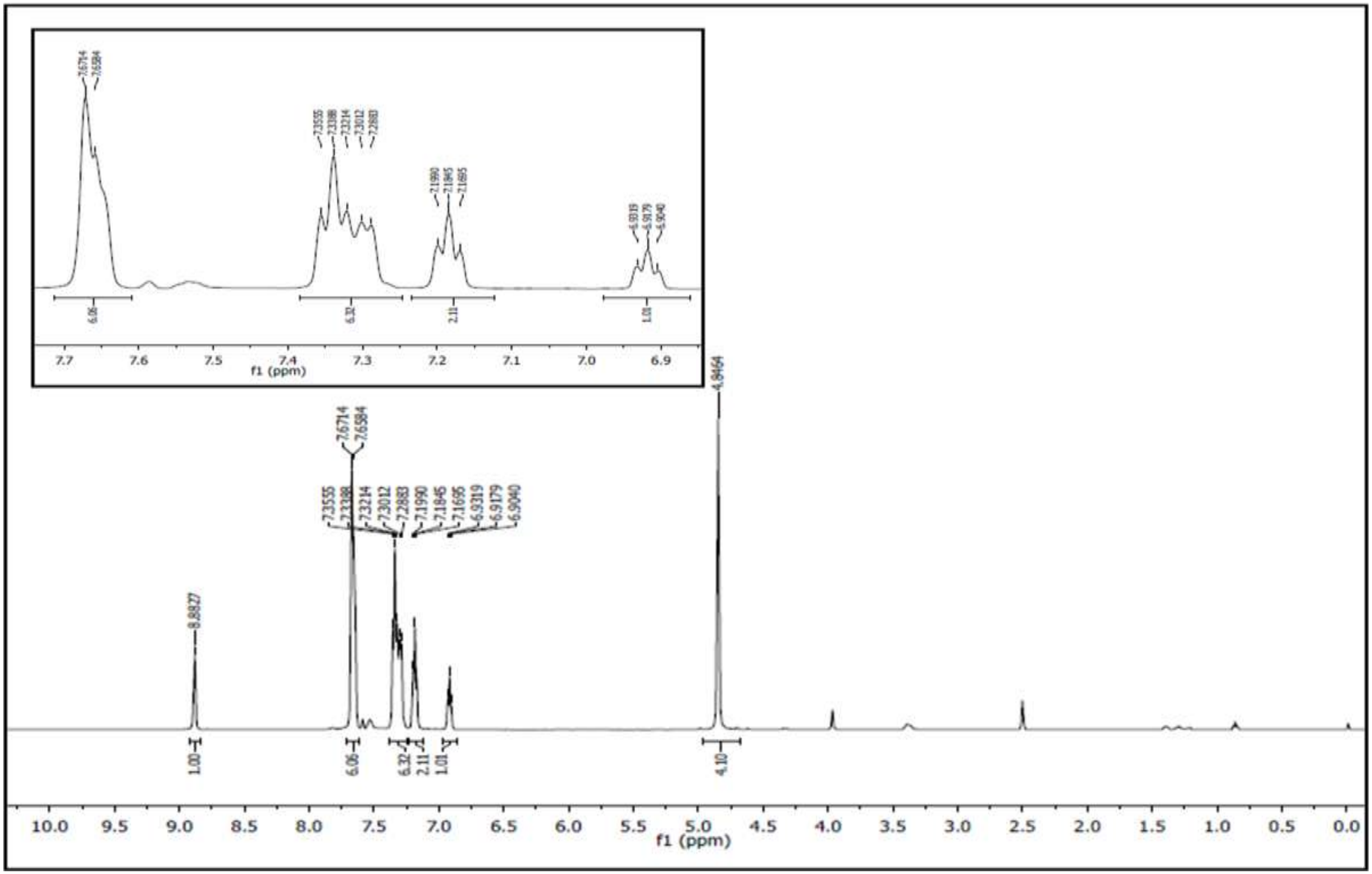

Fig. S32. ^{1}H-NMR spectrum of compound **34** in DMSO-d_6.

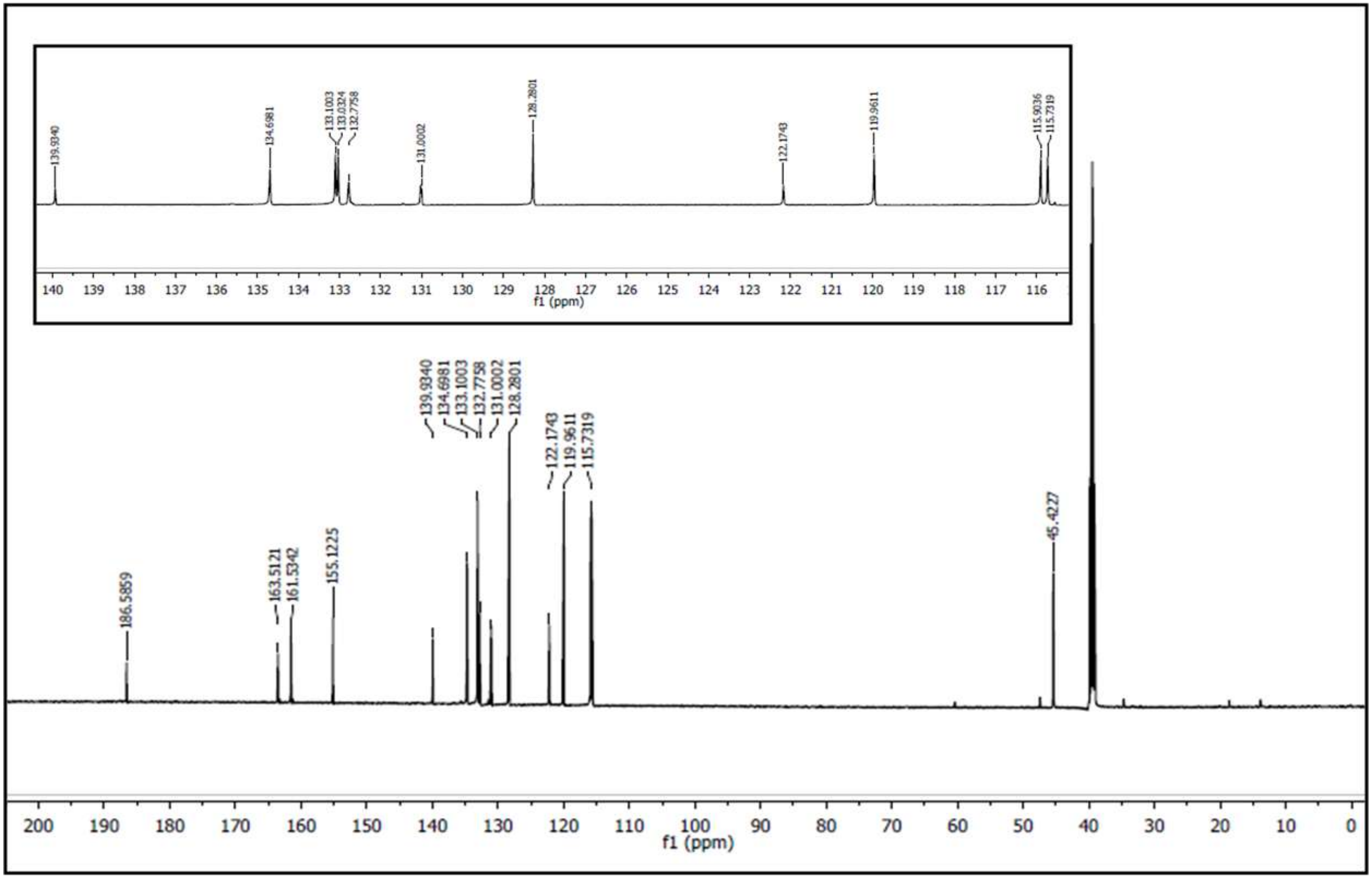

Fig. S33. ^{13}C-NMR spectrum of compound **34** in DMSO-d_6.

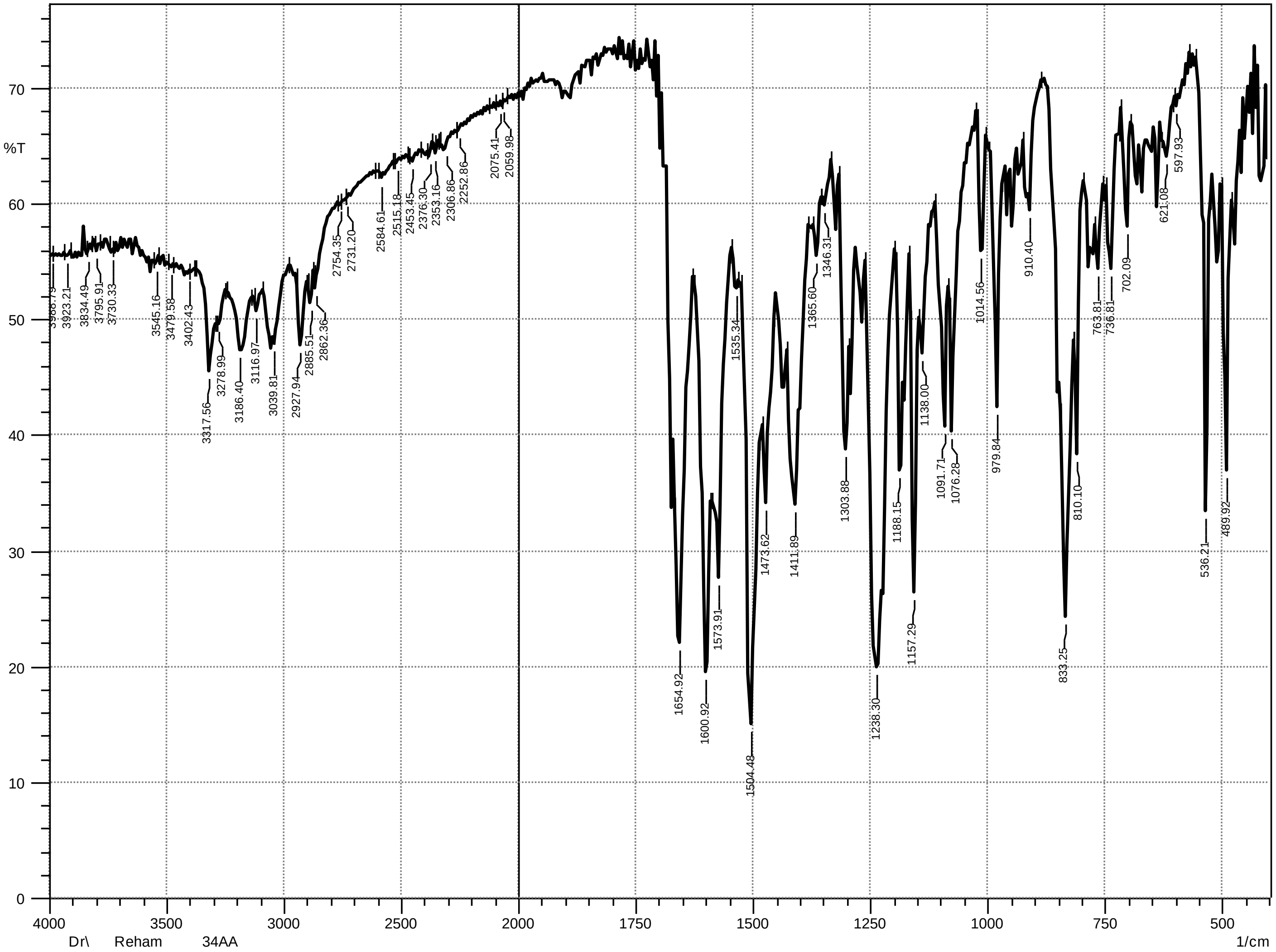

Fig. S34. IR spectrum of compound **35** (KBr pellet).

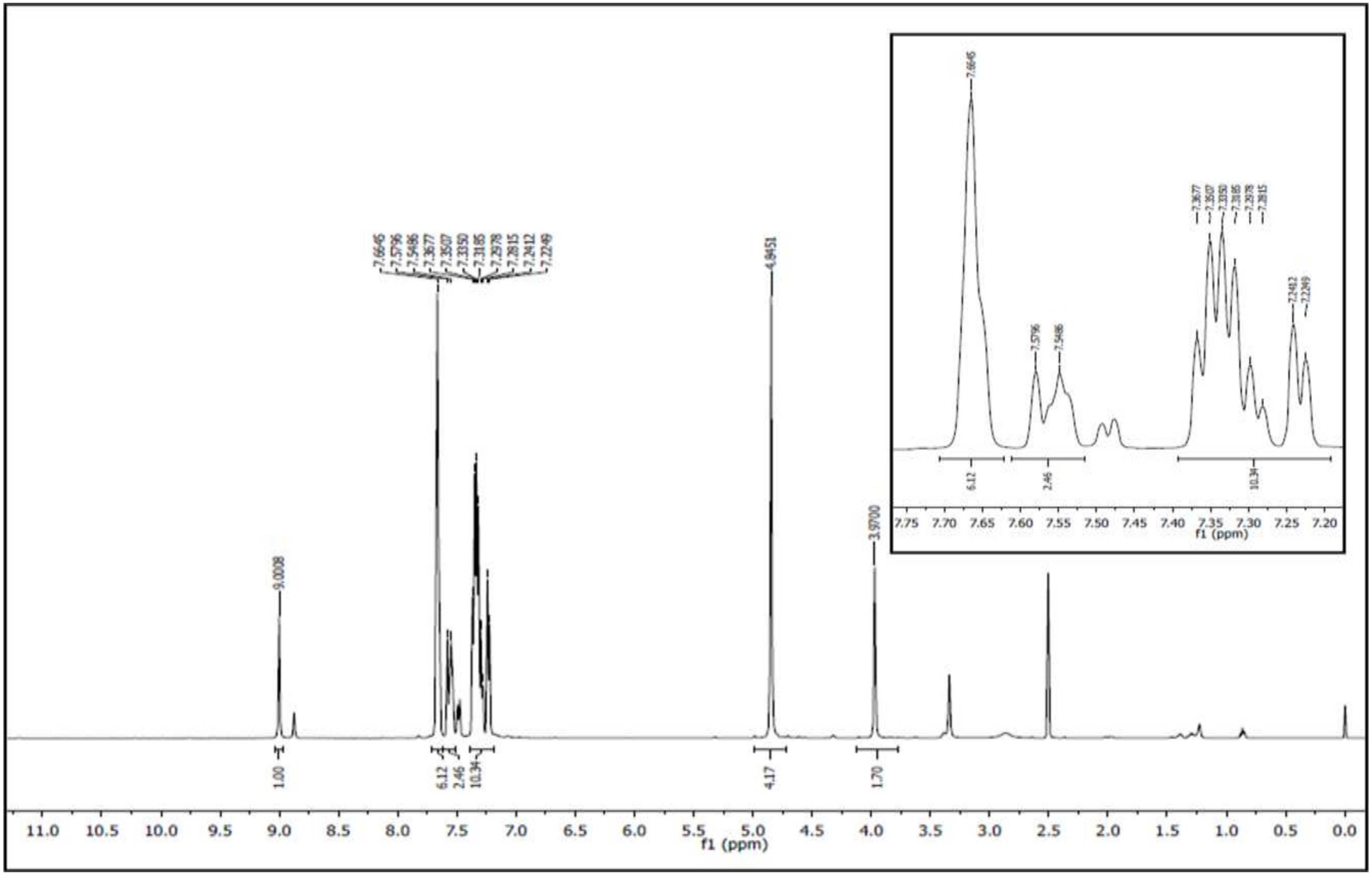

Fig. S35. ^{1}H-NMR spectrum of compound **35** in DMSO-d_6.

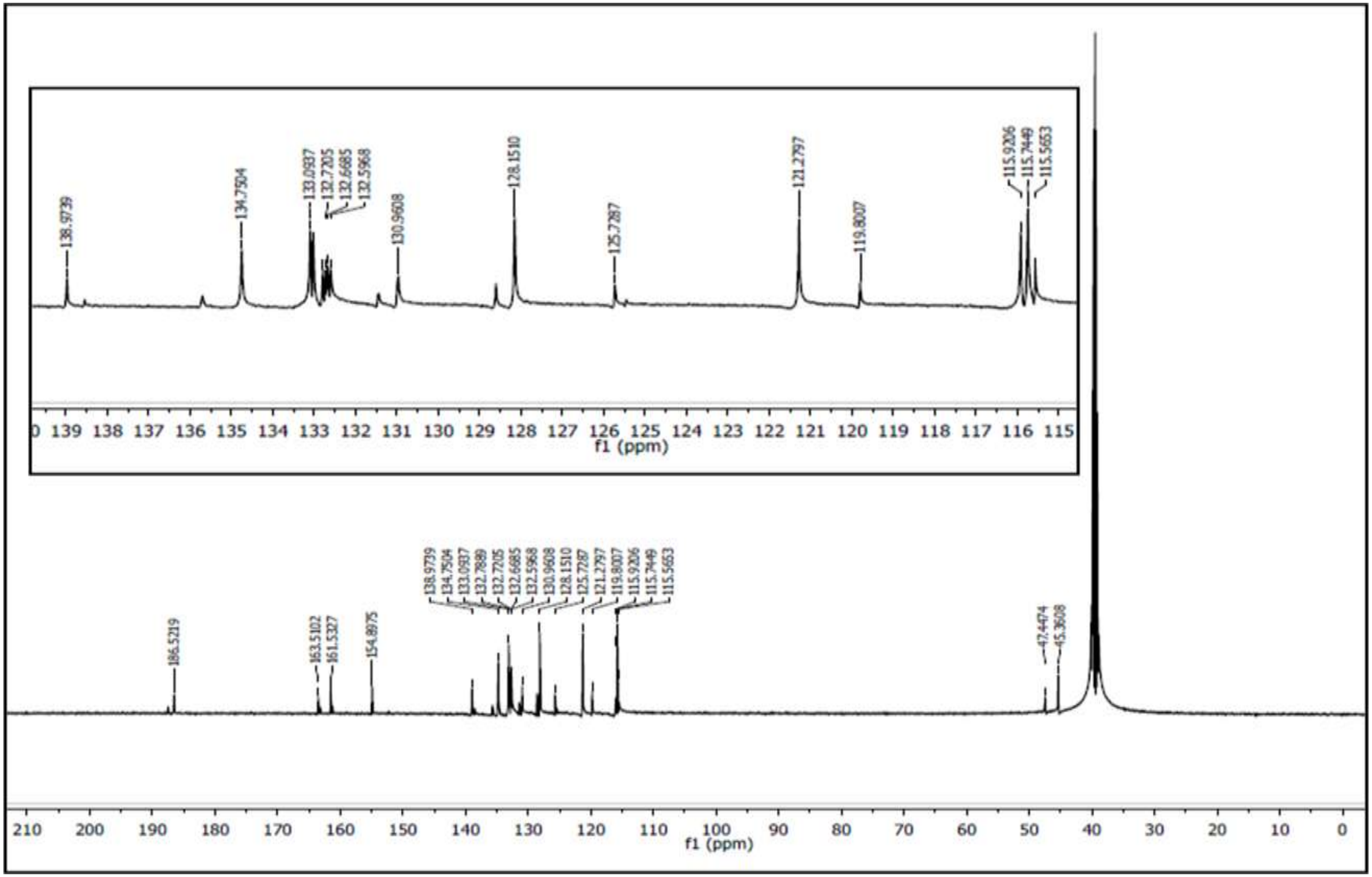

Fig. S36. ^{13}C-NMR spectrum of compound **35** in DMSO-d_6.

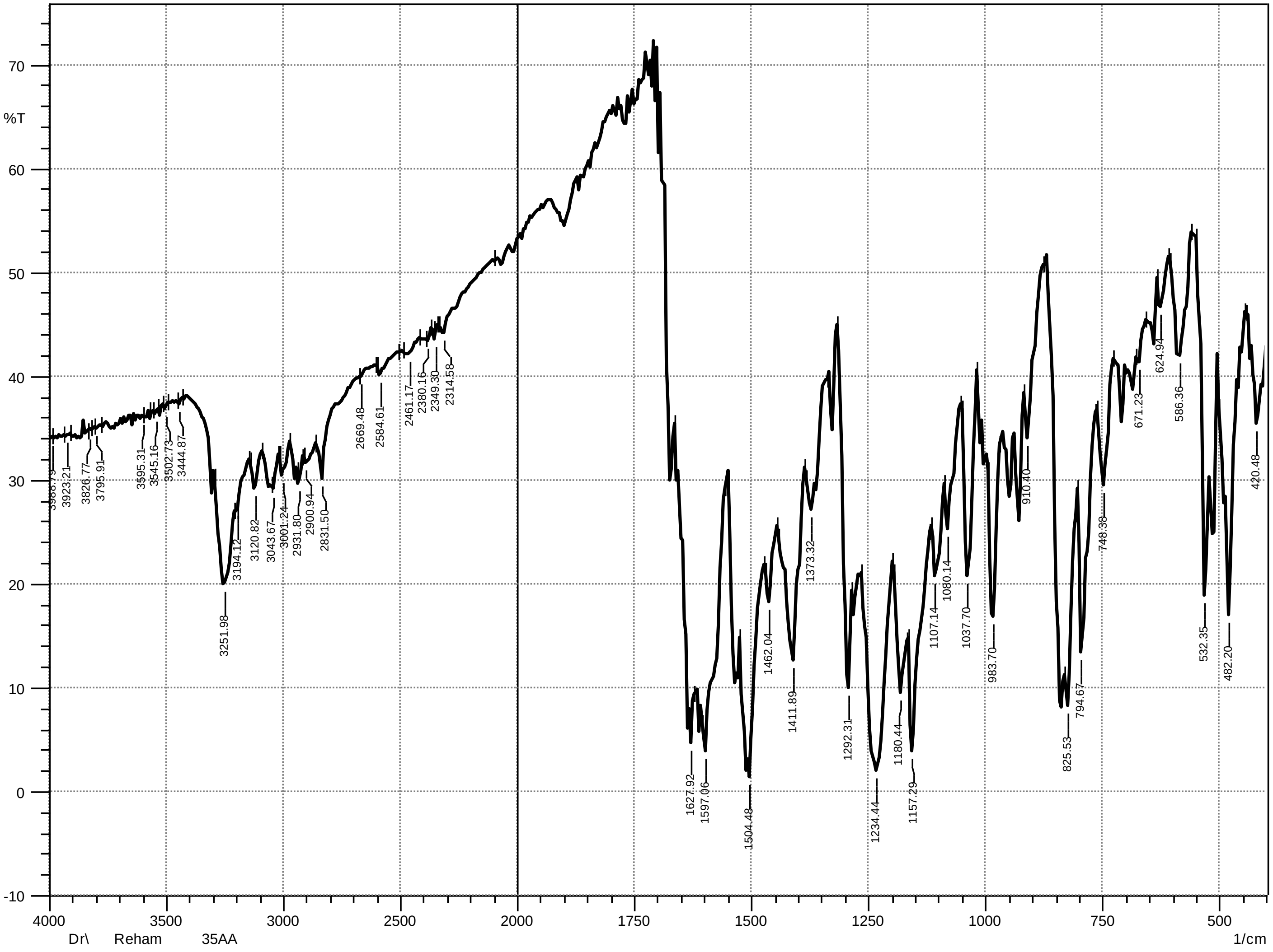

Fig. S37. IR spectrum of compound **36** (KBr pellet).

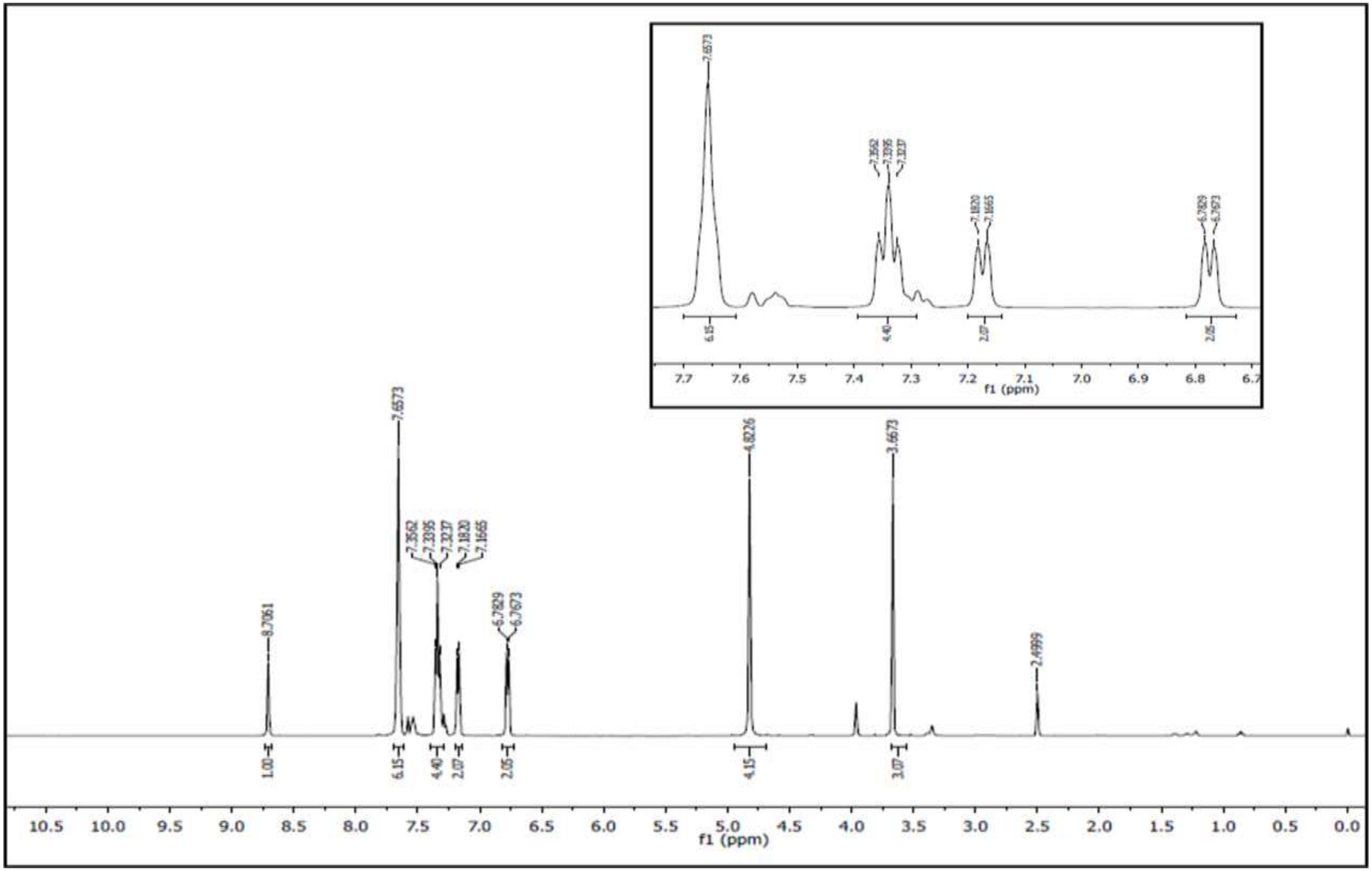

Fig. S38. ^{1}H-NMR spectrum of compound **36** in DMSO-d_6.

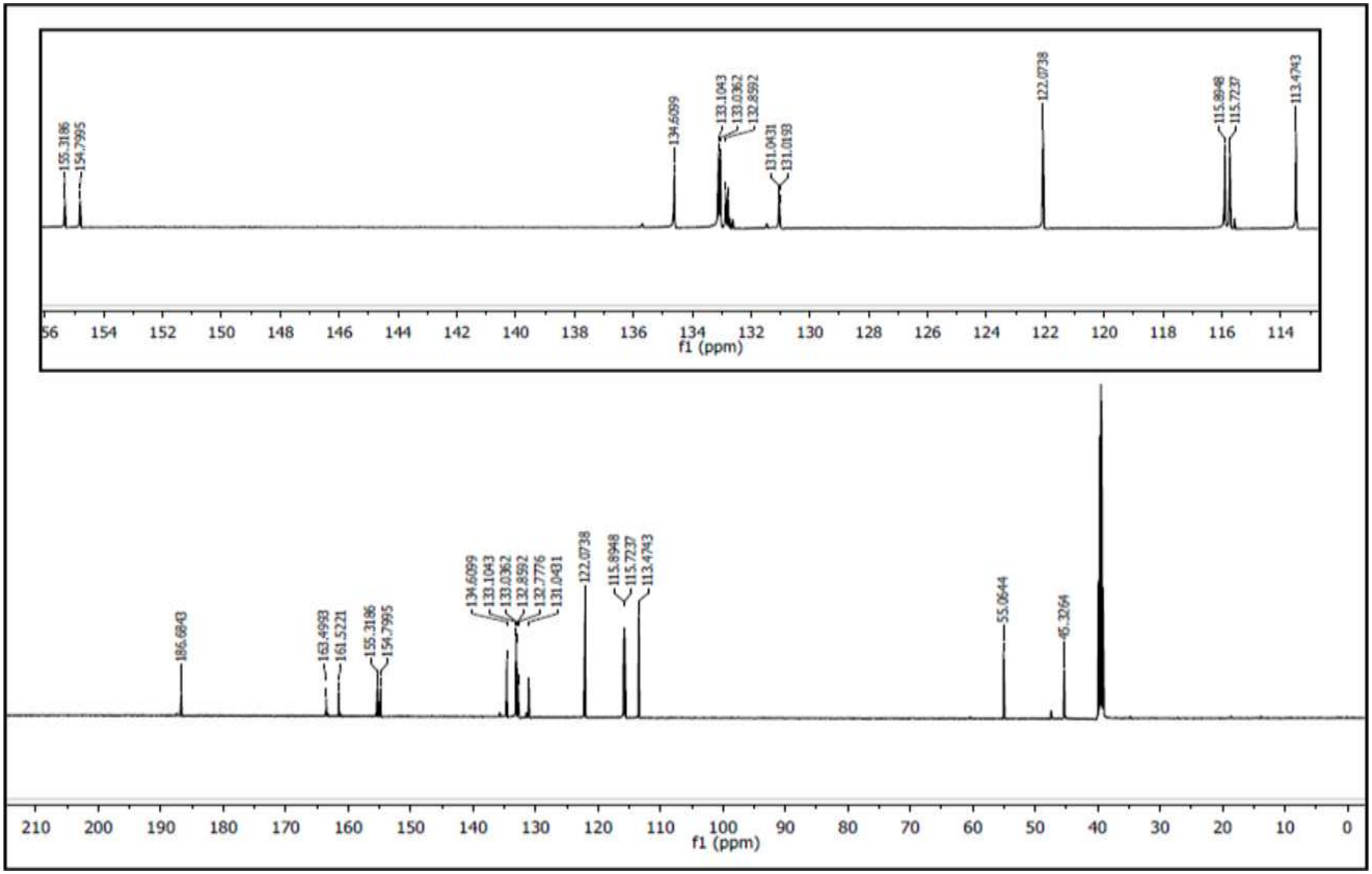

Fig. S39. ^{13}C-NMR spectrum of compound **36** in DMSO-d_6.

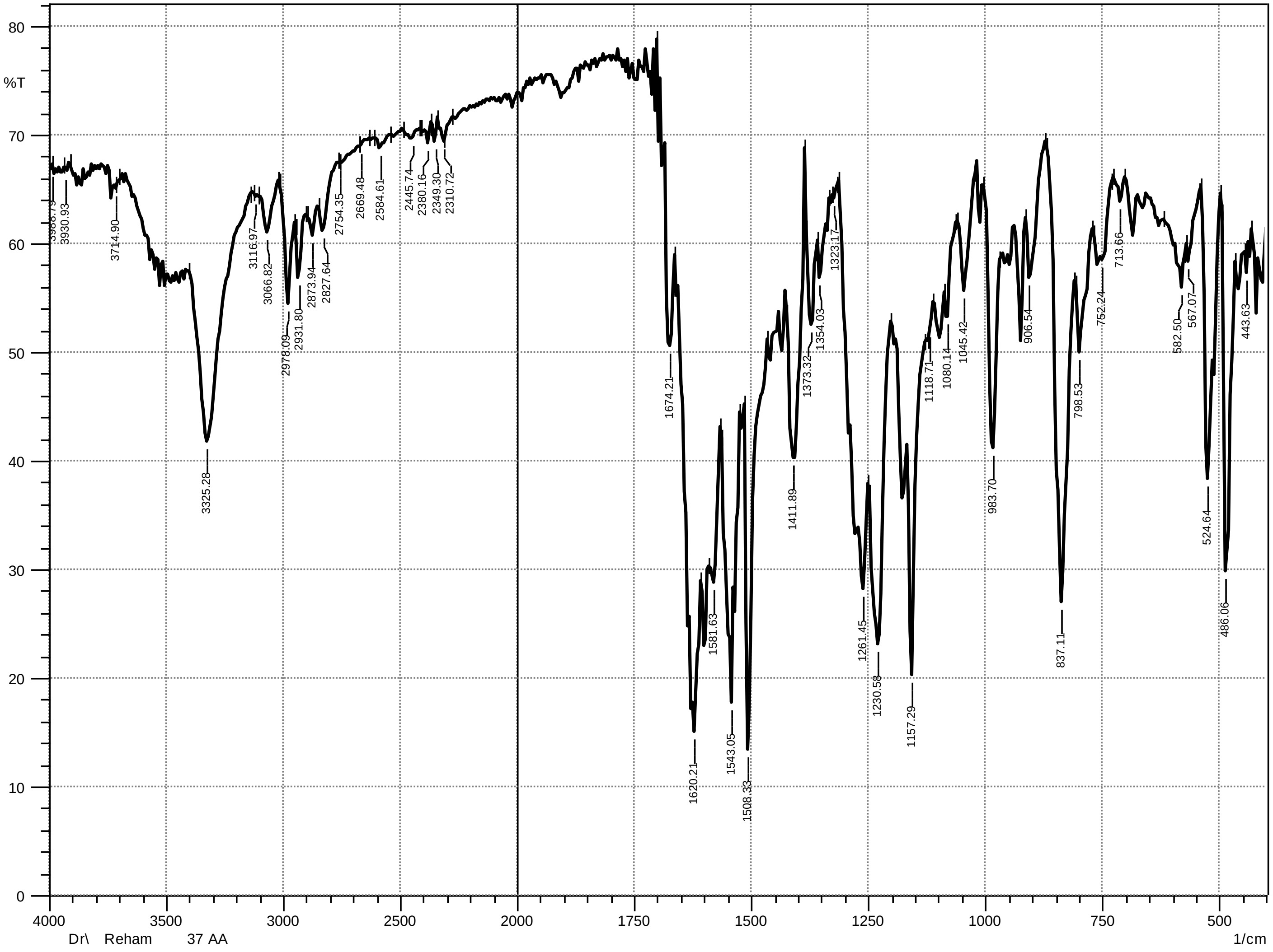

Fig. S40. IR spectrum of compound **37** (KBr pellet).

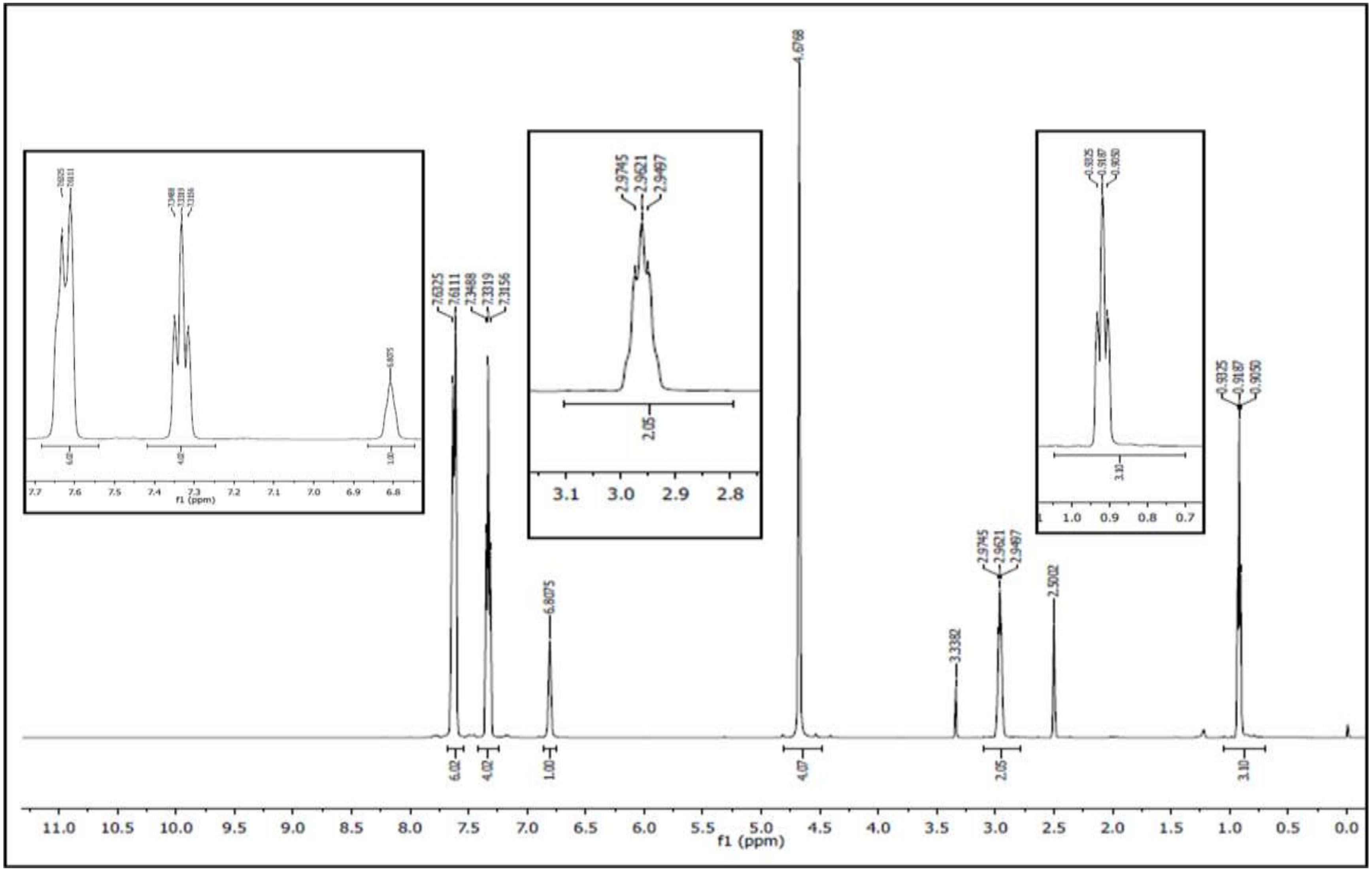

Fig. S41. ^{1}H-NMR spectrum of compound **37** in DMSO-d_6.

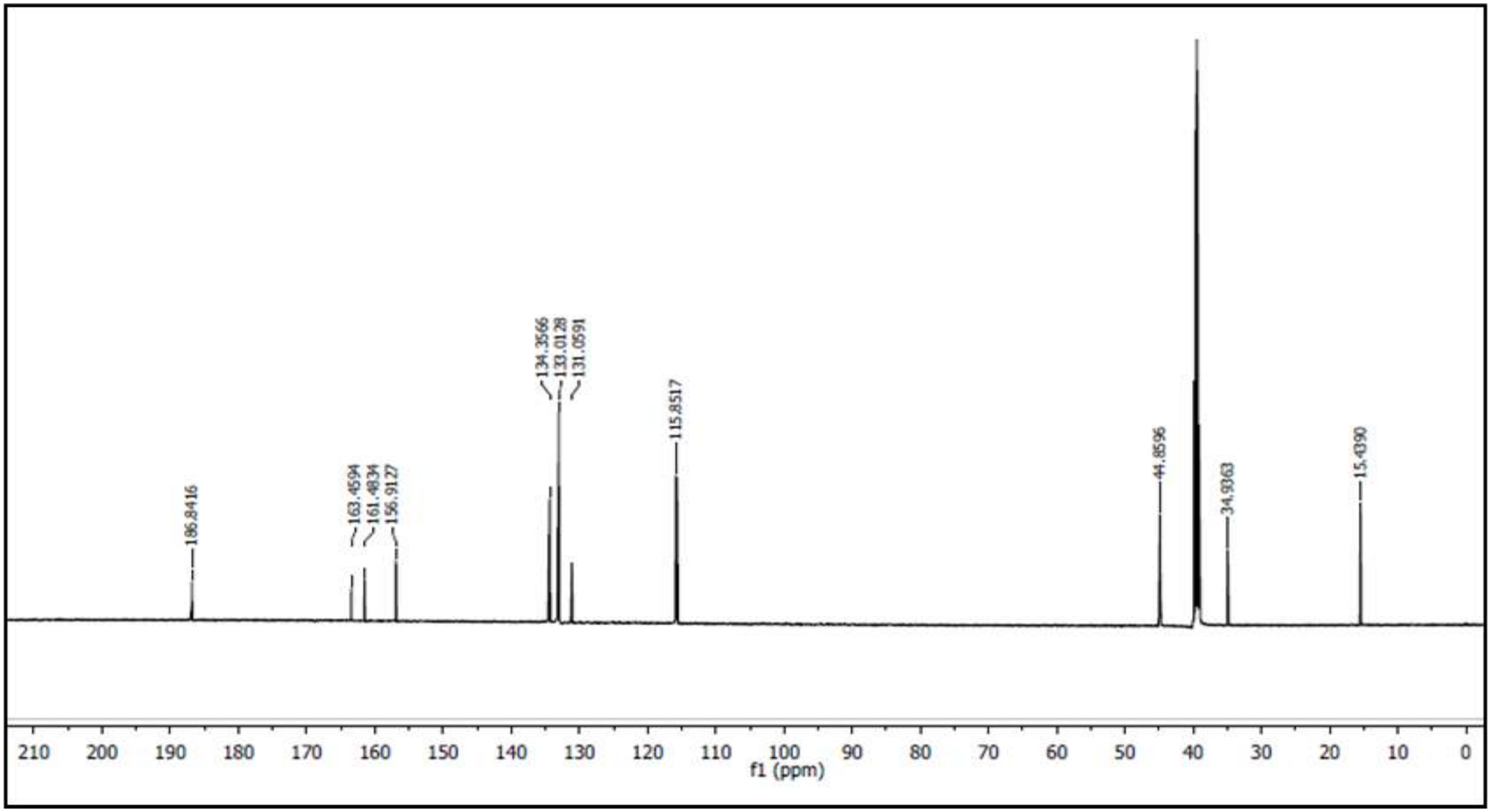

Fig. S42. ^{13}C-NMR spectrum of compound **37** in DMSO-d_6.

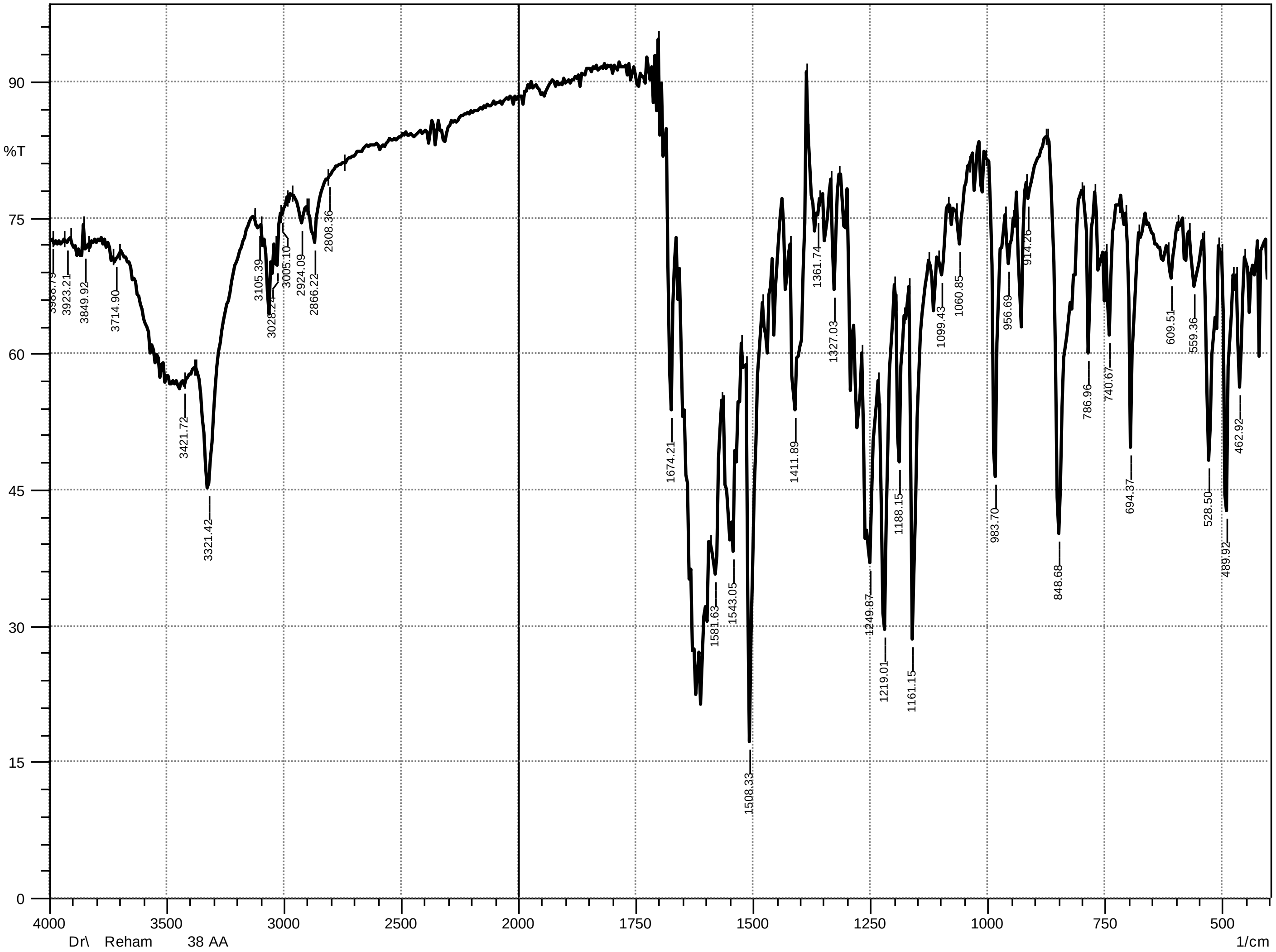

Fig. S43. IR spectrum of compound **38** (KBr pellet).

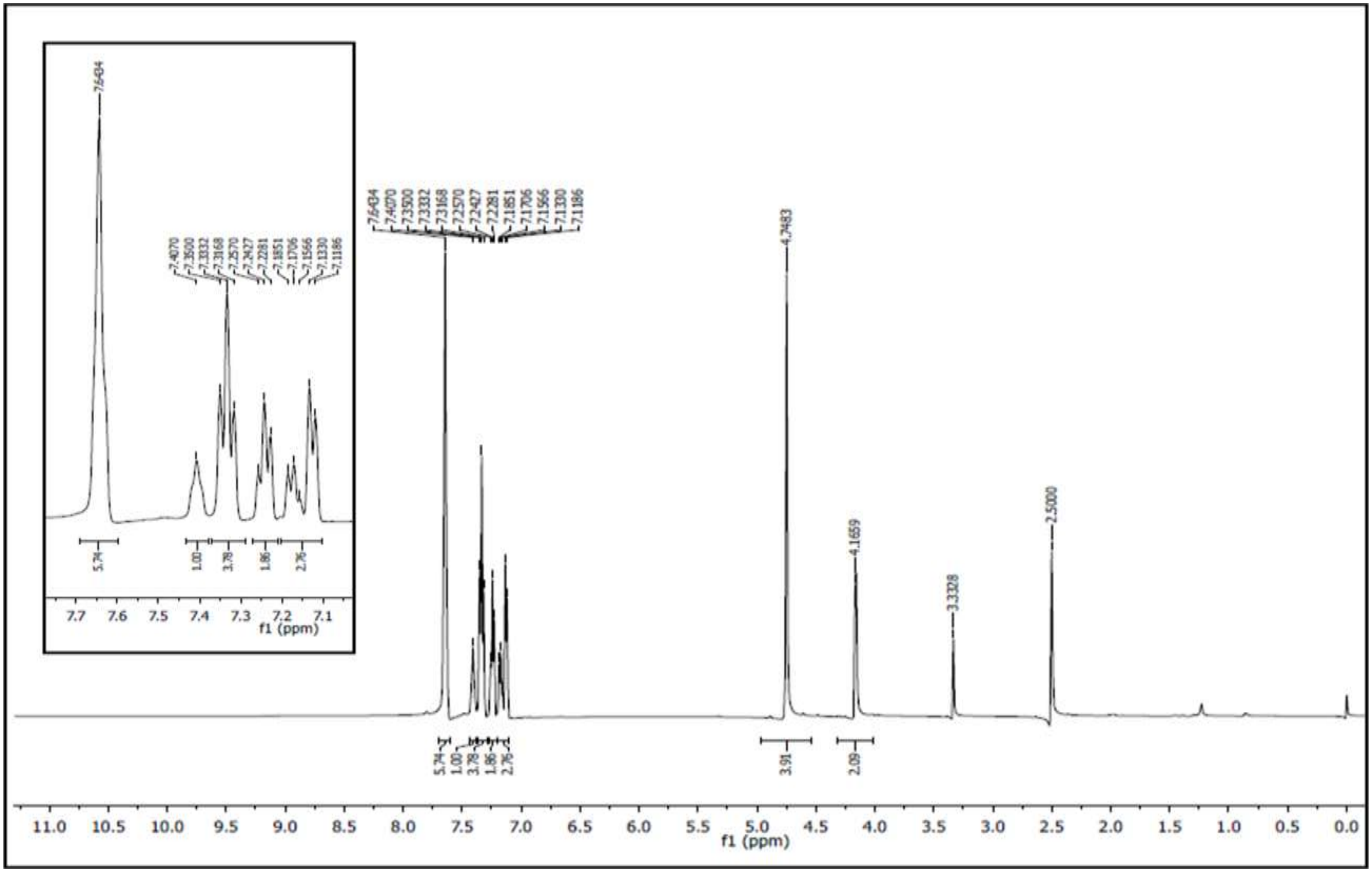

Fig. S44. ^{1}H-NMR spectrum of compound **38** in DMSO-d_6.

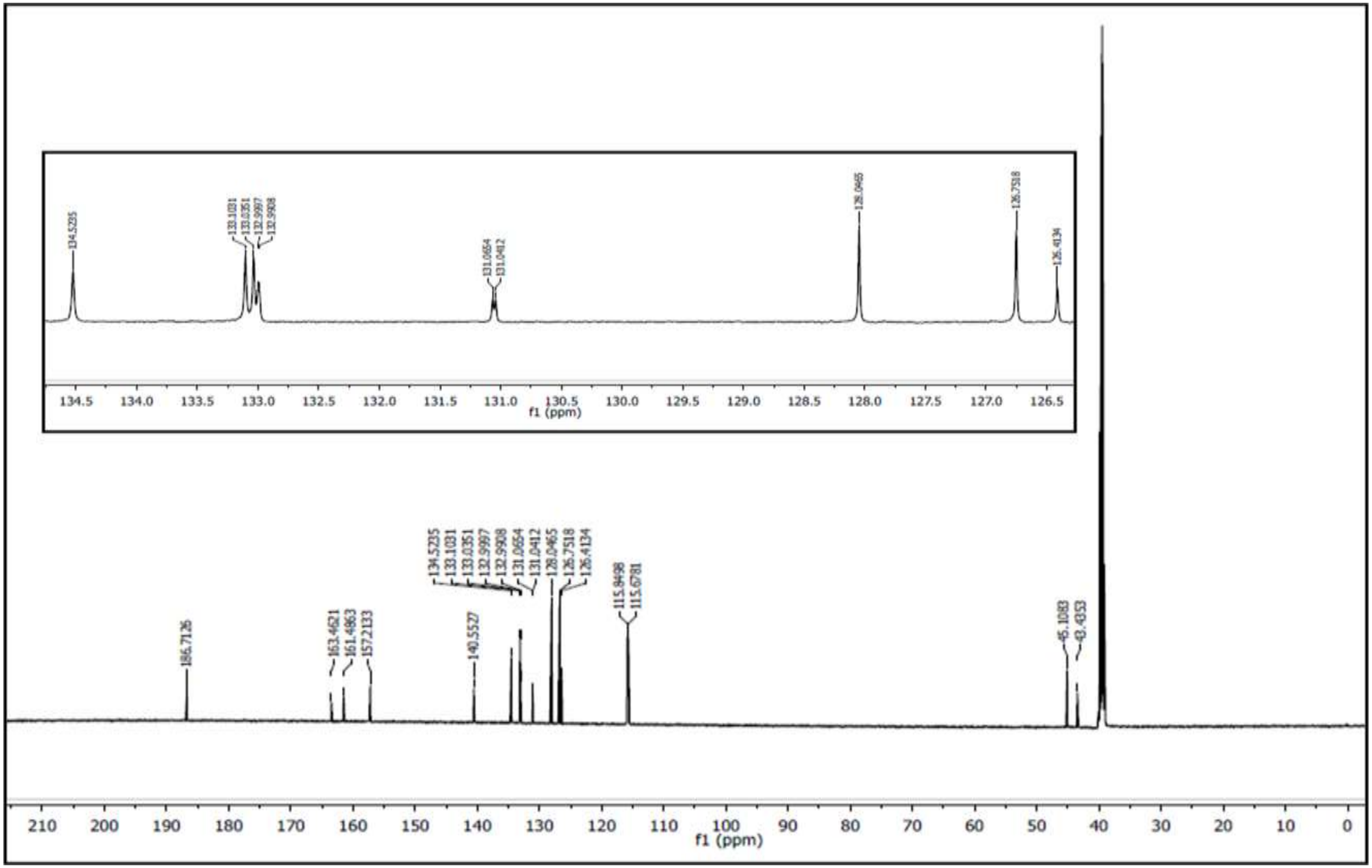

Fig. S45. ^{13}C-NMR spectrum of compound **38** in DMSO-d_6.

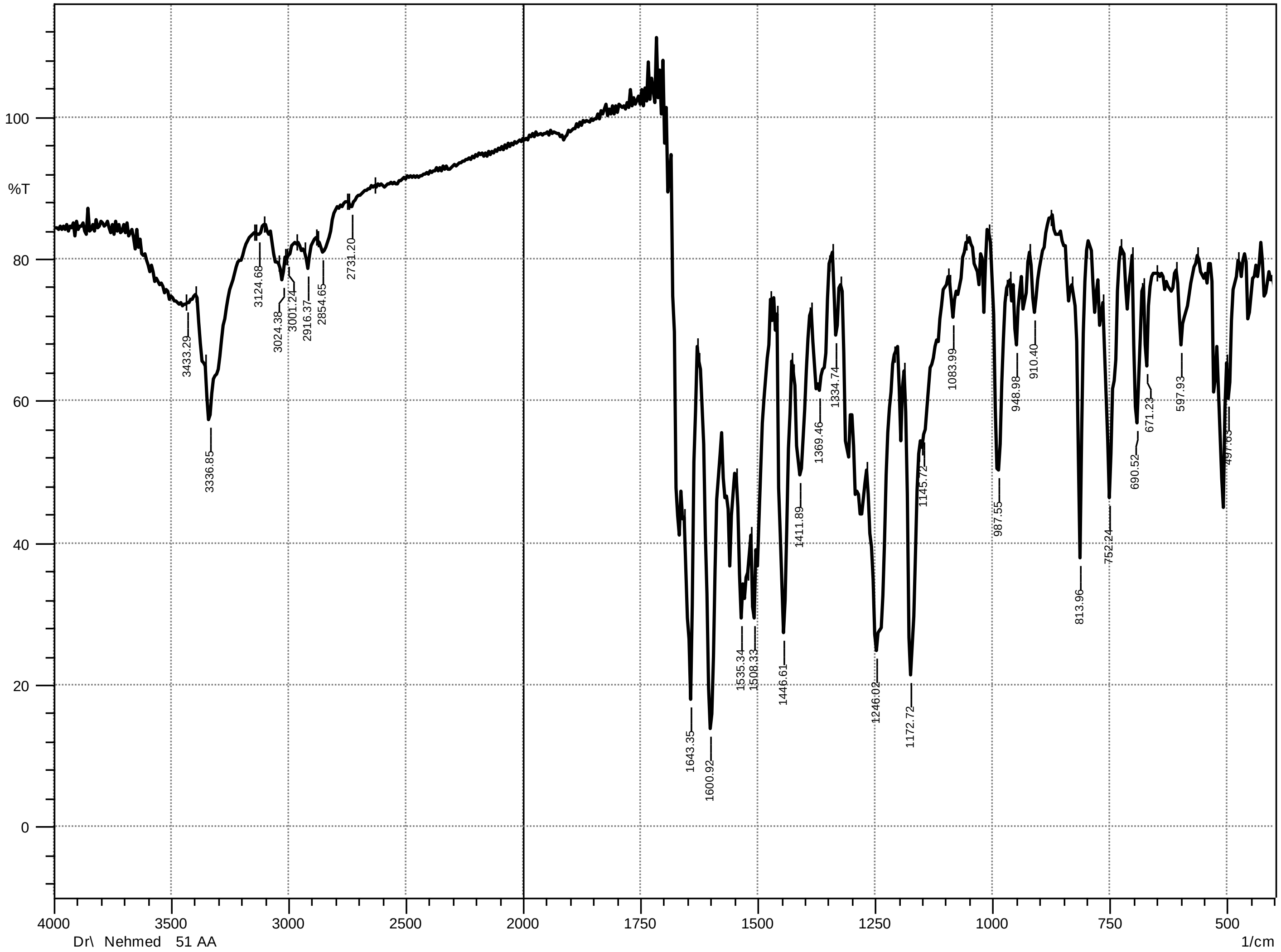

Fig. S46. IR spectrum of compound **39** (KBr pellet).

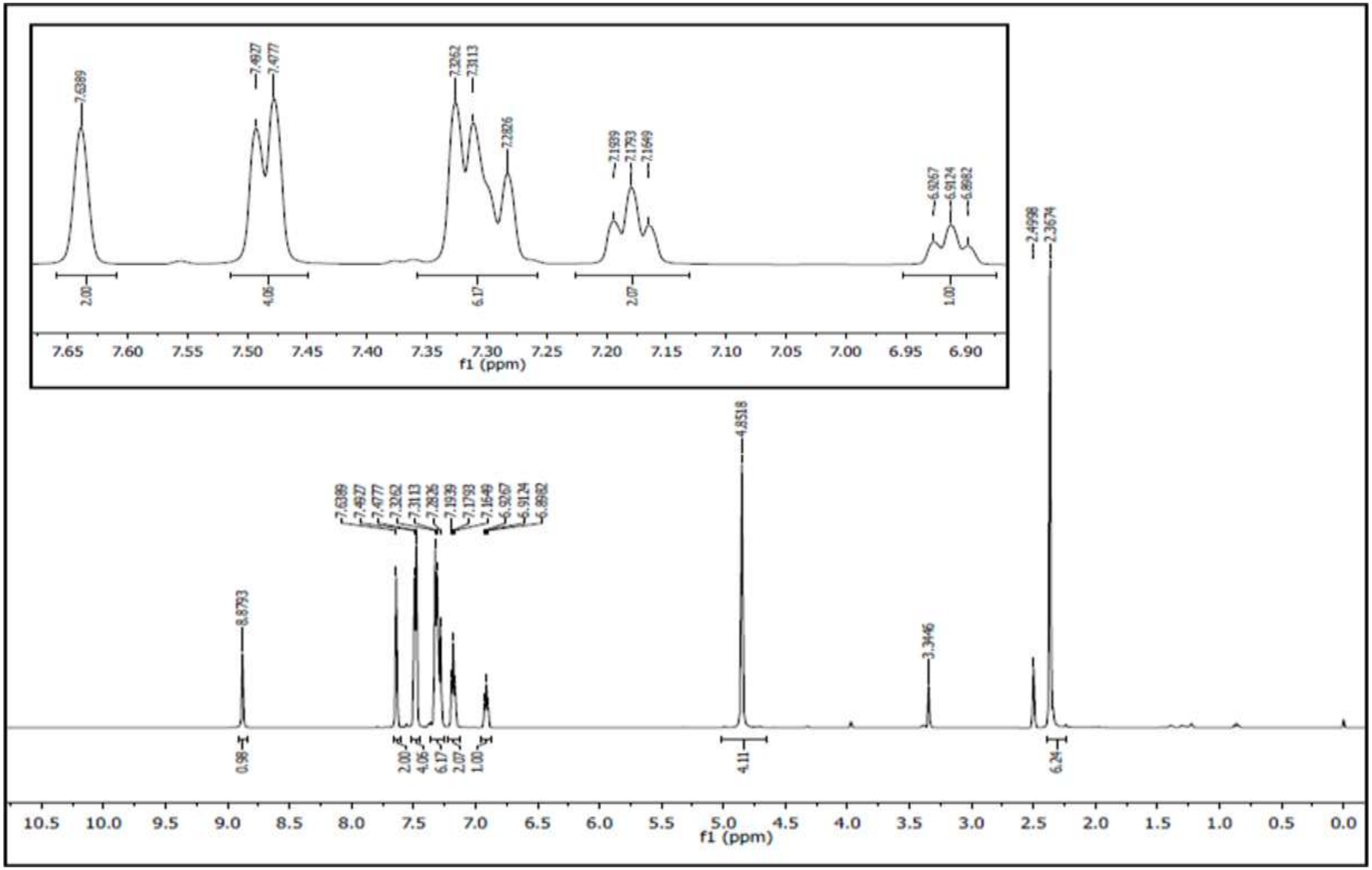

Fig. S47. ^{1}H-NMR spectrum of compound **39** in DMSO-d_6.

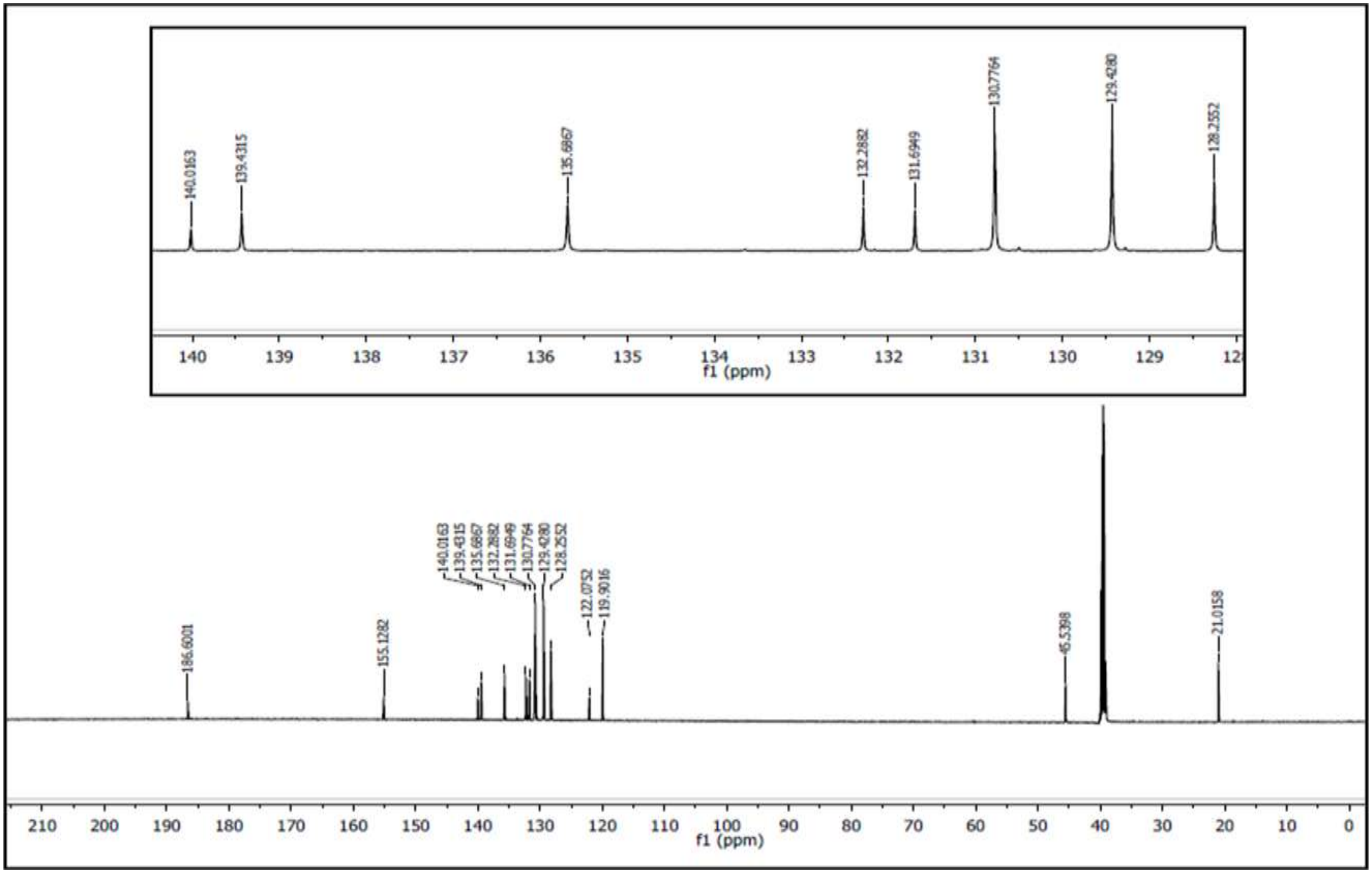

Fig. S48. ^{13}C-NMR spectrum of compound **39** in DMSO-d_6.

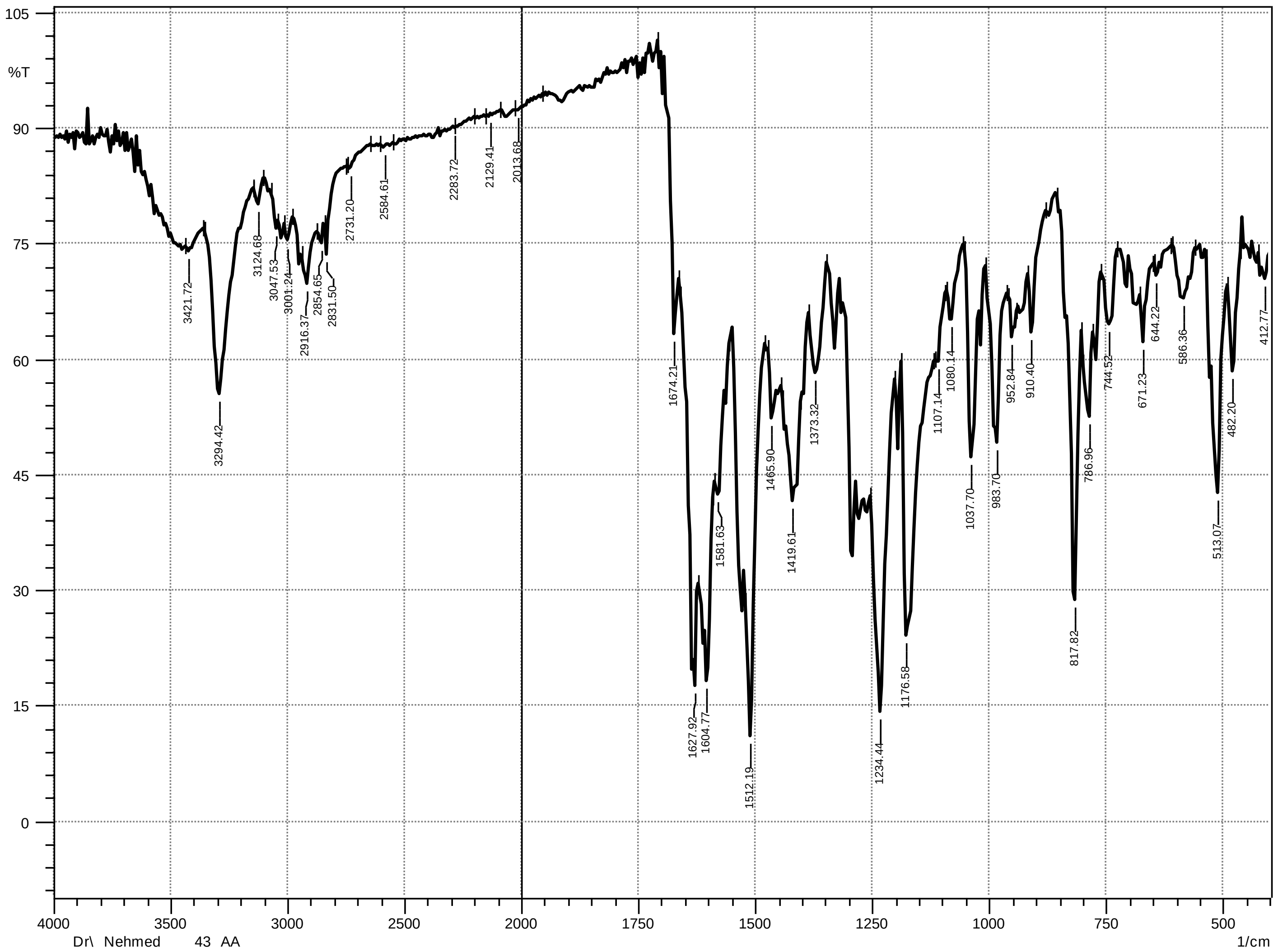

Fig. S49. IR spectrum of compound **40** (KBr pellet).

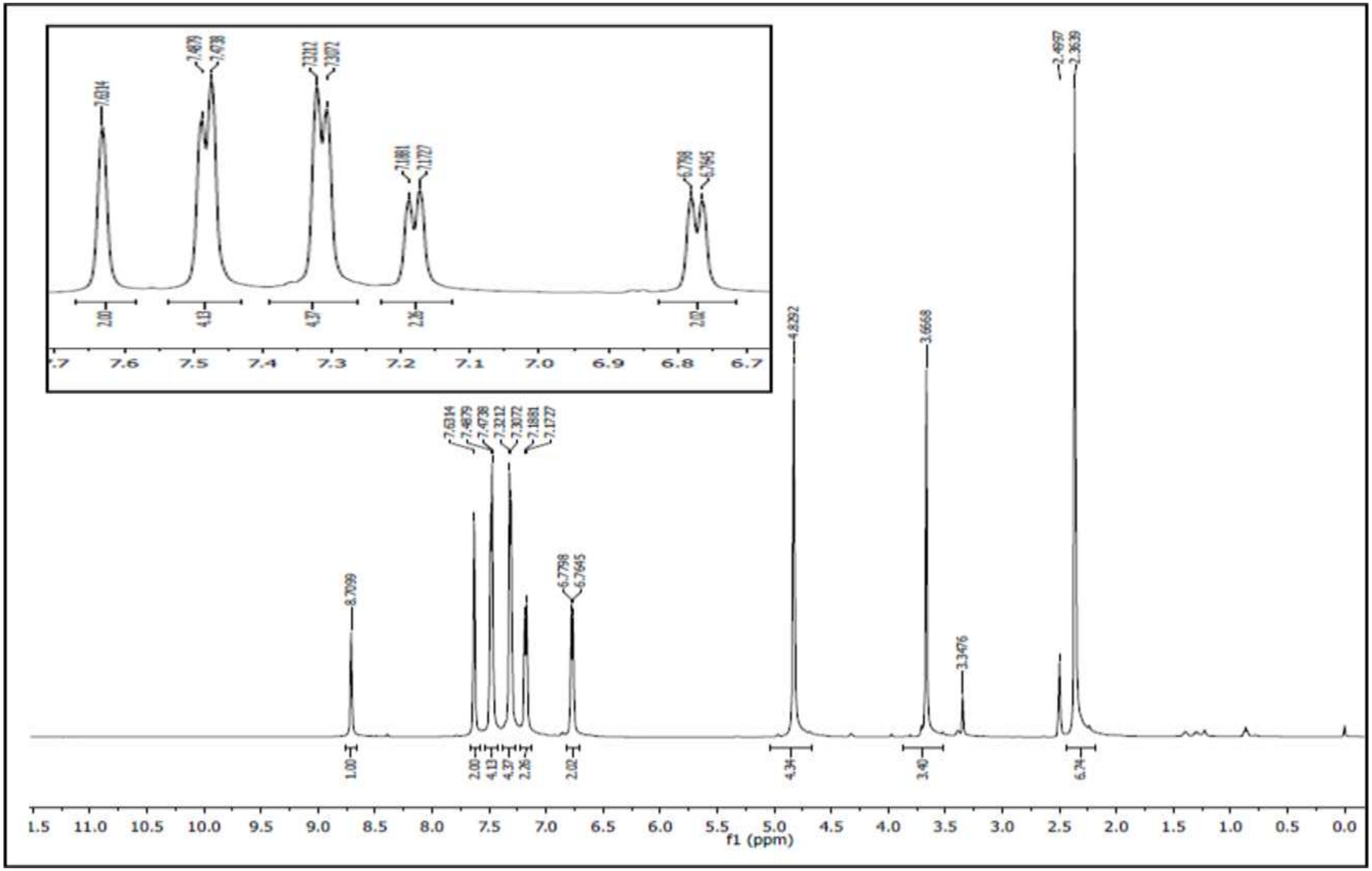

Fig. S50. ^{1}H-NMR spectrum of compound **40** in DMSO-d_6.

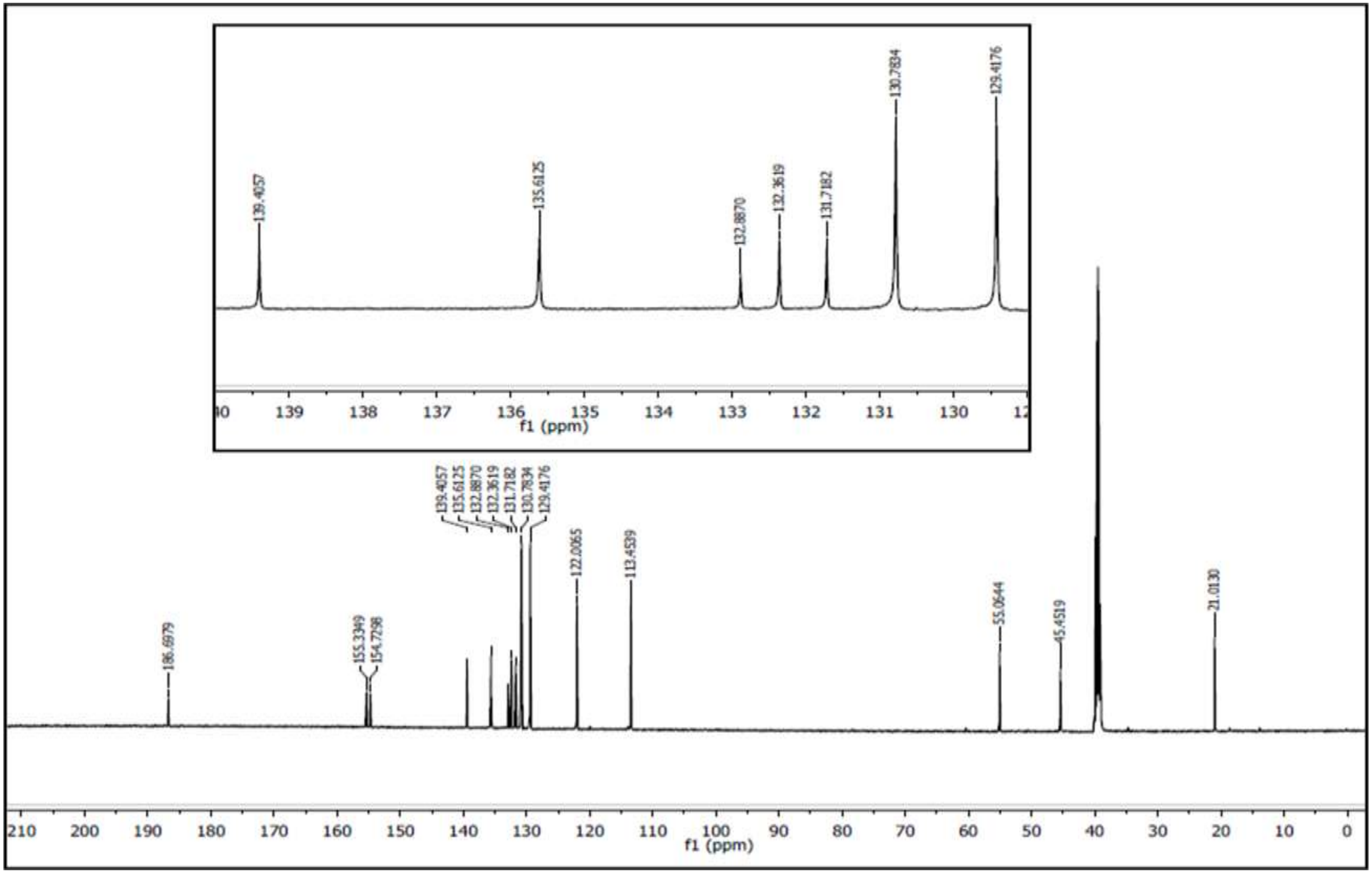

Fig. S51. ^{13}C-NMR spectrum of compound **40** in DMSO-d_6.

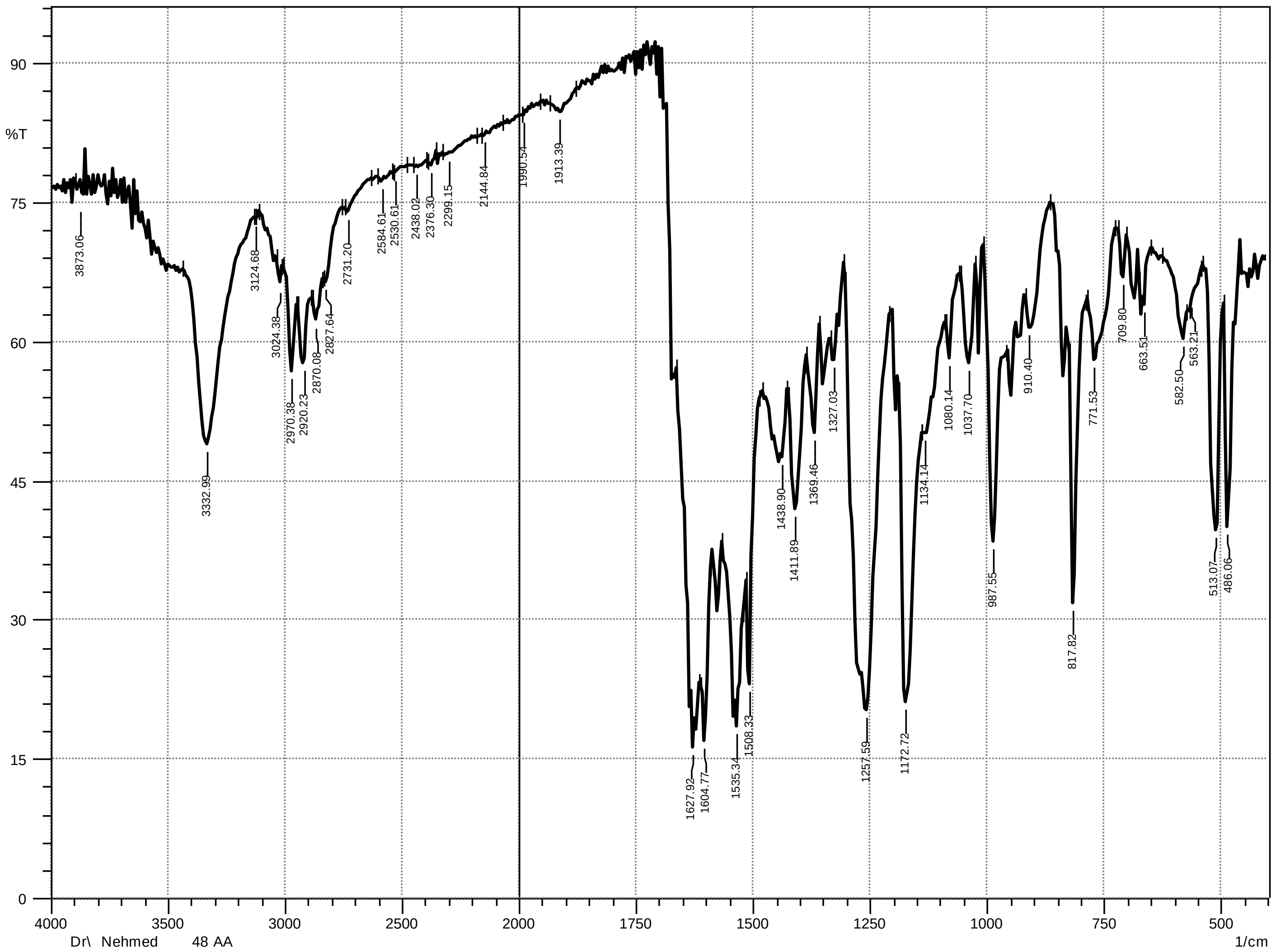

Fig. S52. IR spectrum of compound **41** (KBr pellet).

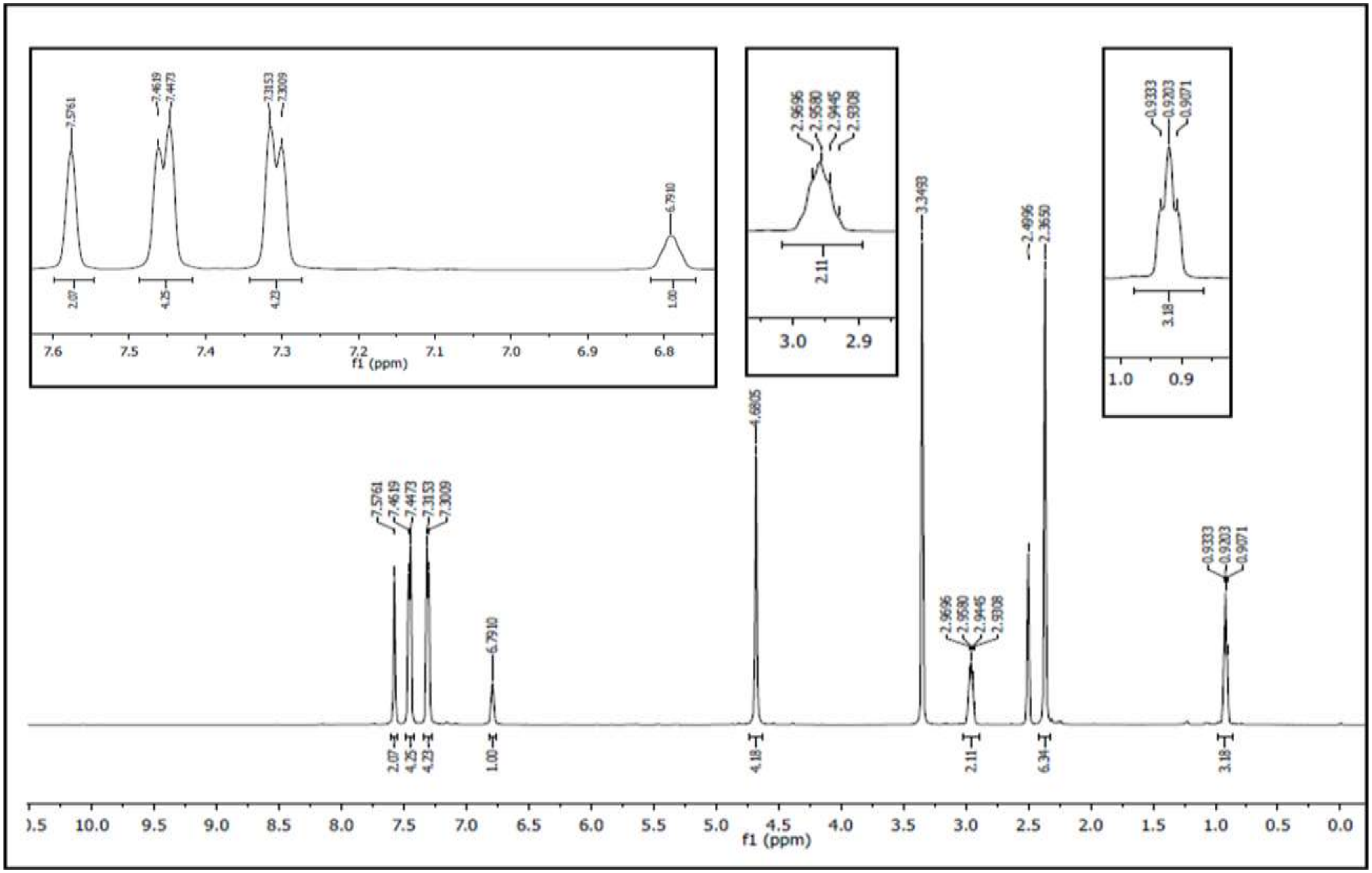

Fig. S53. ^{1}H-NMR spectrum of compound **41** in DMSO-d_6.

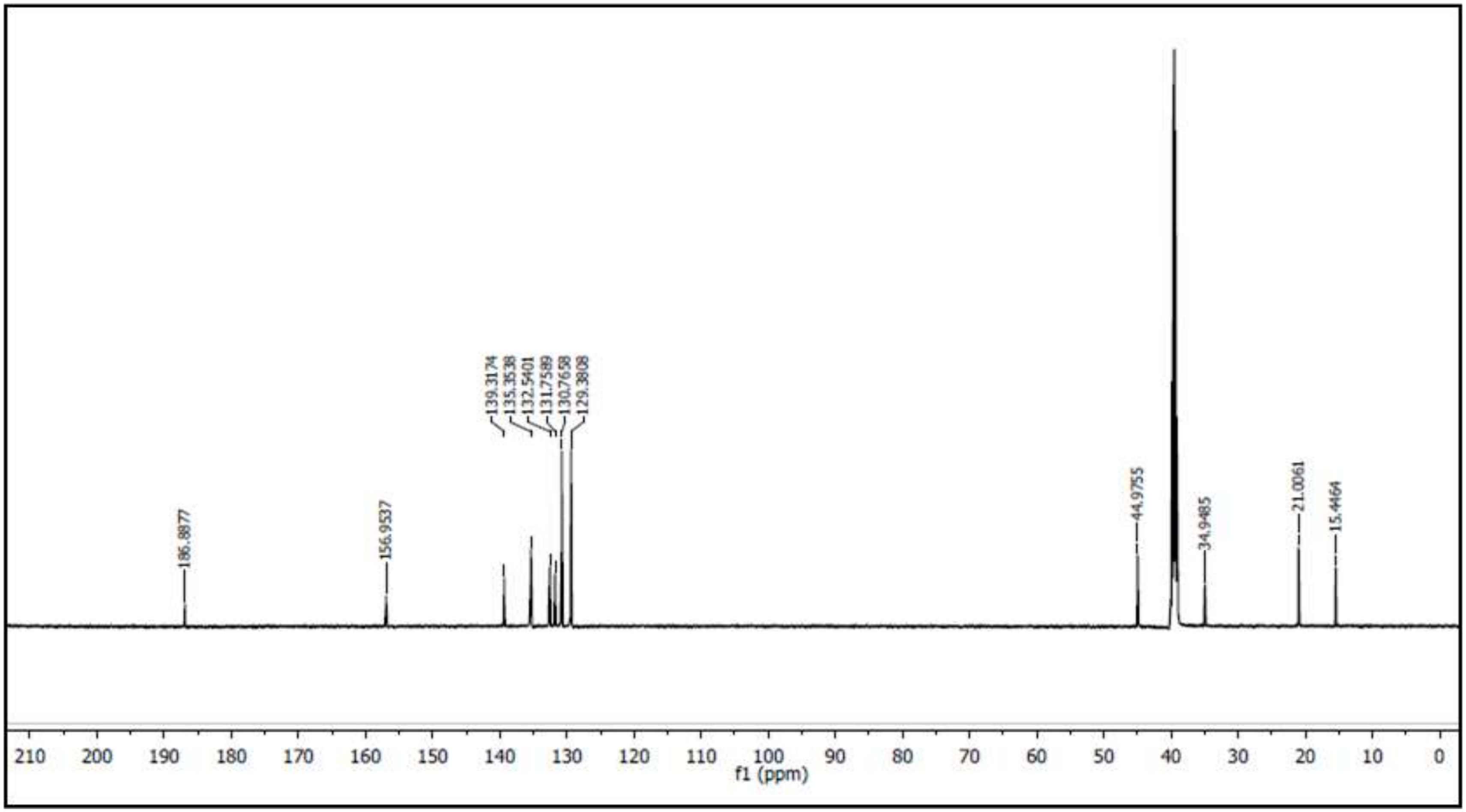

Fig. S54. ^{13}C-NMR spectrum of compound **41** in DMSO-d_6.

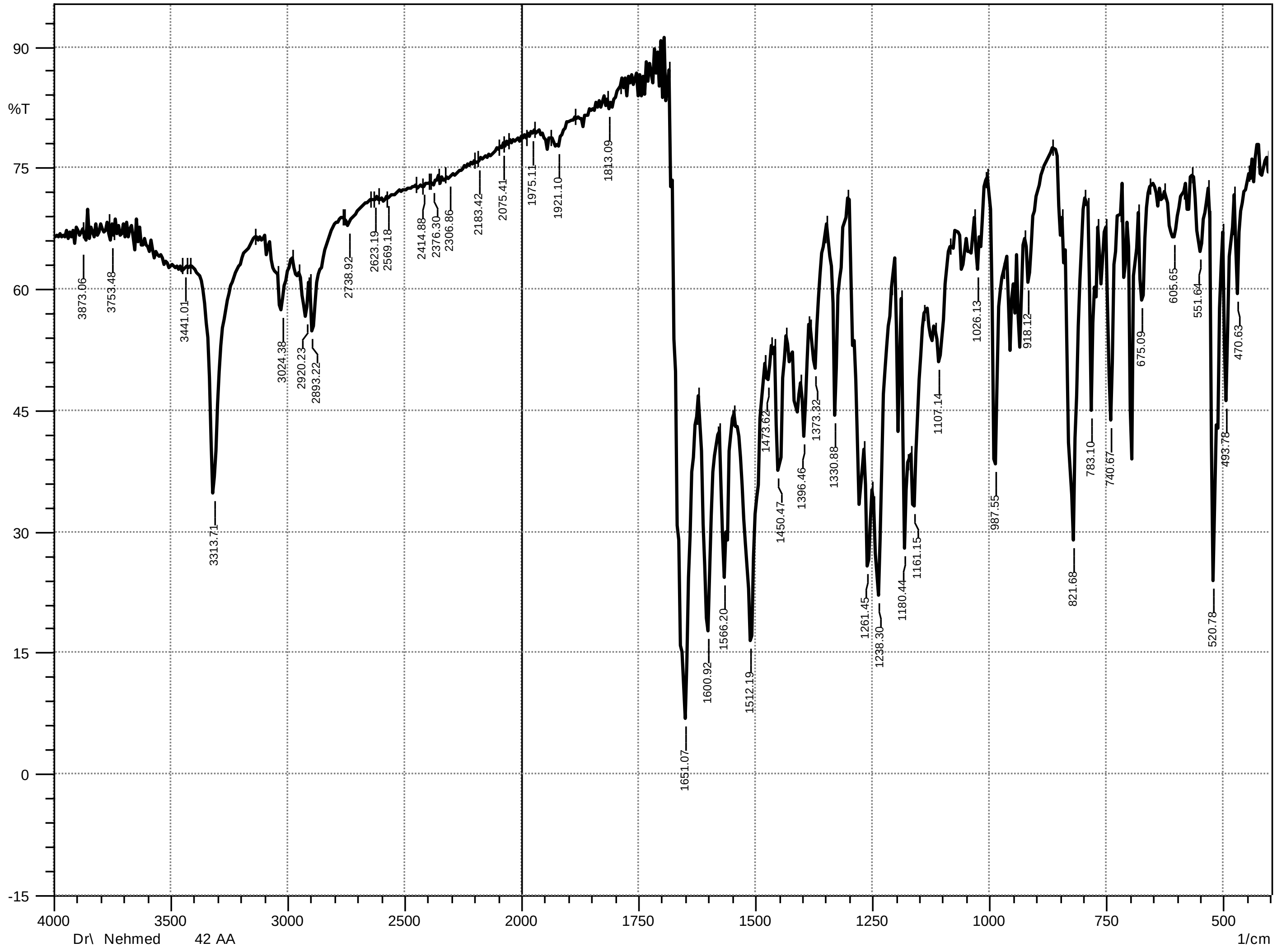

Fig. S55. IR spectrum of compound **42** (KBr pellet).

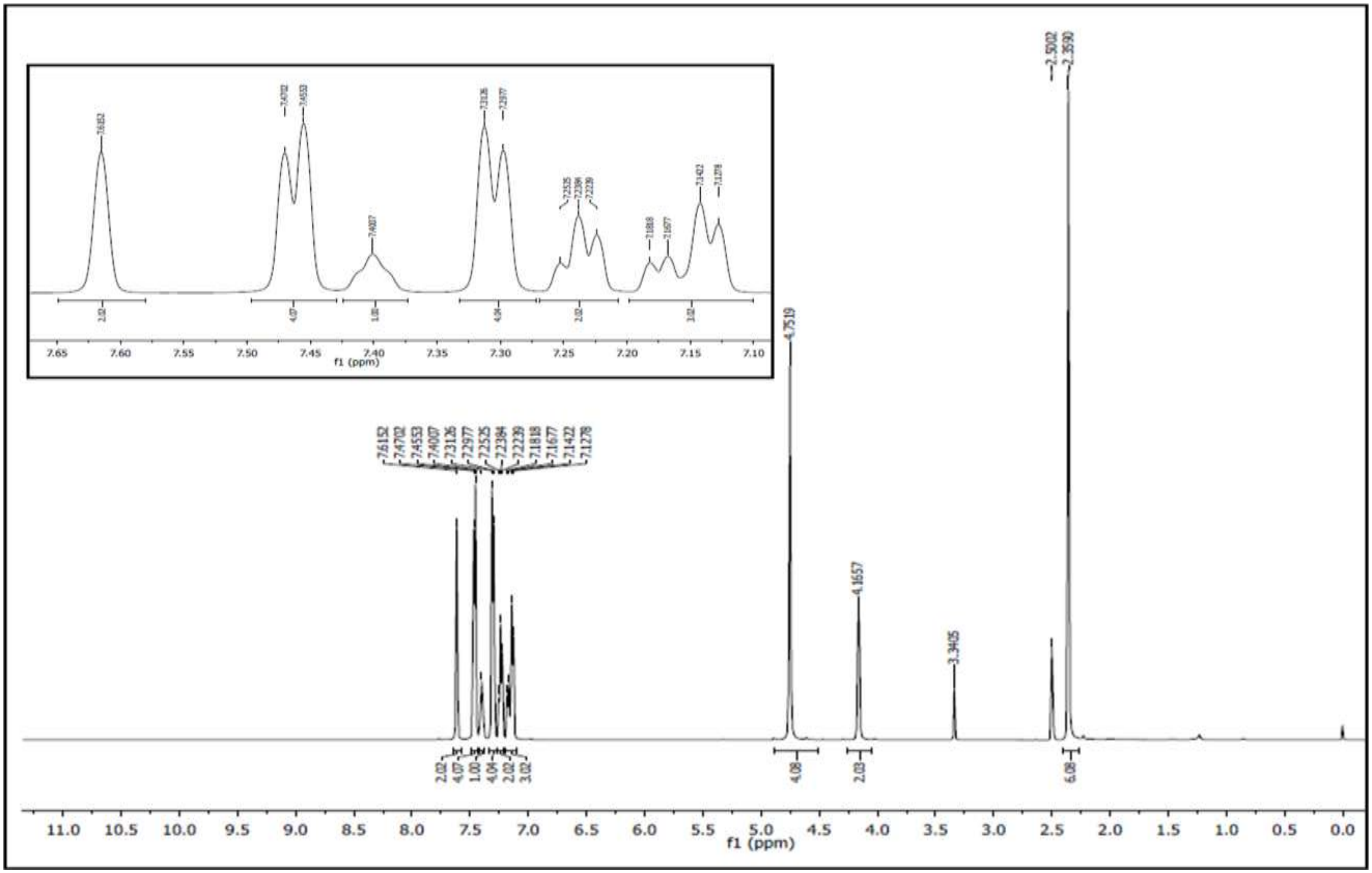

Fig. S56. ^{1}H-NMR spectrum of compound **42** in DMSO-d_6.

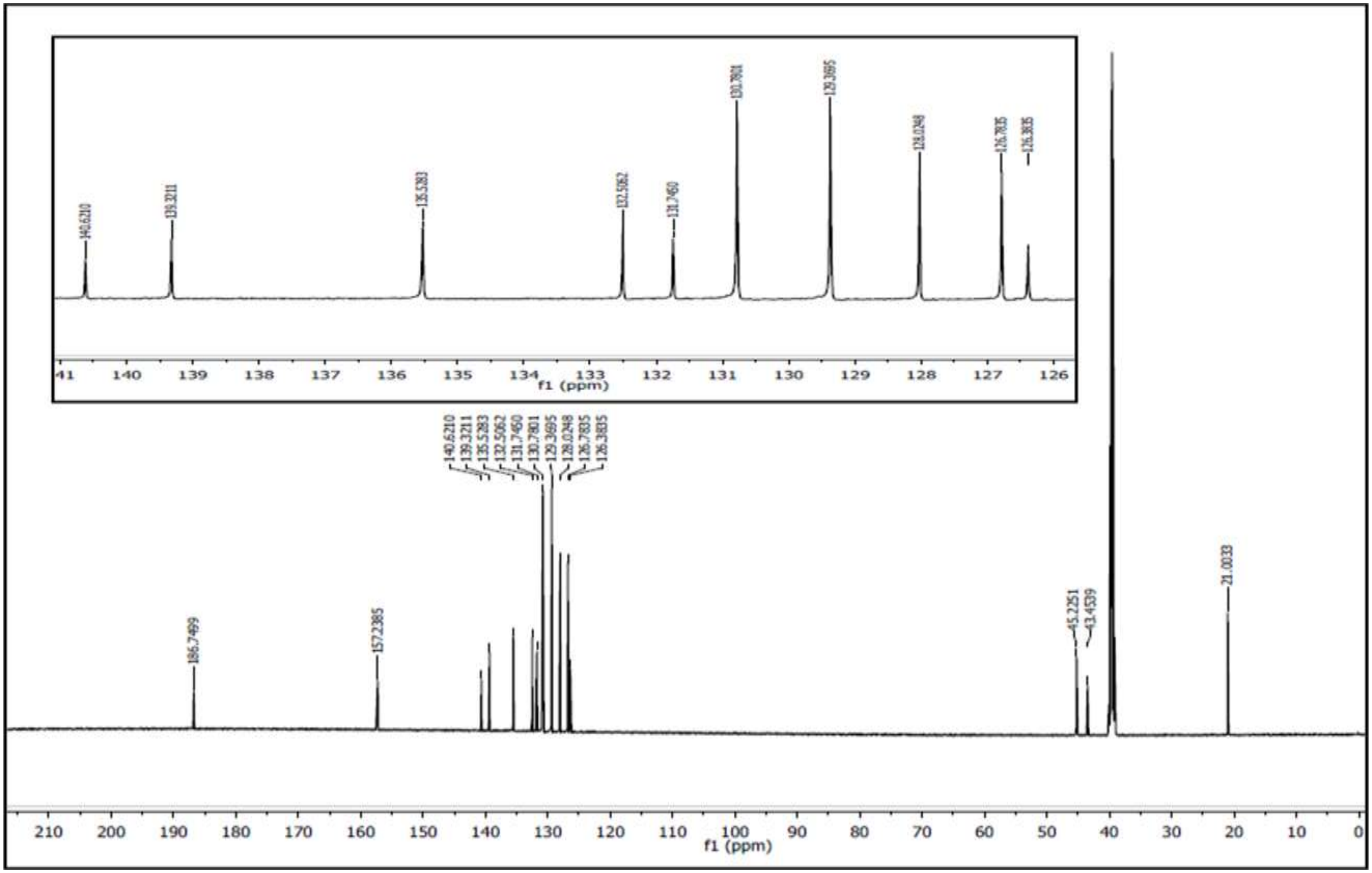

Fig. S57. ^{13}C-NMR spectrum of compound **42** in DMSO-d_6.

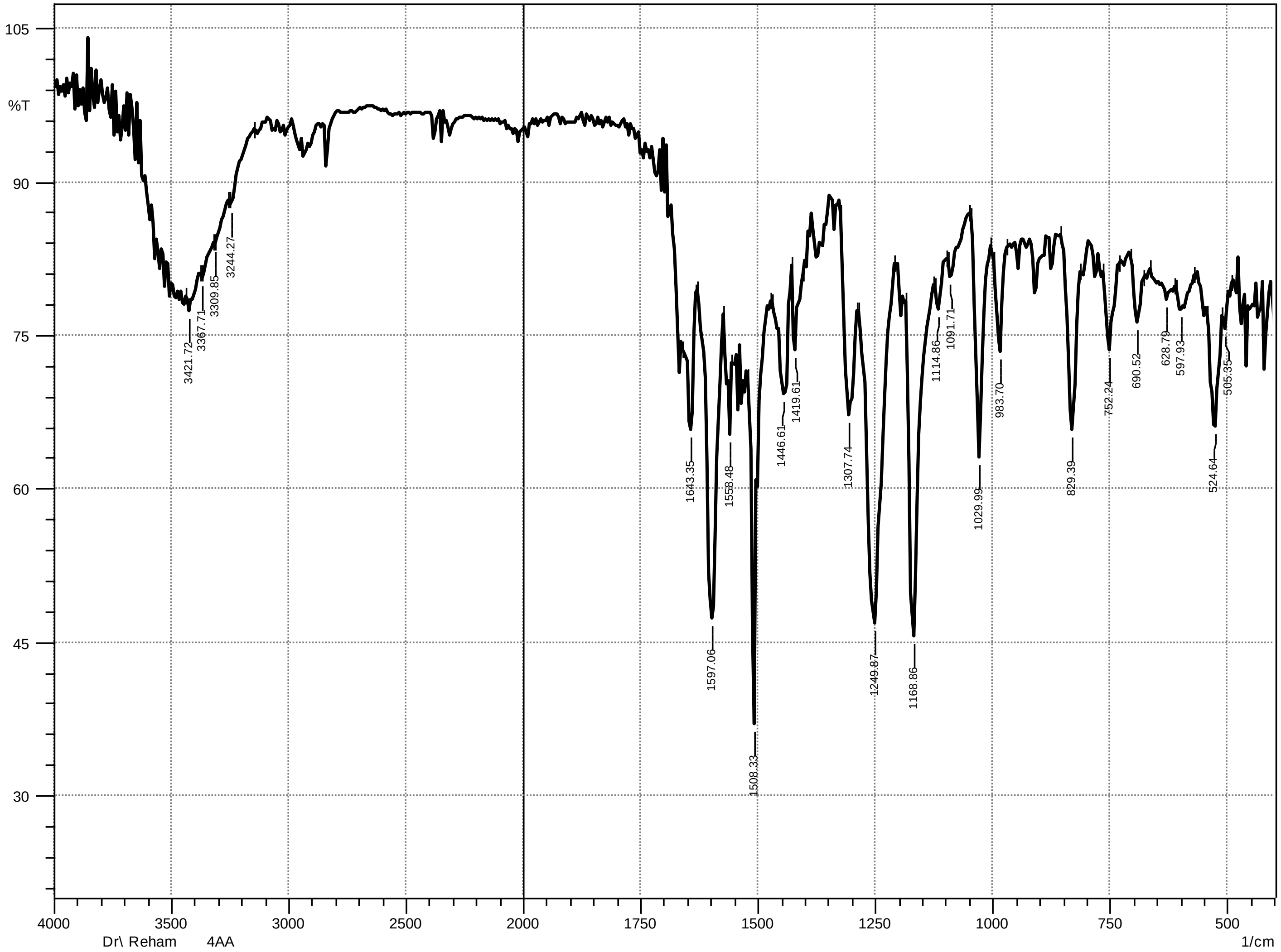

Fig. S58. IR spectrum of compound **43** (KBr pellet).

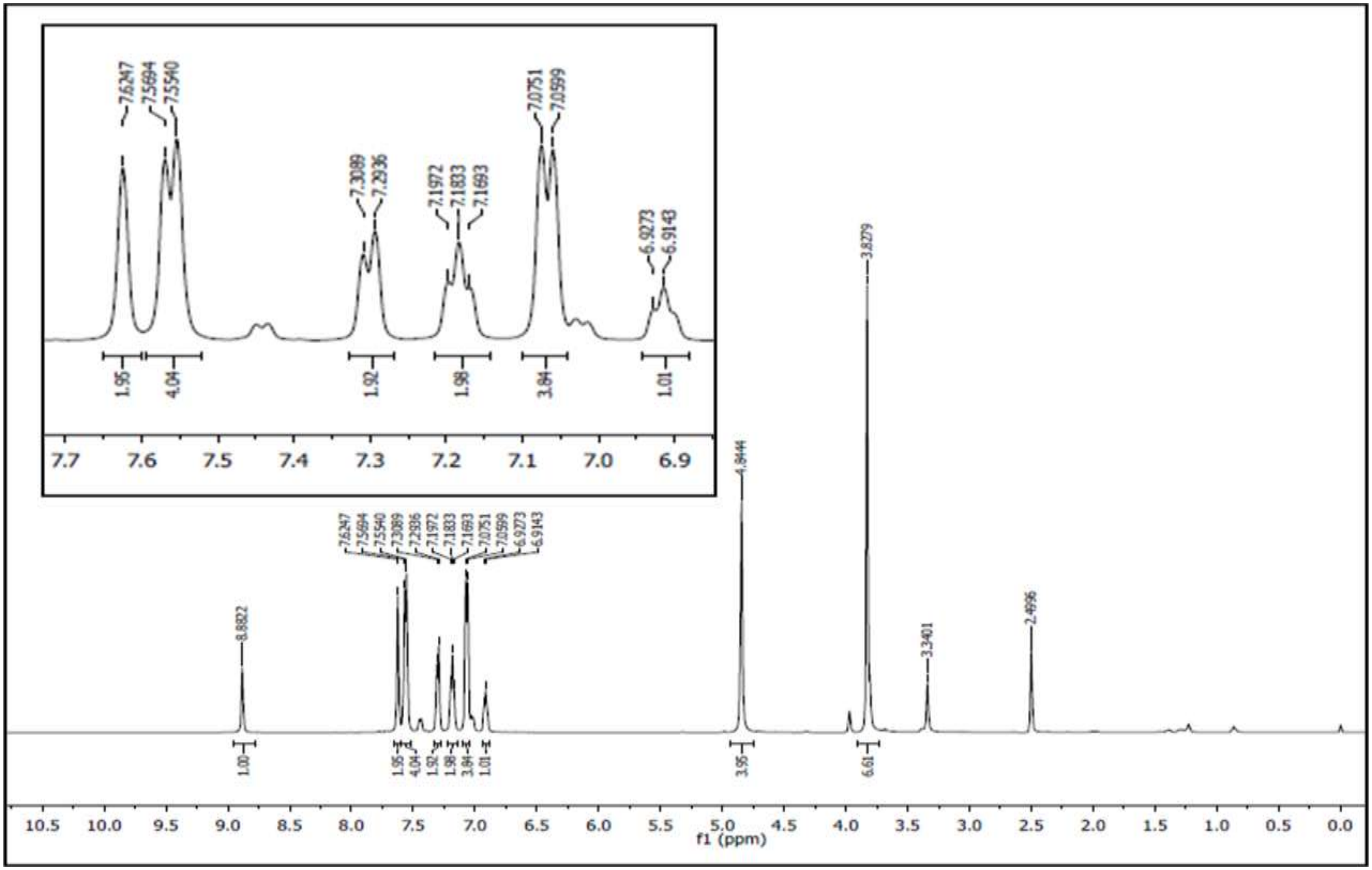

Fig. S59. ^{1}H-NMR spectrum of compound **43** in DMSO-d_6.

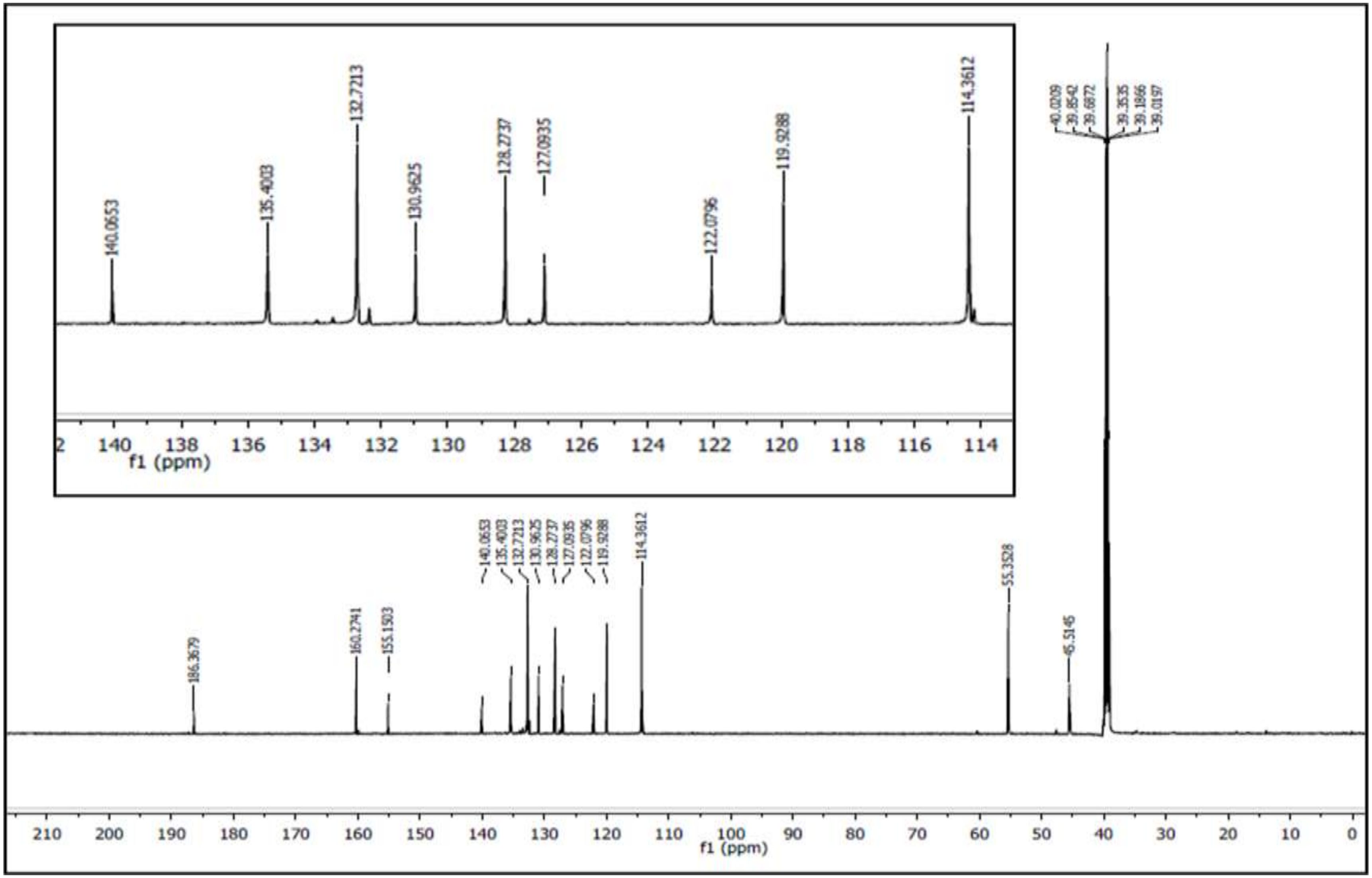

Fig. S60. ^{13}C-NMR spectrum of compound **43** in DMSO-d_6.

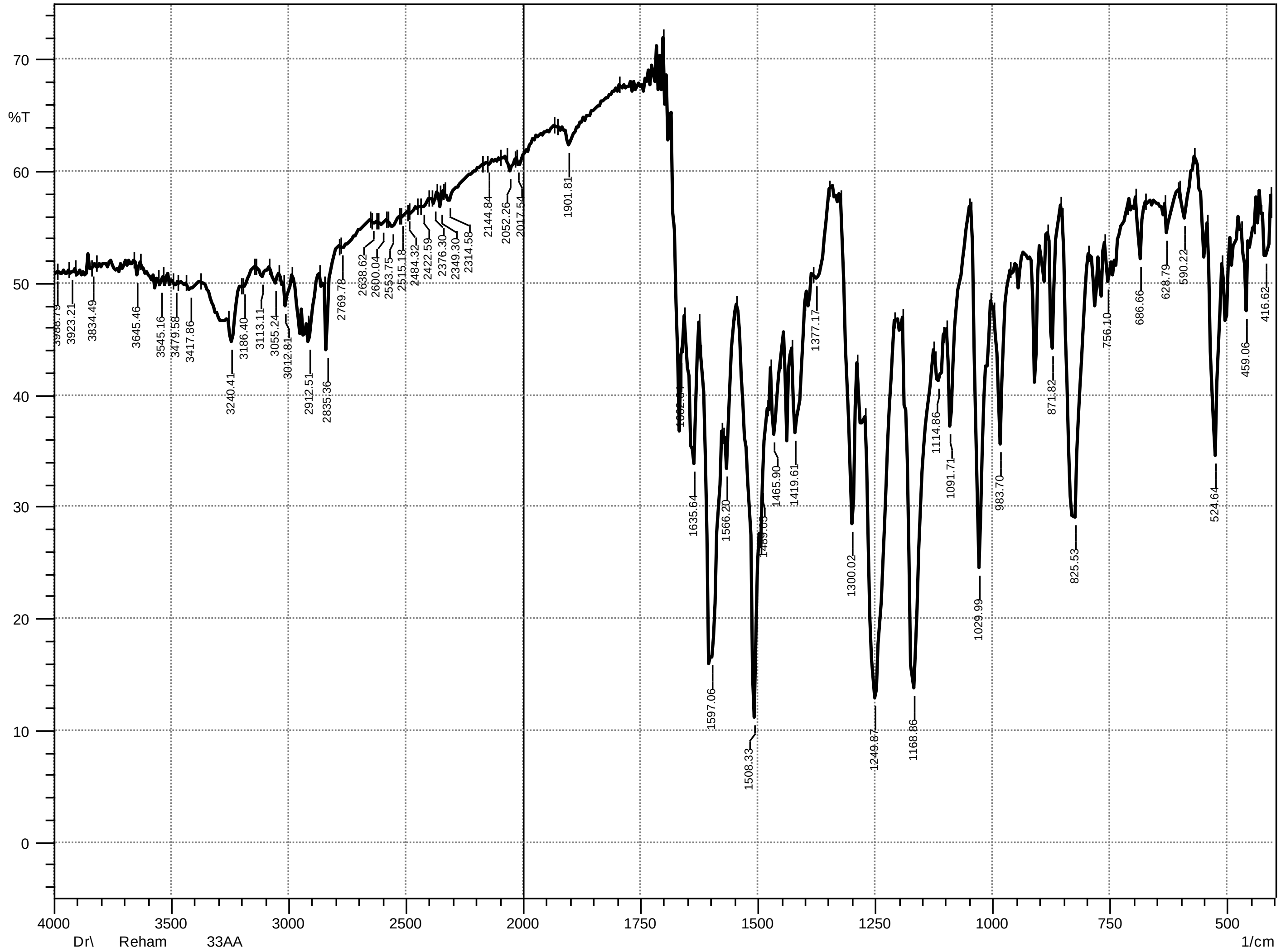

Fig. S61. IR spectrum of compound **44** (KBr pellet).

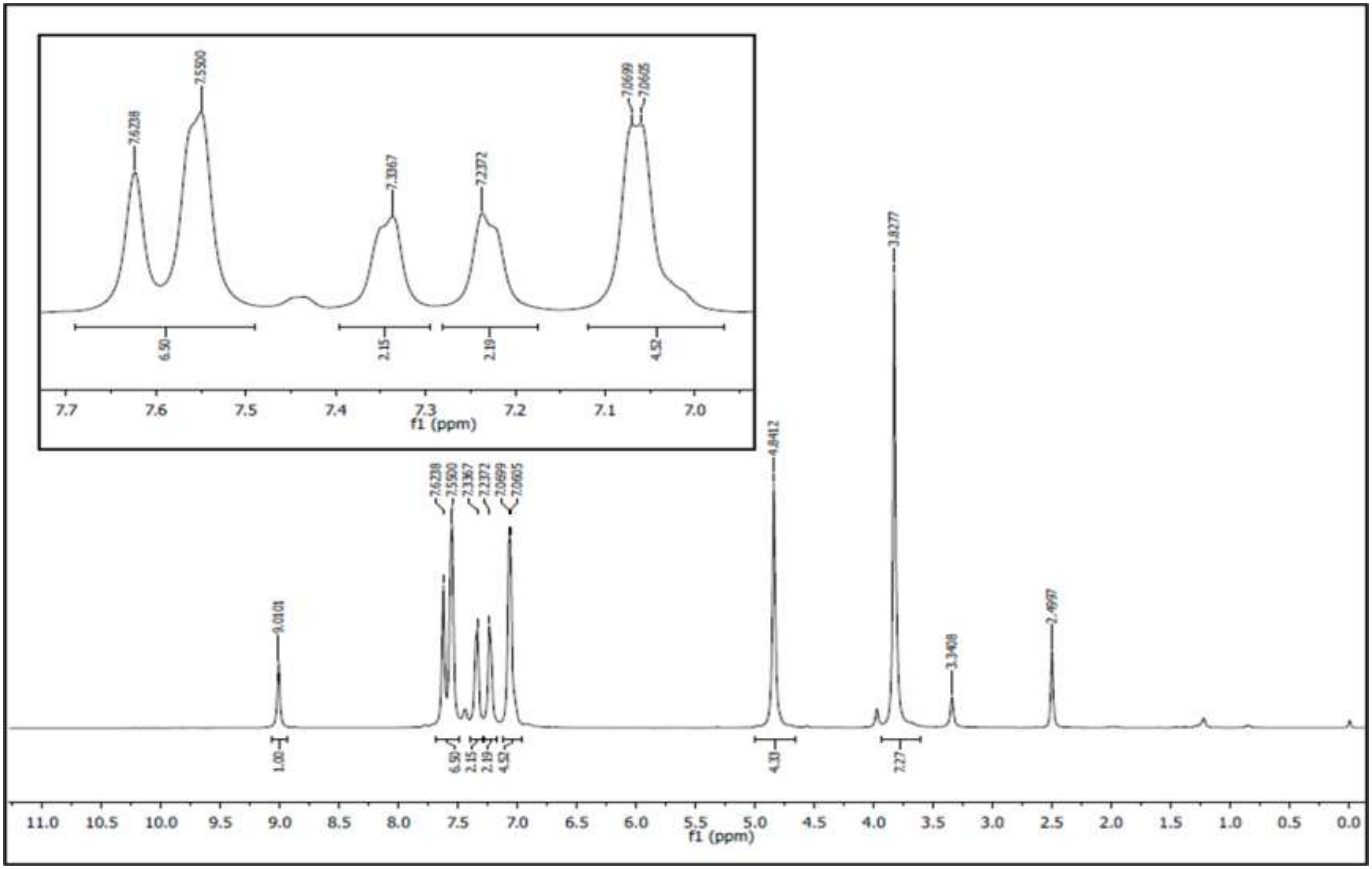

Fig. S62. ^{1}H-NMR spectrum of compound **44** in DMSO-d_6.

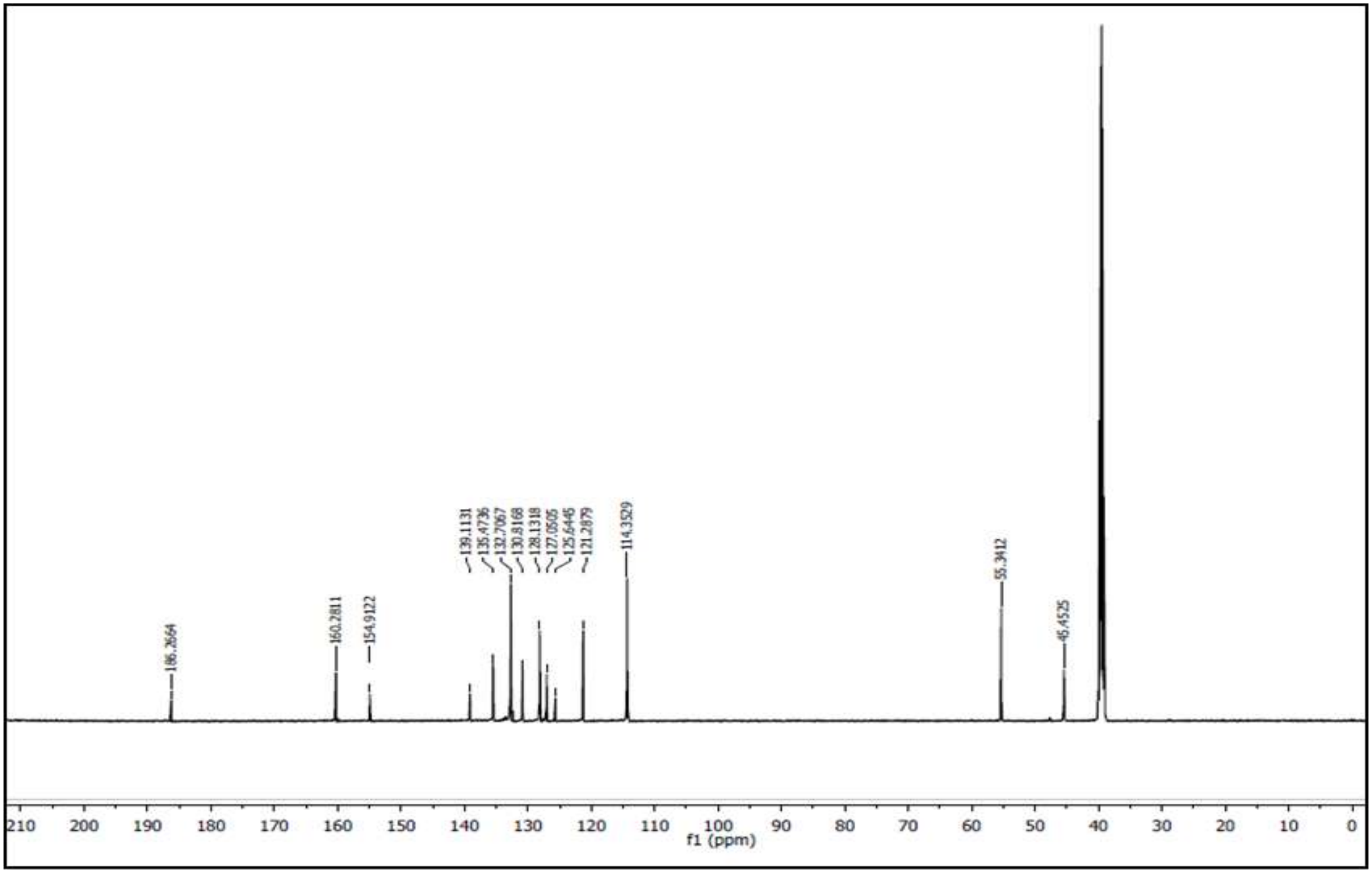

Fig. S63. ^{13}C-NMR spectrum of compound **44** in DMSO-d_6.

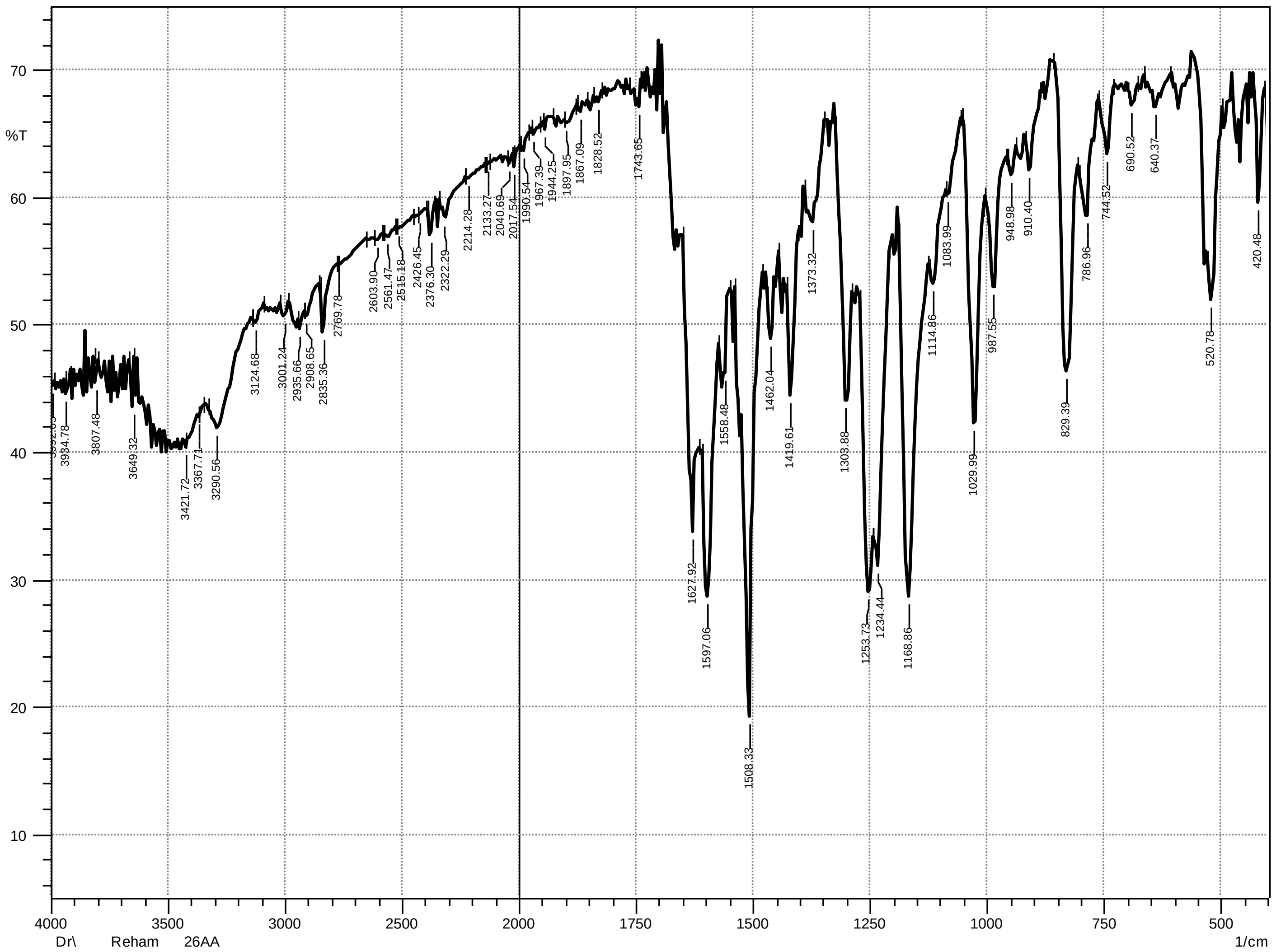

Fig. S64. IR spectrum of compound **45** (KBr pellet).

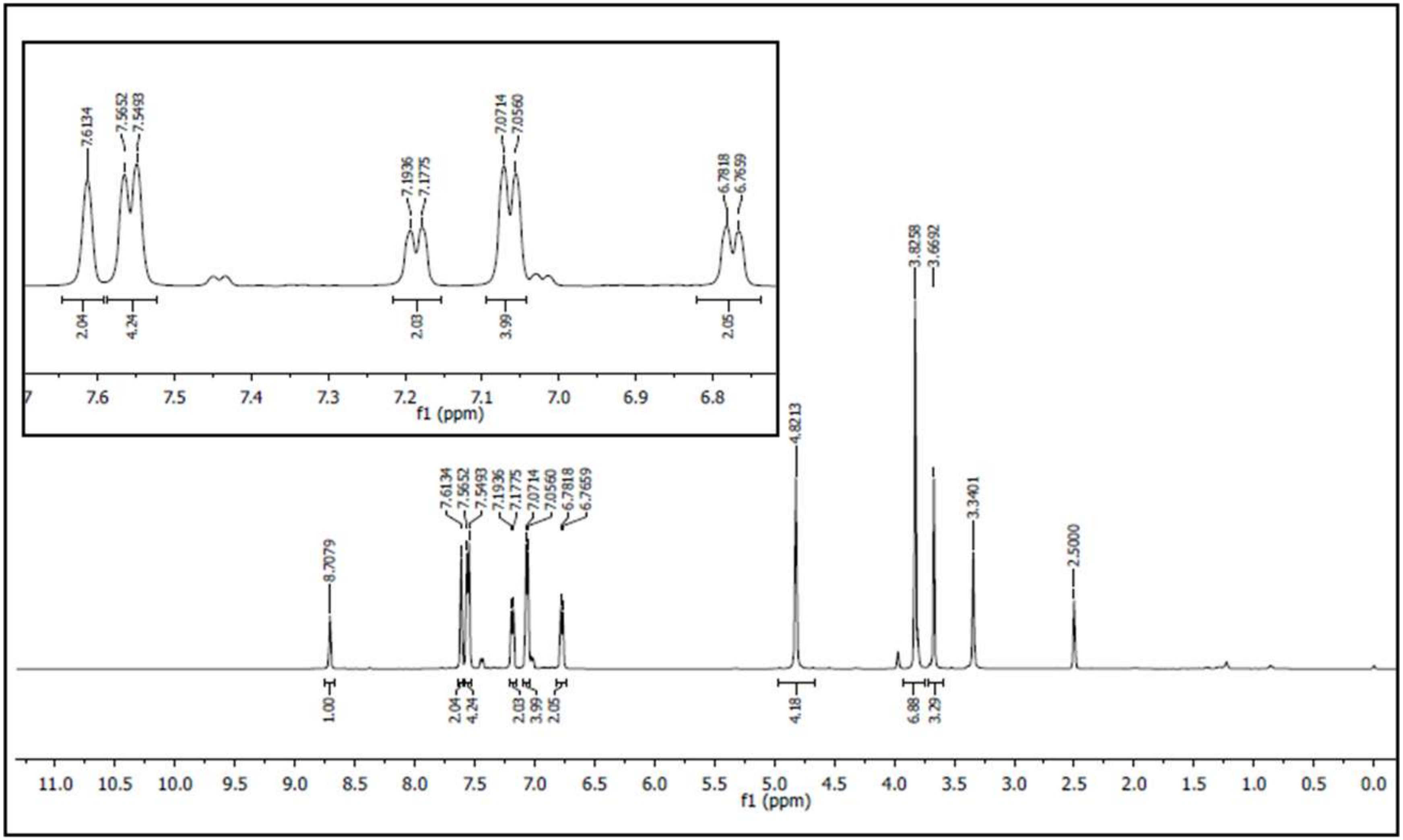

Fig. S65. ^{1}H-NMR spectrum of compound **45** in DMSO-d_6.

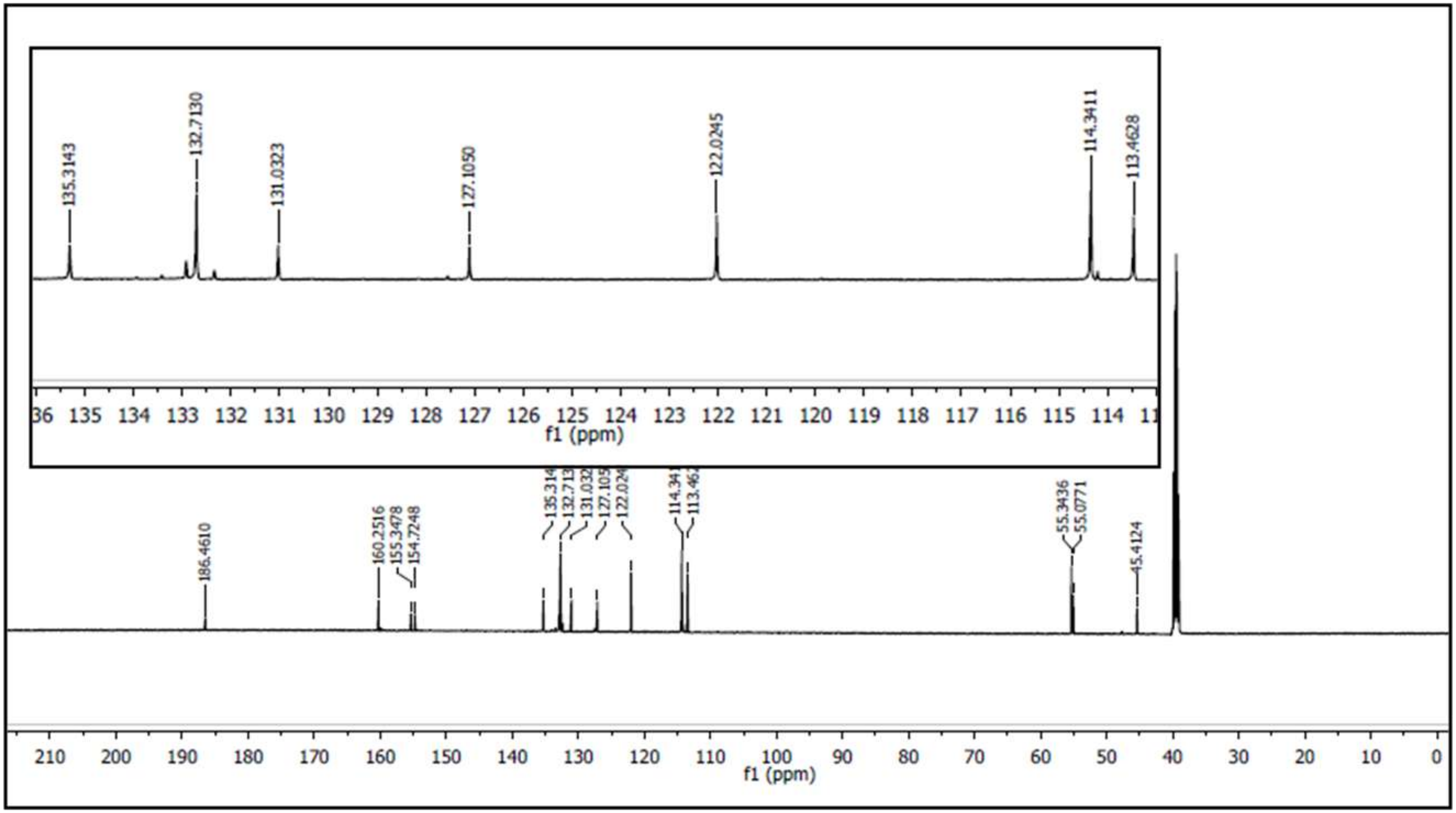

Fig. S66. ^{13}C-NMR spectrum of compound **45** in DMSO-d_6.

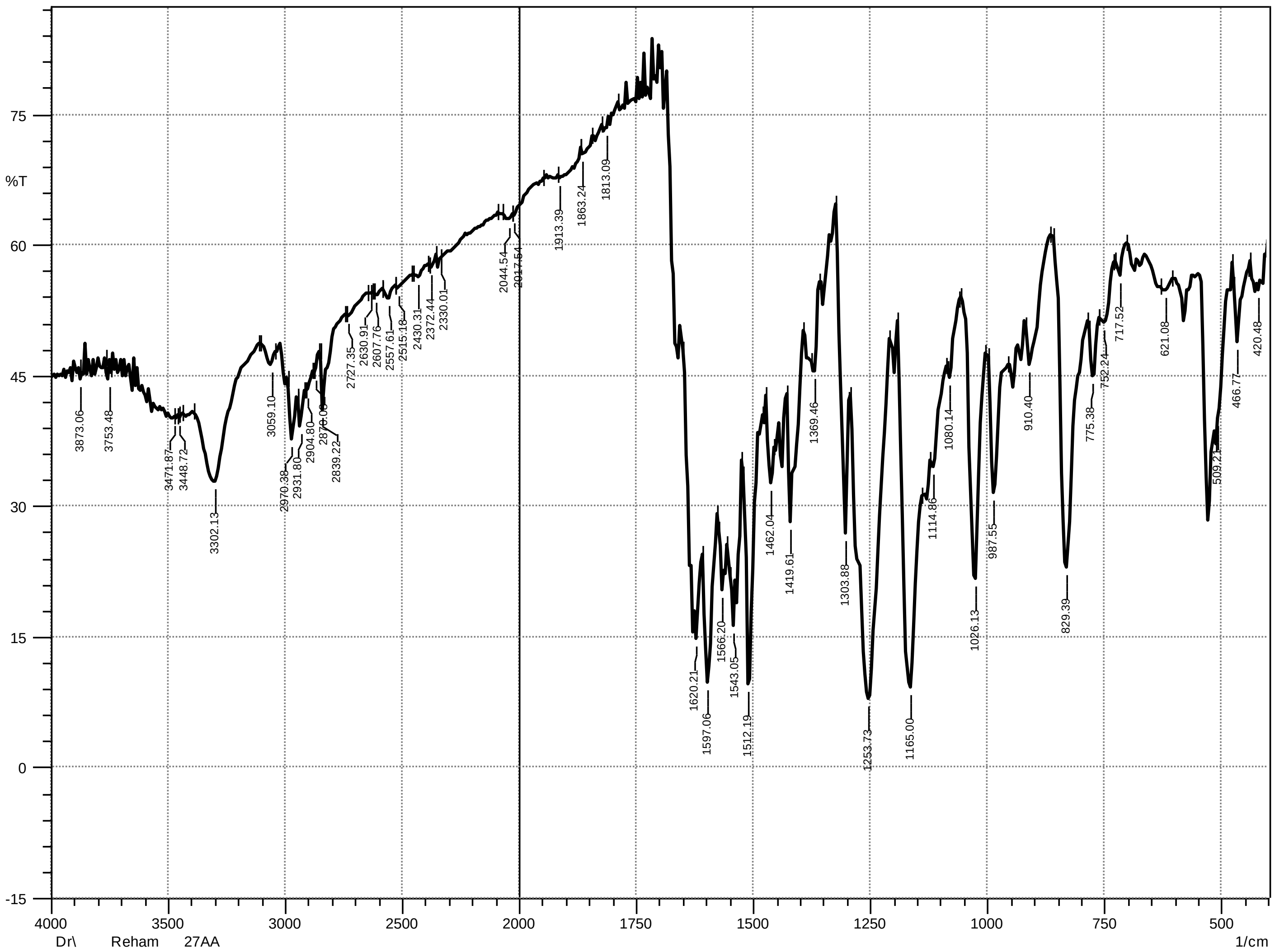

Fig. S67. IR spectrum of compound **46** (KBr pellet).

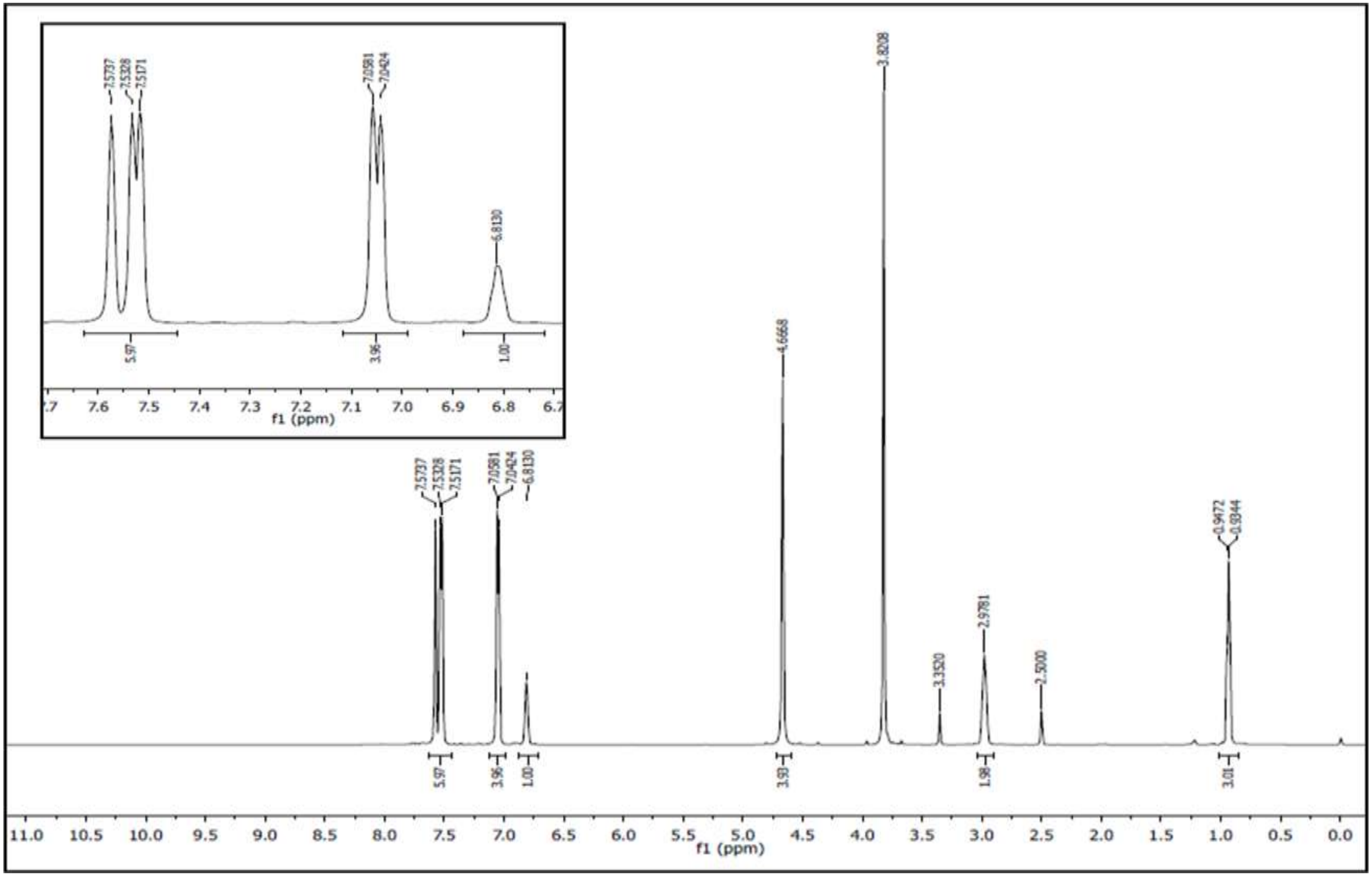

Fig. S68. ^{1}H-NMR spectrum of compound **46** in DMSO-d_6.

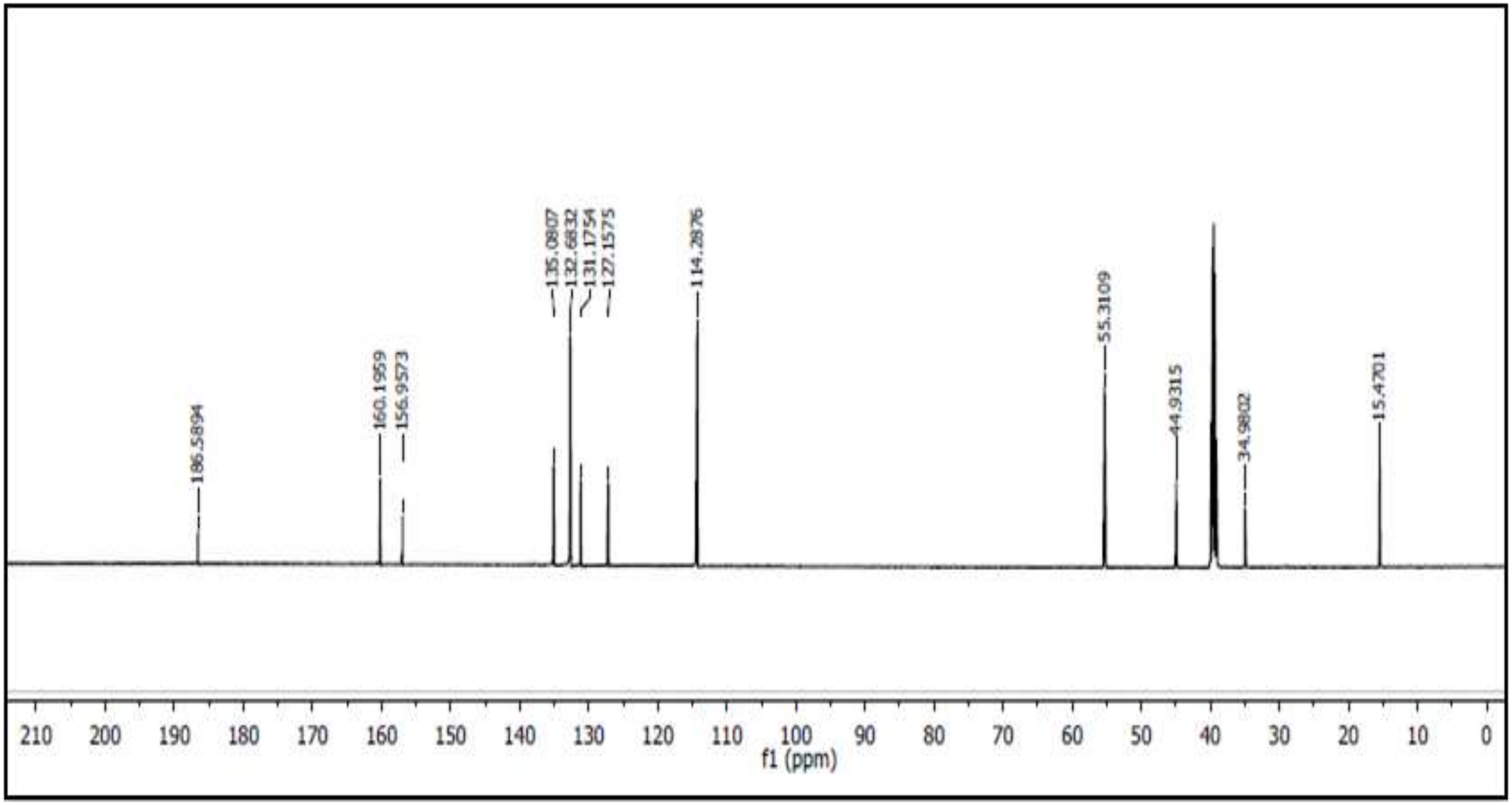

Fig. S69. ^{13}C-NMR spectrum of compound **46** in DMSO-d_6.

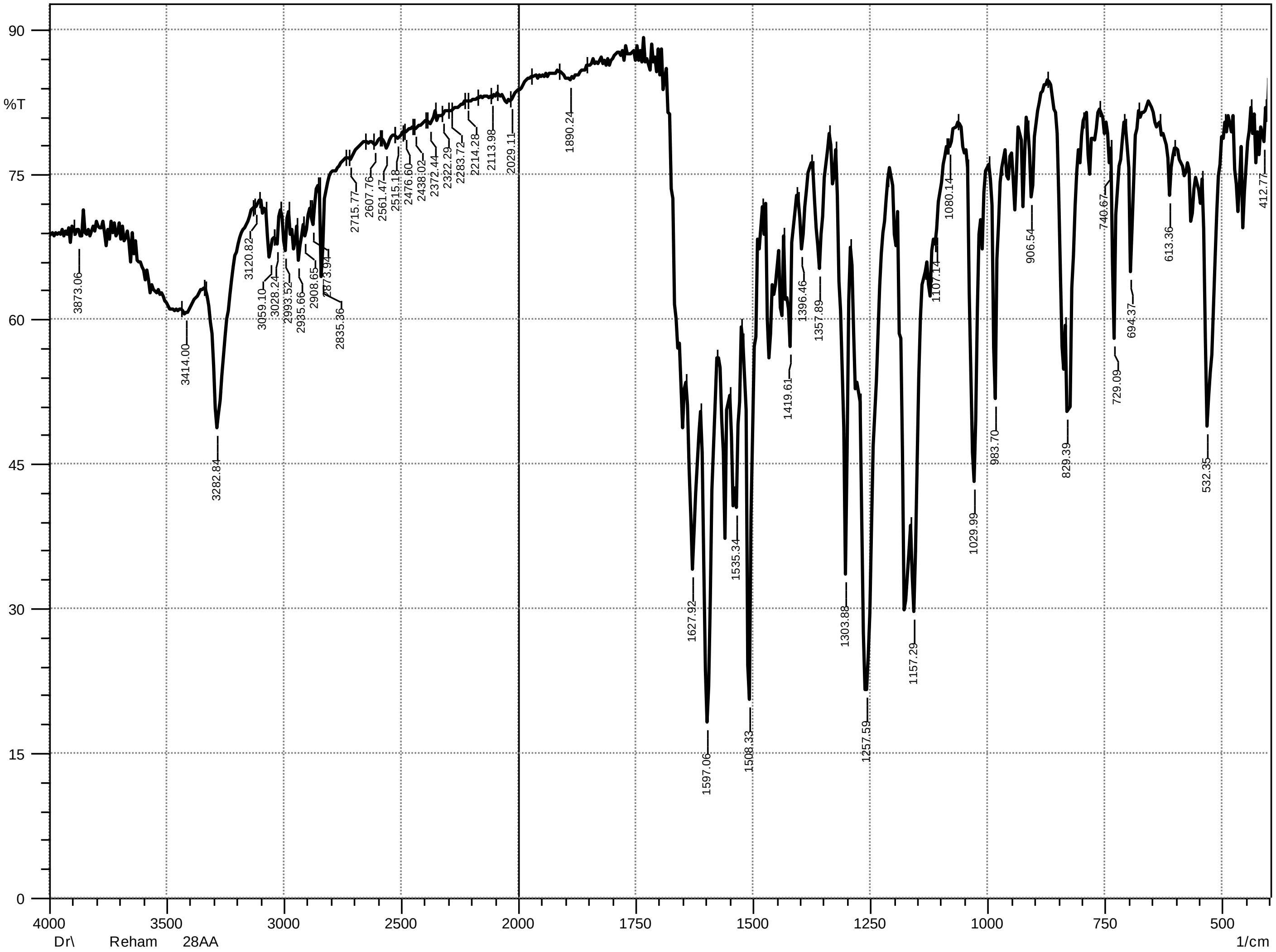

Fig. S70. IR spectrum of compound **47** (KBr pellet).

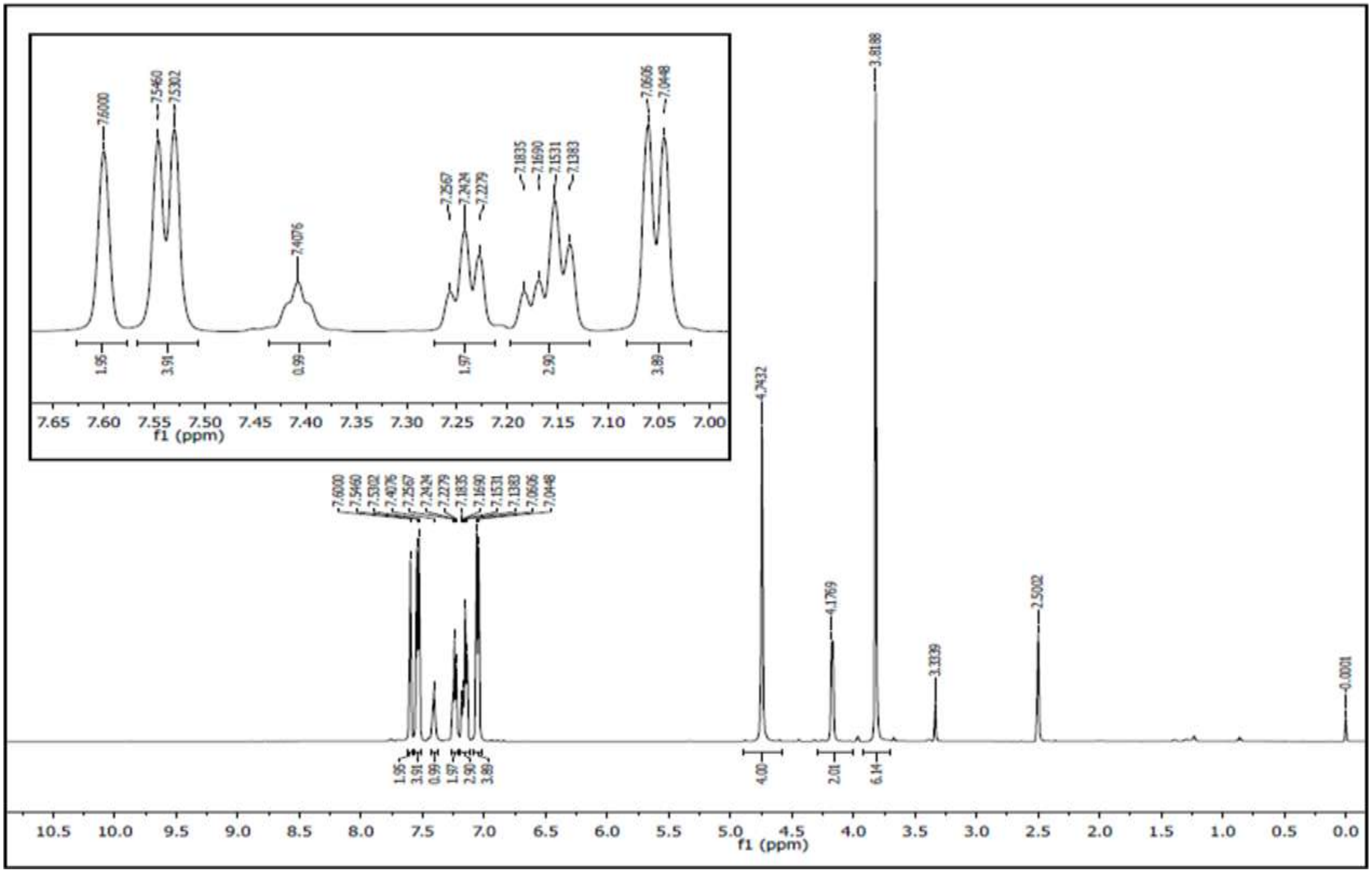

Fig. S71. ^{1}H-NMR spectrum of compound **47** in DMSO-d_6.

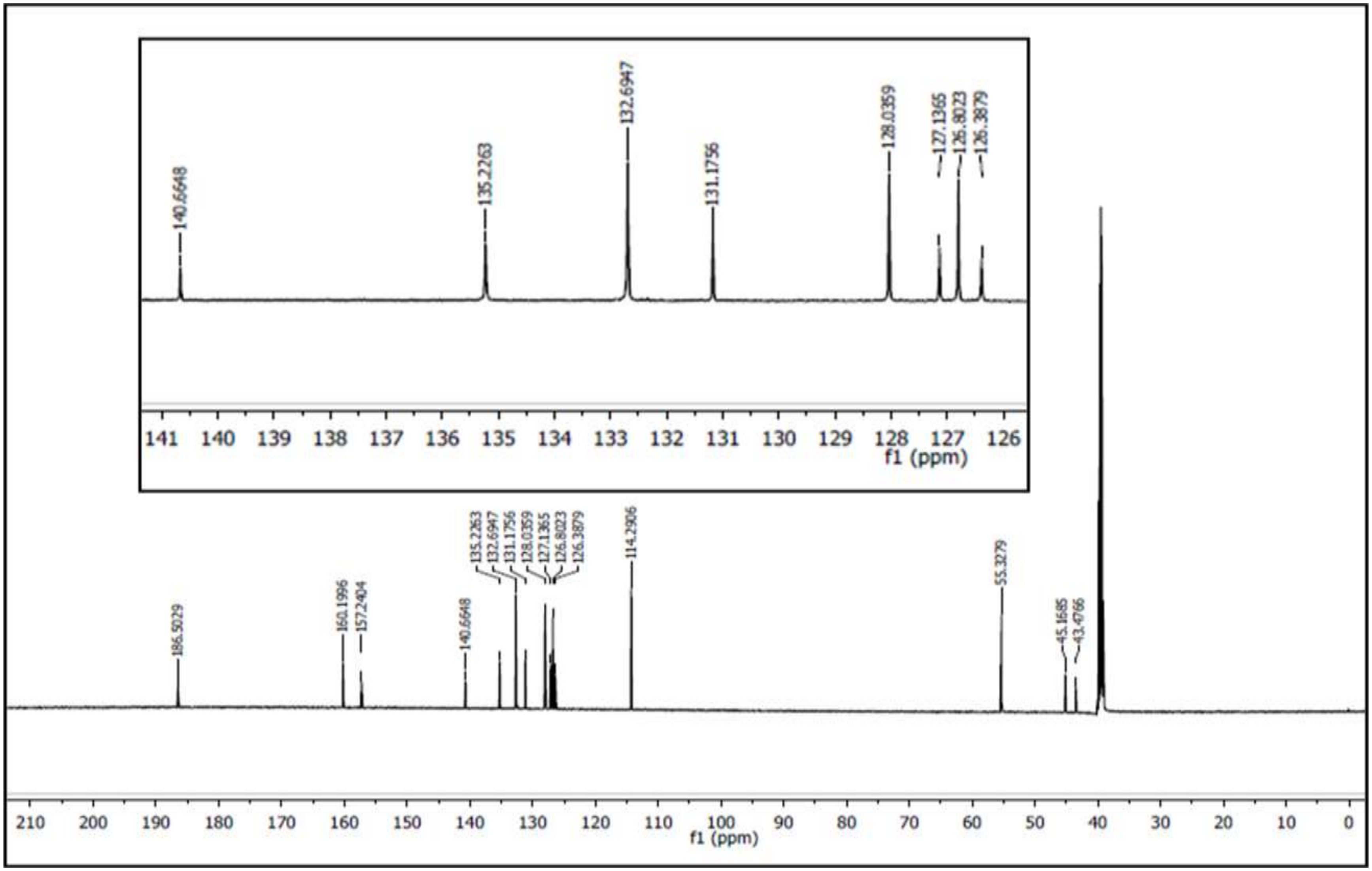

Fig. S72. ^{13}C-NMR spectrum of compound **47** in DMSO-d_6.

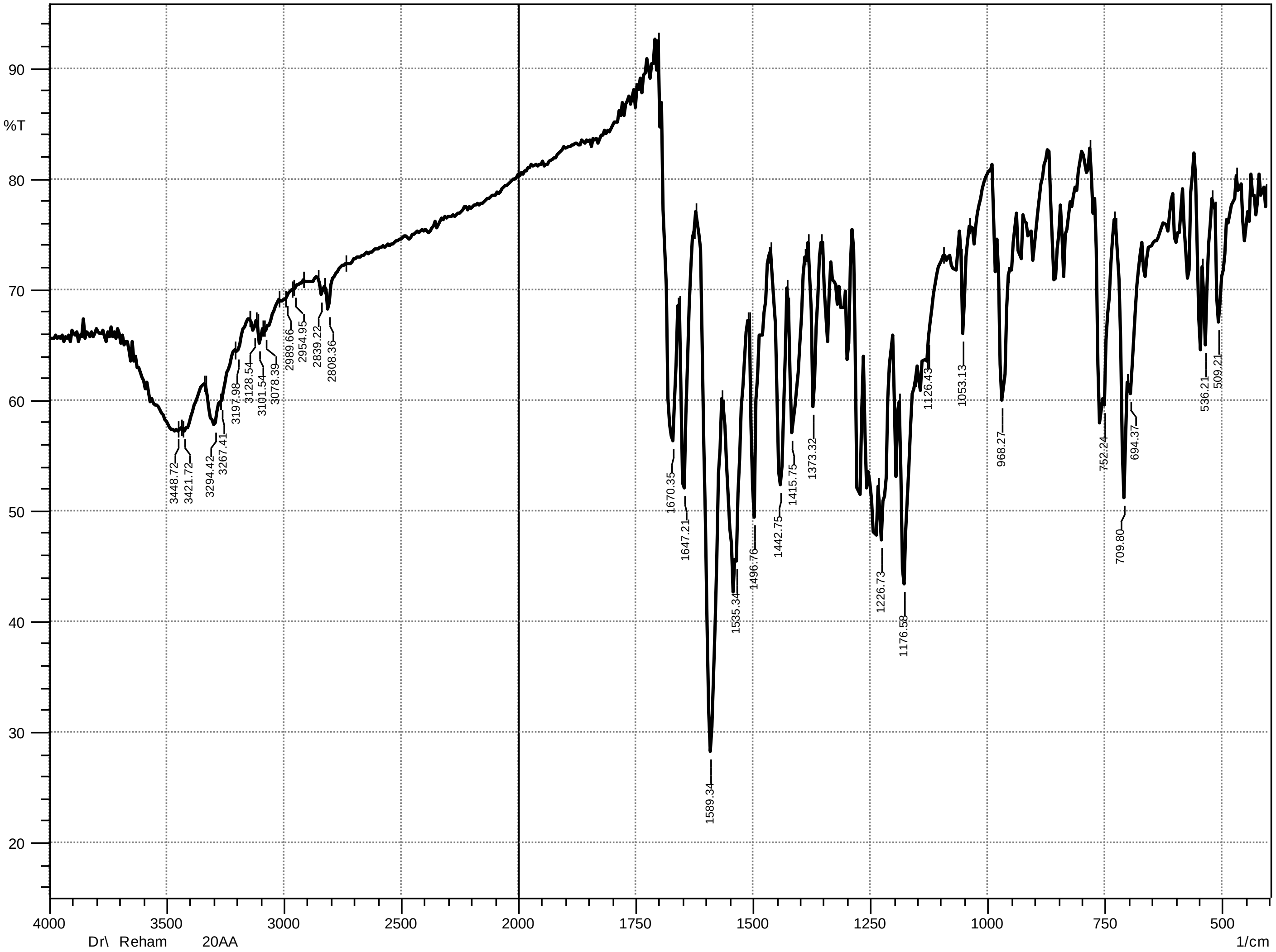

Fig. S73. IR spectrum of compound **48** (KBr pellet).

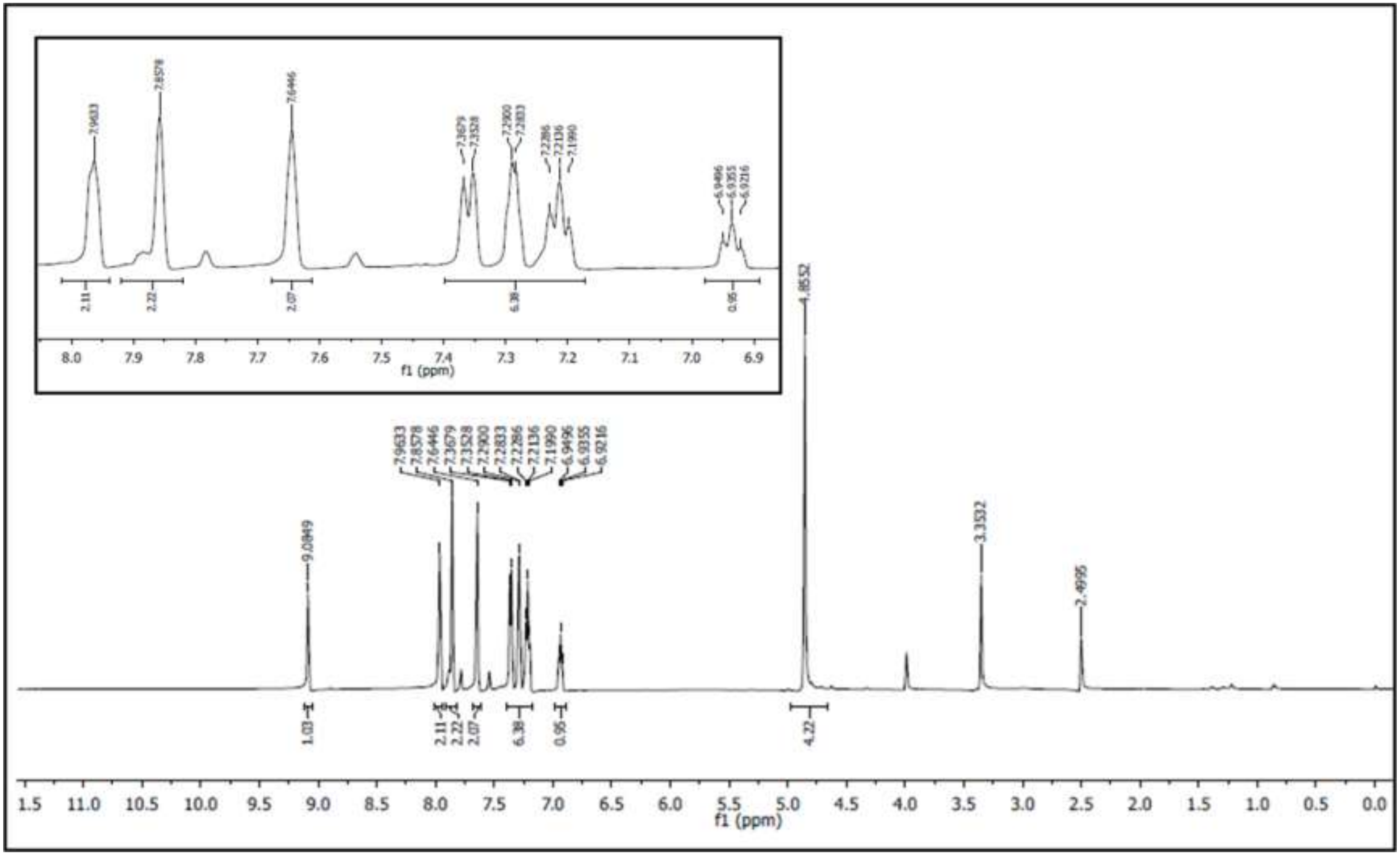

Fig. S74. ^{1}H-NMR spectrum of compound **48** in DMSO-d_6.

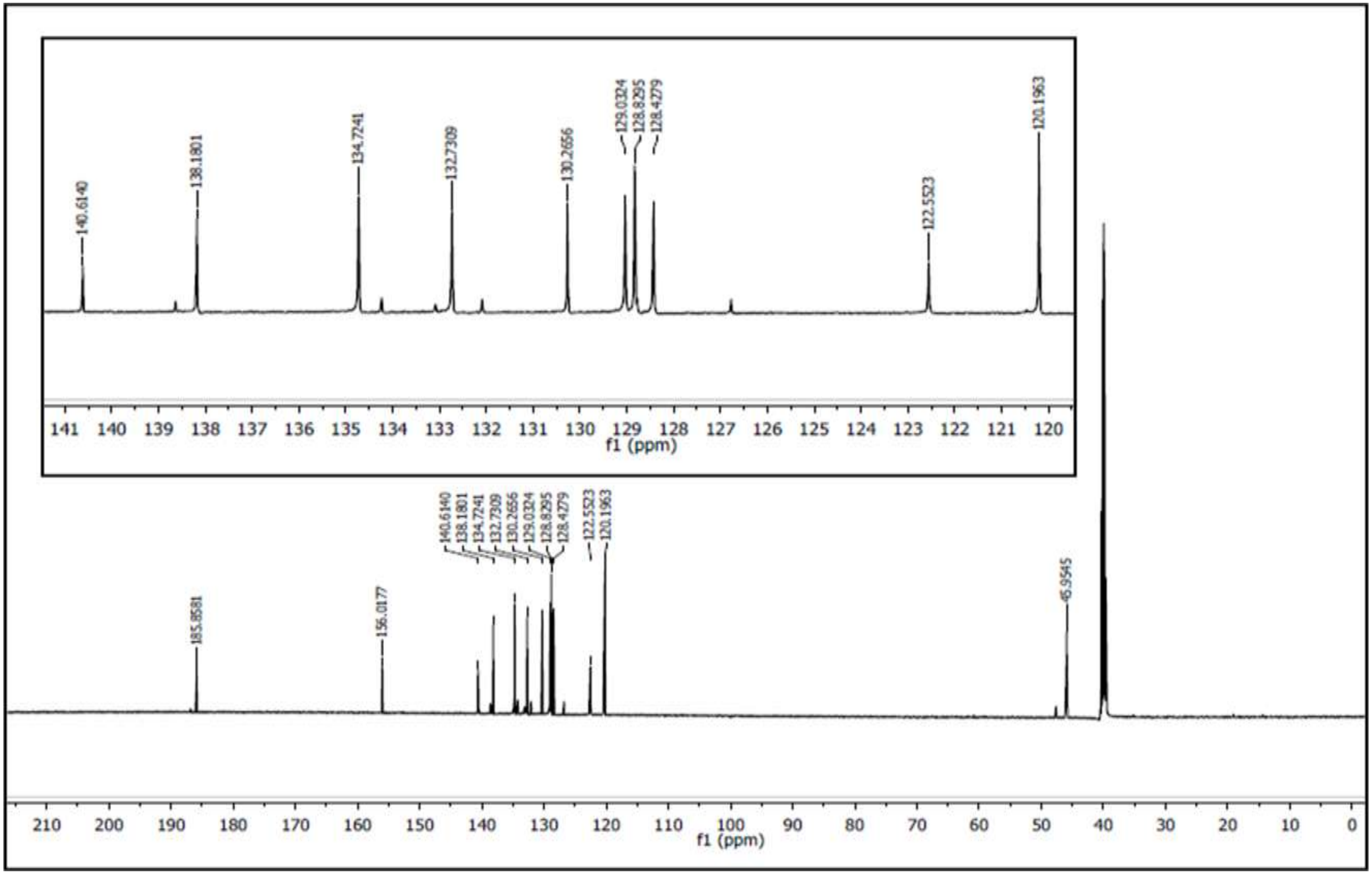

Fig. S75. ^{13}C-NMR spectrum of compound **48** in DMSO-d_6.

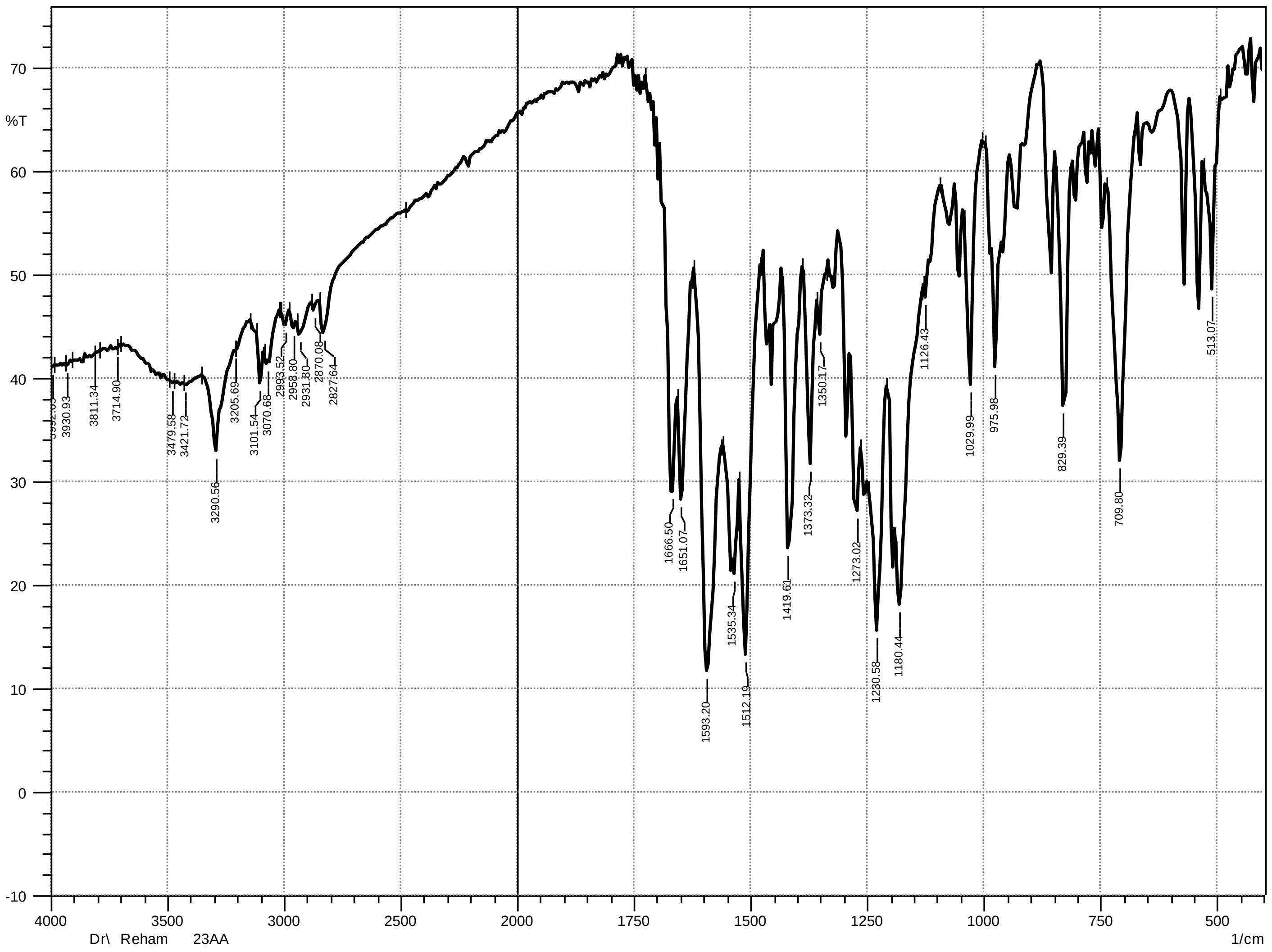

Fig. S76. IR spectrum of compound **49** (KBr pellet).

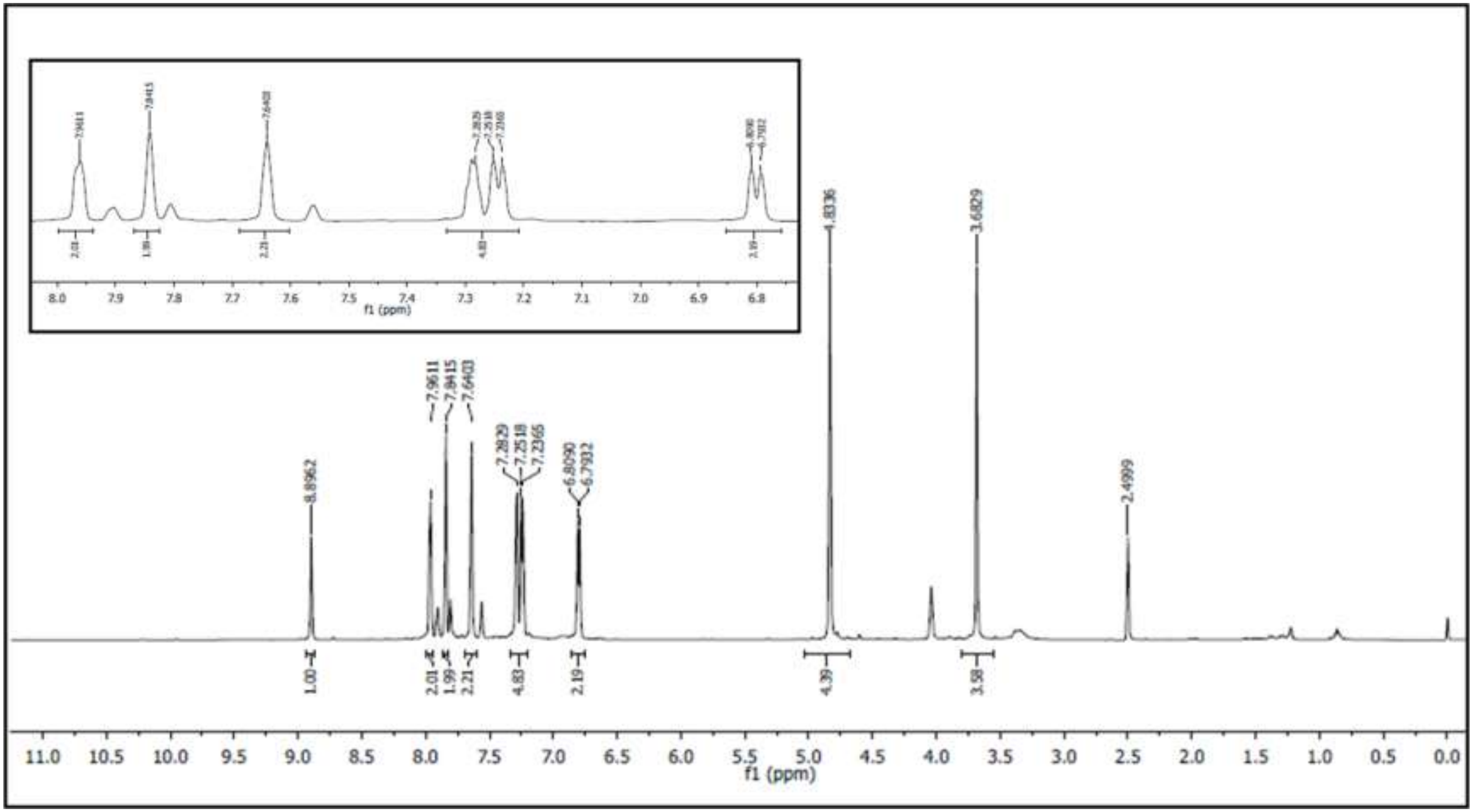

Fig. S77. ^{1}H-NMR spectrum of compound **49** in DMSO-d_6.

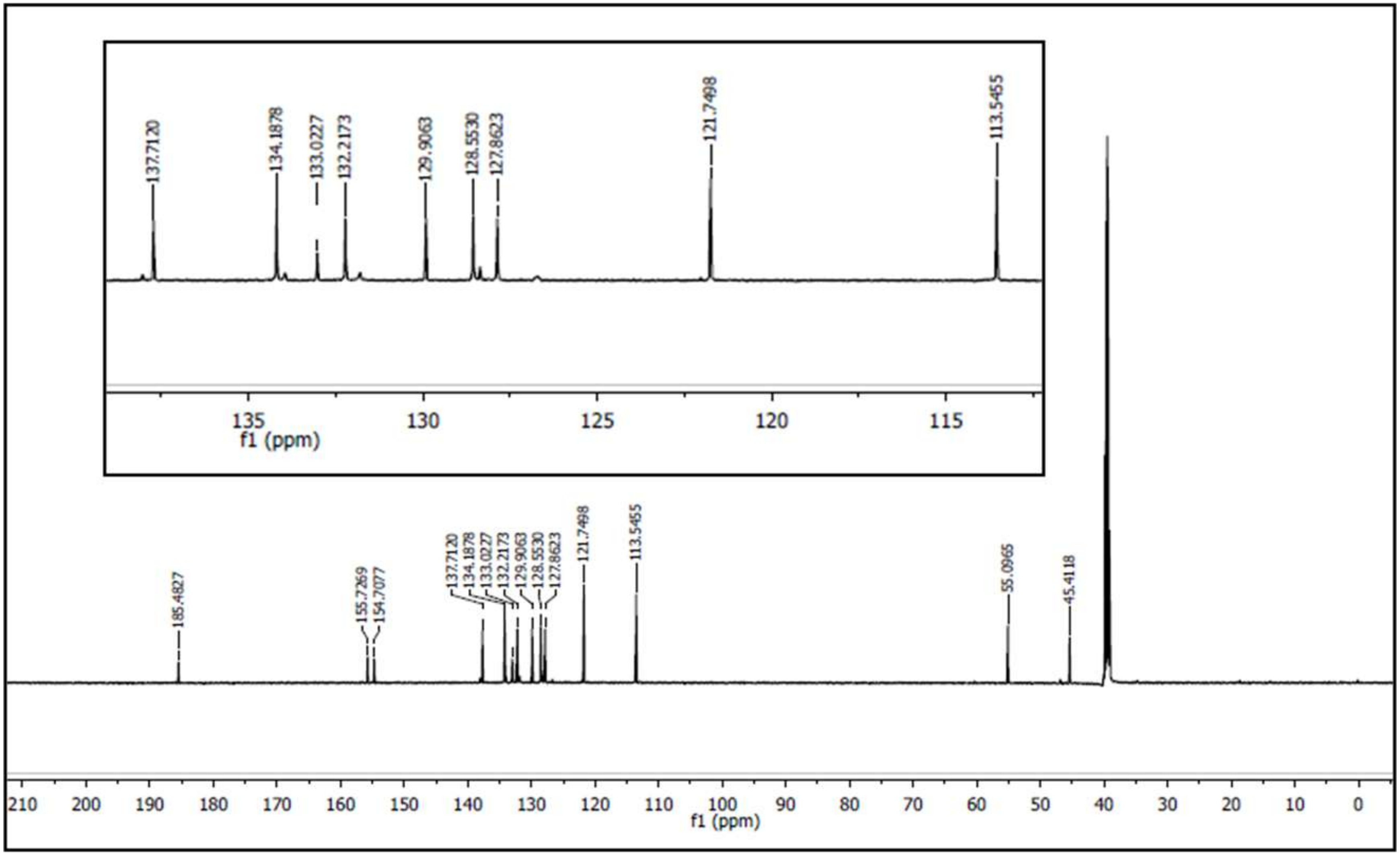

Fig. S78. ^{13}C-NMR spectrum of compound **49** in DMSO-d_6.

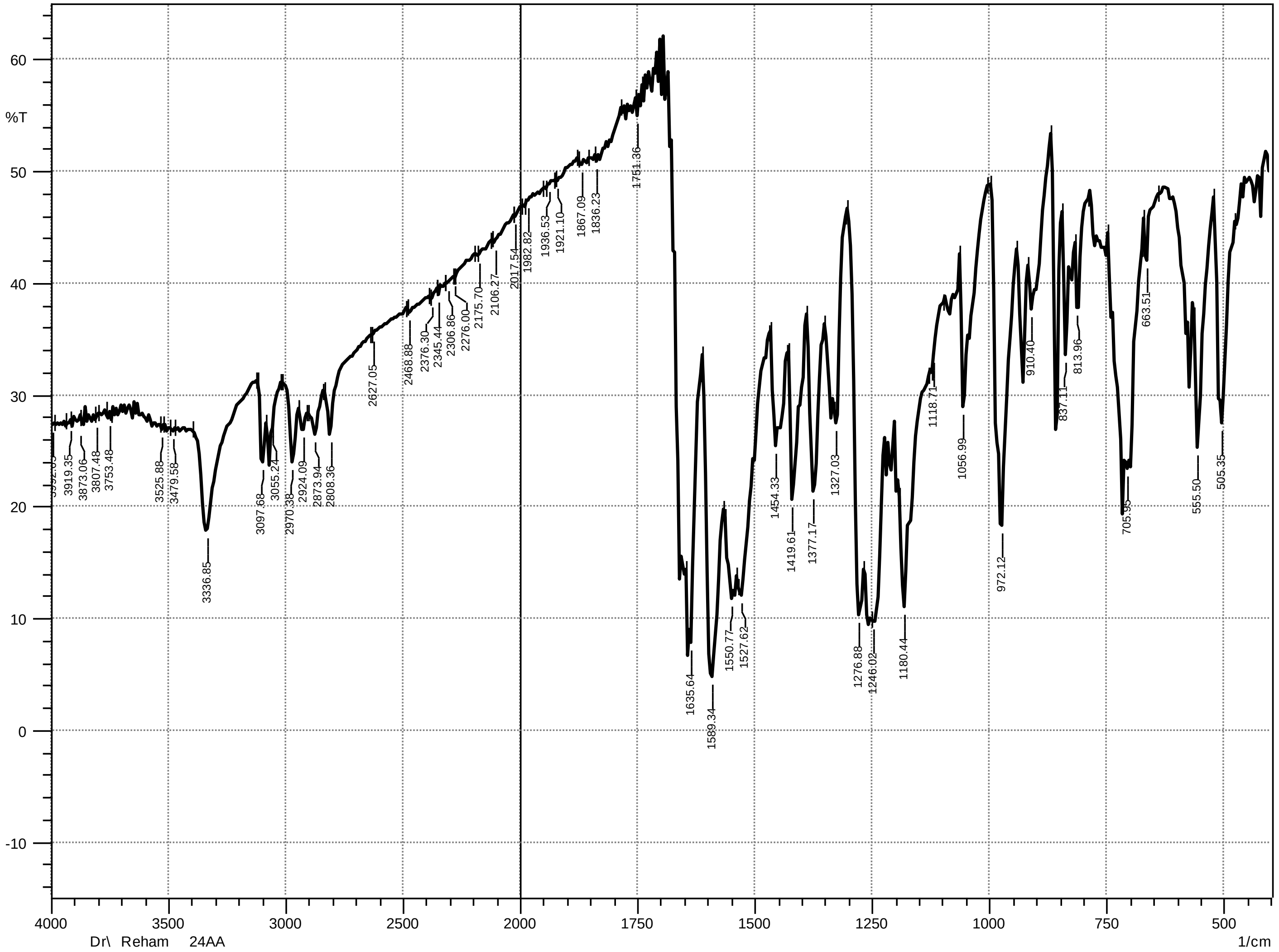

Fig. S79. IR spectrum of compound **50** (KBr pellet).

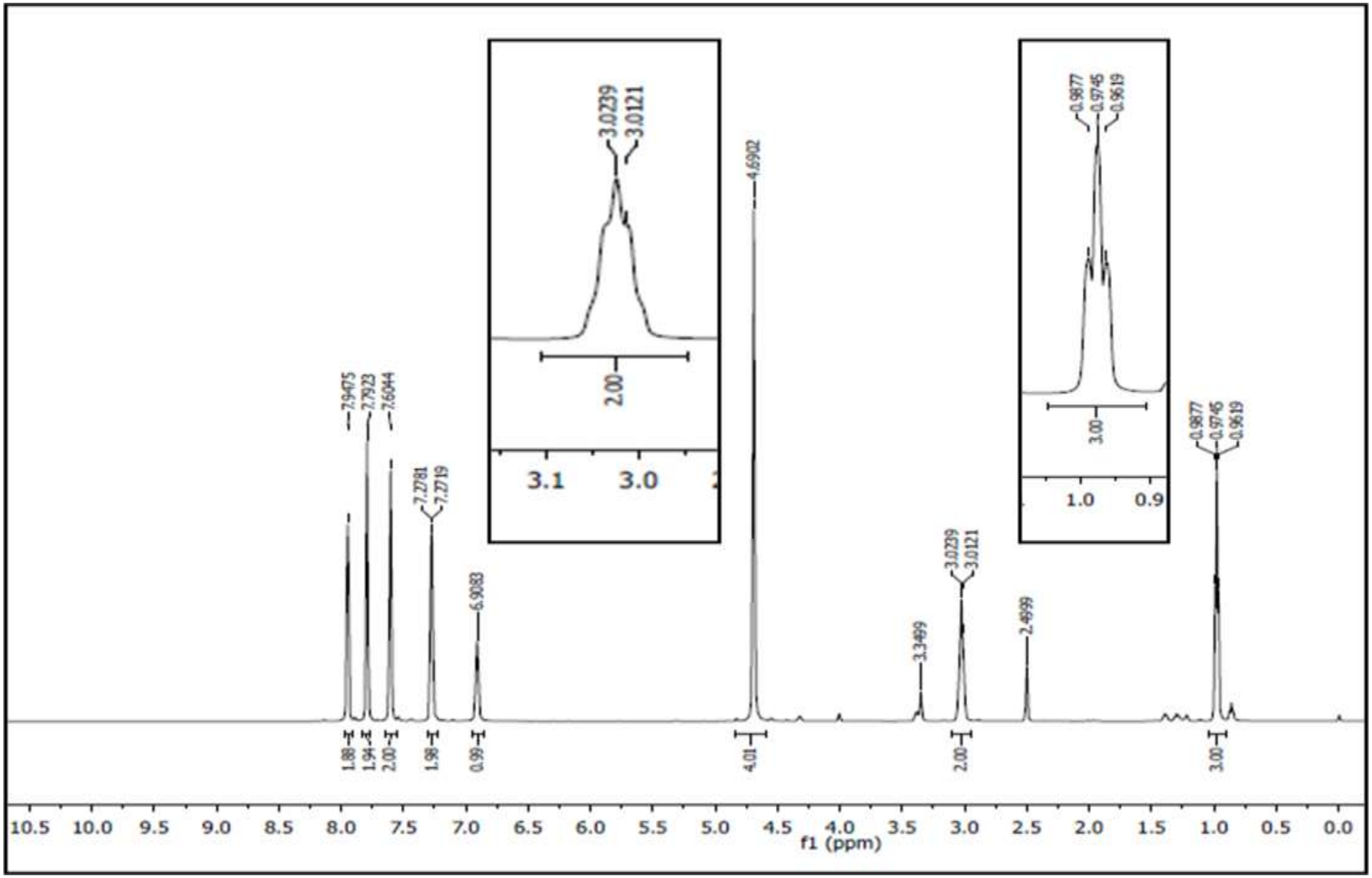

Fig. S80. ^{1}H-NMR spectrum of compound **50** in DMSO-d_6.

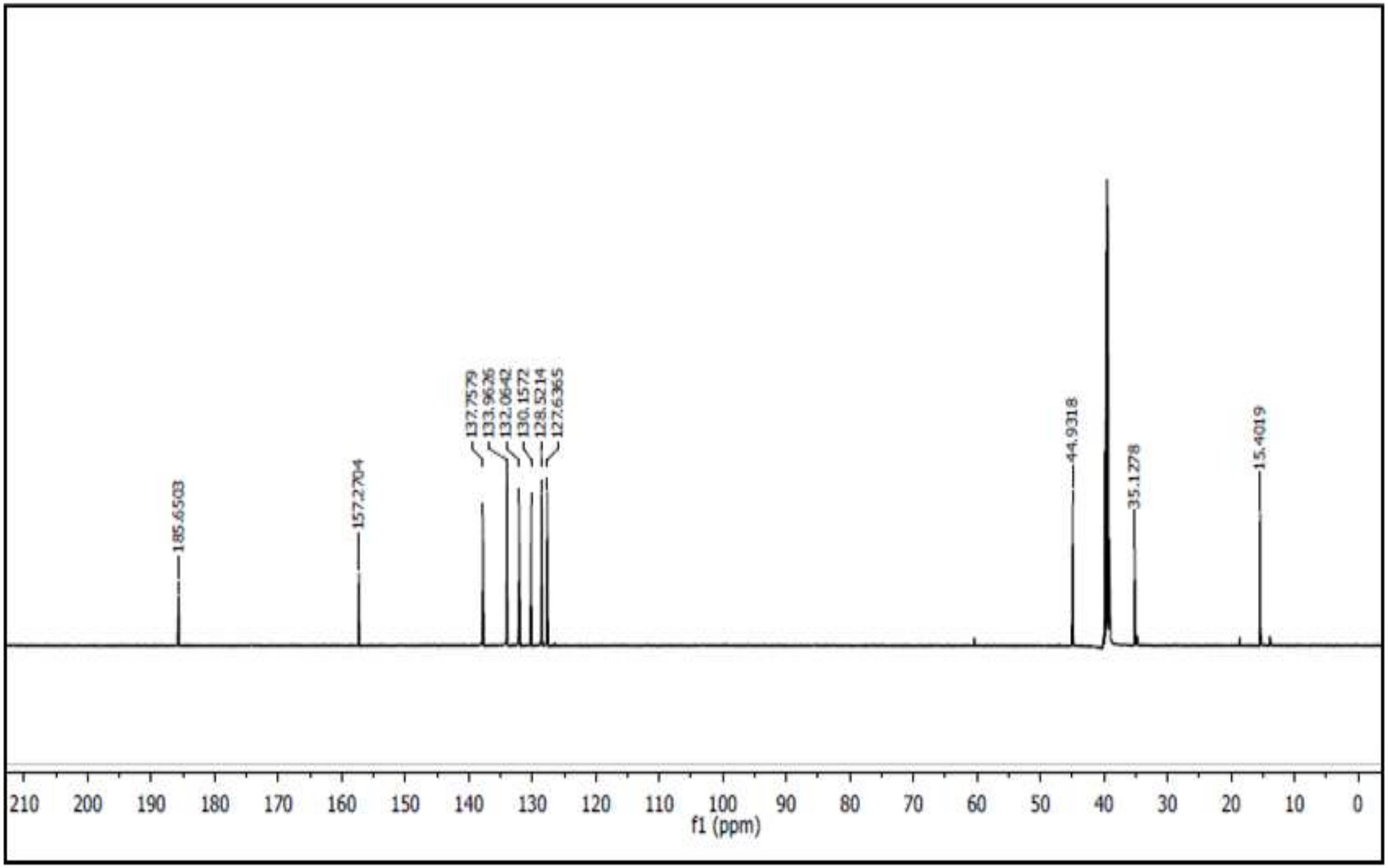

Fig. S81. ^{13}C-NMR spectrum of compound **50** in DMSO-d_6.

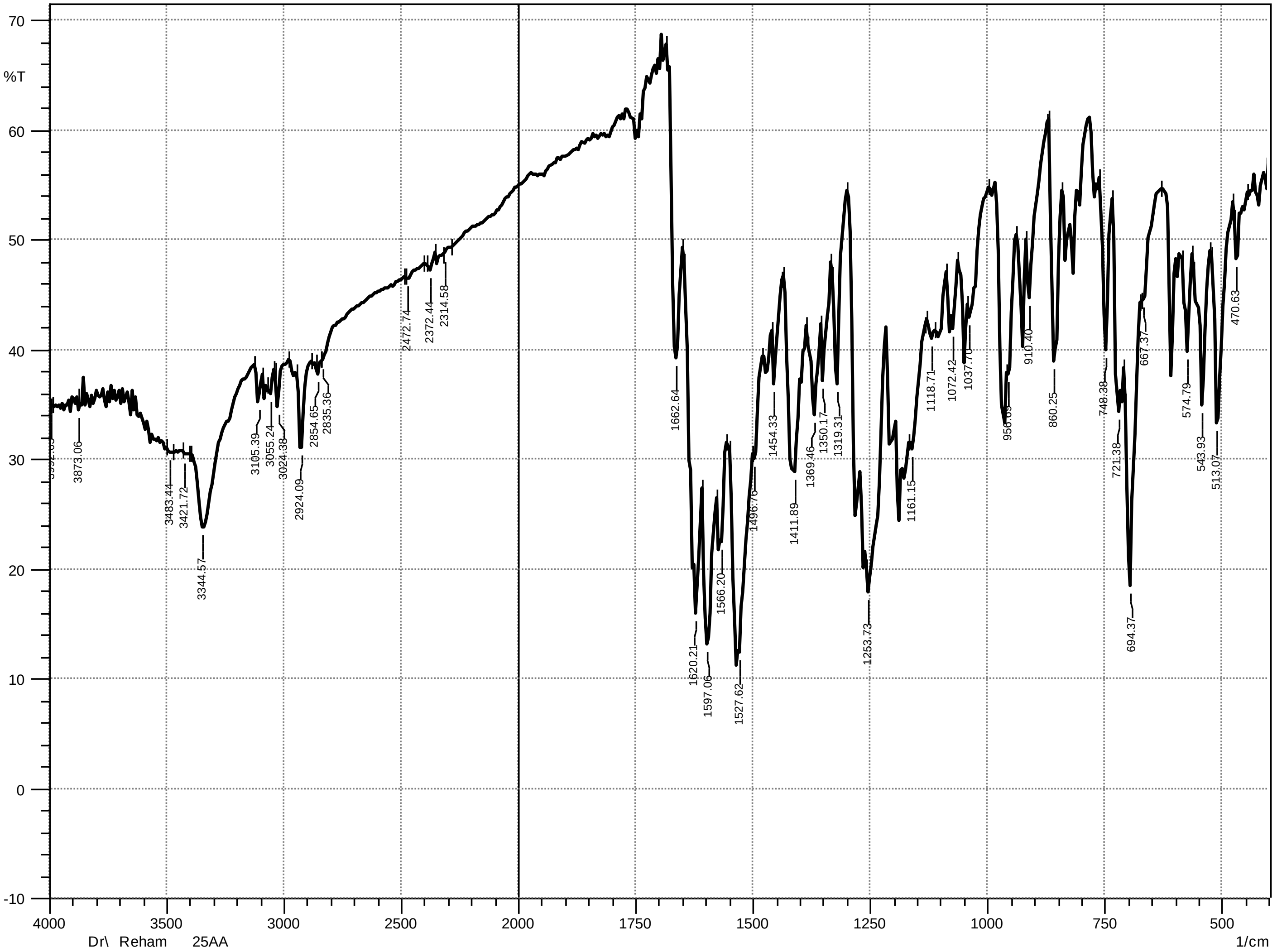

Fig. S82. IR spectrum of compound **51** (KBr pellet).

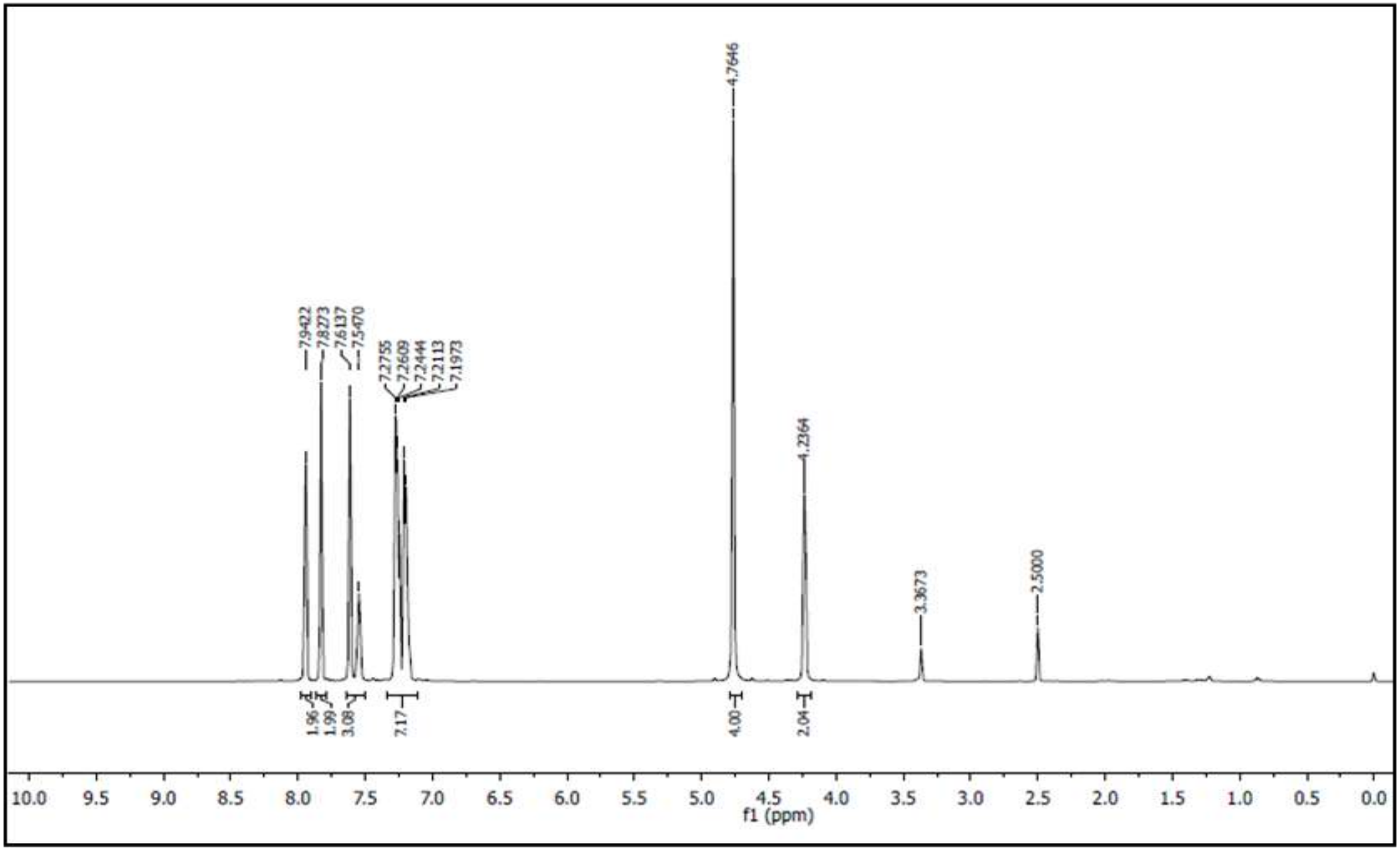

Fig. S83. ^{1}H-NMR spectrum of compound **51** in DMSO-d_6.

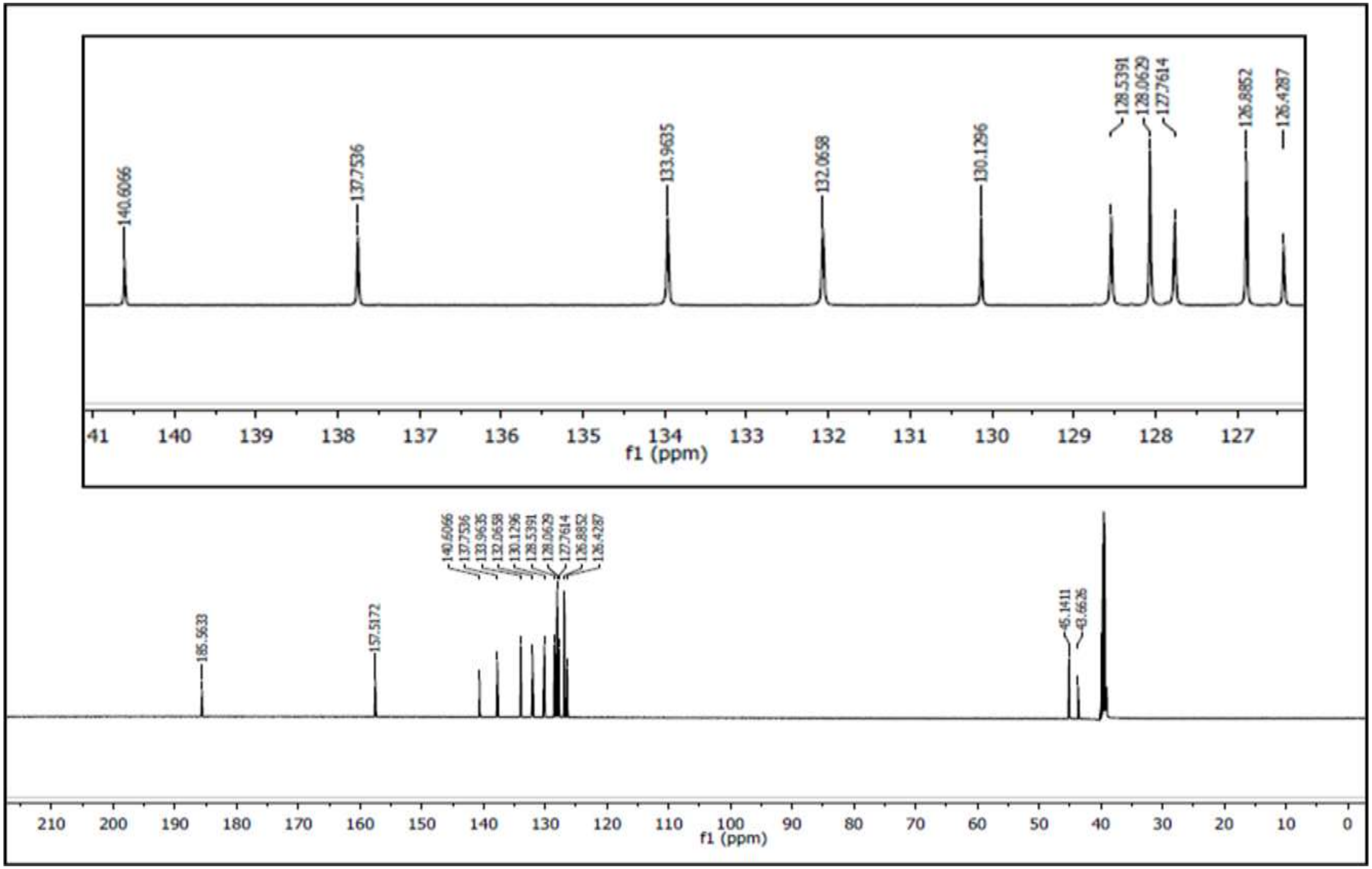

Fig. S84. ^{13}C-NMR spectrum of compound **51** in DMSO-d_6.

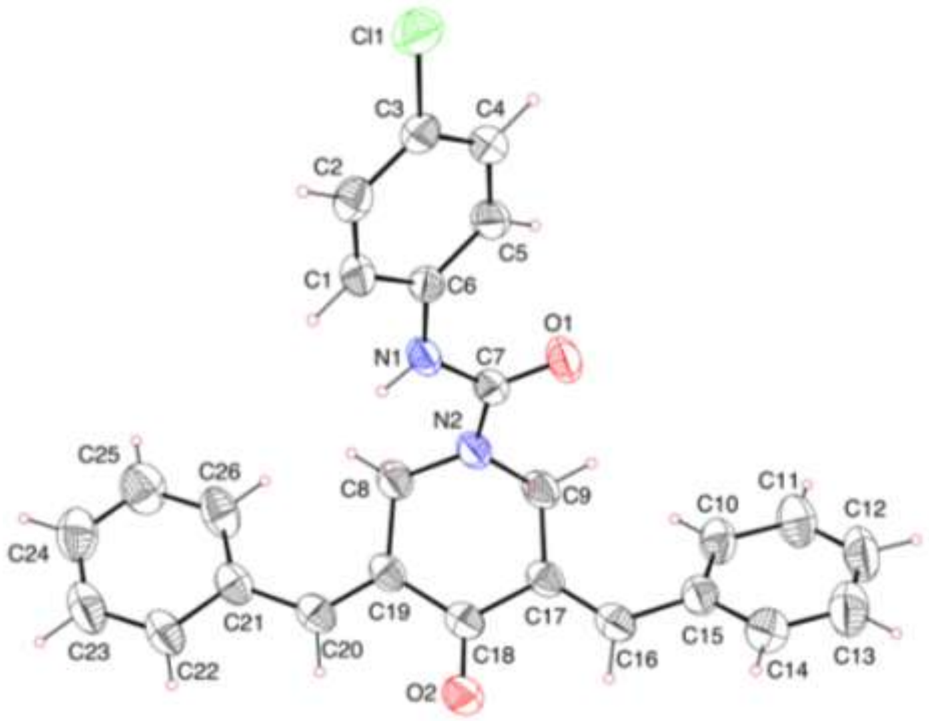

Fig. S85. ORTEP view of compound **25** showing the atom-numbering scheme. Displacement ellipsoids are drawn at the 50 % probability level and H atoms are shown as small spheres of arbitrary radii.

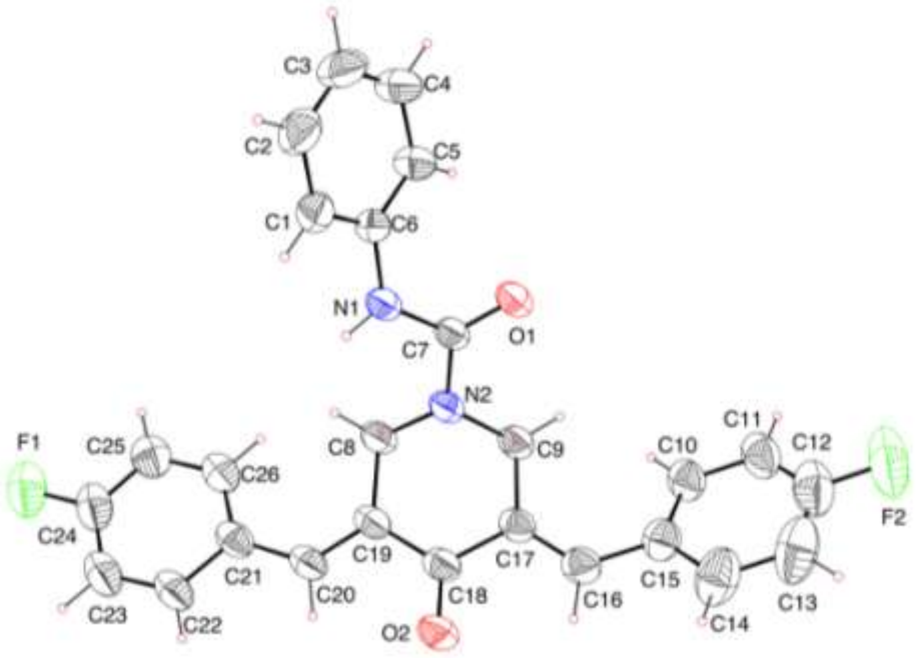

Fig. S86. ORTEP view of compound **34** showing the atom-numbering scheme. Displacement ellipsoids are drawn at the 50 % probability level and H atoms are shown as small spheres of arbitrary radii.

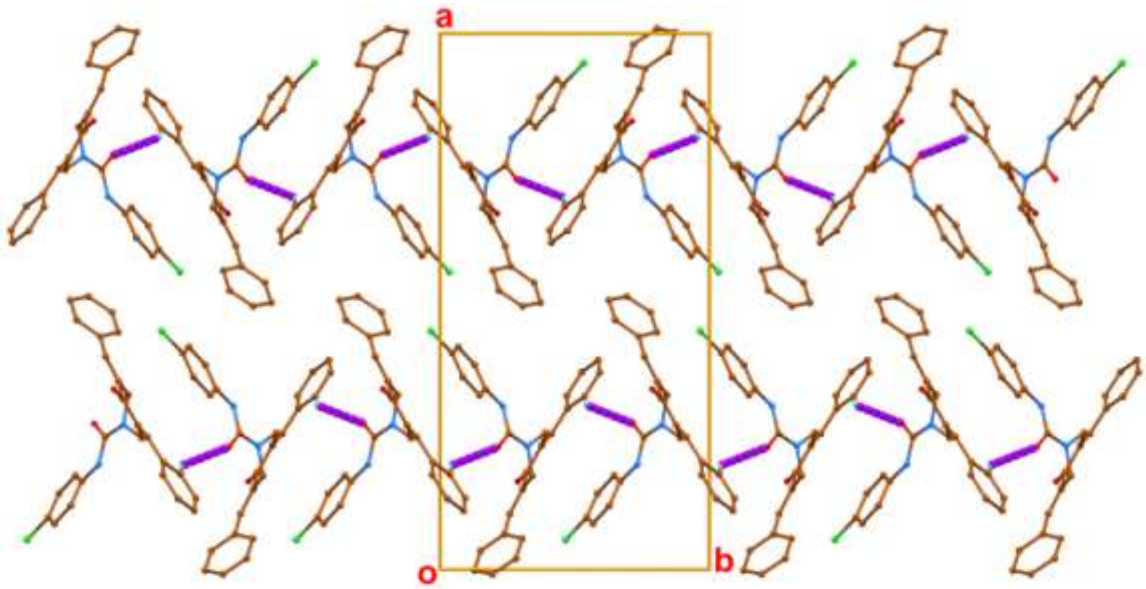

Fig. S87. The crystal packing of compound **25**. The H atoms not engaged in the intermolecular interactions (dashed lines) have been skipped for clarity.

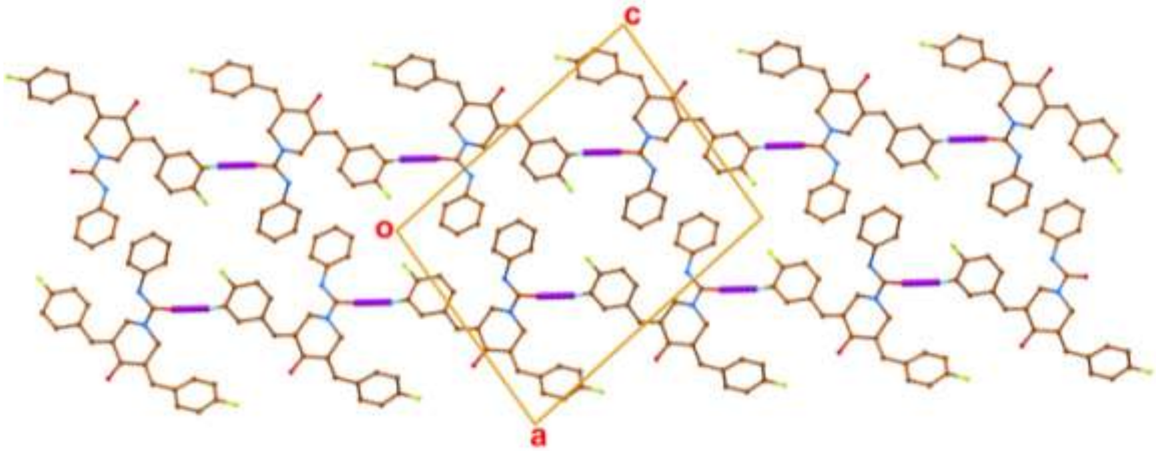

Fig. S88. The crystal packing of compound **34**. The H atoms not engaged in the intermolecular interactions (dashed lines) have been skipped for clarity.

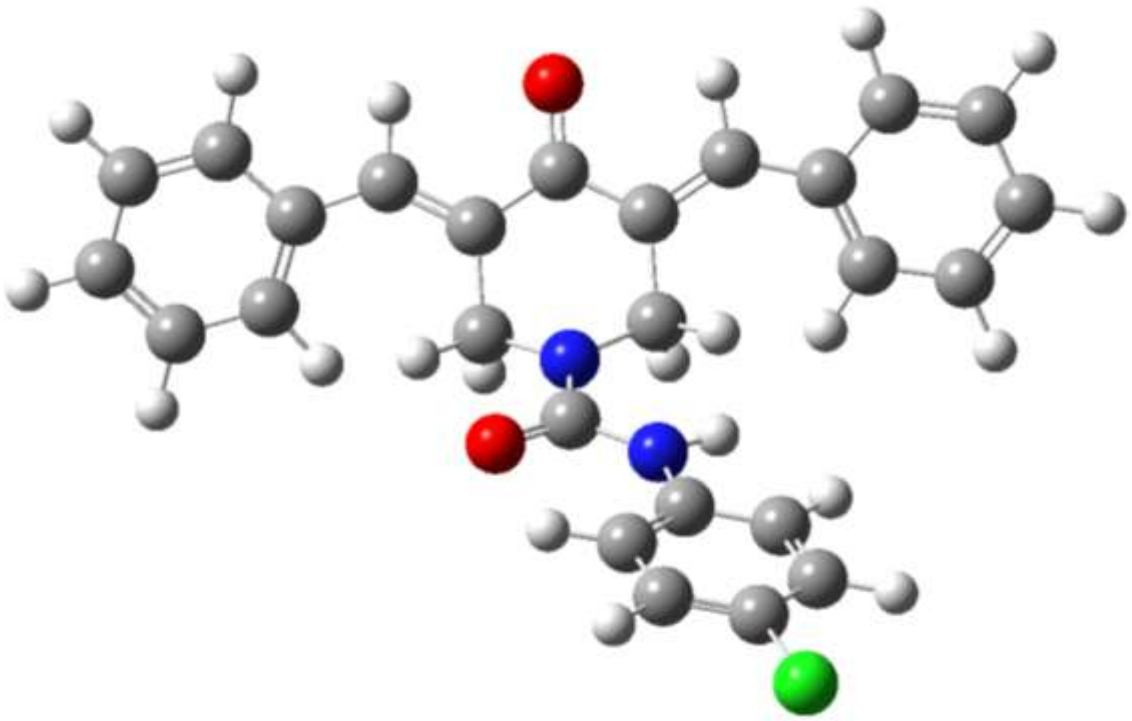

Fig. S89. A projection of the optimized structure of compound **25** by DFT/B3LYP method with 3-21G* basis set.

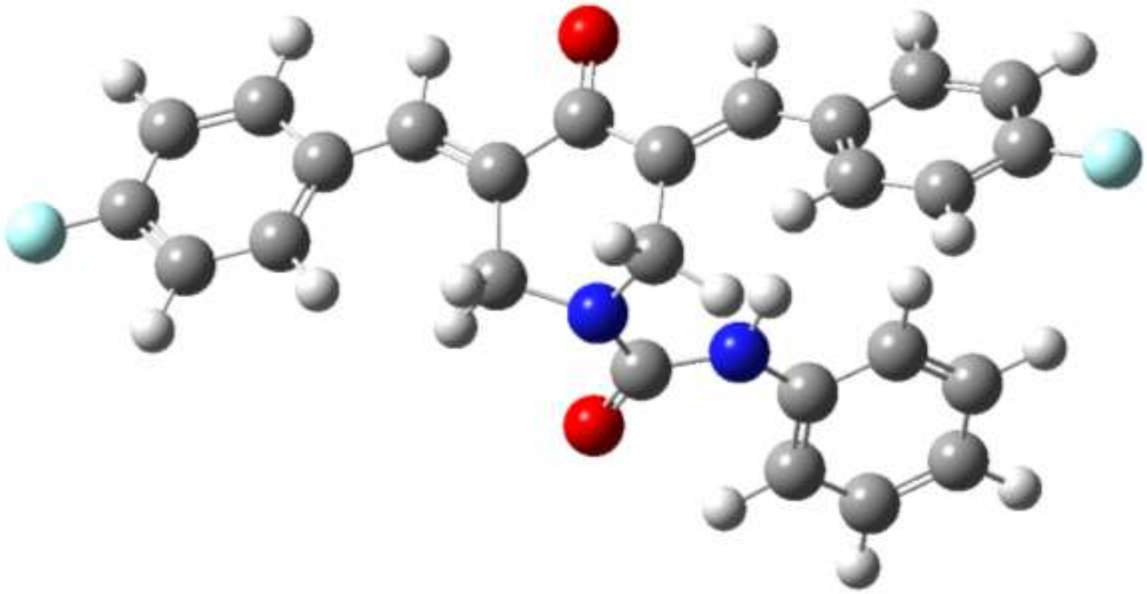

Fig. S90. A projection of the optimized structure of compound **34** by DFT/B3LYP method with 3-21G* basis set.

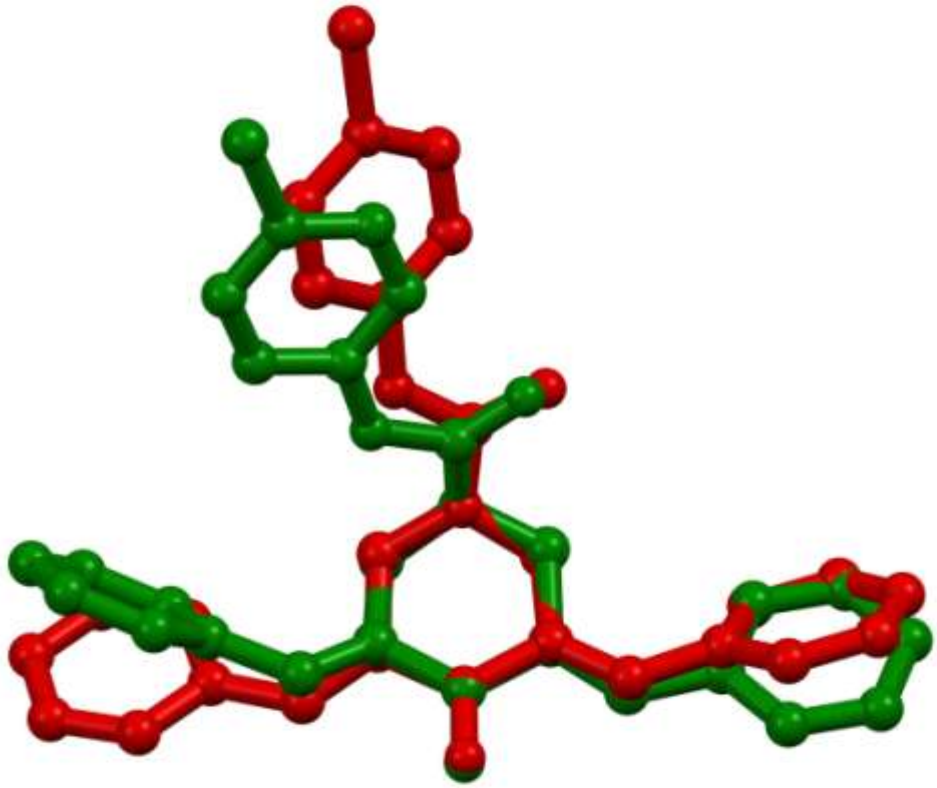

Fig. S91. Overlay diagram of compound **25**; red (X-ray structure), green (DFT).

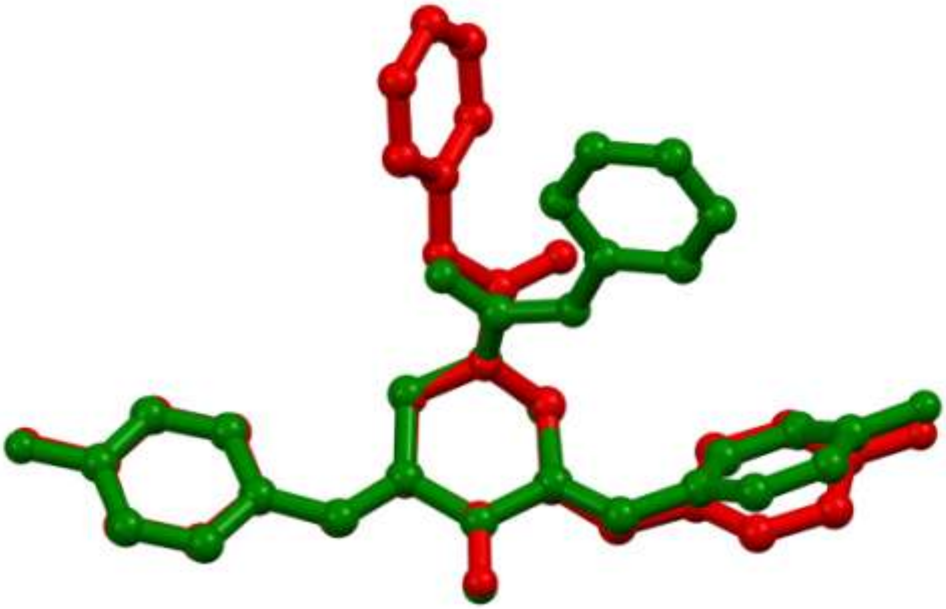

Fig. S92. Overlay diagram of compound **34**; red (X-ray structure), green (DFT).

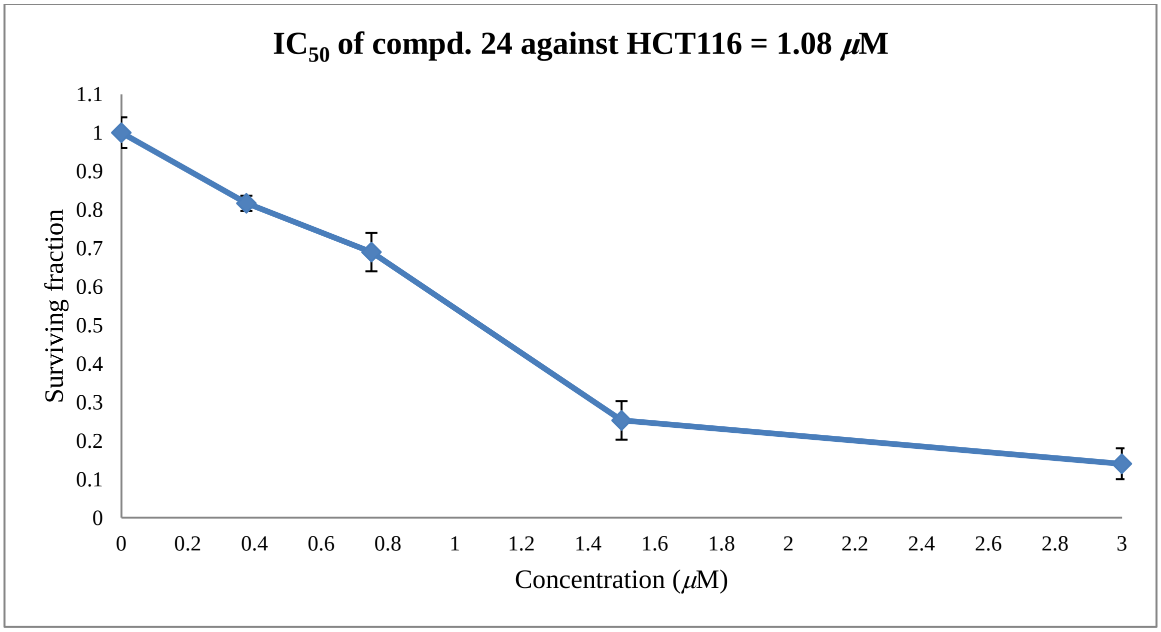
IC50 of compd. 24 against HCT116 = 1.08 μM
Surviving fraction
1.1
1
0.9
0.8
0.7
0.6
0.5
0.4
0.3
0.2
0.1
0
0 0.2 0.4 0.6 0.8 1 1.2 1.4 1.6 1.8 2 2.2 2.4 2.6 2.8 3
Concentration (μM)

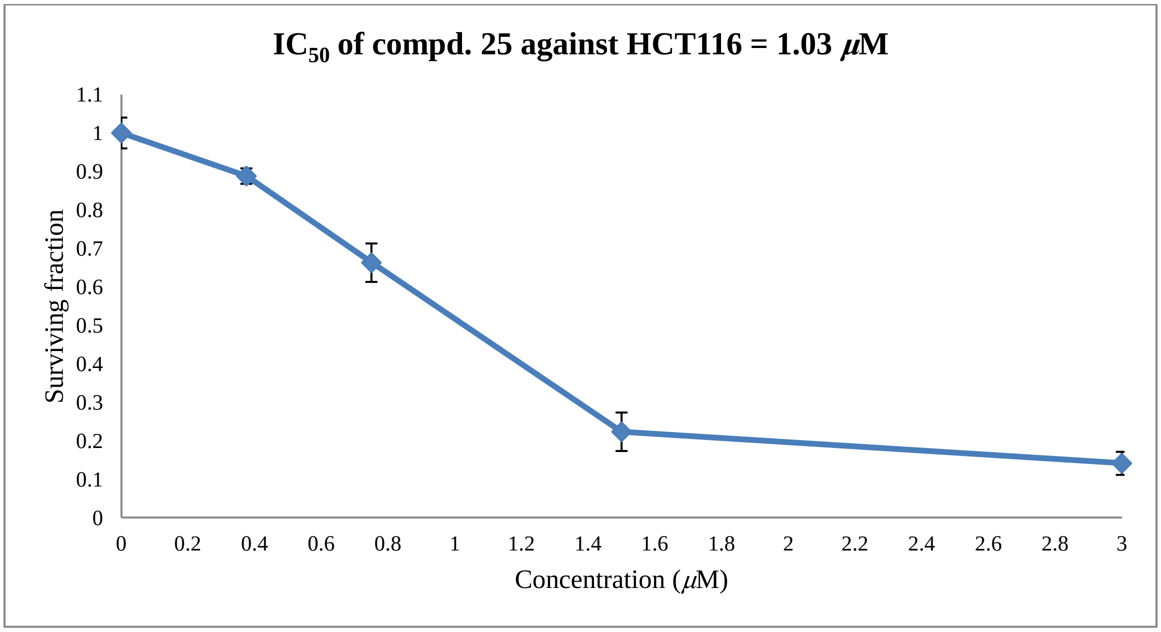
IC50 of compd. 25 against HCT116 = 1.03 μM
Surviving fraction
1.1
1
0.9
0.8
0.7
0.6
0.5
0.4
0.3
0.2
0.1
0
0 0.2 0.4 0.6 0.8 1 1.2 1.4 1.6 1.8 2 2.2 2.4 2.6 2.8 3
Concentration (μM)

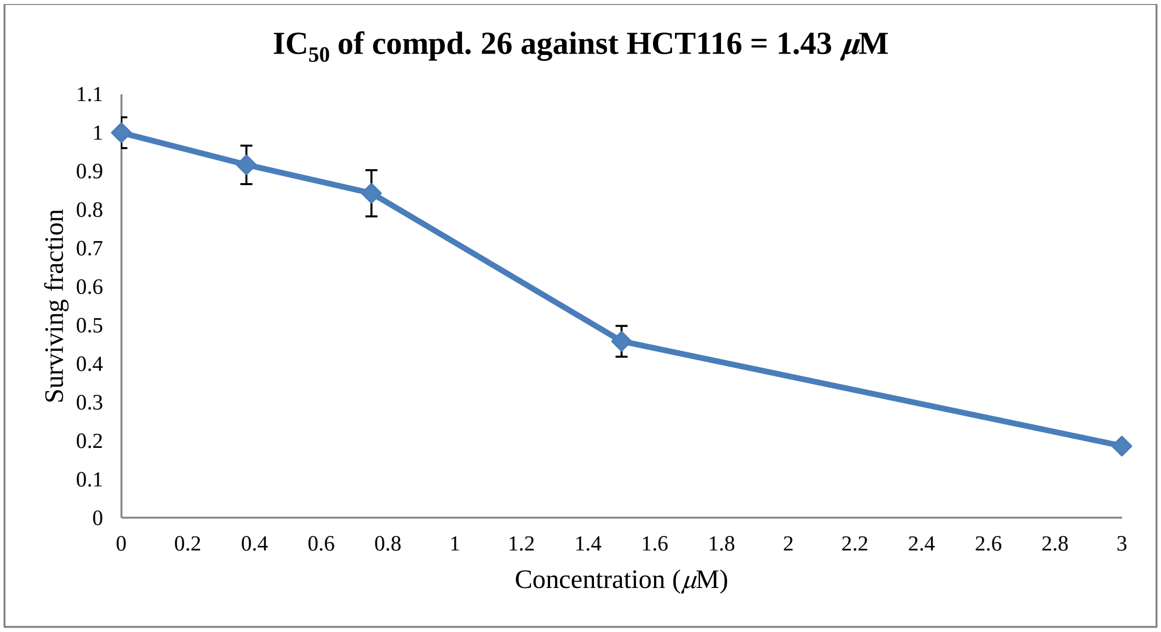
IC$_{50}$ of compd. 26 against HCT116 = 1.43 μM
Surviving fraction
1.1
1
0.9
0.8
0.7
0.6
0.5
0.4
0.3
0.2
0.1
0
0
0.2
0.4
0.6
0.8
1
1.2
1.4
1.6
1.8
2
2.2
2.4
2.6
2.8
3
Concentration (μM)

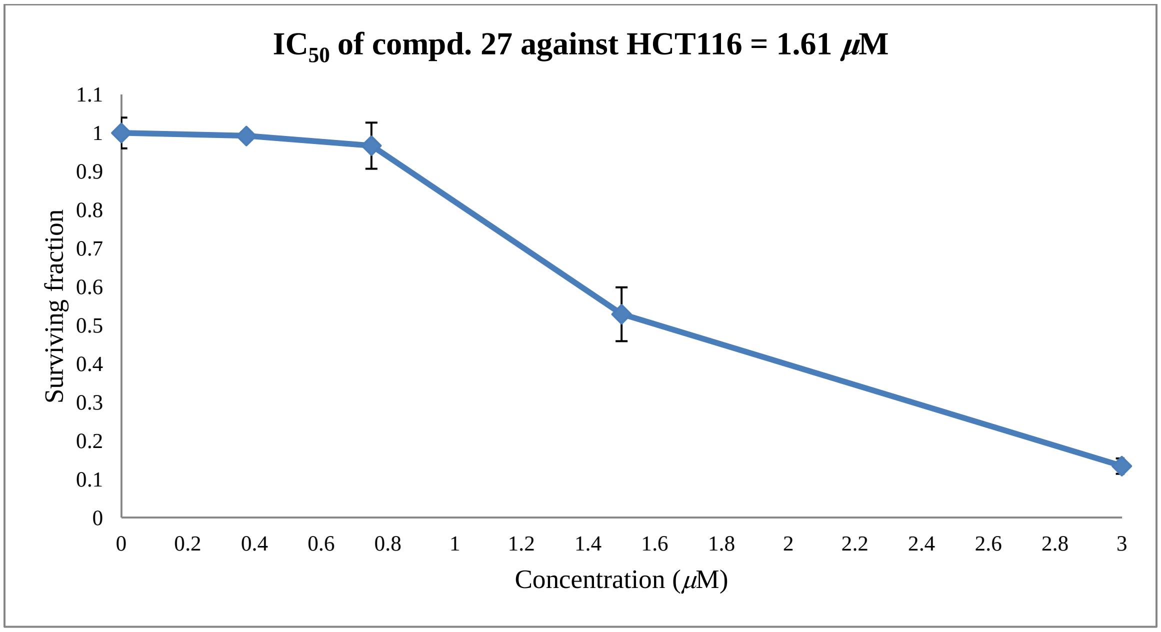
IC$_{50}$ of compd. 27 against HCT116 = 1.61 μM
Surviving fraction
1.1
1
0.9
0.8
0.7
0.6
0.5
0.4
0.3
0.2
0.1
0
0
0.2
0.4
0.6
0.8
1
1.2
1.4
1.6
1.8
2
2.2
2.4
2.6
2.8
3
Concentration (μM)

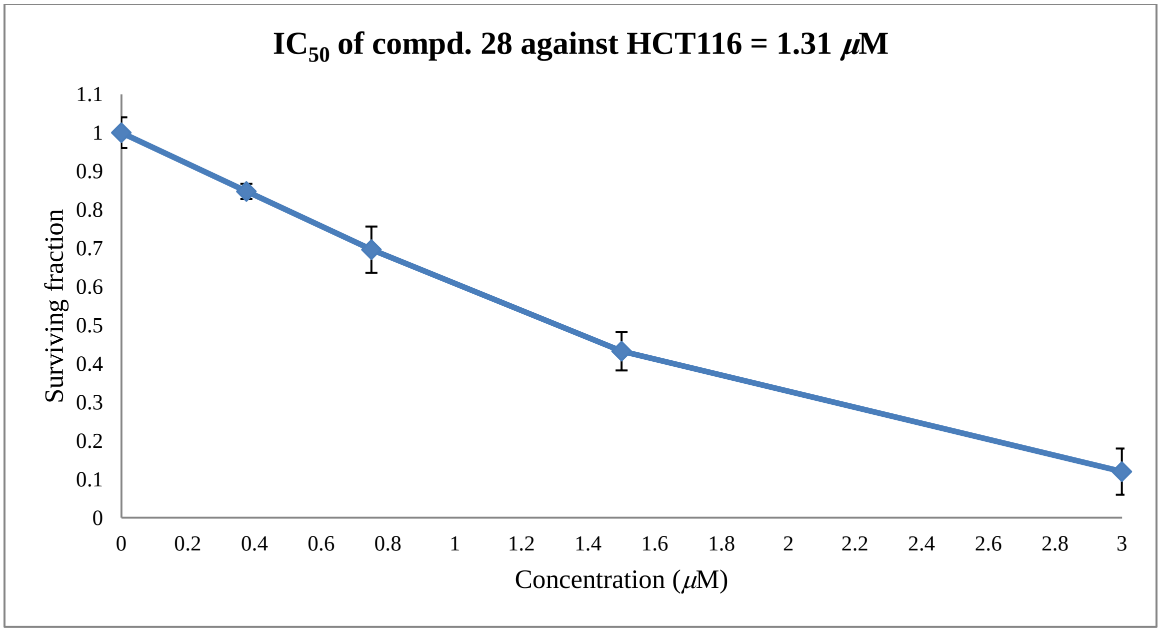

IC50 of compd. 28 against HCT116 = 1.31 μM
Surviving fraction
Concentration (μM)

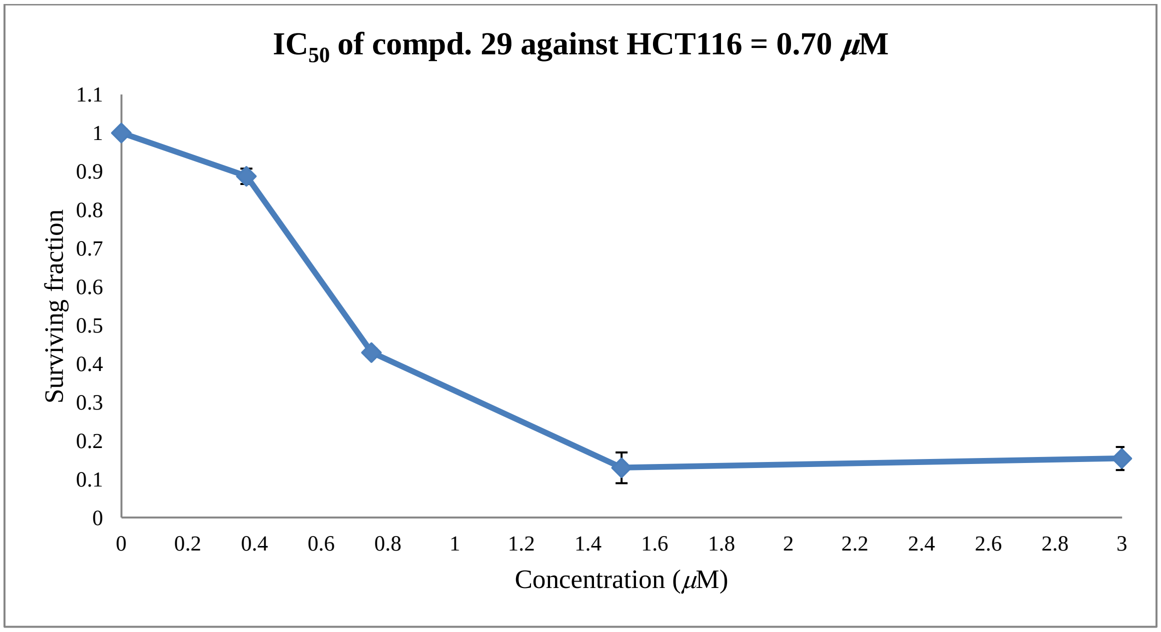

IC50 of compd. 29 against HCT116 = 0.70 μM
Surviving fraction
Concentration (μM)

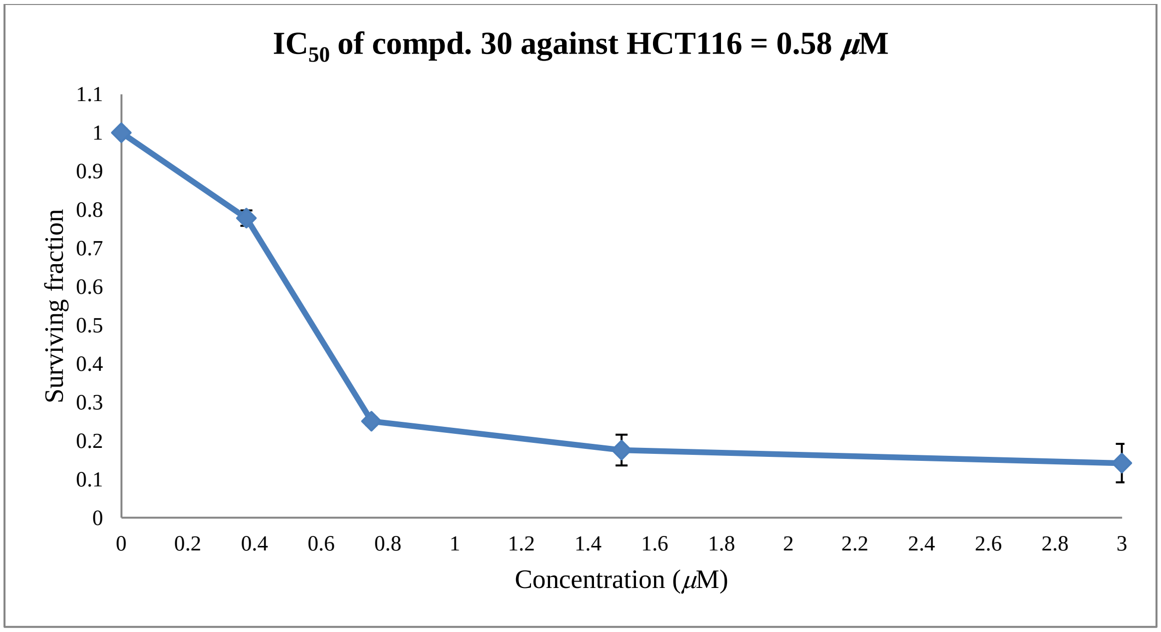

IC$_{50}$ of compd. 30 against HCT116 = 0.58 μM
Surviving fraction
0
0.1
0.2
0.3
0.4
0.5
0.6
0.7
0.8
0.9
1
1.1
0
0.2
0.4
0.6
0.8
1
1.2
1.4
1.6
1.8
2
2.2
2.4
2.6
2.8
3
Concentration (μM)

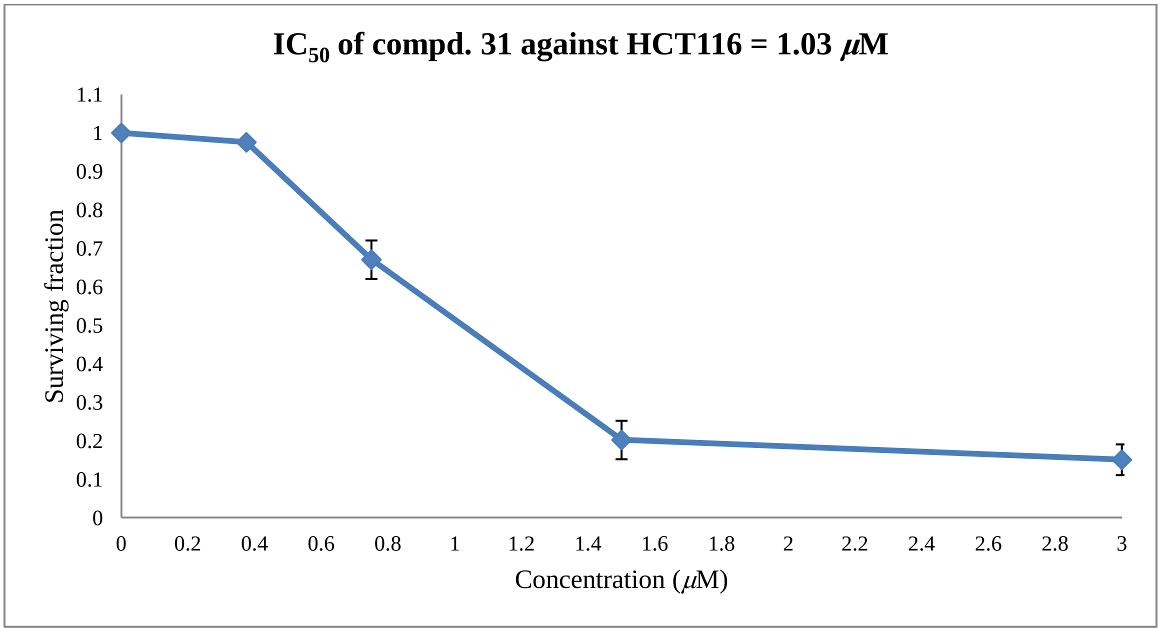

IC$_{50}$ of compd. 31 against HCT116 = 1.03 μM
Surviving fraction
0
0.1
0.2
0.3
0.4
0.5
0.6
0.7
0.8
0.9
1
1.1
0
0.2
0.4
0.6
0.8
1
1.2
1.4
1.6
1.8
2
2.2
2.4
2.6
2.8
3
Concentration (μM)

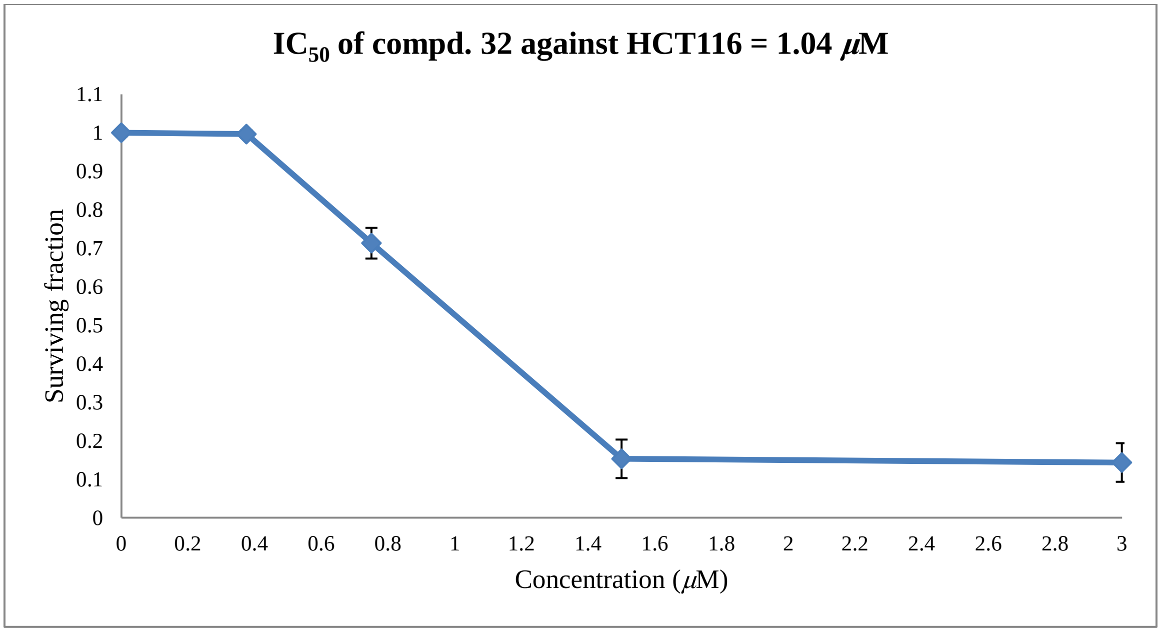
IC50 of compd. 32 against HCT116 = 1.04 μM
Surviving fraction
1.1
1
0.9
0.8
0.7
0.6
0.5
0.4
0.3
0.2
0.1
0
0
0.2
0.4
0.6
0.8
1
1.2
1.4
1.6
1.8
2
2.2
2.4
2.6
2.8
3
Concentration (μM)

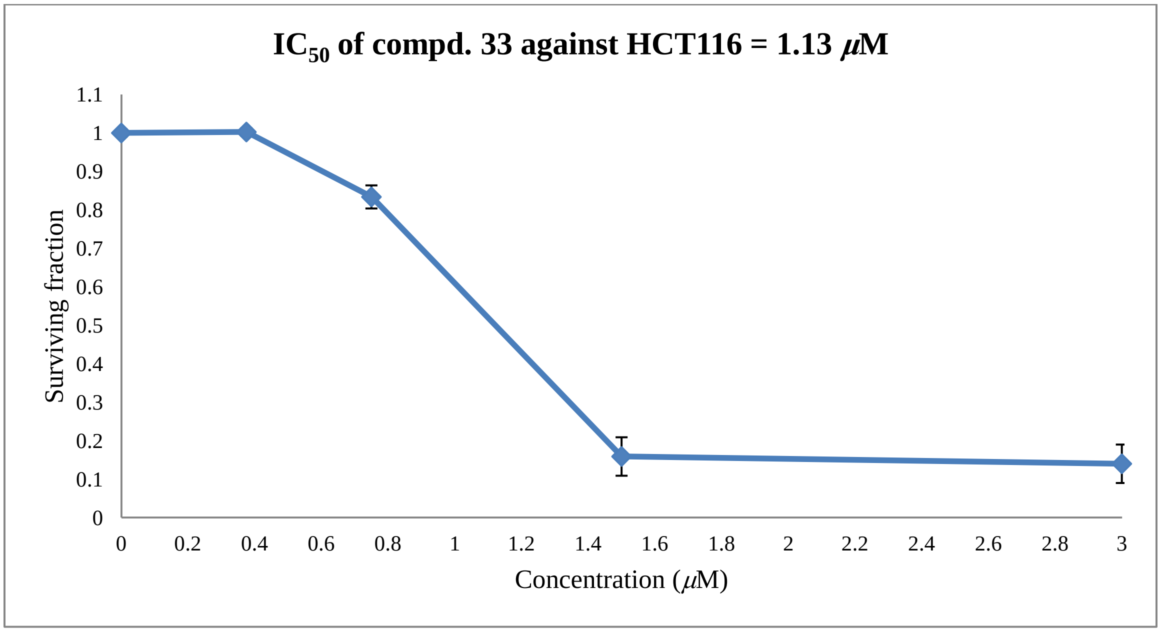
IC50 of compd. 33 against HCT116 = 1.13 μM
Surviving fraction
1.1
1
0.9
0.8
0.7
0.6
0.5
0.4
0.3
0.2
0.1
0
0
0.2
0.4
0.6
0.8
1
1.2
1.4
1.6
1.8
2
2.2
2.4
2.6
2.8
3
Concentration (μM)

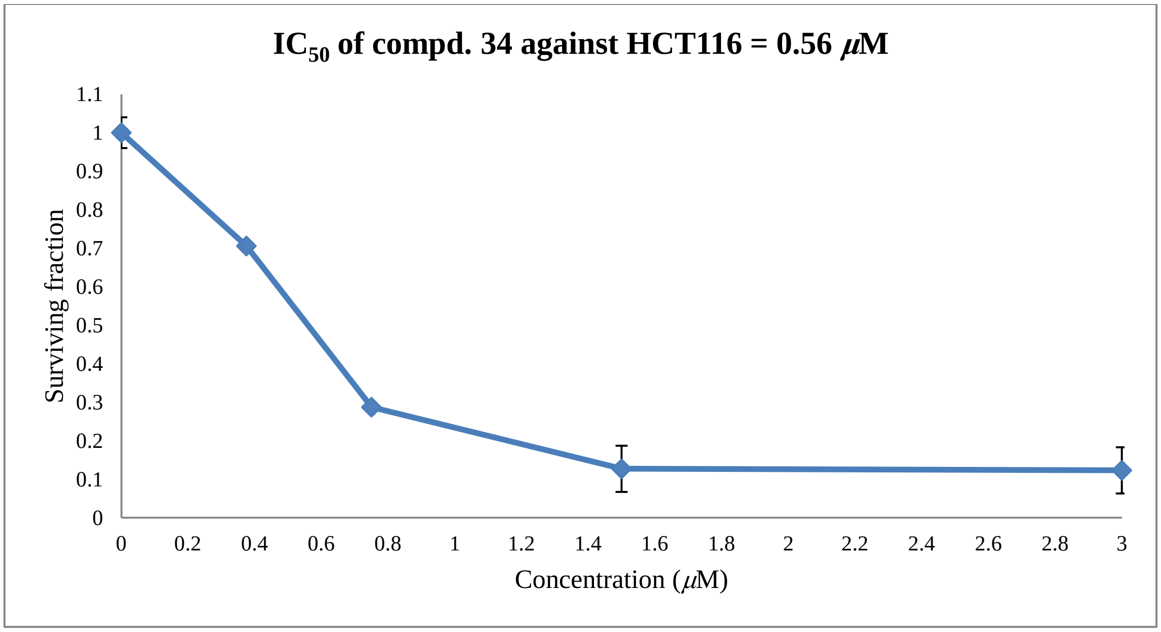
IC50 of compd. 34 against HCT116 = 0.56 μM
1.1
1
0.9
0.8
0.7
0.6
0.5
0.4
0.3
0.2
0.1
0
Surviving fraction
0
0.2
0.4
0.6
0.8
1
1.2
1.4
1.6
1.8
2
2.2
2.4
2.6
2.8
3
Concentration (μM)

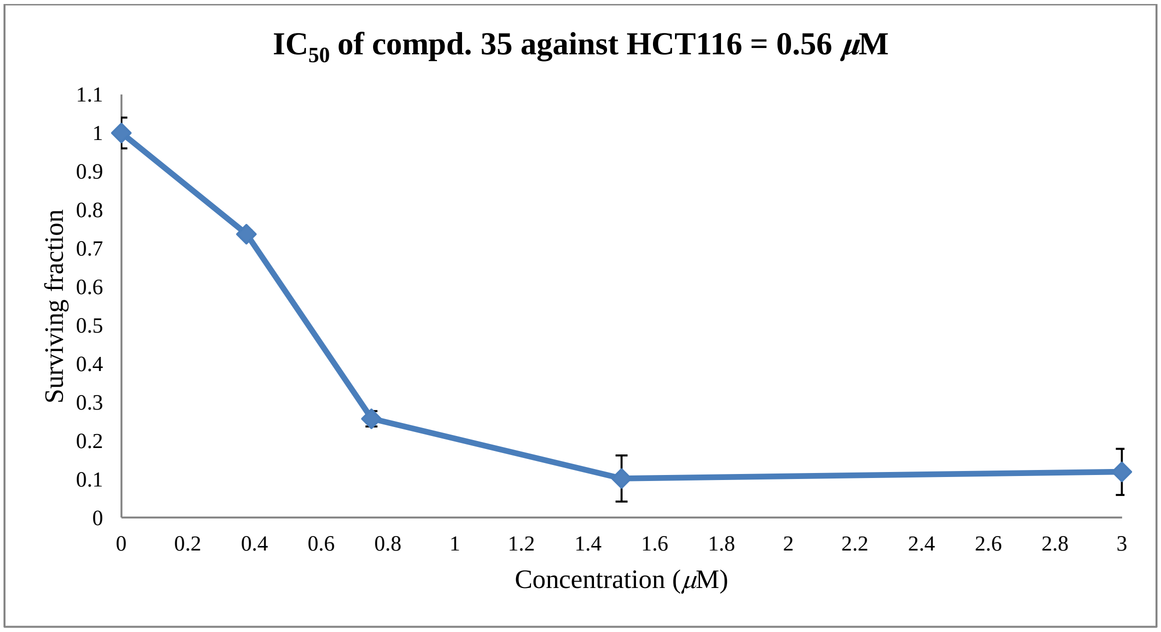
IC50 of compd. 35 against HCT116 = 0.56 μM
1.1
1
0.9
0.8
0.7
0.6
0.5
0.4
0.3
0.2
0.1
0
Surviving fraction
0
0.2
0.4
0.6
0.8
1
1.2
1.4
1.6
1.8
2
2.2
2.4
2.6
2.8
3
Concentration (μM)

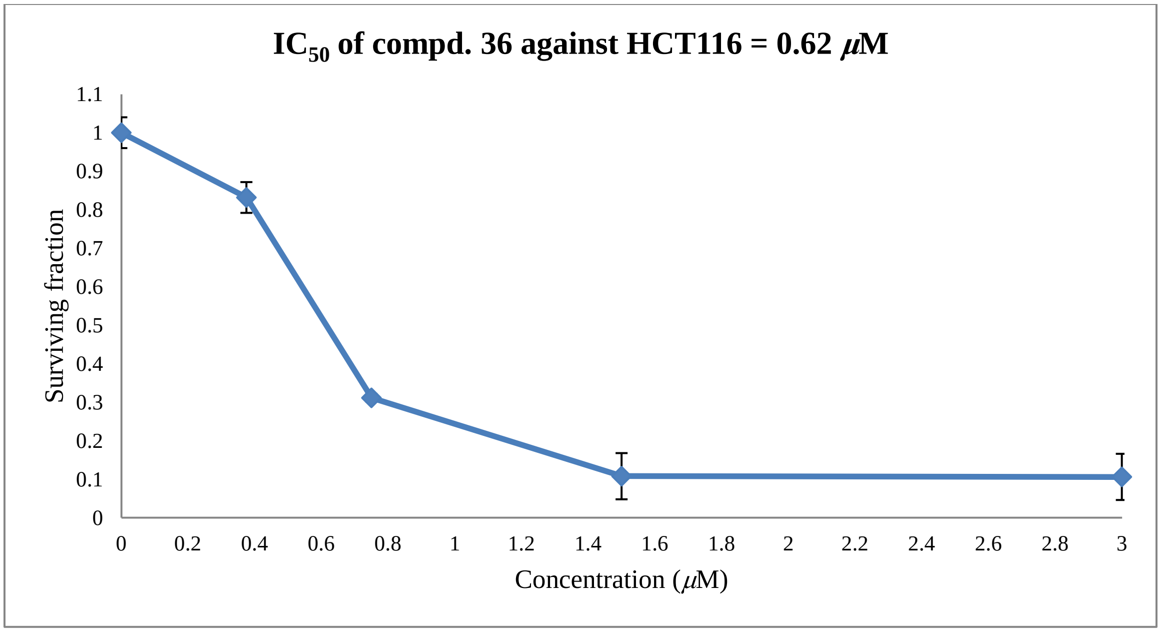
IC50 of compd. 36 against HCT116 = 0.62 μM
Surviving fraction
1.1
1
0.9
0.8
0.7
0.6
0.5
0.4
0.3
0.2
0.1
0
0
0.2
0.4
0.6
0.8
1
1.2
1.4
1.6
1.8
2
2.2
2.4
2.6
2.8
3
Concentration (μM)

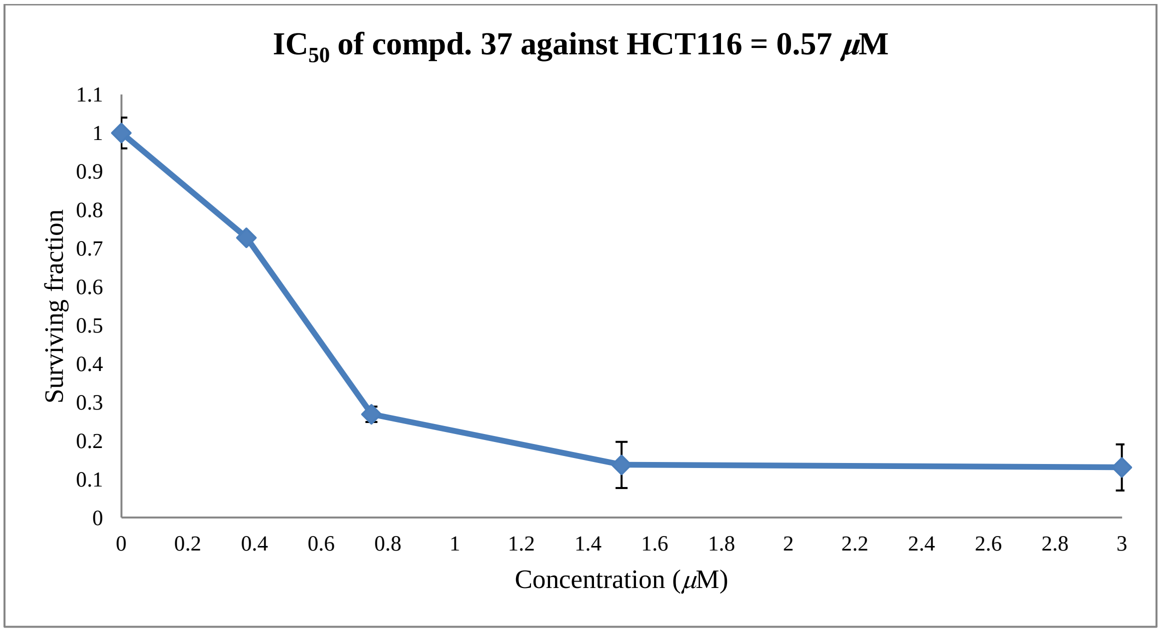
IC50 of compd. 37 against HCT116 = 0.57 μM
Surviving fraction
1.1
1
0.9
0.8
0.7
0.6
0.5
0.4
0.3
0.2
0.1
0
0
0.2
0.4
0.6
0.8
1
1.2
1.4
1.6
1.8
2
2.2
2.4
2.6
2.8
3
Concentration (μM)

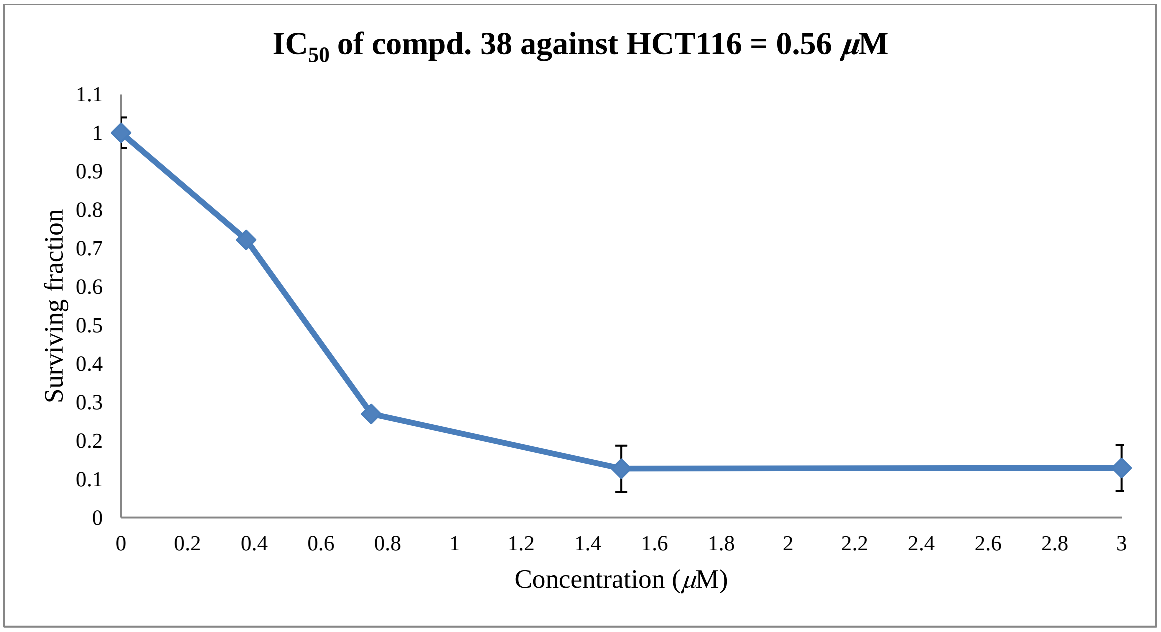
IC50 of compd. 38 against HCT116 = 0.56 μM
Surviving fraction
1.1
1
0.9
0.8
0.7
0.6
0.5
0.4
0.3
0.2
0.1
0
0
0.2
0.4
0.6
0.8
1
1.2
1.4
1.6
1.8
2
2.2
2.4
2.6
2.8
3
Concentration (μM)

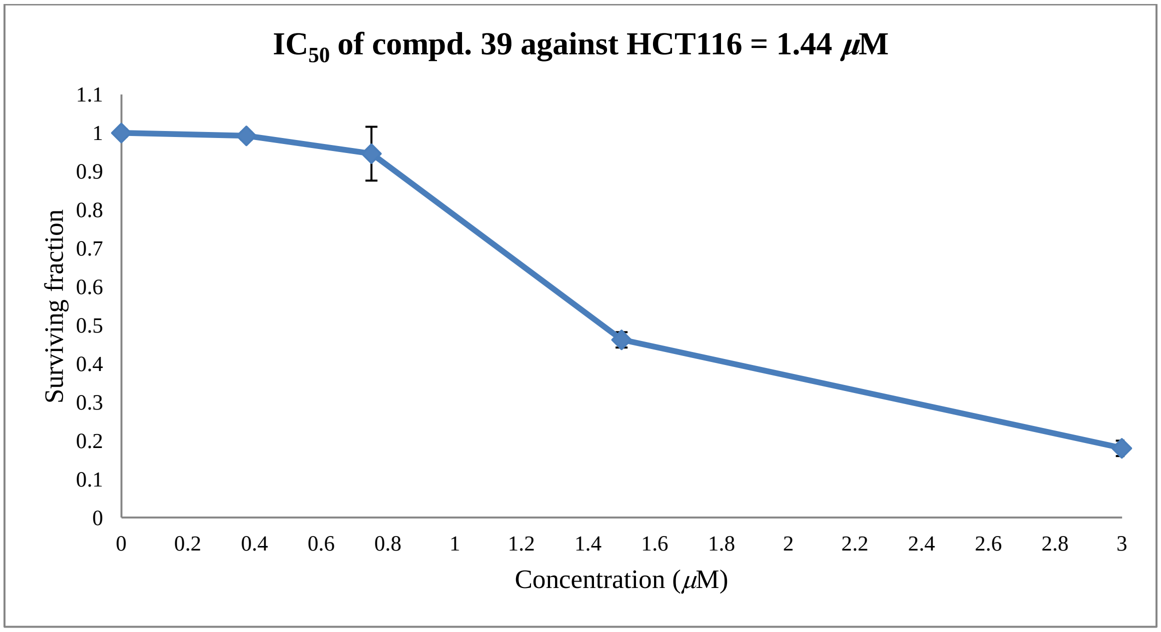
IC50 of compd. 39 against HCT116 = 1.44 μM
Surviving fraction
1.1
1
0.9
0.8
0.7
0.6
0.5
0.4
0.3
0.2
0.1
0
0
0.2
0.4
0.6
0.8
1
1.2
1.4
1.6
1.8
2
2.2
2.4
2.6
2.8
3
Concentration (μM)

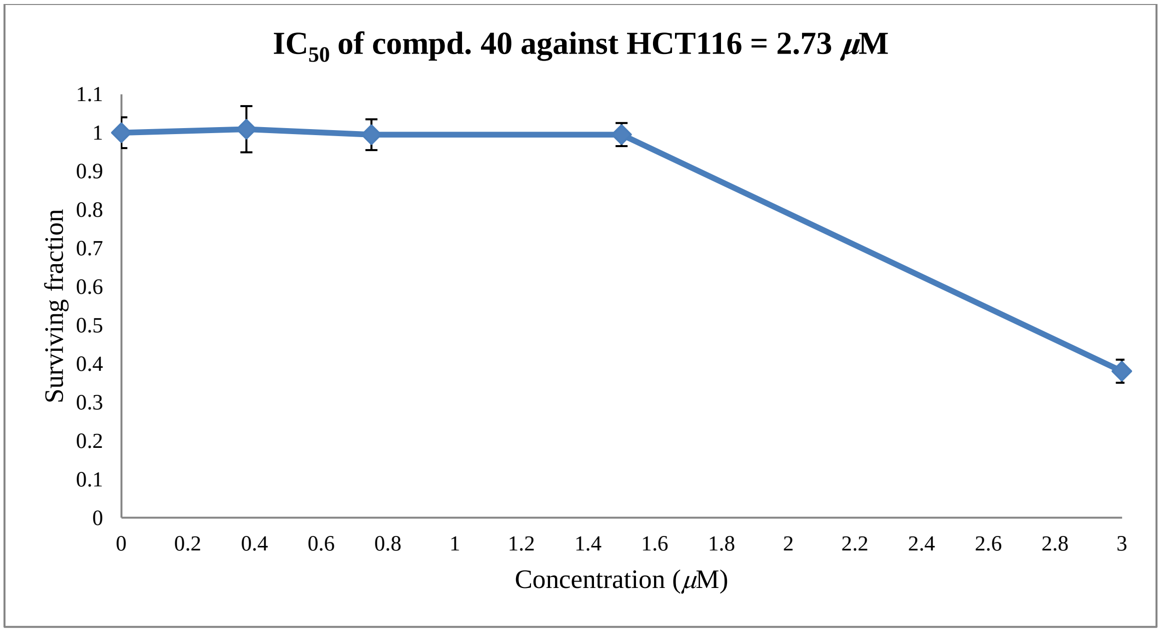

IC50 of compd. 40 against HCT116 = 2.73 μM
Surviving fraction
1.1
1
0.9
0.8
0.7
0.6
0.5
0.4
0.3
0.2
0.1
0
0 0.2 0.4 0.6 0.8 1 1.2 1.4 1.6 1.8 2 2.2 2.4 2.6 2.8 3
Concentration (μM)

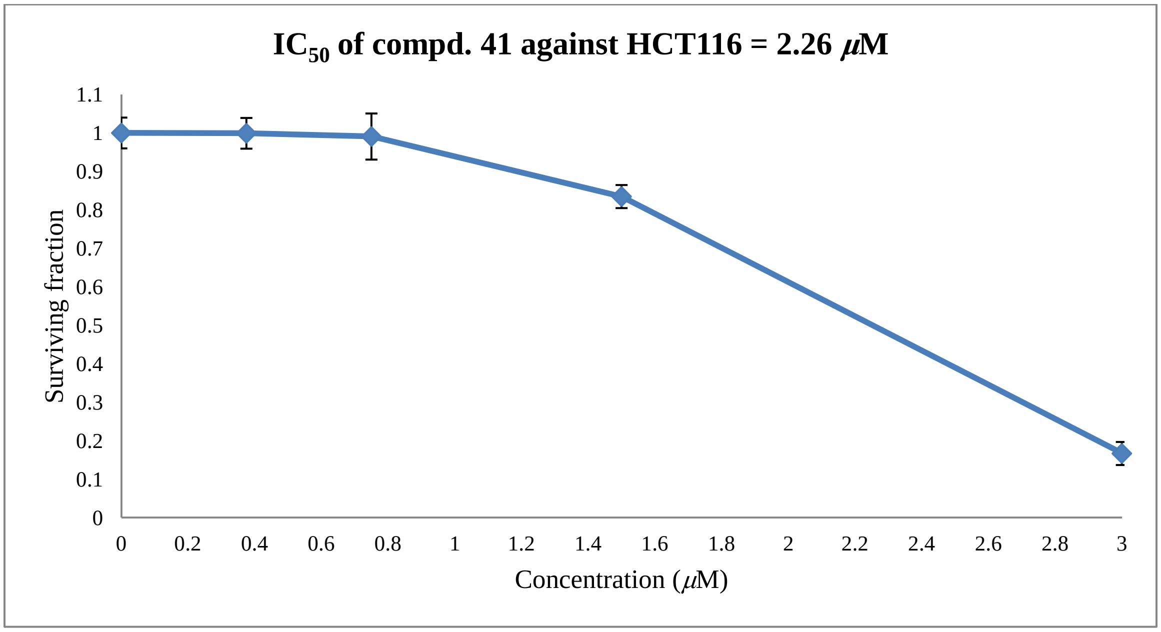

IC50 of compd. 41 against HCT116 = 2.26 μM
Surviving fraction
1.1
1
0.9
0.8
0.7
0.6
0.5
0.4
0.3
0.2
0.1
0
0 0.2 0.4 0.6 0.8 1 1.2 1.4 1.6 1.8 2 2.2 2.4 2.6 2.8 3
Concentration (μM)

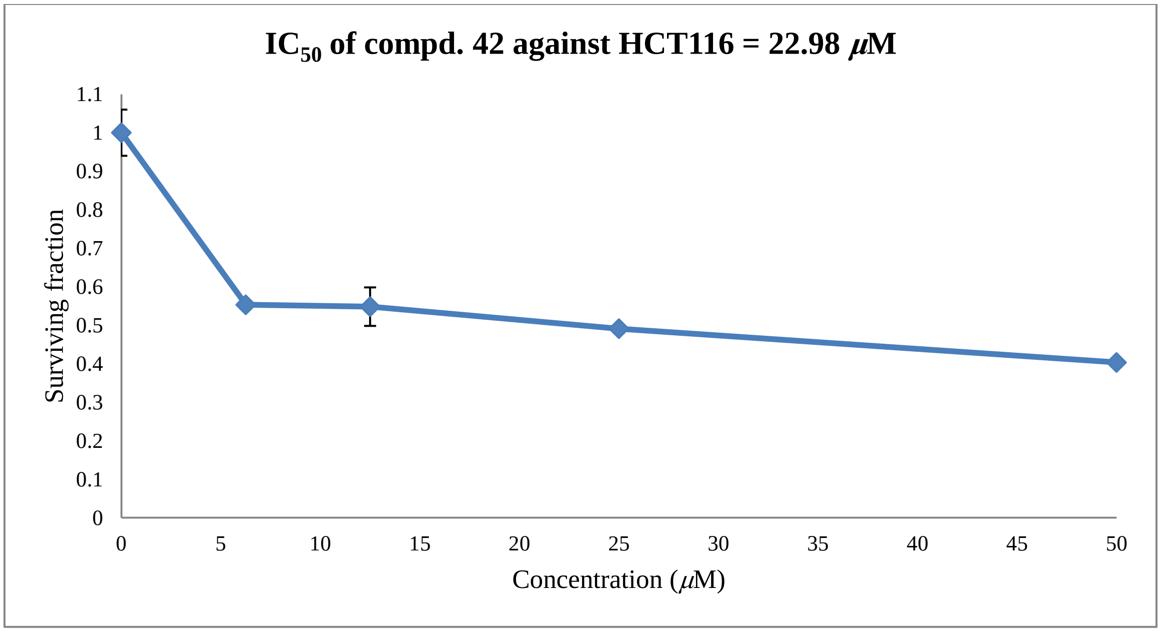
IC50 of compd. 42 against HCT116 = 22.98 μM
Surviving fraction
1.1
1
0.9
0.8
0.7
0.6
0.5
0.4
0.3
0.2
0.1
0
0
5
10
15
20
25
30
35
40
45
50
Concentration (μM)

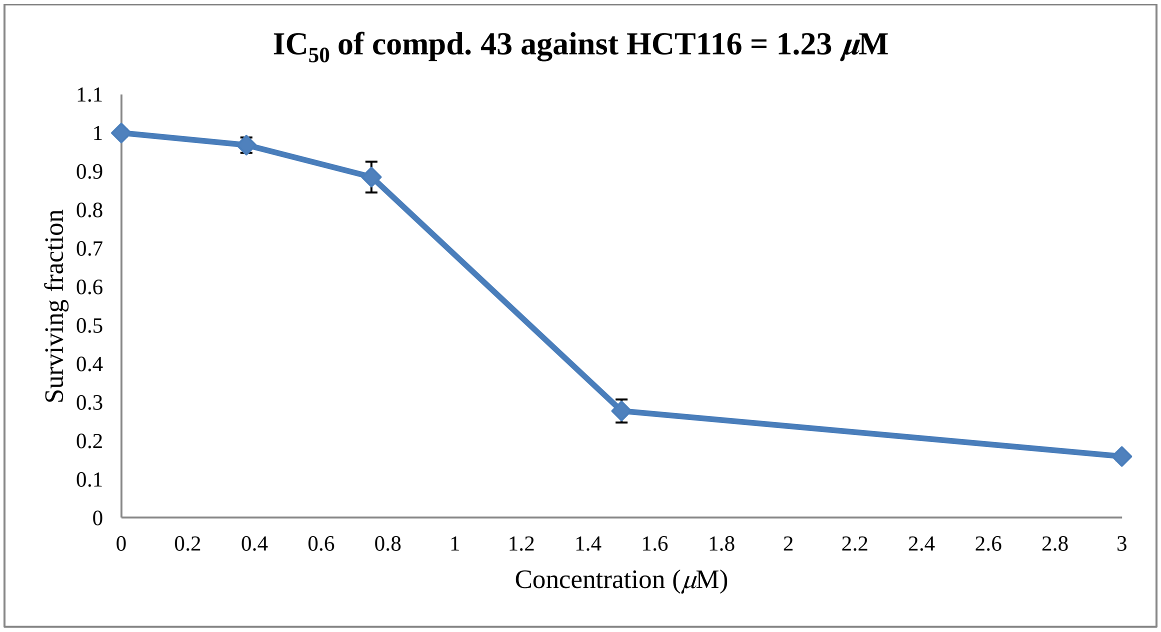
IC50 of compd. 43 against HCT116 = 1.23 μM
Surviving fraction
1.1
1
0.9
0.8
0.7
0.6
0.5
0.4
0.3
0.2
0.1
0
0
0.2
0.4
0.6
0.8
1
1.2
1.4
1.6
1.8
2
2.2
2.4
2.6
2.8
3
Concentration (μM)

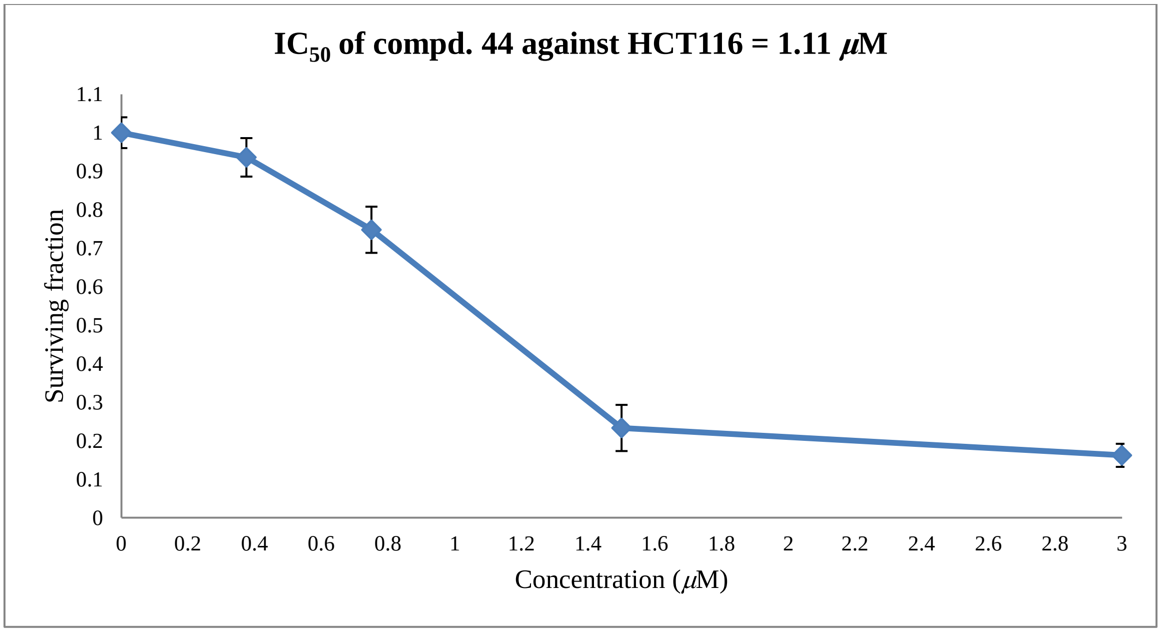

IC50 of compd. 44 against HCT116 = 1.11 μM
Surviving fraction
1.1
1
0.9
0.8
0.7
0.6
0.5
0.4
0.3
0.2
0.1
0
0
0.2
0.4
0.6
0.8
1
1.2
1.4
1.6
1.8
2
2.2
2.4
2.6
2.8
3
Concentration (μM)

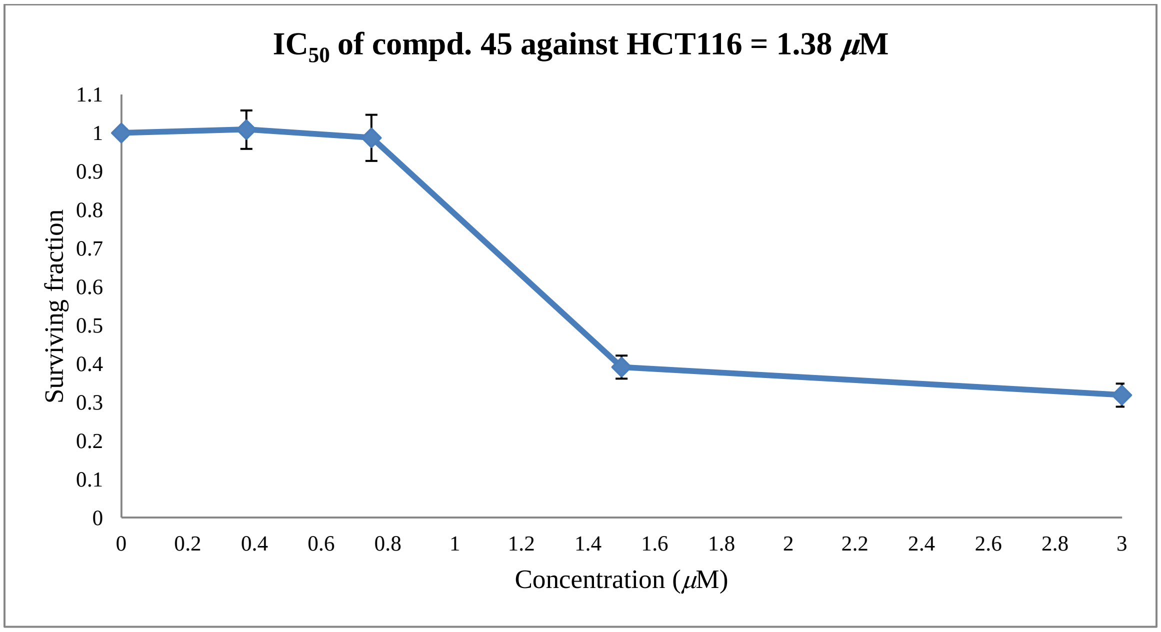

IC50 of compd. 45 against HCT116 = 1.38 μM
Surviving fraction
1.1
1
0.9
0.8
0.7
0.6
0.5
0.4
0.3
0.2
0.1
0
0
0.2
0.4
0.6
0.8
1
1.2
1.4
1.6
1.8
2
2.2
2.4
2.6
2.8
3
Concentration (μM)

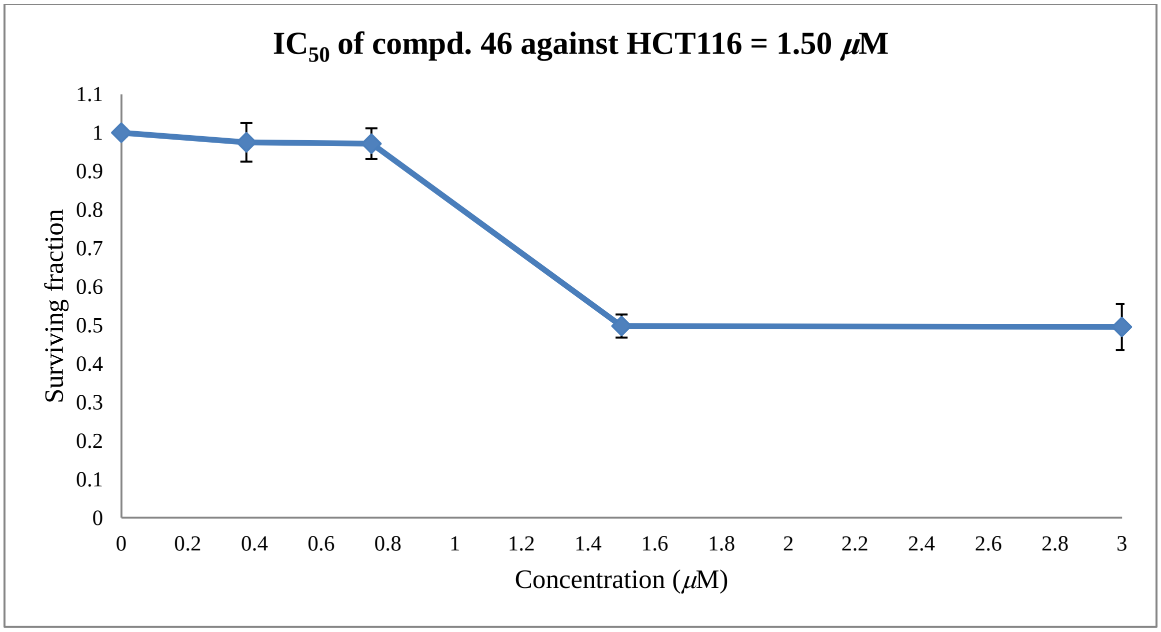
IC50 of compd. 46 against HCT116 = 1.50 μM
Surviving fraction
1.1
1
0.9
0.8
0.7
0.6
0.5
0.4
0.3
0.2
0.1
0
0 0.2 0.4 0.6 0.8 1 1.2 1.4 1.6 1.8 2 2.2 2.4 2.6 2.8 3
Concentration (μM)

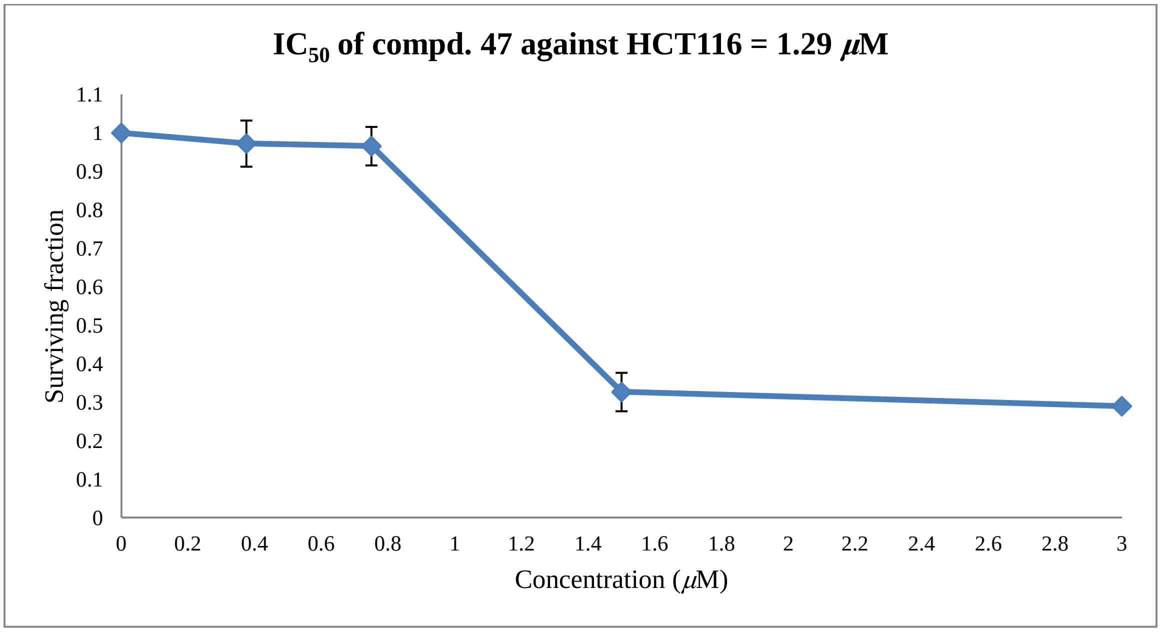
IC50 of compd. 47 against HCT116 = 1.29 μM
Surviving fraction
1.1
1
0.9
0.8
0.7
0.6
0.5
0.4
0.3
0.2
0.1
0
0 0.2 0.4 0.6 0.8 1 1.2 1.4 1.6 1.8 2 2.2 2.4 2.6 2.8 3
Concentration (μM)

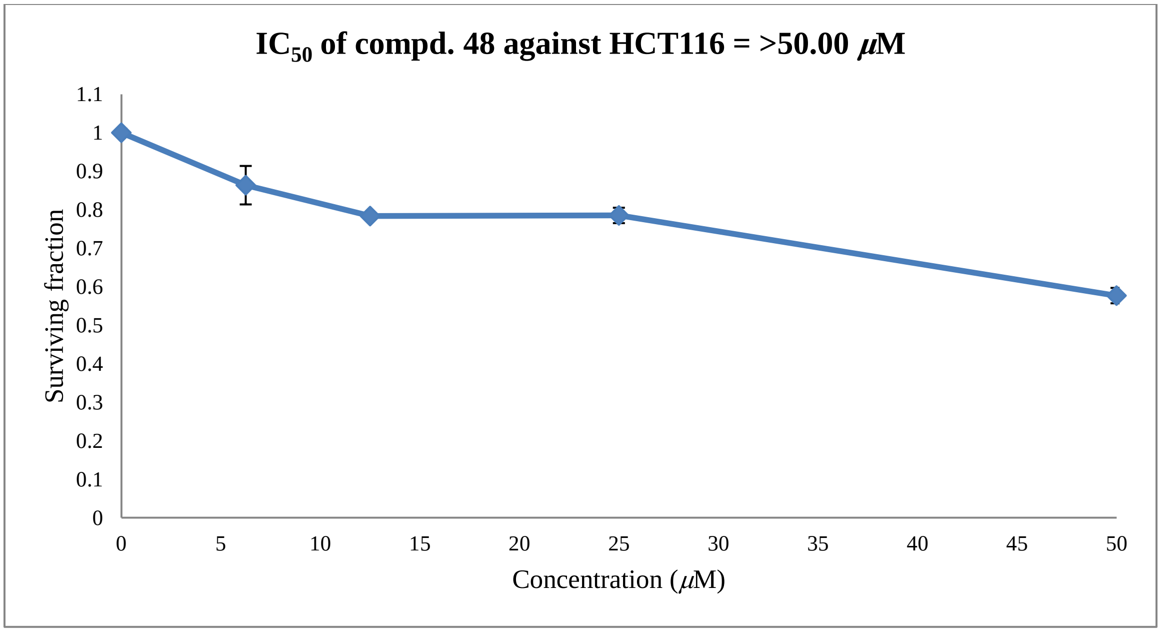
IC$_{50}$ of compd. 48 against HCT116 = >50.00 μM
Surviving fraction
1.1
1
0.9
0.8
0.7
0.6
0.5
0.4
0.3
0.2
0.1
0
0
5
10
15
20
25
30
35
40
45
50
Concentration (μM)

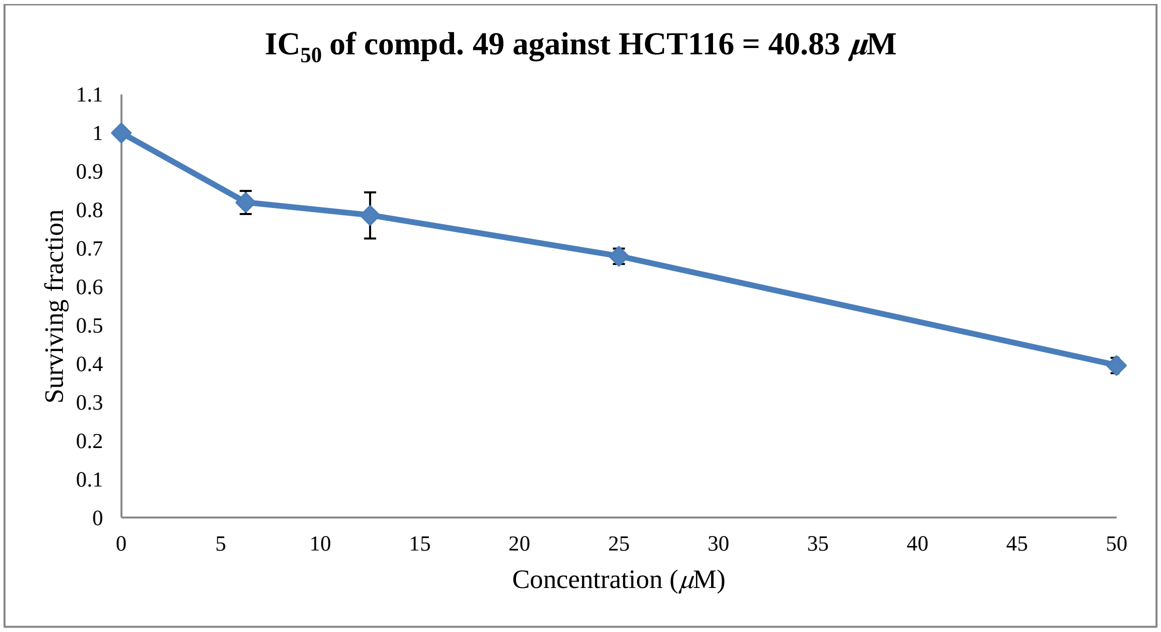
IC$_{50}$ of compd. 49 against HCT116 = 40.83 μM
Surviving fraction
1.1
1
0.9
0.8
0.7
0.6
0.5
0.4
0.3
0.2
0.1
0
0
5
10
15
20
25
30
35
40
45
50
Concentration (μM)

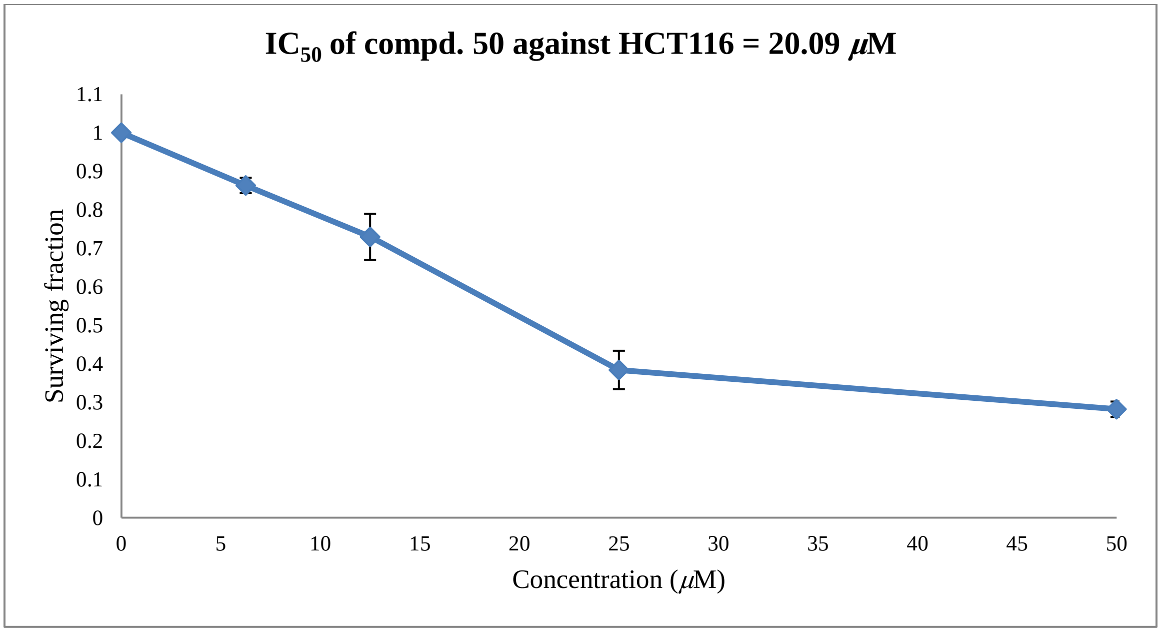
IC50 of compd. 50 against HCT116 = 20.09 μM
Surviving fraction
1.1
1
0.9
0.8
0.7
0.6
0.5
0.4
0.3
0.2
0.1
0
0
5
10
15
20
25
30
35
40
45
50
Concentration (μM)

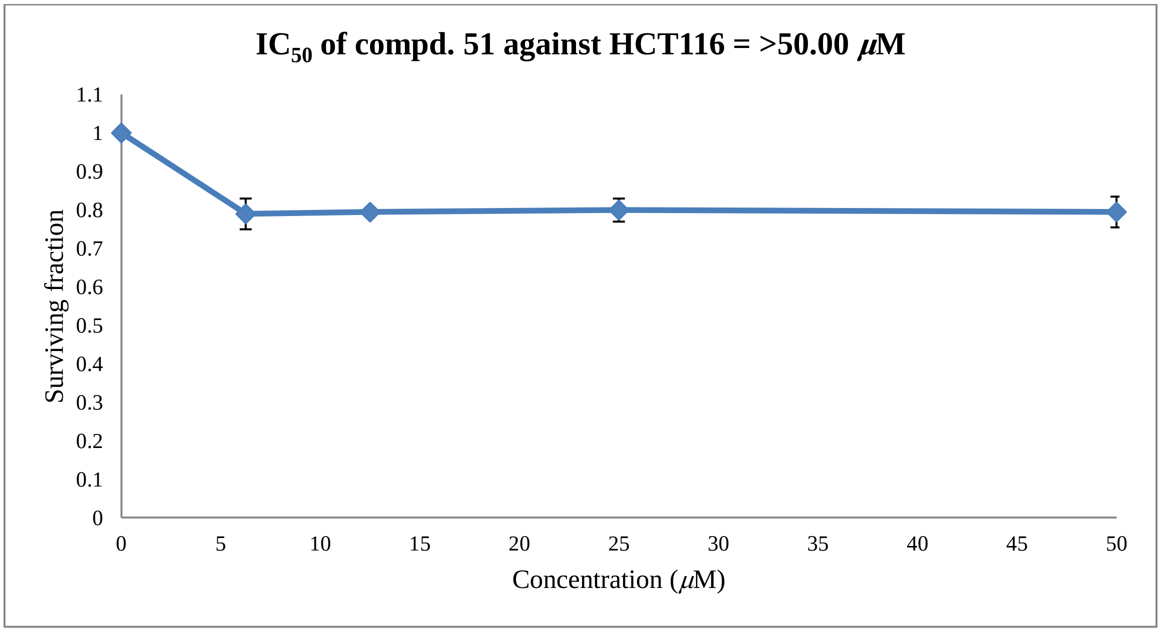
IC50 of compd. 51 against HCT116 = >50.00 μM
Surviving fraction
1.1
1
0.9
0.8
0.7
0.6
0.5
0.4
0.3
0.2
0.1
0
0
5
10
15
20
25
30
35
40
45
50
Concentration (μM)

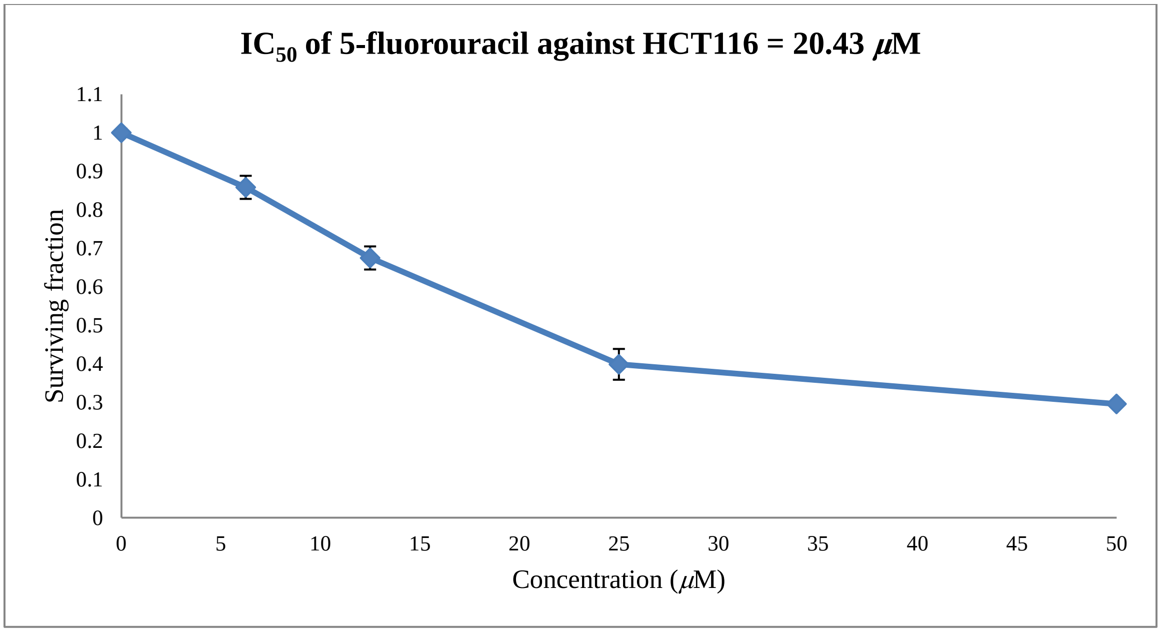

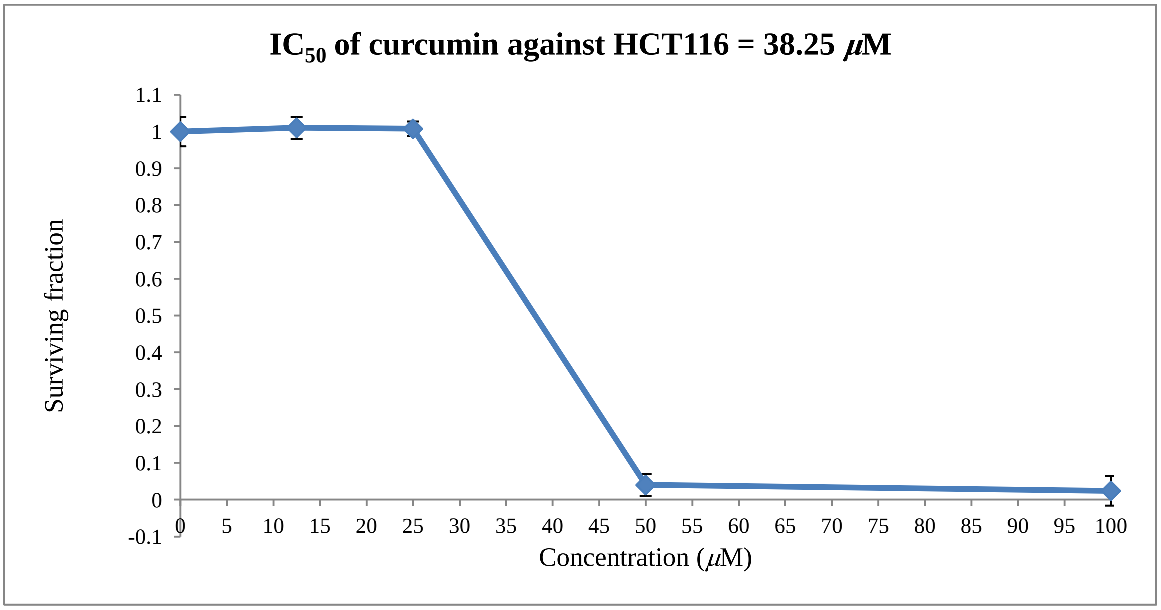

Fig. S93. Dose-response curve for the tested compounds against HCT116 (colon cancer) cell line.

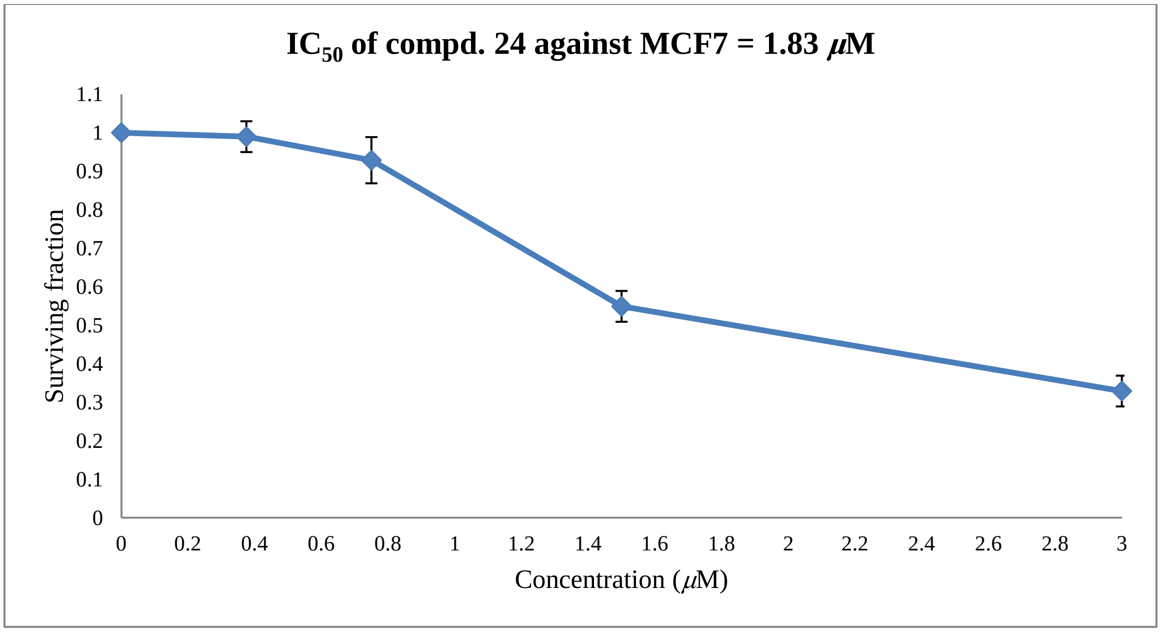
IC50 of compd. 24 against MCF7 = 1.83 μM
Surviving fraction
1.1
1
0.9
0.8
0.7
0.6
0.5
0.4
0.3
0.2
0.1
0
0 0.2 0.4 0.6 0.8 1 1.2 1.4 1.6 1.8 2 2.2 2.4 2.6 2.8 3
Concentration (μM)

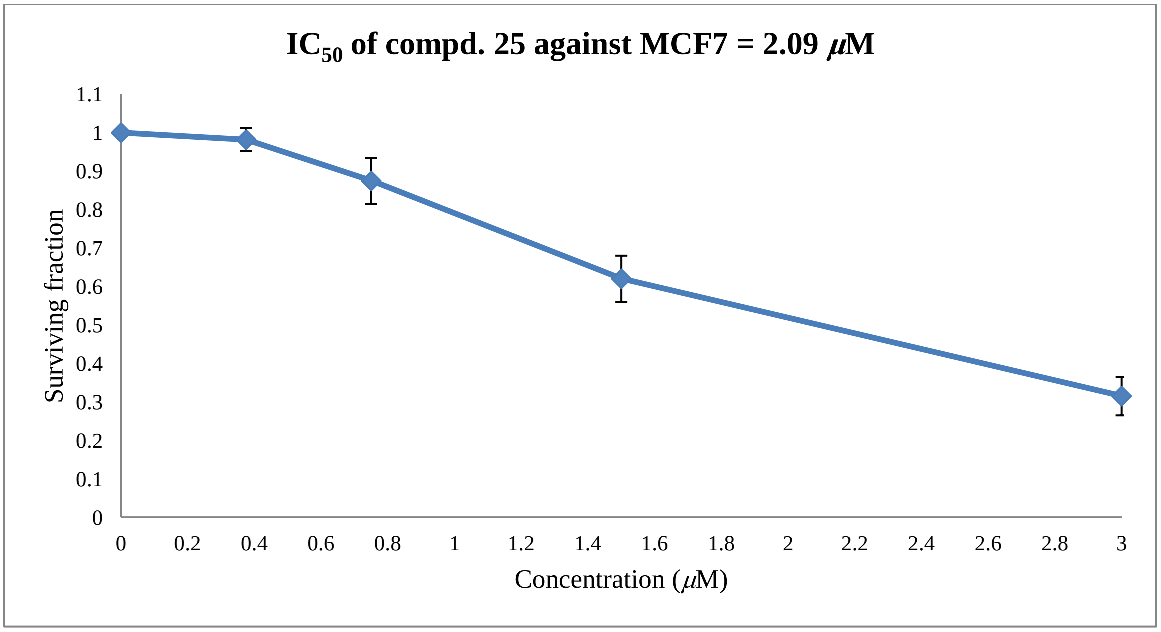
IC50 of compd. 25 against MCF7 = 2.09 μM
Surviving fraction
1.1
1
0.9
0.8
0.7
0.6
0.5
0.4
0.3
0.2
0.1
0
0 0.2 0.4 0.6 0.8 1 1.2 1.4 1.6 1.8 2 2.2 2.4 2.6 2.8 3
Concentration (μM)

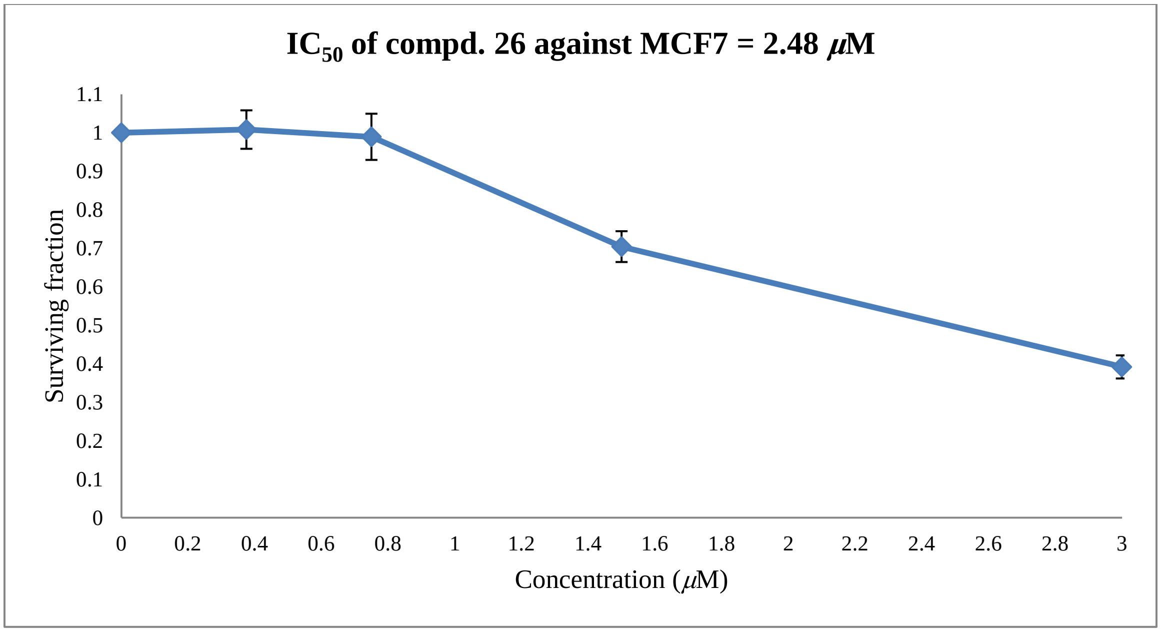
IC50 of compd. 26 against MCF7 = 2.48 μM
Surviving fraction
1.1
1
0.9
0.8
0.7
0.6
0.5
0.4
0.3
0.2
0.1
0
0
0.2
0.4
0.6
0.8
1
1.2
1.4
1.6
1.8
2
2.2
2.4
2.6
2.8
3
Concentration (μM)

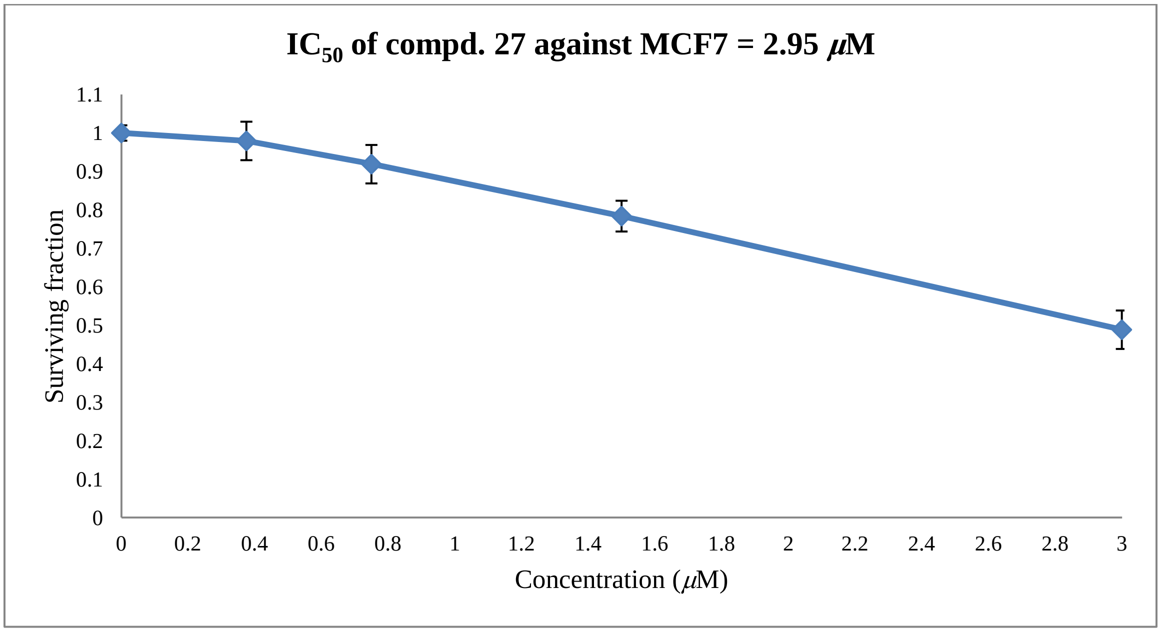
IC50 of compd. 27 against MCF7 = 2.95 μM
Surviving fraction
1.1
1
0.9
0.8
0.7
0.6
0.5
0.4
0.3
0.2
0.1
0
0
0.2
0.4
0.6
0.8
1
1.2
1.4
1.6
1.8
2
2.2
2.4
2.6
2.8
3
Concentration (μM)

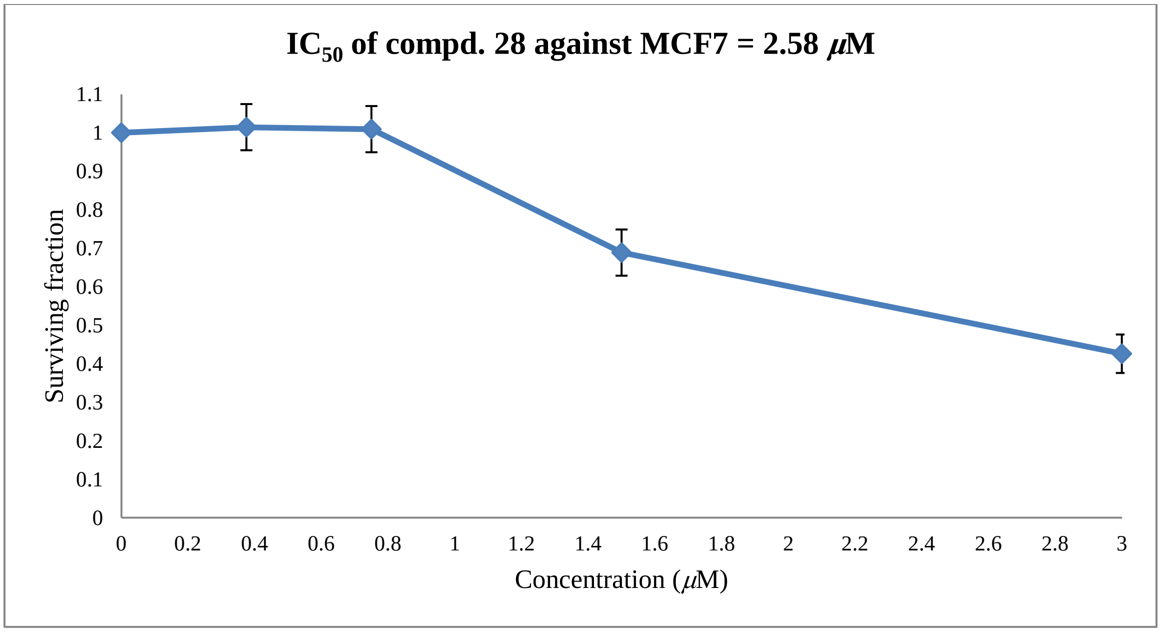
IC50 of compd. 28 against MCF7 = 2.58 μM
Surviving fraction
Concentration (μM)
0
0.1
0.2
0.3
0.4
0.5
0.6
0.7
0.8
0.9
1
1.1
0.2
0.4
0.6
0.8
1.2
1.4
1.6
1.8
2
2.2
2.4
2.6
2.8
3

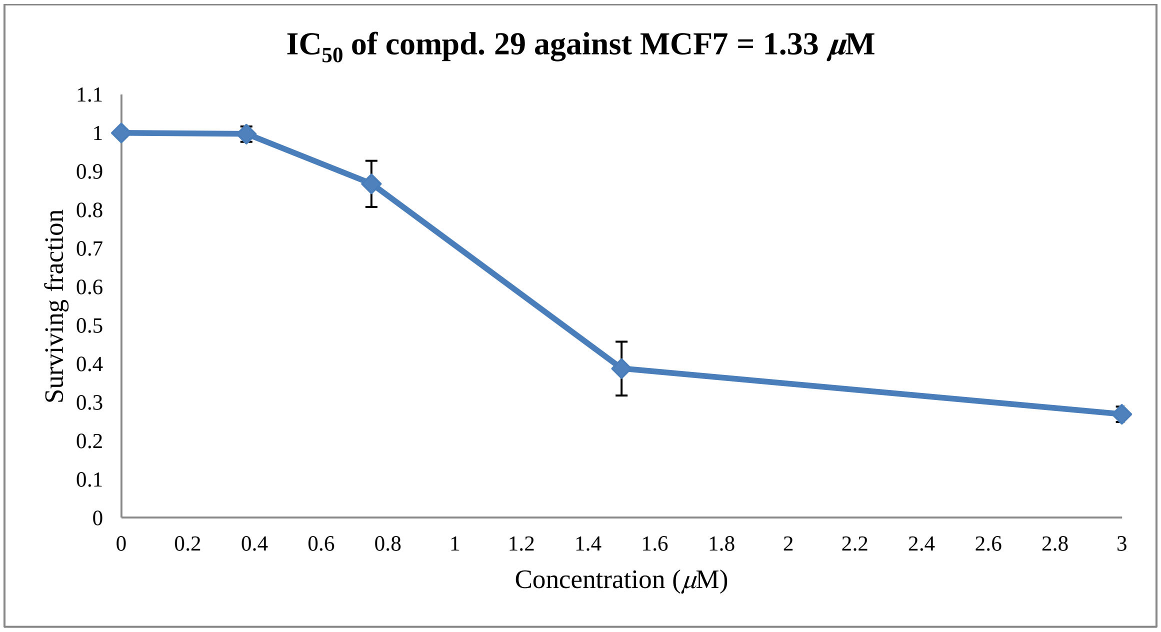
IC50 of compd. 29 against MCF7 = 1.33 μM
Surviving fraction
Concentration (μM)
0
0.1
0.2
0.3
0.4
0.5
0.6
0.7
0.8
0.9
1
1.1
0.2
0.4
0.6
0.8
1.2
1.4
1.6
1.8
2
2.2
2.4
2.6
2.8
3

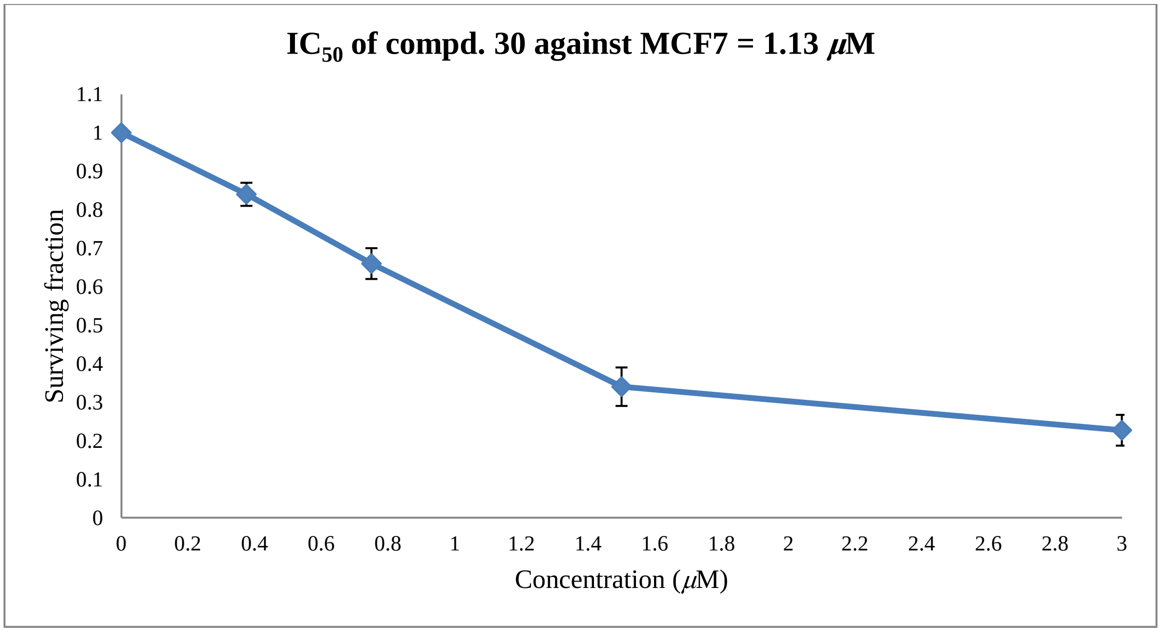
IC50 of compd. 30 against MCF7 = 1.13 μM
Surviving fraction
1.1
1
0.9
0.8
0.7
0.6
0.5
0.4
0.3
0.2
0.1
0
0
0.2
0.4
0.6
0.8
1
1.2
1.4
1.6
1.8
2
2.2
2.4
2.6
2.8
3
Concentration (μM)

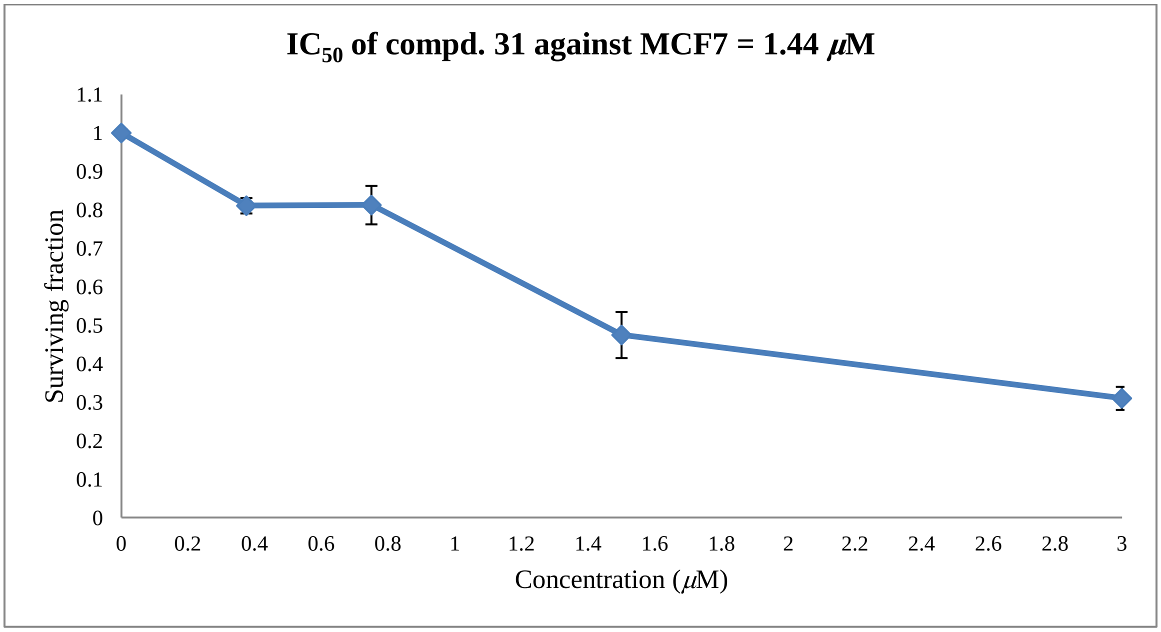
IC50 of compd. 31 against MCF7 = 1.44 μM
Surviving fraction
1.1
1
0.9
0.8
0.7
0.6
0.5
0.4
0.3
0.2
0.1
0
0
0.2
0.4
0.6
0.8
1
1.2
1.4
1.6
1.8
2
2.2
2.4
2.6
2.8
3
Concentration (μM)

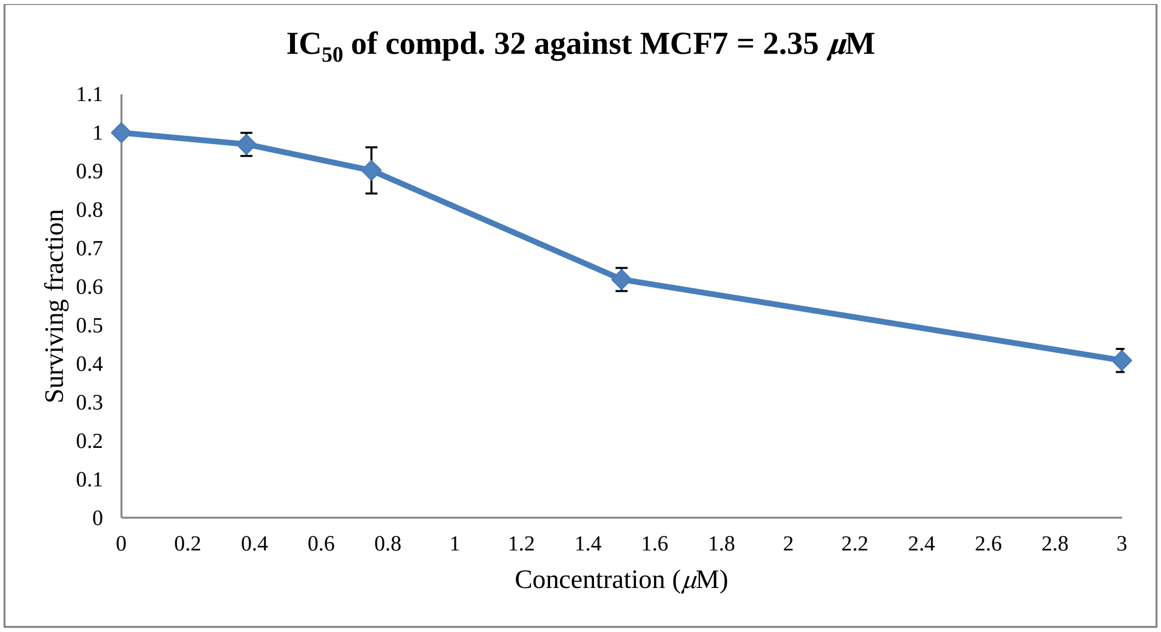
IC50 of compd. 32 against MCF7 = 2.35 μM
Surviving fraction
Concentration (μM)
1.1
1
0.9
0.8
0.7
0.6
0.5
0.4
0.3
0.2
0.1
0
0
0.2
0.4
0.6
0.8
1
1.2
1.4
1.6
1.8
2
2.2
2.4
2.6
2.8
3

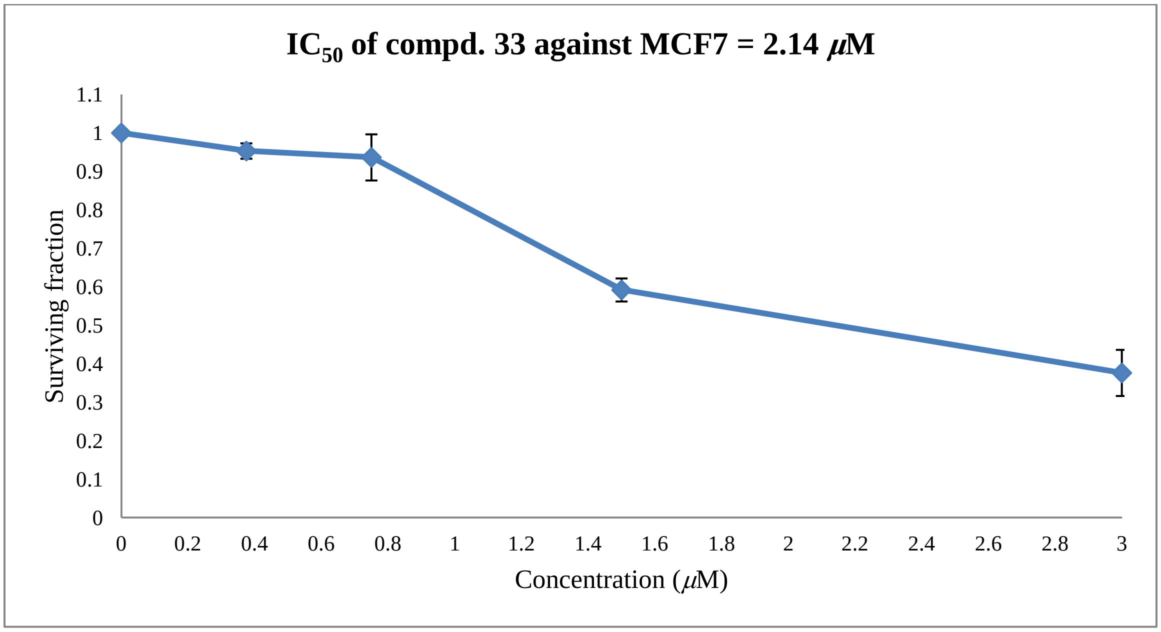
IC50 of compd. 33 against MCF7 = 2.14 μM
Surviving fraction
Concentration (μM)
1.1
1
0.9
0.8
0.7
0.6
0.5
0.4
0.3
0.2
0.1
0
0
0.2
0.4
0.6
0.8
1
1.2
1.4
1.6
1.8
2
2.2
2.4
2.6
2.8
3

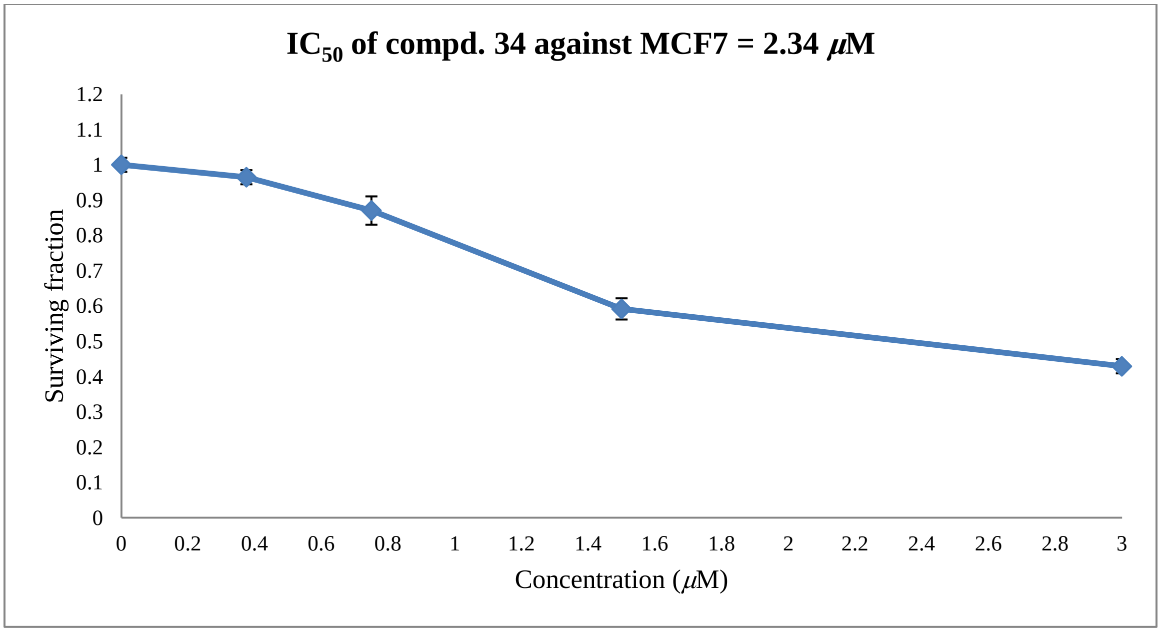
IC50 of compd. 34 against MCF7 = 2.34 μM
Surviving fraction
Concentration (μM)
1.2
1.1
1
0.9
0.8
0.7
0.6
0.5
0.4
0.3
0.2
0.1
0
0
0.2
0.4
0.6
0.8
1
1.2
1.4
1.6
1.8
2
2.2
2.4
2.6
2.8
3

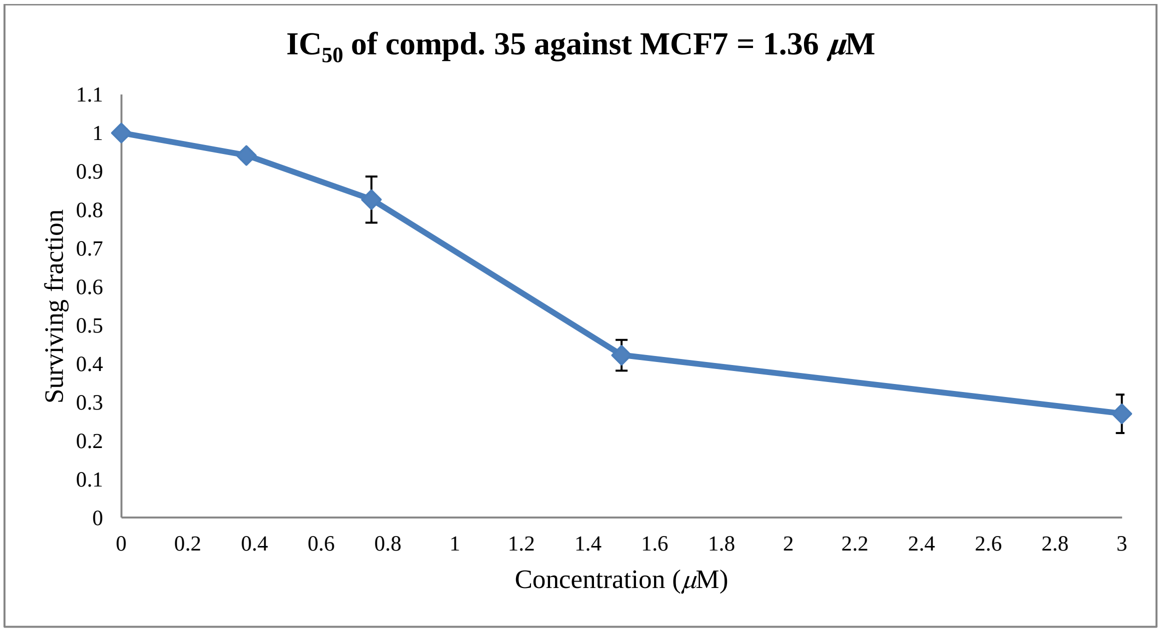
IC50 of compd. 35 against MCF7 = 1.36 μM
Surviving fraction
Concentration (μM)
1.1
1
0.9
0.8
0.7
0.6
0.5
0.4
0.3
0.2
0.1
0
0
0.2
0.4
0.6
0.8
1
1.2
1.4
1.6
1.8
2
2.2
2.4
2.6
2.8
3

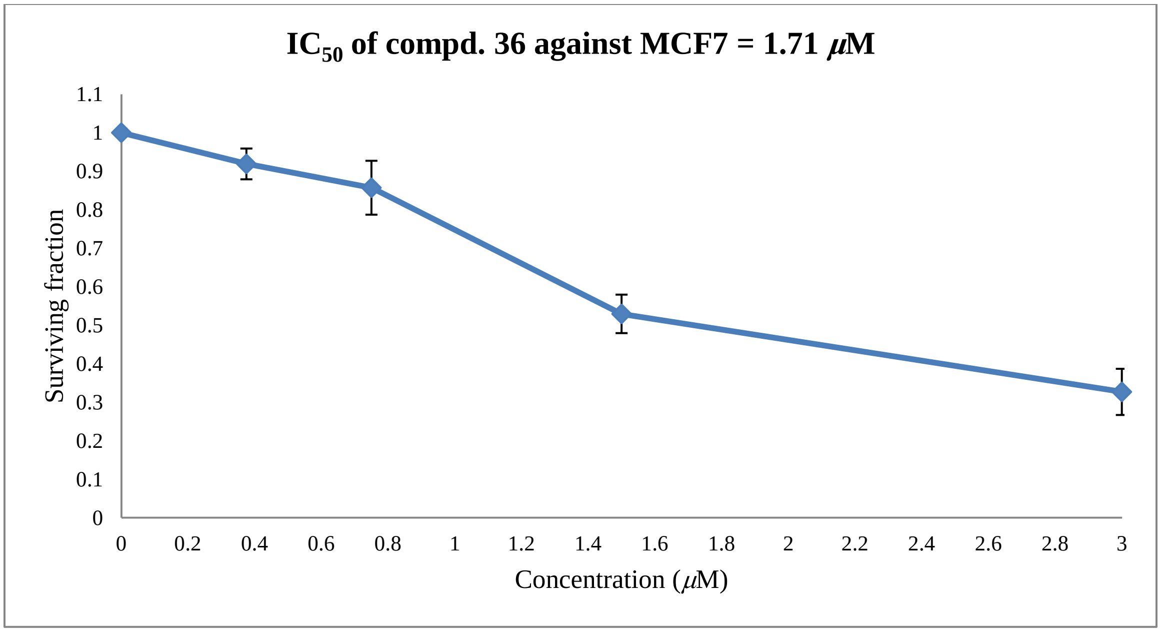
IC50 of compd. 36 against MCF7 = 1.71 μM
Surviving fraction
1.1
1
0.9
0.8
0.7
0.6
0.5
0.4
0.3
0.2
0.1
0
0
0.2
0.4
0.6
0.8
1
1.2
1.4
1.6
1.8
2
2.2
2.4
2.6
2.8
3
Concentration (μM)

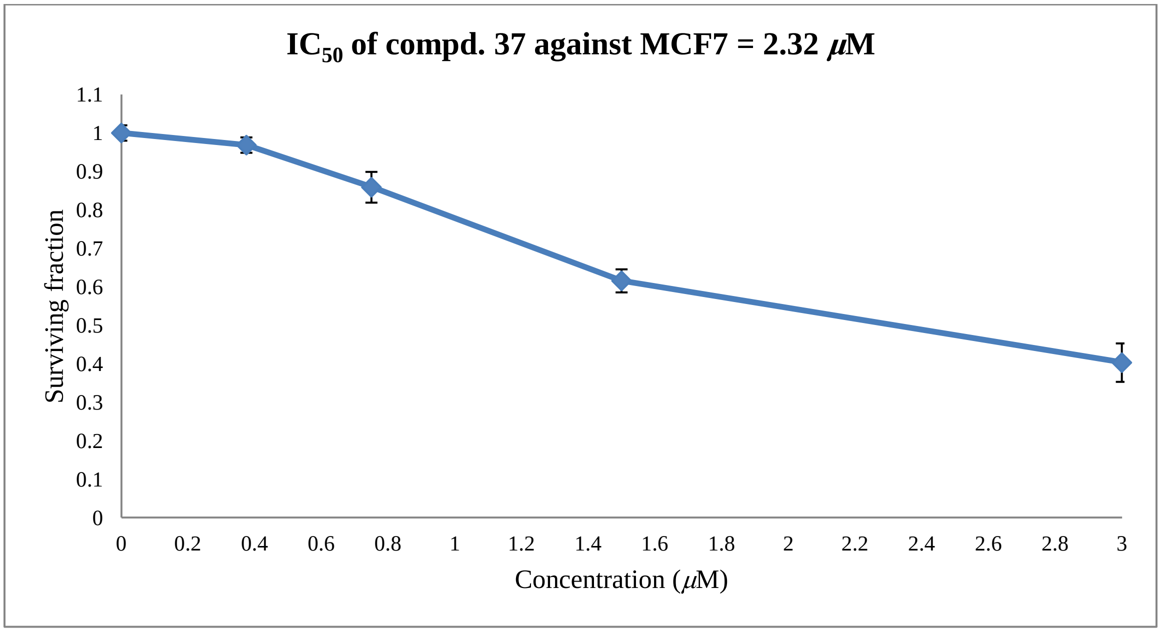
IC50 of compd. 37 against MCF7 = 2.32 μM
Surviving fraction
1.1
1
0.9
0.8
0.7
0.6
0.5
0.4
0.3
0.2
0.1
0
0
0.2
0.4
0.6
0.8
1
1.2
1.4
1.6
1.8
2
2.2
2.4
2.6
2.8
3
Concentration (μM)

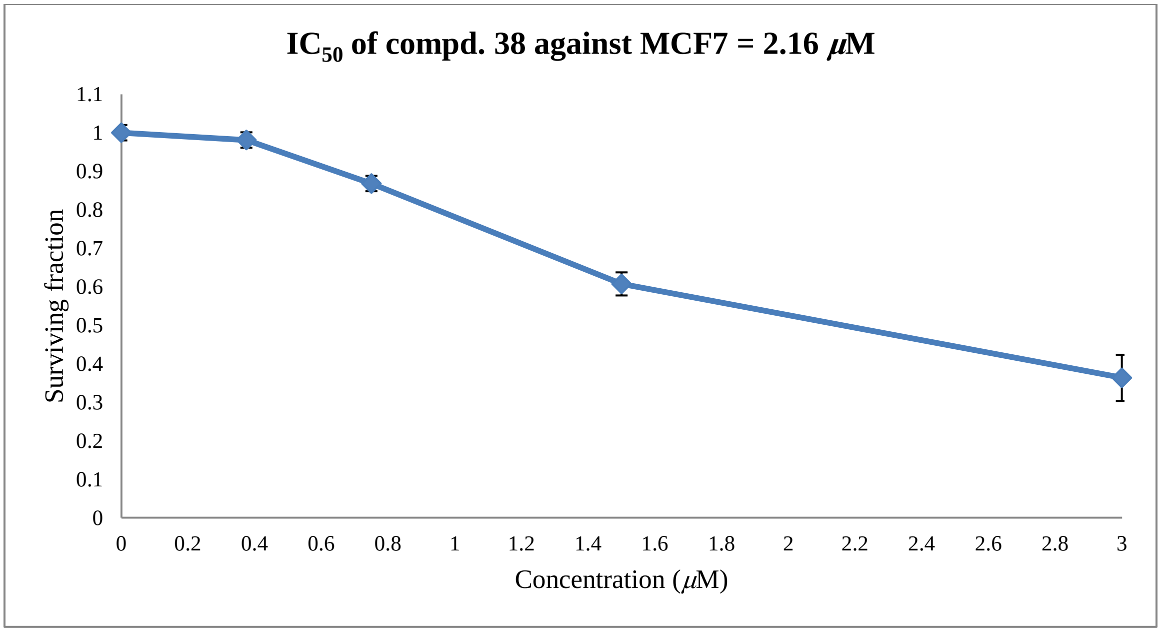

IC50 of compd. 38 against MCF7 = 2.16 μM
Surviving fraction
1.1
1
0.9
0.8
0.7
0.6
0.5
0.4
0.3
0.2
0.1
0
0
0.2
0.4
0.6
0.8
1
1.2
1.4
1.6
1.8
2
2.2
2.4
2.6
2.8
3
Concentration (μM)

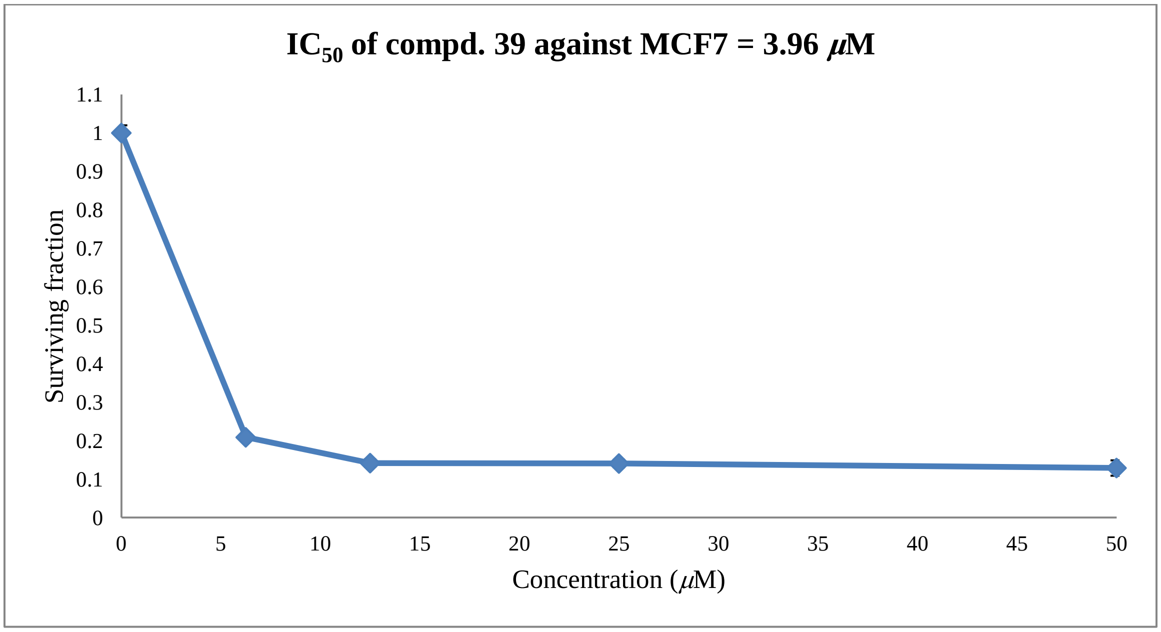

IC50 of compd. 39 against MCF7 = 3.96 μM
Surviving fraction
1.1
1
0.9
0.8
0.7
0.6
0.5
0.4
0.3
0.2
0.1
0
0
5
10
15
20
25
30
35
40
45
50
Concentration (μM)

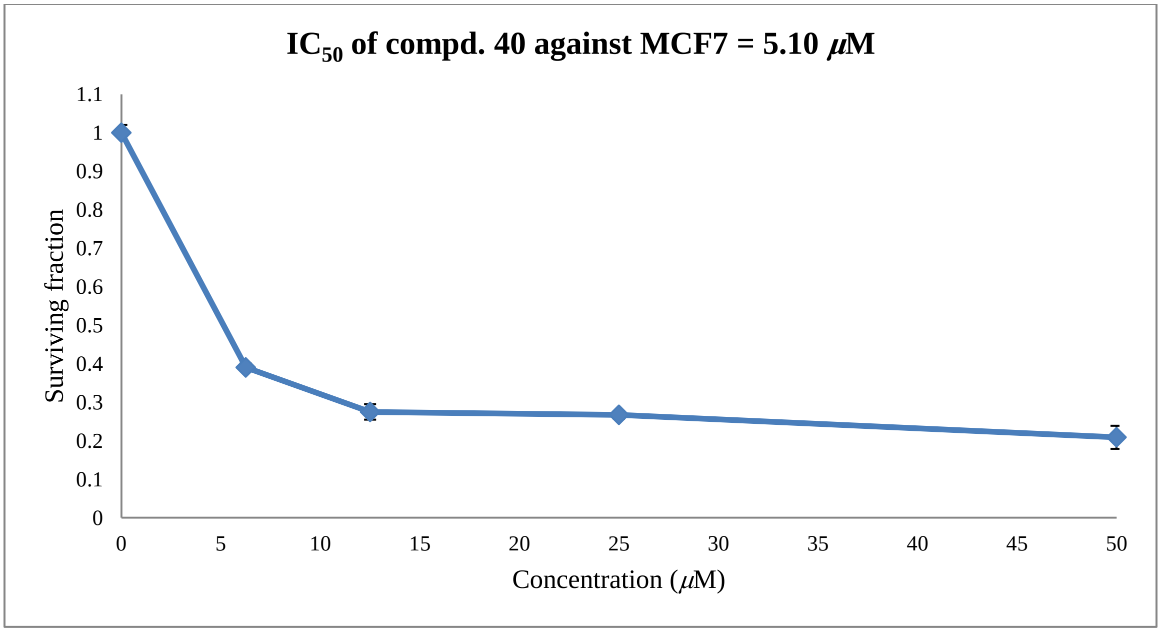
IC50 of compd. 40 against MCF7 = 5.10 μM
Surviving fraction
1.1
1
0.9
0.8
0.7
0.6
0.5
0.4
0.3
0.2
0.1
0
0
5
10
15
20
25
30
35
40
45
50
Concentration (μM)

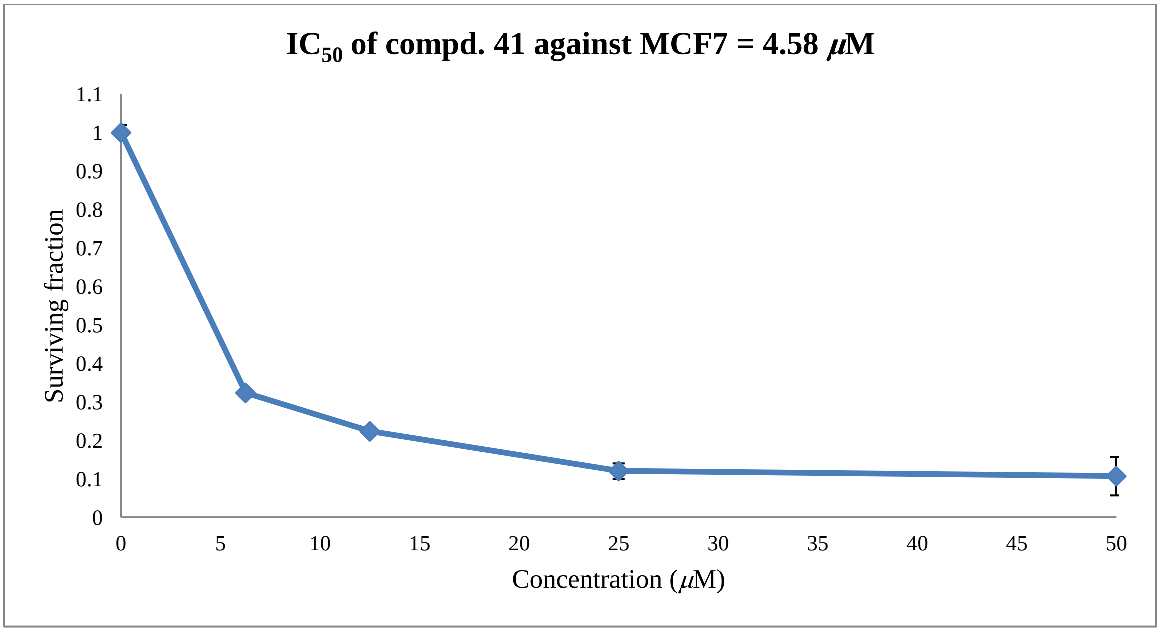
IC50 of compd. 41 against MCF7 = 4.58 μM
Surviving fraction
1.1
1
0.9
0.8
0.7
0.6
0.5
0.4
0.3
0.2
0.1
0
0
5
10
15
20
25
30
35
40
45
50
Concentration (μM)

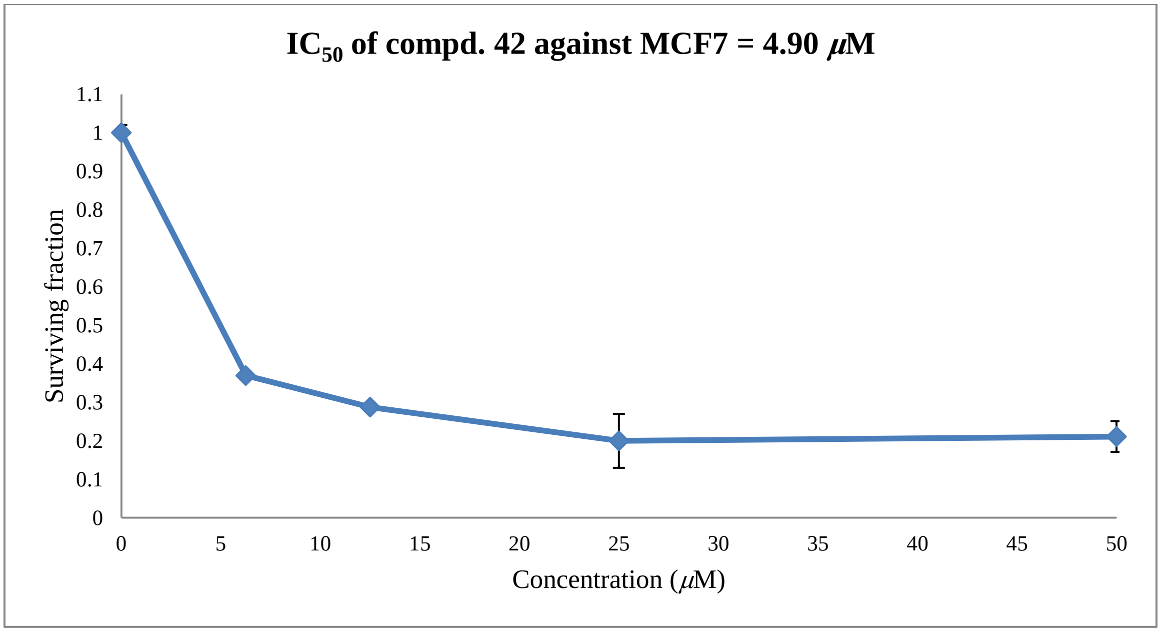
IC50 of compd. 42 against MCF7 = 4.90 μM
Surviving fraction
1.1
1
0.9
0.8
0.7
0.6
0.5
0.4
0.3
0.2
0.1
0
0
5
10
15
20
25
30
35
40
45
50
Concentration (μM)

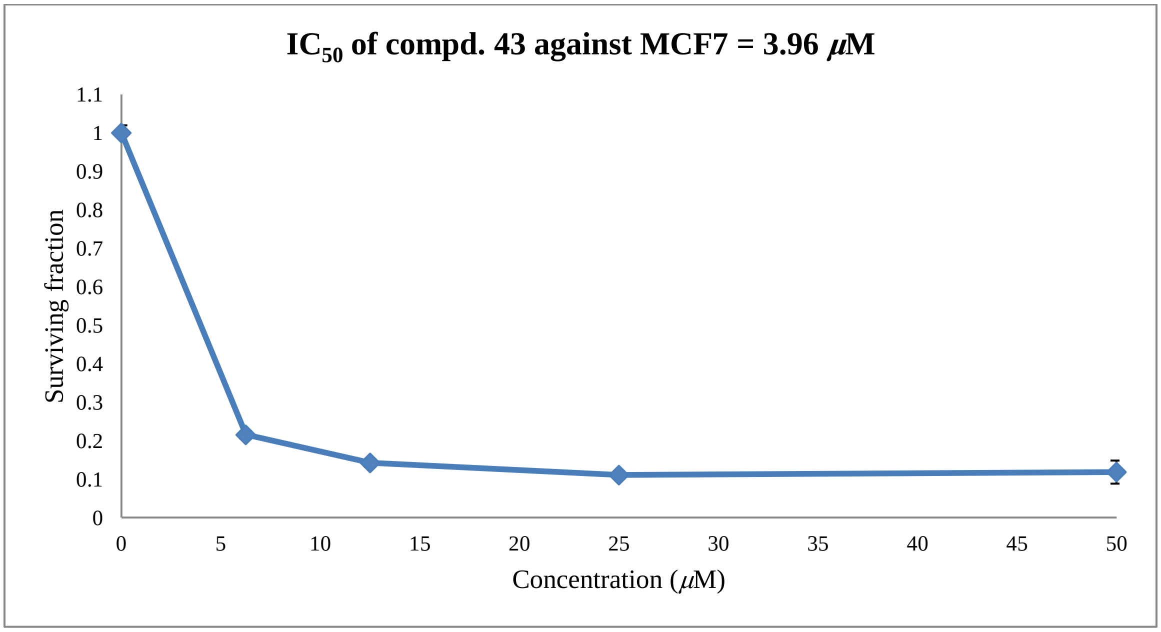
IC50 of compd. 43 against MCF7 = 3.96 μM
Surviving fraction
1.1
1
0.9
0.8
0.7
0.6
0.5
0.4
0.3
0.2
0.1
0
0
5
10
15
20
25
30
35
40
45
50
Concentration (μM)

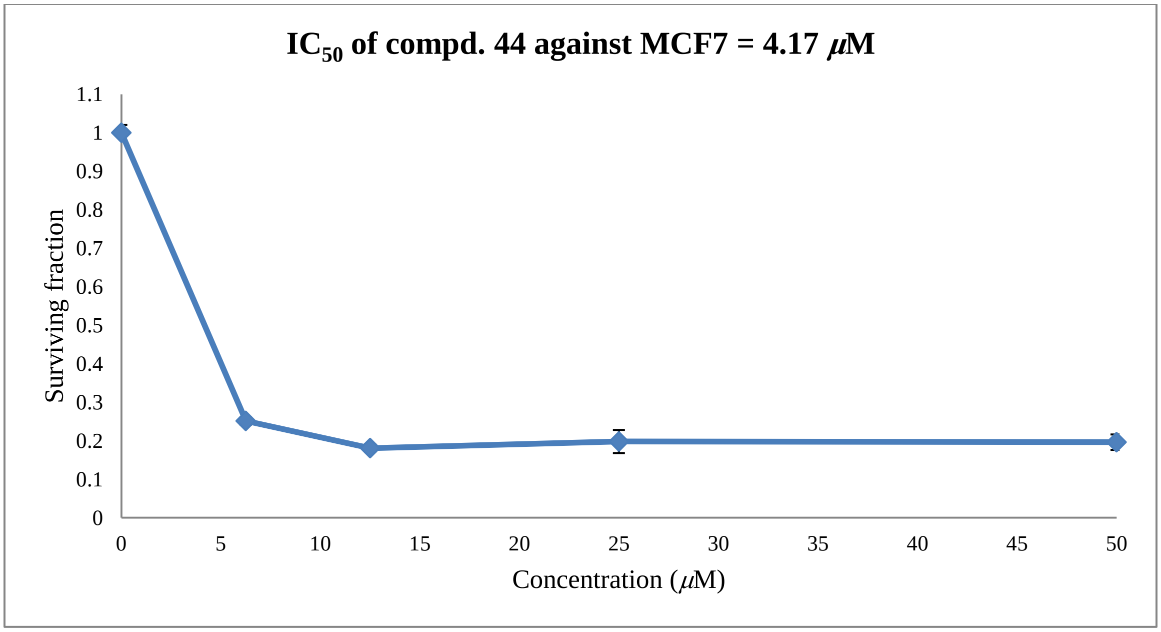
IC50 of compd. 44 against MCF7 = 4.17 μM
Surviving fraction
1.1
1
0.9
0.8
0.7
0.6
0.5
0.4
0.3
0.2
0.1
0
0
5
10
15
20
25
30
35
40
45
50
Concentration (μM)

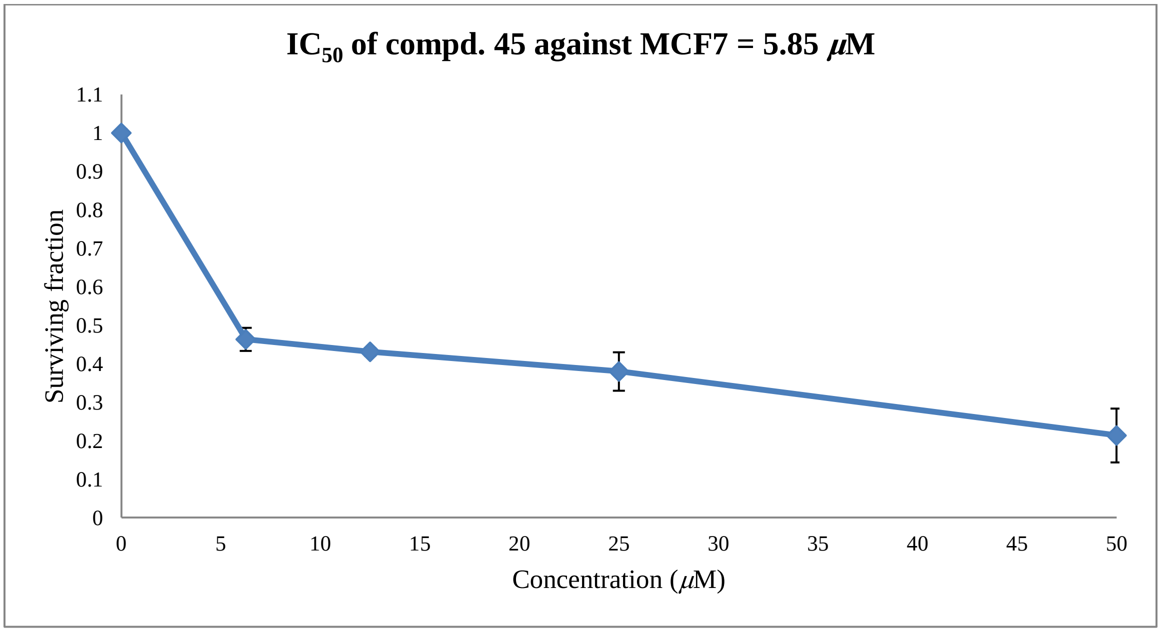
IC50 of compd. 45 against MCF7 = 5.85 μM
Surviving fraction
1.1
1
0.9
0.8
0.7
0.6
0.5
0.4
0.3
0.2
0.1
0
0
5
10
15
20
25
30
35
40
45
50
Concentration (μM)

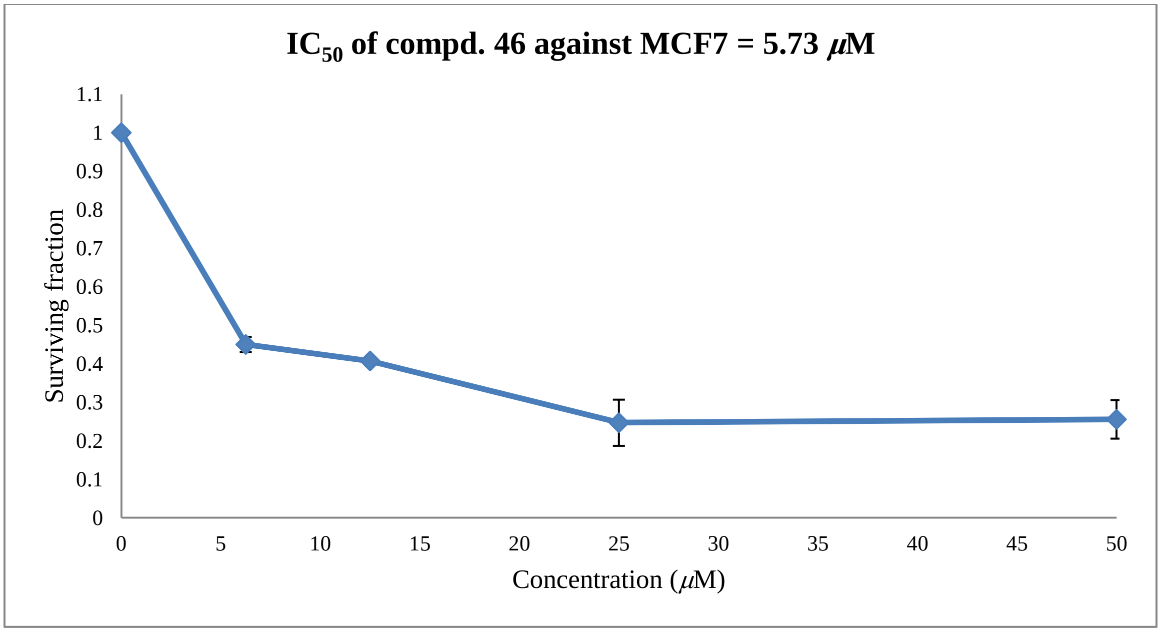
IC50 of compd. 46 against MCF7 = 5.73 μM
Surviving fraction
Concentration (μM)
1.1
1
0.9
0.8
0.7
0.6
0.5
0.4
0.3
0.2
0.1
0
0
5
10
15
20
25
30
35
40
45
50

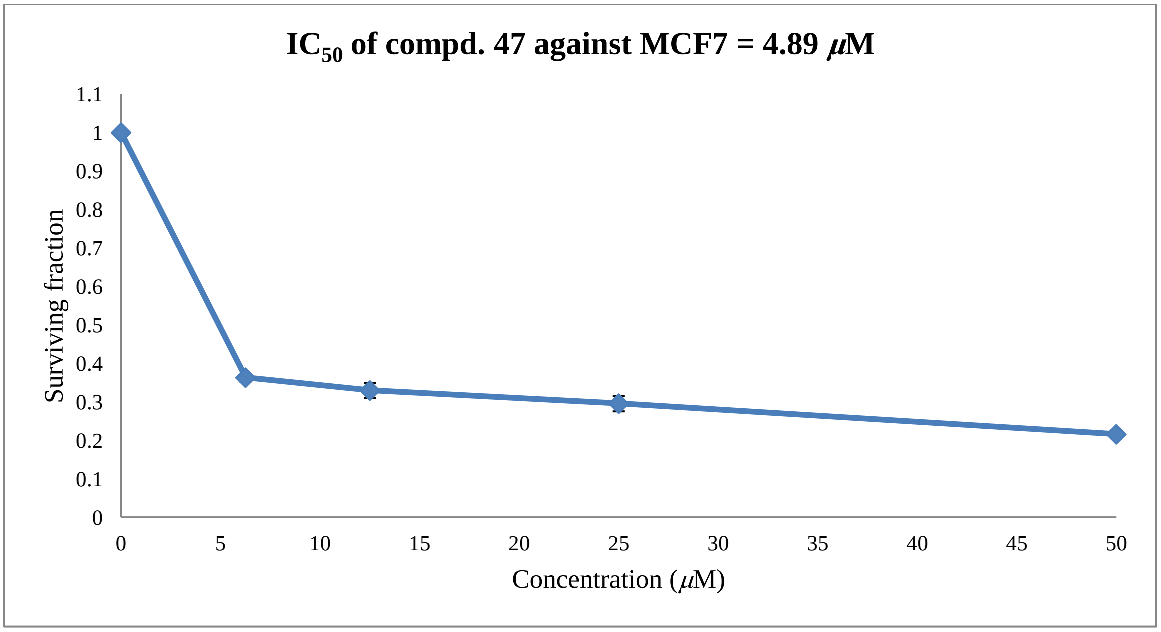
IC50 of compd. 47 against MCF7 = 4.89 μM
Surviving fraction
Concentration (μM)
1.1
1
0.9
0.8
0.7
0.6
0.5
0.4
0.3
0.2
0.1
0
0
5
10
15
20
25
30
35
40
45
50

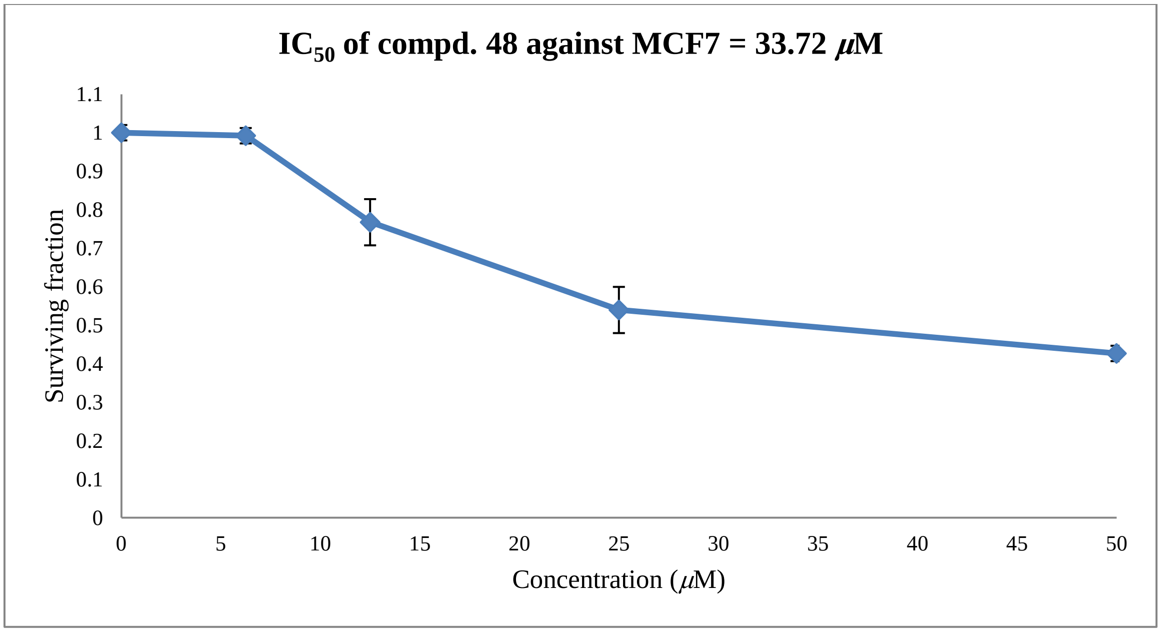
IC50 of compd. 48 against MCF7 = 33.72 μM
Surviving fraction
1.1
1
0.9
0.8
0.7
0.6
0.5
0.4
0.3
0.2
0.1
0
0
5
10
15
20
25
30
35
40
45
50
Concentration (μM)

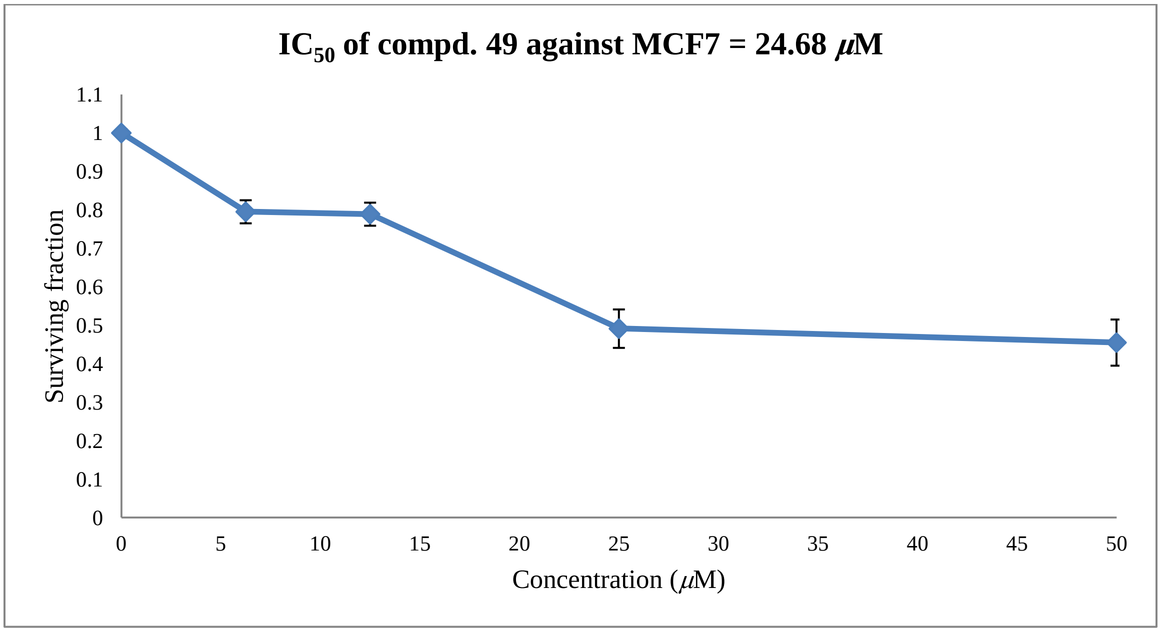
IC50 of compd. 49 against MCF7 = 24.68 μM
Surviving fraction
1.1
1
0.9
0.8
0.7
0.6
0.5
0.4
0.3
0.2
0.1
0
0
5
10
15
20
25
30
35
40
45
50
Concentration (μM)

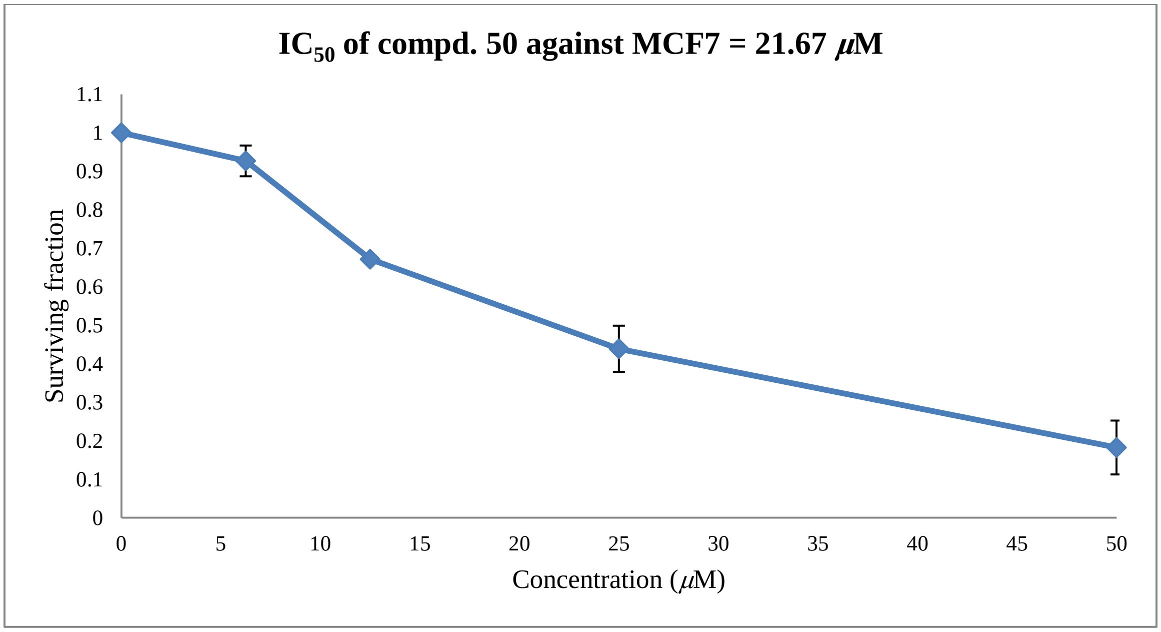
IC50 of compd. 50 against MCF7 = 21.67 μM
Surviving fraction
1.1
1
0.9
0.8
0.7
0.6
0.5
0.4
0.3
0.2
0.1
0
0
5
10
15
20
25
30
35
40
45
50
Concentration (μM)

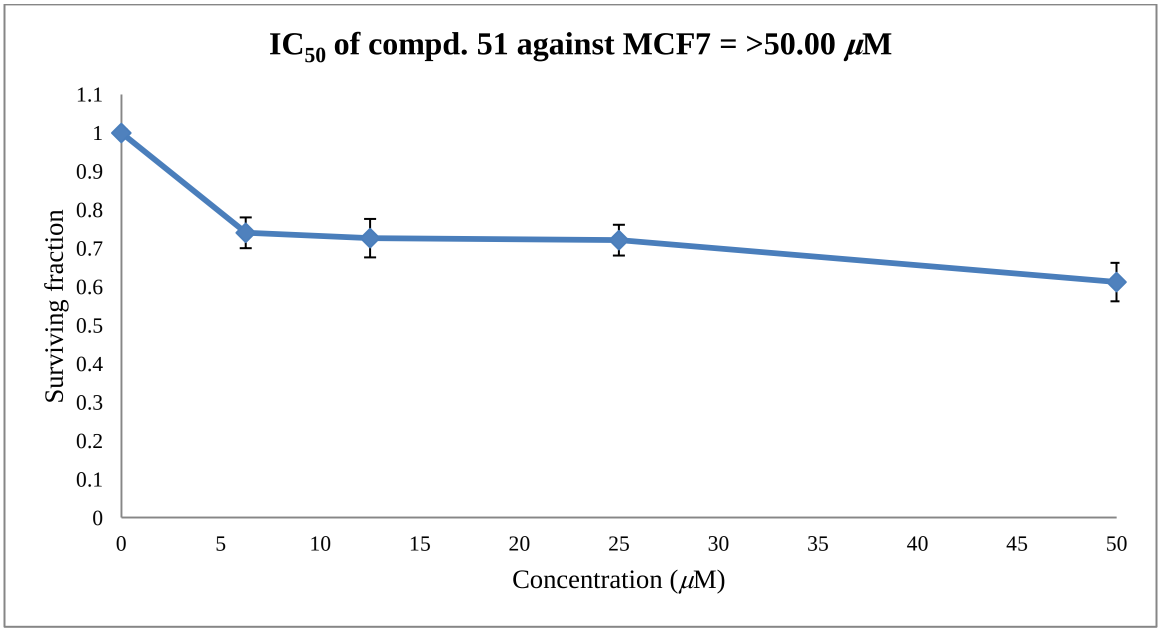
IC50 of compd. 51 against MCF7 = >50.00 μM
Surviving fraction
1.1
1
0.9
0.8
0.7
0.6
0.5
0.4
0.3
0.2
0.1
0
0
5
10
15
20
25
30
35
40
45
50
Concentration (μM)

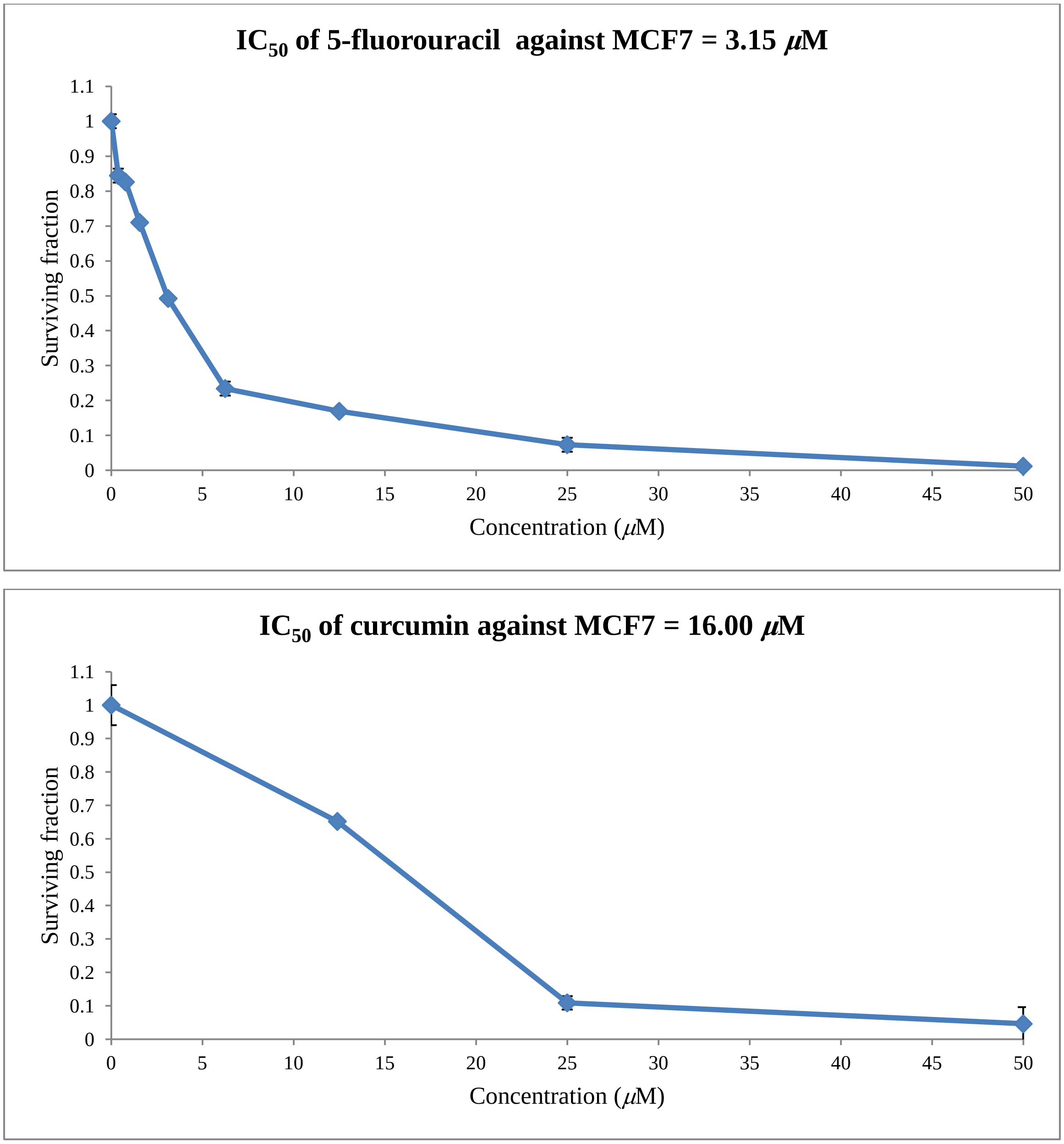

Fig. S94. Dose-response curve for the tested compounds against MCF7 (breast cancer) cell line.

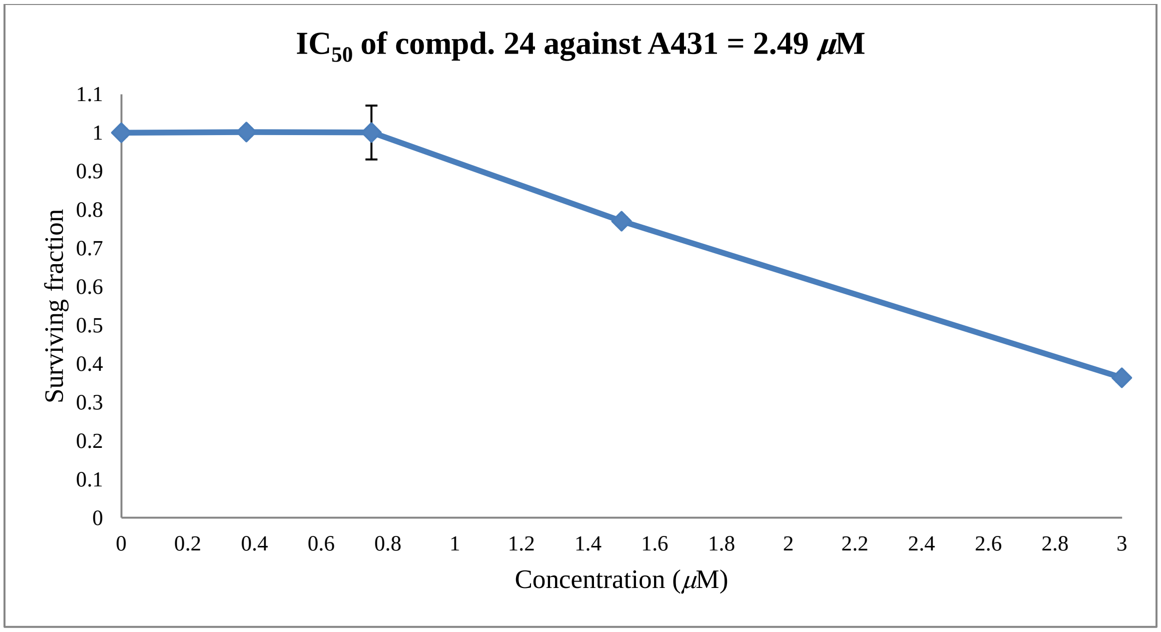
IC50 of compd. 24 against A431 = 2.49 μM
Surviving fraction
1.1
1
0.9
0.8
0.7
0.6
0.5
0.4
0.3
0.2
0.1
0
0
0.2
0.4
0.6
0.8
1
1.2
1.4
1.6
1.8
2
2.2
2.4
2.6
2.8
3
Concentration (μM)

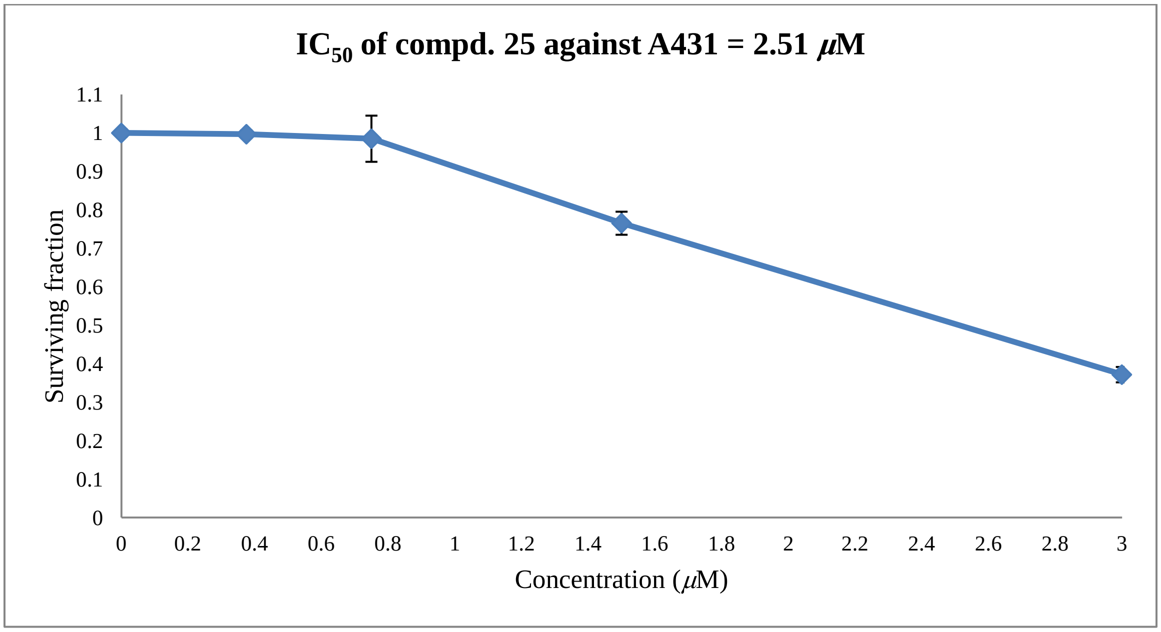
IC50 of compd. 25 against A431 = 2.51 μM
Surviving fraction
1.1
1
0.9
0.8
0.7
0.6
0.5
0.4
0.3
0.2
0.1
0
0
0.2
0.4
0.6
0.8
1
1.2
1.4
1.6
1.8
2
2.2
2.4
2.6
2.8
3
Concentration (μM)

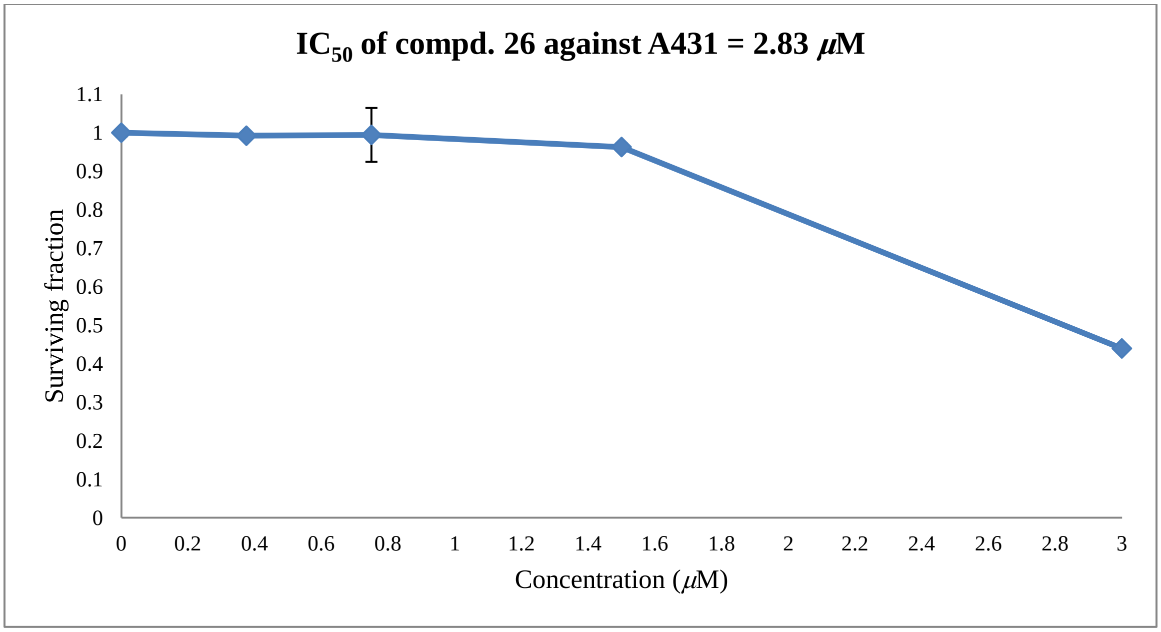

IC50 of compd. 26 against A431 = 2.83 μM
Surviving fraction
1.1
1
0.9
0.8
0.7
0.6
0.5
0.4
0.3
0.2
0.1
0
0 0.2 0.4 0.6 0.8 1 1.2 1.4 1.6 1.8 2 2.2 2.4 2.6 2.8 3
Concentration (μM)

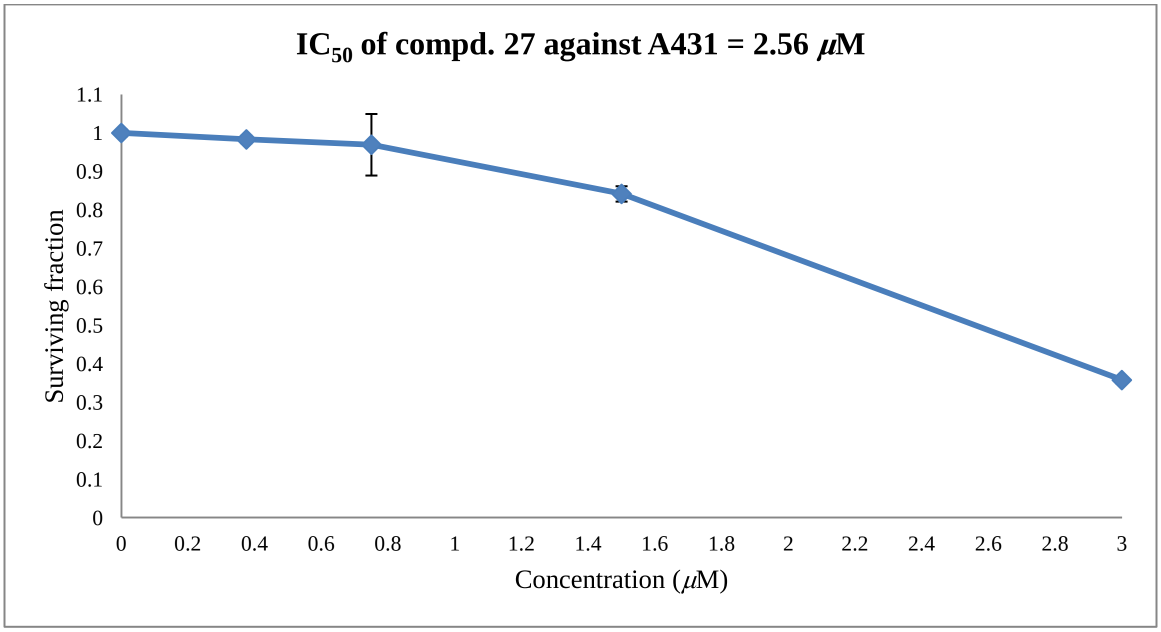

IC50 of compd. 27 against A431 = 2.56 μM
Surviving fraction
1.1
1
0.9
0.8
0.7
0.6
0.5
0.4
0.3
0.2
0.1
0
0 0.2 0.4 0.6 0.8 1 1.2 1.4 1.6 1.8 2 2.2 2.4 2.6 2.8 3
Concentration (μM)

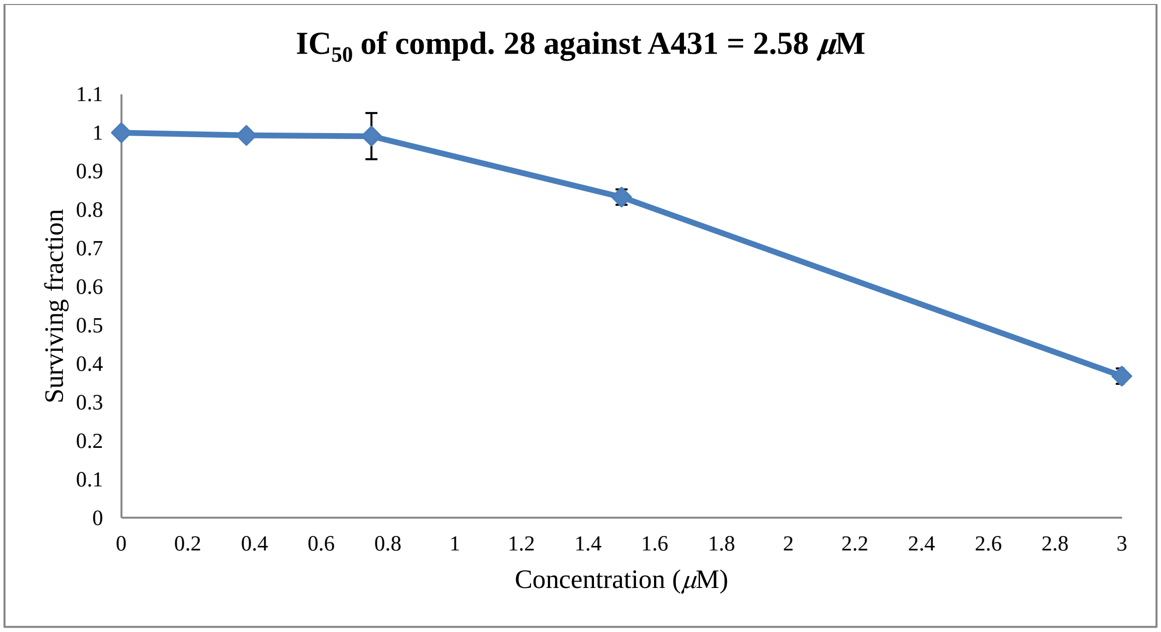
IC50 of compd. 28 against A431 = 2.58 μM
Surviving fraction
Concentration (μM)
0
0.1
0.2
0.3
0.4
0.5
0.6
0.7
0.8
0.9
1
1.1
0
0.2
0.4
0.6
0.8
1
1.2
1.4
1.6
1.8
2
2.2
2.4
2.6
2.8
3

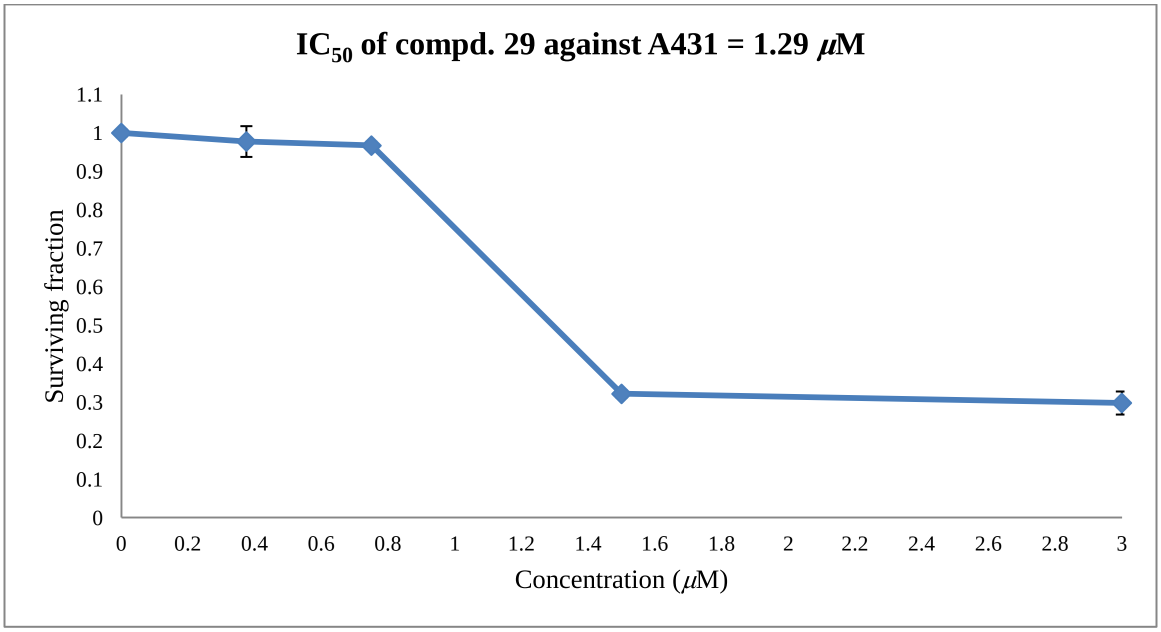
IC50 of compd. 29 against A431 = 1.29 μM
Surviving fraction
Concentration (μM)
0
0.1
0.2
0.3
0.4
0.5
0.6
0.7
0.8
0.9
1
1.1
0
0.2
0.4
0.6
0.8
1
1.2
1.4
1.6
1.8
2
2.2
2.4
2.6
2.8
3

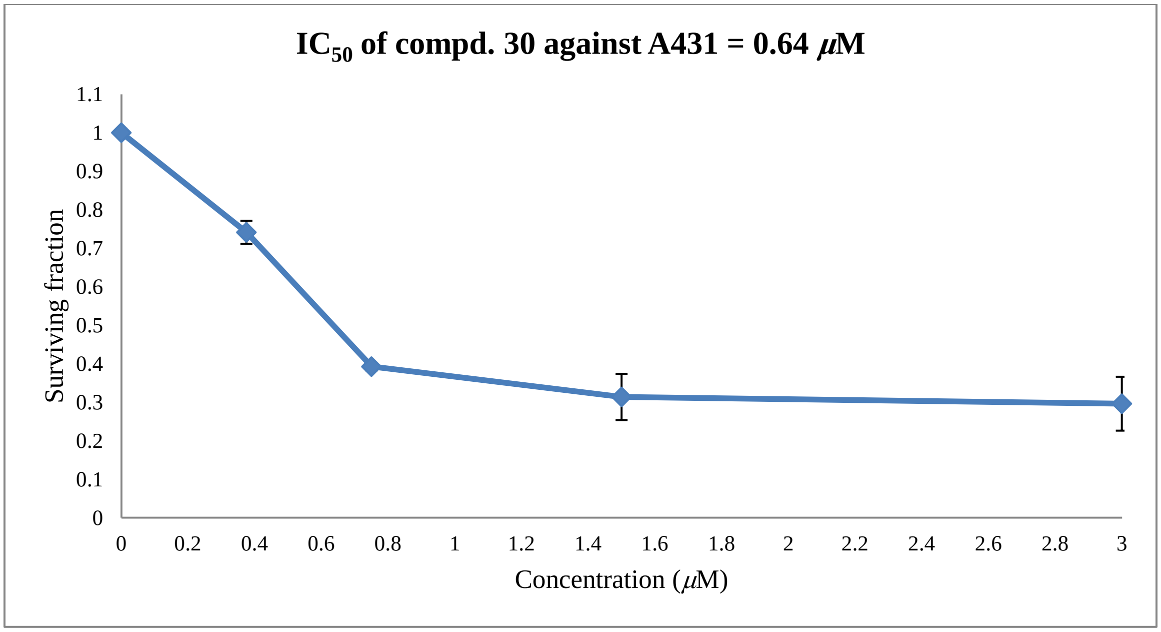
IC50 of compd. 30 against A431 = 0.64 μM
Surviving fraction
1.1
1
0.9
0.8
0.7
0.6
0.5
0.4
0.3
0.2
0.1
0
0
0.2
0.4
0.6
0.8
1
1.2
1.4
1.6
1.8
2
2.2
2.4
2.6
2.8
3
Concentration (μM)

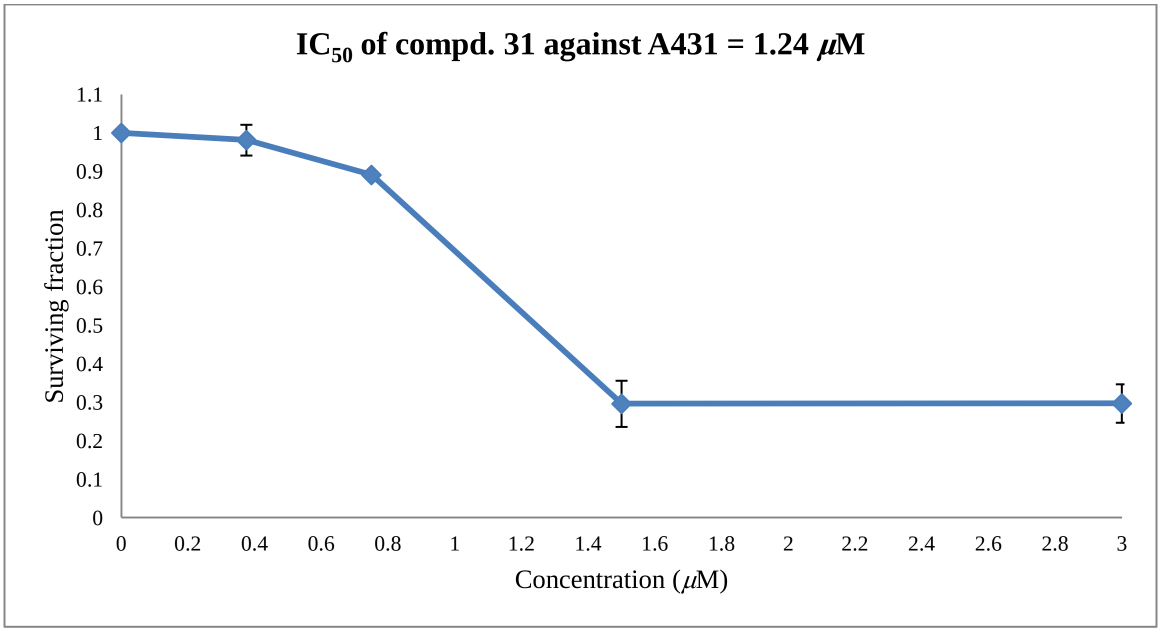
IC50 of compd. 31 against A431 = 1.24 μM
Surviving fraction
1.1
1
0.9
0.8
0.7
0.6
0.5
0.4
0.3
0.2
0.1
0
0
0.2
0.4
0.6
0.8
1
1.2
1.4
1.6
1.8
2
2.2
2.4
2.6
2.8
3
Concentration (μM)

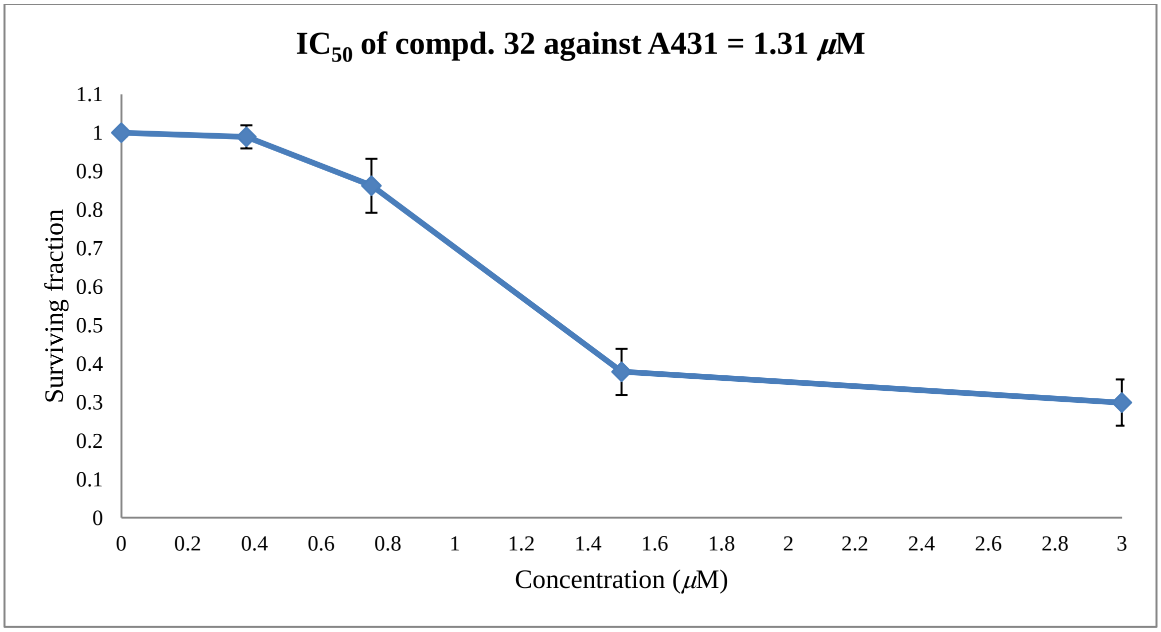
IC50 of compd. 32 against A431 = 1.31 μM
Surviving fraction
1.1
1
0.9
0.8
0.7
0.6
0.5
0.4
0.3
0.2
0.1
0
0
0.2
0.4
0.6
0.8
1
1.2
1.4
1.6
1.8
2
2.2
2.4
2.6
2.8
3
Concentration (μM)

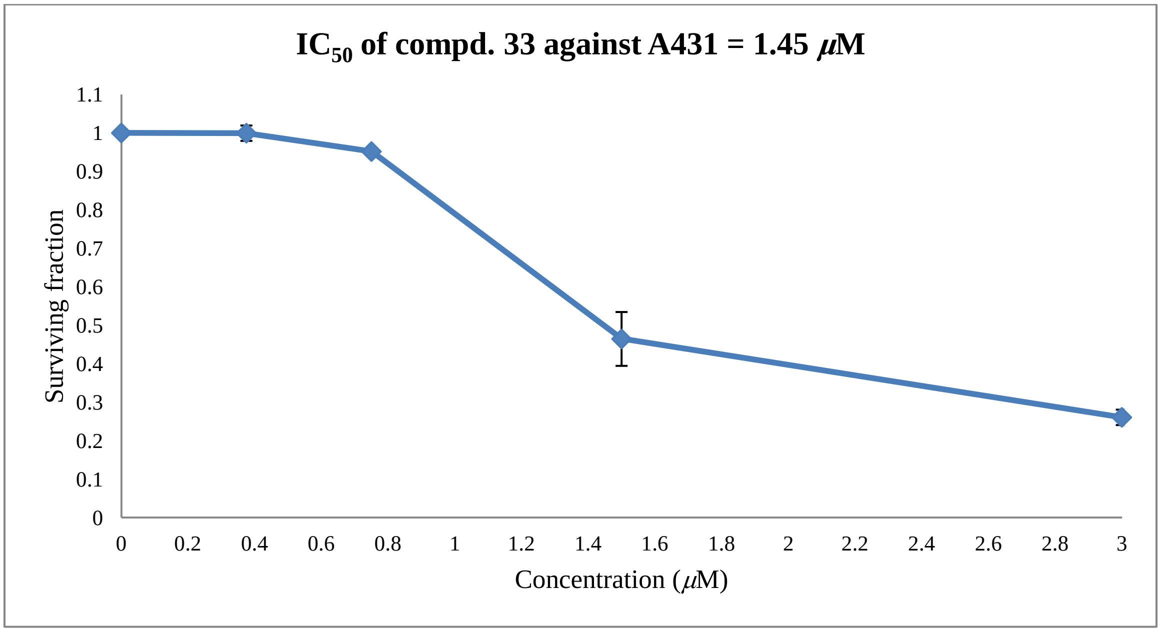
IC50 of compd. 33 against A431 = 1.45 μM
Surviving fraction
1.1
1
0.9
0.8
0.7
0.6
0.5
0.4
0.3
0.2
0.1
0
0
0.2
0.4
0.6
0.8
1
1.2
1.4
1.6
1.8
2
2.2
2.4
2.6
2.8
3
Concentration (μM)

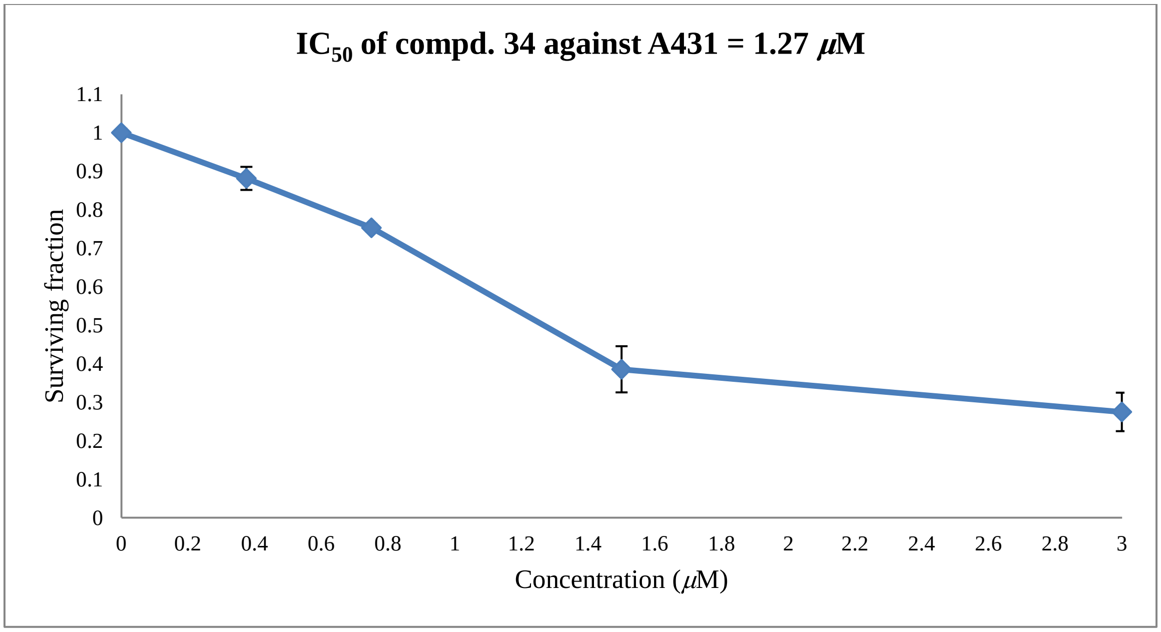
IC50 of compd. 34 against A431 = 1.27 μM
Surviving fraction
1.1
1
0.9
0.8
0.7
0.6
0.5
0.4
0.3
0.2
0.1
0
0 0.2 0.4 0.6 0.8 1 1.2 1.4 1.6 1.8 2 2.2 2.4 2.6 2.8 3
Concentration (μM)

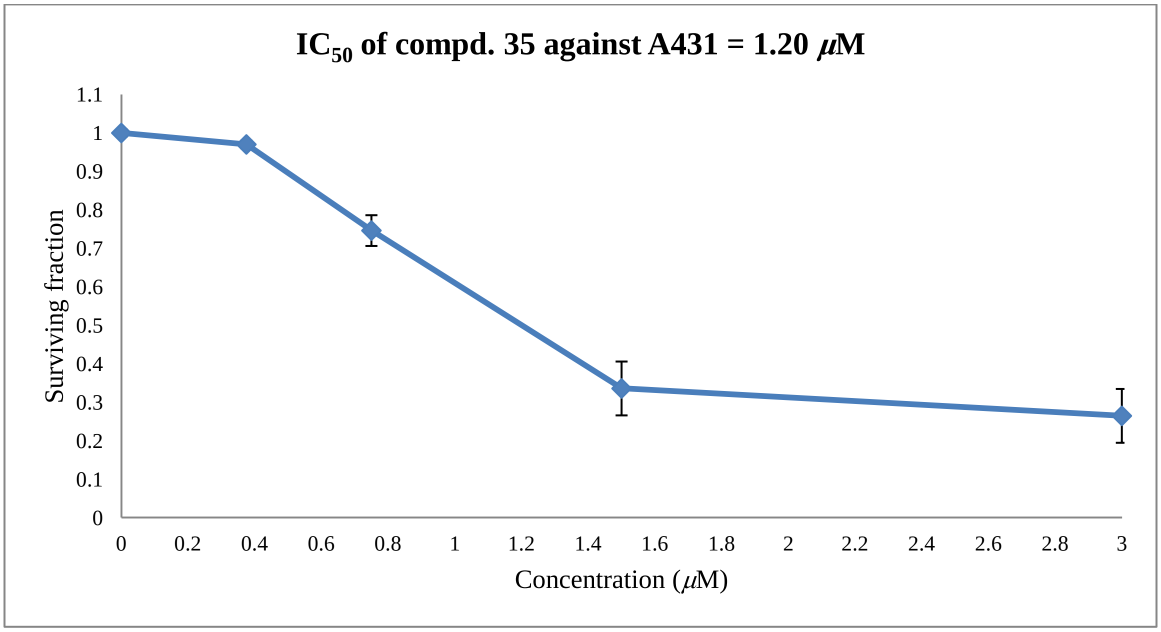
IC50 of compd. 35 against A431 = 1.20 μM
Surviving fraction
1.1
1
0.9
0.8
0.7
0.6
0.5
0.4
0.3
0.2
0.1
0
0 0.2 0.4 0.6 0.8 1 1.2 1.4 1.6 1.8 2 2.2 2.4 2.6 2.8 3
Concentration (μM)

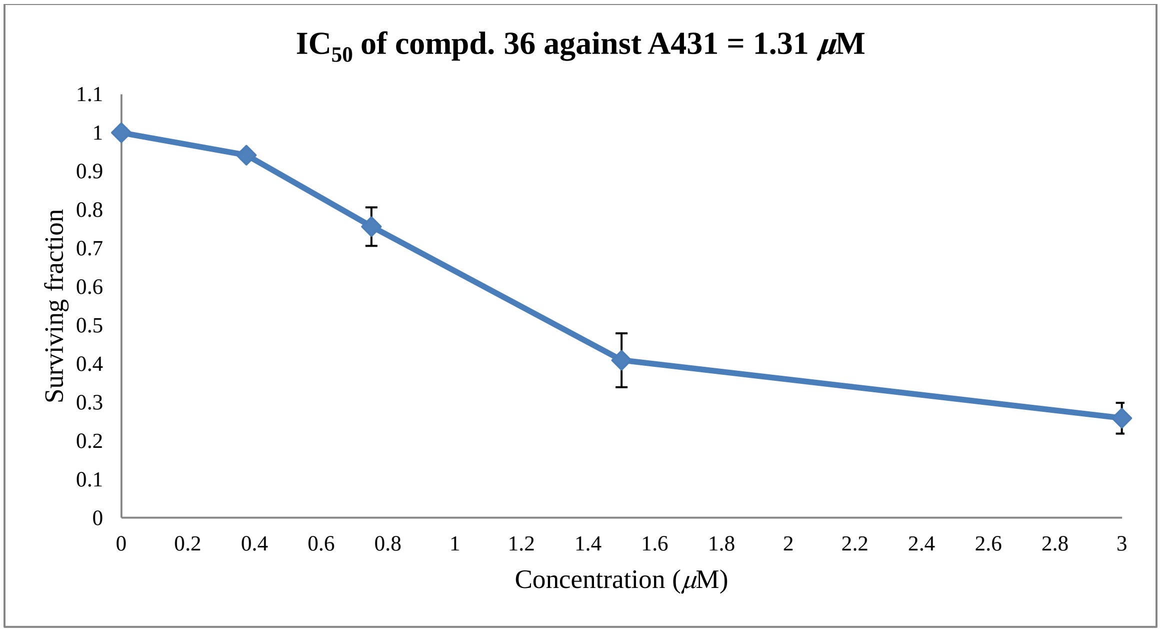
IC$_{50}$ of compd. 36 against A431 = 1.31 μM
Surviving fraction
Concentration (μM)
0
0.1
0.2
0.3
0.4
0.5
0.6
0.7
0.8
0.9
1
1.1
0.2
0.4
0.6
0.8
1.2
1.4
1.6
1.8
2
2.2
2.4
2.6
2.8
3

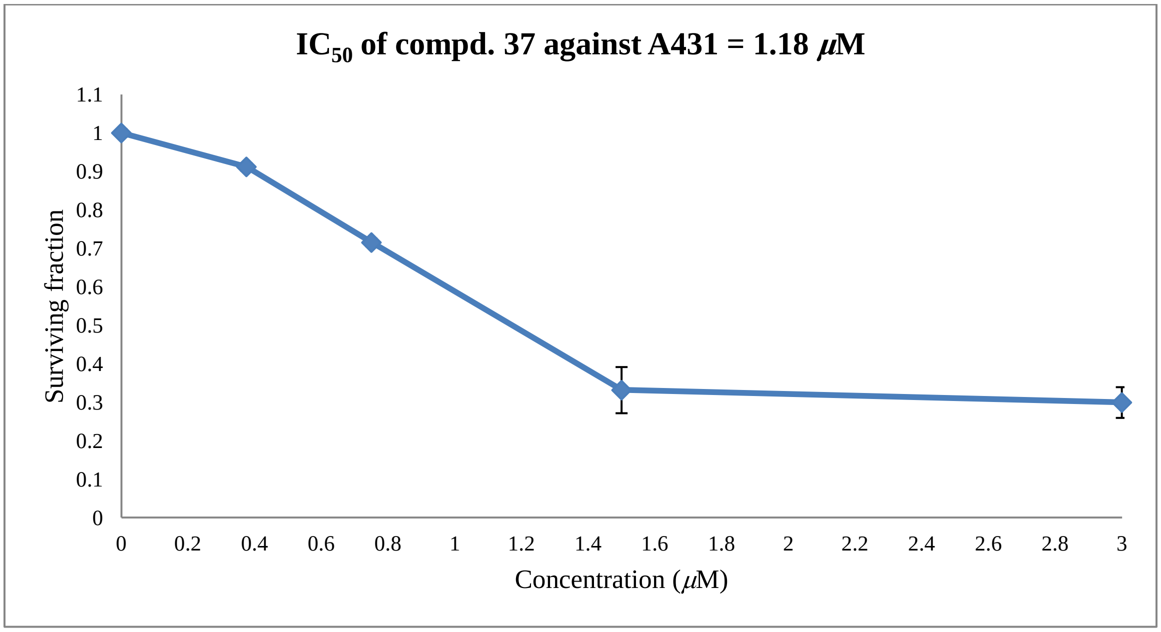
IC$_{50}$ of compd. 37 against A431 = 1.18 μM
Surviving fraction
Concentration (μM)
0
0.1
0.2
0.3
0.4
0.5
0.6
0.7
0.8
0.9
1
1.1
0.2
0.4
0.6
0.8
1.2
1.4
1.6
1.8
2
2.2
2.4
2.6
2.8
3

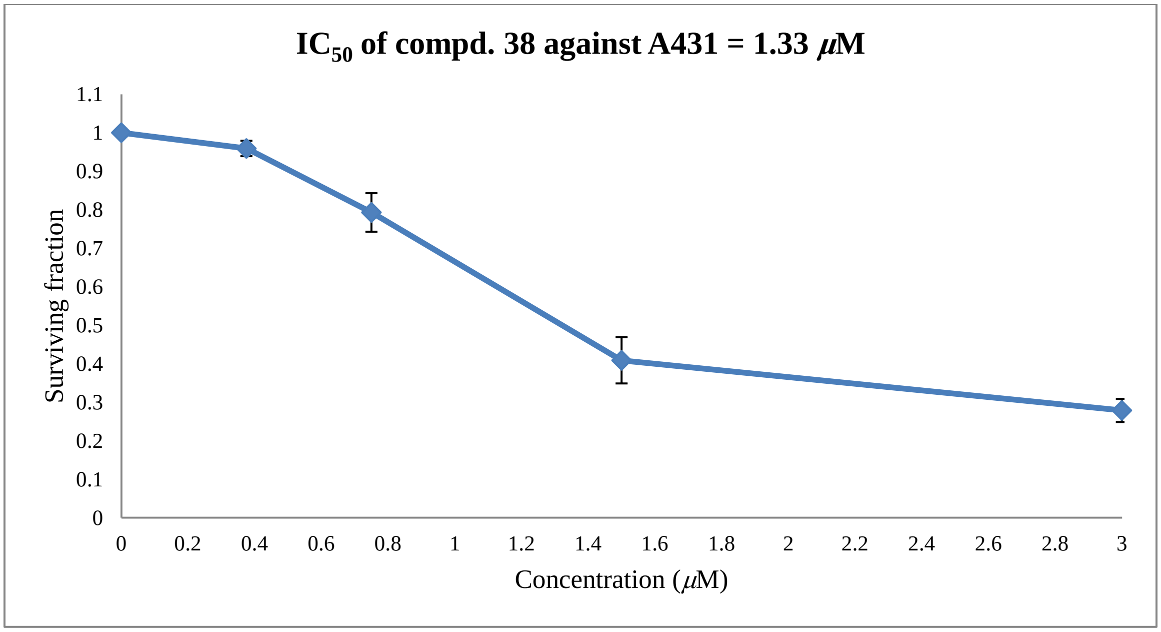
IC50 of compd. 38 against A431 = 1.33 μM
Surviving fraction
1.1
1
0.9
0.8
0.7
0.6
0.5
0.4
0.3
0.2
0.1
0
0
0.2
0.4
0.6
0.8
1
1.2
1.4
1.6
1.8
2
2.2
2.4
2.6
2.8
3
Concentration (μM)

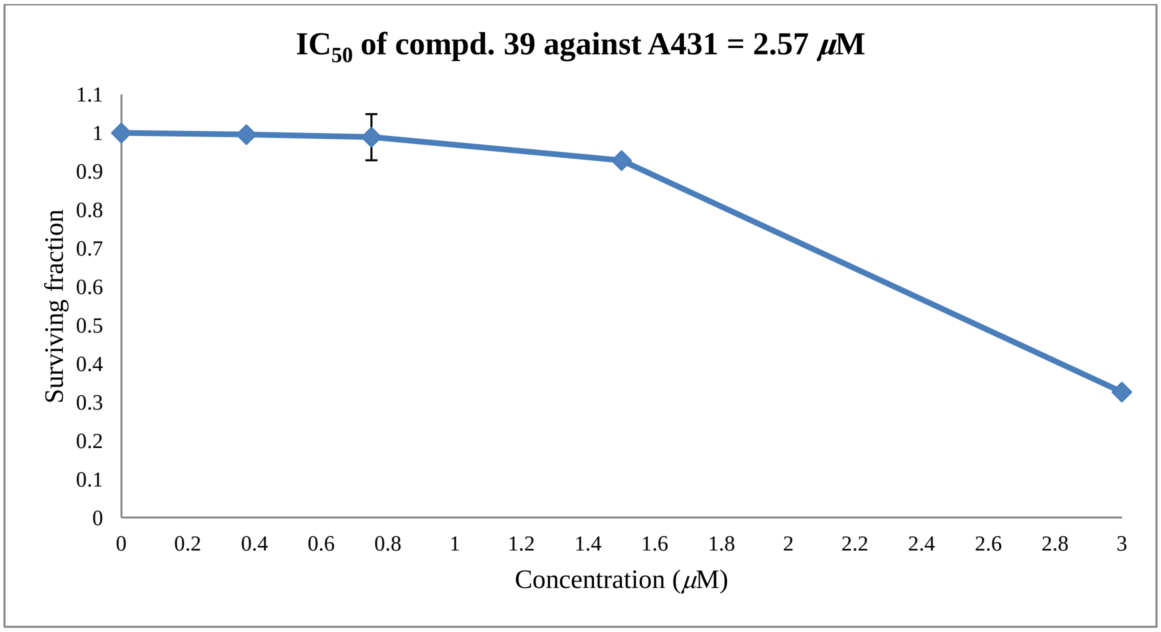
IC50 of compd. 39 against A431 = 2.57 μM
Surviving fraction
1.1
1
0.9
0.8
0.7
0.6
0.5
0.4
0.3
0.2
0.1
0
0
0.2
0.4
0.6
0.8
1
1.2
1.4
1.6
1.8
2
2.2
2.4
2.6
2.8
3
Concentration (μM)

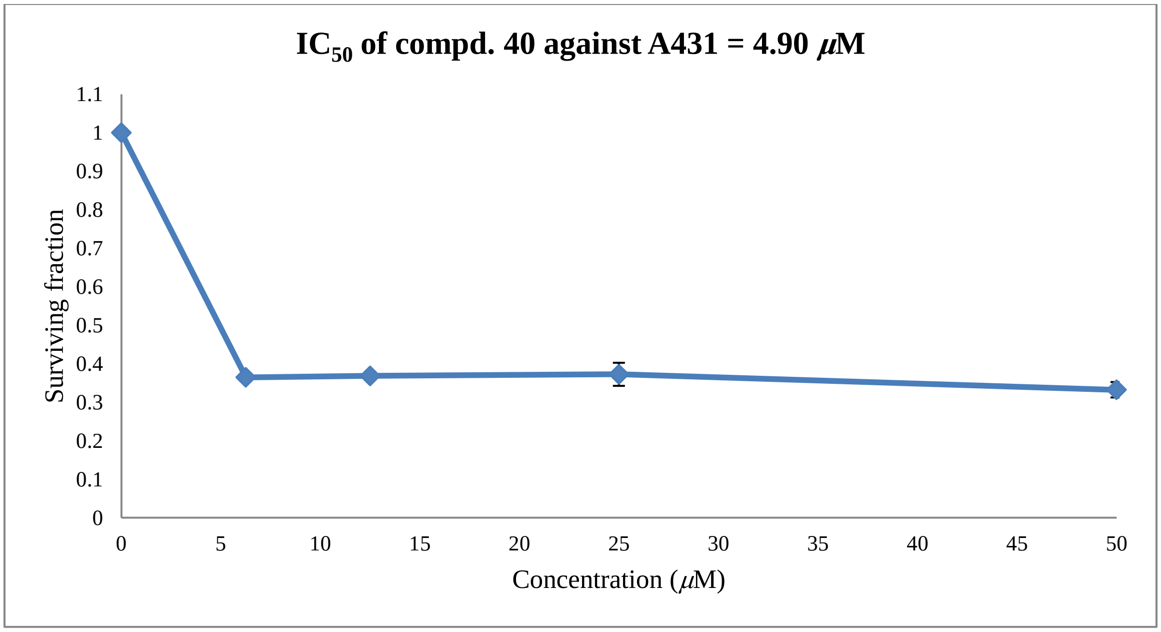
IC50 of compd. 40 against A431 = 4.90 μM
Surviving fraction
Concentration (μM)
1.1
1
0.9
0.8
0.7
0.6
0.5
0.4
0.3
0.2
0.1
0
0
5
10
15
20
25
30
35
40
45
50

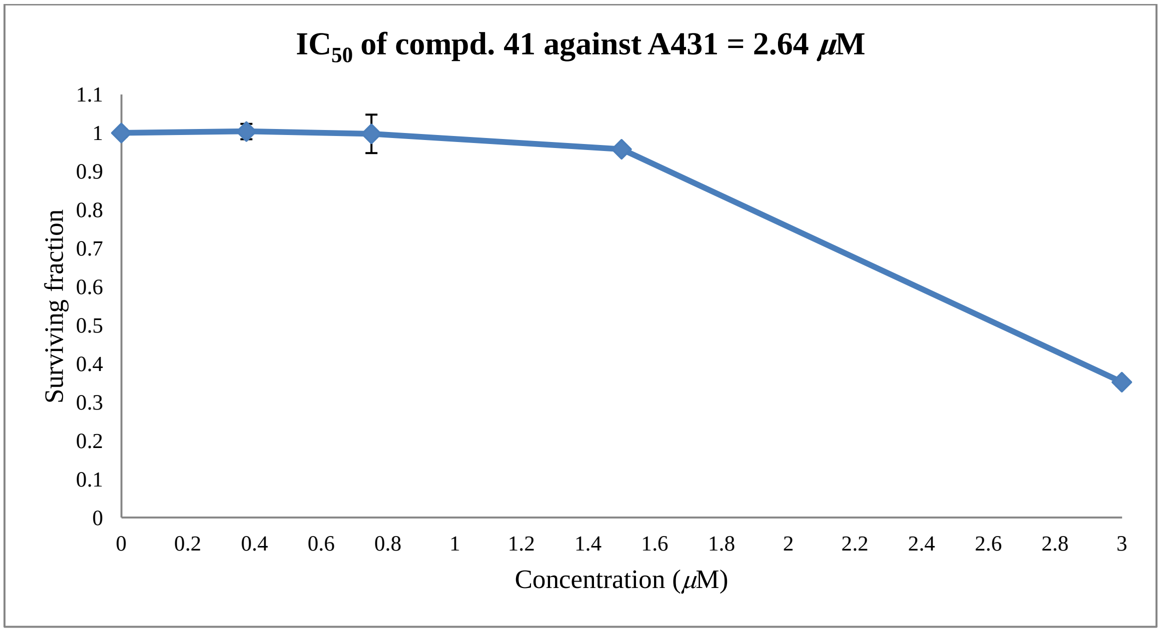
IC50 of compd. 41 against A431 = 2.64 μM
Surviving fraction
Concentration (μM)
1.1
1
0.9
0.8
0.7
0.6
0.5
0.4
0.3
0.2
0.1
0
0
0.2
0.4
0.6
0.8
1
1.2
1.4
1.6
1.8
2
2.2
2.4
2.6
2.8
3

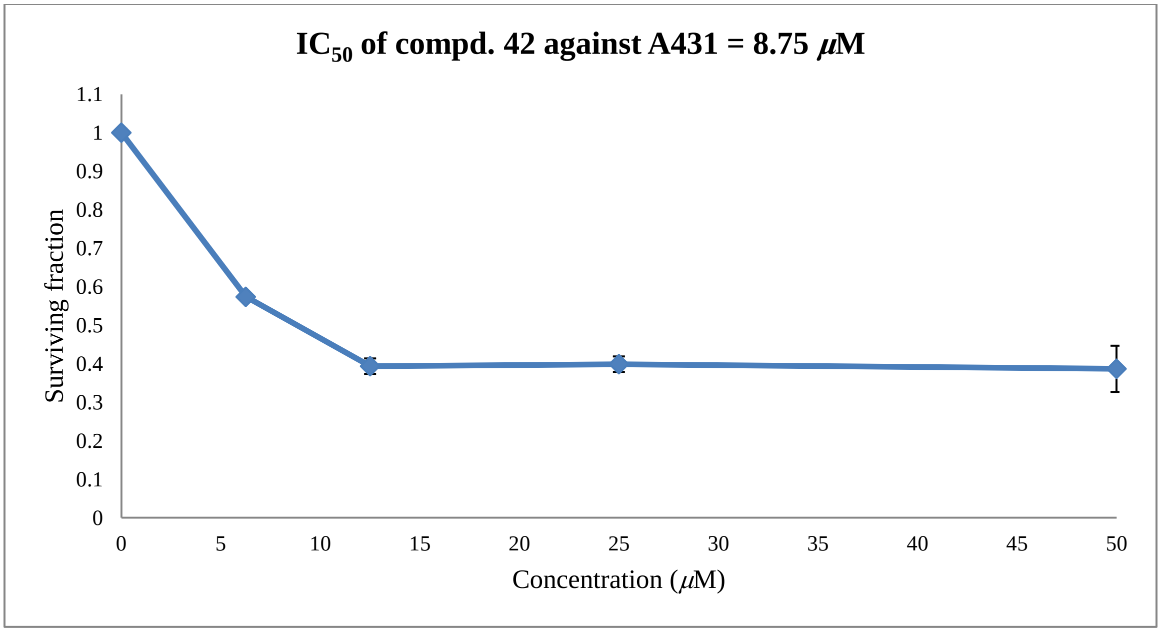
IC50 of compd. 42 against A431 = 8.75 μM
Surviving fraction
1.1
1
0.9
0.8
0.7
0.6
0.5
0.4
0.3
0.2
0.1
0
0 5 10 15 20 25 30 35 40 45 50
Concentration (μM)

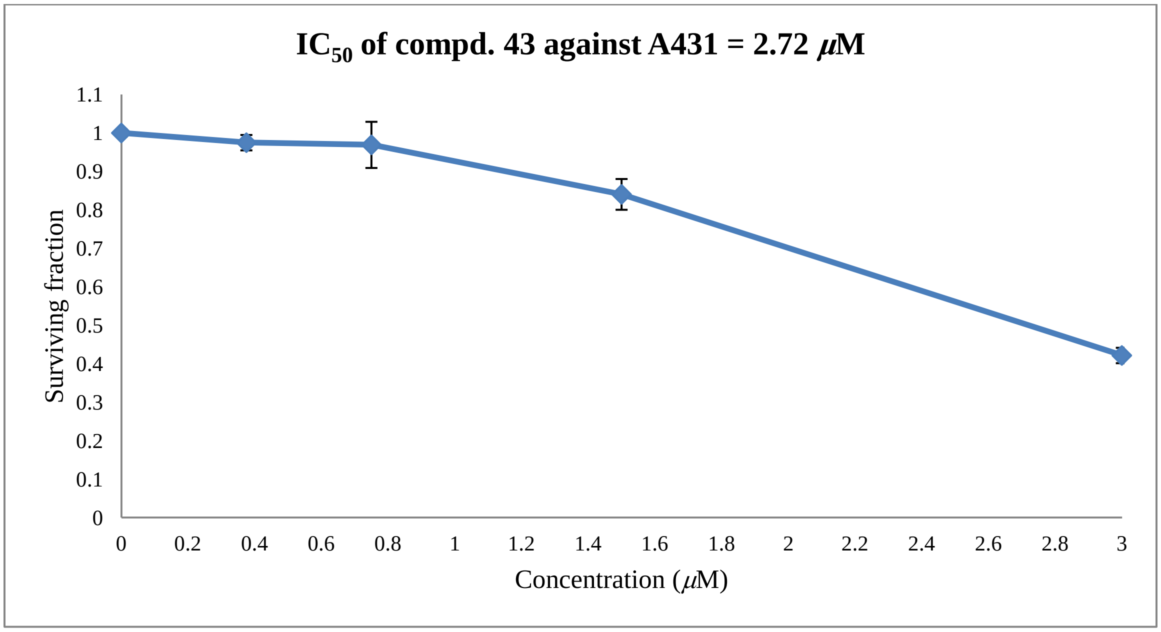
IC50 of compd. 43 against A431 = 2.72 μM
Surviving fraction
1.1
1
0.9
0.8
0.7
0.6
0.5
0.4
0.3
0.2
0.1
0
0 0.2 0.4 0.6 0.8 1 1.2 1.4 1.6 1.8 2 2.2 2.4 2.6 2.8 3
Concentration (μM)

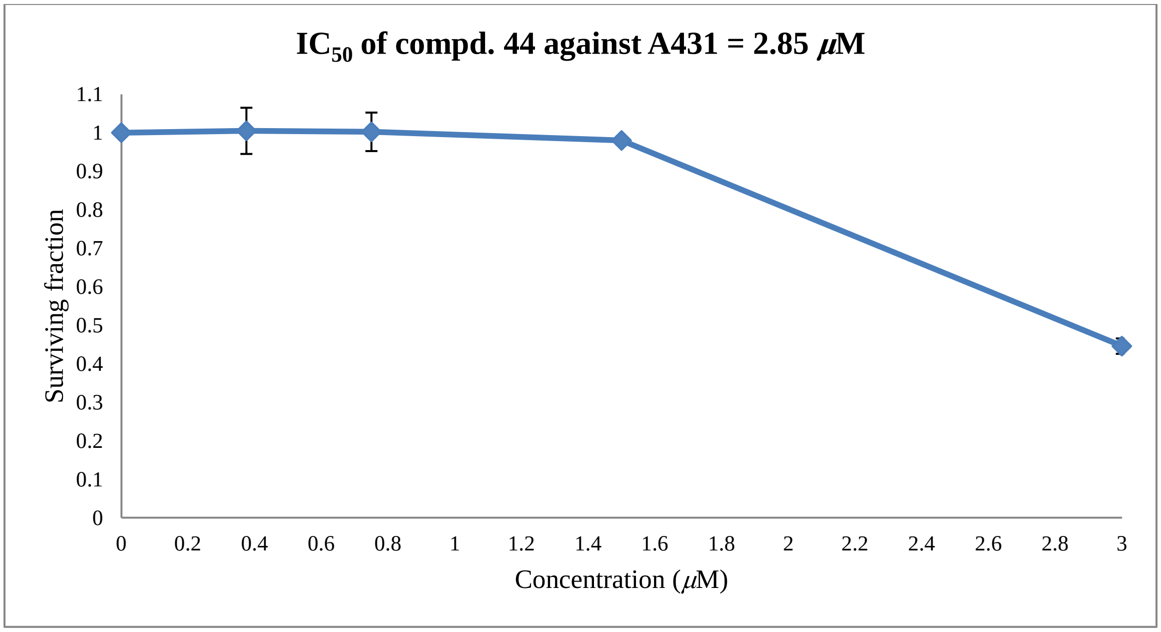
IC50 of compd. 44 against A431 = 2.85 µM
Surviving fraction
1.1
1
0.9
0.8
0.7
0.6
0.5
0.4
0.3
0.2
0.1
0
0
0.2
0.4
0.6
0.8
1
1.2
1.4
1.6
1.8
2
2.2
2.4
2.6
2.8
3
Concentration (µM)

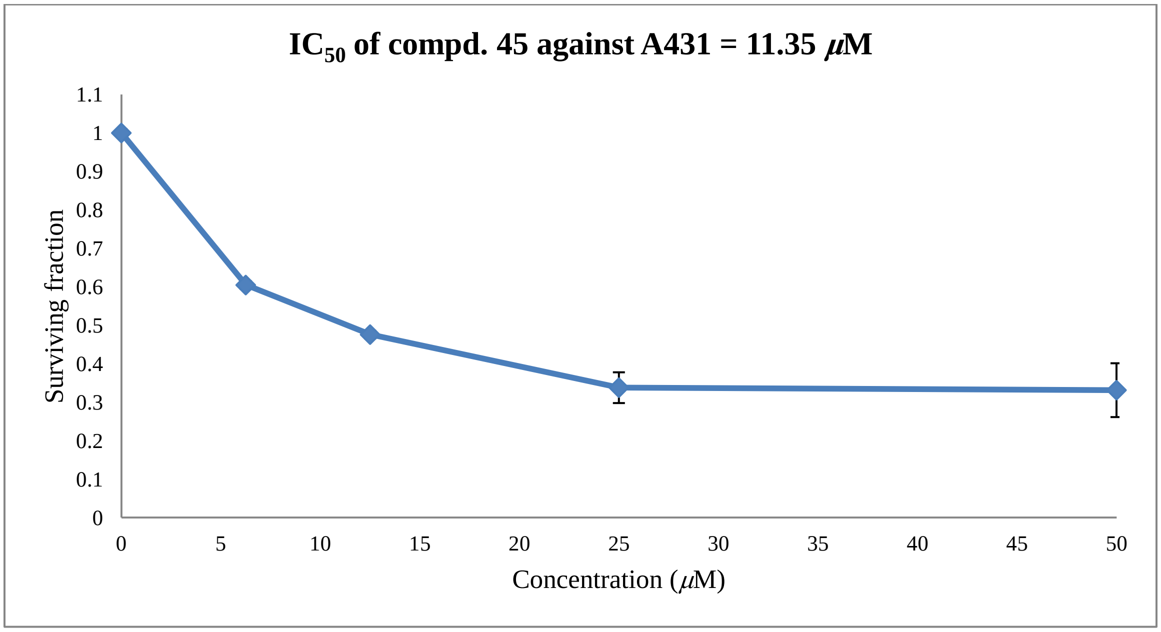
IC50 of compd. 45 against A431 = 11.35 µM
Surviving fraction
1.1
1
0.9
0.8
0.7
0.6
0.5
0.4
0.3
0.2
0.1
0
0
5
10
15
20
25
30
35
40
45
50
Concentration (µM)

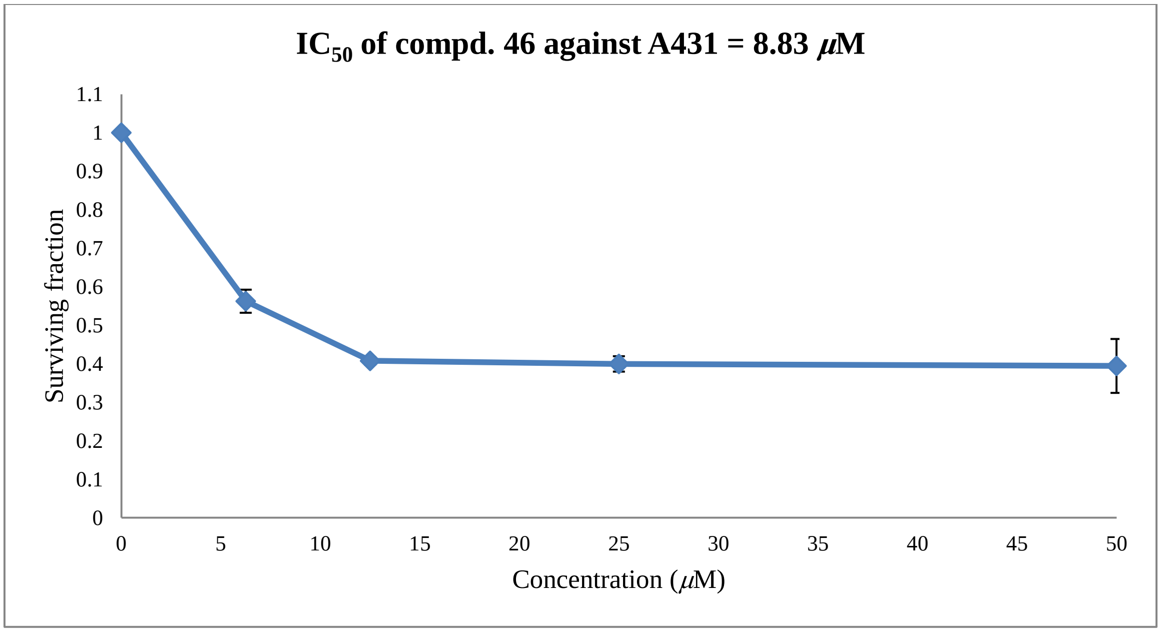
IC50 of compd. 46 against A431 = 8.83 μM
Surviving fraction
1.1
1
0.9
0.8
0.7
0.6
0.5
0.4
0.3
0.2
0.1
0
0
5
10
15
20
25
30
35
40
45
50
Concentration (μM)

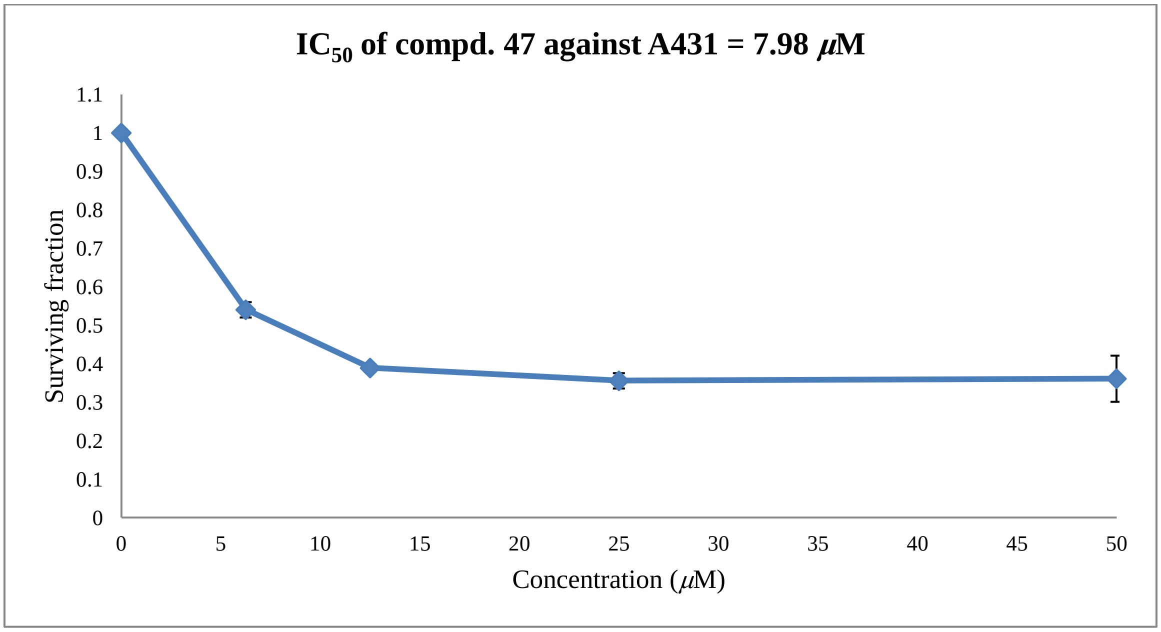
IC50 of compd. 47 against A431 = 7.98 μM
Surviving fraction
1.1
1
0.9
0.8
0.7
0.6
0.5
0.4
0.3
0.2
0.1
0
0
5
10
15
20
25
30
35
40
45
50
Concentration (μM)

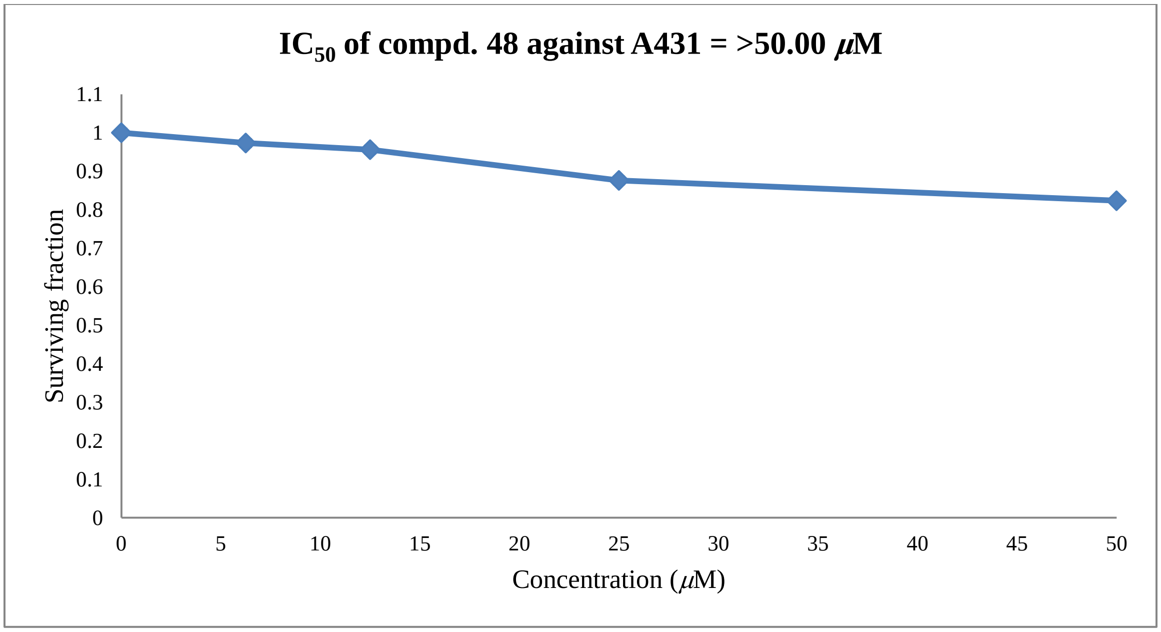
IC50 of compd. 48 against A431 = >50.00 μM
Surviving fraction
1.1
1
0.9
0.8
0.7
0.6
0.5
0.4
0.3
0.2
0.1
0
0
5
10
15
20
25
30
35
40
45
50
Concentration (μM)

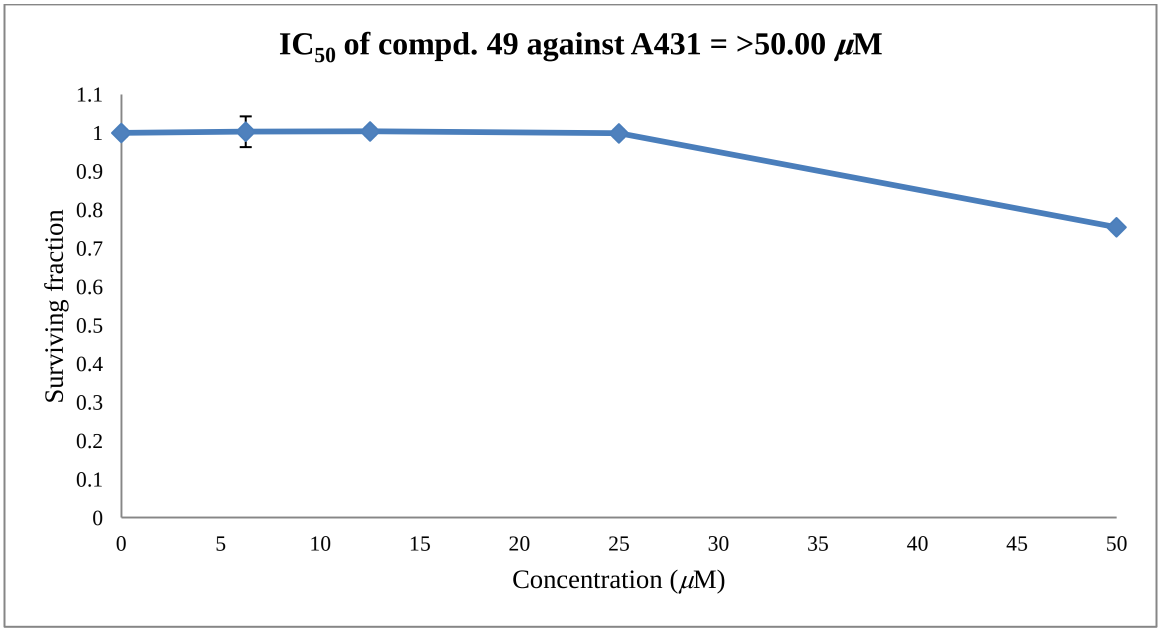
IC50 of compd. 49 against A431 = >50.00 μM
Surviving fraction
1.1
1
0.9
0.8
0.7
0.6
0.5
0.4
0.3
0.2
0.1
0
0
5
10
15
20
25
30
35
40
45
50
Concentration (μM)

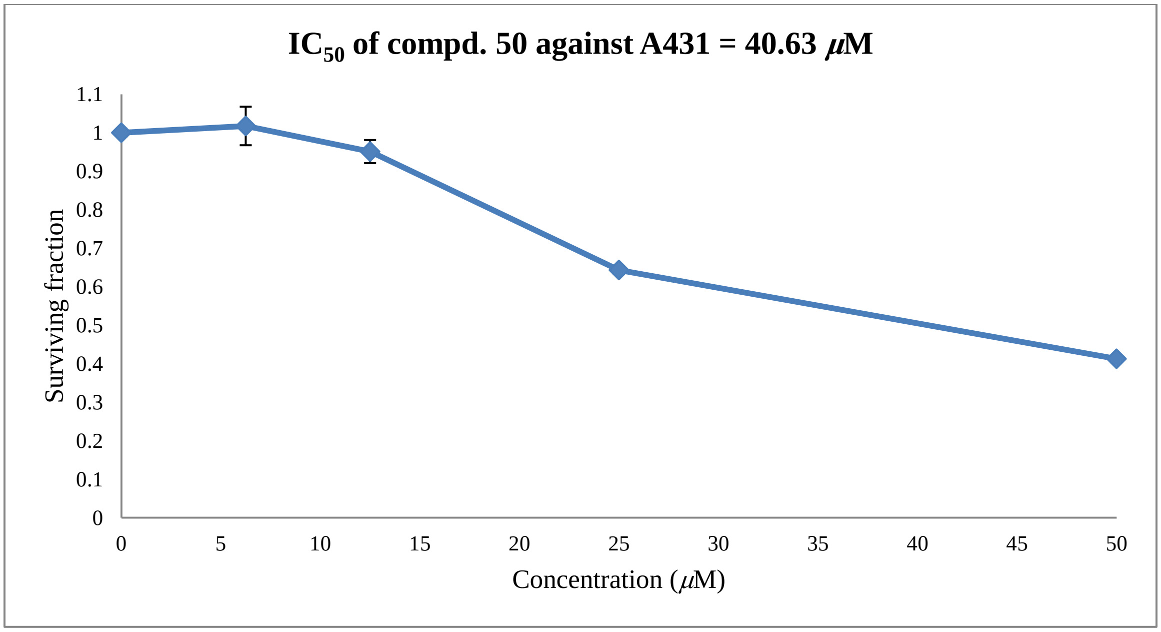
IC50 of compd. 50 against A431 = 40.63 μM
Surviving fraction
1.1
1
0.9
0.8
0.7
0.6
0.5
0.4
0.3
0.2
0.1
0
0
5
10
15
20
25
30
35
40
45
50
Concentration (μM)

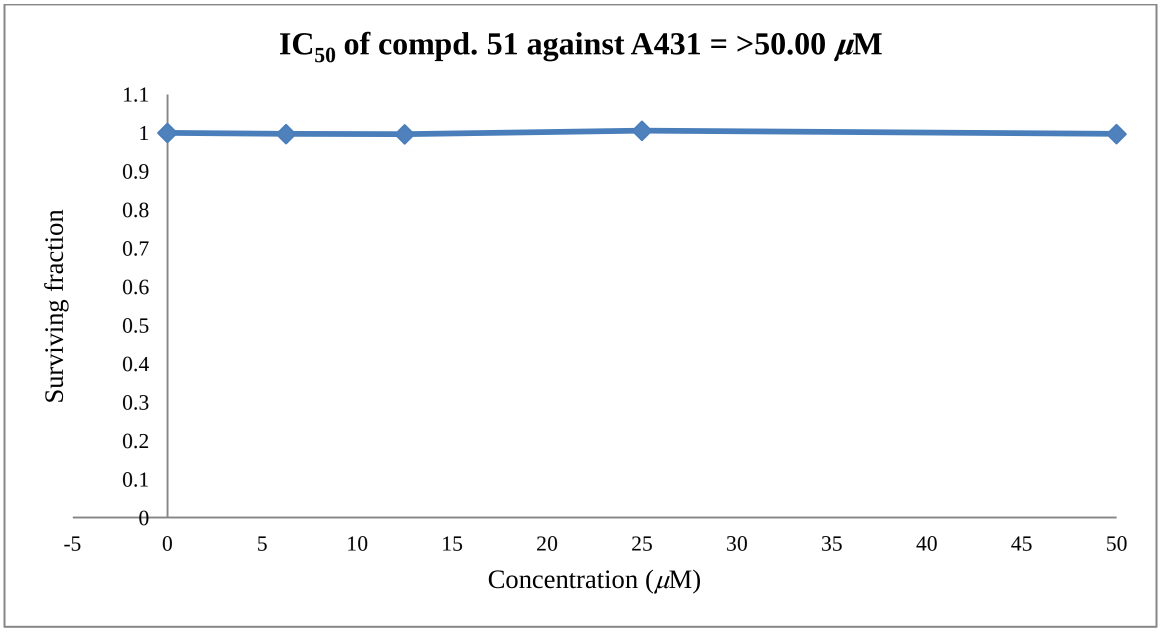
IC50 of compd. 51 against A431 = >50.00 μM
Surviving fraction
1.1
1
0.9
0.8
0.7
0.6
0.5
0.4
0.3
0.2
0.1
0
-5
0
5
10
15
20
25
30
35
40
45
50
Concentration (μM)

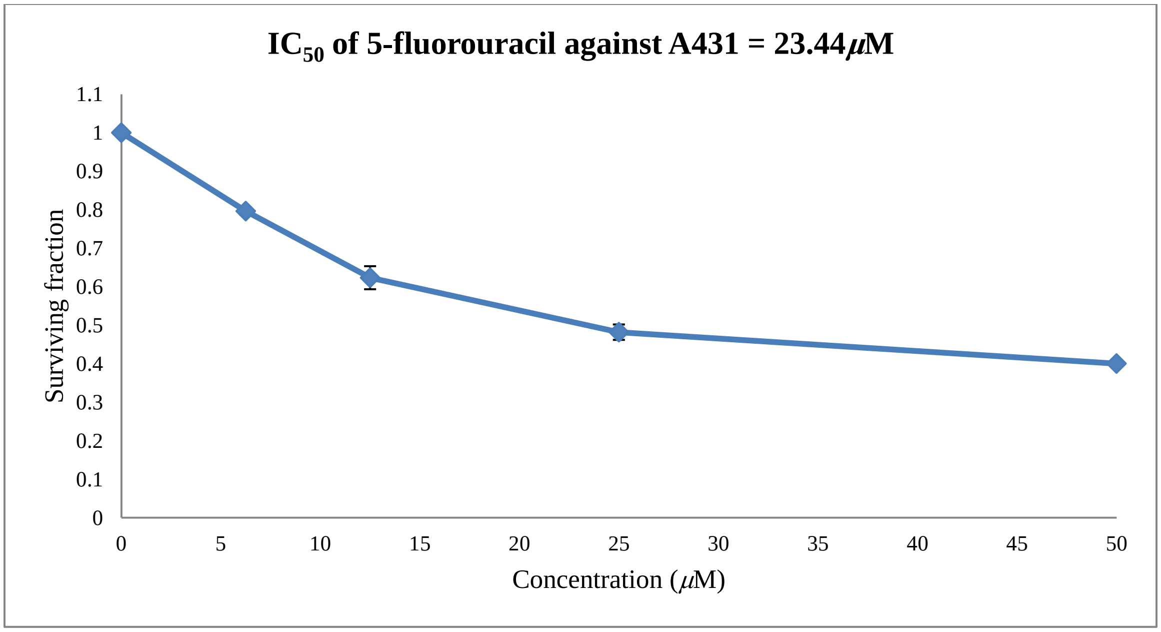

Fig. S95. Dose-response curve for the tested compounds against A431 (squemous cancer) cell line.

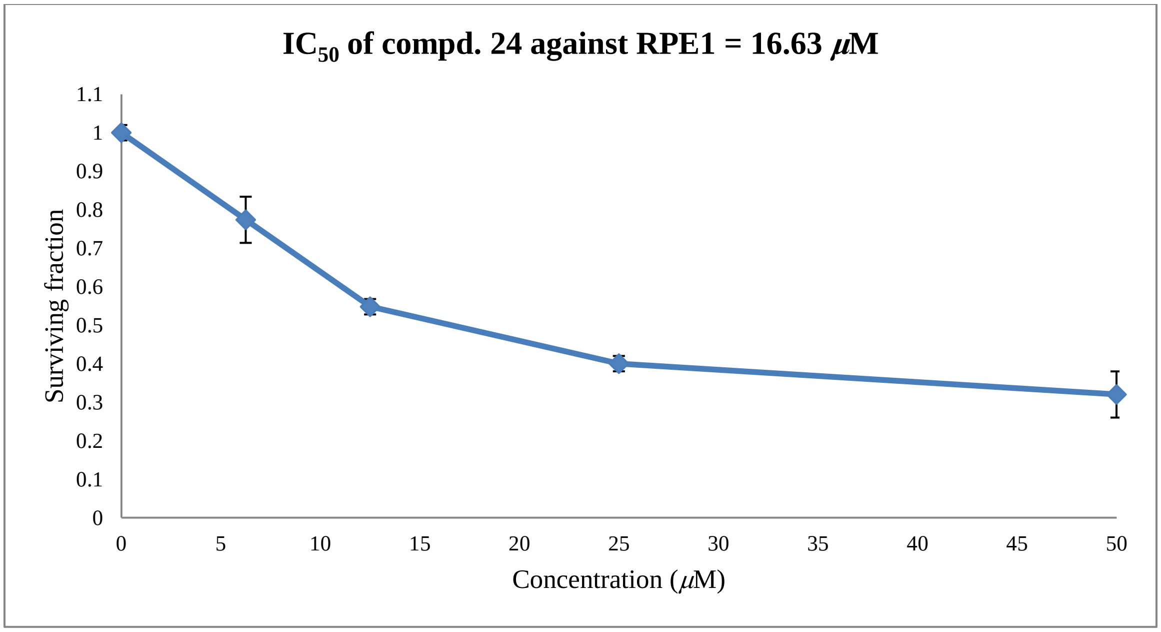
IC50 of compd. 24 against RPE1 = 16.63 μM
1.1
1
0.9
0.8
0.7
0.6
0.5
0.4
0.3
0.2
0.1
0
Surviving fraction
0
5
10
15
20
25
30
35
40
45
50
Concentration (μM)

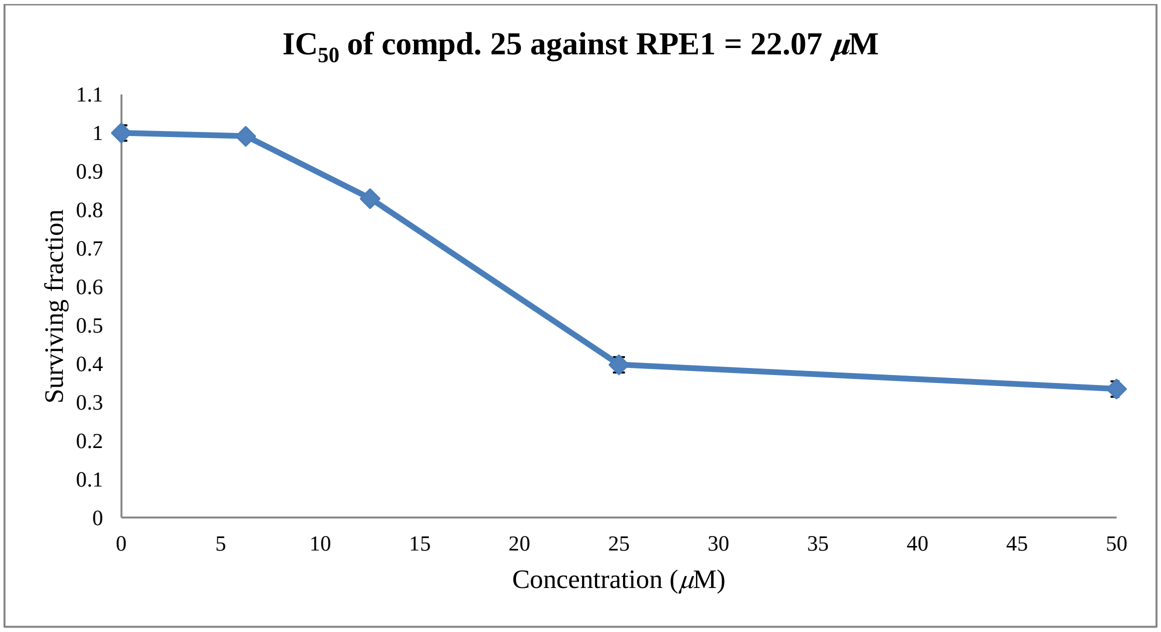
IC50 of compd. 25 against RPE1 = 22.07 μM
1.1
1
0.9
0.8
0.7
0.6
0.5
0.4
0.3
0.2
0.1
0
Surviving fraction
0
5
10
15
20
25
30
35
40
45
50
Concentration (μM)

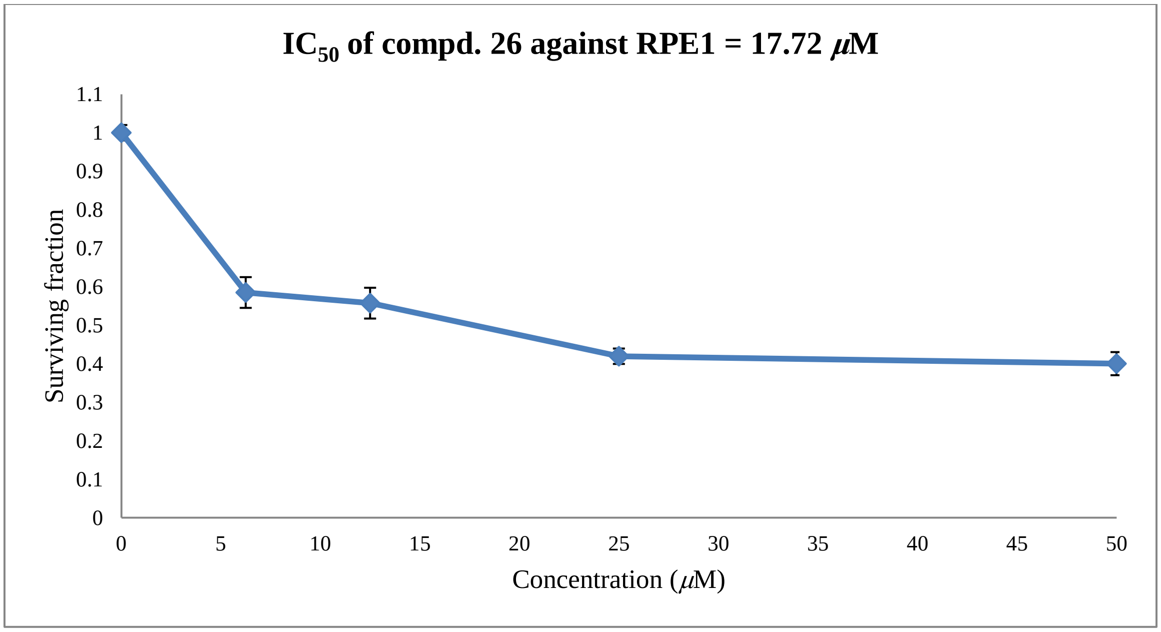
IC50 of compd. 26 against RPE1 = 17.72 μM
Surviving fraction
1.1
1
0.9
0.8
0.7
0.6
0.5
0.4
0.3
0.2
0.1
0
0
5
10
15
20
25
30
35
40
45
50
Concentration (μM)

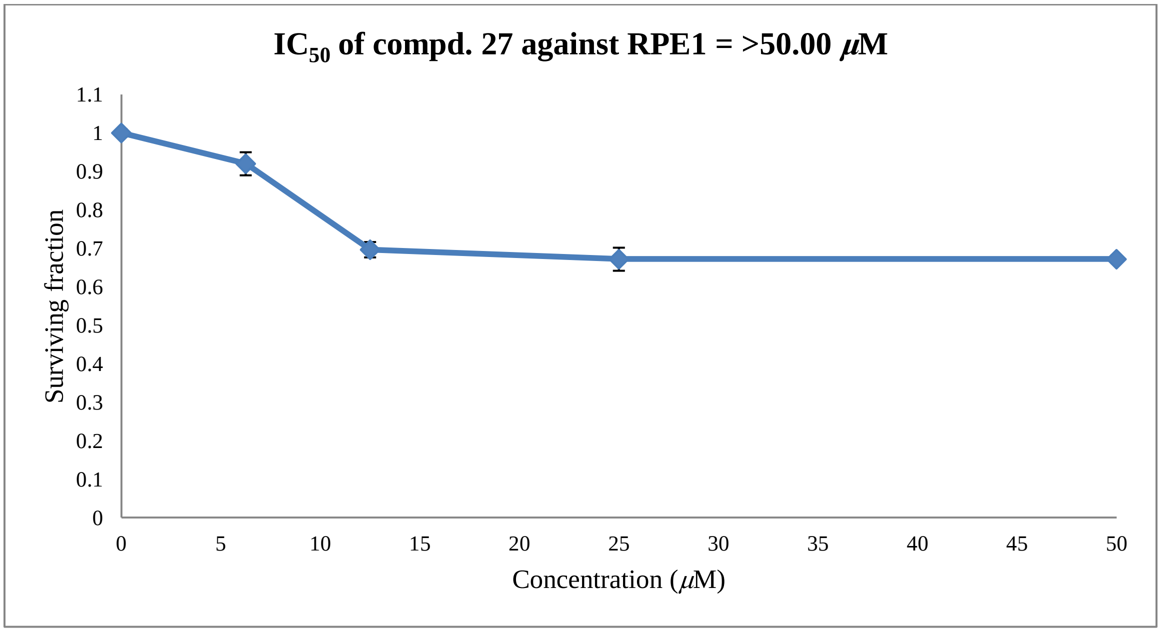
IC50 of compd. 27 against RPE1 = >50.00 μM
Surviving fraction
1.1
1
0.9
0.8
0.7
0.6
0.5
0.4
0.3
0.2
0.1
0
0
5
10
15
20
25
30
35
40
45
50
Concentration (μM)

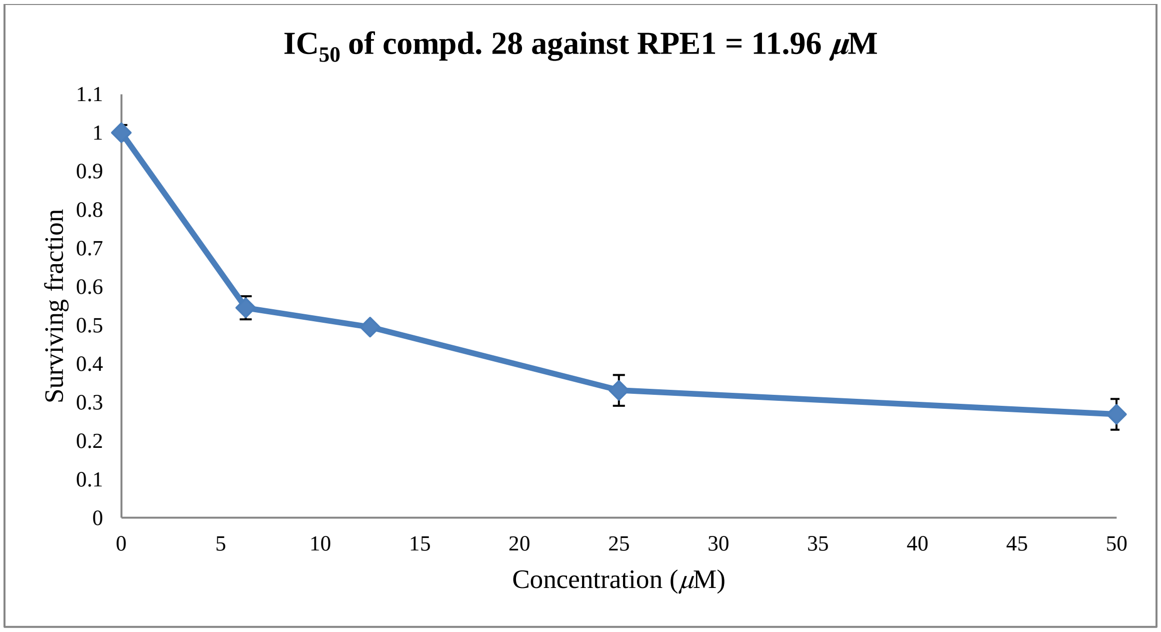
IC50 of compd. 28 against RPE1 = 11.96 μM
Surviving fraction
1.1
1
0.9
0.8
0.7
0.6
0.5
0.4
0.3
0.2
0.1
0
0
5
10
15
20
25
30
35
40
45
50
Concentration (μM)

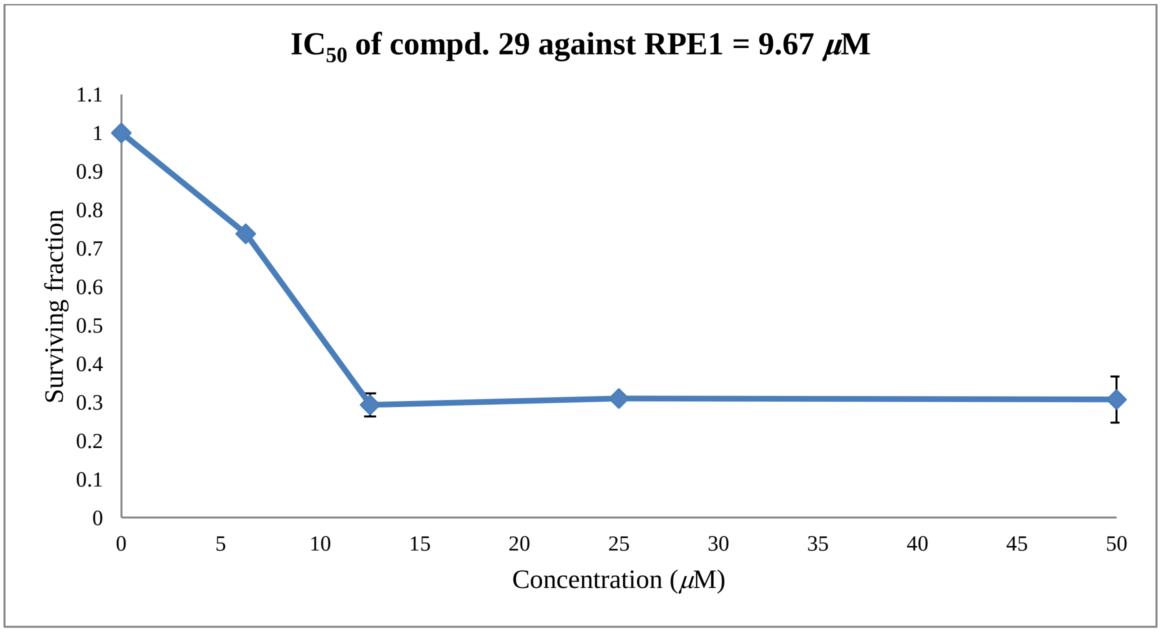
IC50 of compd. 29 against RPE1 = 9.67 μM
Surviving fraction
1.1
1
0.9
0.8
0.7
0.6
0.5
0.4
0.3
0.2
0.1
0
0
5
10
15
20
25
30
35
40
45
50
Concentration (μM)

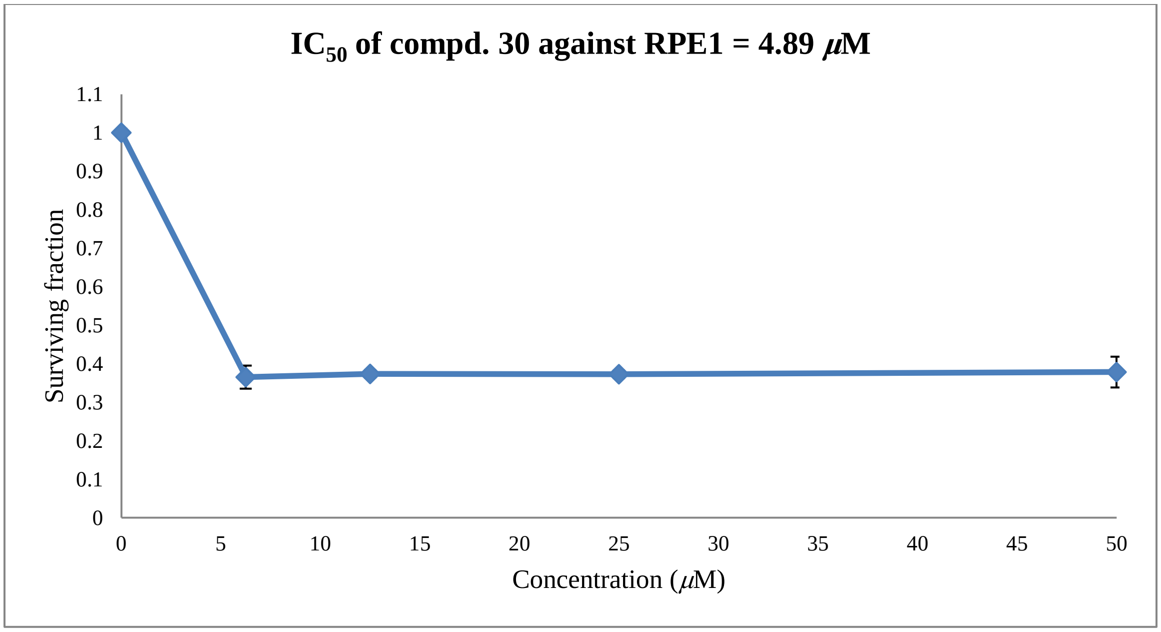
IC50 of compd. 30 against RPE1 = 4.89 μM
Surviving fraction
1.1
1
0.9
0.8
0.7
0.6
0.5
0.4
0.3
0.2
0.1
0
0
5
10
15
20
25
30
35
40
45
50
Concentration (μM)

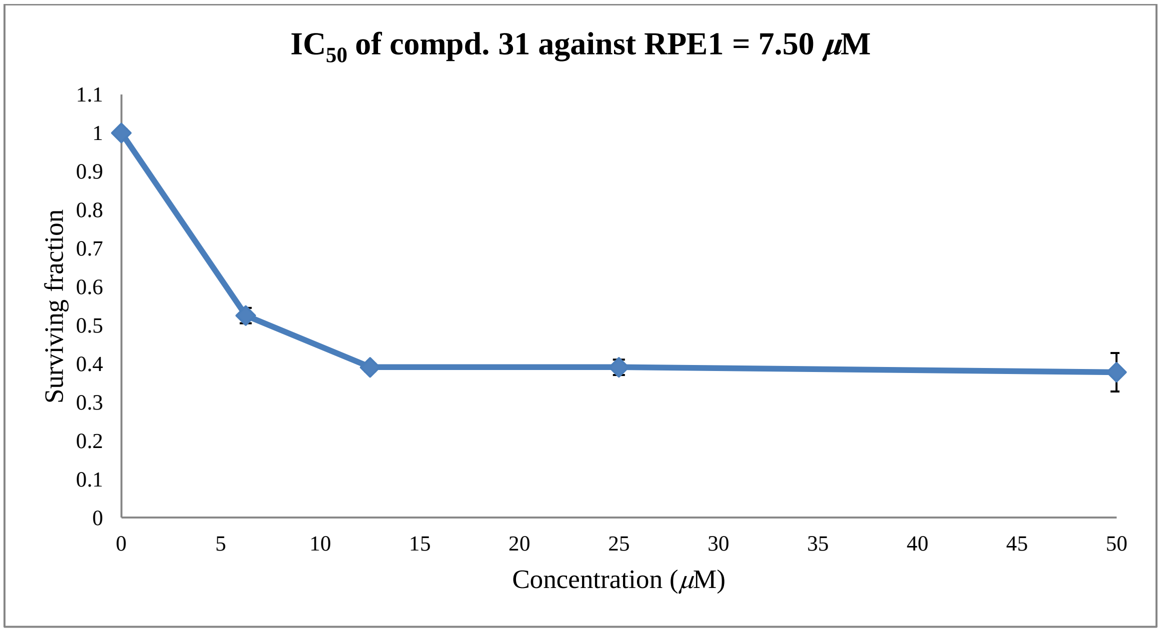
IC50 of compd. 31 against RPE1 = 7.50 μM
Surviving fraction
1.1
1
0.9
0.8
0.7
0.6
0.5
0.4
0.3
0.2
0.1
0
0
5
10
15
20
25
30
35
40
45
50
Concentration (μM)

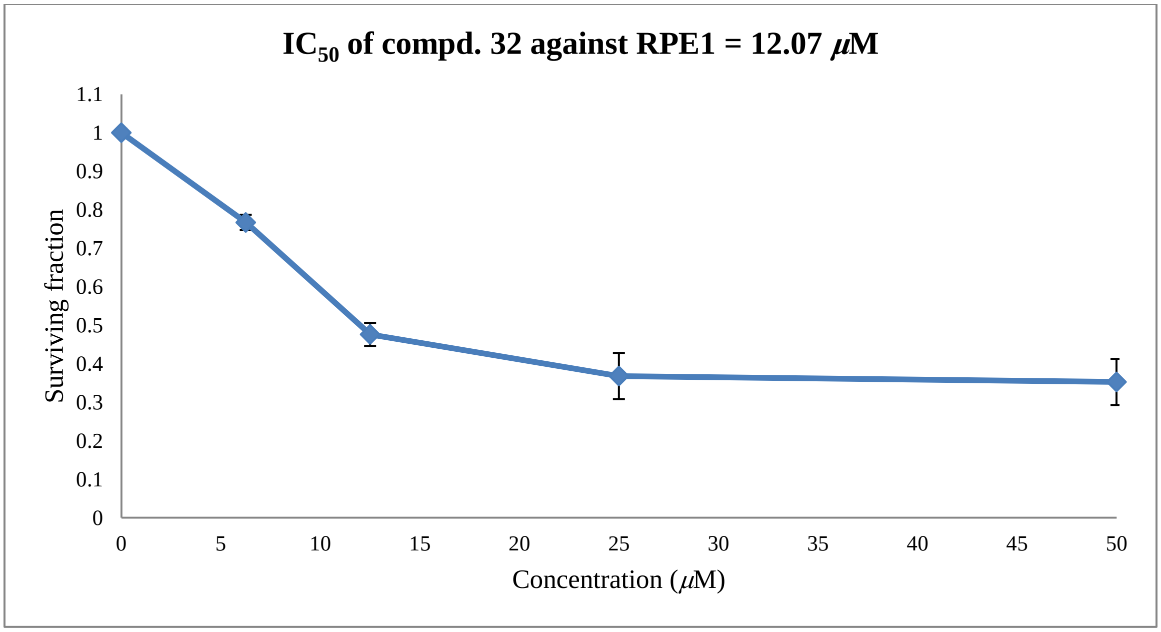
IC50 of compd. 32 against RPE1 = 12.07 μM
Surviving fraction
1.1
1
0.9
0.8
0.7
0.6
0.5
0.4
0.3
0.2
0.1
0
0 5 10 15 20 25 30 35 40 45 50
Concentration (μM)

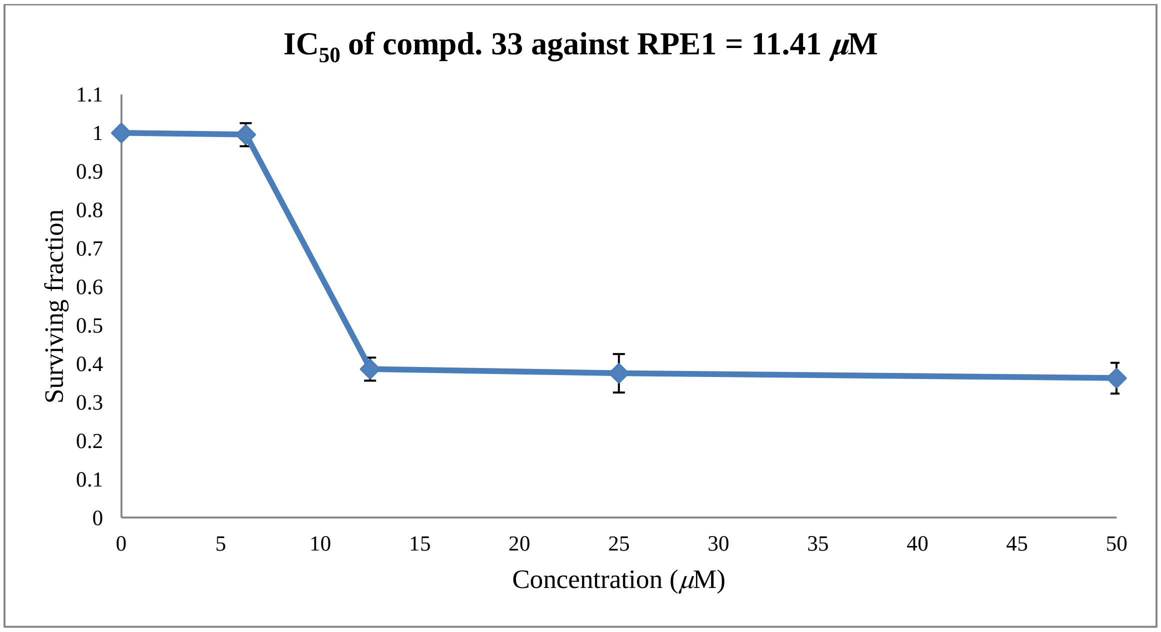
IC50 of compd. 33 against RPE1 = 11.41 μM
Surviving fraction
1.1
1
0.9
0.8
0.7
0.6
0.5
0.4
0.3
0.2
0.1
0
0 5 10 15 20 25 30 35 40 45 50
Concentration (μM)

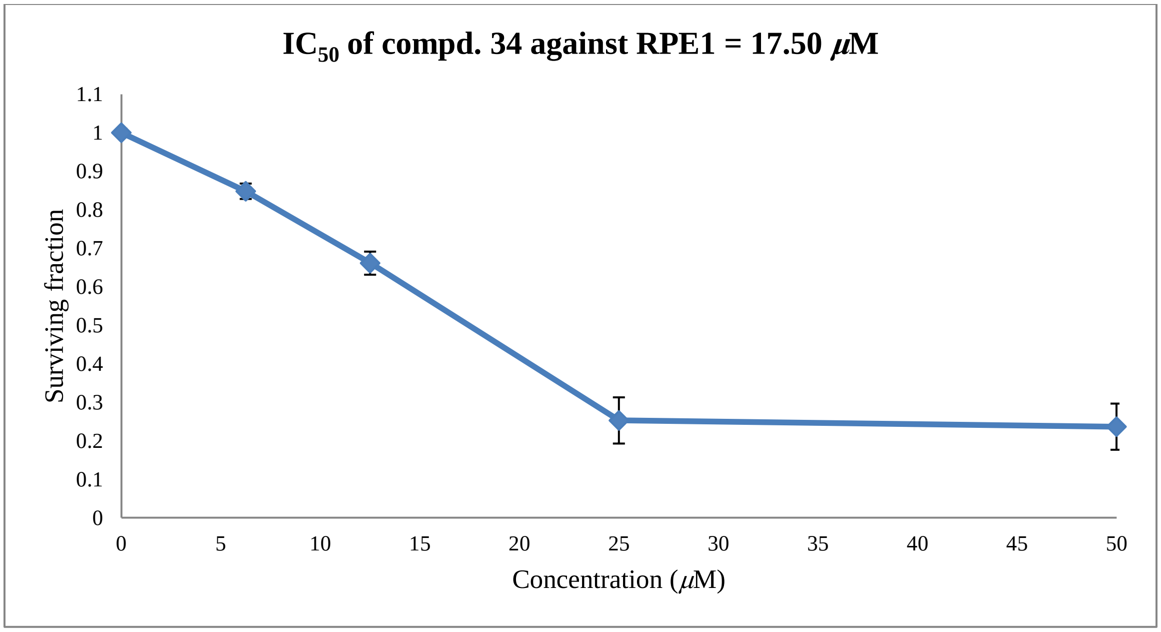
IC50 of compd. 34 against RPE1 = 17.50 μM
Surviving fraction
1.1
1
0.9
0.8
0.7
0.6
0.5
0.4
0.3
0.2
0.1
0
0
5
10
15
20
25
30
35
40
45
50
Concentration (μM)

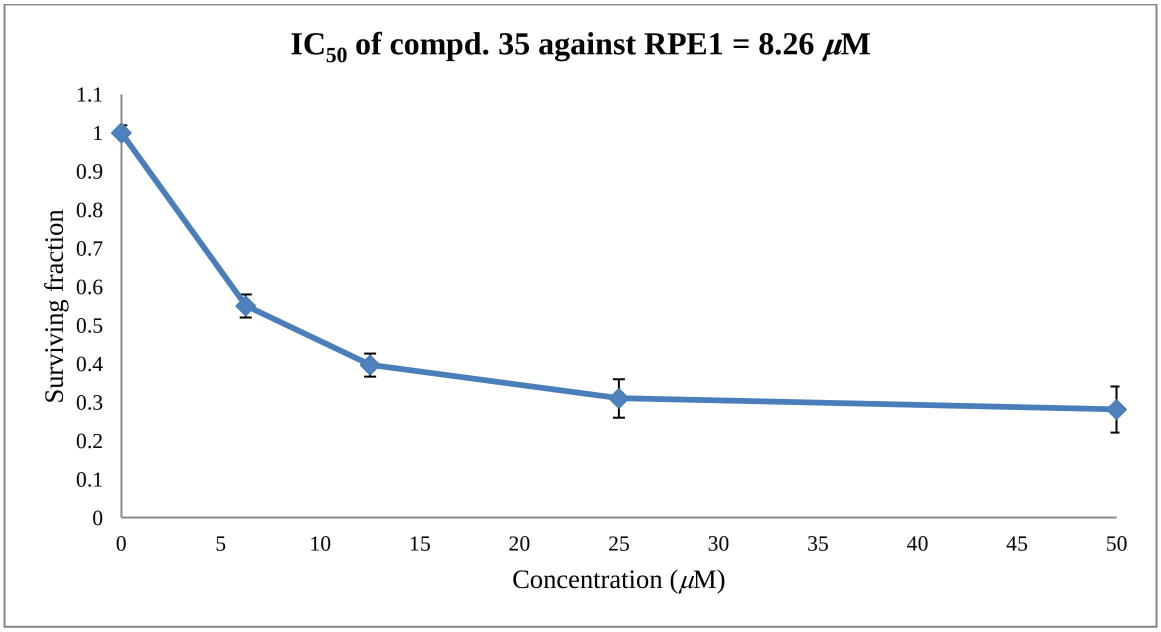
IC50 of compd. 35 against RPE1 = 8.26 μM
Surviving fraction
1.1
1
0.9
0.8
0.7
0.6
0.5
0.4
0.3
0.2
0.1
0
0
5
10
15
20
25
30
35
40
45
50
Concentration (μM)

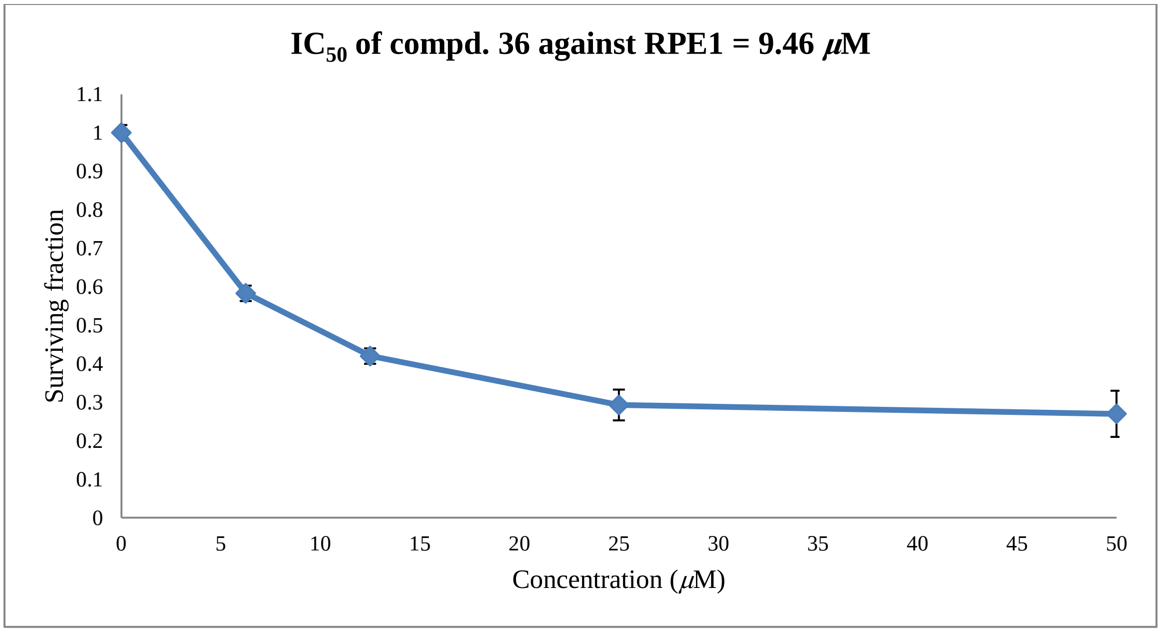
IC50 of compd. 36 against RPE1 = 9.46 μM
Surviving fraction
1.1
1
0.9
0.8
0.7
0.6
0.5
0.4
0.3
0.2
0.1
0
0
5
10
15
20
25
30
35
40
45
50
Concentration (μM)

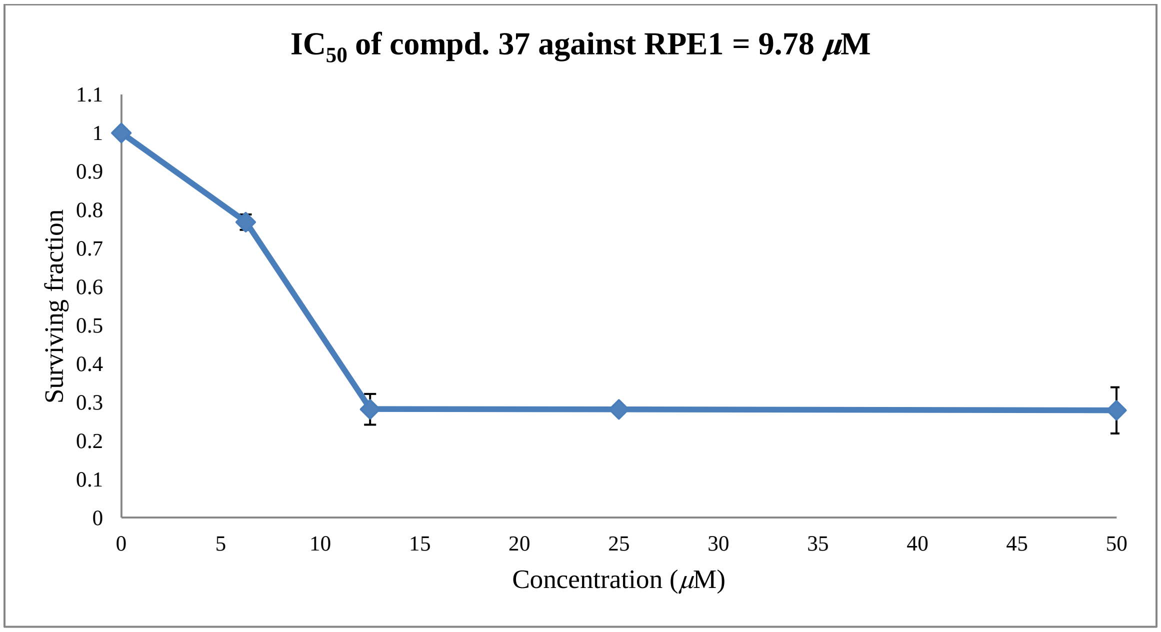
IC50 of compd. 37 against RPE1 = 9.78 μM
Surviving fraction
1.1
1
0.9
0.8
0.7
0.6
0.5
0.4
0.3
0.2
0.1
0
0
5
10
15
20
25
30
35
40
45
50
Concentration (μM)

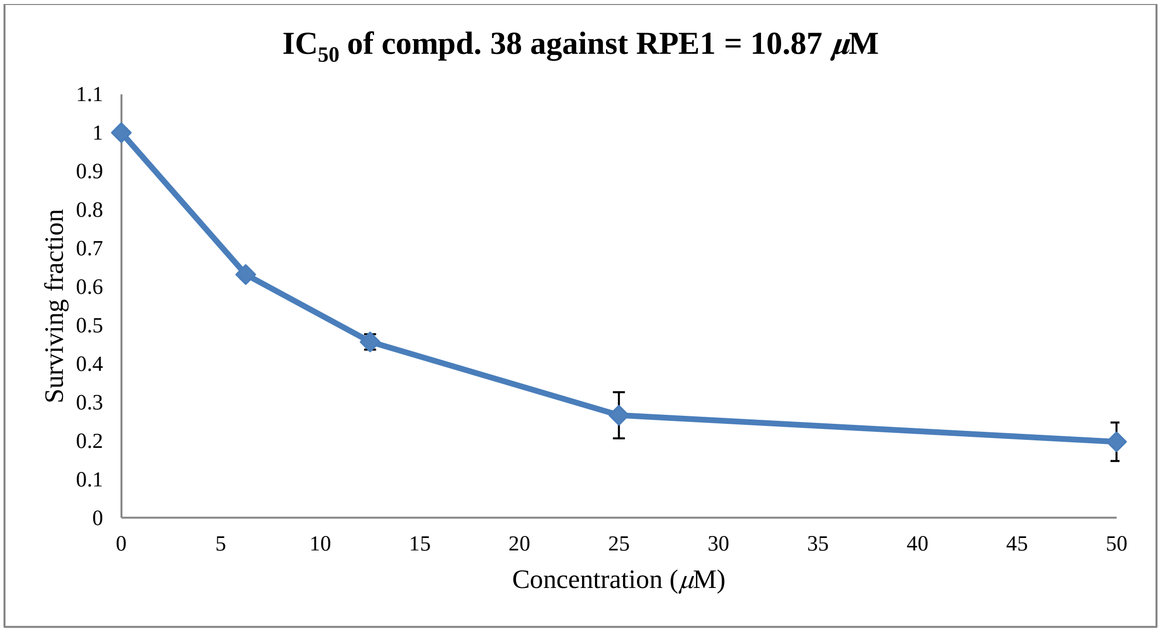
IC50 of compd. 38 against RPE1 = 10.87 μM
Surviving fraction
1.1
1
0.9
0.8
0.7
0.6
0.5
0.4
0.3
0.2
0.1
0
0
5
10
15
20
25
30
35
40
45
50
Concentration (μM)

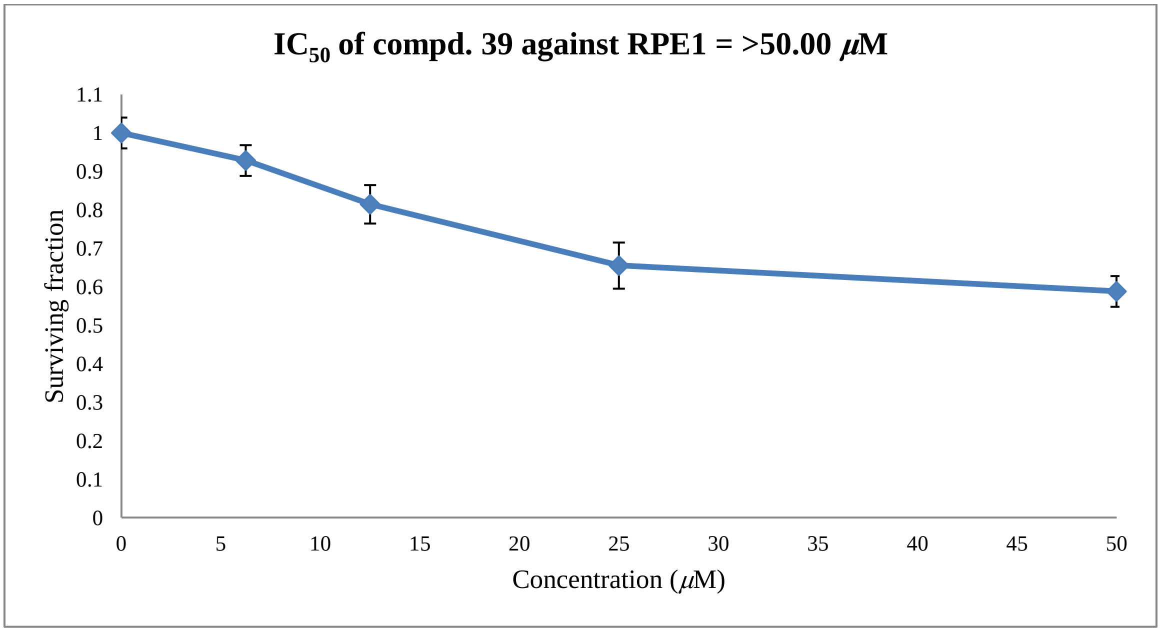
IC50 of compd. 39 against RPE1 = >50.00 μM
Surviving fraction
1.1
1
0.9
0.8
0.7
0.6
0.5
0.4
0.3
0.2
0.1
0
0
5
10
15
20
25
30
35
40
45
50
Concentration (μM)

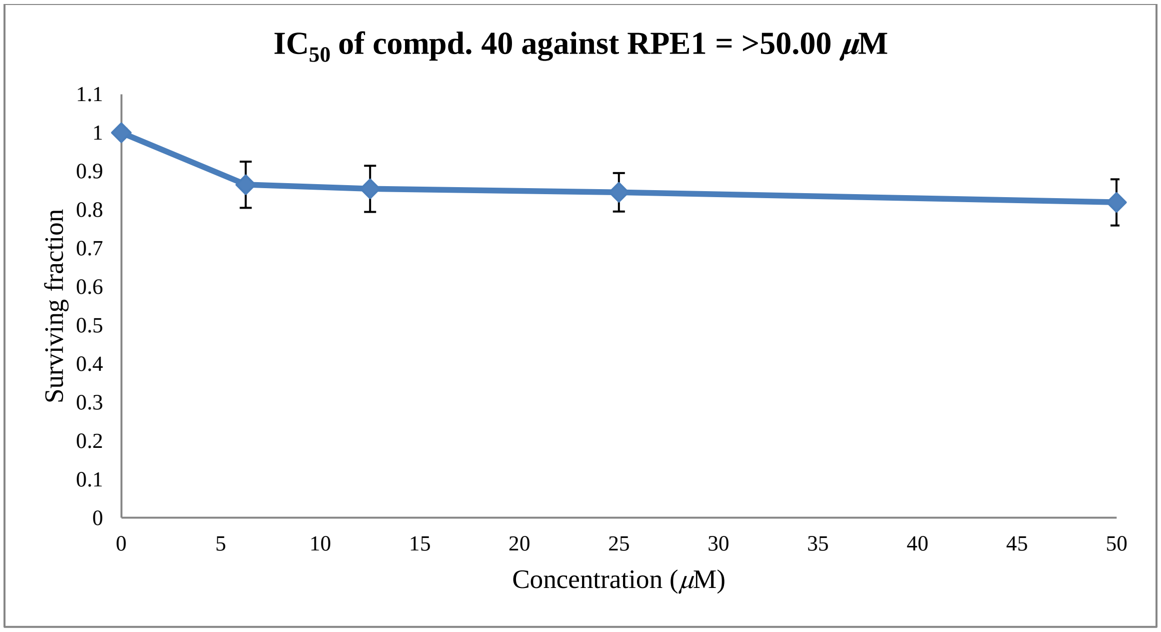
IC50 of compd. 40 against RPE1 = >50.00 μM
Surviving fraction
1.1
1
0.9
0.8
0.7
0.6
0.5
0.4
0.3
0.2
0.1
0
0
5
10
15
20
25
30
35
40
45
50
Concentration (μM)

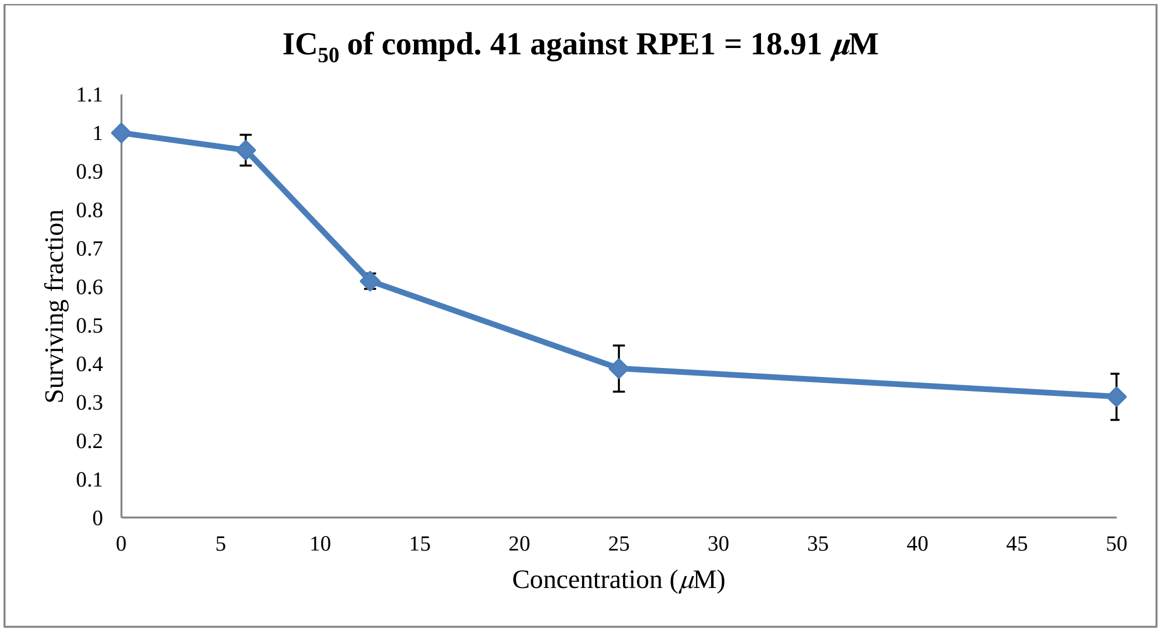
IC50 of compd. 41 against RPE1 = 18.91 μM
Surviving fraction
1.1
1
0.9
0.8
0.7
0.6
0.5
0.4
0.3
0.2
0.1
0
0
5
10
15
20
25
30
35
40
45
50
Concentration (μM)

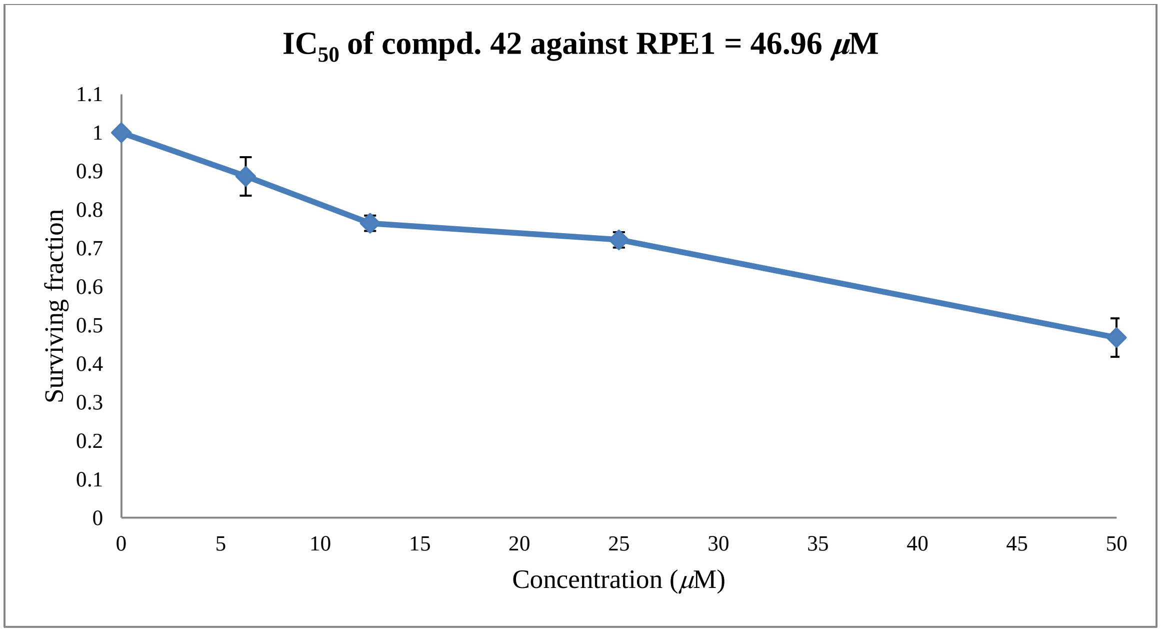
IC$_{50}$ of compd. 42 against RPE1 = 46.96 μM
Surviving fraction
Concentration (μM)
0
0.1
0.2
0.3
0.4
0.5
0.6
0.7
0.8
0.9
1
1.1
0
5
10
15
20
25
30
35
40
45
50

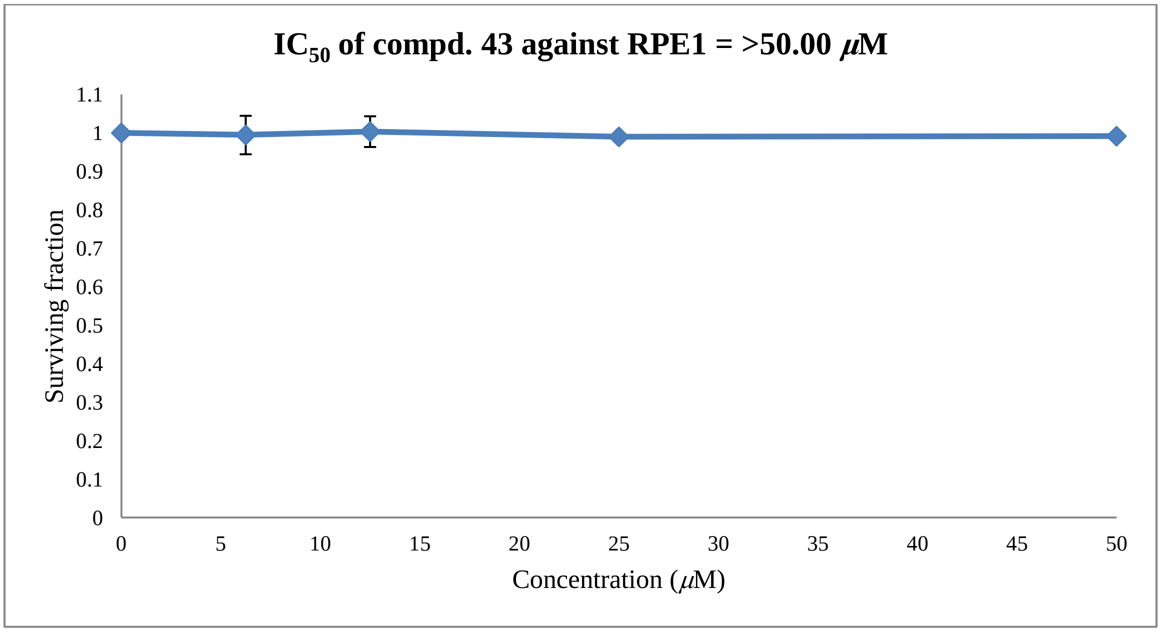
IC$_{50}$ of compd. 43 against RPE1 = >50.00 μM
Surviving fraction
Concentration (μM)
0
0.1
0.2
0.3
0.4
0.5
0.6
0.7
0.8
0.9
1
1.1
0
5
10
15
20
25
30
35
40
45
50

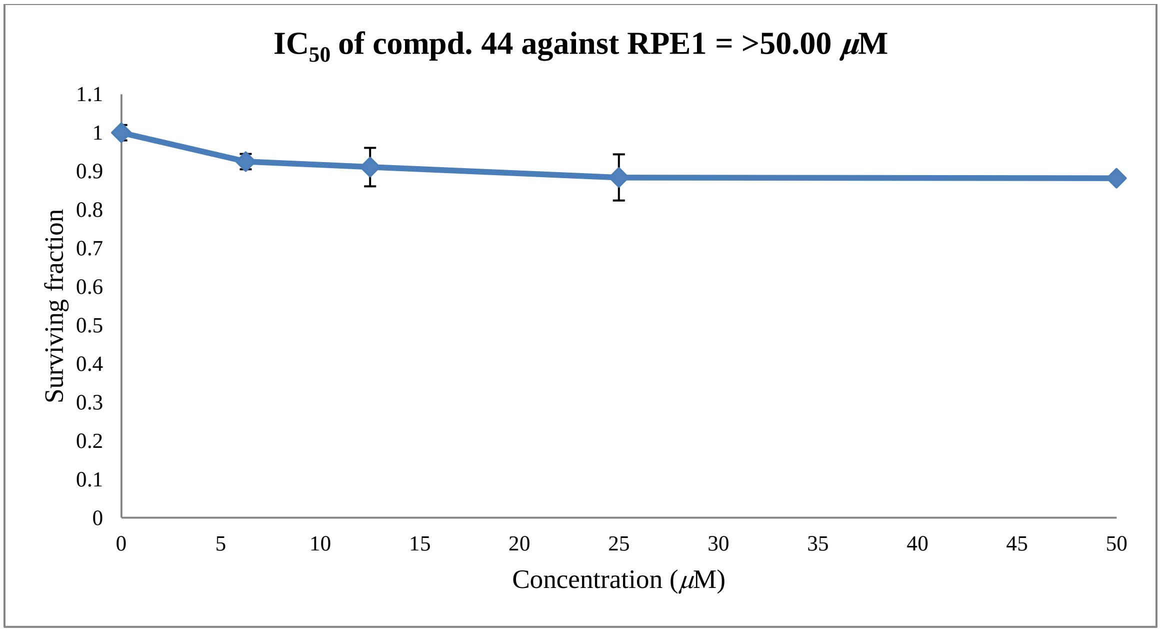
IC50 of compd. 44 against RPE1 = >50.00 μM
Surviving fraction
1.1
1
0.9
0.8
0.7
0.6
0.5
0.4
0.3
0.2
0.1
0
0
5
10
15
20
25
30
35
40
45
50
Concentration (μM)

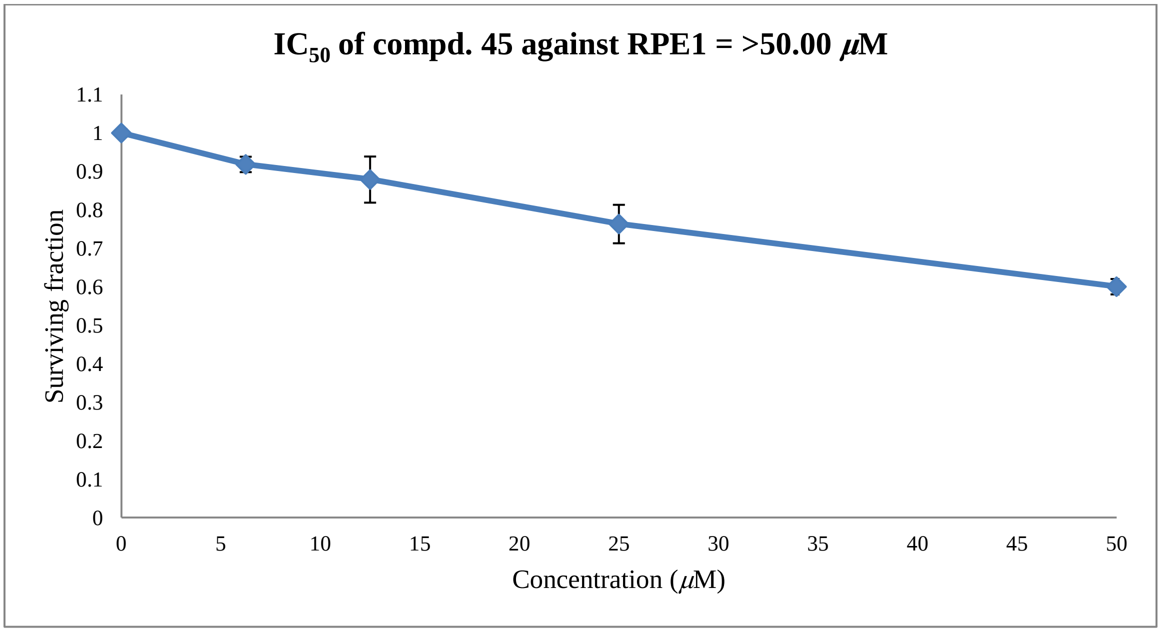
IC50 of compd. 45 against RPE1 = >50.00 μM
Surviving fraction
1.1
1
0.9
0.8
0.7
0.6
0.5
0.4
0.3
0.2
0.1
0
0
5
10
15
20
25
30
35
40
45
50
Concentration (μM)

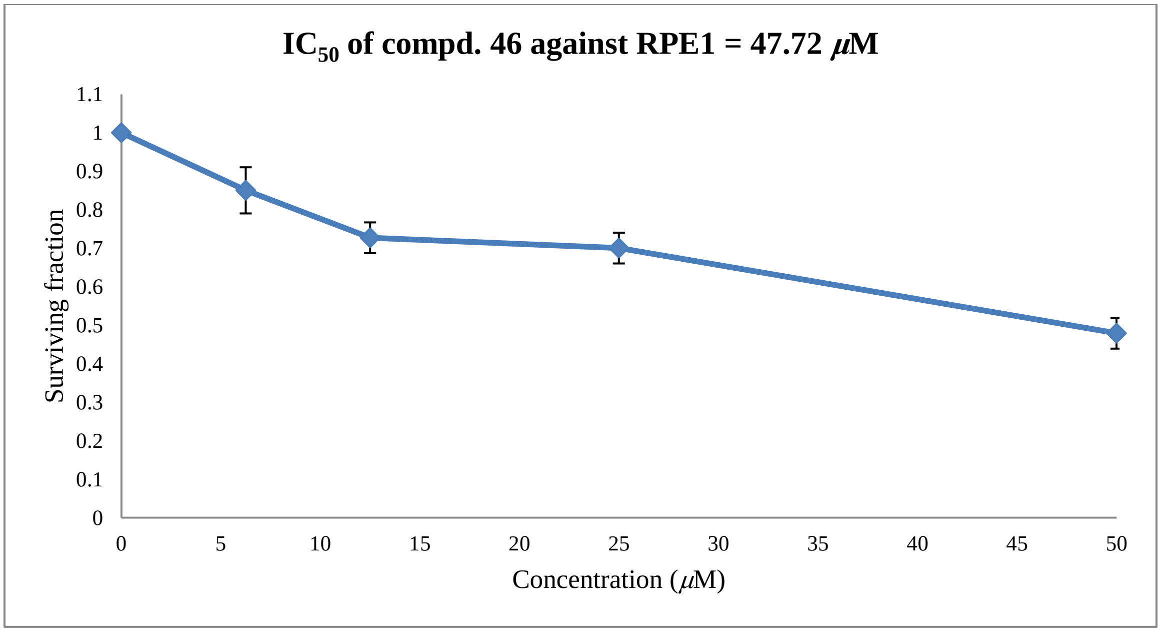
IC50 of compd. 46 against RPE1 = 47.72 μM
Surviving fraction
Concentration (μM)
1.1
1
0.9
0.8
0.7
0.6
0.5
0.4
0.3
0.2
0.1
0
0
5
10
15
20
25
30
35
40
45
50

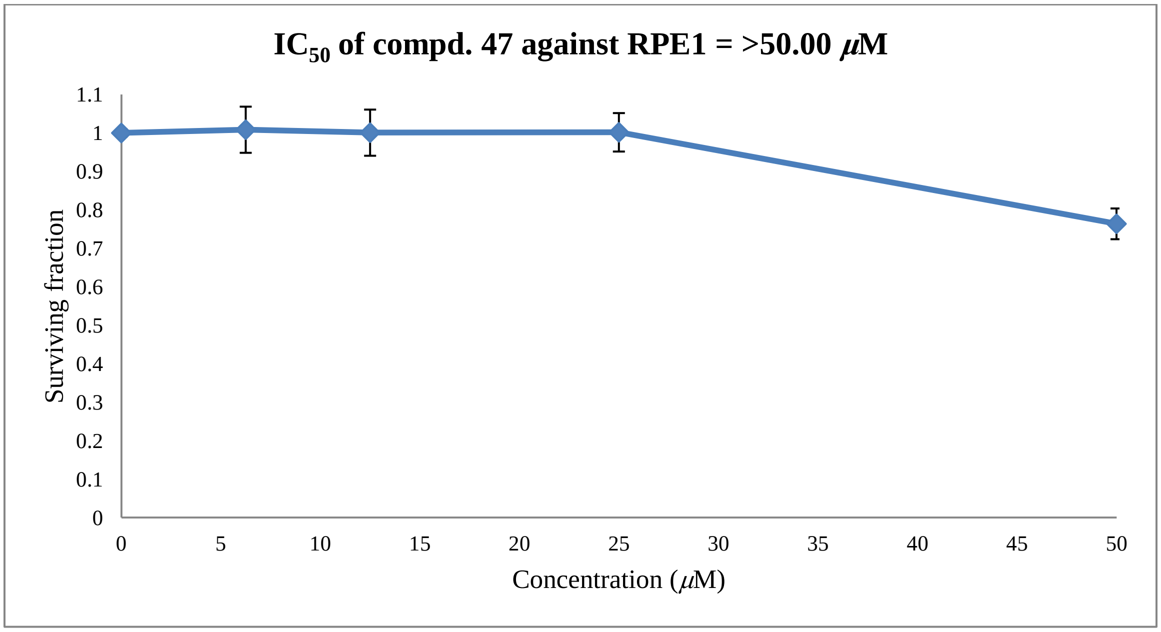
IC50 of compd. 47 against RPE1 = >50.00 μM
Surviving fraction
Concentration (μM)
1.1
1
0.9
0.8
0.7
0.6
0.5
0.4
0.3
0.2
0.1
0
0
5
10
15
20
25
30
35
40
45
50

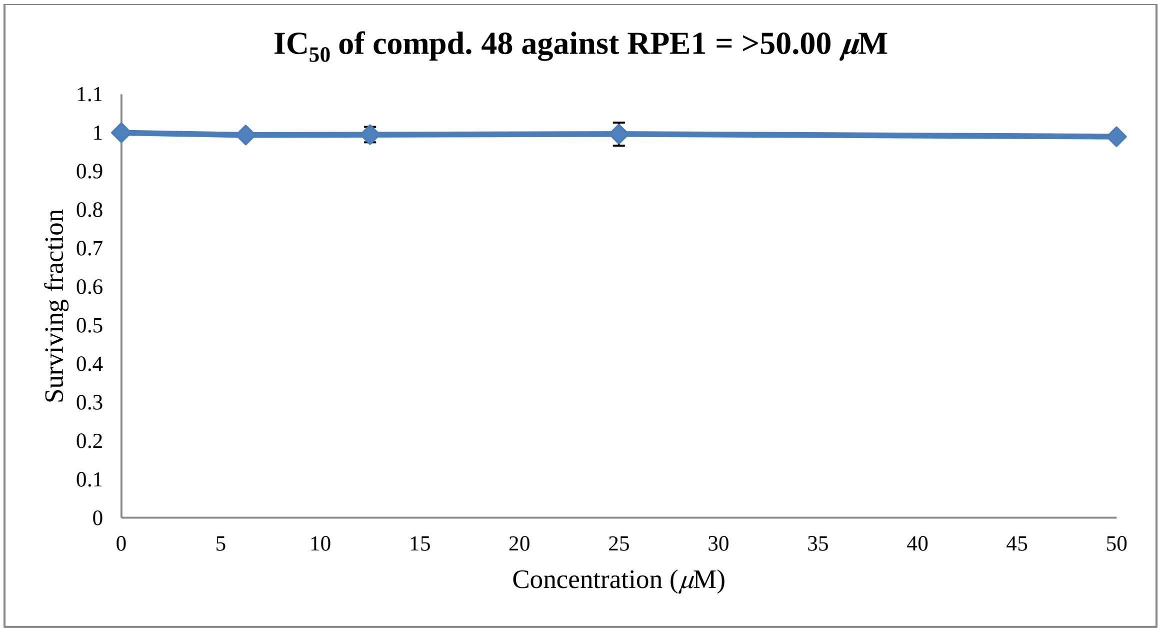
IC$_{50}$ of compd. 48 against RPE1 = >50.00 μM
Surviving fraction
1.1
1
0.9
0.8
0.7
0.6
0.5
0.4
0.3
0.2
0.1
0
0
5
10
15
20
25
30
35
40
45
50
Concentration (μM)

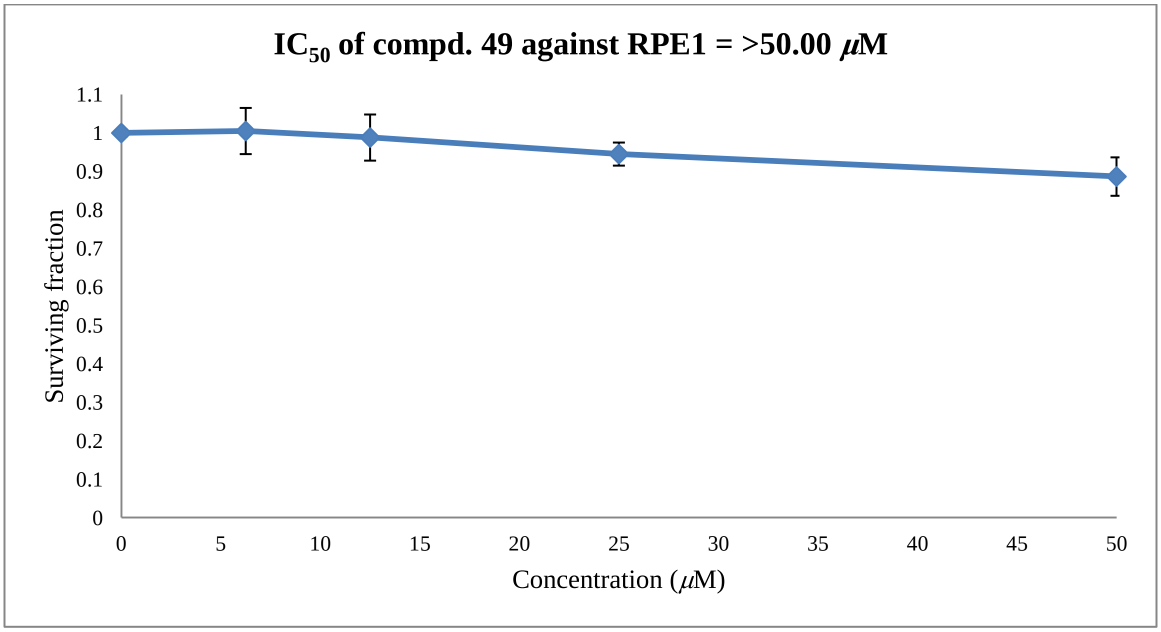
IC$_{50}$ of compd. 49 against RPE1 = >50.00 μM
Surviving fraction
1.1
1
0.9
0.8
0.7
0.6
0.5
0.4
0.3
0.2
0.1
0
0
5
10
15
20
25
30
35
40
45
50
Concentration (μM)

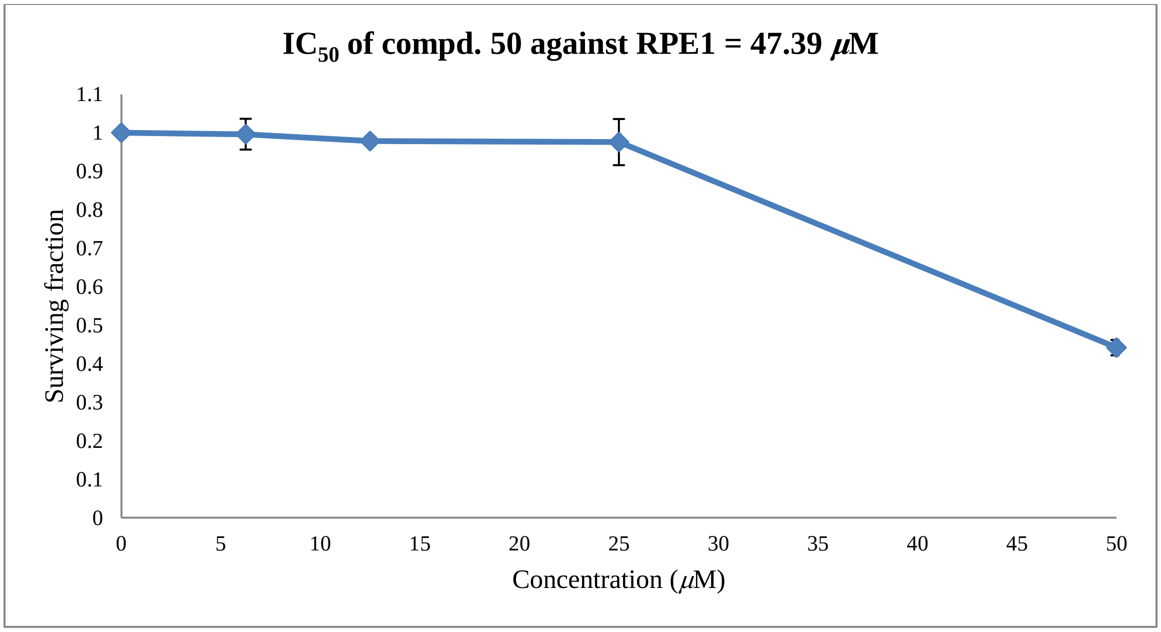

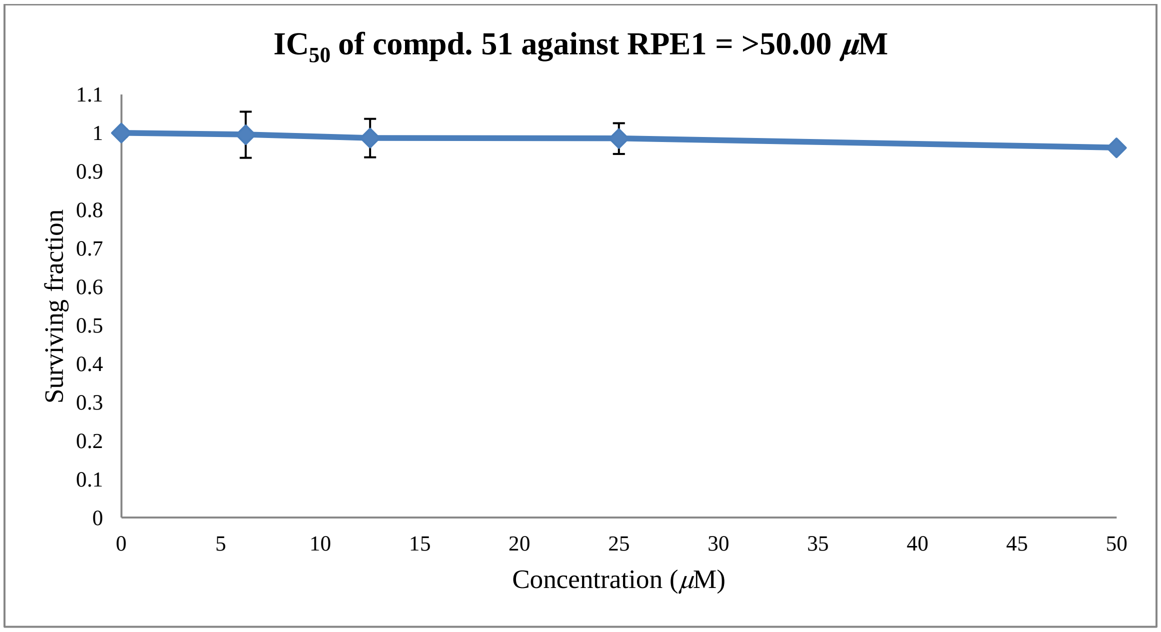

Fig. S96. Dose-response curve for the tested compounds against RPE1 (retinal pigment epithelium) cell line.

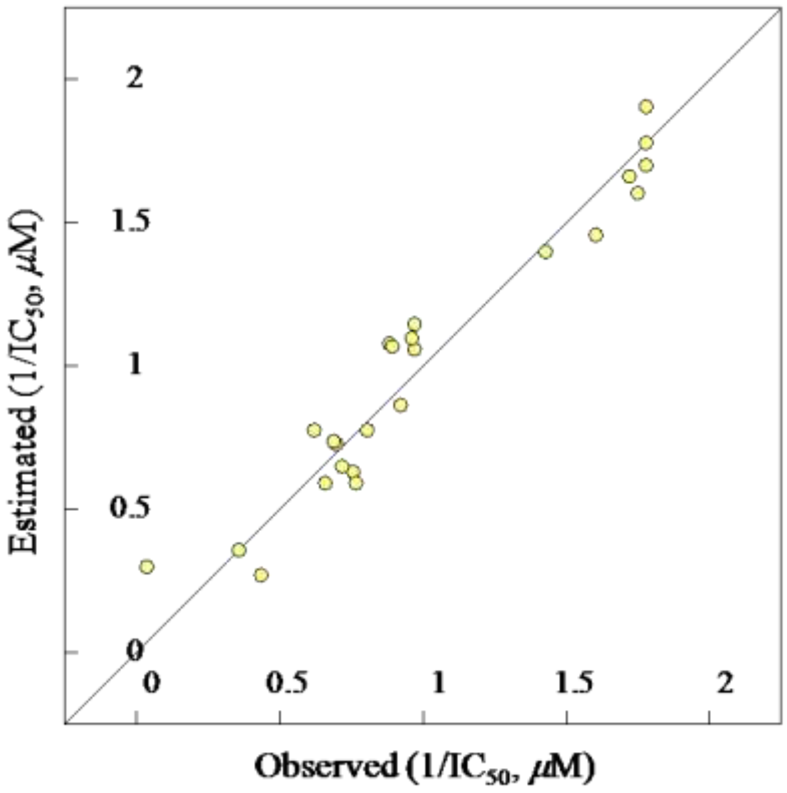

Fig. S97. BMLR-QSAR model plot of correlations representing the observed *vs.* predicted 1/IC_{50}, μM values for the tested compounds against HCT116 (colon) carcinoma cell line.

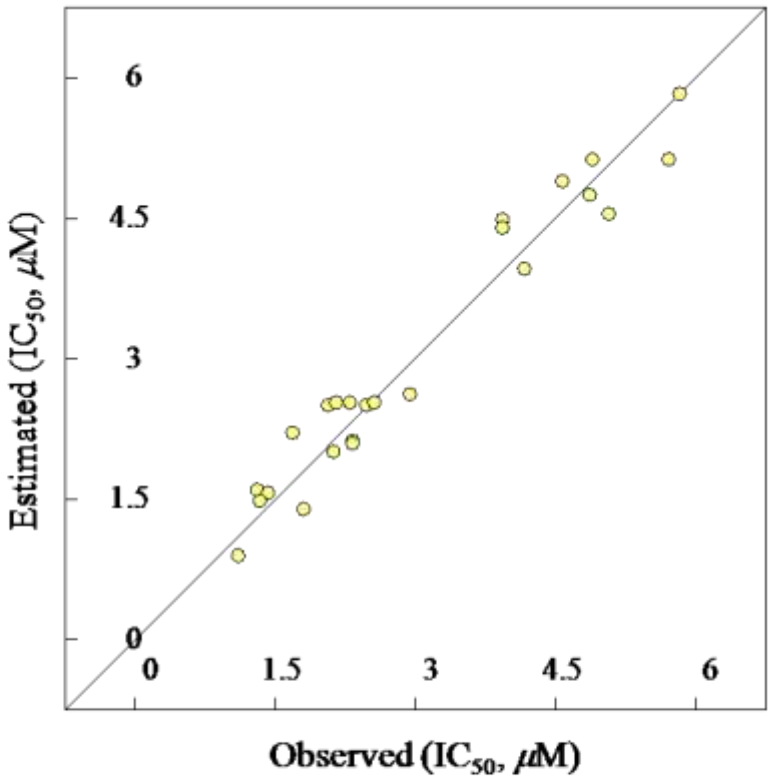

Fig. S98. BMLR-QSAR model plot of correlations representing the observed *vs.* predicted IC_{50}, μM values for the tested compounds against MCF7 (breast) carcinoma cell line.

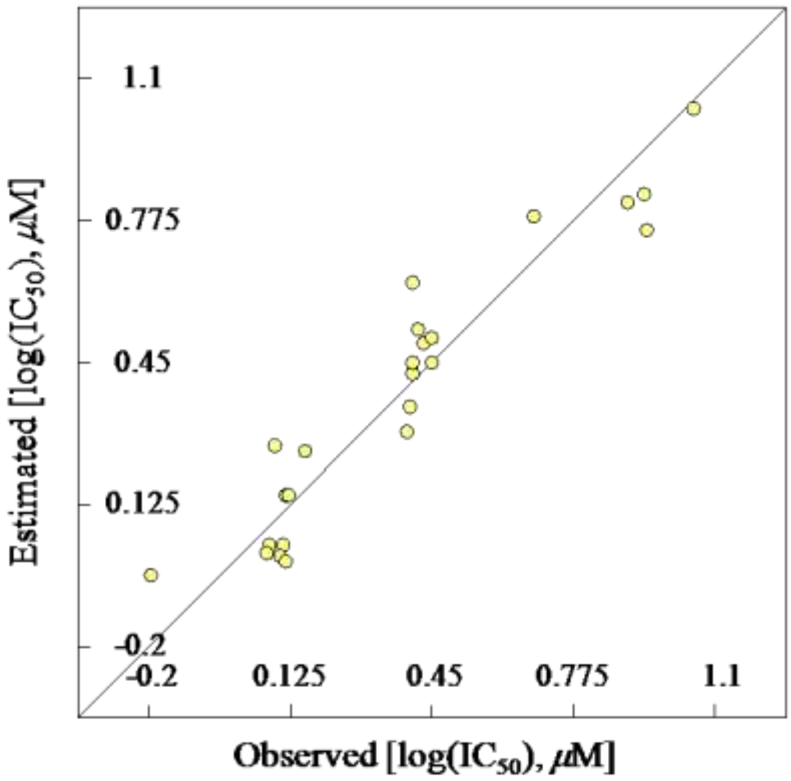

Fig. S99. BMLR-QSAR model plot of correlations representing the observed *vs.* predicted log(IC$_{50}$), μM values for the tested compounds against A431 (squamous) carcinoma cell line.

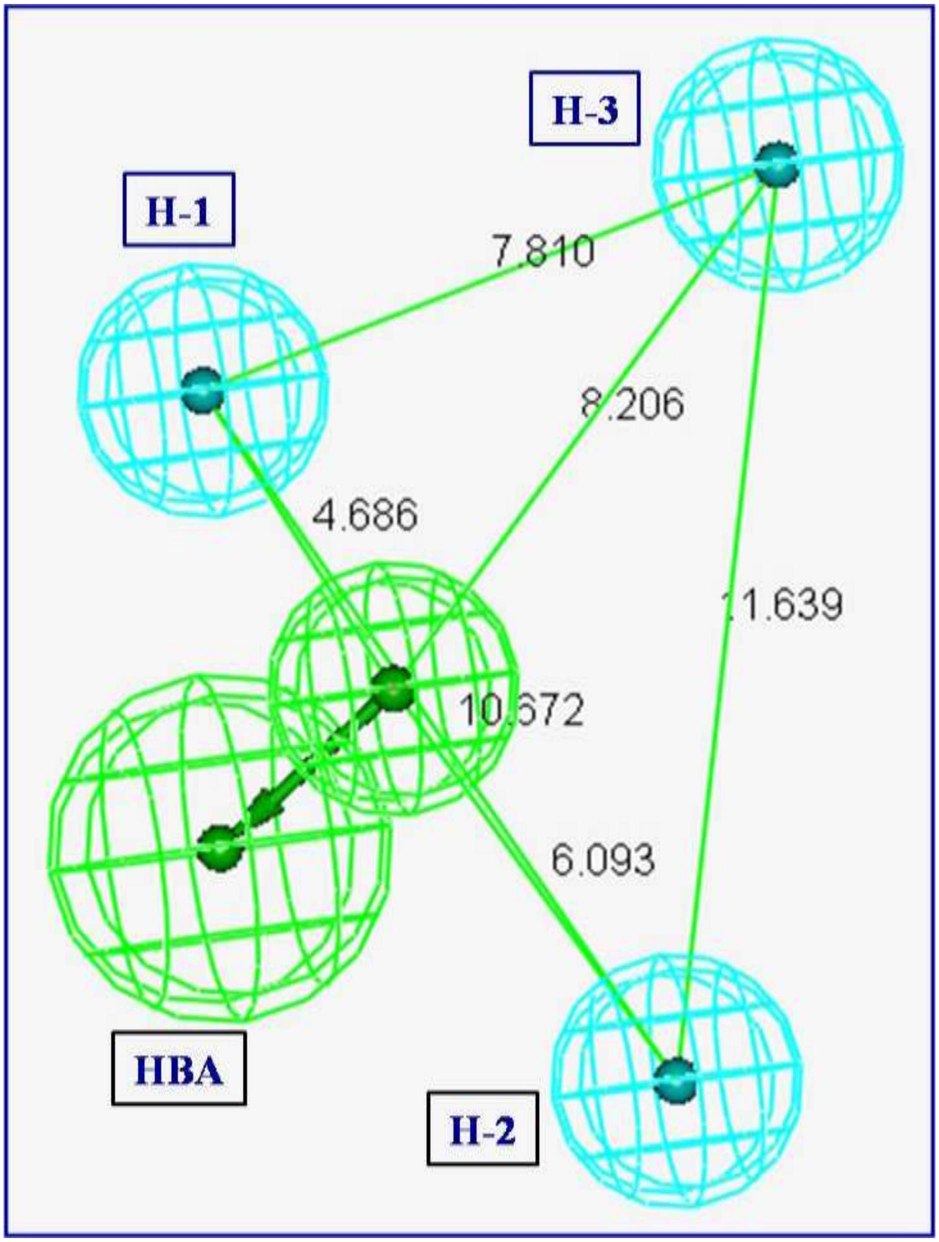

Fig. S100. Constraint distances "H-1 – H-2 = 10.672, H-1 – H-3 = 7.810, H-2 – H-3 = 11.639, H-1 – HBA = 4.686, H-2 – HBA = 6.093, H-3 – HBA = 8.206 Å" of the generated 3D-pharmacophore for the tested piperidinecarboxamides **24–47** against HCT116 (colon) carcinoma cell line which contains three hydrophobics (H-1, H-2, H-3; light blue) and one hydrogen bonding acceptor (HBA; green).

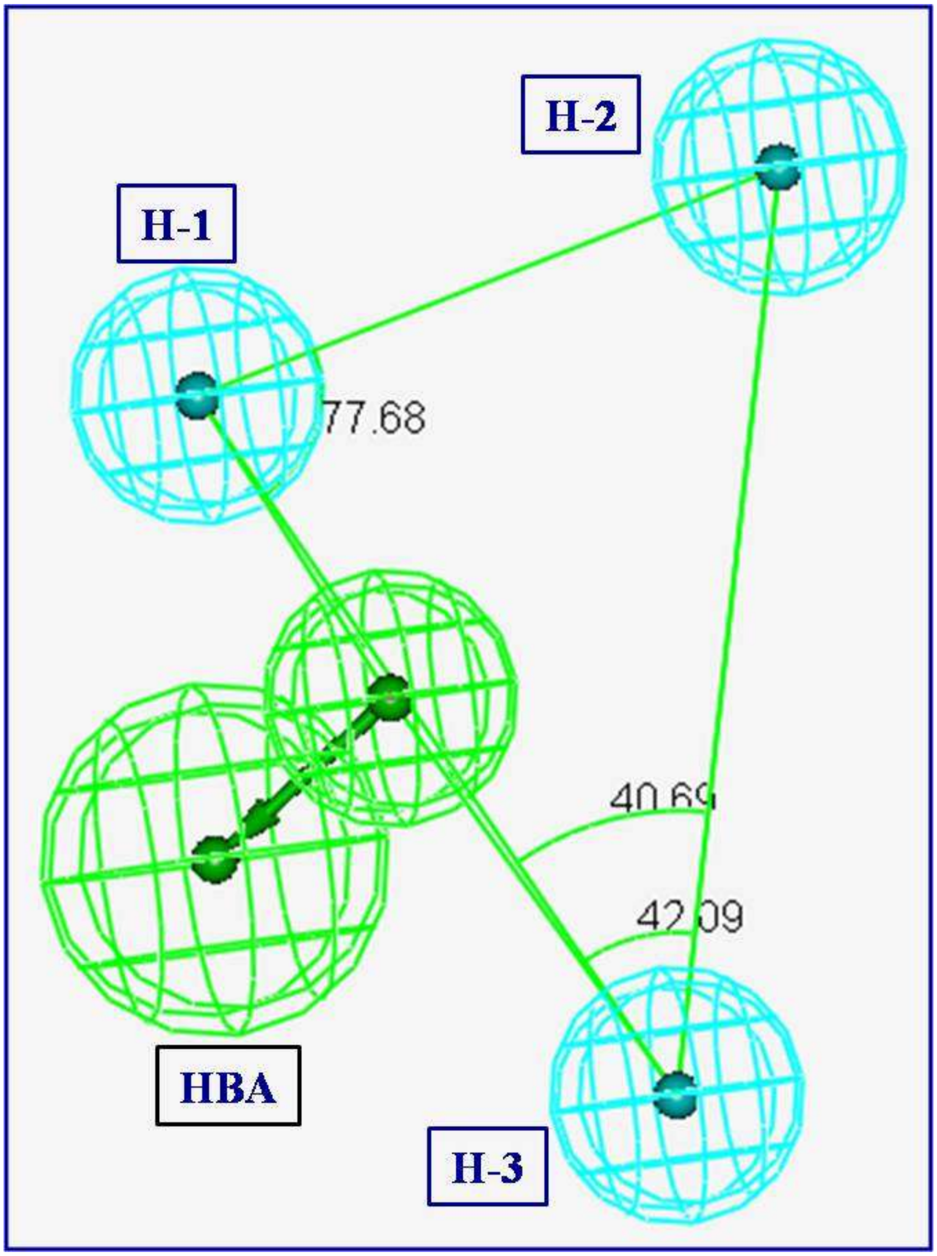

Fig. S101. Constraint angles "H-1 – H-2 – H-3 = 40.69, H-3 – H-1 – HBA = 77.68, H-3 – H-2 – HBA = 42.09 °" of the generated 3D-pharmacophore for the tested piperidinecarboxamides **24–47** against HCT116 (colon) carcinoma cell line which contains three hydrophobics (H-1, H-2, H-3; light blue) and one hydrogen bonding acceptor (HBA; green).

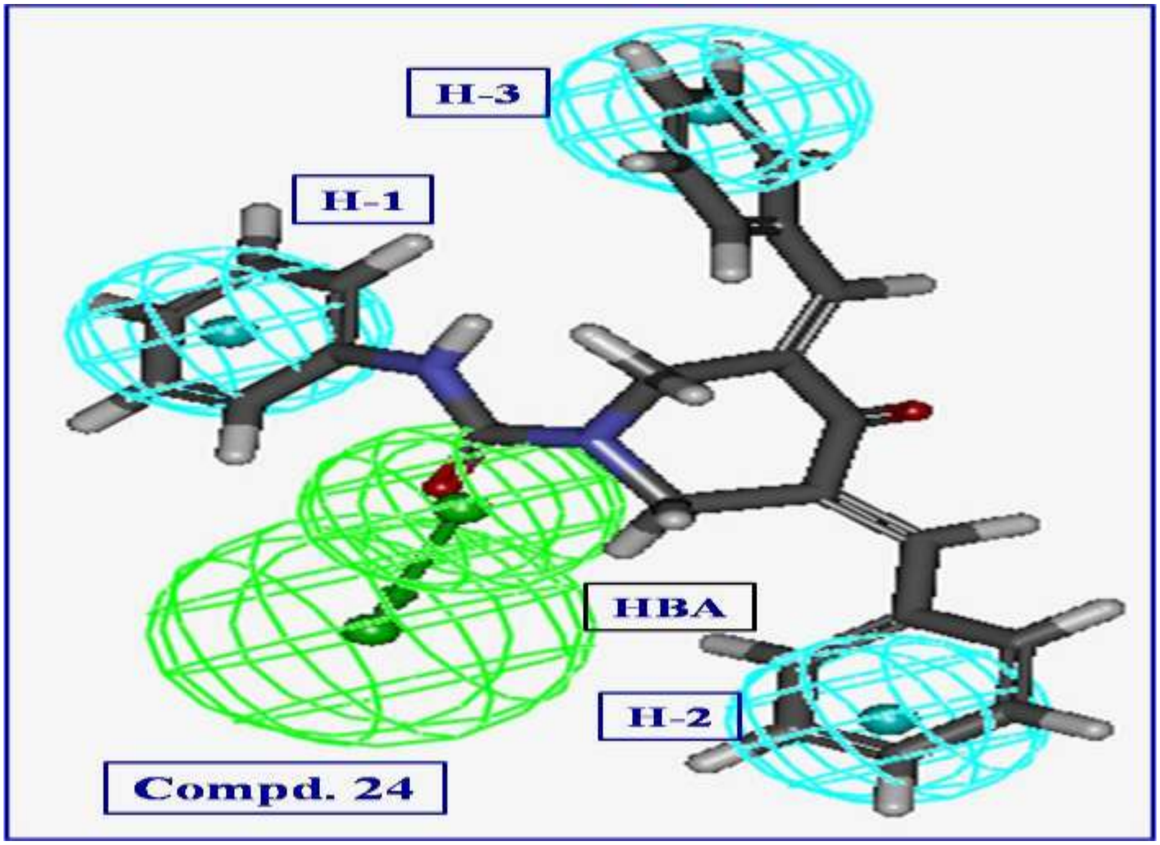
H-3
H-1
HBA
H-2
Compd. 24

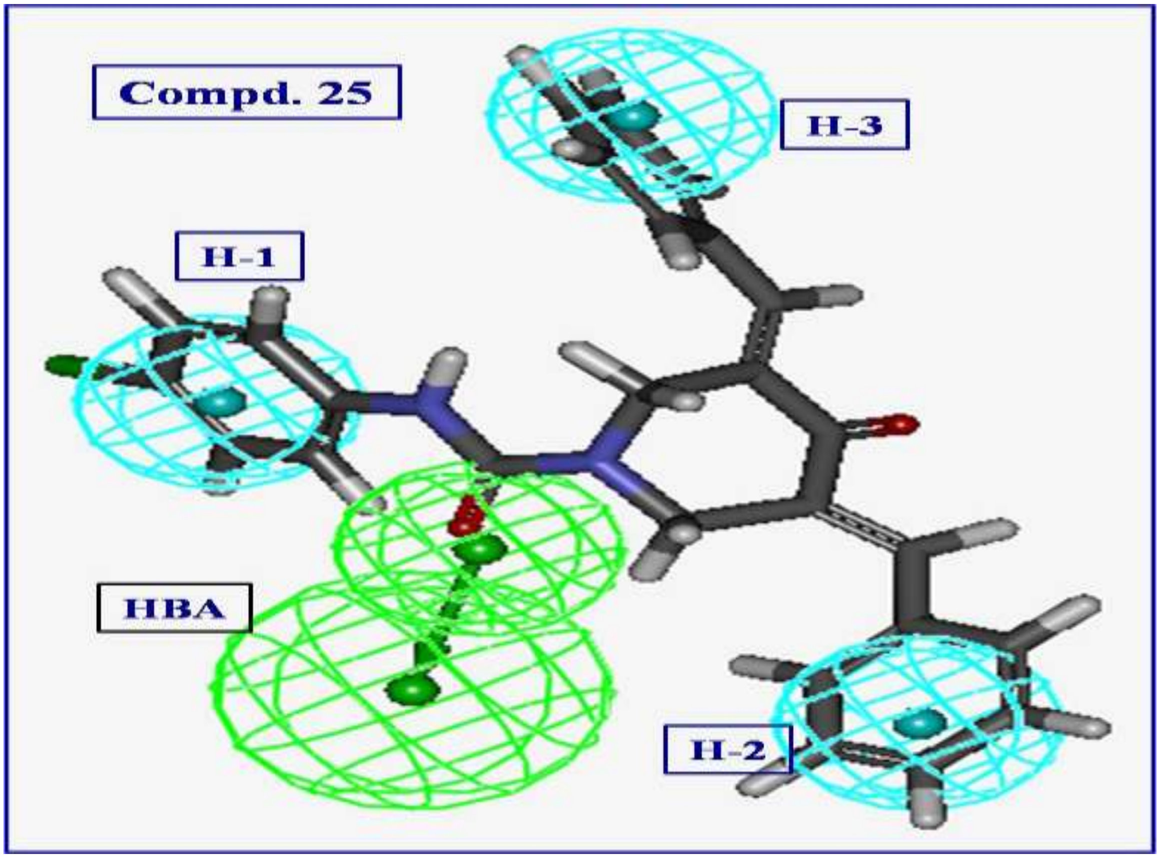
Compd. 25
H-3
H-1
HBA
H-2

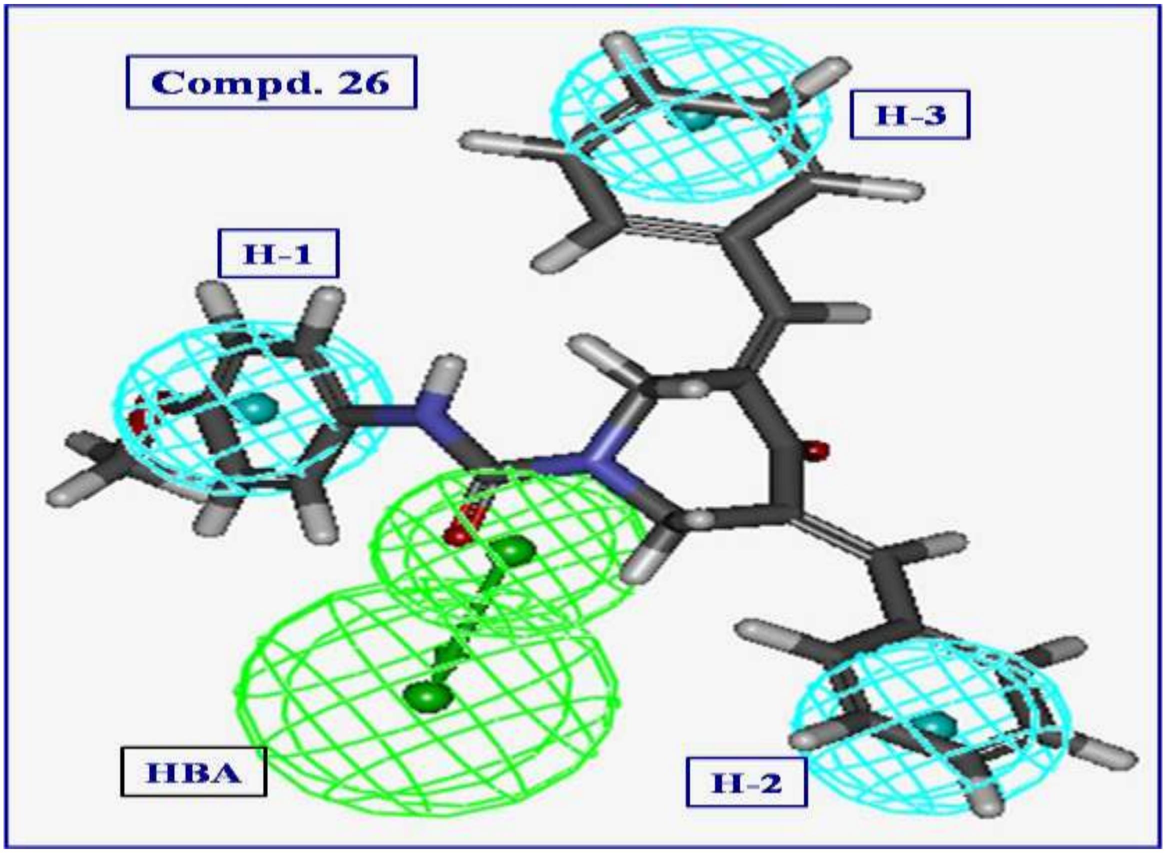

Compd. 26
H-3
H-1
HBA
H-2

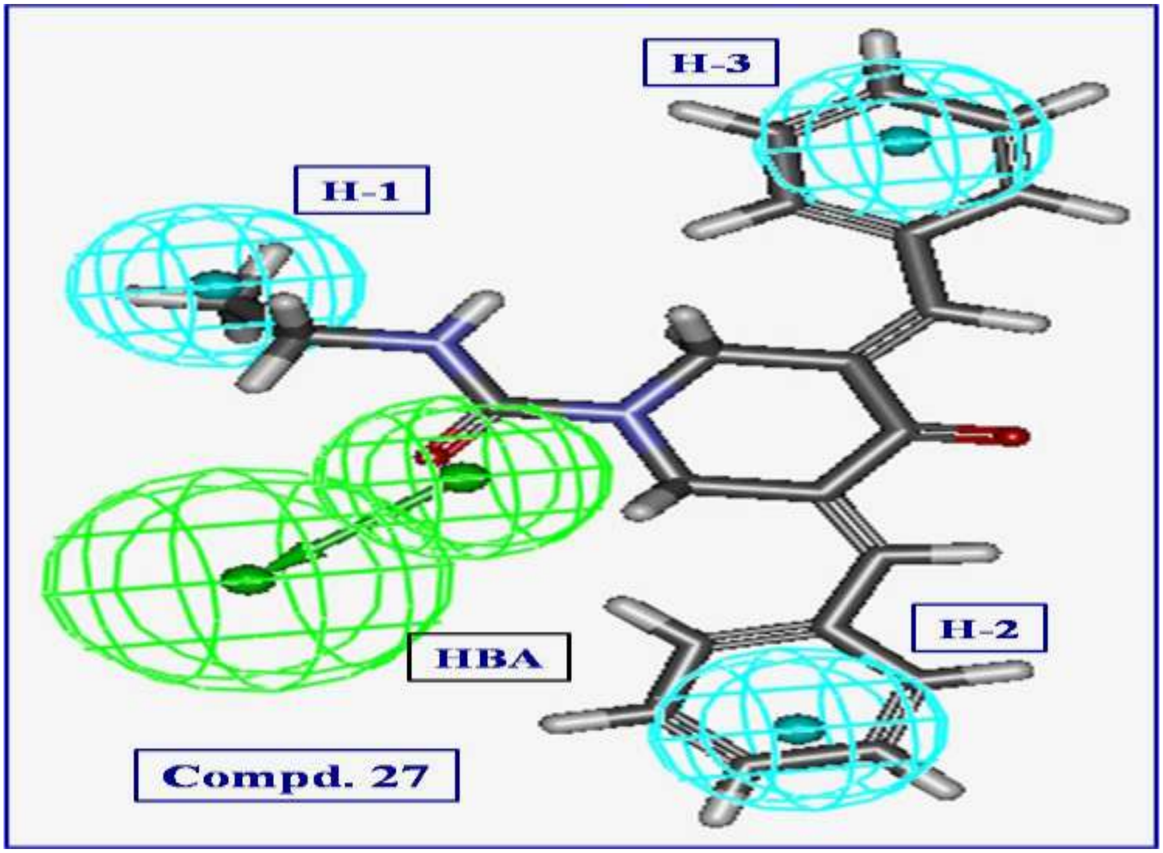

H-3
H-1
H-2
HBA
Compd. 27

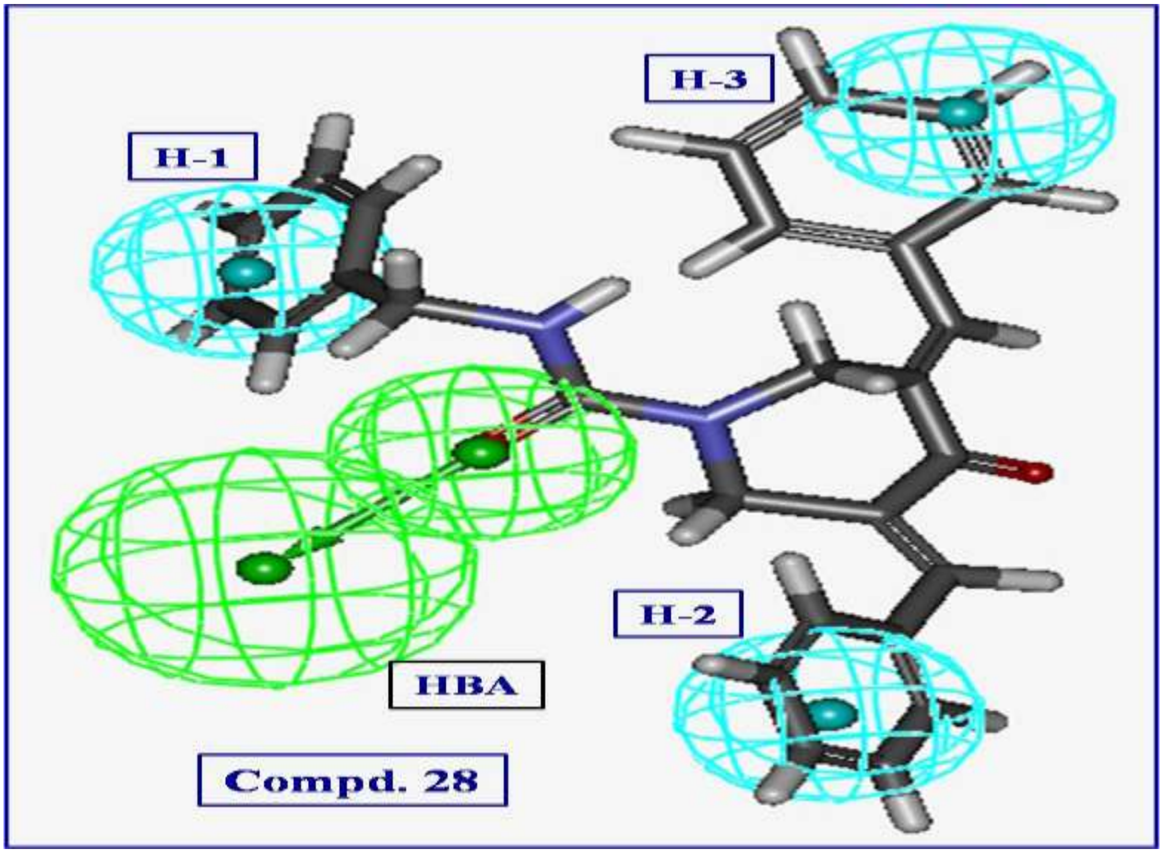
H-3
H-1
H-2
HBA
Compd. 28

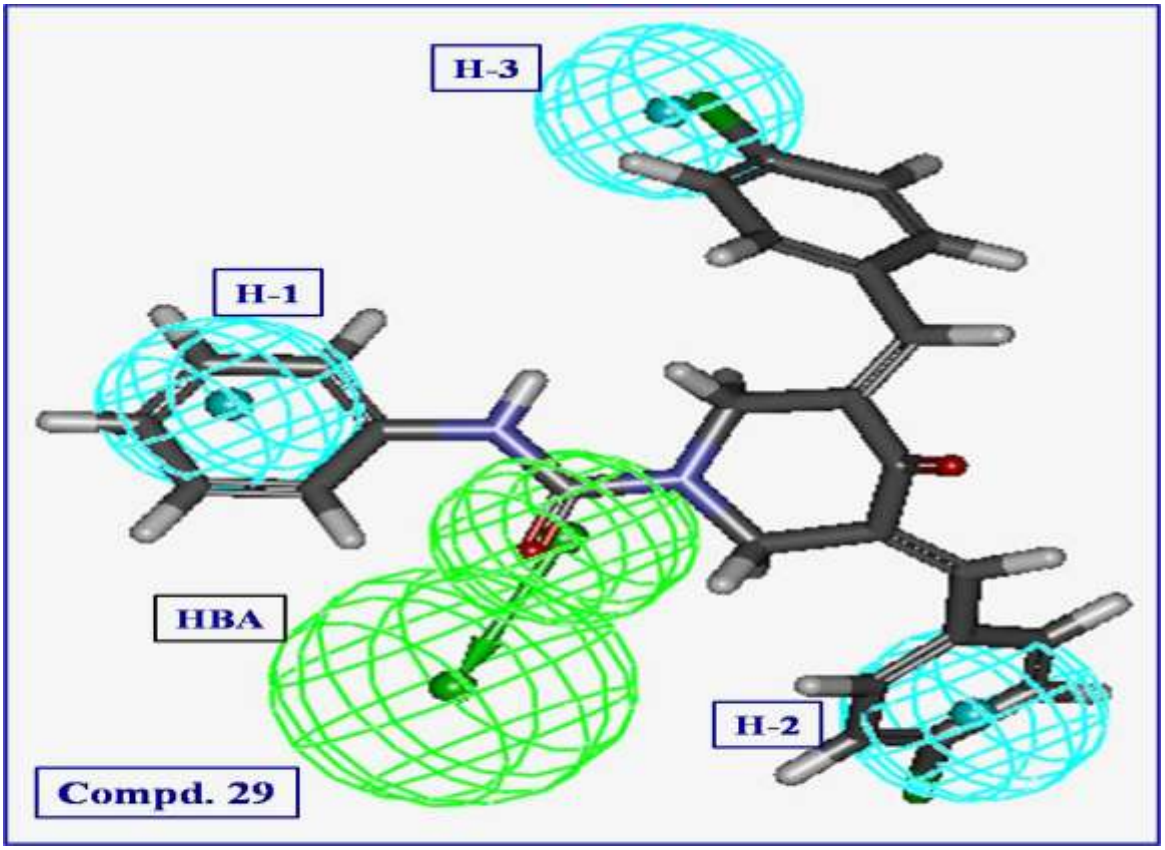
H-3
H-1
HBA
H-2
Compd. 29

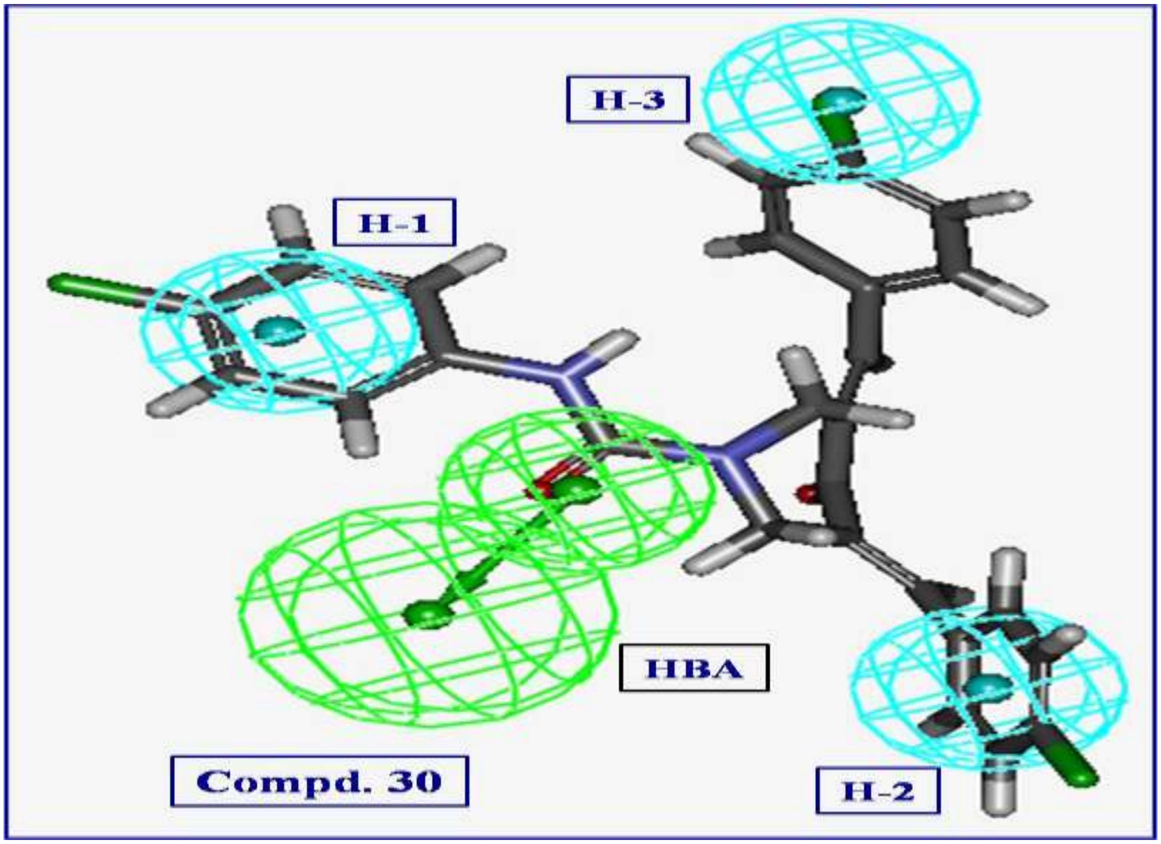
H-3
H-1
HBA
Compd. 30
H-2

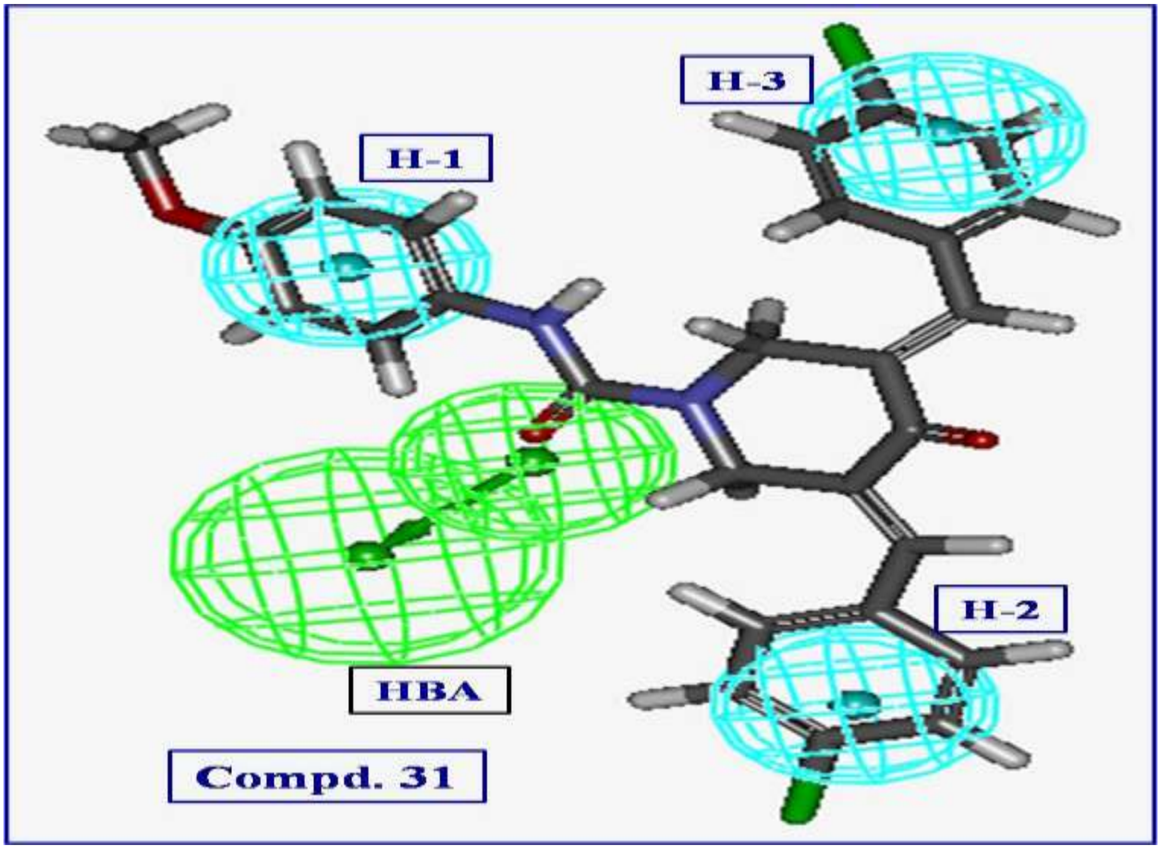
H-3
H-1
H-2
HBA
Compd. 31

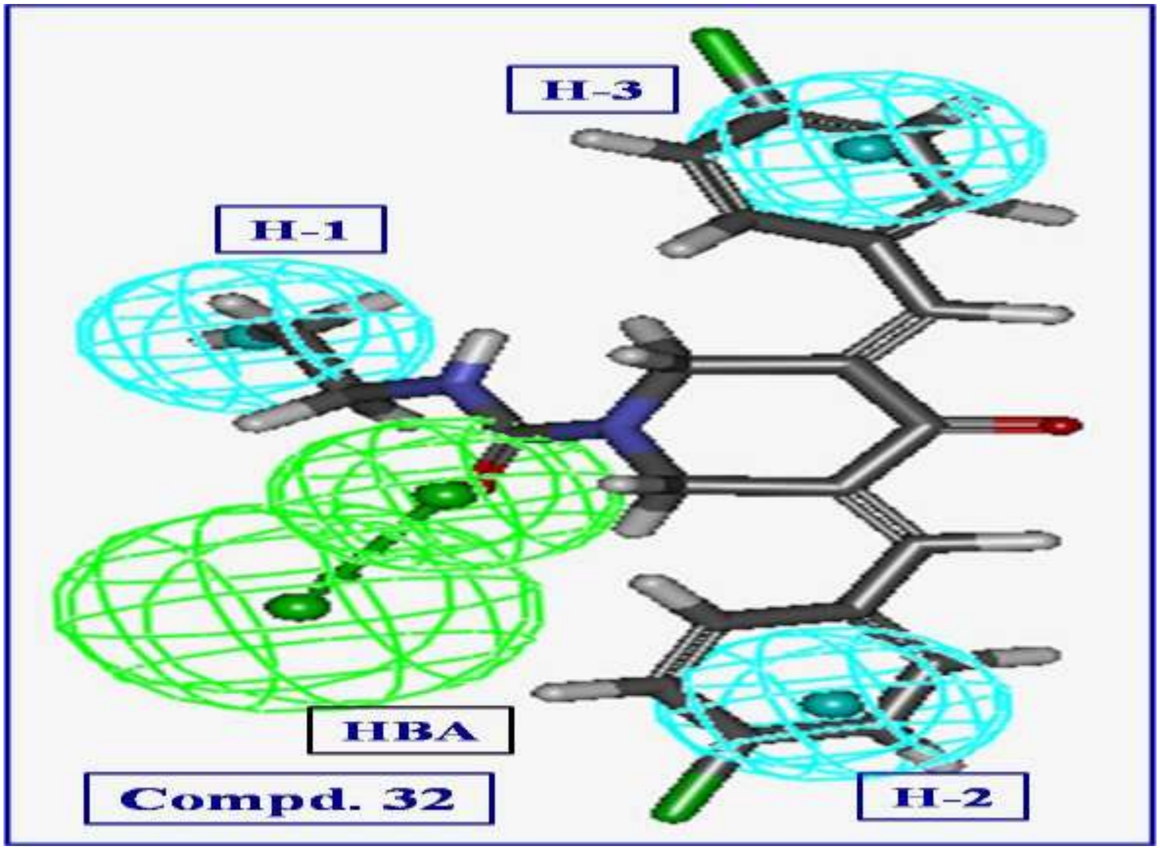
H-3
H-1
HBA
Compd. 32
H-2

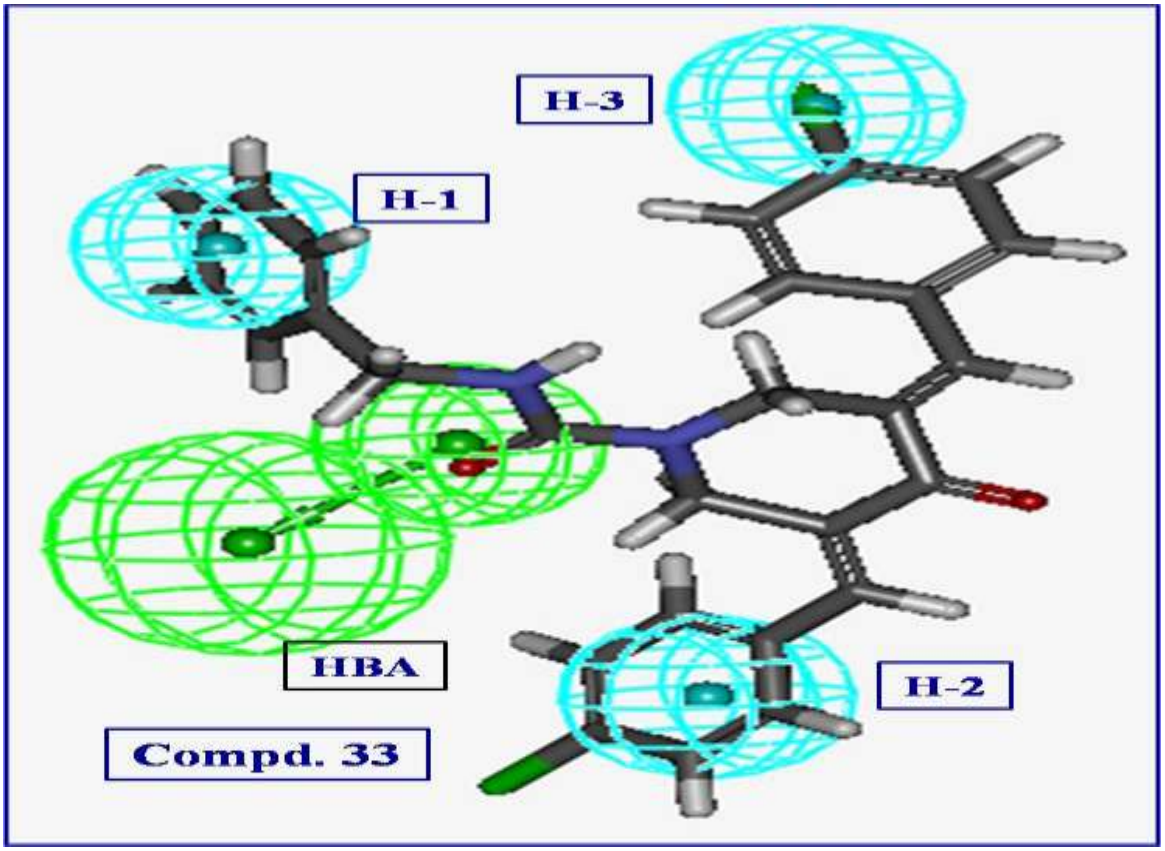
H-3
H-1
HBA
H-2
Compd. 33

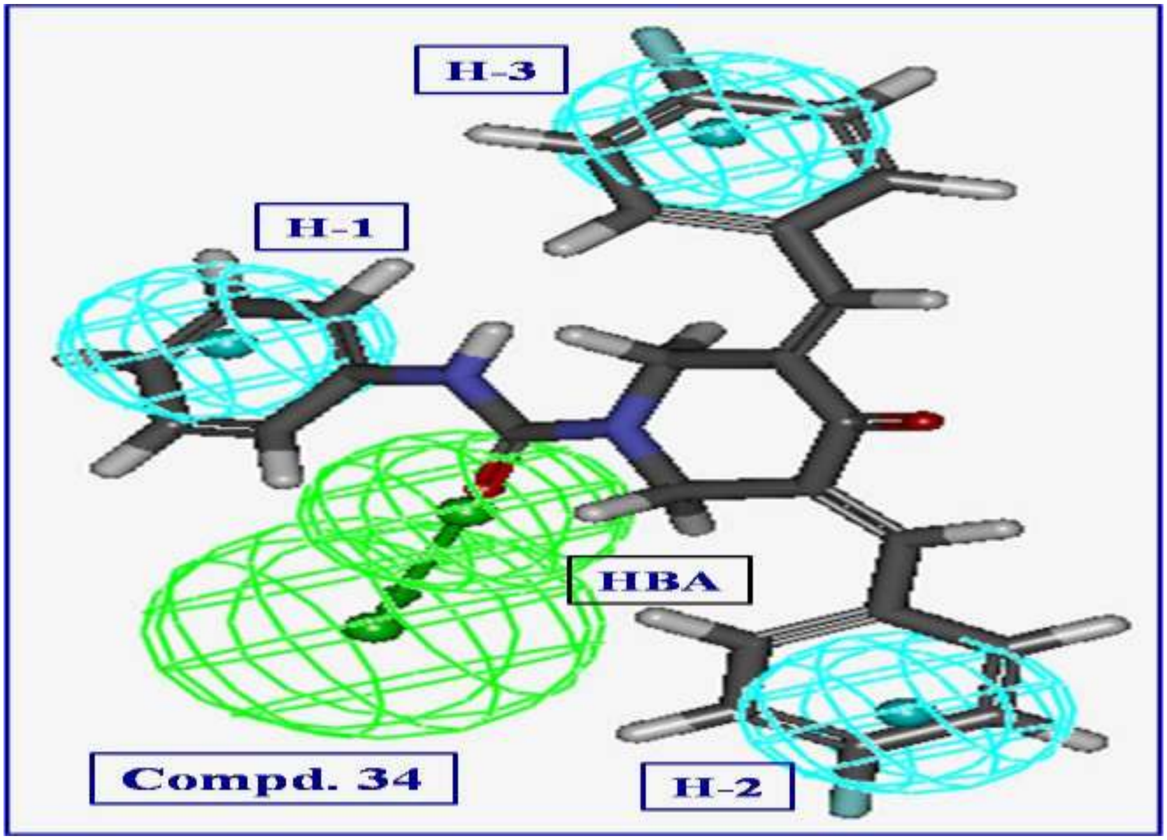
H-3
H-1
HBA
Compd. 34
H-2

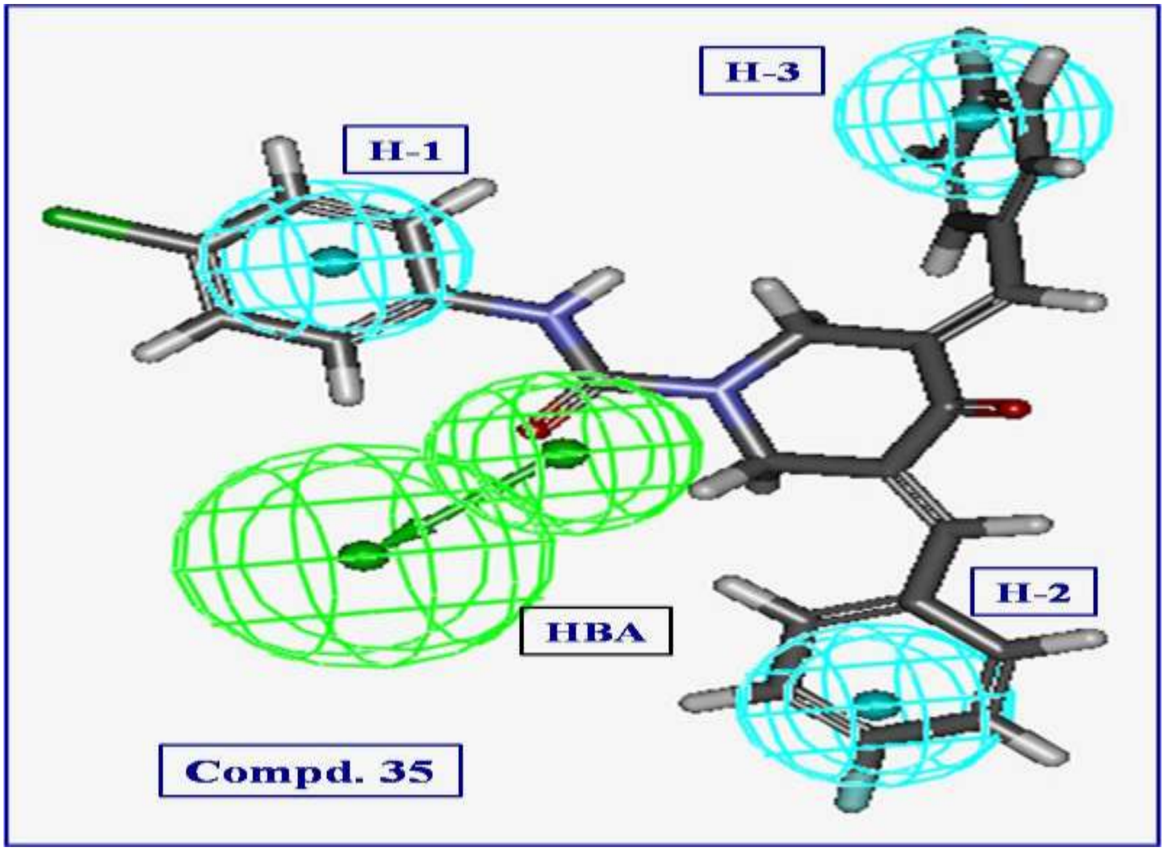
H-3
H-1
H-2
HBA
Compd. 35

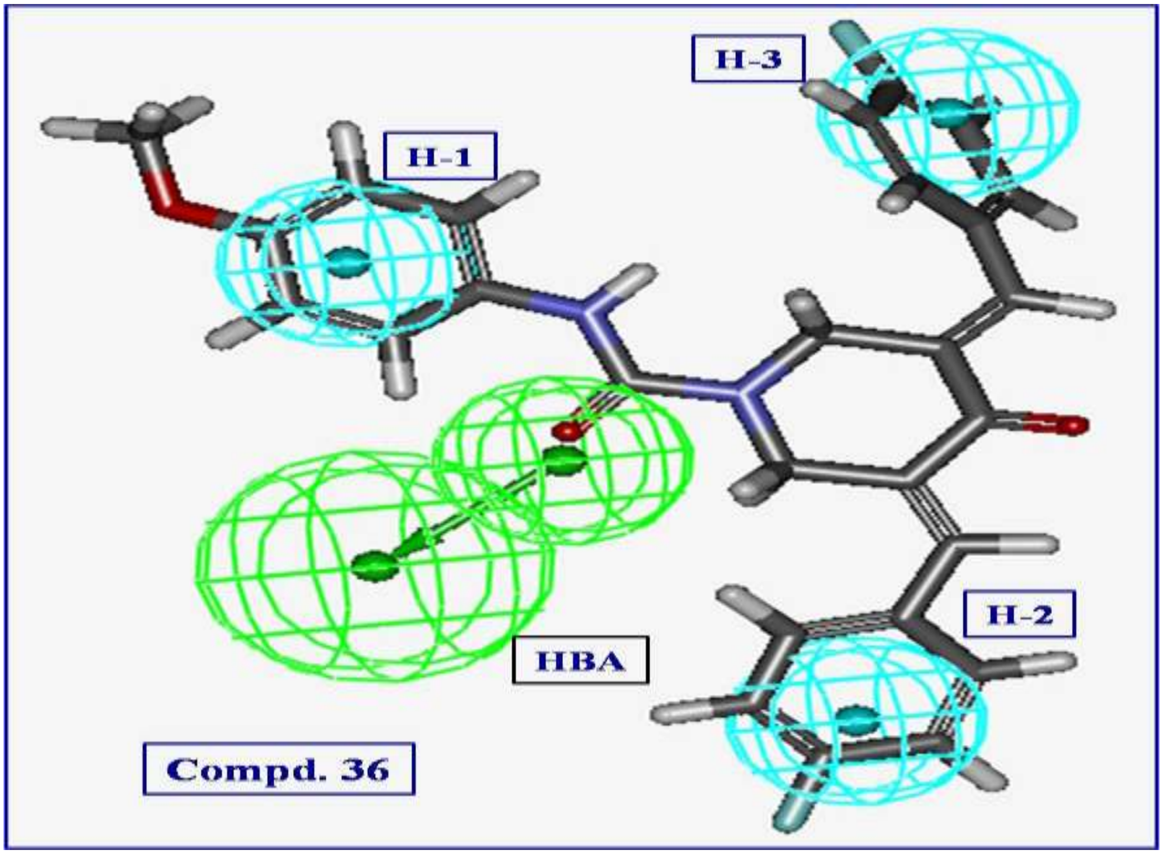
H-3
H-1
HBA
H-2
Compd. 36

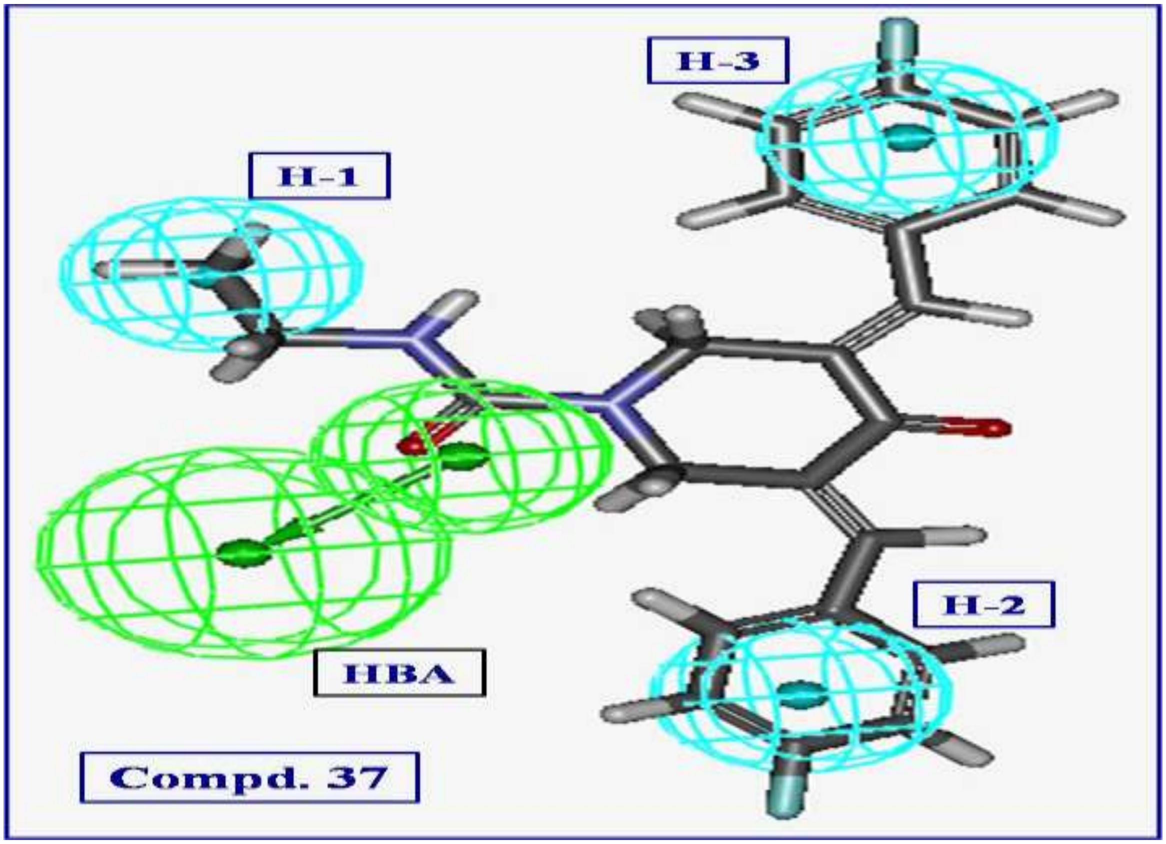
H-3
H-1
HBA
H-2
Compd. 37

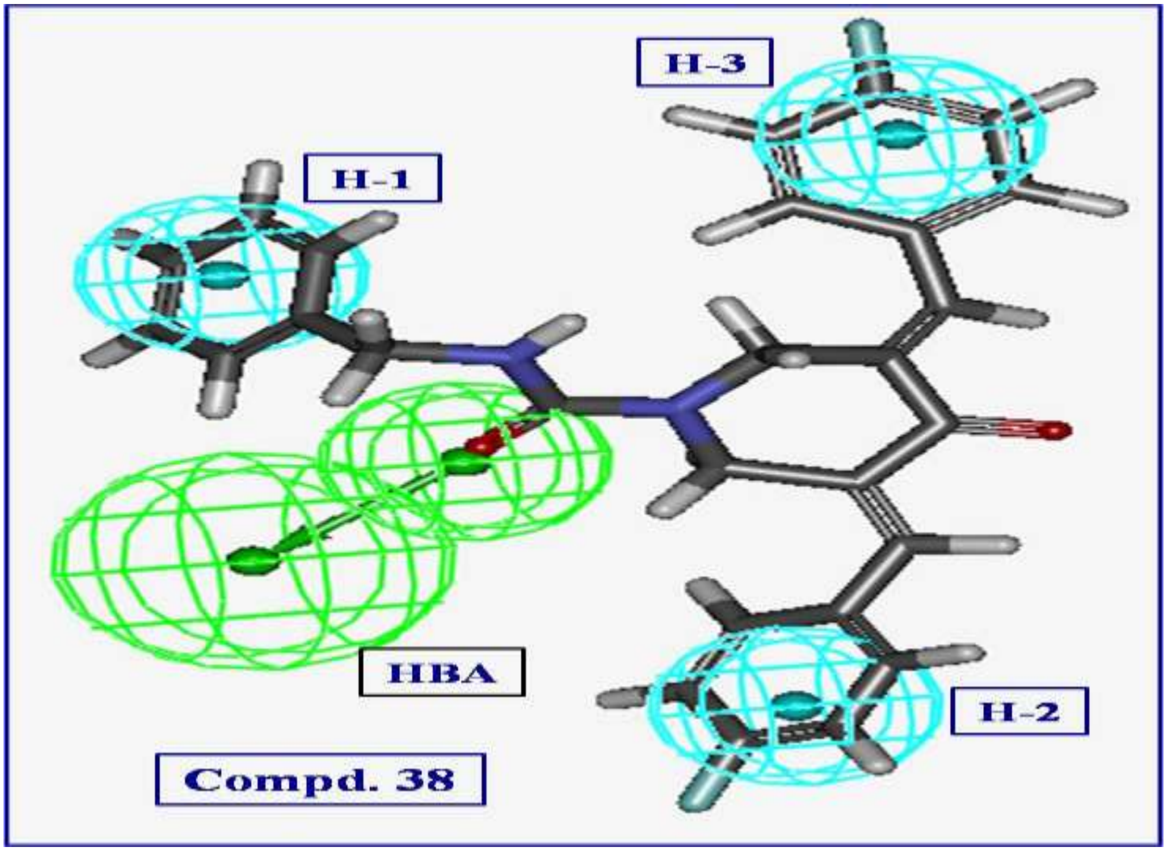
H-3
H-1
HBA
H-2
Compd. 38

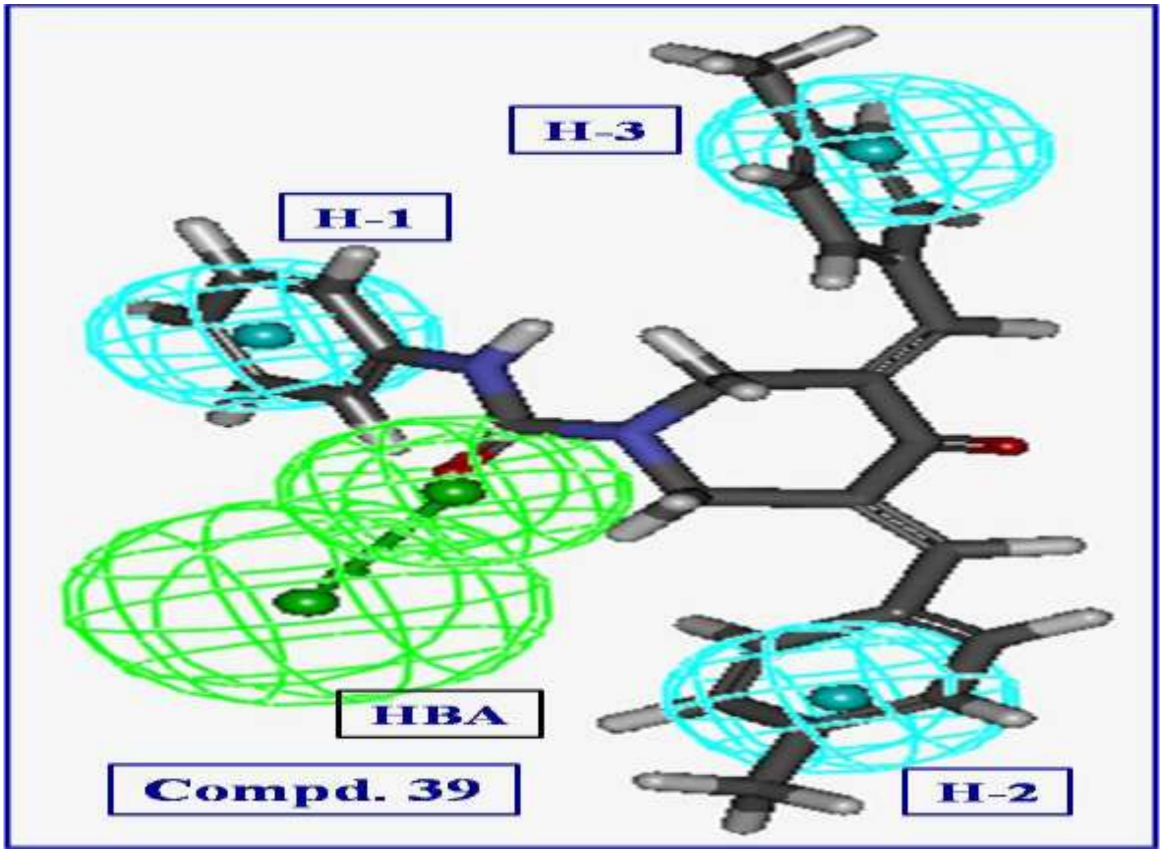
H-3
H-1
HBA
Compd. 39
H-2

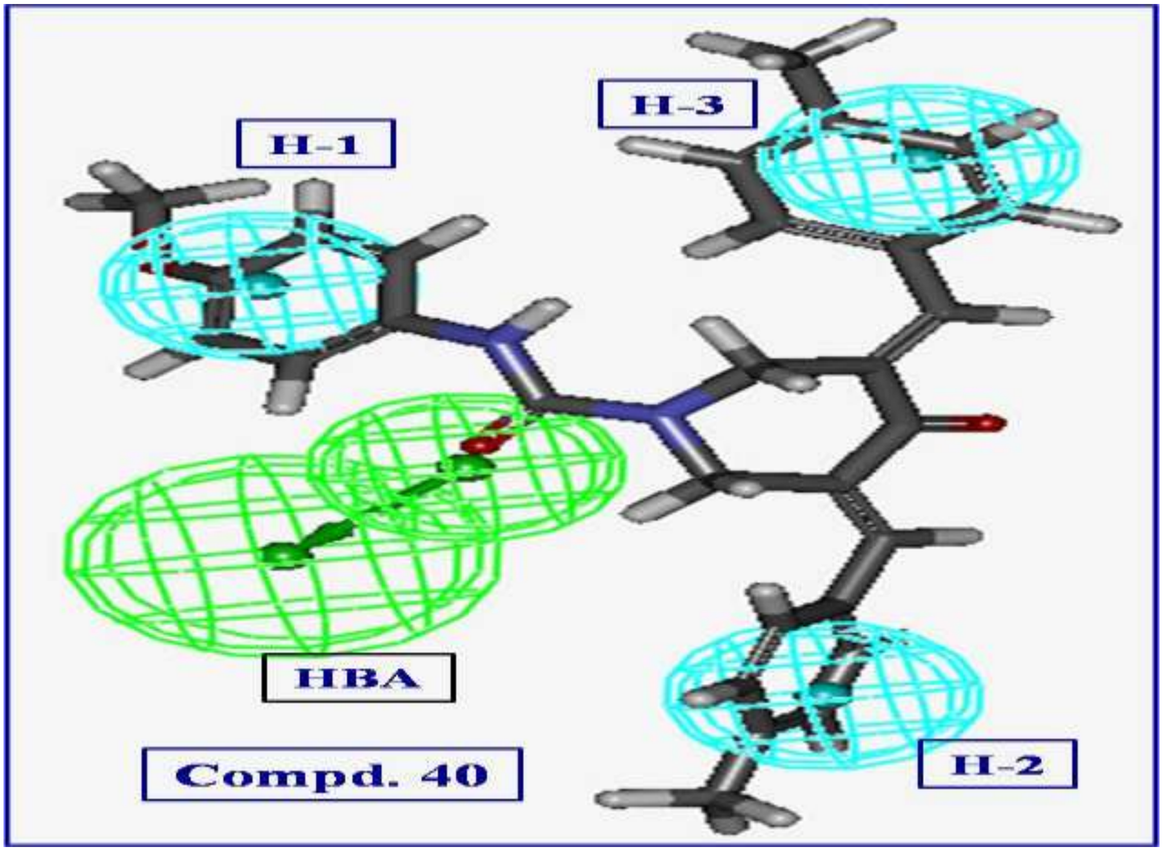
H-1
H-3
HBA
Compd. 40
H-2

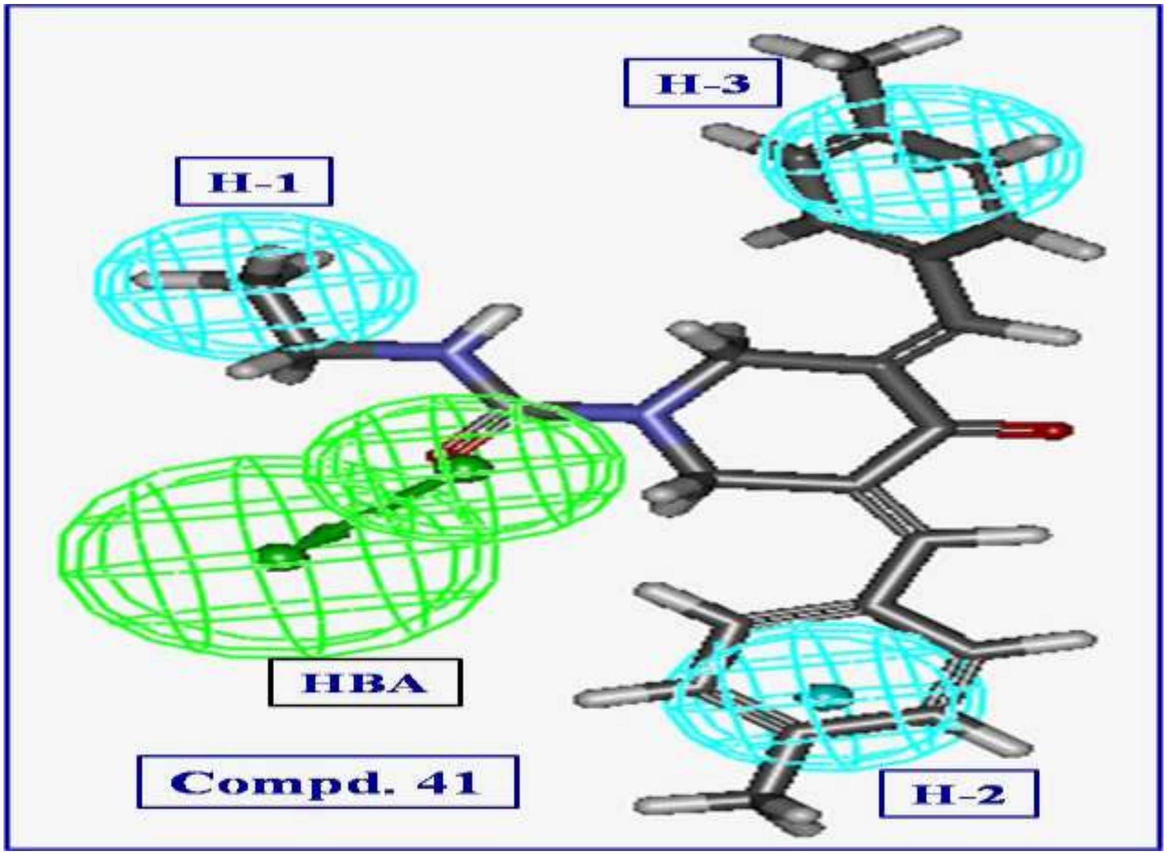
H-3
H-1
HBA
Compd. 41
H-2

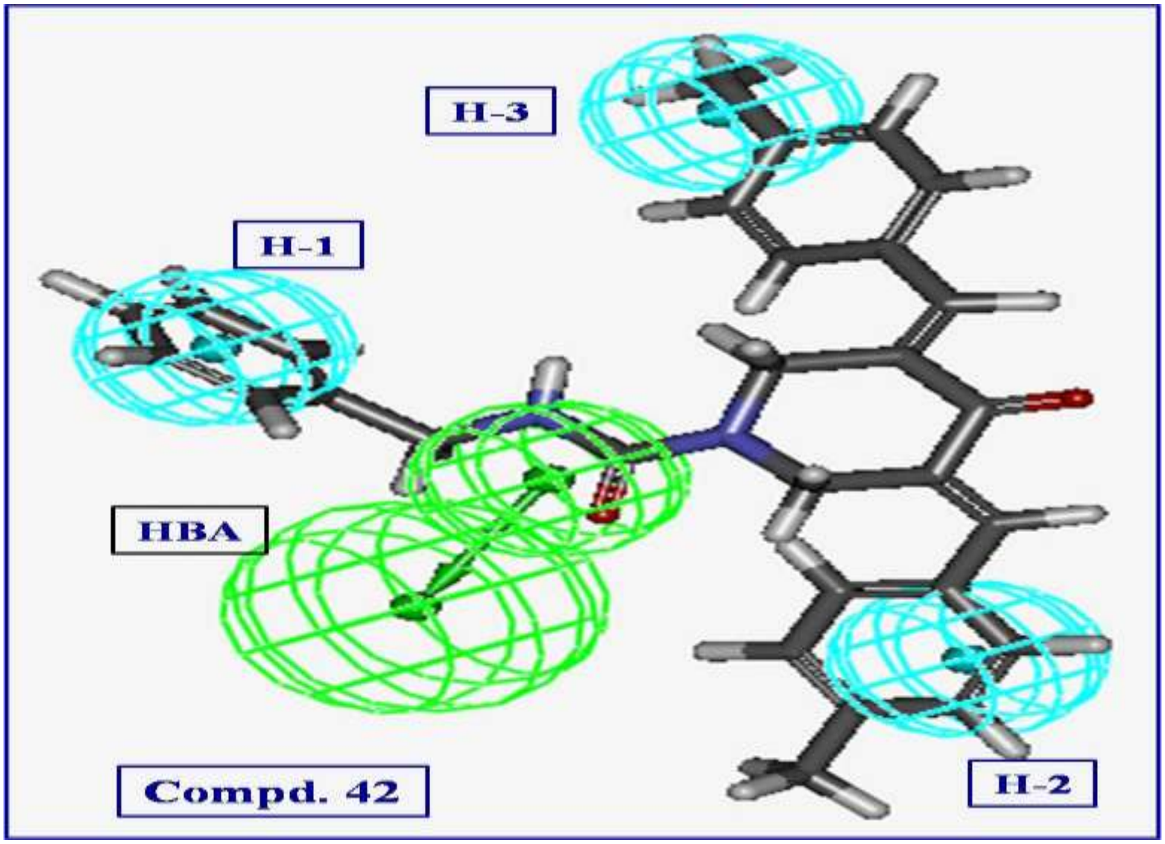
H-3
H-1
HBA
Compd. 42
H-2

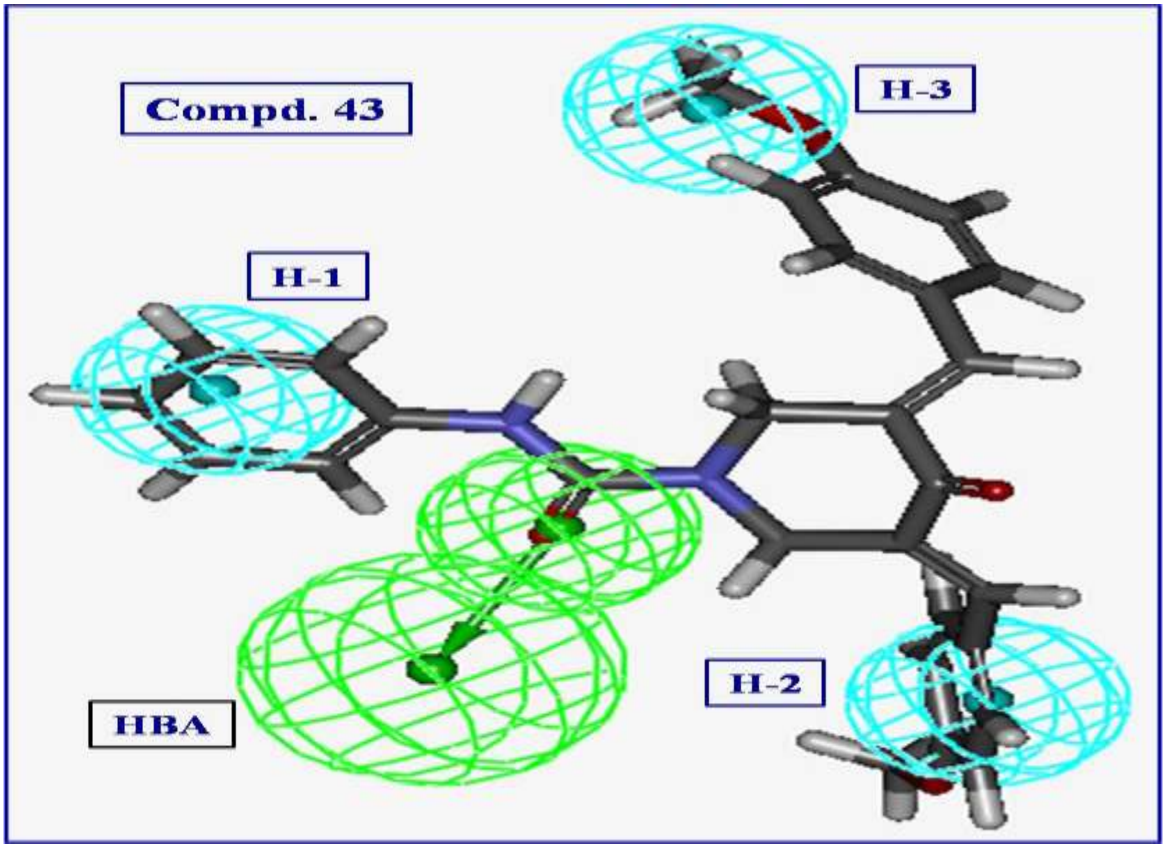
Compd. 43
H-3
H-1
H-2
HBA

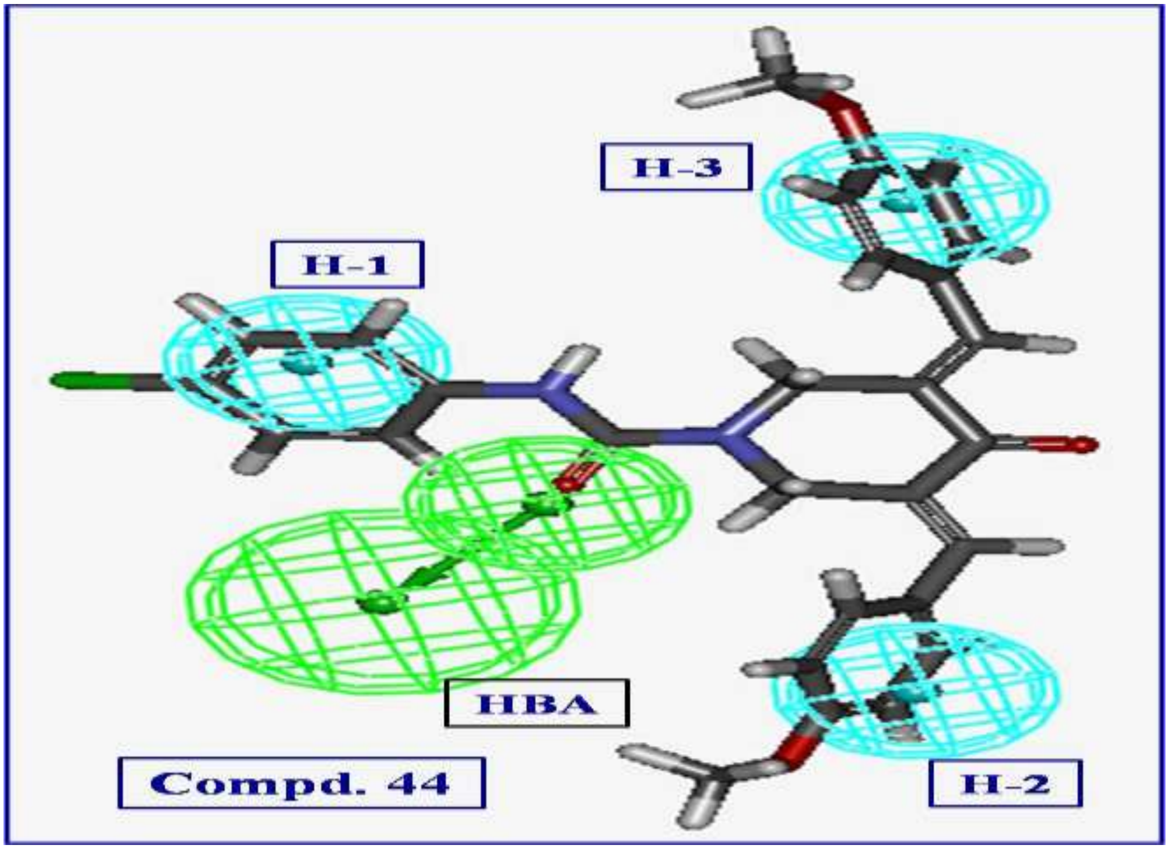
H-3
H-1
HBA
Compd. 44
H-2

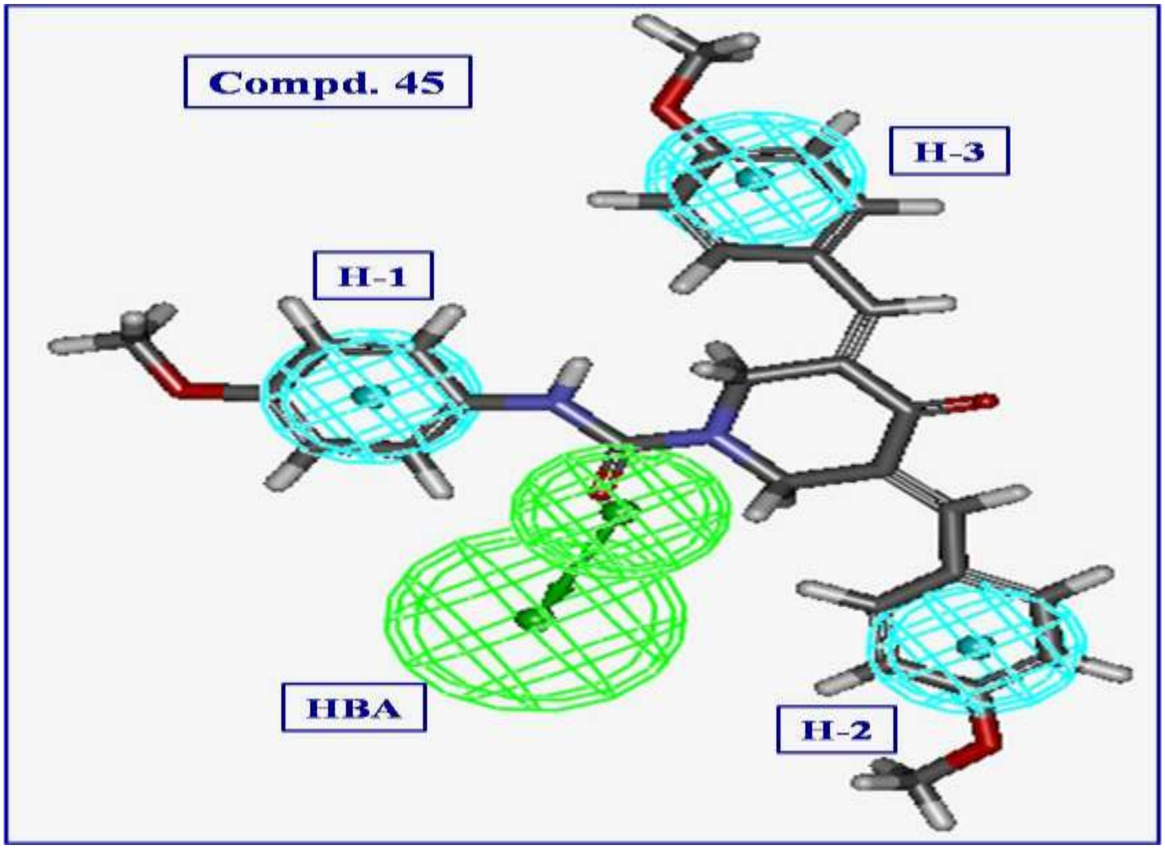
Compd. 45
H-3
H-1
HBA
H-2

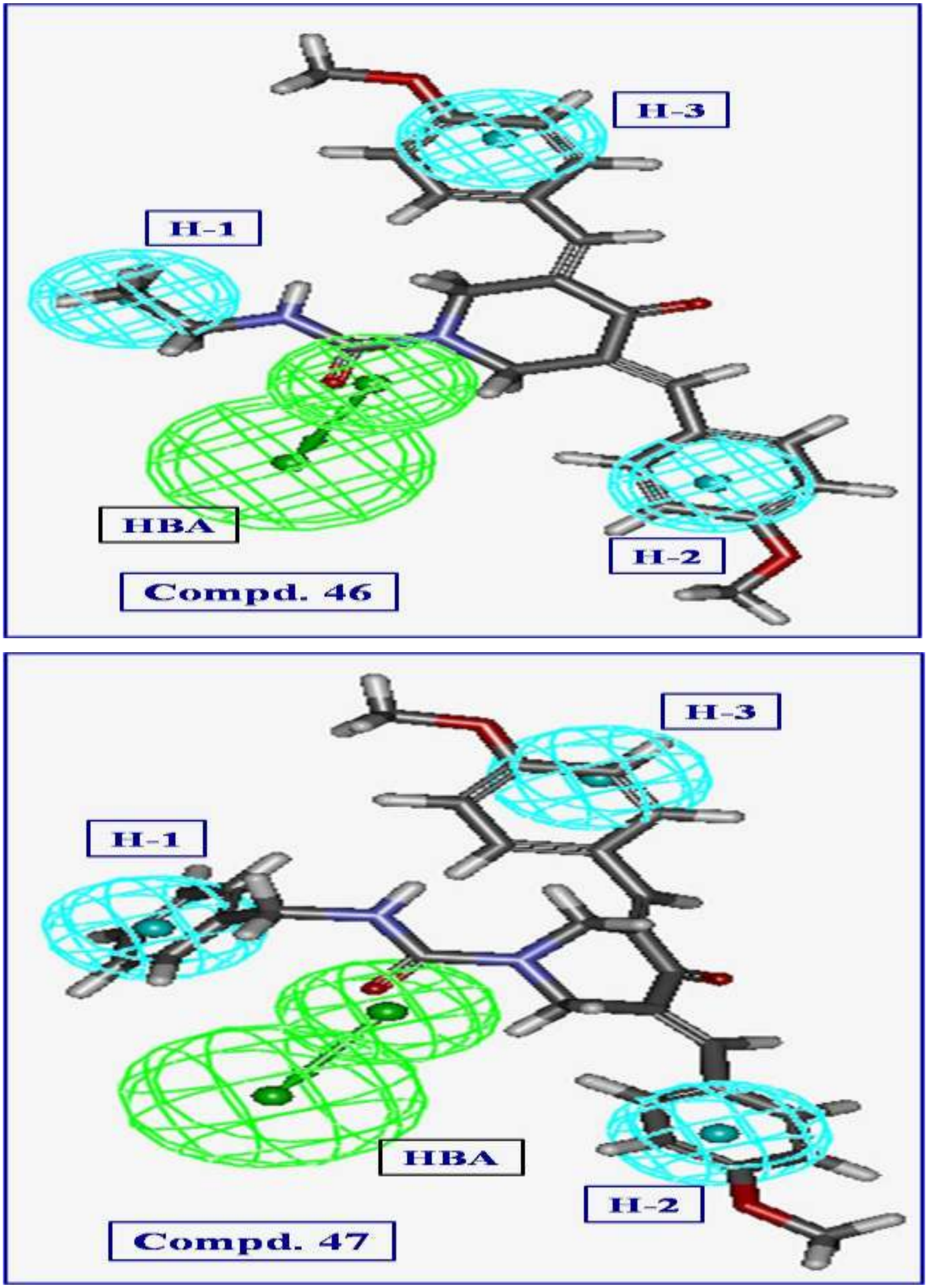

Fig. S102. 3D-pharmacophore model mapped on the tested piperidinecarboxamides **24–47** against HCT116 (colon) carcinoma cell line.

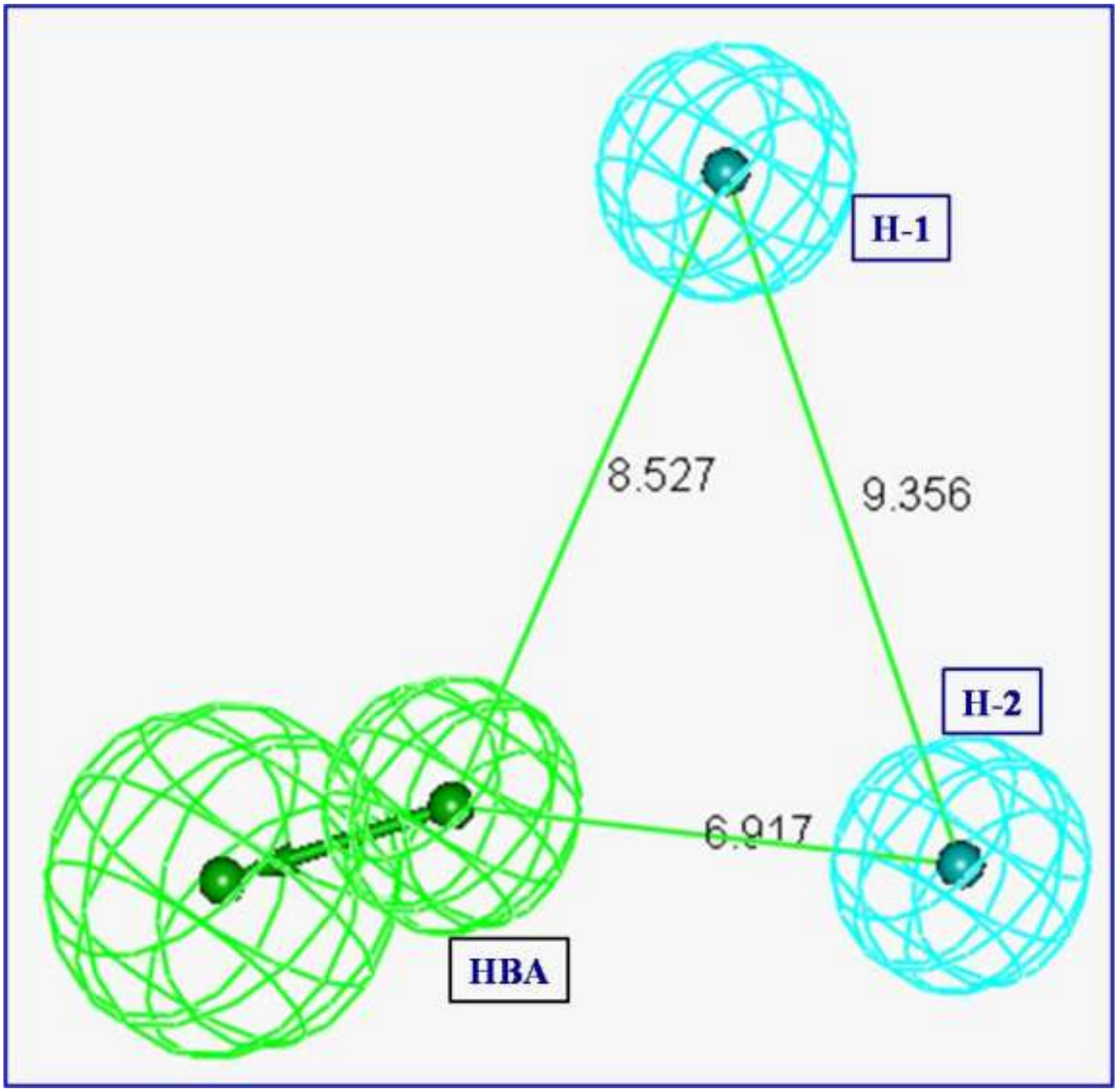

Fig. S103. Constraint distances "H-1 – H-2 = 9.356, H-1 – HBA = 8.527, H-2 – HBA = 6.917 Å" of the generated 3D-pharmacophore for the tested piperidinecarboxamides **24–47** against MCF7 (breast) carcinoma cell line which contains two hydrophobics (H-1, H-2; light blue) and one hydrogen bonding acceptor (HBA; green).

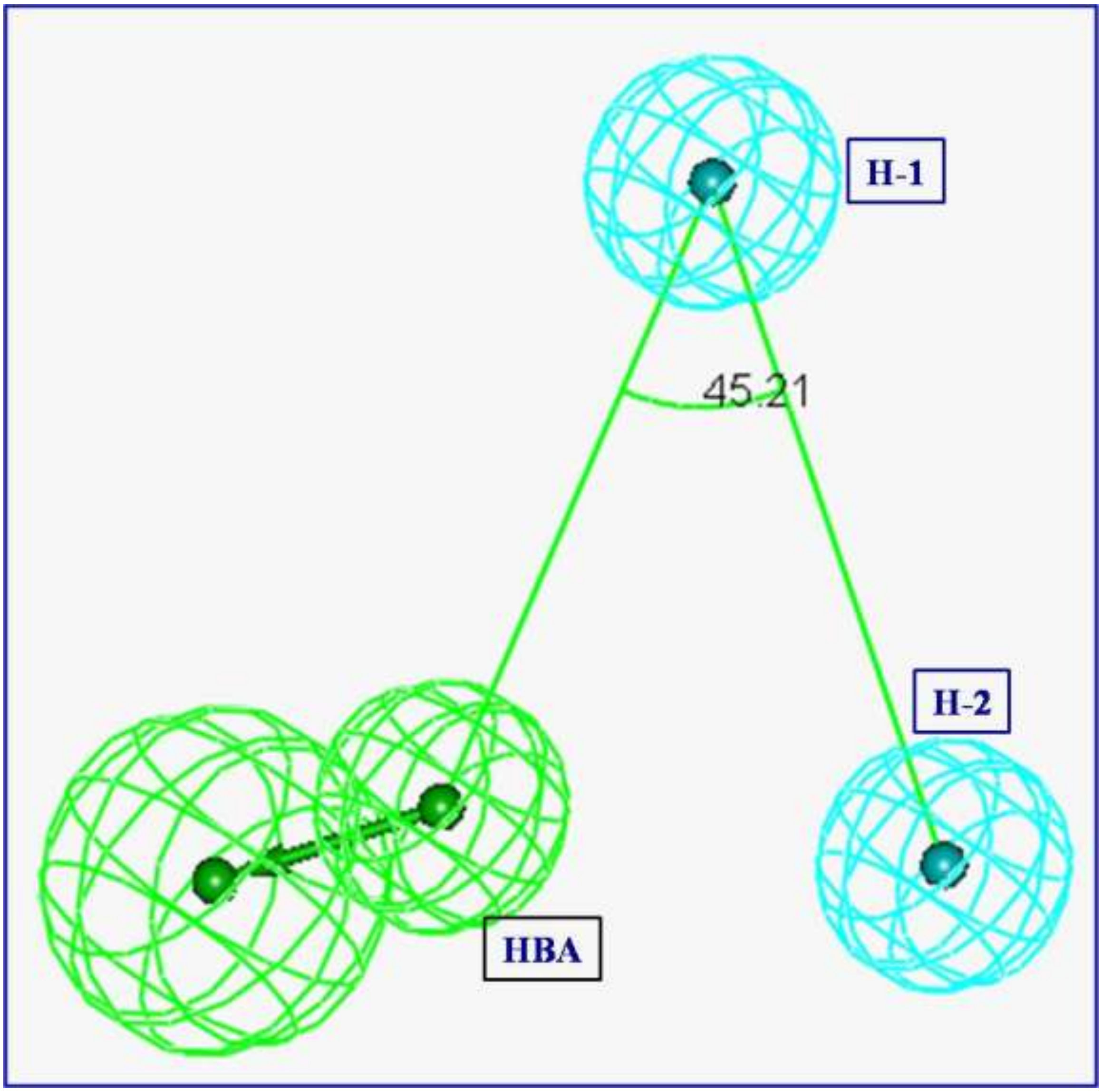

Fig. S104. Constraint angle “H-2 – H-1 – HBA = 45.21 °” of the generated 3D-pharmacophore for the tested piperidinecarboxamides **24–47** against MCF7 (breast) carcinoma cell line which contains two hydrophobics (H-1, H-2; light blue) and one hydrogen bonding acceptor (HBA; green).

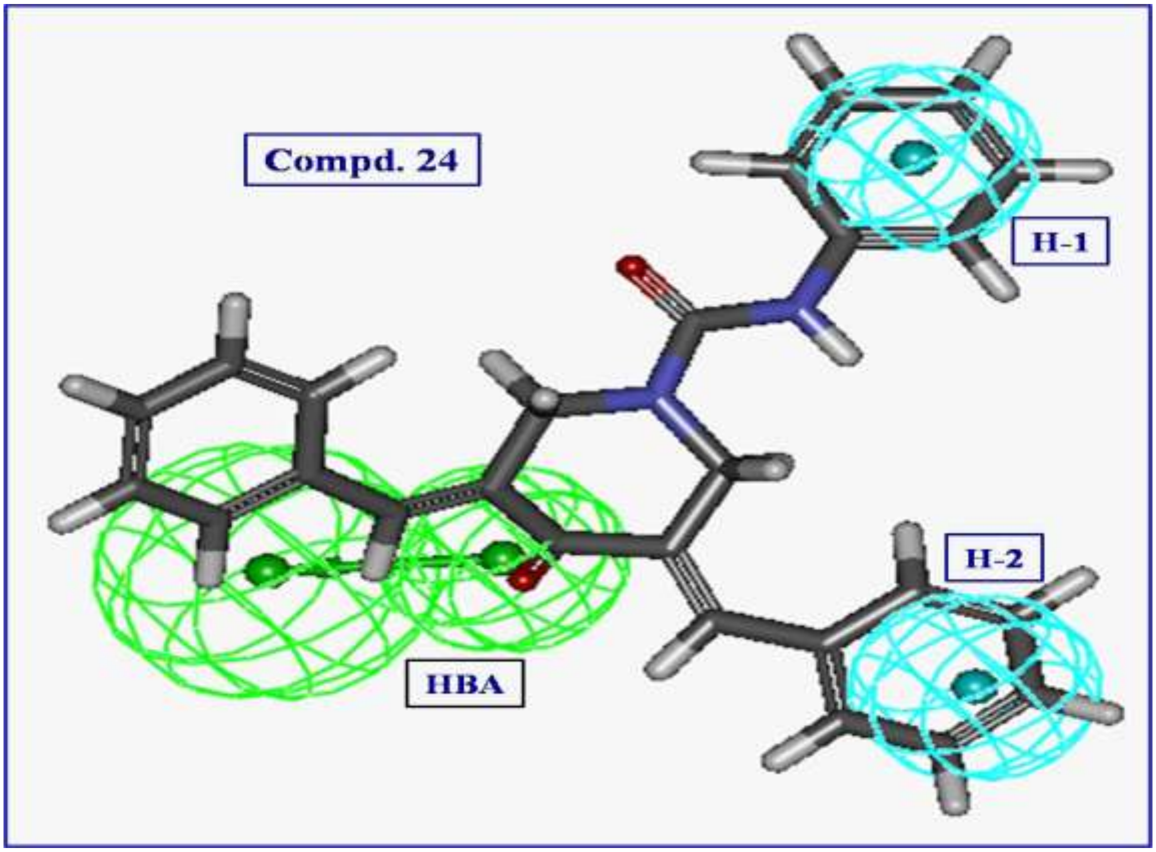
Compd. 24
H-1
H-2
HBA

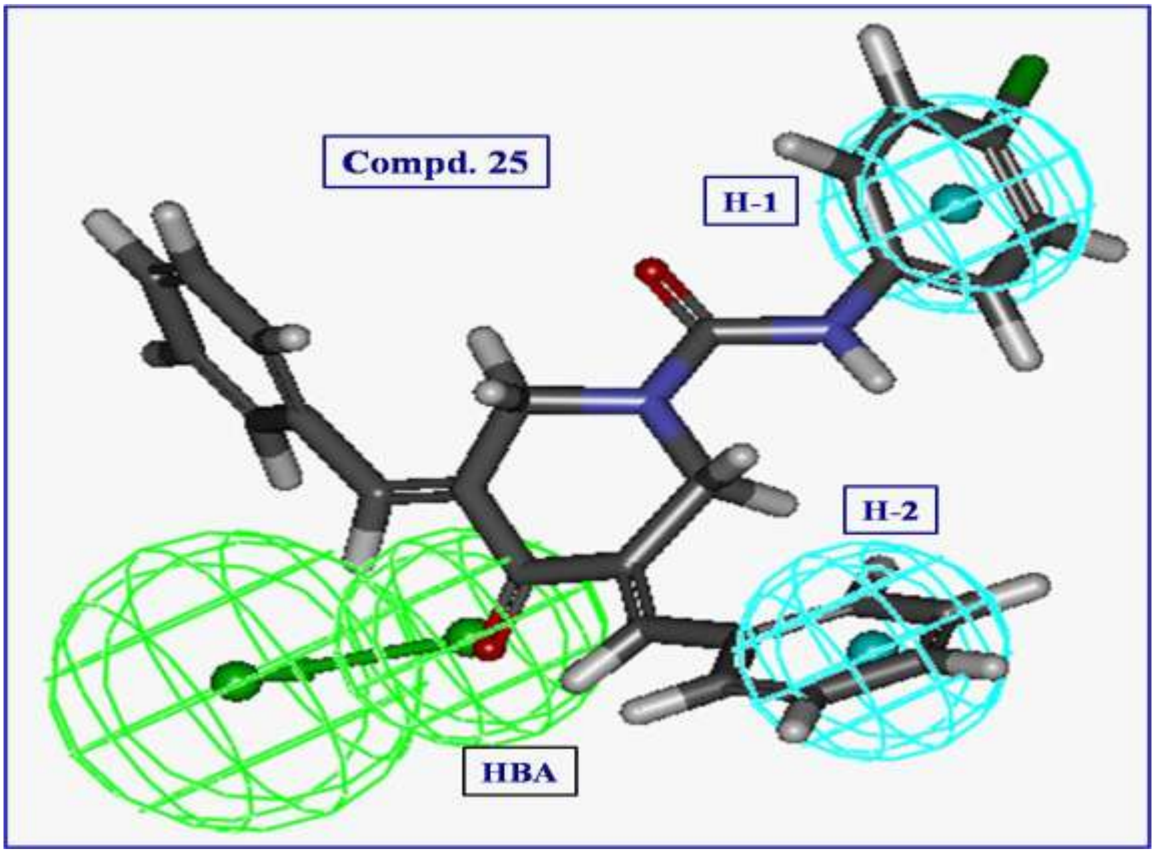
Compd. 25
H-1
H-2
HBA

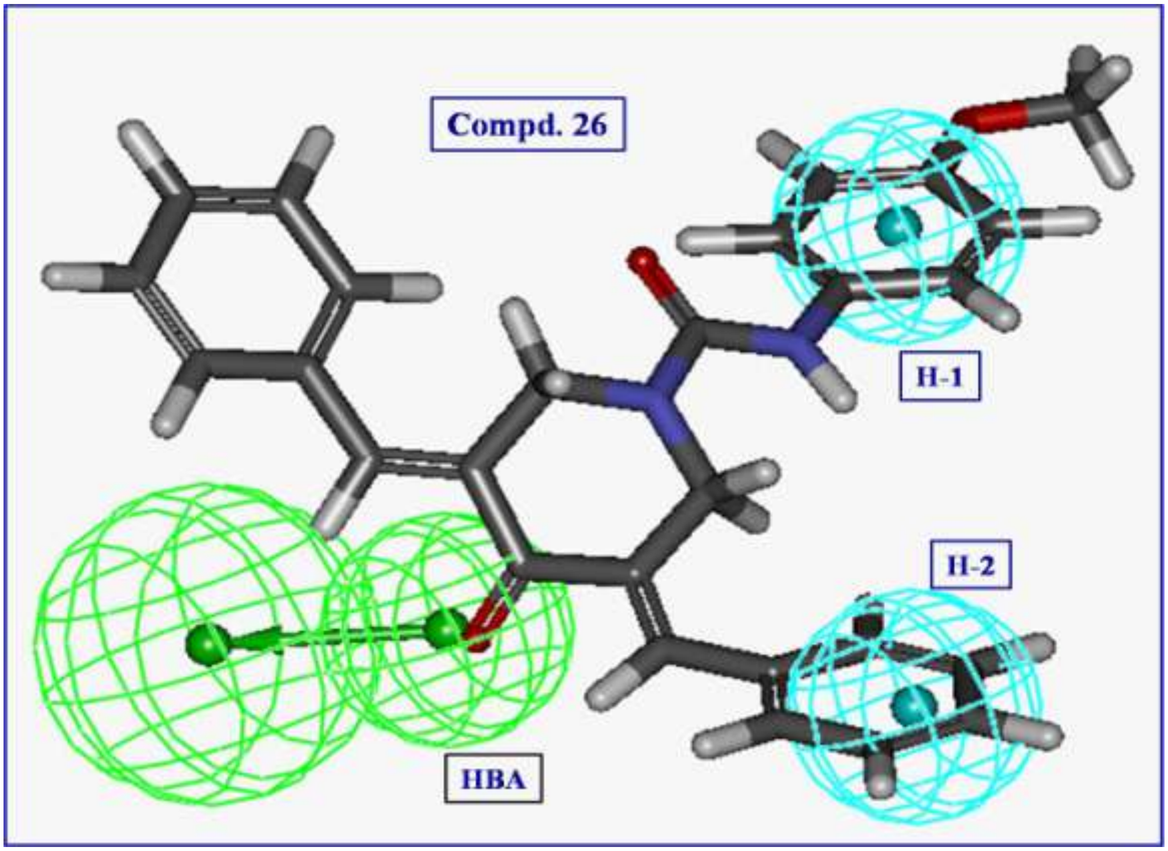

Compd. 26
H-1
H-2
HBA

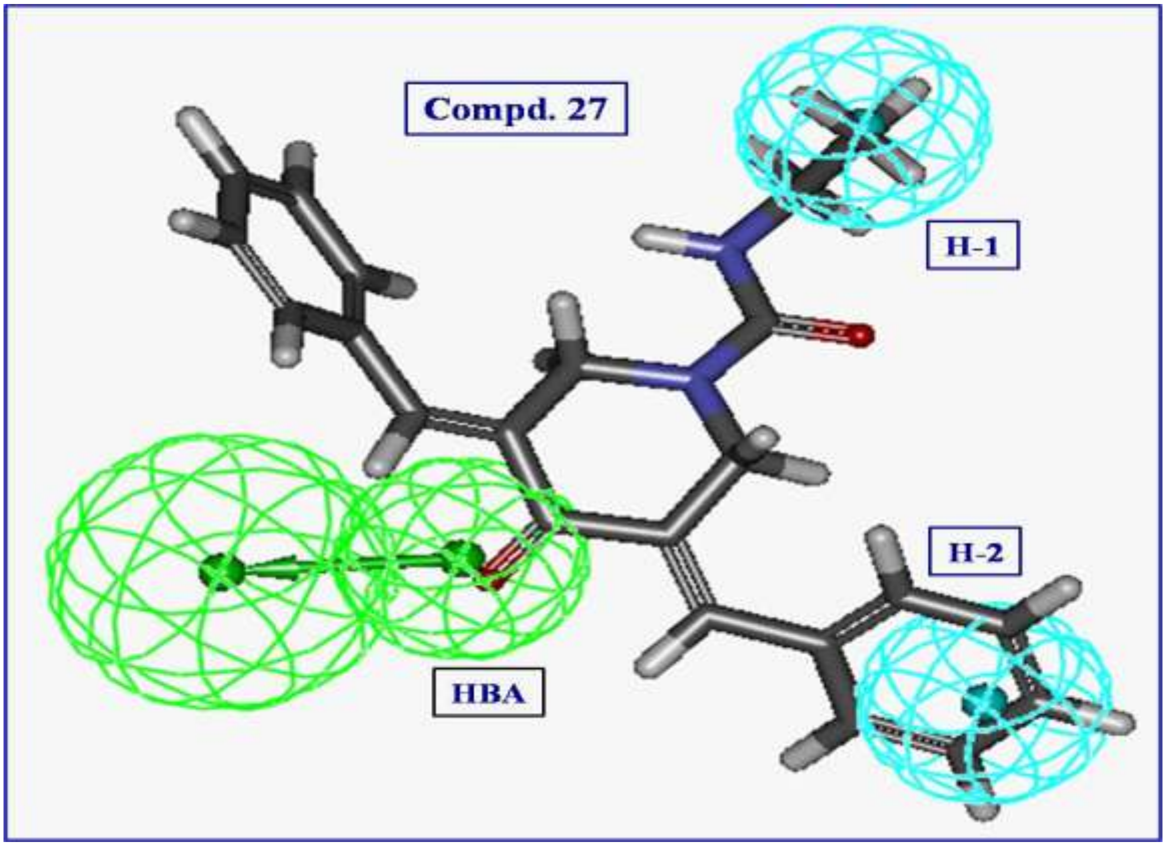

Compd. 27
H-1
H-2
HBA

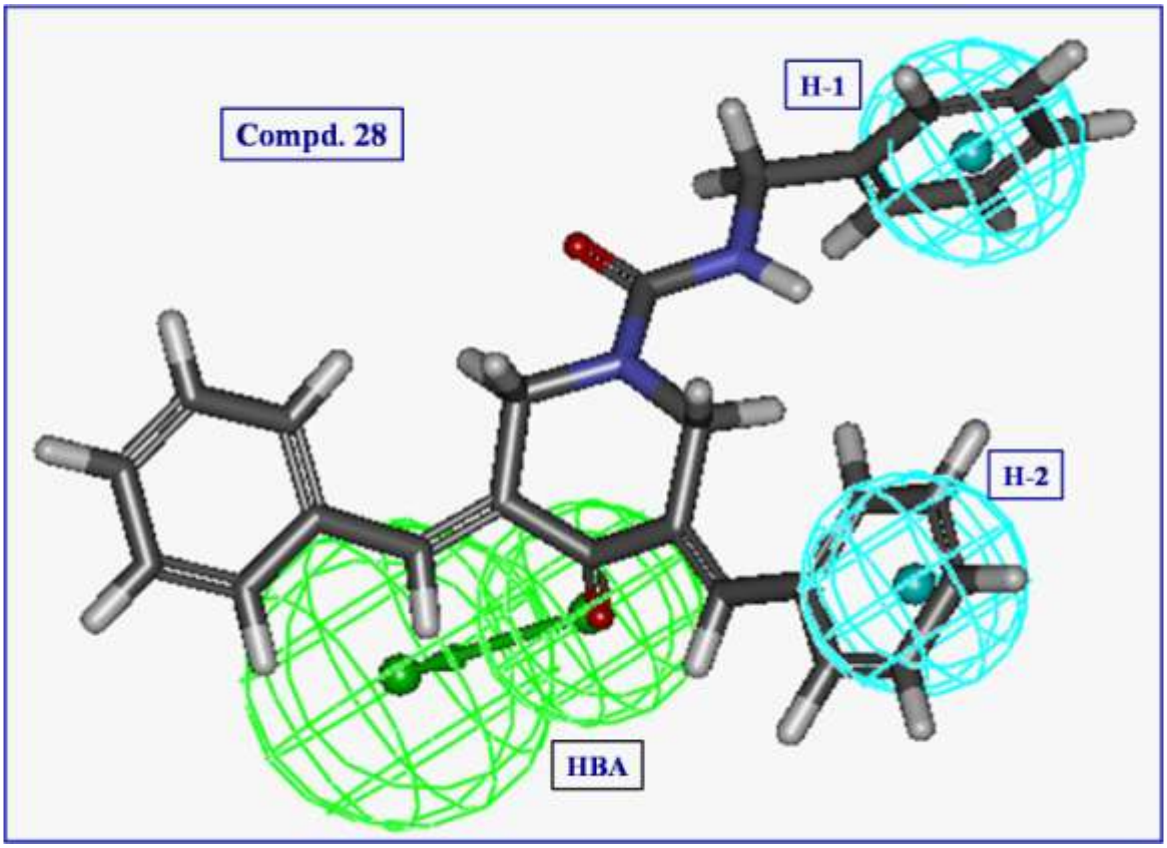
Compd. 28
H-1
H-2
HBA

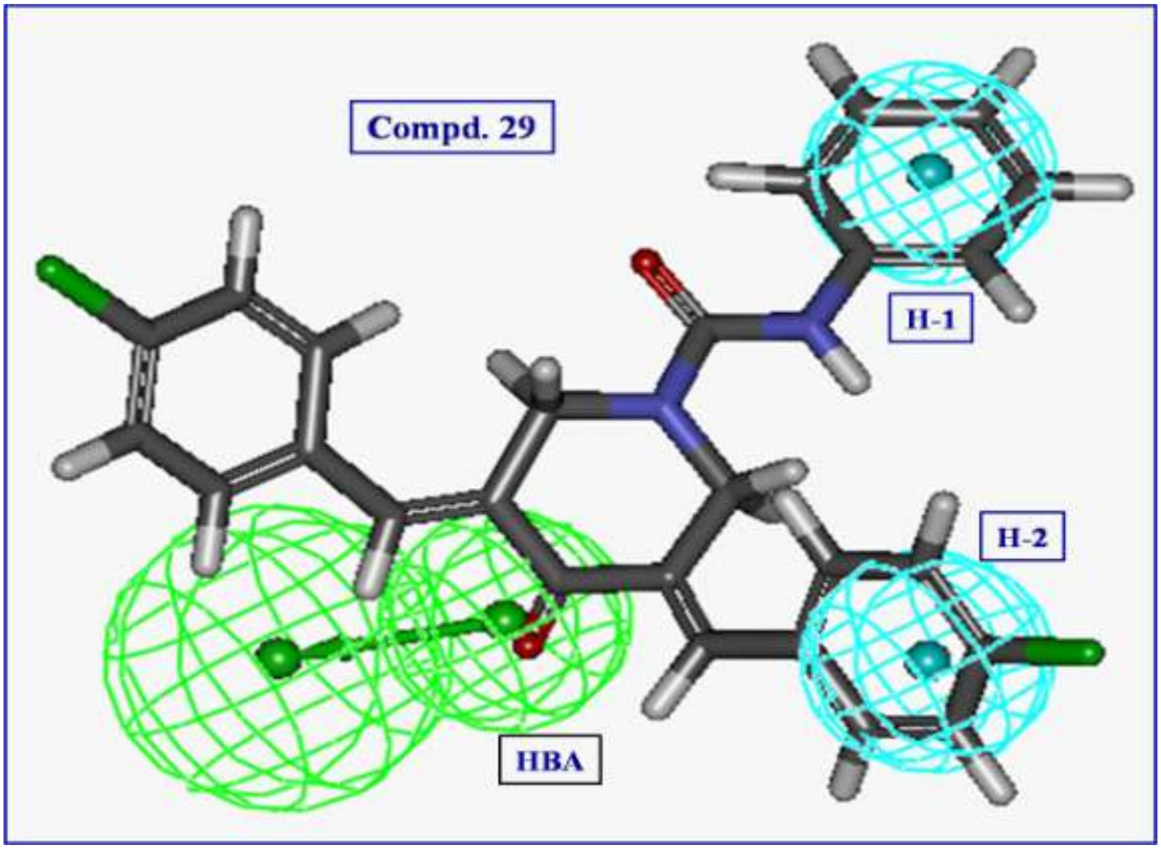
Compd. 29
H-1
H-2
HBA

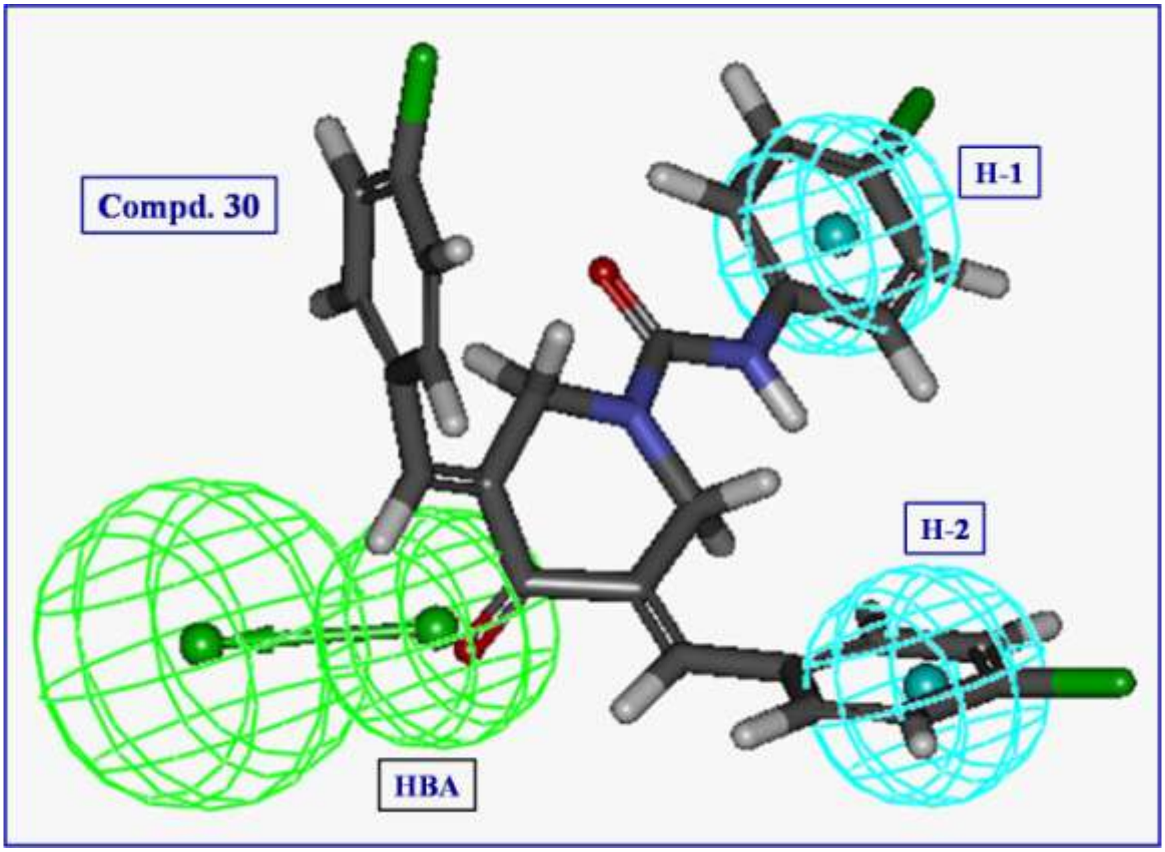
Compd. 30
H-1
H-2
HBA

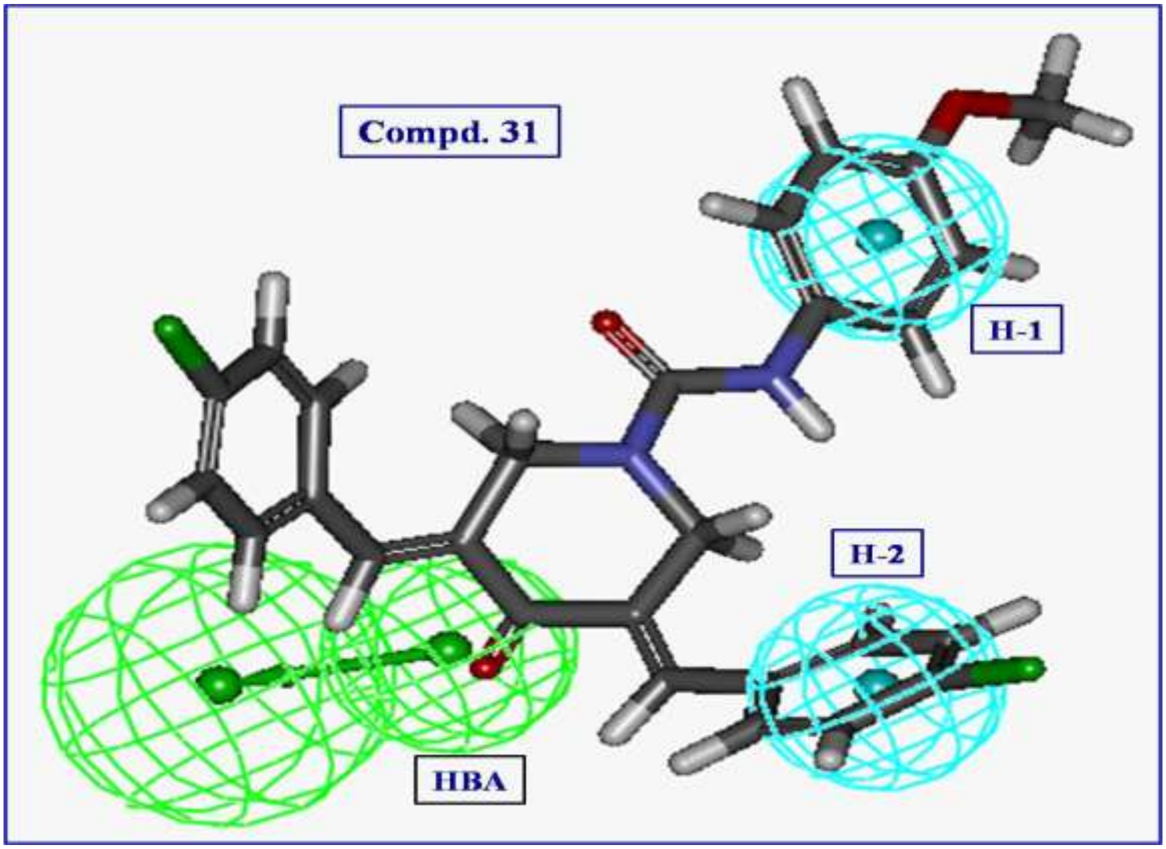
Compd. 31
H-1
H-2
HBA

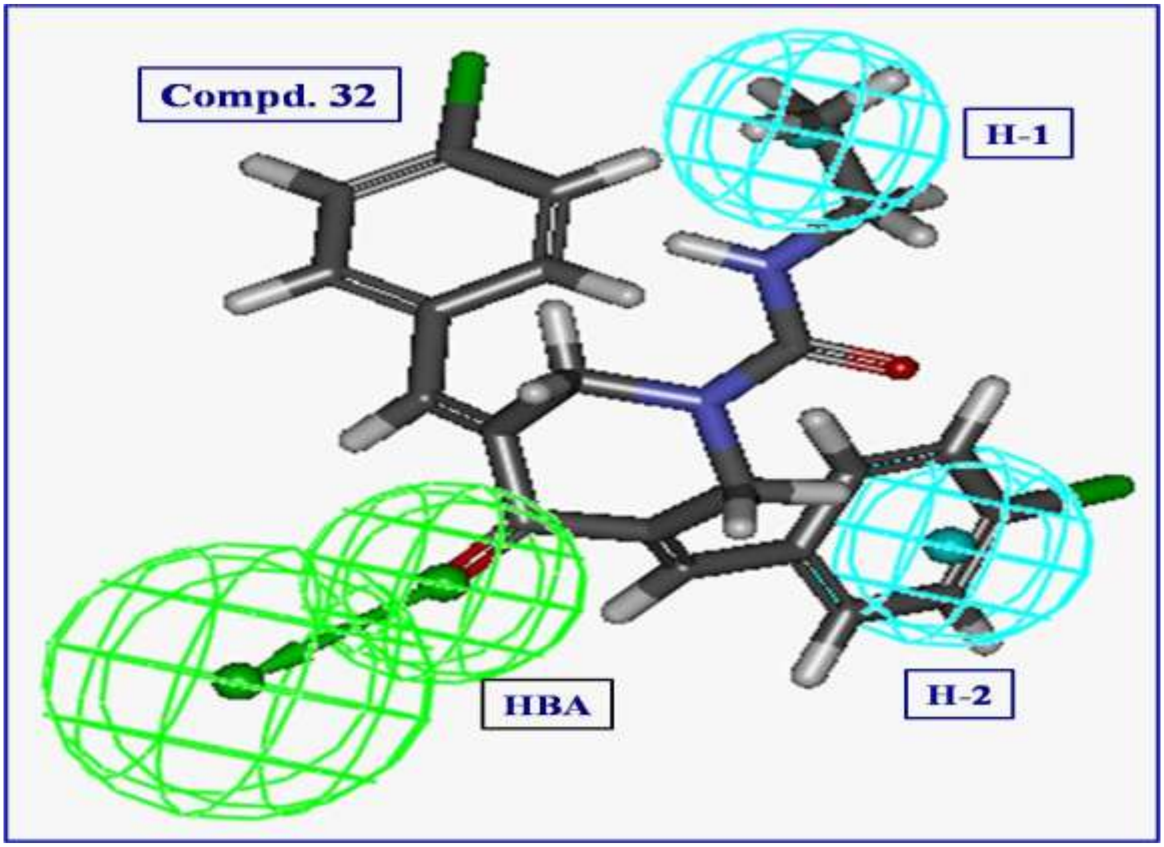
Compd. 32
H-1
HBA
H-2

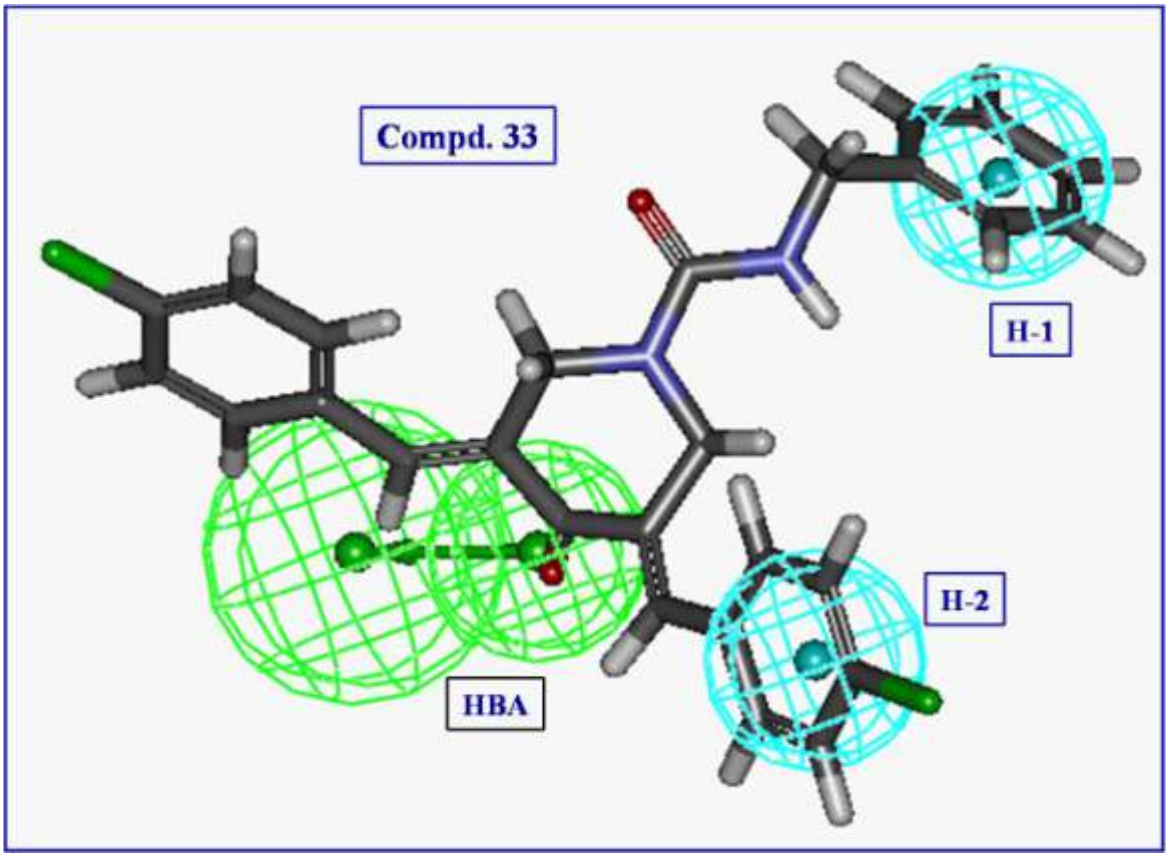
Compd. 33
H-1
H-2
HBA

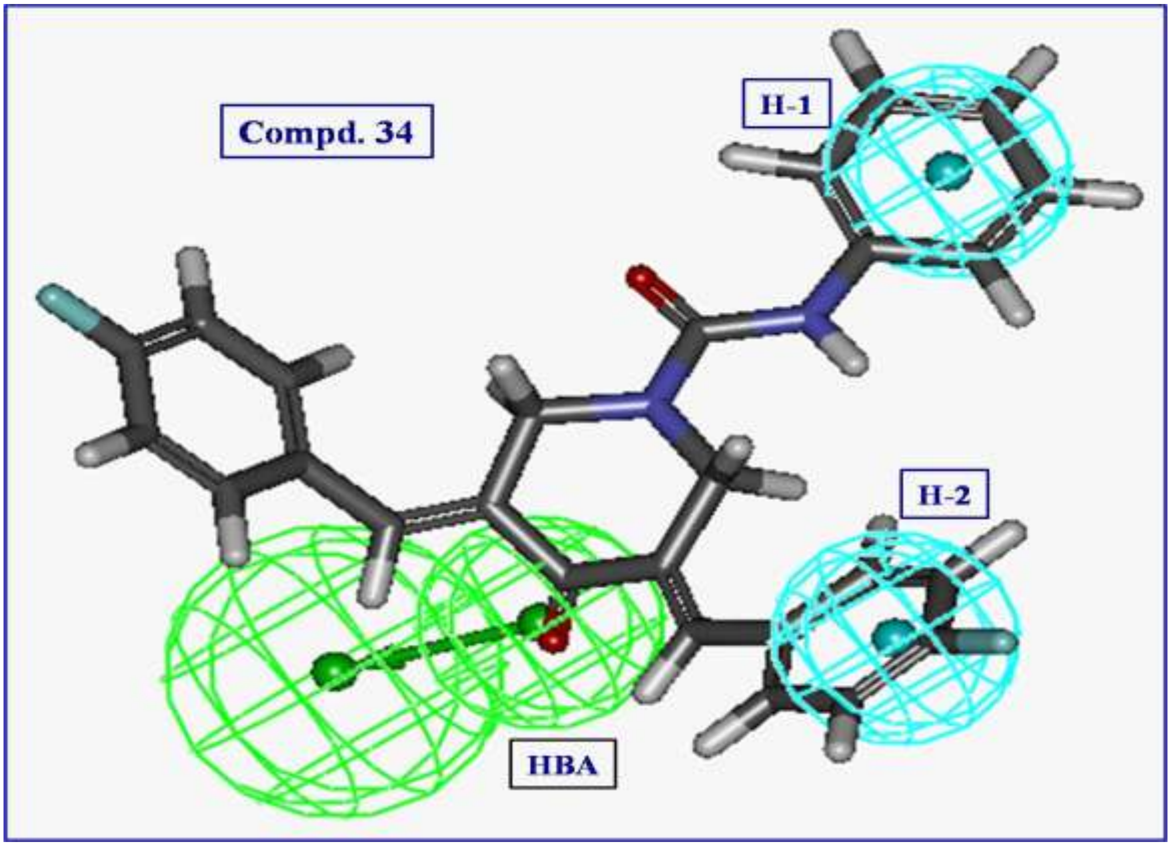
Compd. 34
H-1
H-2
HBA

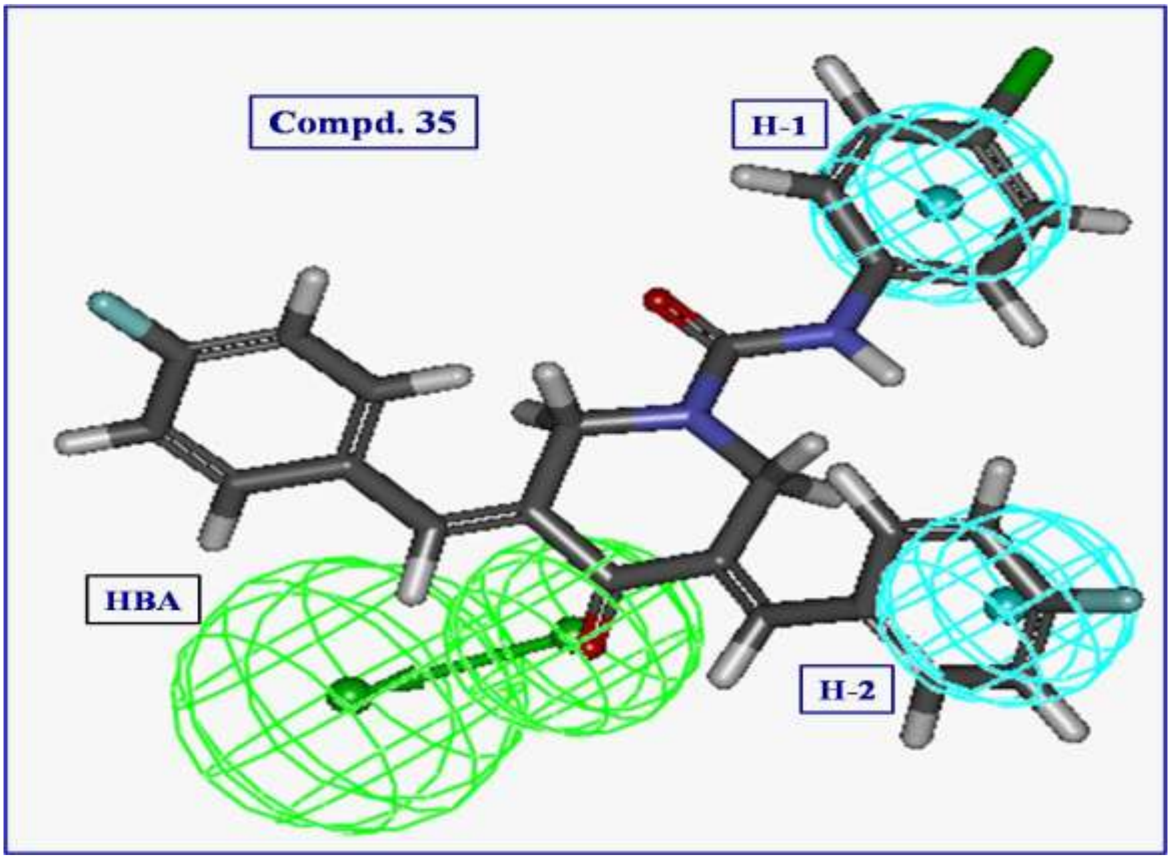
Compd. 35
H-1
HBA
H-2

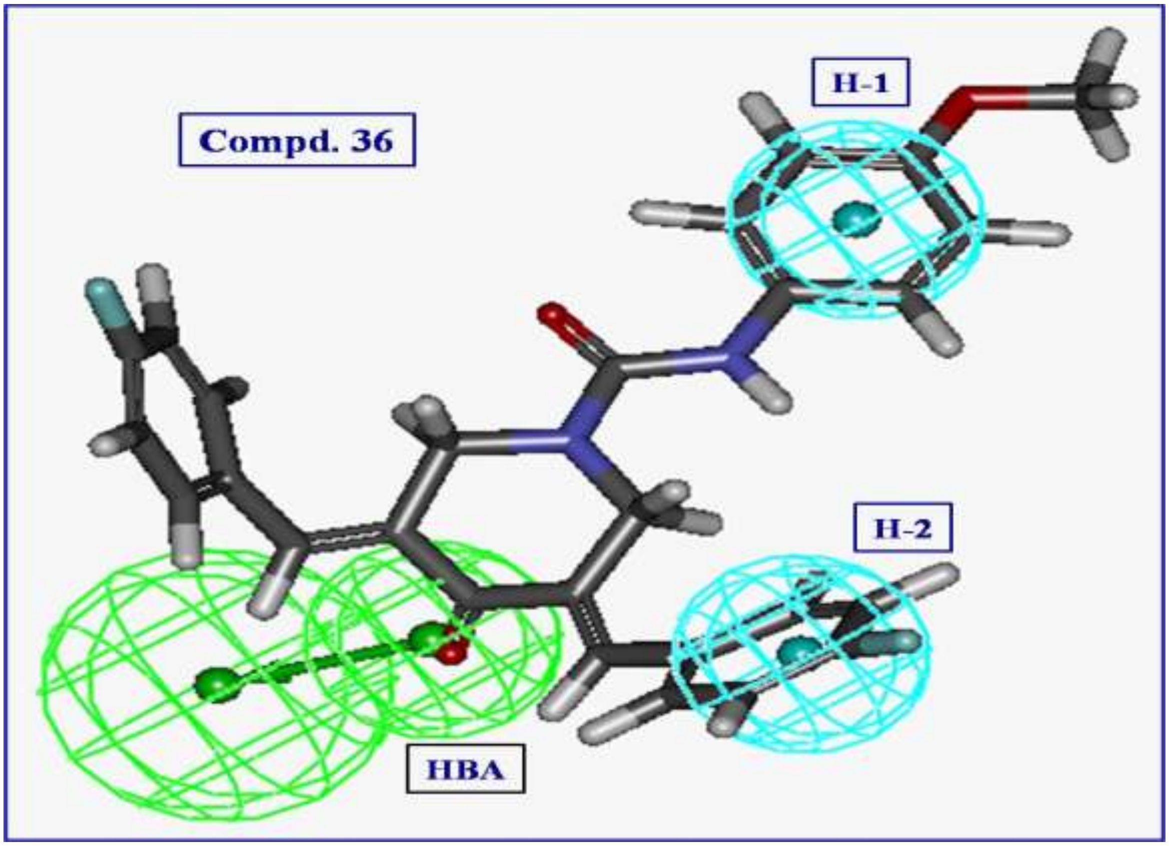
Compd. 36
H-1
H-2
HBA

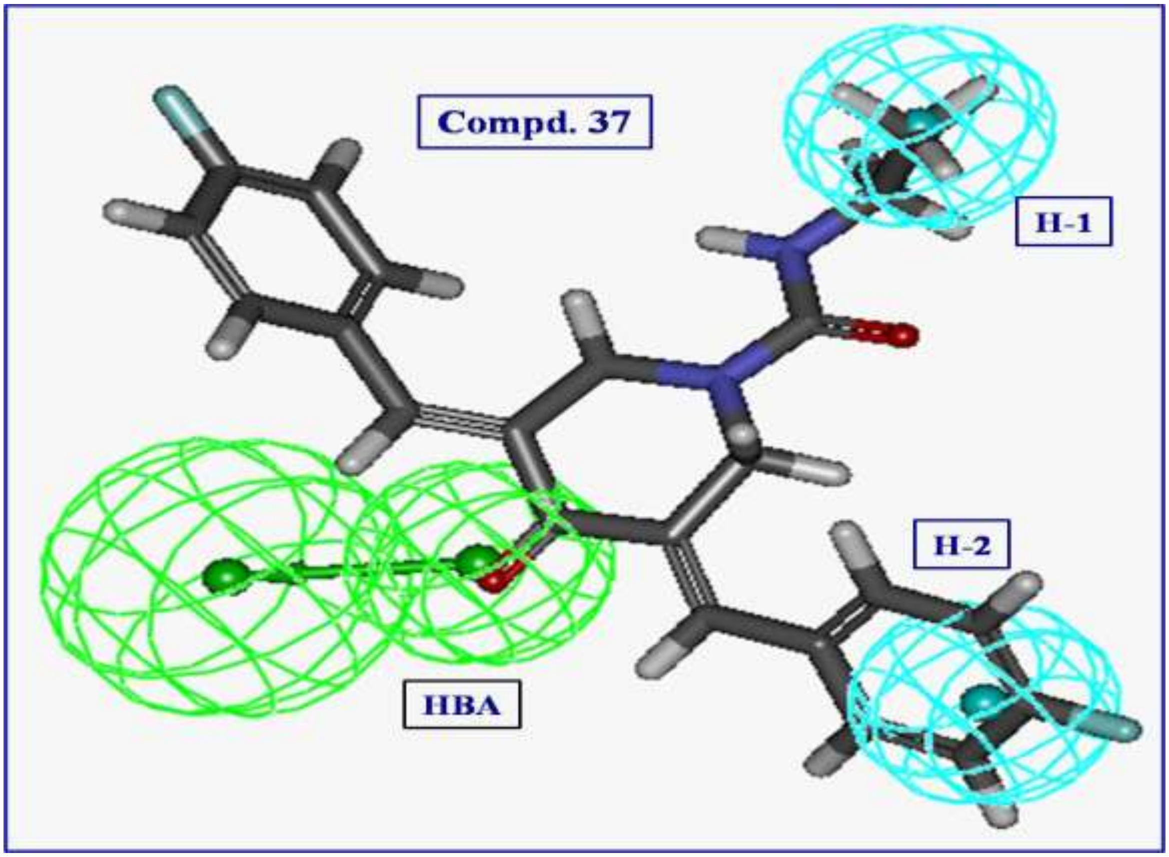
Compd. 37
H-1
H-2
HBA

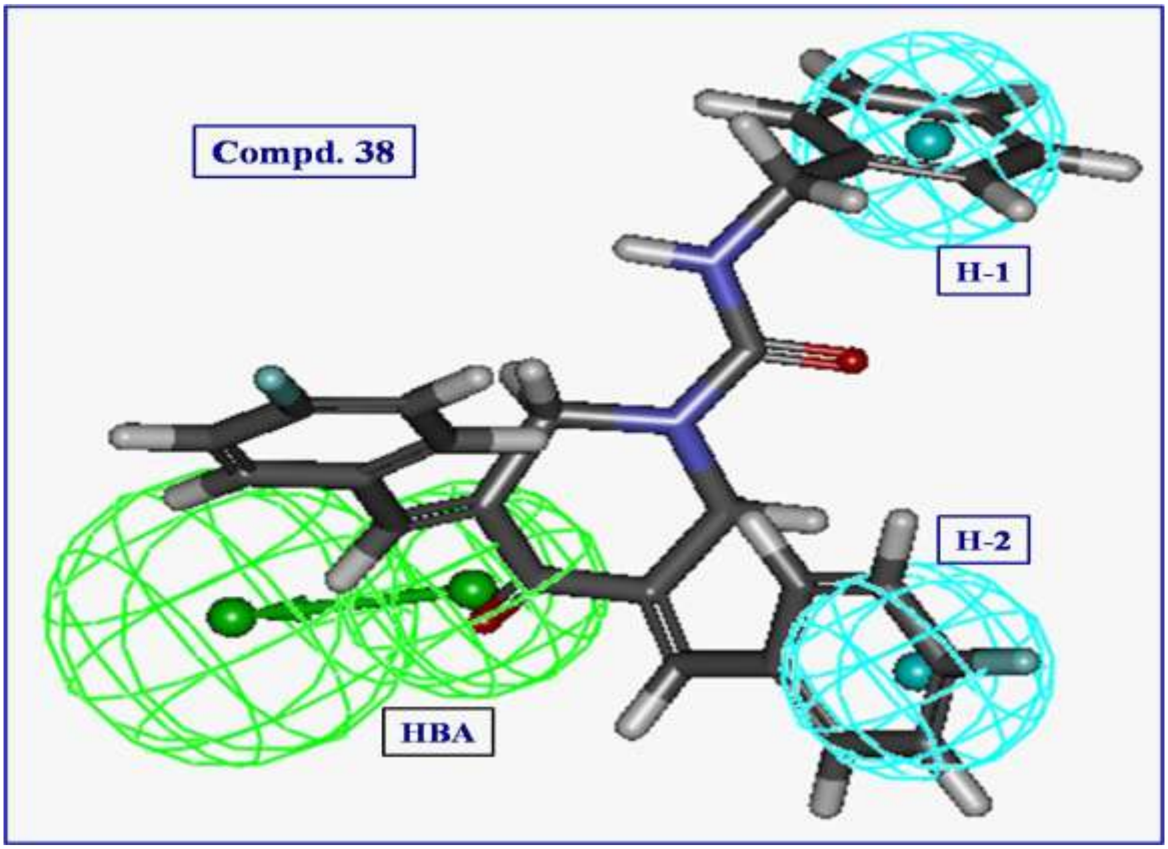
Compd. 38
H-1
H-2
HBA

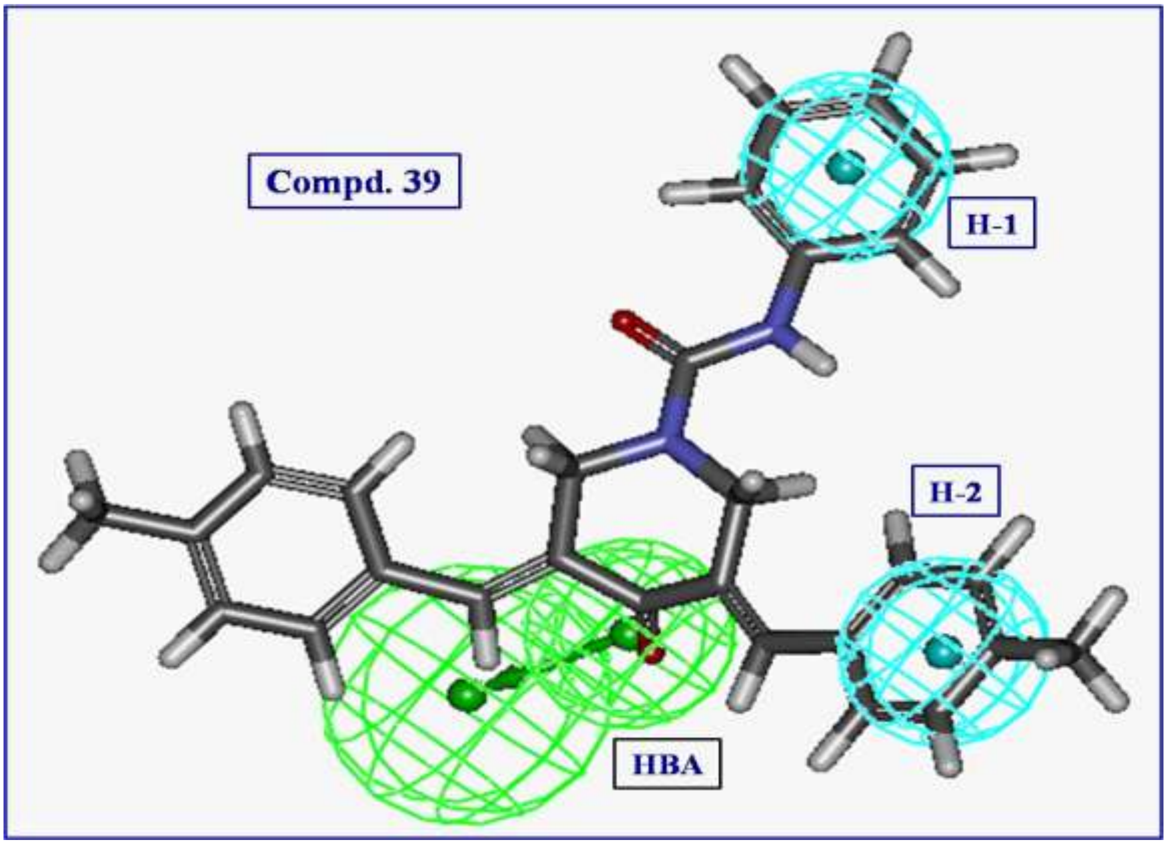
Compd. 39
H-1
H-2
HBA

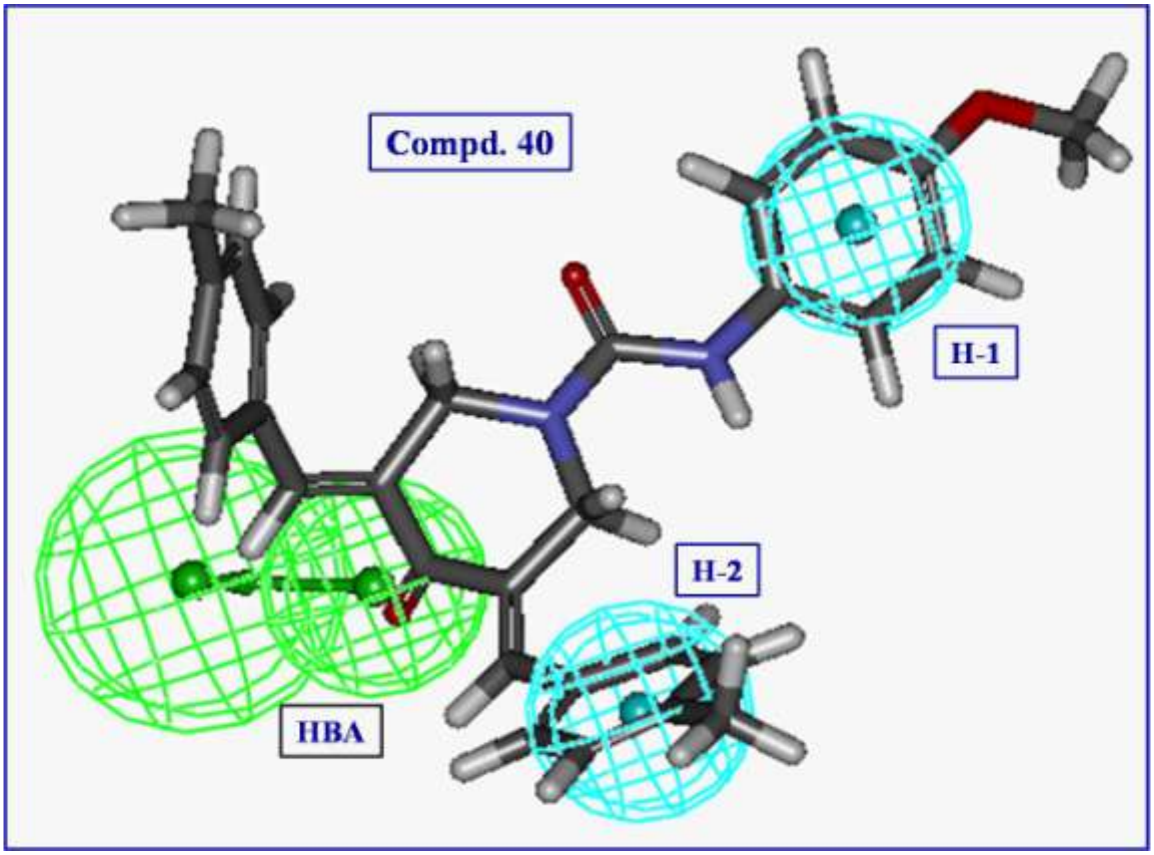
Compd. 40
H-1
H-2
HBA

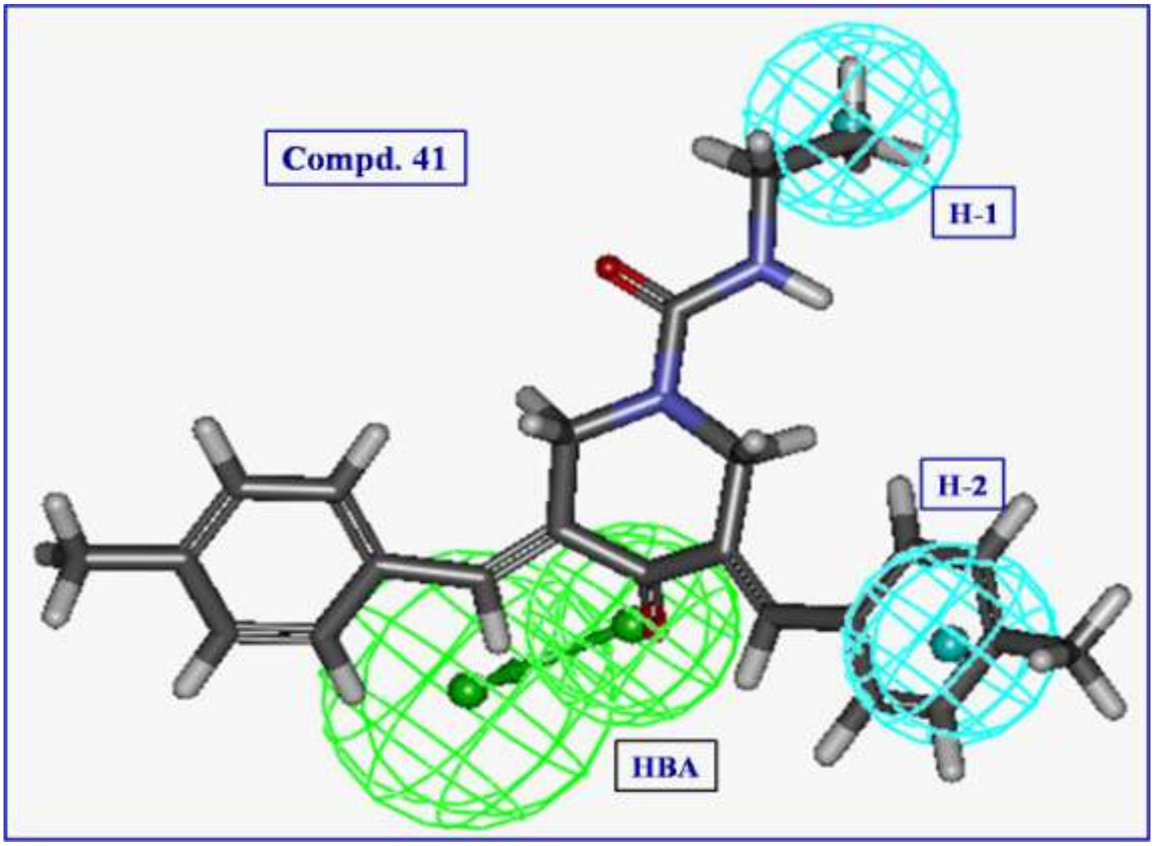
Compd. 41
H-1
H-2
HBA

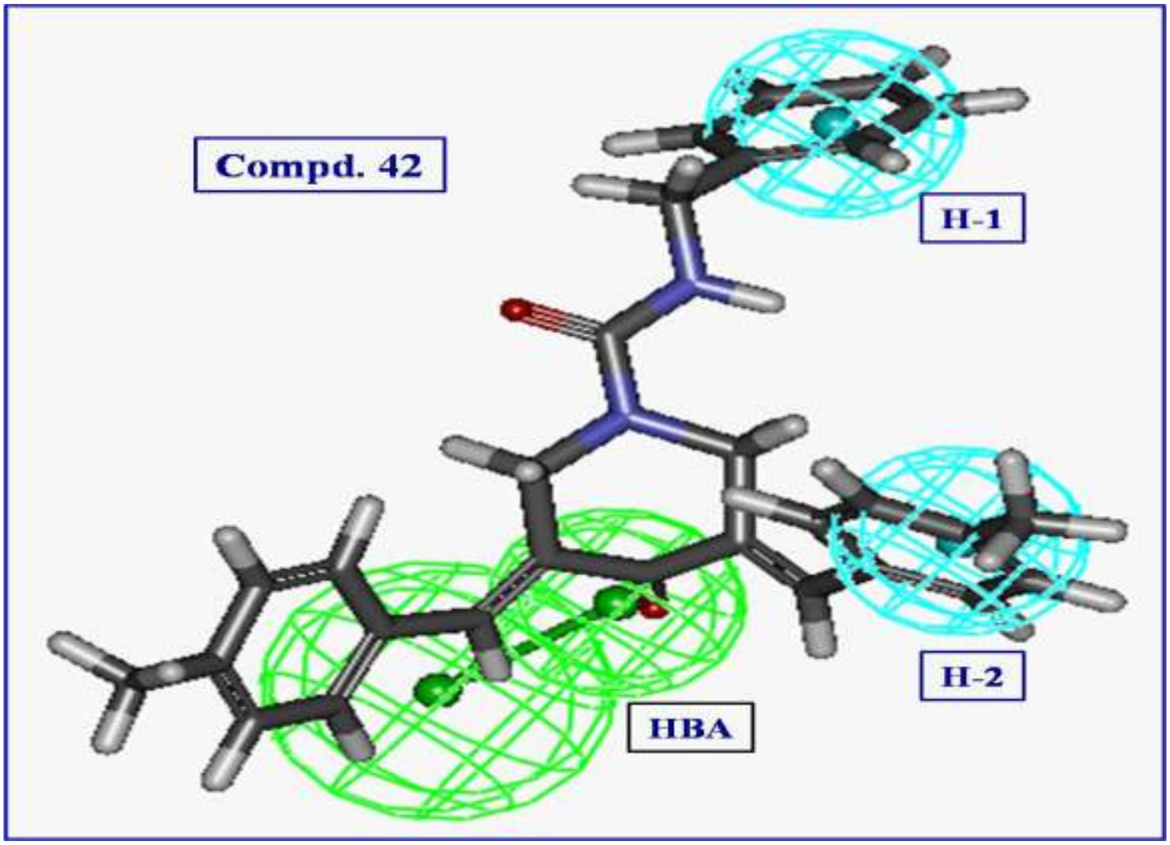
Compd. 42
H-1
H-2
HBA

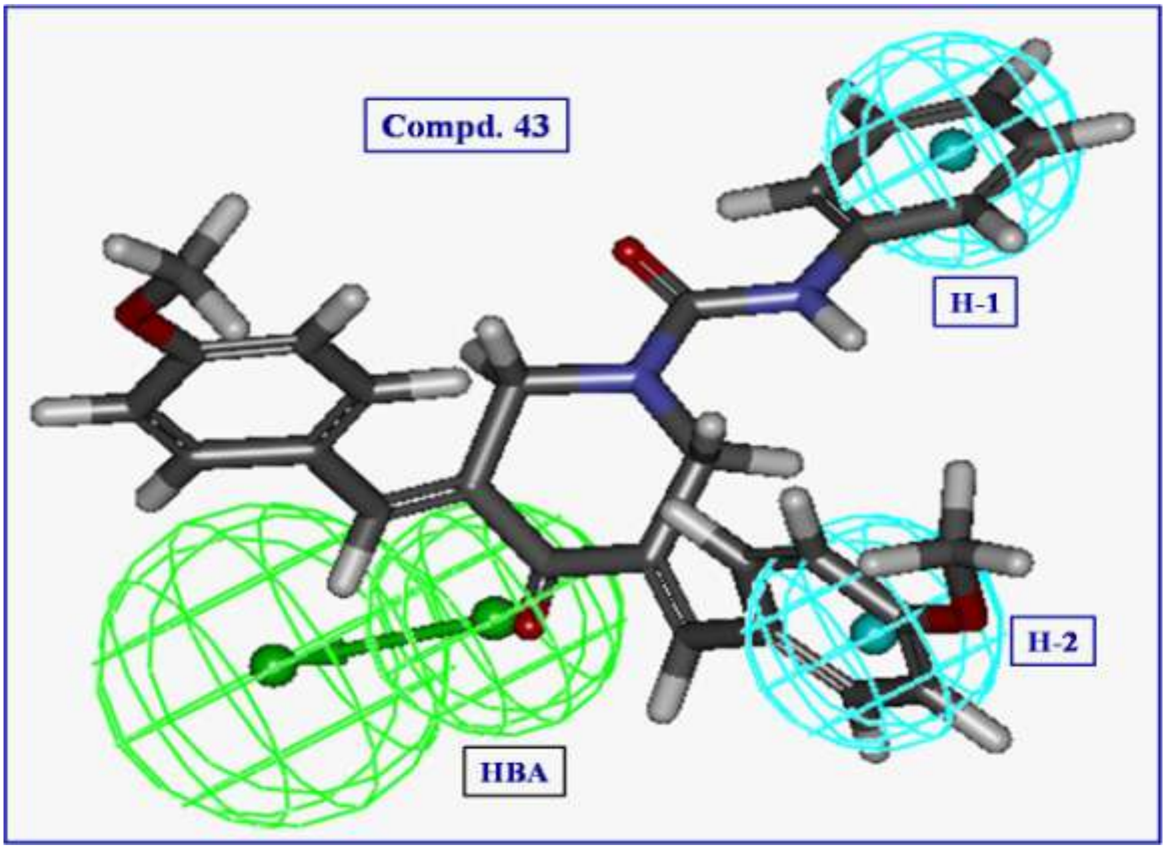
Compd. 43
H-1
H-2
HBA

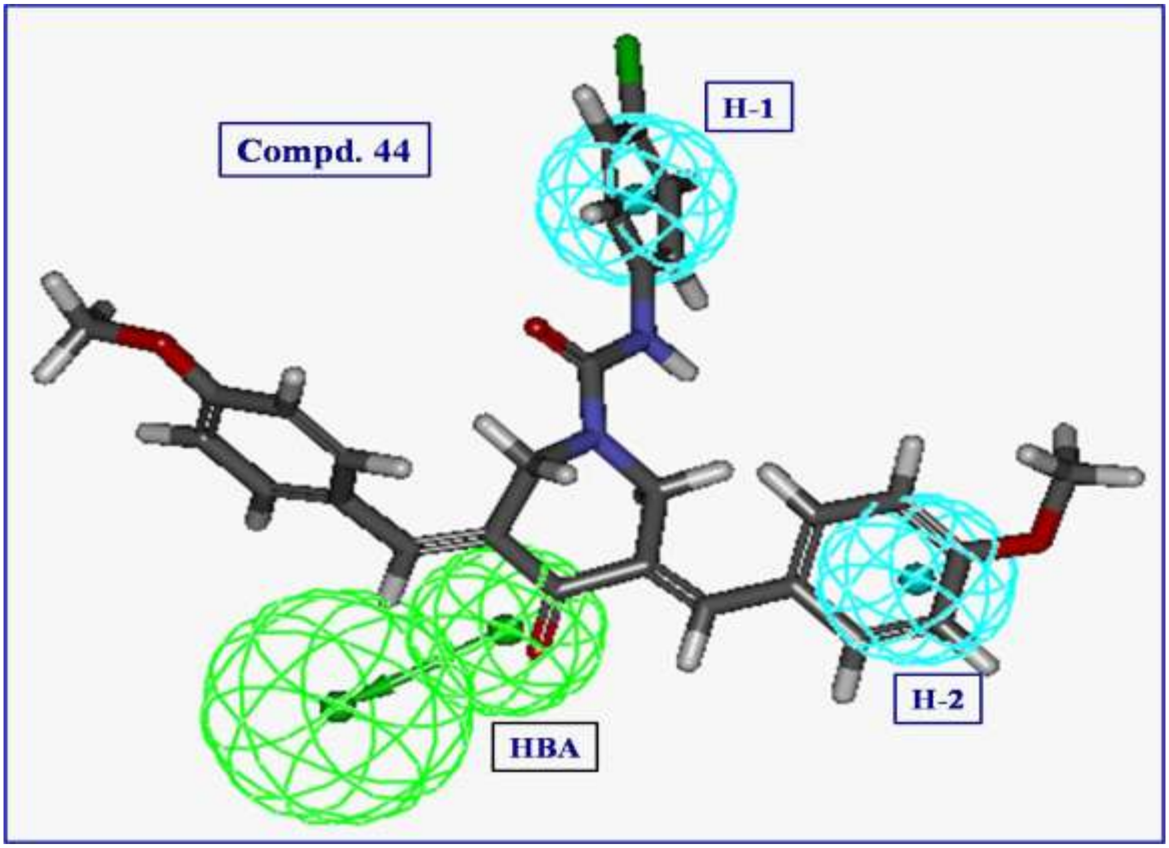
Compd. 44
H-1
H-2
HBA

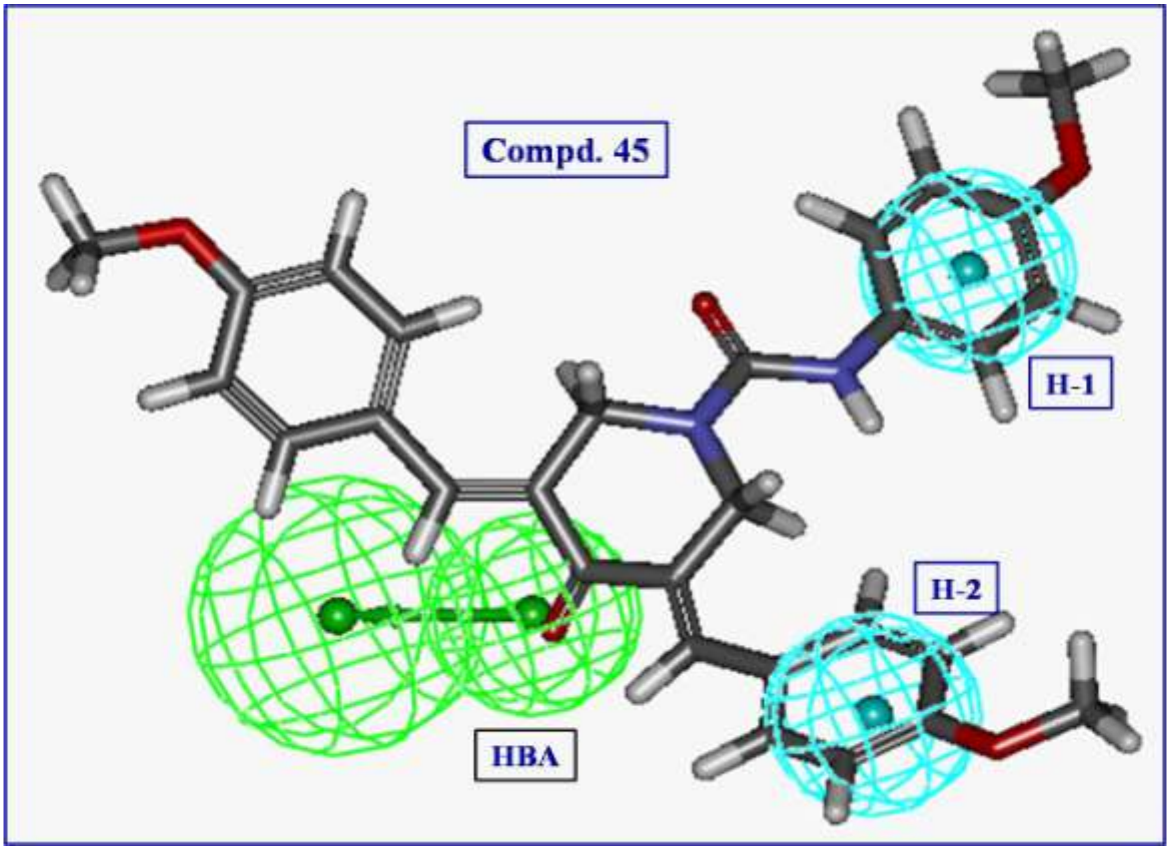
Compd. 45
H-1
H-2
HBA

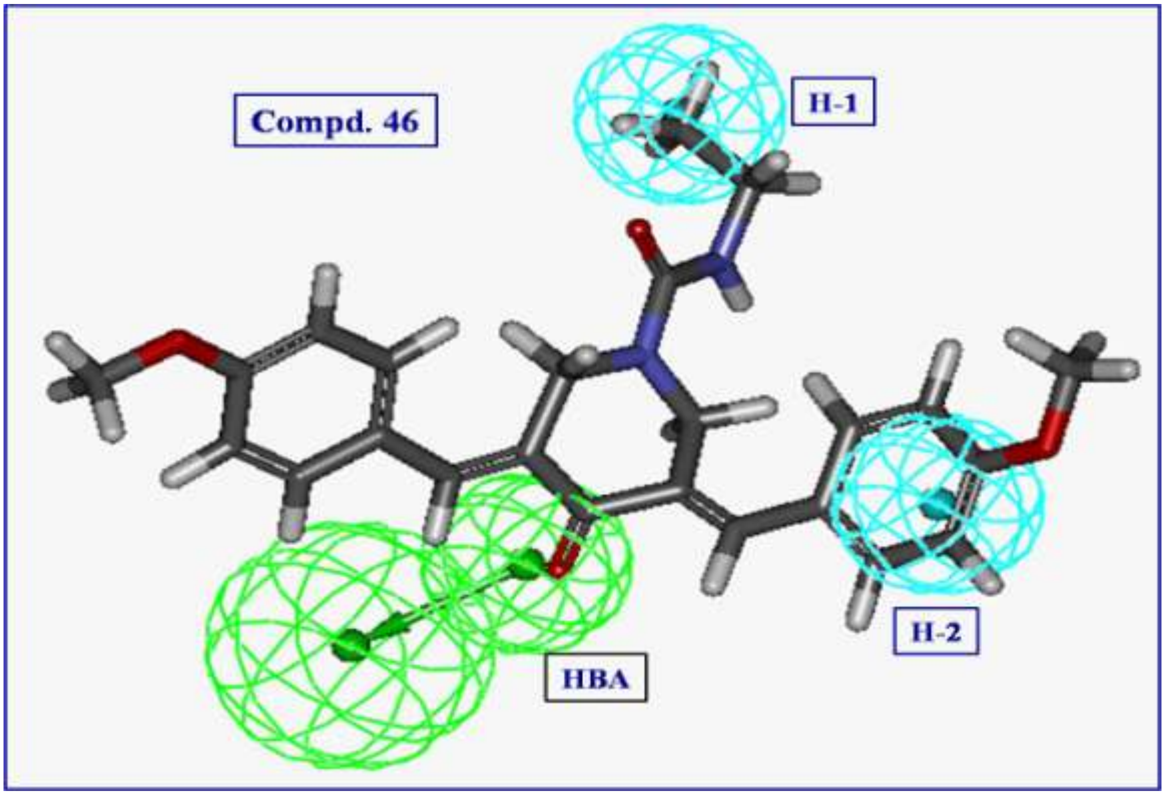

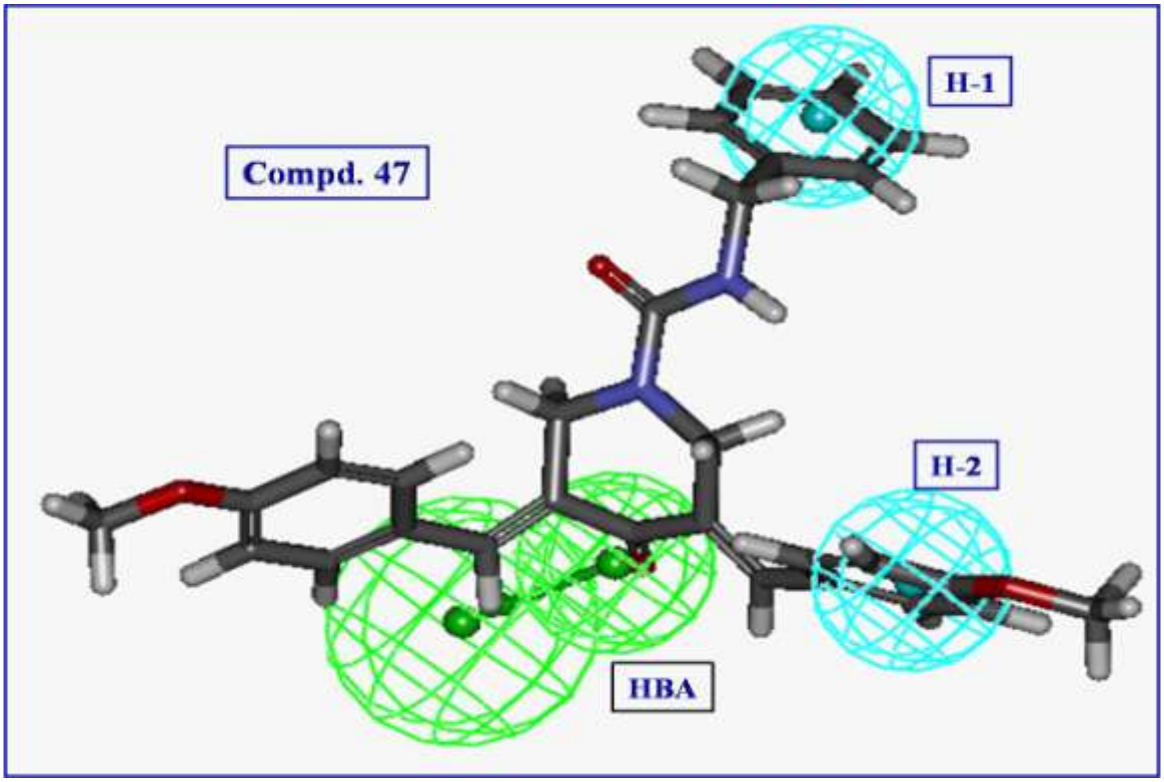

Fig. S105. 3D-pharmacophore model mapped on the tested piperidinecarboxamides **24–47** against MCF7 (breast) carcinoma cell line.

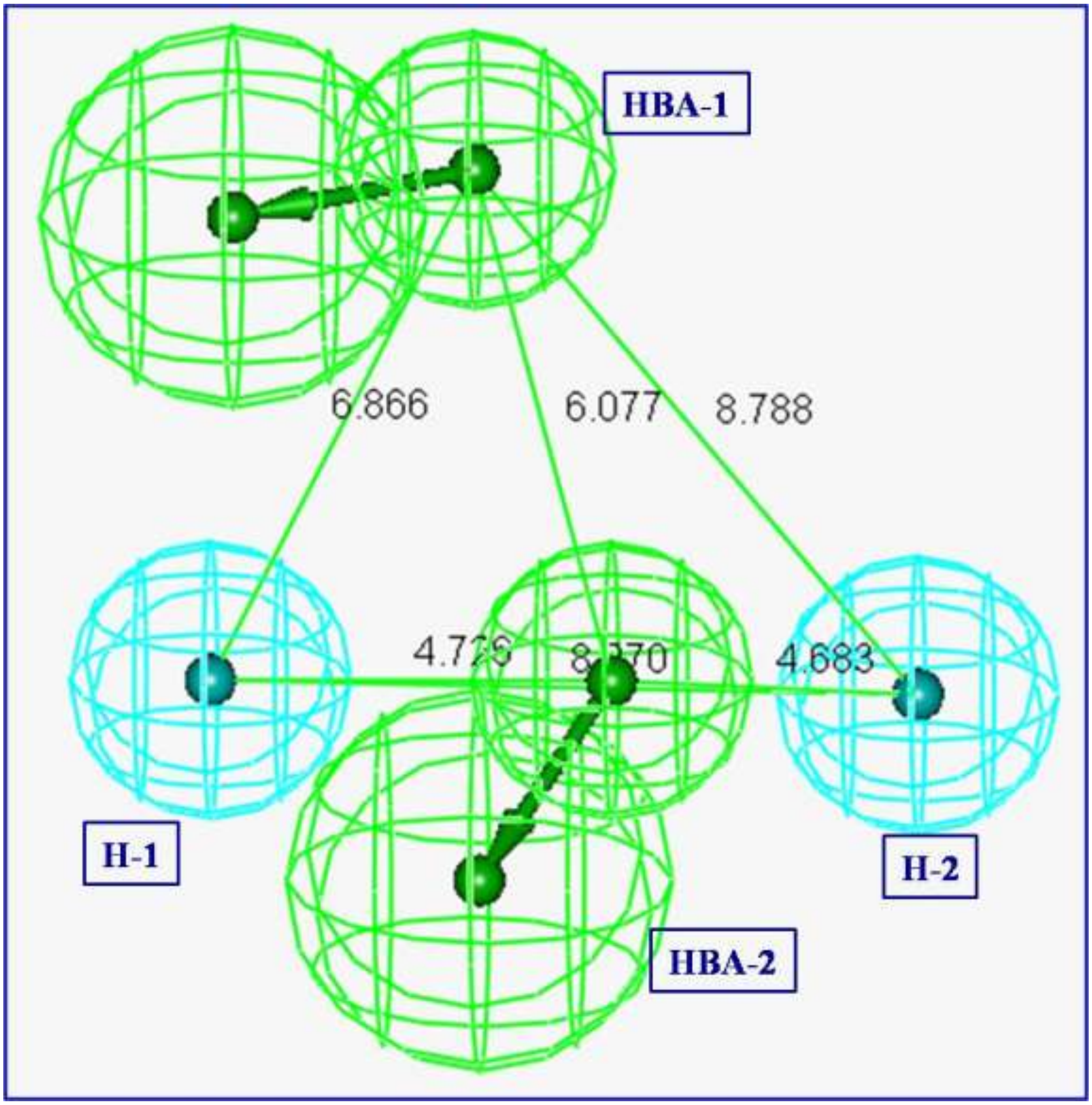

Fig. S106. Constraint distances "H-1 – H-2 = 8.270, H-1 – HBA-1 = 6.866, H-1 – HBA-2 = 4.726, H-2 – HBA-1 = 8.788, H-2 – HBA-2 = 4.683, HBA-1 – HBA-2 = 6.077 Å" of the generated 3D-pharmacophore for the tested piperidinecarboxamides **24–47** against A431 (squamous) carcinoma cell line which contains two hydrophobics (H-1, H-2; light blue) and two hydrogen bonding acceptor (HBA-1, HBA-2; green).

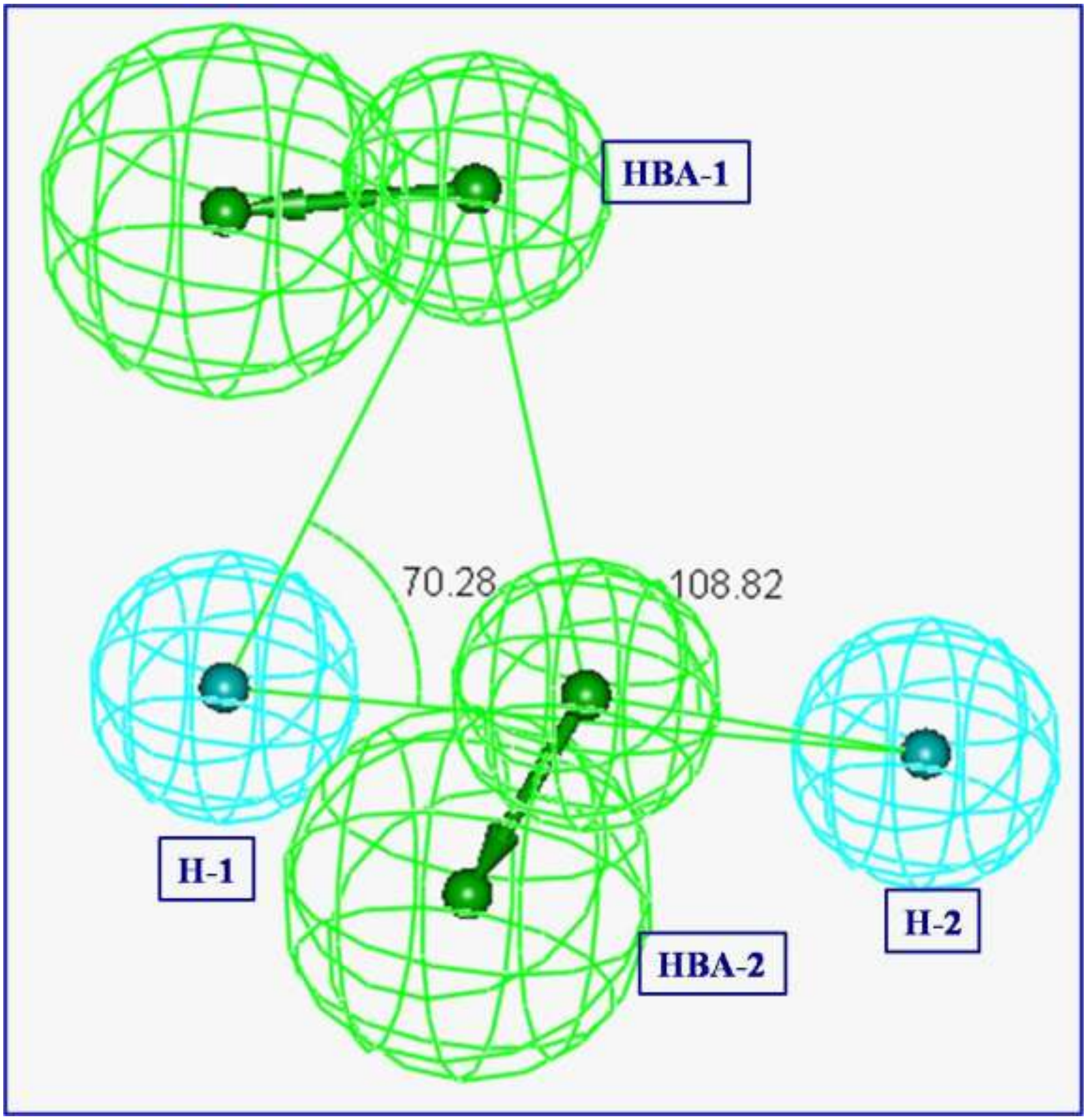

Fig. S107. Constraint angles "H-2 – H-1 – HBA-1 = 70.28, H-2 – HBA-2 – HBA-1 = 108.82 °" of the generated 3D-pharmacophore for the tested piperidinecarboxamides **24–47** against A431 (squamous) carcinoma cell line which contains two hydrophobics (H-1, H-2; light blue) and two hydrogen bonding acceptor (HBA-1, HBA-2; green).

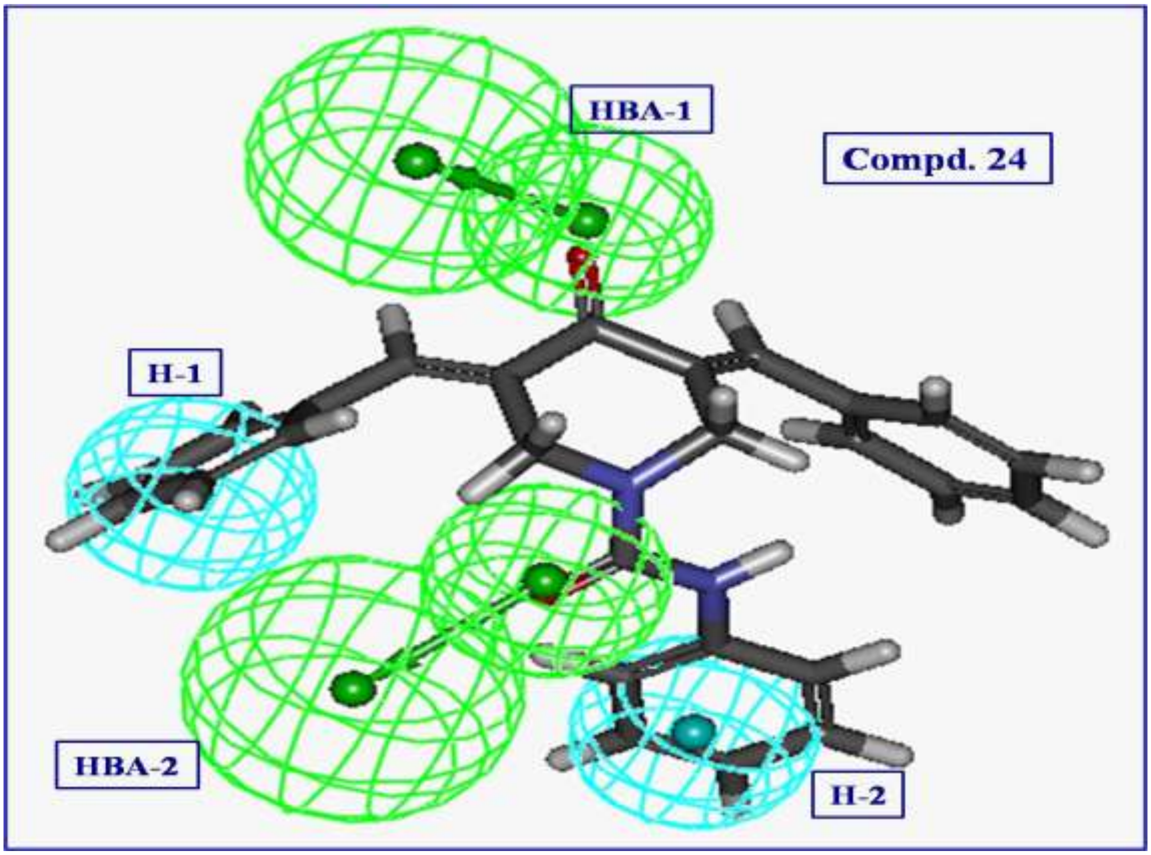
HBA-1
Compd. 24
H-1
HBA-2
H-2

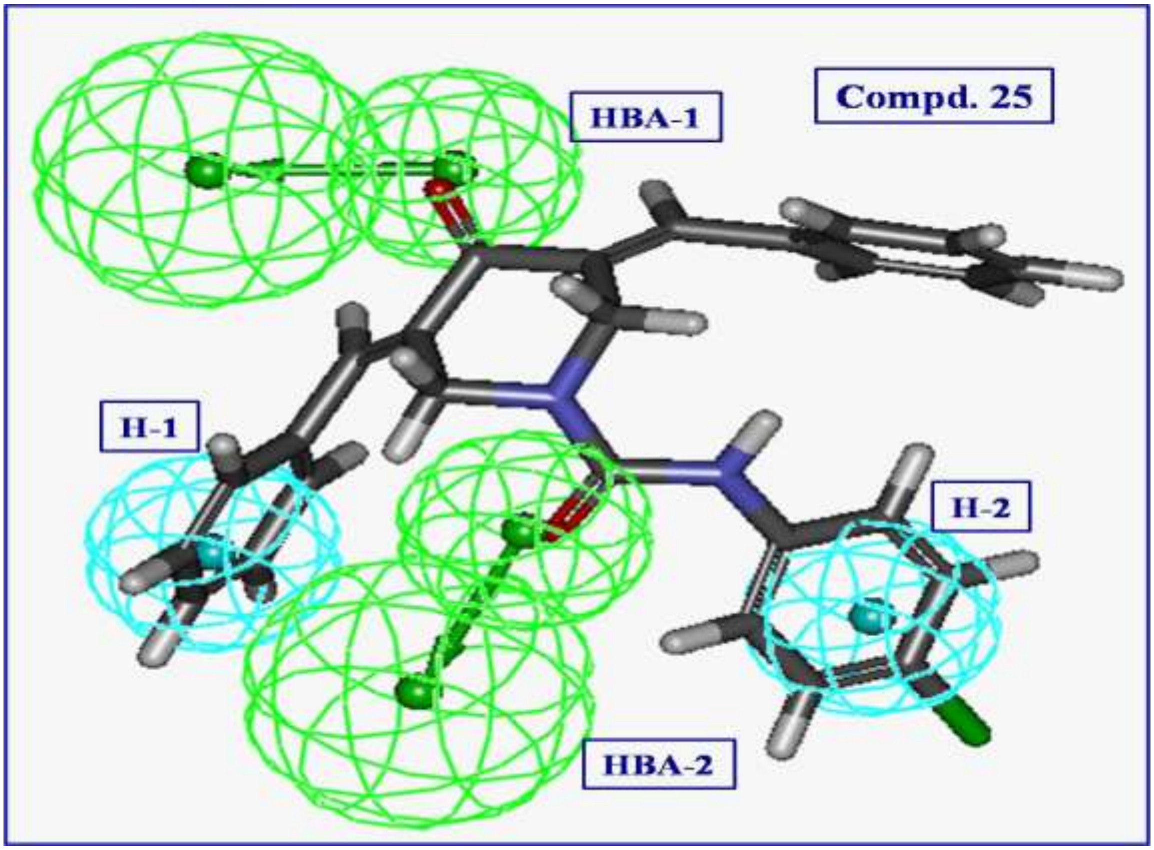
HBA-1
Compd. 25
H-1
H-2
HBA-2

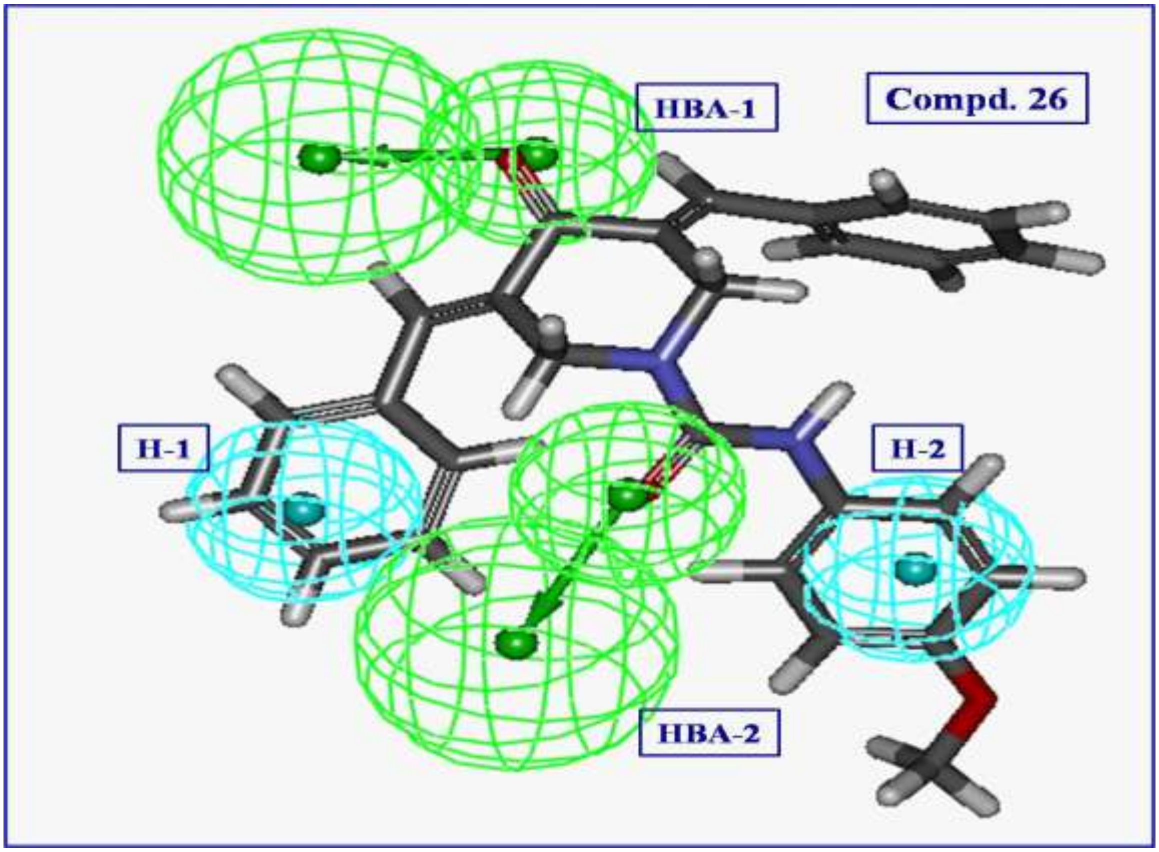
HBA-1
Compd. 26
H-1
H-2
HBA-2

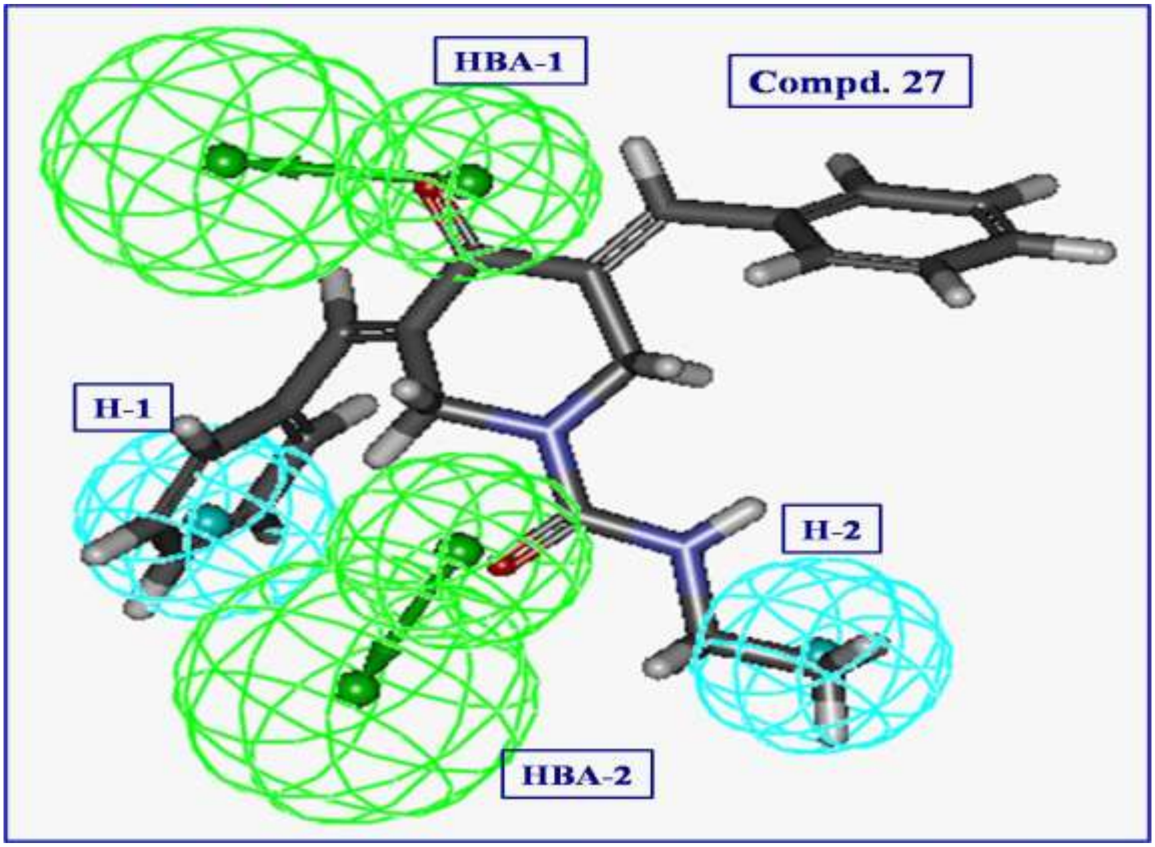
HBA-1
Compd. 27
H-1
H-2
HBA-2

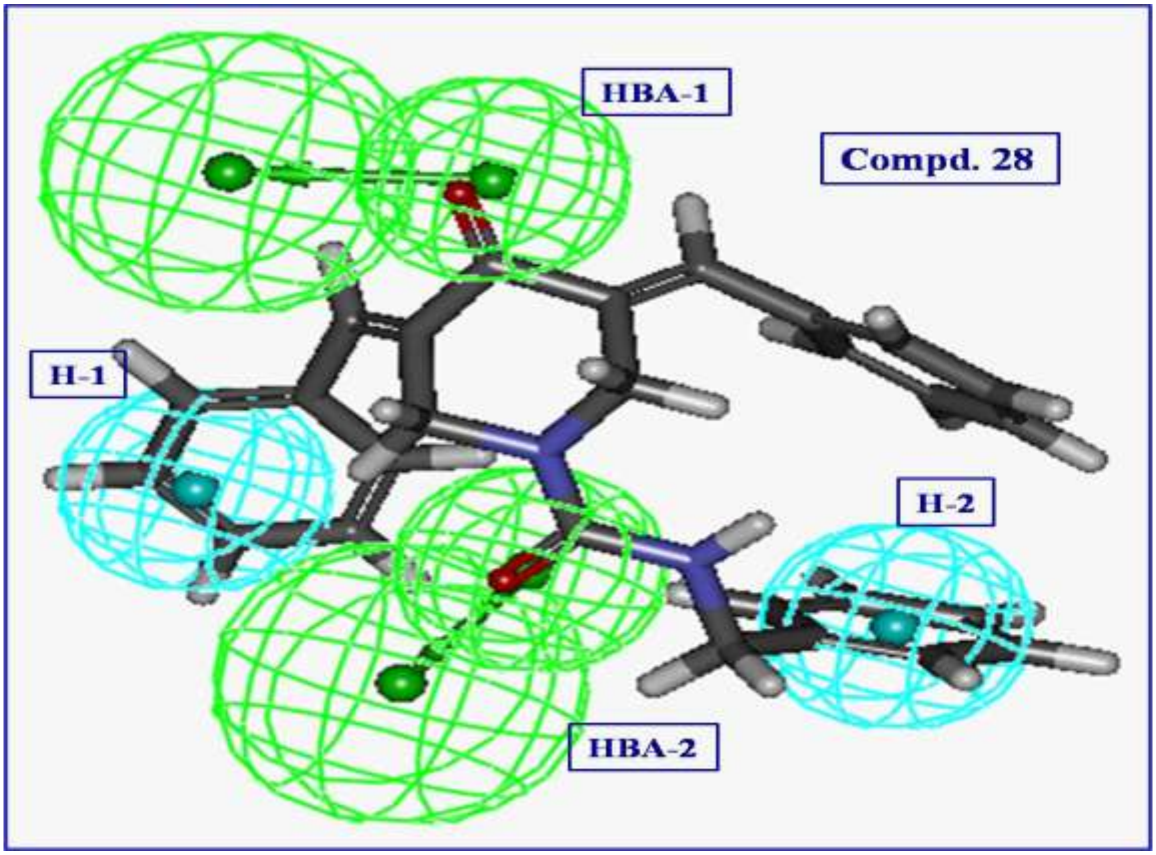
HBA-1
Compd. 28
H-1
H-2
HBA-2

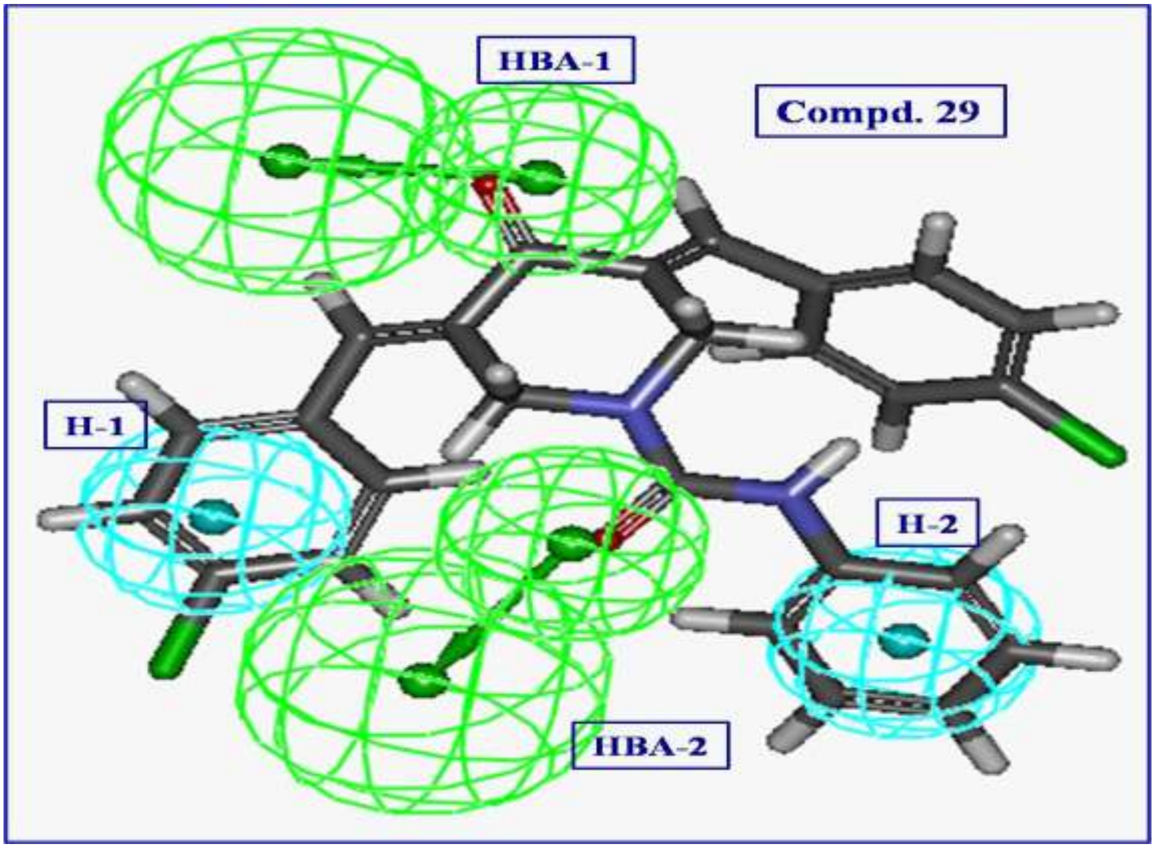
HBA-1
Compd. 29
H-1
H-2
HBA-2

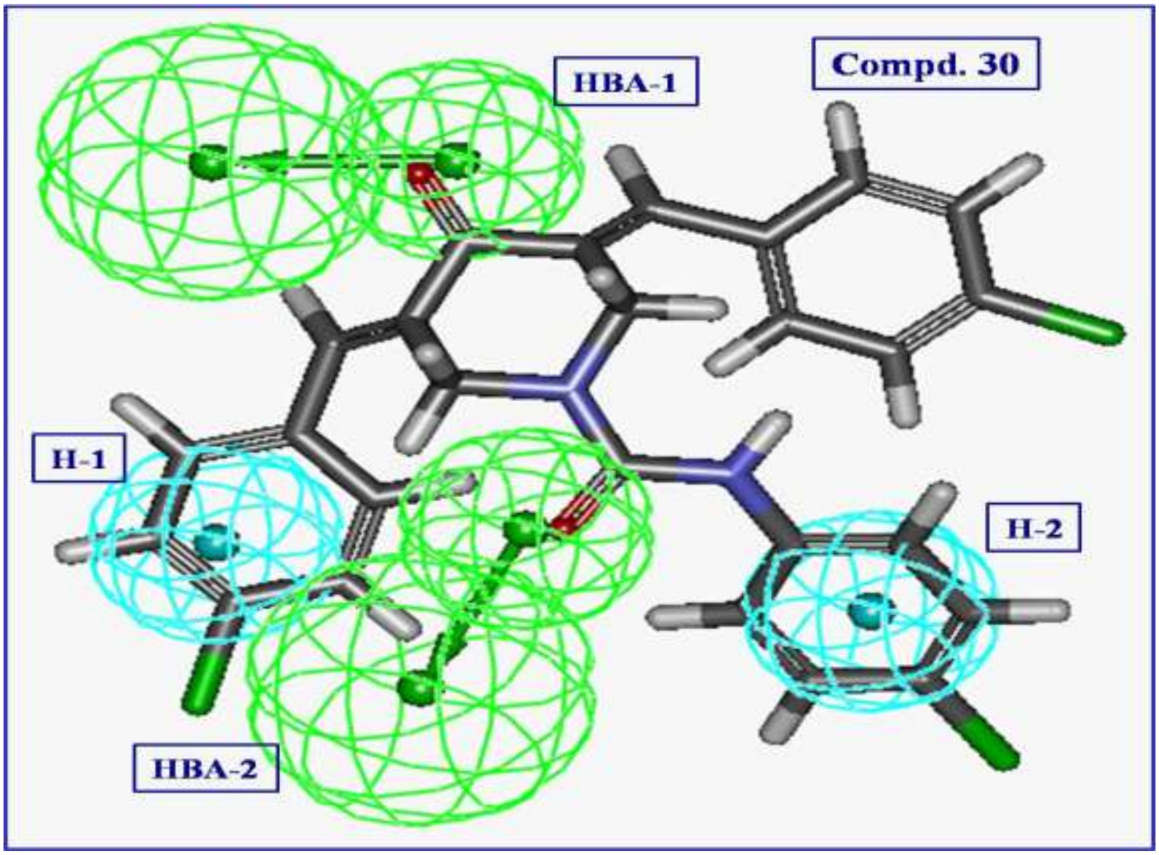
Compd. 30
HBA-1
H-1
H-2
HBA-2

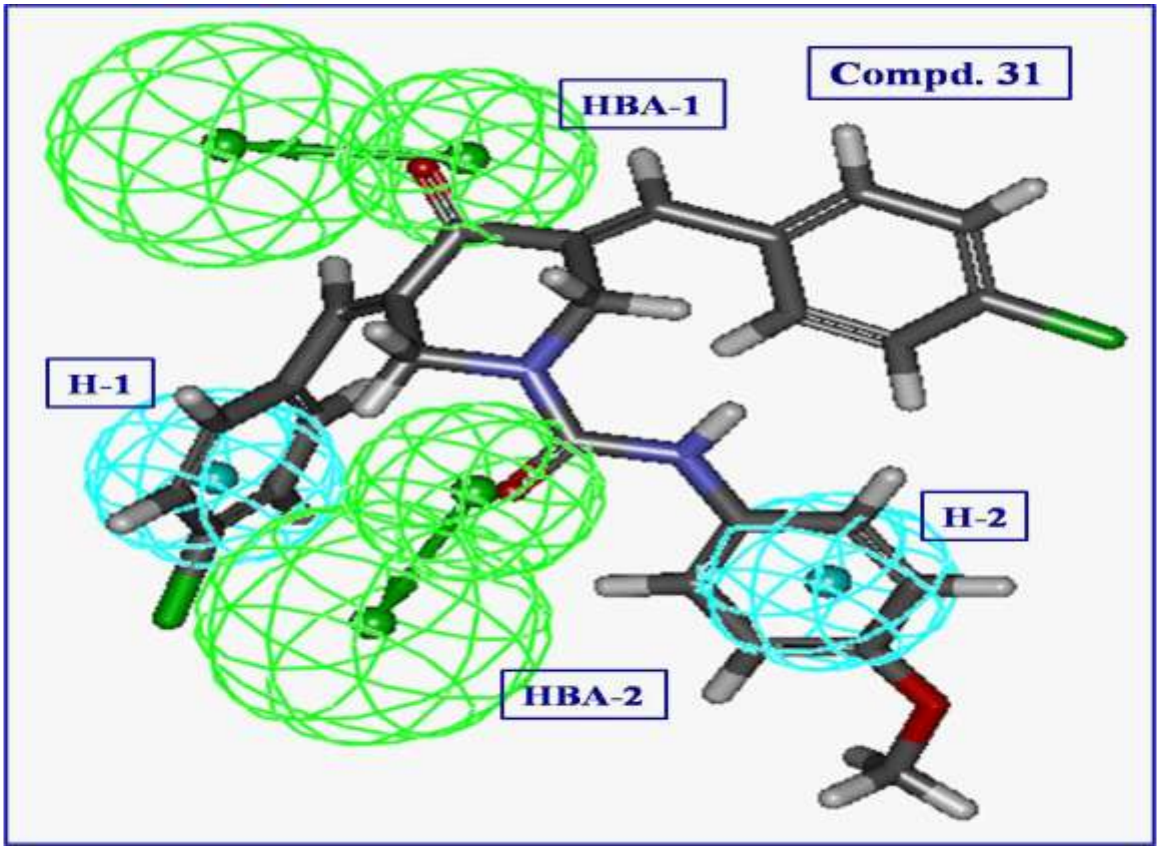
Compd. 31
HBA-1
H-1
H-2
HBA-2

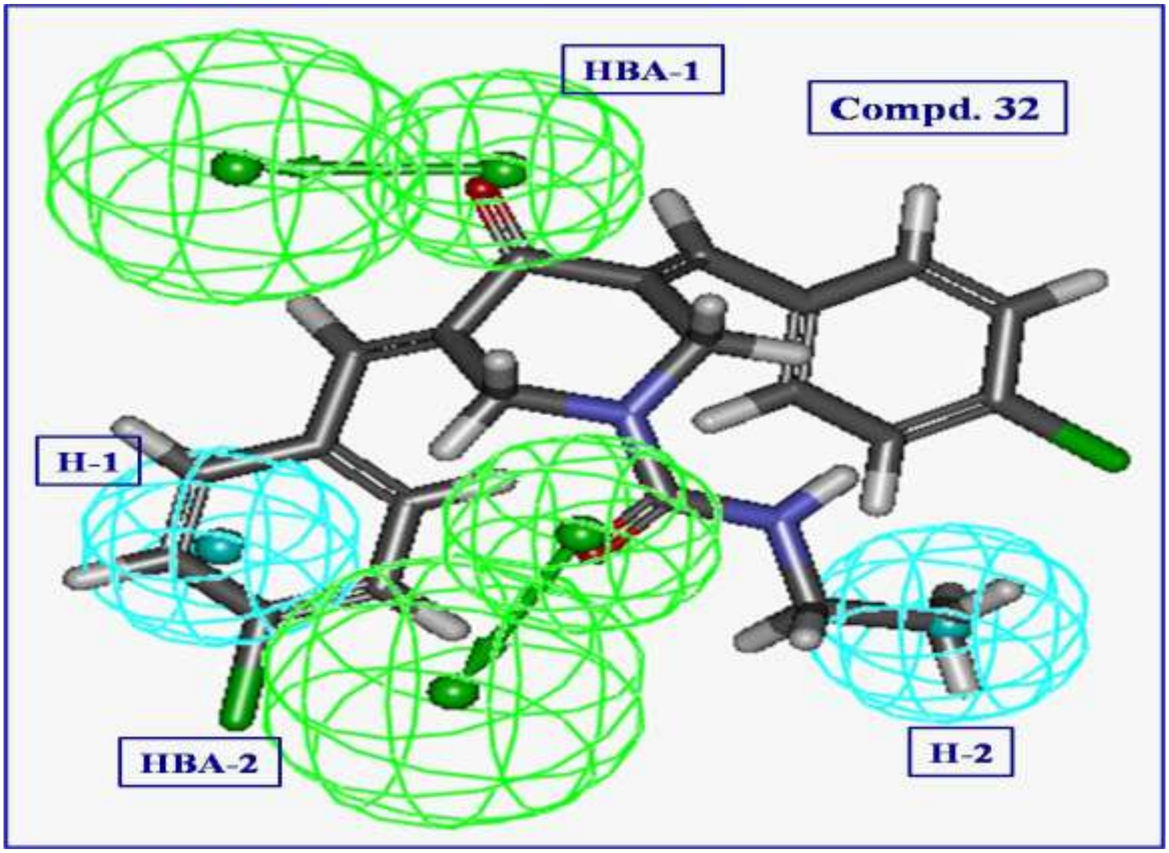
HBA-1
Compd. 32
H-1
HBA-2
H-2

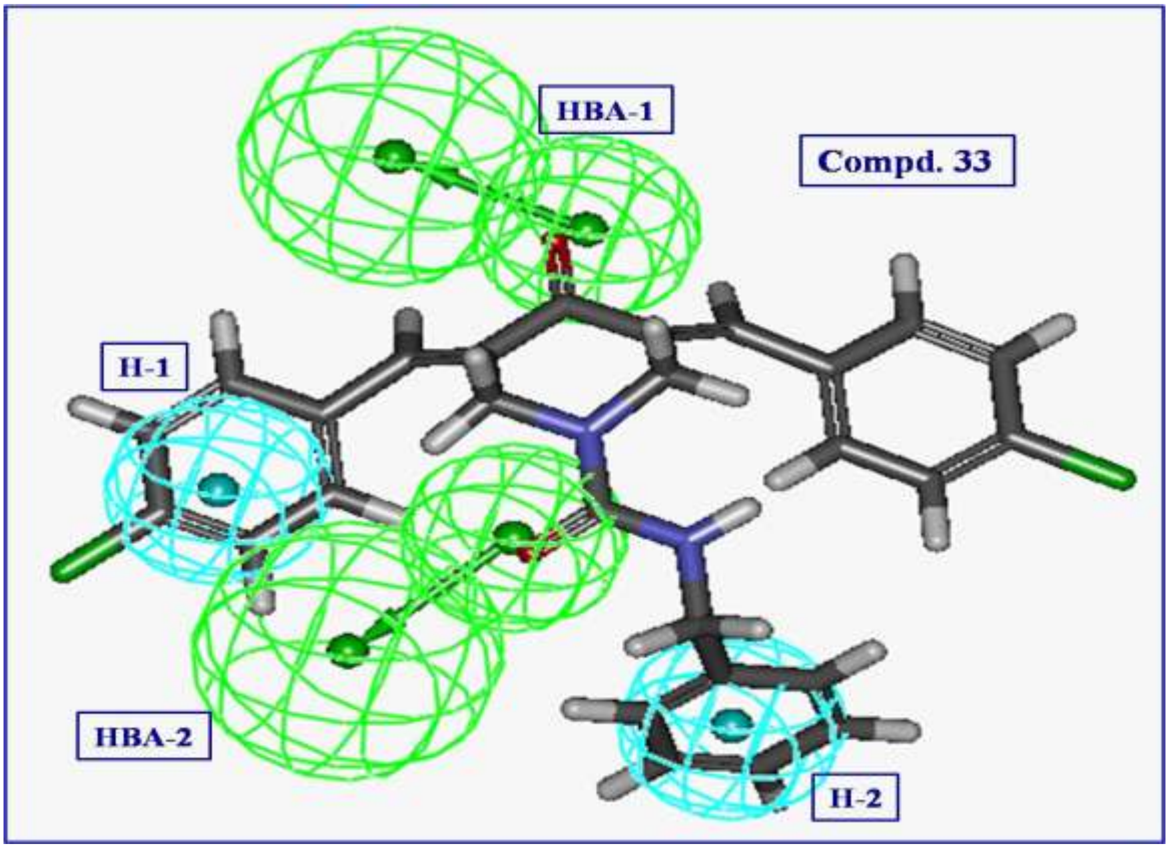
HBA-1
Compd. 33
H-1
HBA-2
H-2

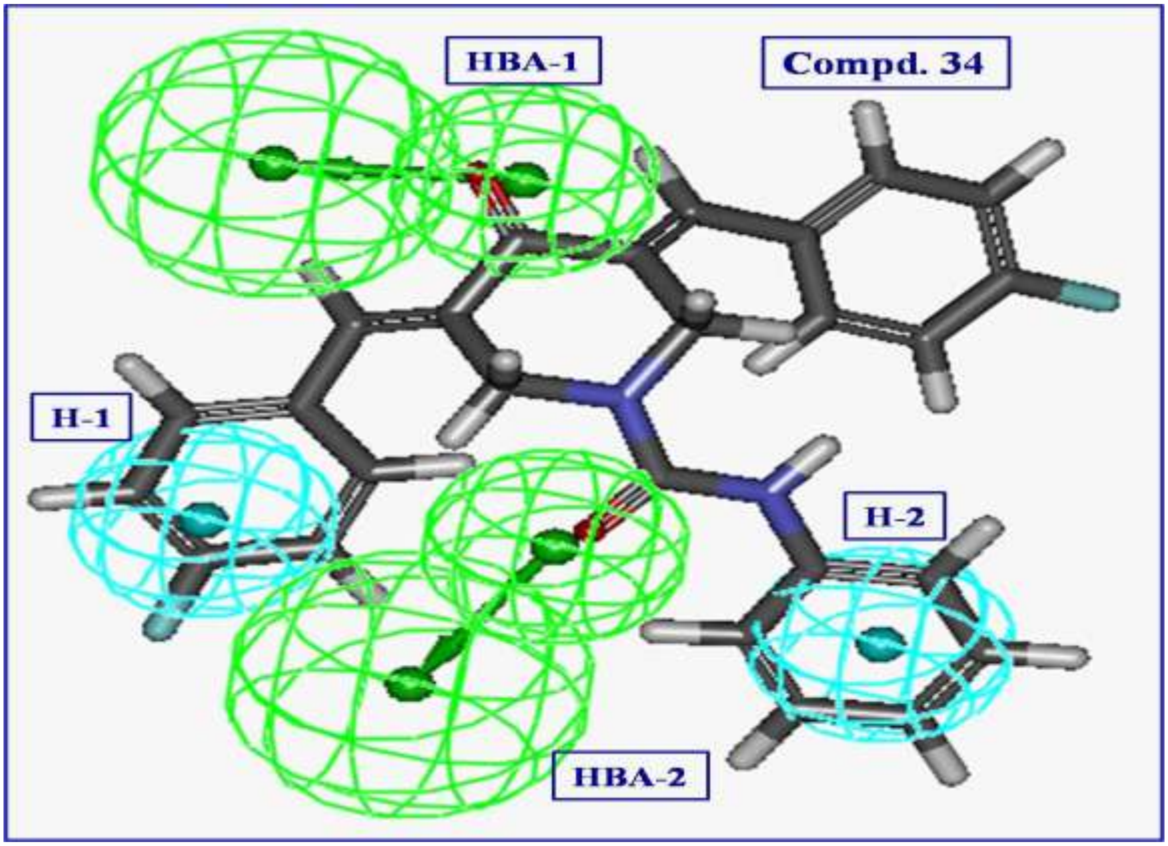
HBA-1
Compd. 34
H-1
H-2
HBA-2

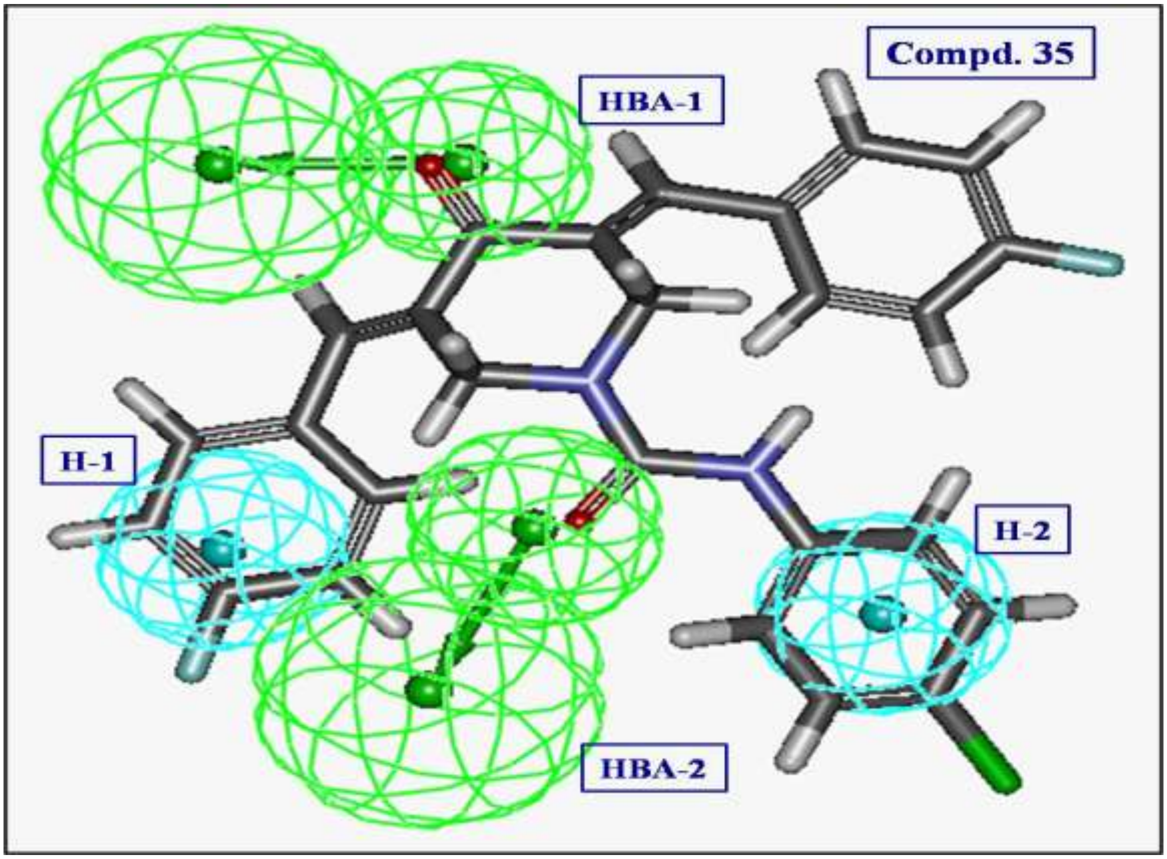
Compd. 35
HBA-1
H-1
H-2
HBA-2

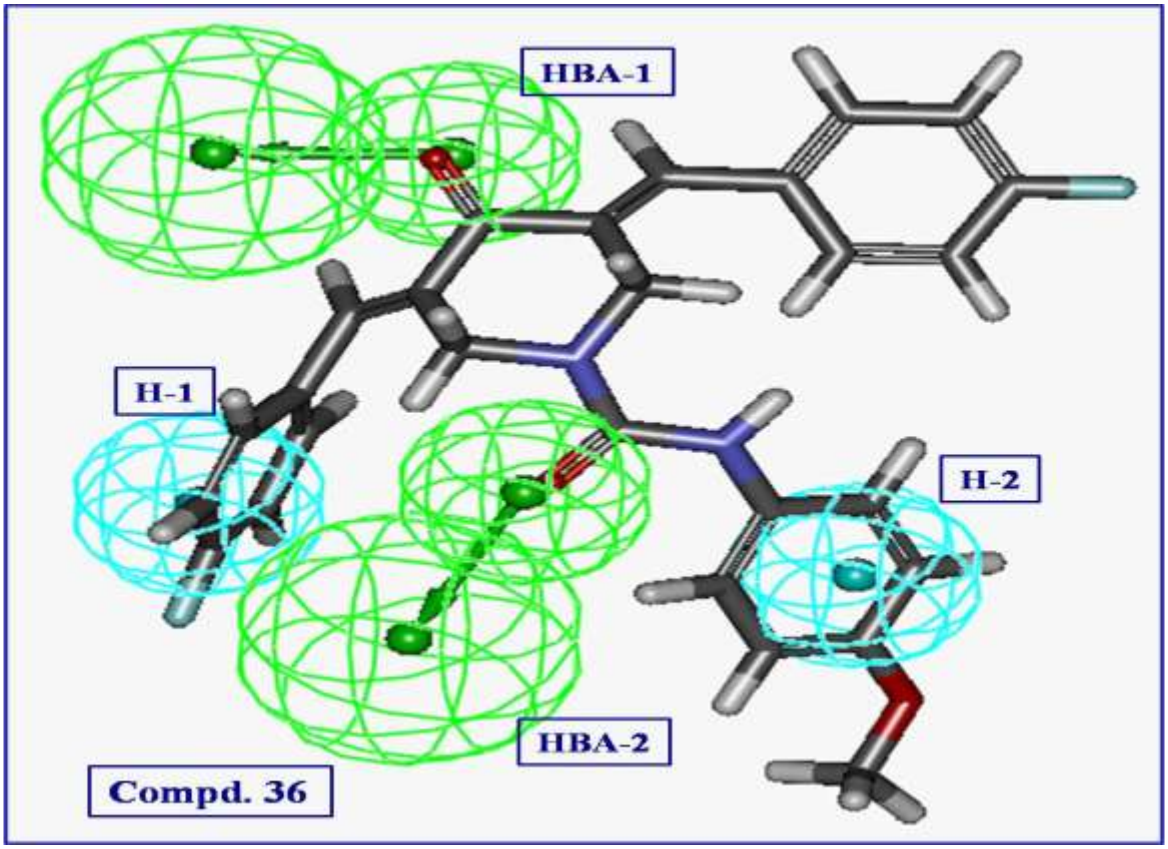
HBA-1
H-1
H-2
HBA-2
Compd. 36

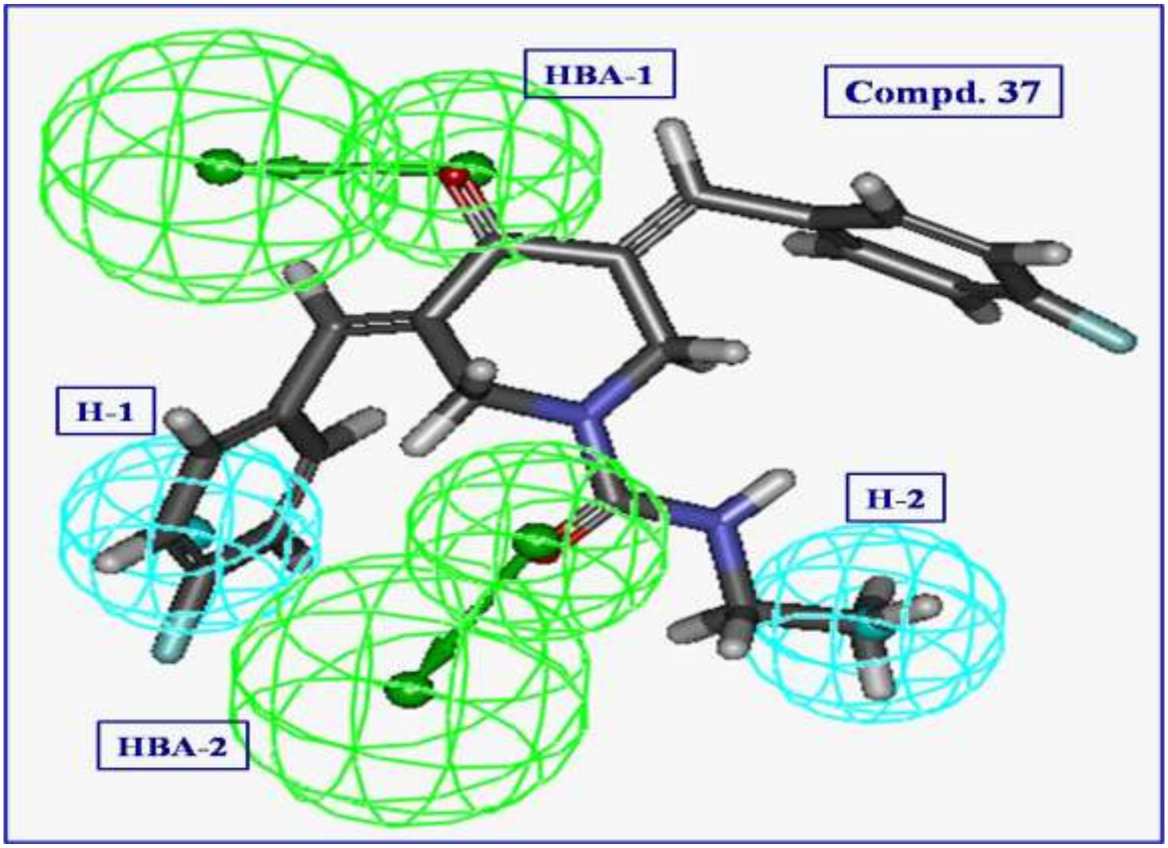
HBA-1
Compd. 37
H-1
H-2
HBA-2

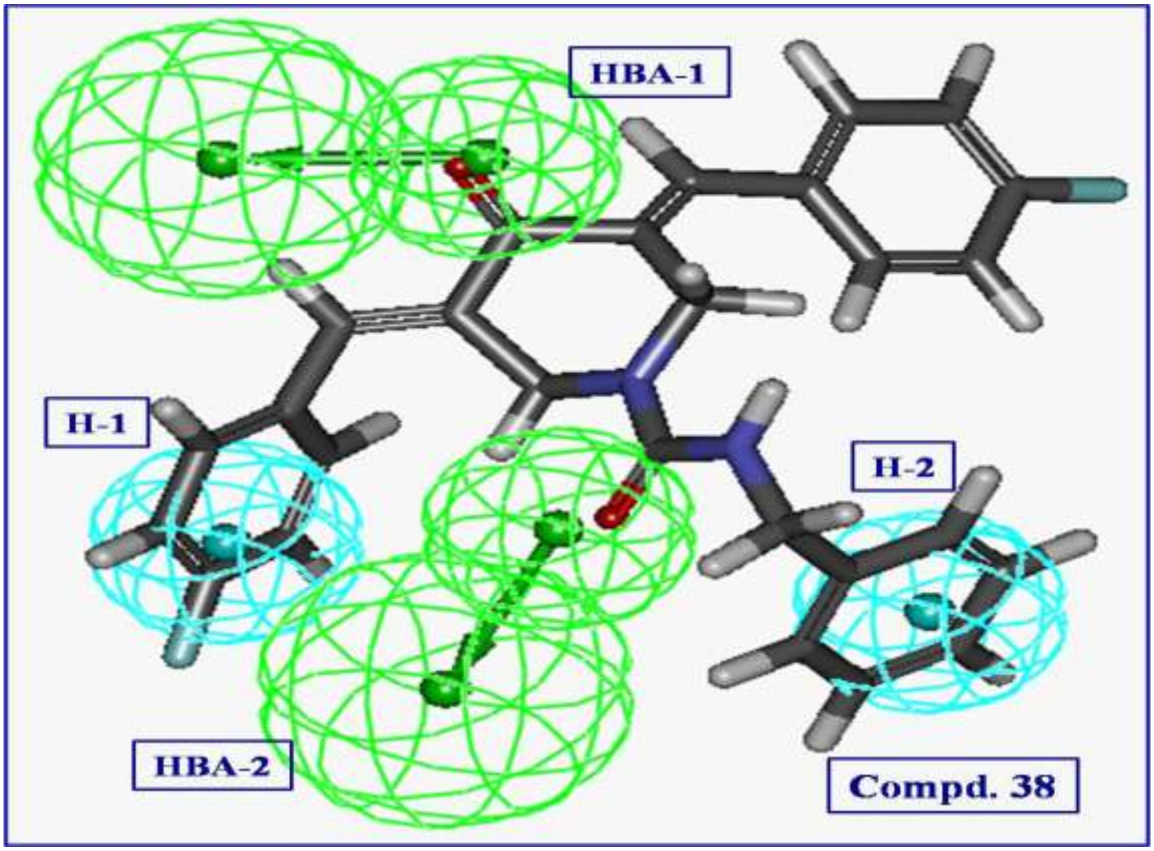
HBA-1
H-1
H-2
HBA-2
Compd. 38

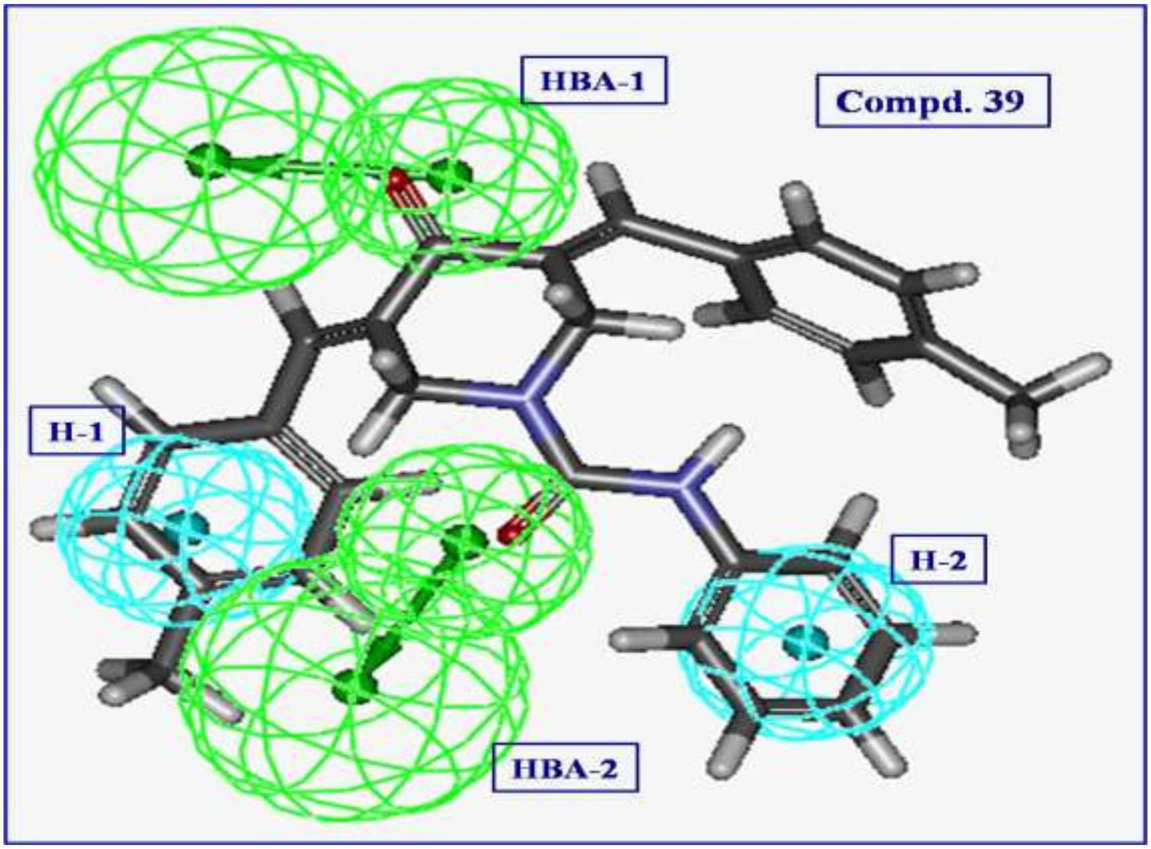
HBA-1
Compd. 39
H-1
H-2
HBA-2

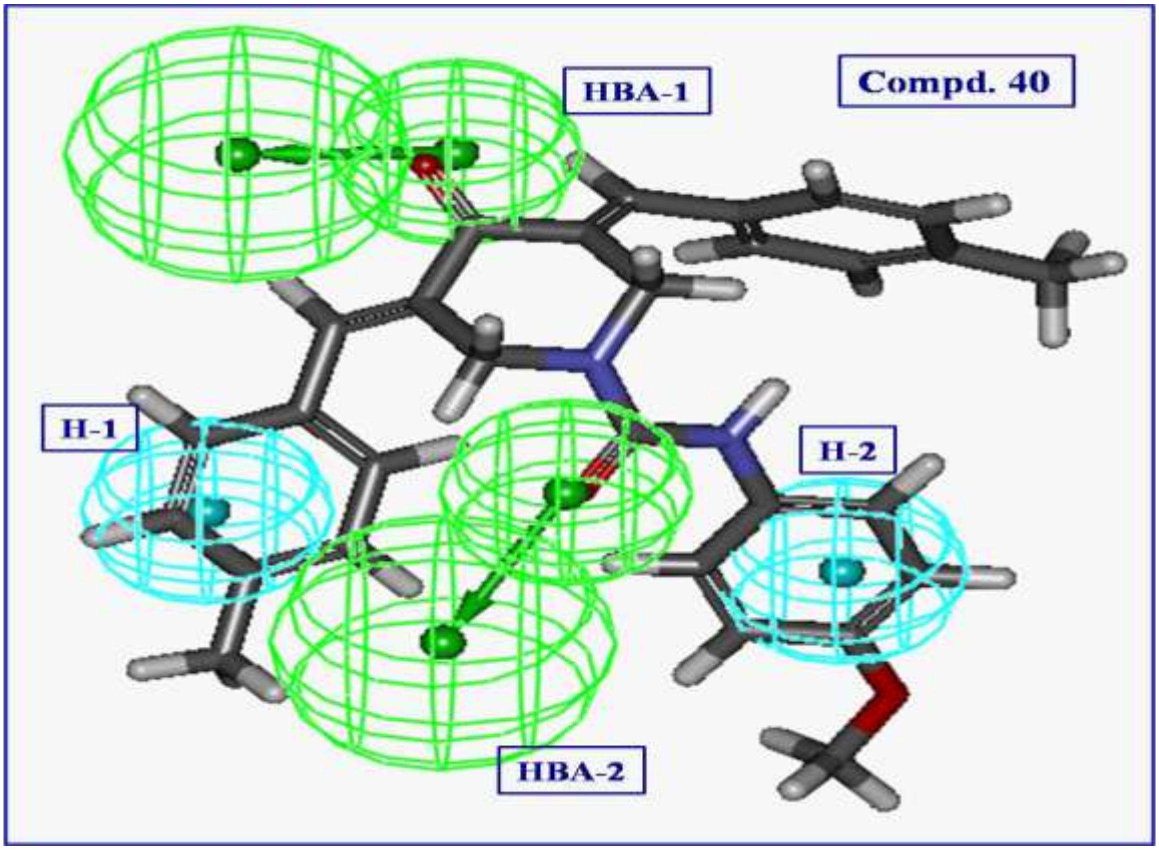
HBA-1
Compd. 40
H-1
H-2
HBA-2

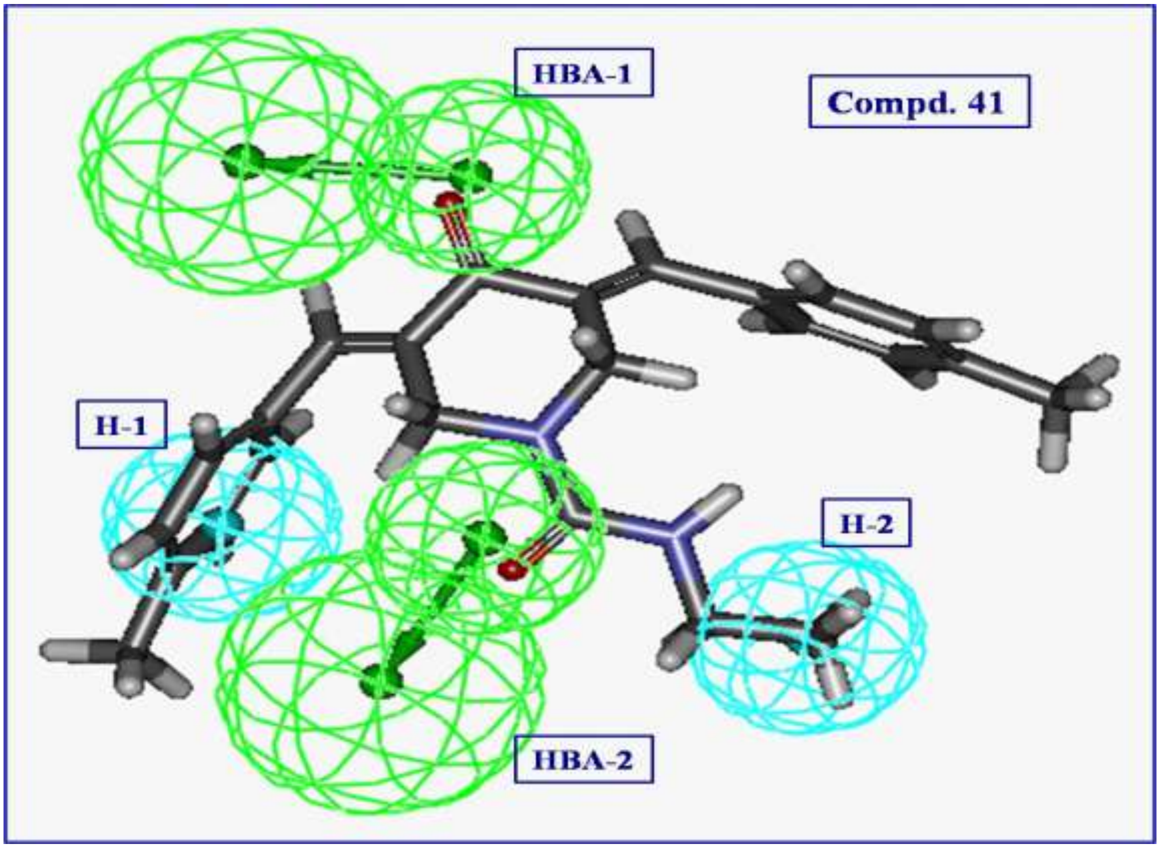
HBA-1
Compd. 41
H-1
H-2
HBA-2

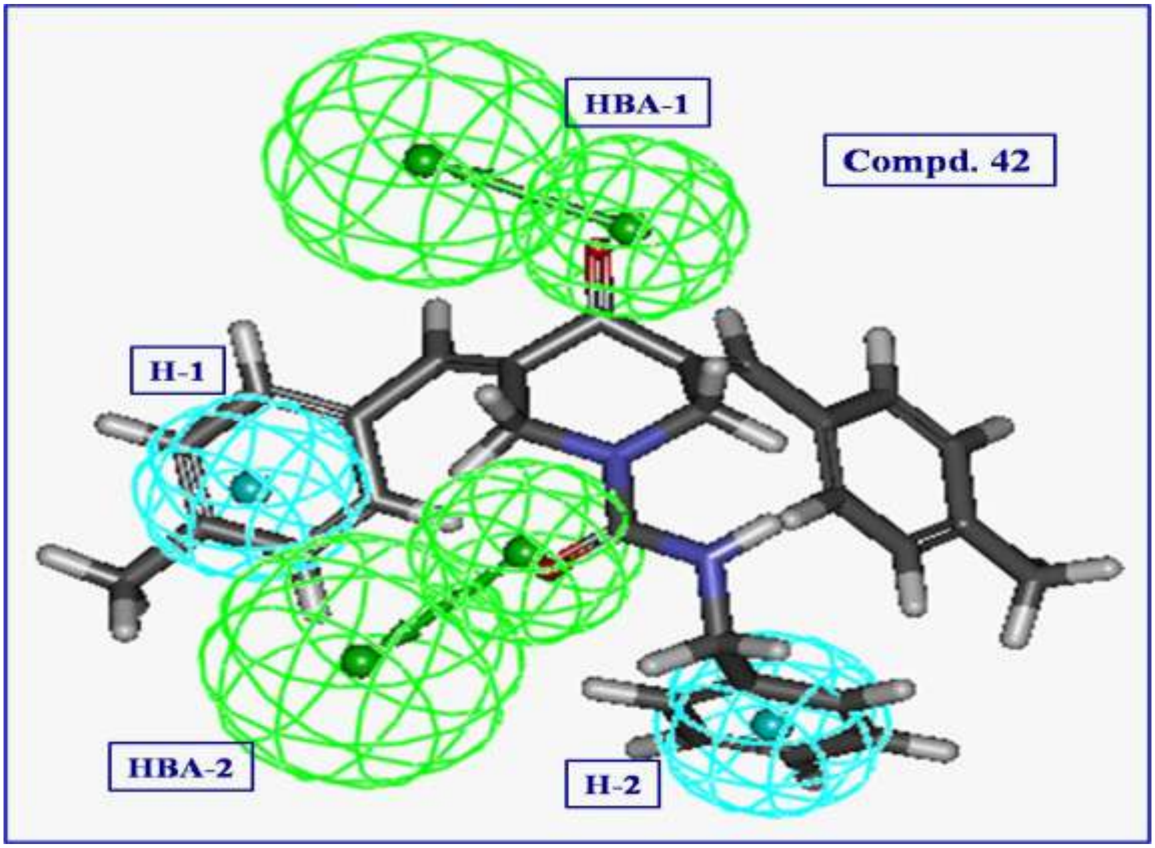
HBA-1
Compd. 42
H-1
HBA-2
H-2

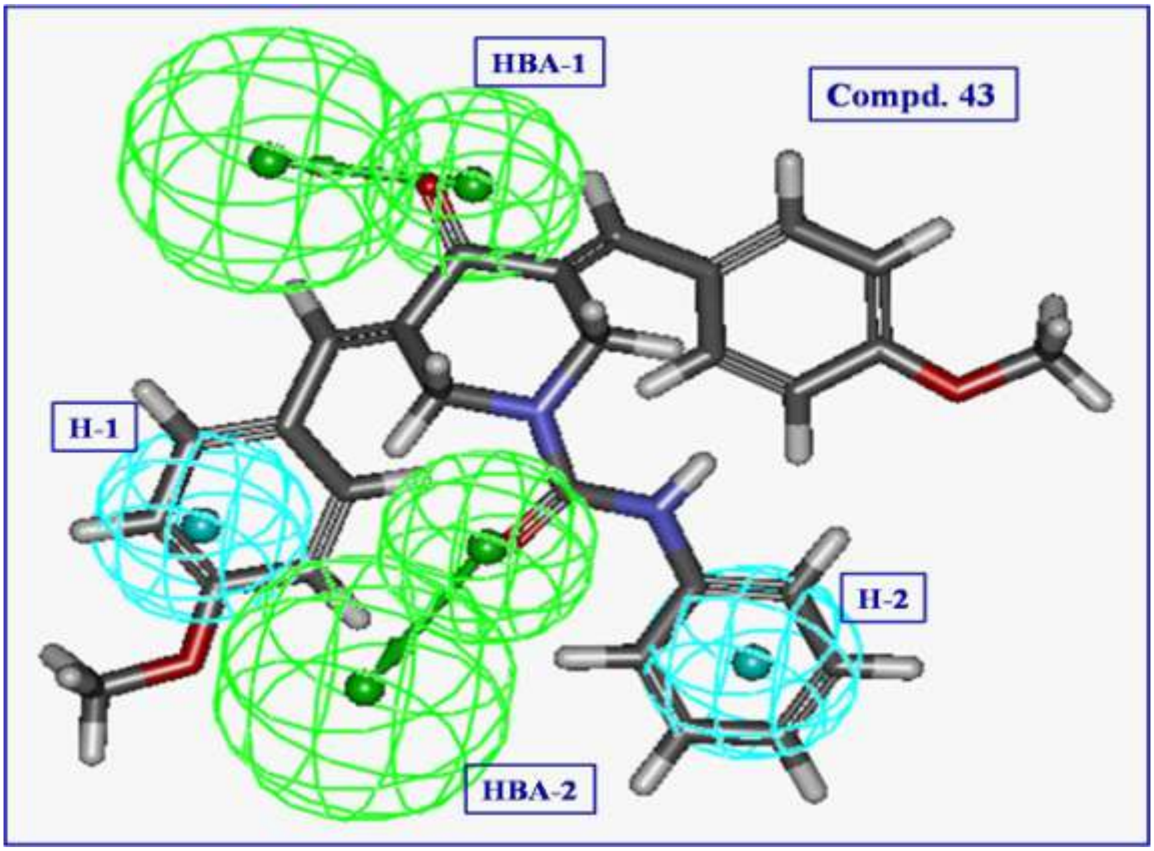
HBA-1
Compd. 43
H-1
H-2
HBA-2

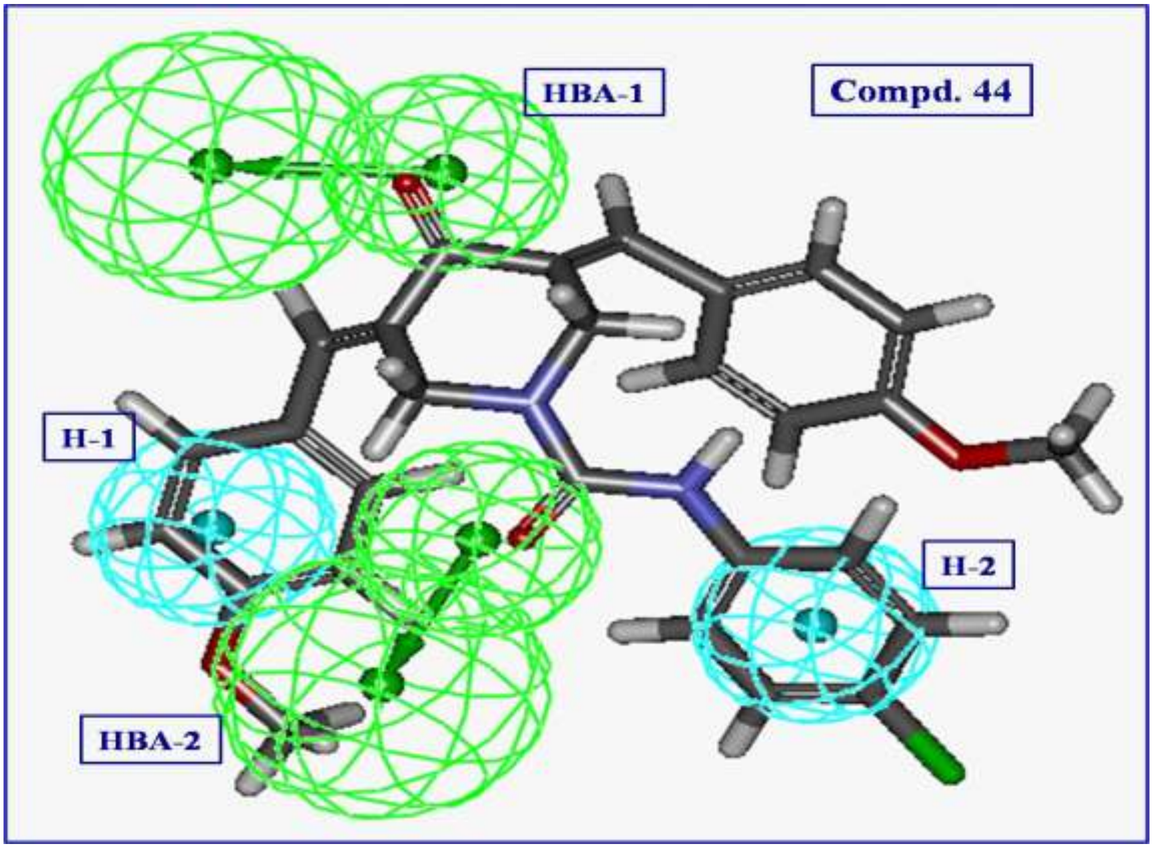
HBA-1
Compd. 44
H-1
H-2
HBA-2

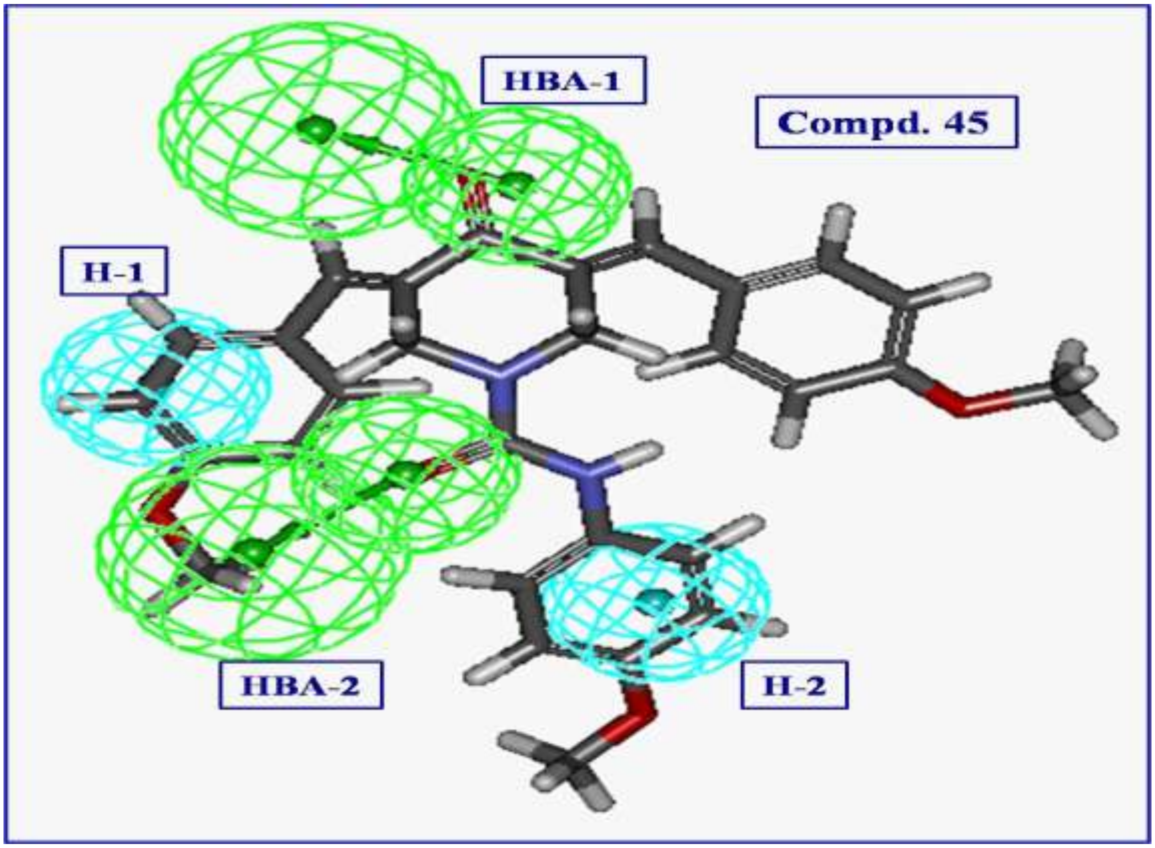
HBA-1
Compd. 45
H-1
HBA-2
H-2

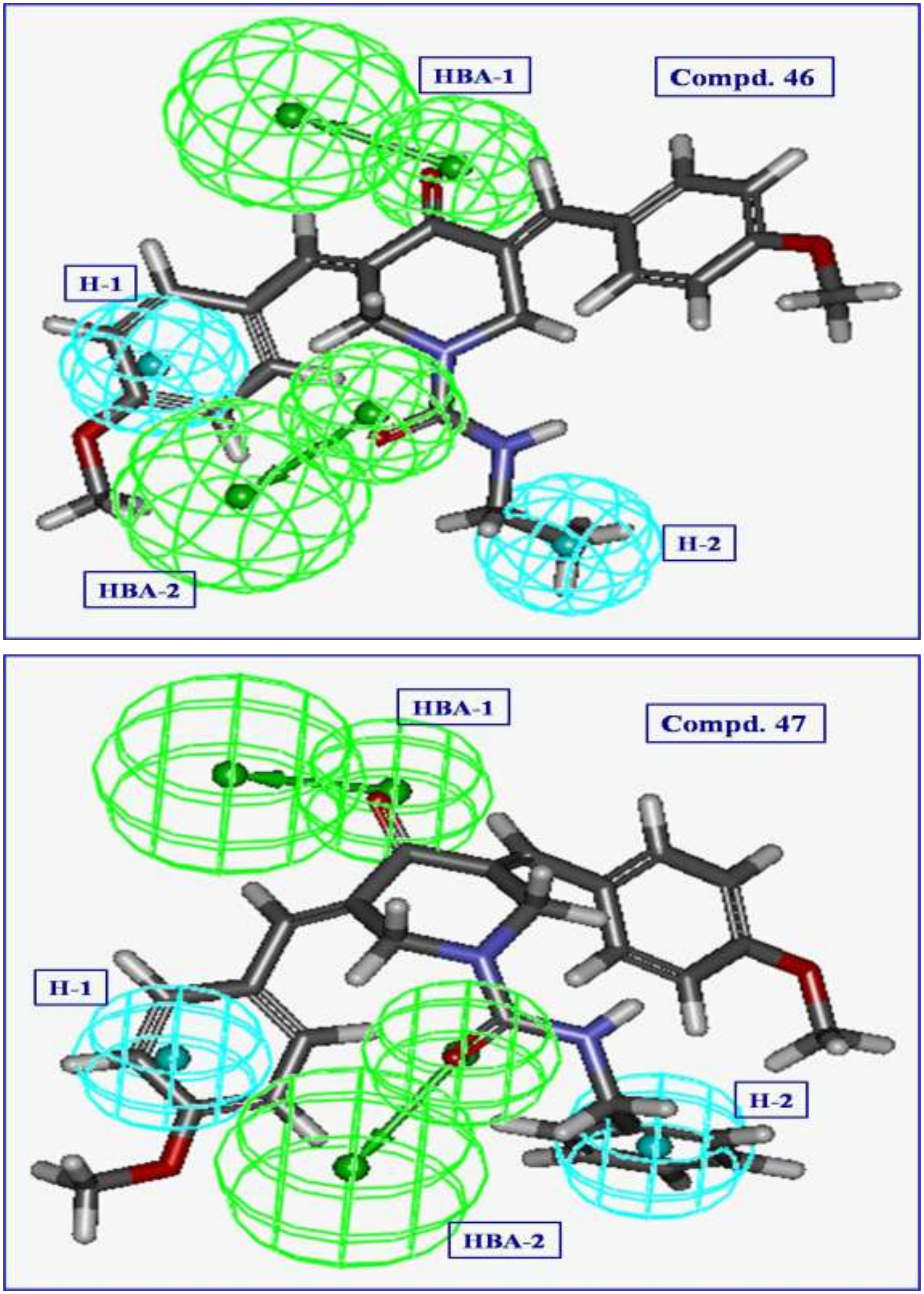

Fig. S108. 3D-pharmacophore model mapped on the tested piperidinecarboxamides **24–47** against A431 (squamous) carcinoma cell line.
